# Hydroxy acid conjugation to lipids increases structural and hydrolytic stability

**DOI:** 10.1038/s41467-026-75192-5

**Published:** 2026-07-27

**Authors:** R. Edri, S. Fisher, Y. Levi-Kalisman, M. Frenkel-Pinter

**Affiliations:** 1 Institute of Chemistry, The Hebrew University of Jerusalem, Jerusalem, Israel; 2 The Center for Nanoscience and Nanotechnology, The Hebrew University of Jerusalem, Jerusalem, Israel; 3 Casali Center of Applied Chemistry, The Hebrew University of Jerusalem, Jerusalem, Israel

**Keywords:** Origin of life, Fatty acids, Biophysical chemistry

## Abstract

Contemporary life requires functional polymers and phospholipid-derived compartmentalization. Mutualistic relationships between compartmentalization and polymerization have never been demonstrated in an abiotic scenario. As both the polymerization of hydroxy acids and the primitive cell-like aggregation of short-chain fatty acids are well known, we study cooperative interactions between compartmentalization and polymerization using these two classes of molecules as a model system. To that end, we explore the formation of various hydroxy acid-fatty acid conjugates. All reactions produce two types of condensation products: hydroxy acid oligoesters and lipid-conjugated oligoesters. We find that conjugation of hydroxy acids to fatty acids leads to the reduction of fatty acid critical aggregation concentration by an order of magnitude. Furthermore, hydroxy acid oligomers are protected against hydrolysis only upon conjugation to fatty acids. Our work offers meaningful insights into the role of self-assembly and cooperative chemistry as selective driving forces in lipid-polymer co-evolution.

## Abstract (web summary)

Contemporary life requires functional polymers and lipid-based compartmentalization, but mutualistic relationships between the two remain poorly understood in a prebiotic scenario. Here, the authors investigate cooperative interactions between fatty acid compartmentalization and hydroxy acid oligomerization as a model prebiotic system, and find that conjugation of hydroxy acids to fatty acids reduces the critical aggregation concentration of the amphiphiles, while protecting hydroxy acid oligomers against hydrolysis.

## Introduction

One of the puzzling questions in origins of life research is which pathways might allow a direct evolutionary linkage between compartmentalization and polymerization^1^. The existence of compartments is essential in promoting Darwinian evolution, forming microenvironments in which cellular chemistry can occur^2,3^. In the prebiotic context, compartmentalization offers physical mechanisms to promote polymerization by localizing different building blocks at high concentration and in close proximity. Compartmentalization can also hinder polymer hydrolysis, which is thermodynamically favorable in aqueous environments. This effect can be achieved, for example, when molecules partition to, or are shielded by, the membrane interface, or when the compartment lumen differs from the bulk solution in pH or ionic strength^4–10^. In modern biology, the role of compartmentalization is dominantly played by phospholipids^11^, which are the key building blocks of cell membranes, although other mechanisms of sub-cellular compartmentalization exist, such as membraneless liquid-liquid phase separation condensates^12^. Membranes formed by the self-aggregation of phospholipids and other contemporary lipids possess low permeability and physical durability, allowing the membranes to act as selective barriers^13^. Such properties, while desirable from a biological perspective, may raise difficulties in the context of prebiotic environments, wherein greater diffusion of small molecules through the membrane or other transport mechanisms were likely necessary in the absence of highly evolved specific membrane transporters^14^.

Phospholipids are complex amphiphiles and likely emerged at later stages of chemical evolution, although several prebiotic routes for their synthesis have been suggested^15–19^. The core building blocks of phospholipids, fatty acids, are found in model prebiotic reactions via Fischer–Tropsch type synthesis^20–23^ and are abundant in meteorites^23–26^, although they are considerably shorter than the fatty acids found in contemporary phospholipids. While most abundant prebiotic fatty acids are commonly short (up to C12-C15)^26,27^, contemporary phospholipids contain long-chain acids (typically C16-C26)^28^. Fatty acids are perhaps the simplest model of protocell building blocks^29^, and their self-assembly into vesicles has been thoroughly studied^7,30–32^. By themselves, fatty acids suffer from poor physical stability, which may affect their robustness as protocells. However, this fragility can be attenuated under certain conditions, such as in the gel phase^33^ or through cooperative aggregation in the presence of other building blocks that help to stabilize fatty acid vesicles^20,34^. For example, the presence of even small amounts of fatty alcohols, mono-acyl glycerides, and alkylamines reduces the critical vesicle concentration (CVC) and stabilizes decanoic acid vesicles at a wider range of pH conditions and over a greater range of salt concentrations^31,35,36^. Amino acids, peptides, nucleobases, and sugars also stabilize decanoic acid vesicles via noncovalent interactions^37–41^. Other non-covalent and covalent associations of lipids have been reported^42–46^, providing evolutionary advantages and underlining the importance of cooperation between different classes of chemical species during molecular evolution^33,34,47^.

The molecular diversity on Early Earth was immense, and included various organic molecules that were either delivered exogenously by meteorites or produced from fundamental building blocks in situ^25,48–52^. In the context of lipids, such diversity can assist in stabilizing lipid vesicles non-covalently, but also holds the potential path towards the formation of more complex and robust amphiphiles. Hydroxy acids (HAs) are highly attractive prebiotic molecules in the context of origins of life; they serve as metabolites in extant biology, exhibit high reactivity, and play a catalytic role in prebiotic peptide synthesis^53^. HAs such as glycolic acid and lactic acid have been found to facilitate peptide bond formation via ester-amide exchange under wet-dry cycling and drying reactions^54,55^. HAs have been suggested as key non-biomolecular players in prebiotic chemistry, and the polymerization and aggregation properties of their resulting oligoesters have been thoroughly studied^56–63^.

All biopolymers result from condensation-dehydration reactions and can hydrolyze in water^47,64^. Hence, in a prebiotic context, formation of polymers by itself is insufficient, and strategies to impart hydrolytic stability are perhaps of equivalent importance. Therefore, it is important to unravel plausible molecular mechanisms that enable simultaneous polymerization, hindered hydrolysis, and compartmentalization.

Here, we investigate whether simple reactions between prebiotically plausible fatty acids and hydroxy acids can generate molecular systems in which oligomerization, protection from hydrolysis, and compartmentalization become directly coupled. To that end, we investigated the formation of prebiotic amphiphiles obtained by reacting decanoic acid (DA) and additional fatty acids with four different HAs. Under simple drying conditions, we were able to produce a wide variety of lipid products that were characterized via various analytical and structural techniques. Our results indicate that DA is mostly reactive towards lactic acid, although lipid-conjugated oligoesters comprised of DA and HAs were obtained for all tested HAs. We then asked whether these conjugates alter properties that could matter functionally in prebiotic settings. Specifically, we examined how conjugation affects amphiphile assembly, permeability-related behavior, and resistance of hydroxy acid esters to hydrolysis. Our results indicate synergistic interactions and pure molecular mutualism, where, on one hand, conjugation of hydroxy acids to lipids promoted greater self-assembly propensity of the lipids, and on the other hand, this conjugation protected hydroxy acid esters from hydrolysis. Moreover, the resulting lipid-conjugated oligoesters exhibited greater permeability compared to unreacted DA. Together, these results identify lipid-conjugated oligoesters as a model system in which covalent coupling between two molecular classes gives rise to emergent physicochemical consequences and highlight the importance of cooperative interactions between different classes of molecules in shaping early chemical evolution.

## Results and discussion

### Drying reactions between decanoic acid and hydroxy acids

**Fig. 1:**
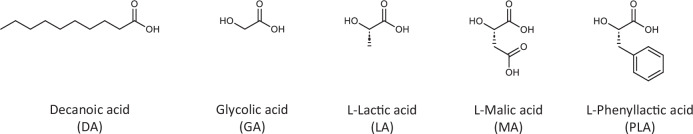
Chemical structure and abbreviations of the studied monomers. Mixtures of decanoic acid with each of the four alpha hydroxy acids were allowed to react at 85 °C for 7 days under dry conditions to produce hydroxy acid oligoesters or lipid-conjugated oligoesters.

**Fig. 2:**
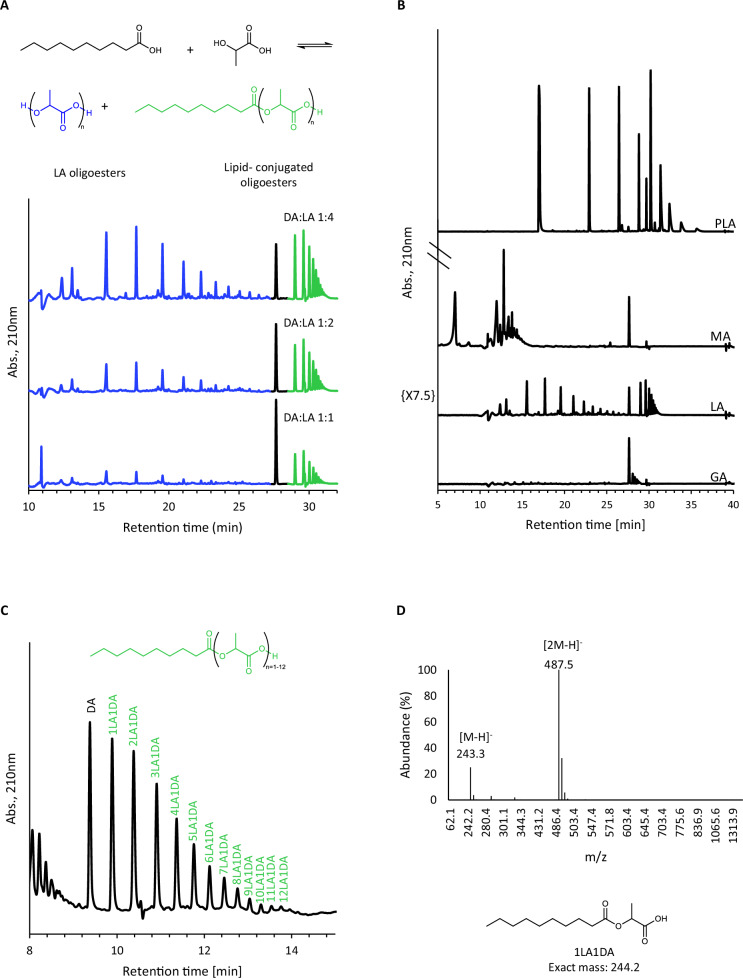
Amphiphiles derived from DA-HA conjugates were prebiotically synthesized under simple dry conditions. HPLC chromatograms were obtained for DA:LA reaction products at 1:1, 1:2, and 1:4 molar ratios. Two types of products, including LA oligoesters and DA-LA conjugated oligoesters were formed (**A**). HPLC chromatograms were obtained for DA:HA reaction products at a 1:4 molar ratio (DA:HA) for the four tested HAs (**B**). HPLC chromatogram obtained for DA:LA reaction product at a 1:4 molar ratio. The labeled signals are lipid-conjugated oligoesters of DA-LA_n_ with up to 12LA-mer conjugated to DA (**C**). MS spectrum (negative mode) was obtained for the identification of the 1LA1DA reaction product. The obtained m/z values correspond to [M-H]^-^ and [2M-H]^-^ ions (**D**). Source data are provided as a Source Data file.

We investigated esterification reactions between decanoic acid (10-carbon long fatty acid, DA) and four different HAs: glycolic acid (GA), L-lactic acid (LA), L-malic acid (MA), and L-phenyllactic acid (PLA) (Fig. 1), to produce the *O*-acylhydroxy acids. Decanoic acid and single HAs (either GA, LA, MA, or PLA) were allowed to react under dry conditions in the absence of water, other than water originating in the raw materials for 7 days at 85 °C at a 1:1, 1:2, and 1:4 molar ratio (in favor of the HAs). Following the reaction, we analyzed the resulting products via LC-UV-MS, FTIR, and NMR. We found that all four HAs reacted to produce either hydroxy acid oligoesters (oligomers of pure HAs) or lipid-conjugated oligoesters containing oligoesters covalently bound to DA (Fig. 2A, B). For all HAs tested, the DA-HAs conjugates contained one DA and n-HAs, as expected. Typically, the number of products and their concentration (both hydroxy acid oligoesters and lipid-conjugated oligoesters) increased as the amount of the HA increased, with the exception of PLA, for which no significant differences were observed as the molar ratio increased (Figs. S1–S21). When examining the effect of DA:LA molar ratio on DA conversion across different batch sizes, we observed similar trends for 200 and 400 μmol DA, with conversion increasing as the LA fraction increases (Figs. S22–23). However, for 800 μmol DA, conversion reaches a plateau at a 1:2 molar ratio (DA:LA).

The identification of oligomer length and composition was achieved using LC-MS (Fig. 2C, D, Figs. S24–S157, Tables S1–S9). Out of the four HAs tested, we found that LA produced the longest lipid-conjugated oligoesters (12LA1DA), while GA, PLA, and MA produced similar length oligomers of up to 5–6 HA (Table 1). As for hydroxy acid oligoesters, MA produced the longest oligomers (16MA) and LA, GA, and PLA produced up to 13-,12-, and 7-mer, respectively. Interestingly, in the absence of DA, LA oligomerized to form products of up to 18-mer and possibly longer species (Tables S4–S6, Figs. S74–S126), suggesting that the formation of DA-LA conjugated oligoesters came at the expense of LA oligoesters. The oligomerization degree of the HAs formed in the absence of DA is in good agreement with previous studies, although we observed deviations in the maximum oligomer length detected, likely due to differences in analytical methodology^61,65^. The formation of the esterification products was further confirmed by FTIR (Figs. S158–S166) and NMR measurements (Figs. S167–S187). A shift in the carbonyl stretching in FTIR and downfield shifts of the alpha protons in NMR indicated that ester bonds were formed in all tested mixtures upon drying.

**Table 1:** The extent of oligomerization of both hydroxy acid oligoesters and lipid-conjugated oligoesters in the absence and presence of decanoic acid (DA)

	In the absence of DA	In the presence of DA	
Hydroxy acid	Hydroxy acid oligoesters	Hydroxy acid oligoesters	Lipid-conjugated oligoesters
Glycolic acid	14-mer	12-mer	6-mer
Lactic acid	18-mer	13-mer	12-mer
Malic acid	17-mer	16-mer	5-mer
Phenyllactic acid	7-mer	7-mer	6-mer

We measured the conversion of DA into products in the various reactions via HPLC and found that the highest consumption was observed when DA reacted with LA, followed by PLA, GA, and MA. DA conversion was dependent on the DA:HA ratio: as the ratio increased, the DA conversion also increased. For instance, the consumption of DA when reacted with GA at a 1:1 molar ratio was 18 ± 2%, while at a 1:4 molar ratio the conversion was 24 ± 3%. One exception was the case of MA, for which DA conversion was constant regardless of the amount of MA, at around 15%. The highest conversion of DA was observed for reactions with LA, with a 29 ± 1%, a 40 ± 2%, and a 54 ± 1% conversion at 1:1, 1:2, and 1:4 molar ratios, respectively (Fig. S188).

The reactivity of DA towards esterification seemed to be dictated mostly by the chemical nature of the HA. Less hydrophilic HAs such as LA and PLA (logP of −0.72 and 1.01, respectively) appeared to facilitate the conversion of DA with up to 54% and 32%. On the other hand, the more hydrophilic HAs, GA and MA (logP of −1.1), reacted with DA to a lesser extent. Other factors, such as steric hindrance and acid strength, which were found to affect the consumption of amino acids in depsipeptide formation^30,54,66^, were relatively negligible. An additional factor that may affect the extent to which DA reacted with HAs is the physical state of the HA under reaction conditions. Among the four HAs, GA, MA, and PLA were introduced as solids, while LA was a concentrated solution. Under reaction conditions, GA remained in the liquid state as well as LA. However, MA and PLA remained in the solid state and therefore their mobility was lower, in accordance with their higher melting points (~135 °C and ~121 °C, respectively). While the physical state in the case of MA did not prevent homo-oligomerization of MA, it may inhibit PLA homo-oligomerization, as indicated in the control PLA sample (Fig. S17). Remarkably, the conversion of PLA and the formation of the PLA oligoesters in the presence of DA were significantly greater than the control sample (in the absence of DA) (Fig. S189–190). A possible explanation for the positive effect of DA on PLA oligomerization may suggest that DA serves as a molten hydrophobic medium, allowing partial solubility of PLA; consequently, PLA molecules became more mobile and thus reacted to a greater extent. It is possible that the oligomerization may have occurred at the solid-liquid interface formed by both PLA and DA molecules.

### Structural characterization of the reaction products

Subsequently, we sought to study the assembly profile of DA-HA reaction products and observed distinct structural features in all reaction products. Decanoic acid is well known to form different types of structures depending on the pH, temperature, and concentration^30,66,67^. At pH levels around DA pK_a_ (~7), where the molar ratio between the protonated decanoic acid and the charged, deprotonated decanoate species is about 1:1, vesicles form (commonly achieved within the range of pH 6.6–8.0)^68^. At higher pH levels, at which most of the DA is in the deprotonated form (i.e., decanoate anions), micelles form. At pH levels much lower than the pK_a_, most of the decanoic acid is in the neutral protonated form and forms either oil droplets or solid precipitates, depending on temperature^67^. We sought to study the complex reaction mixture as a whole rather than isolated reaction products, in accordance with recent studies in systems chemistry^69–73^. We expected that the physical properties of the system will be affected by interconnected interactions between all components within the system. To study how the presence of DA-HA reaction products affects the structure formation, we rehydrated the reaction products of DA and each of the four HAs in a 50 mM phosphate buffer and adjusted the pH to 6.8, at which vesicles are expected to form. The concentrations of DA and HAs were 50 mM and either 50, 100, or 200 mM, respectively, referring to the initial amounts prior to the reaction. The obtained samples were visually inspected and characterized by turbidity measurements and microscopy. As indicated by both turbidity measurements and visual inspection, most of the samples exhibited turbidity to some extent, suggesting that structures formed upon the rehydration process (Figs. S191–S192). The specific structural characteristics of these assemblies were characterized as shown below using fluorescence microscopy, bright-field microscopy, dynamic light scattering, and cryo-TEM.

Turbidity correlates with the light scattered from the sample, which depends on the structures present in the sample. Accordingly, turbidity measurements give a good indication of both structure size and distribution^74,75^. There was no clear correlation between turbidity and the DA:HA molar ratio; indeed, each HA exhibited different behavior. For instance, while no significant differences in turbidity were observed for GA, in the case of LA, turbidity significantly decreased as the ratio increased in favor of LA. We further confirmed structure formation under a fluorescent microscope using rhodamine 6 G dye. The resulting rehydrated products consisted of DA monomer at concentrations varying from 23 to 43 mM (Fig. S193), which is above DA’s critical vesicle concentration (CVC) at pH 6.8 (10–12 mM)^7^. Indeed, under the microscope, we were able to observe structures with distinct vesicle bilayers for most of the samples (Fig. S194). However, the lipid bilayer was not clearly visualized for all samples, for example in the case of DA-LA products. As expected, an abundance of giant vesicles of several microns in diameter correlated with increased turbidity of the samples. Samples of lower turbidity were visualized under a fluorescent microscope, but no clear image of their bilayer membrane was observed.

**Fig. 3:**
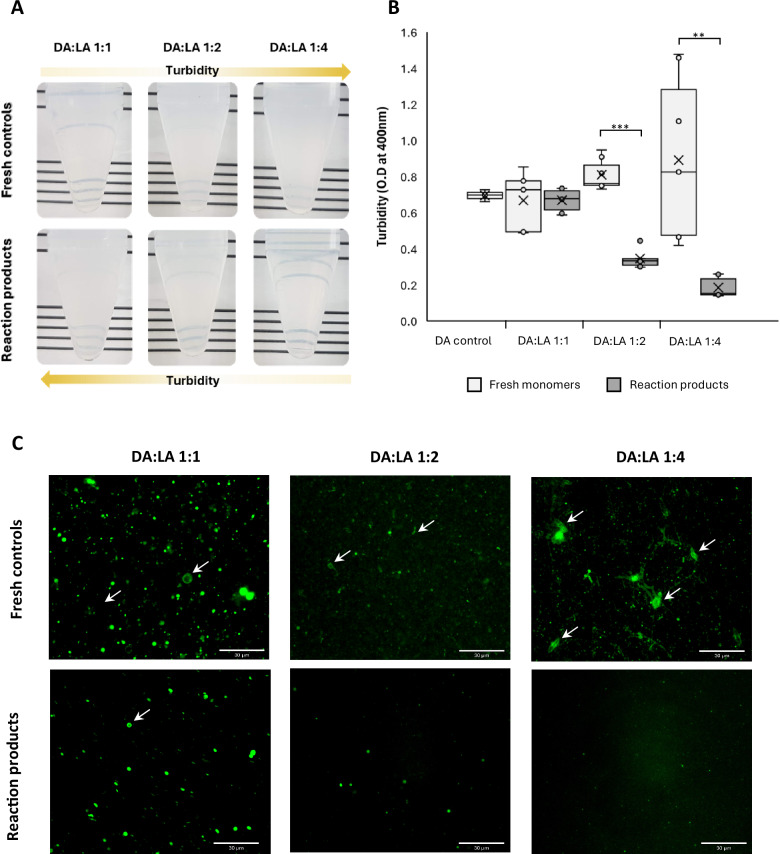
Characterization of DA-LA mixtures reveals structural differences between fresh monomers and reaction products. All samples were prepared in a phosphate buffer (50 mM) at pH 6.8. The concentration of DA was 50 mM, and the concentration of LA was either 50, 100, or 200 mM. In the case of the reaction products, DA and LA concentrations refer to the initial amounts prior to the reaction. Visual appearance of DA-LA mixtures of fresh monomers (top) or reaction products (bottom) (**A**). Turbidity (measured as the optical density at 400 nm) of DA-LA mixtures of fresh monomers (light gray boxes) and reaction products (dark gray boxes). As indicated by the turbidity measurements, opposing trends were observed for the fresh monomers and the reaction products. While an increase in LA concentration resulted in increased turbidity in the case of the fresh monomers (not statistically significant), the turbidity significantly decreased in the case of the reaction products (*p* < 0.001). The effects were not significant for DA:LA 1:1 molar ratio, however at 1:2 and 1:4 the differences in turbidity were significant (*p* < 0.001 and *p* < 0.05 respectively). T-test was used to determine statistical significance. Box plots represent the distribution of values across replicates (**B**). Fluorescent microscopy images (x60 magnification) of DA-LA fresh monomers (top) or reaction products (bottom). Rhodamine 6 G was used as a fluorescent probe (1 mM). Structures were formed in all tested samples, as indicated by the images. Vesicles, indicated by the staining of the membrane (labeled with white arrows), were detected in all samples with the exception of DA:LA reaction products at 1:2 and 1:4 molar ratios, for which no clear images of the membrane were detected (**C**). Source data are provided as a Source Data file.

The particular case of DA-LA mixture is distinct within the scope of the experiment in both chemical and structural perspectives. Therefore, we conducted a more detailed characterization of this system. To understand how hydroxy acid oligoester and lipid-conjugated oligoester formation affected the observed structure formation, we prepared control samples of DA and LA fresh monomers at 1:1, 1:2, and 1:4 molar ratios, fixing DA concentration at 50 mM. The control samples were further characterized by turbidity measurements and fluorescence microscopy (Fig. 3). In the case of the control monomer samples, as the DA:LA ratio increased in favor of LA, the turbidity of the samples increased (Fig. 3A, B). Under the microscope, vesicles were clearly observed for all samples. However, the abundance of larger and deformed vesicles positively correlated with high LA concentrations. When comparing these observations to the reaction products, the opposite trend is observed. Namely, at a lower DA:LA molar ratio, the turbidity of the samples increased, and larger structures were detected under the microscope. Yet, as the ratio increased to 1:2 and 1:4 in favor of LA, the turbidity of the reaction products significantly decreased and fewer structures of larger size were observed under the microscope (Fig. 3C). Since the concentration of monomeric DA decreased significantly in the reaction, we further prepared control samples consisting of DA at the concentration measured in the reaction products. Indeed, turbidity was slightly lower as DA concentration decreased. However, the abundance of structures was relatively high compared to the reaction products, mostly the ones for DA:LA at 1:2 and 1:4 molar ratios (Figs. S195–S196). These results suggest that the self-assembly properties of the systems were affected by the presence of the new products.

The reaction products were composed of two types of oligomers, LA oligoesters and DA-LA conjugated oligoesters, leading us to examine the individual contribution of each type of product to the observed assemblies. For that purpose, we prepared a mixture of DA and LA hydroxy acid oligoesters (obtained by drying LA in the absence of DA) by rehydrating the LA reaction product in the presence of 50 mM DA at pH 6.8. As a control, we also rehydrated LA reaction products in the absence of DA. We found that both samples were turbid to some extent, while in the absence of DA, the LA esters exhibited significant inhomogeneity. Under a fluorescent microscope, the samples consisting of DA and LA oligoesters without DA-LA conjugated oligoesters were found to form vesicles of various sizes for which the membrane was visualized. Aggregates related to LA oligomers were also observed (Fig. S197). Throughout this work, we use the term ‘aggregates’ broadly to refer to any form of molecular association (e.g., clustering, phase-separated domains, vesicles), without implying a particular morphology or degree of structural order. Interestingly, the samples in the absence of DA formed aggregates that were not dyed by rhodamine 6 G. This observation highlights the fact that DA molecules tend to be adsorbed at the surface of LA aggregates, resulting in their dying by rhodamine 6 G. The absence of LA aggregates in the combined DA-LA reaction products might be due to the presence of DA-LA conjugates that interfere with LA oligomer aggregation. Alternatively, aggregates of LA oligomers may be formed only for minimum oligomer length. As mentioned previously, LA oligomerizes to form shorter hydroxy acid oligoesters of up to 13-mer in the presence of DA (Table S4, Figs. S74–S91). However, in the absence of DA, LA oligomers reached 18-mer (Table S6, Figs. S104–S126). It may be possible that only oligomers of the longest chain length form such aggregates. While LA-derived oligoesters have been reported to form microdroplets when dispersed in water-acetonitrile mixtures^56,59,65^, we did not observe microdroplet formation under our conditions, likely due to differences in sample preparation. Nonetheless, further work is needed to explore possible co-existence of lipidic structures such as vesicles and HAs microdroplets. Recently, non-covalent interactions between phospholipids and HA assemblies were reported by Jia and colleagues, in which the assembly of phospholipids around polyphenyllactate droplets has been observed^76^. Moreover, several works studying the interactions of coacervates and lipids have shown that coacervate-containing vesicles enhance both permeability and encapsulation of hydrophilic components, contribute to vesicle uniformity, and alter physicochemical properties of the lipid vesicles^77–79^. Hierarchical compartmentalization and multi-compartment systems are an essential property of living organisms as they construct a complex network of interconnected environments and promote segregated processes^80–82^. Accordingly, concomitant formation of vesicle and microdroplet from simple, prebiotically plausible molecules such as fatty acids and hydroxy acids could provide a tractable model for studying emergent, cell-like compartmentalization.

**Fig. 4:**
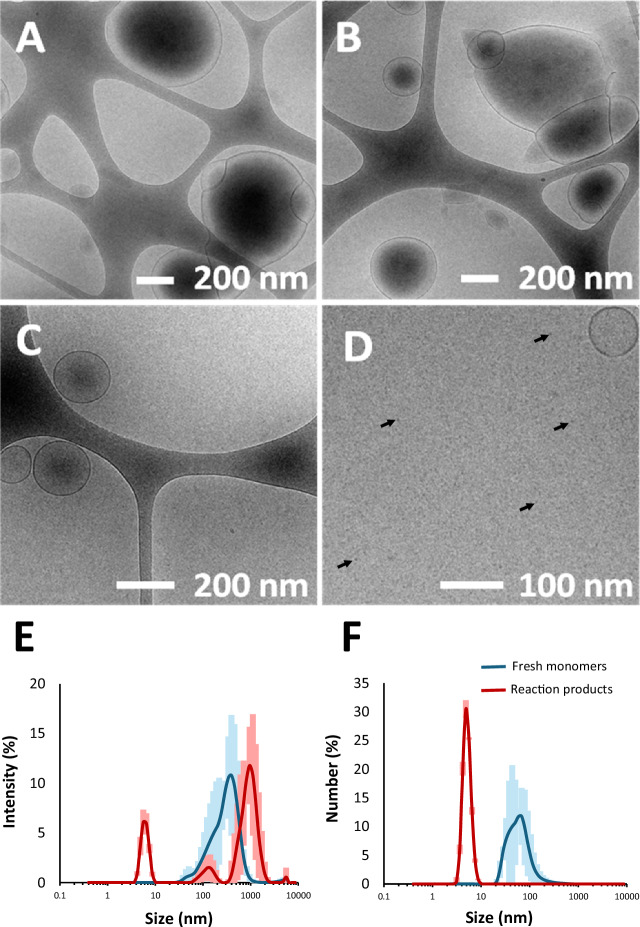
DA-LA reaction products and fresh monomers exhibit distinct assembly profiles. Cryo-TEM images of DA:LA fresh monomers (**A**, **B**) and reaction products (**C**, **D**) at a 1:4 molar ratio. Cryo-TEM images confirm the formation of spherical and non-spherical membranous structures in the fresh monomers. In the reaction products we observed vesicles that were almost exclusively of the unilamellar subtype, and mostly less than 200 nm in diameter. Many small dots (black arrows), mostly of about 4–6 nm, were also observed, consistent with micellar structures. DLS results (**E**, **F**): Intensity-weighted size distribution (**E**) and number-weighted size distribution (**F**) obtained for fresh monomers (blue line) and reaction products (red line) at DA:LA of a 1:4 molar ratio. DA and LA concentrations were 25 mM and 100 mM, respectively. Error bars represent SD of three independent preparations. Source data are provided as a Source Data file.

To further characterize the structures that were formed, we conducted cryogenic transmission electron microscopy (cryo-TEM) measurements for DA-LA reaction products and control samples at pH 6.8 (Fig. 4). In all the reaction products, vesicles were detected (Fig. S198). At a 1:1 DA:LA molar ratio, large vesicles of varied lamellarity (both unilamellar vesicles (ULVs) and multilamellar vesicles (MLVs)) were observed, similarly to DA control. As the DA:LA ratio decreased to 1:2 and 1:4, vesicle abundance decreased, consistent with the fluorescence microscopy results. In addition, the fraction of ULVs in both samples increased. Surprisingly, at a 1:4 molar ratio of DA:LA, we were able to detect small ULVs, as well as tiny structures (dark dots of about 4–6 nm), which we attribute to micelles (Fig. 4D). The formation of micelles at this pH is not related solely to DA but probably to either the reaction products of DA and LA or to the co-aggregation of both DA remaining monomers and its LA derivatives. Indeed, esters of LA and fatty acid salts have been reported to form micelles at concentration levels of 1–83 mM, depending on fatty acid chain length^83^. These products consist of an anionic, carboxylic acid head group with a pK_a_ lower than that of DA due to the adjacent ester group. Accordingly, at pH 6.8, most of the DA-LA conjugates are ionized and tend to form micelles rather than vesicles. The samples are composed of a mixture of at least 10 different amphiphiles, and accordingly, it is likely that co-aggregates of the different amphiphiles are present. The possible formation of micelles may be essential for promoting catalytic activity and inducing protocellular fission, as previous studies have shown^8,84,85^.

Vesicles were also observed in the control samples prepared by mixing DA and LA fresh monomers at similar molar ratios, however the nature of these vesicles differed (Fig. 4A, B). Overall, the vesicles observed for the fresh samples were more heterogeneous in terms of size and lamellarity. Remarkably, the shape of the vesicles gradually changed as the DA:LA ratio decreased to 1:2 and 1:4 (Fig. S198). While at a 1:1 molar ratio of DA:LA, most of the vesicles were spherical, at a 1:2 molar ratio some of the vesicles appeared partially deformed. This was much more prominent at a 1:4 molar ratio, at which ill-defined membranous structures were observed (Fig. 4A, B and Fig. S198). We cannot exclude the possibility that vesicle deformation observed under both fluorescence and electron microscopy might be due to the pressure and compression applied by the microscope cover slip and blotting step upon preparation^86–88^. Overall, the structural properties of the fresh monomers and the reaction products differed significantly, exhibiting different structures and varied size distribution.

While microscopic methods provide high-resolution information, it is on the local level. Bulk characterization is essential to gain more comprehensive understanding of the explored systems. For that purpose, dynamic light scattering (DLS) measurements were employed. DA control as well as DA-LA fresh monomers and reaction products at all tested molar ratios were measured with a back-scattering DLS instrument, and based on the autocorrelation function, the size and polydispersity were determined. For DA:LA systems at 1:1 and 1:2 molar ratios, the average size obtained by the cumulant algorithm (Z-ave) was significantly lower for the reaction products compared to the fresh monomers (Fig. S199–S200). For DA:LA 1:4 molar ratio samples, we used the size distribution profile obtained by the non-negative least square fit (Fig. 4E, F and Fig. S201) to compare the reaction products and fresh monomers. As indicated by Fig. 4E, in the case of the fresh monomers, the intensity-weighted distribution demonstrates a broad population of structures with a maximum at ca. 350 nm, while multiple populations were observed for the reaction products, as was also indicated in the correlograms (Fig. S199). Two of them are within the nanometric and micron size ranges with a maximum at about 5.6 nm and 950 nm, respectively. When translating the intensity-weighted to a number-weighted distribution (Fig. 4F), a clear single population of about 4–6 nm was observed for the reaction products, in agreement with cryo-TEM assessment of a micellar population. We complemented the DLS measurements with cryo-TEM–based vesicle size statistics (*n* ≥ 80 vesicles per sample from ≥3 grid regions; micelles were excluded), which supported a greater fraction of <100 nm vesicles in the reaction products, especially at a DA:LA 1:4 molar ratio, while measured micelle diameters (4–6 nm) agreed with DLS (Figs. S202–203).

**Fig. 5:**
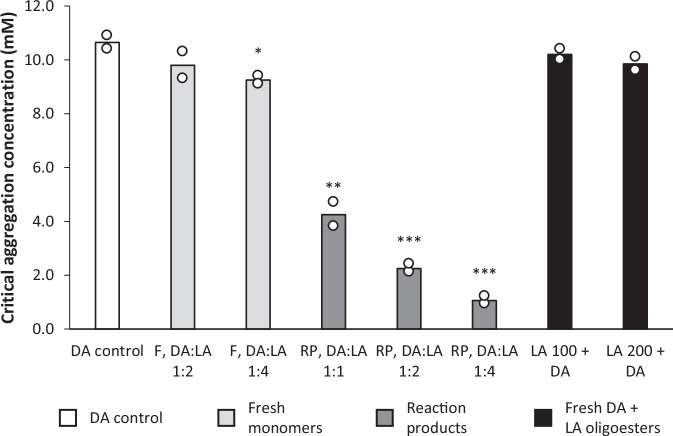
Critical aggregation concentration (CAC) of DA obtained for DA-LA fresh monomers (F, light gray columns), DA-LA reaction products (RP, dark gray columns), and for DA in the presence of LA oligomers (black columns). The oligomerization of DA into DA-(LA)_n_ conjugates resulted in the reduction of DA CAC at pH 6.8 by up to 10-fold. The presence of either LA monomers or LA oligomers had only a minor effect on the CAC of DA. All experiments were carried out in duplicates. Source data are provided as a Source Data file.

The DA-LA reaction products were found to have a tremendous impact on DA self-assembly properties, leading us to postulate that the conjugation of LA to DA will affect its effective aggregation concentration. To test this hypothesis, we measured the critical aggregation concentration (CAC) of DA or DA-LA reaction products at pH 6.8. We used merocyanine 540 (MC540) assay to determine the CAC of DA. Notably, the MC540 assay is not selective towards certain aggregation and can be used for CAC determination of various aggregates, including vesicles and micelles^89,90^. As demonstrated in Fig. 5 and Fig. S204, the CAC of DA at 50 mM phosphate buffer at pH 6.8 was found to be ca. 10 mM, which is in agreement with previous studies^7^. The impact of the DA-LA reaction products on DA’s CAC was dramatic. As the DA:LA molar ratio in the drying reaction increased in favor of LA, the CAC of resulting products decreased, reaching concentrations of about 4.7 mM, 2.0 mM, and 1.0 mM at 1:1, 1:2, and 1:4 molar ratios of DA:LA, respectively (Fig. 5 and Figs. S204–S211). This corresponds to 2-, 5-, and 10-fold reduction, respectively. We attribute this effect to the presence of the new DA-LA conjugates that promote aggregation, likely by increasing the overall hydrophobic driving force for association in the system.

To test whether the observed effect on CAC can be attributed to reaction products of DA-LA or to other molecules in the mixtures, i.e., LA monomers or LA oligomers, we performed control experiments. To that end, we measured the CAC of DA at pH 6.8 in the presence of either LA monomers or oligomers. When LA monomers were introduced at concentrations of 100 mM and 200 mM, the CAC only slightly decreased from 10 mM to about 9.8 mM and 9.3 mM, respectively (Fig. 5 and Figs. S208–S209). The presence of LA oligomers, by contrast, had no significant effect on DA’s CAC (Fig. 5 and Figs. S210–S211). Several factors have been found to promote DA aggregation, including salinity enhancement and additions of lipidic components or other amphiphiles^35,91^. The effect of salts, for instance, is the result of the significant increase in water polarity, essentially turning the amphipathic molecules more hydrophobic^92^. This effect was also observed for LA monomers, albeit to a much lesser extent. The effect of DA-LA conjugates was mechanistically related to the hydrophobic interactions, as was also demonstrated in the case of n-decanol, glycerol mono decanoate, and decylamine, for which their presence led to a reduction of the CVC of DA by up to 10-fold^35,36^. Notably, for the reaction products of DA:LA at a 1:4 molar ratio and pH 6.8, both vesicles and micelles were observed. Therefore, the determined CAC might be attributed to either the micelles or the vesicles. Nonetheless, the concentration is significantly lower than that of DA control samples prepared in the presence and absence of LA monomers or oligomers, highlighting the superior assembly propensity of DA-LA conjugates.

### Permeability and encapsulation by lipid-conjugated oligoesters

**Fig. 6:**
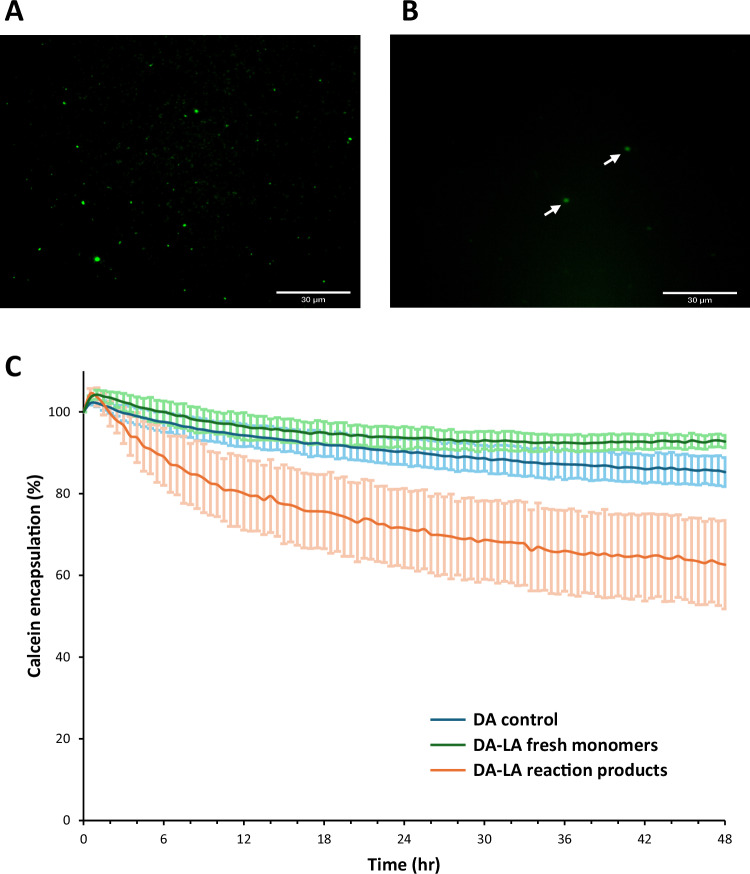
DA-LA reaction products exhibit encapsulation capabilities and greater permeability compared to DA control and DA-LA fresh monomers. Encapsulation of the hydrophilic dye calcein (**A**) and 56-FAM-ssDNA (**B**) within DA-LA reaction products. Calcein leakage expressed as encapsulation percent as a function of time obtained for DA control samples (blue line), DA:LA fresh monomers at a 1:4 molar ratio (orange line), and DA:LA reaction products at a 1:4 molar ratio (**C**). Error bars present standard deviation. Calcein encapsulation percent represents vesicle permeability. As indicated in the figure, calcein leakage from DA-LA reaction products was greater compared to both DA control and the DA-LA fresh monomers, suggesting that vesicles formed in the reaction products are more permeable compared to the unreacted monomers. Source data are provided as a Source Data file.

Cellular permeability is a critical prerequisite in order to promote Darwinian evolution. While passive diffusion through the cell membrane is limited to certain molecules, most of the biologically relevant building blocks require active transportation. Under prebiotic conditions, encapsulation is feasible and almost exclusively relies on transitions in the physical state of the lipids^93,94^. We studied the encapsulation capabilities of our DA-LA lipid-conjugated oligoester products compared to the corresponding controls, and further evaluated the permeability using calcein leakage assay^95^. Encapsulation of calcein (5 mM prior to isolation of encapsulated fractions), a hydrophilic fluorescent dye, was carried out for DA control and DA:LA fresh monomers and reaction products at a 1:4 molar ratio. We confirmed calcein encapsulation using both fluorescence intensity measurements and fluorescence microscopy (Figs. S212A–F). Indeed, we observed encapsulation in all tested systems. Next, we carried out a leakage assay using Triton X-100, a non-ionic surfactant that acts as a piercing agent and increases vesicle permeability^96,97^. To that end, we measured the fluorescence of the samples in the absence or presence of Triton X-100 and determined the encapsulation percent of calcein over a period of 48 h. Higher permeability is correlated with a smaller encapsulation percent^98^. Because we do not know the exact concentration of calcein inside the isolated vesicle fractions, the calcein assay should be interpreted comparatively and not as a direct quantitative measure of encapsulation efficiency. As demonstrated by Fig. 6 and Fig. S212G–I, the leakage of calcein from DA-LA reaction products was greater compared to DA control and DA-LA fresh monomers. This is probably due to the differences in vesicle characteristics, including size, curvature, membrane packing, and membrane thickness^99,100^. The vesicles observed in DA-LA reaction products were considerably smaller compared to the control samples (Figs. S200–S203). As vesicle size decreases, its curvature increases and as a result, the permeability through the membrane increases. Another possible explanation for the observed differences is the presence of micelles, in addition to vesicles, in the calcein encapsulated vesicles of the reaction products, which are not present in DA control and DA:LA fresh monomers. Notably, the increased permeability of the reaction products may represent a tradeoff, potentially facilitating exchange with the environment while at the same time compromising cargo retention and compartment integrity. We also studied the encapsulation of biologically relevant molecules, specifically 56-FAM-ssDNA (Fig. 6B) and poly-(L)-Lysine, within DA:LA reaction products at a 1:4 molar ratio (Fig. S213).

### Differential hydrolysis of DA-LA reaction products

**Fig. 7:**
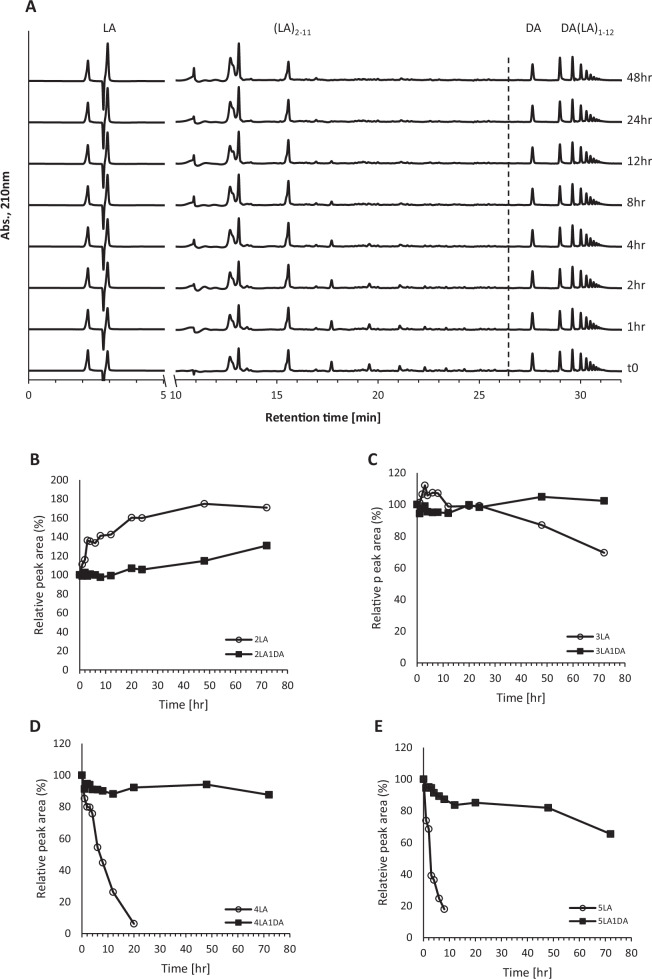
DA-LA conjugated oligoesters exhibit greater persistence than LA oligoesters. Chromatograms obtained for the DA:LA reaction product at a 1:4 molar ratio. The reaction product was rehydrated in a phosphate buffer (50 mM) at pH 6.8 and stored at 40 °C for a period of 72 h, during which samples were withdrawn and analyzed via HPLC **A**. Changes in the peak area of LA dimers that were not covalently bound to DA (empty circles) and LA dimers covalently bound to DA (filled squares) are shown (**B**). Changes in the peak area of LA trimers that were not covalently bound to DA (empty circles) and LA trimers covalently bound to DA (filled squares) are shown (**C**). Changes in the peak area of LA tetramers that were not covalently bound to DA (empty circles) and LA tetramers covalently bound to DA (filled squares) are shown (**D**). Changes in the peak area of LA pentamers that were not covalently bound to DA (empty circles) and LA pentamers covalently bound to DA (filled squares) are shown (**E**). LA dimers accumulated over the course of the reaction regardless of the conjugation to DA. By contrast, longer LA oligomers were more persistent when covalently bound to DA. For longer oligomers the protective effect of the conjugation is more pronounced, as indicated in panels D-E. Source data are provided as a Source Data file.

In the context of origins of life, except for self-assembly and structural properties of prebiotic molecules, which are highly important for compartmentalization, the hydrolysis of such molecules is also crucial. The survival of certain molecules is closely related to their capability to avoid hydrolysis in aqueous environments^64^. As indicated by LC-MS measurements, the products of the reaction between DA and LA comprised two different types of oligomers: LA hydroxy acid oligoesters and DA-LA conjugated oligoesters, both of which contain hydrolysable ester bonds. Therefore, we sought to study the degradation of the esterified products, which can occur both via regular hydrolysis or through a back-biting mechanism^101^. To that end, we focused on the reaction product of DA:LA at a 1:4 molar ratio and studied the degradation rates of LA oligoesters and DA-LA conjugated oligoesters at pH 6.8 and 40 °C for a period of 72 h. The most prominent observation was the differential degradation rates of LA oligoesters compared to DA-LA conjugated oligoesters. At this timescale, LA oligoesters were gradually hydrolyzed within this period, and, after 8 h, only trace amounts of LA pentamer and tetramer were observed. The shortest oligomers, LA dimer and LA trimer, were detected even after 72 h. As opposed to the hydroxy acid oligoesters, DA-LA conjugated oligoesters of up to LA_5_DA were detected even after 72 h (Fig. 7 and Figs. S214–S215). It is worth noting that over the course of the hydrolysis experiment, the pH levels remained approximately constant. The hydrolysis rate of the products was further determined in terms of product degradation by monitoring the changes in the peak area of each product in HPLC chromatograms. Indeed, LA monomers, dimers, and trimers accumulated over the initial incubation period at the expense of the longer LA oligomers and heterooligomers (Fig. 7A). At the end of the experiment, LA monomers had doubled in terms of peak area and LA dimers had increased by ~1.5-fold compared to the beginning of the reaction (Fig. 7A, B and Fig. S216). In the case of DA-LA conjugated oligoesters, LA_1_DA and LA_2_DA accumulated 1.3-fold over the incubation period at the expense of the longer DA-LA oligomers (Fig. 7A, B and Fig. S216). Yet the levels of DA were slightly decreased, probably due to minor volatilization of DA over the course of the reaction (Fig. S216).

From the perspective of LA oligomers, when comparing the persistence of those that were not conjugated to DA to those that were conjugated to DA, one can clearly see the enhanced chemical stability of the oligomers that were conjugated to DA over the ones that were not. For instance, 30% of LA trimers that were not conjugated to DA were hydrolyzed after 72 h at 40 °C, while the corresponding ones that were conjugated to DA were practically not hydrolyzed over the course of the experiment (Fig. 7C). In the case of the longer oligomers, the resistance to hydrolysis was even more pronounced when the oligomers were conjugated to DA (Fig. 7D, E): a reduction of 82% after 8 h at 40 °C in the peak area of LA pentamers that were not conjugated to DA was observed, compared to only 13% for the DA-conjugated ones. The non-conjugated LA pentamers were completely hydrolyzed after 72 h, while the DA-conjugated pentamers were only partially hydrolyzed, with a reduction of 35% (Fig. 7E). The faster hydrolysis rate of hydroxy acid oligoesters compared to lipid-conjugated oligoesters was also observed across various pH levels (5.5, 6.8, or 8.0) and temperatures (room temperature (RT), 40 °C, or 60 °C) (Figs. S217–S228). As expected, the hydrolysis rate of both types of products was temperature- and pH-dependent.

The immense protection against hydrolysis can be attributed to structure formation. Upon structure formation, the accessibility of the ester groups to hydrolysis via water molecules is significantly hindered. Medium-chain fatty acids and their ester derivatives are known to form various structures^102^. A study by Bonfio *et al*. achieved accumulation of medium-chain acylglycerol-phosphates over short-chain acylglycerol-phosphate through iterative acylation-hydrolysis cycles^16^. The accumulation was accompanied by vesicle formation. The authors ascribed this effect to the selective hydrolysis of the shorter-chain acylglycerol-phosphate due to their lack of self-assembly capability. Regardless of the particular nature of the amphiphile or the specific nature of the assembly, the hydrolysis rate is highly dependent on the concentration of the amphiphiles and on the presence of structures. Typically, hydrolysis rates are hindered upon assembly, even though the reverse phenomenon has also been observed^103–105^. In addition, studies have demonstrated that sucrose esters tend to hydrolyze to a greater extent under acidic conditions below their critical micelle concentration (CMC)^106^. Ethoxylated fatty esters hydrolysis under basic conditions was also hindered due to the presence of micelles compared to the non-aggregated surfactants^107^. This hydrolysis inhibition manifested in different kinetics of the hydrolysis; above the CMC of the tested ethoxylated fatty esters, hydrolysis profiles fit pseudo zero-order reaction, while below the CMC, first-order kinetics was observed. The protective effect of self-assembly is observed in that study even under basic conditions, which is remarkable given that ester bonds hydrolyze much faster under basic conditions^108^. The authors primarily attributed this to the fact that the ester bond is not accessible to the hydroxide ions as a result of micelle formation. Other physicochemical characteristics, such as polarity of the structure’s surface and its charge, significantly affect the hydrolysis rate as well.

The selective hydrolysis of LA oligoesters as opposed to the hydrolysis of DA-LA conjugated oligoesters suggests that the LA oligoesters formed in the dry reaction in the presence of DA are not capable of self-assembly under tested conditions. Indeed, these esters were found to be shorter than those obtained in the absence of DA, which can affect their aggregation properties. It is worth noting that even if LA oligoesters do not form structures by themselves under the tested conditions, co-aggregation with DA and its products is theoretically possible, but then the hydrolysis rate of LA oligoesters is expected to be reduced. For instance, the hydrolysis of ethyl benzoate and several aliphatic esters was significantly inhibited in the presence of anionic, cationic, and nonionic surfactants above their CMC in a concentration-dependent manner^109^. The authors ascribed this effect to the presence of ester-micelle complexes. The partitioning of the esters in the bulk and in the micellar phases resulted in overall inhibition of hydrolysis rates. However, based on our results, such co-aggregation is very unlikely due to the rapid hydrolysis of the hydroxy acid oligoesters.

We further examined the hydrolysis of LA oligoesters obtained by the dry reaction of LA at 85 °C in the absence of DA. As mentioned, in the absence of DA, LA oligomerized to a greater extent, forming longer hydroxy acid oligoesters. These products form aggregates at pH 6.8, as indicated by microscopy (Fig. S197). Upon incubation at RT and pH 6.8 for up to 4 days, LA oligoesters of medium chain length hydrolyzed while the longest ones were still detected and exhibited lower degradation rates (Figs. S229–S232). The protective effect of self-assembly has been reported previously in the context of chemical evolution^64^. It has been demonstrated that chemical entities capable of self-assembly in aqueous environments are recalcitrant (i.e., protected by assembly) and therefore undergo hydrolysis to a lesser extent due to kinetic trapping associated with the assembled state. That is, molecules capable of self-assembly possess an evolutionary advantage over other molecules lacking self-assembly. Our result underscores the crucial protective role of self-assembly against degradation in water, which is a universal phenomenon not restricted to any particular assembly mode^64^.

### Green chemical approach for the synthesis of amphiphile mixtures

Amphiphiles based on HA derivatives, especially the ones obtained by the condensation of fatty acids and HAs, have been known for years and used for several applications in the food and pharmaceutical industries^110,111^. The current synthesis strategies of such amphiphiles involve either one-step or two-step reactions^111,112^. In one-step reactions, lactic acid, fatty acid, and sodium carbonate are reacted together, while the two-step reactions involve consecutive polymerization and esterification. These surfactants are considered environmentally friendly and biodegradable. The current derivatives of HA amphiphiles are mostly fatty acyl lactylates, which are mixtures of molecules with various lactic acid polymerization degrees^83,113^. Our simple prebiotic, green synthesis was found to be highly reproducible and resulted in a mixture of both hydroxy acid oligoesters and DA-HA conjugated oligoesters that exhibit unique properties and can be used for multiple applications. The differential hydrolytic rates of the hydroxy acid oligoesters and DA-HA conjugated oligoesters can be used for the selective release of drugs based on their affinity to either type of oligomer.

### The universality of cooperation between additional hydroxy acids and fatty acids

The process of chemical evolution has led to the emergence of the four fundamental, well-organized components of life: proteins, carbohydrates, nucleic acids, and lipids. It is plausible that throughout this evolutionary process, the individual pathways leading to the formation of these major biomolecular classes have occasionally converged, giving rise to symbiotic coevolution. In such cases, mutual interactions between different evolving molecular species may have contributed to their evolutionary trajectory. An example of this is the interaction of DA and LA, which exhibited characteristics of symbiotic coevolution where both molecular species benefited; LA oligomers gained greater hydrolytic stability when conjugated to DA, and DA molecules formed vesicles more readily and at lower concentrations when linked to LA oligomers.

To assess whether this mutualistic effect is generalizable, we tested additional lipid-hydroxy acid systems. Specifically, we performed hydrolysis assays and determination of the CAC for mixtures of either octanoic acid and dodecanoic acid with LA, DA with PLA, and a ternary mixture of DA with both LA and GA (Supplementary Methods and Figs. S233–S248). Indeed, the hydrolysis assays confirmed the positive effect of fatty acid conjugation: all tested oligomers showed enhanced hydrolytic stability when covalently linked to fatty acids (Figs. S233–S241). In turn, the conjugated HA oligomers facilitated the aggregation of the lipids as indicated by the merocyanine assay. For instance, the CAC of octanoic acid, decanoic acid, and dodecanoic acid decreased by approximately tenfold upon conjugation with LA oligomers (Figs. S242–S248). These results demonstrate that the mutualistic effect between DA and LA is generalizable to various lipid-hydroxy acid systems and further support the plausibility of symbiotic coevolution of different molecular species.

In the past, decanoic acid (DA) and hydroxy acids (HAs) have been investigated separately in the context of origins of life as a protocell model amphiphile and as a proto-polymer model, respectively. In this work we studied the coevolution of these two different building blocks, focusing on the interplay between polymerization, hydrolysis, and compartmentalization. Through simple drying reactions, we were able to generate amphiphiles derived from DA-HA ester conjugates that readily self-assemble in aqueous solutions. We focused our investigation on drying reactions between DA and lactic acid (LA), which produced a mixture of both hydroxy acid oligoesters and lipid-conjugated oligoesters. The structural characteristics of DA assemblies were positively affected by the presence of DA-LA conjugated oligoesters, lowering the critical assembly concentration of the amphiphile and facilitating the formation of both vesicles and micelles. In a mutualistic manner, the LA oligomers that were conjugated to DA exhibited slower degradation rates than the corresponding LA oligoesters. In our work, experiments with additional lipid-hydroxy acid systems have shown that the mutualistic effect between DA and LA is generalizable. In this sense, the conjugates do not simply represent new products, but a molecular system in which assembly propensity and oligomer stability become linked.

The functional implications of coupling between lipid moieties and oligoesters remain prospective and may go beyond the mere persistence/stability level. First, HAs could have played a significant role as prebiotic catalysts^114^, for example in proto-peptide synthesis^65^, and perhaps the existence of oligoesters on lipid surfaces could have catalyzed reactions such as lipid-mediated peptide bond formation^85^. Hence, it is possible that hydrolytic stability can be harnessed to control and catalyze various chemical reactions on lipid surfaces, possibly even in an aqueous environment. Second, HAs and the conjugated lipids described here are individually capable of self-assembly and therefore could support hierarchical compartmentalization where HA droplets could be encapsulated within DA-HA conjugates. Such hierarchical compartmentalization could further be used to support a network of different concomitant processes, each taking place in a different microenvironment. Third, by changing both assembly behavior and permeability-related properties, such conjugates may shift the balance between uptake and retention in primitive compartments. Our results support the idea that oligomerization and compartmentalization could have become coupled during chemical evolution, such that interactions between distinct molecular subsystems generated emergent properties not present in either subsystem alone.

## Methods

### Single-step dry reactions

Binary mixtures of decanoic acid (DA) and hydroxy acids (HAs) at 1:1, 1:2, and 1:4 molar ratios were prepared in 7 mL scintillation vials. All reagents were used as received under neat (solvent-free) conditions. DA’s amount was fixed at 200 μmol and HAs’ amount was adjusted accordingly (either 200, 400, or 800 μmol). All components were weighed except for lactic acid, which was added volumetrically. The mixtures were placed at 85 °C for seven days. Samples of either DA (200 µmol) alone or hydroxy acids (400 µmol) alone were prepared as control. An aliquot of 1 M HCl (1 µmol) was added to the DA control. All samples were prepared in triplicate. A similar procedure was carried out for the other mixtures of fatty acids (FAs) and HAs.

### Samples rehydration and structure reconstitution

For structural characterization, crude reaction products were rehydrated in water, concentrated phosphate monobasic solution (1.0 M), and NaOH solution (1.0 M). Samples were vigorously mixed, and their pH was adjusted to 6.8 using NaOH. The final buffer concentration was 50 mM. DA and HAs concentration was 50 mM and 50–200 mM, respectively, referring to initial amounts prior to the reaction. Control samples of fresh monomers were prepared as well. For DA control sample, DA stock (180 mM DA and 200 mM NaOH) was mixed with water and phosphate monobasic solution (1.0 M), following pH adjustment to 6.8 using HCl solution (1.0 M). The subsequent concentration of NaCl obtained by HCl titration was not greater than 10 mM, which has a negligible effect on structural properties. For DA-LA fresh controls, DA stock (180 mM DA and 200 mM NaOH) was mixed with water and NaOH solution (1.0 M). Then, LA stock (1.0 M) and phosphate monobasic (1.0 M) were added, and pH was adjusted to 6.8 using NaOH solution. In all control samples, the final buffer and DA concentrations were 50 mM. LA final concentration in DA-LA fresh monomers was either 50, 100 or 200 mM, corresponding to DA:LA at a 1:1, 1:2, or 1:4 molar ratio, respectively. All samples (reaction products and controls) were kept stationary at room temperature for 16–24 h prior to characterization. All samples were prepared in triplicates.

### High-performance liquid chromatography and LC-MS

HPLC analyses were conducted using an Agilent 1260 quaternary pump and autosampler (Agilent Technologies, Santa Clara, CA, USA) with a DAD UV–vis detector at 210 nm and 259 nm. LC-MS data were collected using an Agilent G6135C single quadrupole mass spectrometer with a capillary voltage of 4.0 kV and a source fragmentation voltage of 70 V. Scan range: 50–1500 m/z. Chromatographic separation was achieved using an InfinityLab Poroshell 120 EC-C18 column (150 × 3.0 mm, 2.7 µm, with a SecurityGuard^TM^ C18 4 × 2.0 mm), at a constant 0.3 mL/min flow rate. Column cell temperature was maintained at 20 °C. Gradient elution was carried out using (A) 0.1% formic acid in water and (B) acetonitrile as follows: 5 min 100% A, 20 min ramp to 20% A, 10 min 100% B. Further details on chromatographic methods are given in the SI.

### Preparation of fatty acid (FA) standard solutions for HPLC analyses

Stock solutions of FA were prepared by dissolving FAs in acetonitrile (ACN) in volumetric flasks. Calibration standards of concentration ranging between 1 mM and 8 mM were prepared by diluting the FA stock solution in ACN:water 50:50.

### Preparation of samples for HPLC analyses

The samples obtained from the dry reactions were dissolved in ACN and sonicated to allow the complete extraction of the analytes. All samples were completely solubilized in ACN except for GA samples, which were only partially solubilized. Samples were further diluted in ACN:water 50:50 to reach a final concentration of 5 mM FA, referring to initial amounts prior to the reaction. The consumption of DA was calculated using the calibration curve.

### ATR-FTIR measurements

Attenuated total reflectance Fourier transform infrared measurements were carried out on a Jasco (Tokyo, Japan) FT/IR-4×1 Spectrometer. Prior to measurement, 50 µL of 100 mM samples solubilized in acetonitrile were placed on aluminum foil and allowed to dry under ambient conditions. The aluminum foil was then placed in an Attenuated Total Reflectance (ATR) sample chamber for analysis of the dried sample. Spectra were signal-averaged (60 scans per spectrum) and background-subtracted and ranged from 400 to 4000 cm^−1^. Spectra were analyzed using Excel software.

### NMR spectroscopy

^1^H-NMR spectra were recorded on a 500 MHz Neo spectrometer. Reaction products or fresh monomers were dispersed in chloroform-D_3_ and sonicated for 1 min in an ice bath. In the case of glycolic acid and malic acid, fresh monomers and the HA controls were also dispersed in D_2_O. All data were plotted and processed using the TopSpin software.

### Microscopy imaging

100 µL of reconstituted samples were mixed with rhodamine 6 G solution (which was prepared in ethanol). Final dye concentration was 1 mM. Images were conducted utilizing an ECHO Revolve microscope (ECHO, San Diego CA), equipped with an 8MP CMOS Color camera, with a magnification of X60 Olympus^®^ air objective. FITC filter (EX: 470/40, EM: 525/50, DM: 495) was used for both fluorescent and bright field measurements.

### Cryogenic-transmission electron microscopy (Cryo-TEM)

FEI Tecnai 12 G2 TWIN TEM Operated at 120 kV and equipped with a Gatan Model 626 Cold Stage was used. The Images were recorded by a 4 k × 4 k FEI Eagle CCD Camera in Low Dose Mode. TIA (Tecnai Imaging and Analysis) software was used to record the images. Sample preparation was performed by applying 3 µl samples onto a glow-discharged TEM grid (300-mesh Cu, Lacey substrate, Ted Pella, Ltd., 4595 Mountain Lakes Blvd., Redding, CA, USA). The excess liquid was blotted for 2 s, and the specimens were vitrified by a rapid plunging into liquid ethane precooled with liquid nitrogen using Vitrobot Mark IV (FEI). For statistical assessment of vesicle size, vesicle diameter was measured using the TIA software. For that purpose, only spherical or almost spherical vesicles were included. Micelles were excluded from the analysis.

### Turbidity measurements

Turbidity measurements were carried out on a Synergy H1 plate reader (BioTek Instruments, VT, USA). 200 µL samples were placed in 96-wells plate in technical duplicates. Spectra were recorded between 400 nm and 800 nm. Turbidity was reported as the absorbed light at 400 nm.

### Dynamic light scattering (DLS) measurements

Mean particle size, polydispersity index, and size distribution were determined based on dynamic light scattering measurements. All measurements were carried out using a Nano-ZS instrument (Malvern Panalytical, Malvern, UK) equipped with a laser at a wavelength of 633 nm and a back scattering detector (173°). All measurements were carried out at 25 °C following 120 s equilibration step. Five repeat measurements were carried out for each sample. Instrument settings were automatically optimized. For DLS measurements, the samples were rehydrated as described above to reach DA concentration of 50 mM. The samples were diluted twice with 50 mM phosphate buffer at pH 6.8 to reach DA concentration of 25 mM. All DLS experiments were carried out in triplicates. Data was analyzed using Zetasizer software. Details on data analysis are provided in the Supplementary methods.

### Critical aggregation concentration (CAC) determination

The critical aggregation concentration of DA and other fatty acids (FAs) in fresh controls and reaction products was determined using the Merocyanine 540 assay. To that end, dilution lines were prepared by diluting stocks of fresh monomers or reaction products (see sample rehydration) with phosphate buffer and pH was adjusted as required. The dilution lines were constructed to cover a range of FA concentration that are below and above the CAC. The resulting dilutions were mixed with Merocyanine 540 stock of 1 mg/mL, resulting in a final Merocyanine 540 concentration of 20 μg/mL. 150 µL samples were placed in 96-wells black plate. Spectra were recorded on a Synergy H1 plate reader (BioTek Instruments, VT, USA) between 400 nm and 620 nm. The ratio between the absorption at 570 nm and 530 nm was calculated and plotted against FA concentration. The point at which the ratio increases significantly was considered as the CAC. The determined values were extracted from the intersection between the linear fitting lines in the first two regions of the curves. All experiments were carried out in duplicates.

### Calcein encapsulation and leakage

Encapsulation experiments were carried out using calcein (a hydrophilic dye). Samples were prepared as described in the above-mentioned sections. The only exception was that calcein (80 mM solution, 167 mM NaOH) was added to the DA prior to the addition of either LA or phosphate monobasic solutions in order to introduce it to the lipid phase prior to the formation of vesicles. Calcein final concentration was 5 mM. Samples were left at RT overnight prior to isolation of the encapsulated calcein. The isolation of calcein-encapsulated vesicles was achieved by size exclusion chromatography (SEC) over Sepharose^TM^ 4B beads. Sepharose columns were prepared in 5 mL disposable syringe into which 5 mL Sepharose beads were added. The mobile phase used for the separation was a vesicular system of 30 mM DA in 50 mM phosphate buffer at pH 6.8. The column was first washed with 50 mM phosphate buffer at pH 6.8 following by equilibration with the mobile phase (about 3 column volumes). Then, 0.5 mL samples were loaded into the column and 10 fractions of 0.5 mL were collected. To confirm the encapsulation, the collected fractions were examined under a fluorescence microscope (FITC filter, EX: 470/40, EM: 525/50, DM: 495). In addition, fluorescence measurements were carried out using a Synergy H1 plated reader at Ex: 490/10, Em 520/10. Calcein-encapsulated fractions were further used for the leakage assay

Leakage assay was carried out as previously described^95^, with minor modifications. Briefly, the third, fourth and fifth fractions (i.e., the calcein-encapsulated fractions) obtained from SEC separation were mixed together. Then, the mixture was divided into two. Triton X-100 was added to one of the aliquots at a final concentration of 0.3% (wt/vol). In addition, control samples of 0.025 mM calcein in either 50 mM phosphate buffer or mobile phase were prepared similarly with or without Triton X-100. Additional control was prepared using the eighth, ninth, and tenth fractions obtained from the SEC, which are the free calcein fractions. All samples were placed in 96-wells black plate covered with sealing tape, and the fluorescence was measured (Ex: 490/10, Em 520/10) over a period of 48 h in 5 min intervals. The encapsulation percentage of calcein was then calculated according to the formula:

$$\%\,{encapsulation}=\left(1-\frac{{F}_{t}-{F}_{0}}{{F}_{{Tx},t}-{F}_{0}}\right)\times100\%$$

Where *F*_t_ is the measured fluorescence of the samples without Triton X-100 at time *t*, *F*_0_ is the measured fluorescence of the samples without Triton X-100 at *t*_0_, and *F*_Tx,t_ is the measured fluorescence of the samples with 0.3% Triton X-100 (wt/vol) at time *t*.

### ssDNA encapsulation

The encapsulation of ss-DNA (5’- | 56-FAM/CGCTAAATCG-3’, Mw 3549.5 g/mol) was carried out using the reaction product of DA:LA at a 1:4 molar ratio. For sample preparation, the dry reaction product was dissolved in acetonitrile to a final concentration of 100 mM. Then, aliquots of 100 μL (corresponding to 10 μmol DA and 40 μmol LA) were withdrawn and acetonitrile was removed under vacuum at 30 °C. Then labeled ss-DNA (100 μM solution) was added to the dry DA-LA mixture followed by the addition of phosphate monobasic and sodium hydroxide. The pH was adjusted with sodium hydroxide (1.0 M and 100 mM solutions) to pH 6.8 using an InLab Micro pH electrode (Mettler Toledo). Following preparation, the DNA-encapsulated fraction was isolated using Sepharose^TM^ 4B SEC and the encapsulated fraction was inspected under a fluorescence microscope (FITC filter, EX: 470/40, EM: 525/50, DM: 495).

### Hydrolysis and degradation experiments

The hydrolysis and degradation profile of the dried products obtained in the drying reactions was evaluated for various reaction products. Crude reaction products were rehydrated in water and with either 1.0 M solutions of tris, phosphate monobasic, or citric acid, and the pH of the samples was further adjusted to 8.0, 6.8, or 5.5, respectively. The resulting rehydrated samples were dispensed into aliquots in 1.5 mL microtubes. Each sample was withdrawn at different time points. Samples were stored at various temperatures (RT, 40 °C, or 60 °C) and withdrawn at different time points. All withdrawn samples were stored at −80 °C until being analyzed by LC-UV/MS. Prior to HPLC analyses, the pH of each sample was roughly measured using a pH Indicator Strip, universal Specification (0–14.0) (Millipore, Merck). For HPLC analyses, samples were diluted four to ten times fold. To confirm that no degradation occurred during the analysis itself, representative samples were injected several times during the course of the analysis.

Control samples of LA reaction products using the initial amount of either 400 µmol or 800 µmol were tested for hydrolysis as well. The dry reaction products were rehydrated in water and phosphate buffer and the pH was adjusted to 6.8. The final concentration of LA was either 100 mM or 200 mM, referring to initial amounts prior to the reaction. The rehydrated products were stored at RT and samples of 800 µL were withdrawn immediately after preparation and after 12 h and 4 days. The withdrawn samples were treated as described above.

To monitor the degradation rate of the products, the area of each product was integrated by OpenLab software using Chemstation integrator. Every peak area obtained at each time point was normalized to t0 and the relative change was plotted over time.

## Supplementary information

Supplementary Information

Transparent Peer Review file

## Source data

Source data for Figure 2

Source data for Figure 3

Source data for Figure 4

Source data for Figure 5

Source data for Figure 6

Source data for Figure 7

Source data for Figure S2

Source data for Figure S3

Source data for Figure S4

Source data for Figure S5

Source data for Figure S6

Source data for Figure S7

Source data for Figure S8

Source data for Figure S9

Source data for Figure S10

Source data for Figure S11

Source data for Figure S12

Source data for Figure S13

Source data for Figure S14

Source data for Figure S15

Source data for Figure S16

Source data for Figure S17

Source data for Figure S22

Source data for Figure S23

Source data for Figure S163

Source data for Figure S164

Source data for Figure S165

Source data for Figure S166

Source data for Figure S214-S215

Source data for Figure S217

Source data for Figure S218

Source data for Figure S219

Source data for Figure S2220

Source data for Figure S221

Source data for Figure S222

Source data for Figure S223

Source data for Figure S224

Source data for Figure S225

## Data Availability

All the data supporting the findings of this study are available within the main text and its Supplementary Information and from the corresponding author upon request. Source data are provided with this paper.
