# Supplementary material for: Hydroxy acid conjugation to lipids increases structural and hydrolytic stability
Source: Nat Commun. 2026 Jul 27;17:6973. doi: 10.1038/s41467-026-75192-5 (PMC13408983; doi:10.1038/s41467-026-75192-5)
Supplement: Supplementary file 1 — Supplementary Information [file 41467_2026_75192_MOESM1_ESM.pdf]

Supplementary Information

Lipid-Hydroxy Acids Conjugates as Hydrolytically stable Prebiotic Amphiphiles

R. Edri[1], S. Fisher[1], Y. Levi-Kalisman[2], M. Frenkel-Pinter[1,2,3]*

[1] Institute of Chemistry, The Hebrew University of Jerusalem, Israel 9190401

[2] The Center for Nanoscience and Nanotechnology, The Hebrew University of Jerusalem, Israel 9190401

[3] Casali Center of Applied Chemistry, The Hebrew University of Jerusalem, Israel 9190401

Contents

Materials 19

Methods 19

Single-step dry reactions. 19

Poly-Lys encapsulation. 20

High-Performance Liquid Chromatography / LC-MS. 20

Critical aggregation concentration (CAC) determination. 21

Hydrolysis and degradation experiments. 22

Dynamic light scattering (DLS) measurements. 24

Figure S1. Visual appearance of the binary mixtures of DA and the tested HAs at 1:1, 1:2, and 1:4 molar ratios and HAs control samples. 26

Figure S2. LC-MS analysis confirms the formation of products obtained by the reaction of DA and GA at a 1:1 molar ratio. 27

Figure S3. LC-MS analysis confirms the formation of products obtained by the reaction of DA and GA at a 1:2 molar ratio. 28

Figure S4. LC-MS analysis confirms the formation of products obtained by the reaction of DA and GA at a 1:4 molar ratio. 29

Figure S5. LC-MS analysis confirms the formation of products obtained by the reaction of DA and LA at a 1:1 molar ratio. 30

Figure S6. LC-MS analysis confirms the formation of products obtained by the reaction of DA and LA at a 1:2 molar ratio. 31

Figure S7. LC-MS analysis confirms the formation of products obtained by the reaction of DA and LA at a 1:4 molar ratio. 32

Figure S8. LC-MS analysis confirms the formation of products obtained by the reaction of DA and MA at a 1:1 molar ratio. 33

Figure S9. LC-MS analysis confirms the formation of products obtained by the reaction of DA and MA at a 1:2 molar ratio. 34

Figure S10. LC-MS analysis confirms the formation of products obtained by the reaction of DA and MA at a 1:4 molar ratio. 35

Figure S11. LC-MS analysis confirms the formation of products obtained by the reaction of DA and PLA at a 1:1 molar ratio. 36

Figure S12. LC-MS analysis confirms the formation of products obtained by the reaction of DA and PLA at a 1:2 molar ratio. 37

Figure S13: LC analysis confirms the formation of products obtained by the reaction of DA and PLA at a 1:4 molar ratio. 38

Figure S14. LC-MS analysis confirms the formation of products obtained for GA control in the absence of DA. 39

Figure S15. LC-MS analysis confirms the formation of products obtained for LA control in the absence of DA. 40

Figure S16. LC-MS analysis confirms the formation of products obtained for MA control in the absence of DA. 41

Figure S17. LC-MS analysis confirms the formation of products obtained for PLA control in the absence of DA. 42

Figure S18. DA and GA react to produce two types of products: homoesters of GA and heteroesters of DA and GA. 43

Figure S19. DA and LA react to produce two types of products: homoesters of LA and heteroesters of DA and LA. 44

Figure S20. DA and MA react to produce two types of products: homoesters of MA and heteroesters of DA and MA. 45

Figure S21. DA and PLA react to produce two types of products: homoesters of PLA and heteroesters of DA and PLA.. 46

Figure S22. Batch size affects product formation and the concentration of products obtained by the dry-state reaction of DA and LA. 47

Figure S23. DA conversion obtained for different batch sizes and different molar ratios. 48

Figure S24. MS spectrum extracted from DA-GA reaction product chromatogram at retention time 11.6 min 50

Figure S25. MS spectrum extracted from DA-GA reaction product chromatogram at retention time 12.2 min.. 51

Figure S26. MS spectrum extracted from DA-GA reaction product chromatogram at retention time 13.1 min. 52

Figure S27. MS spectrum extracted from DA-GA reaction product chromatogram at retention time 14.2 min.. 53

Figure S28. MS spectrum extracted from DA-GA reaction product chromatogram at retention time15.2 min. 54

Figure S29. MS spectrum extracted from DA-GA reaction product chromatogram at retention time 16.2 min.. 55

Figure S30. MS spectrum extracted from DA-GA reaction product chromatogram at retention time 17.0 min.. 56

Figure S31. MS spectrum extracted from DA-GA reaction product chromatogram at retention time 17.8 min.. 57

Figure S32. MS spectrum extracted from DA-GA reaction product chromatogram at retention time 18.5 min.. 58

Figure S33. MS spectrum extracted from DA-GA reaction product chromatogram at retention time 19.1 min. 59

Figure S34. MS spectrum extracted from DA-GA reaction product chromatogram at retention time 19.7 min.. 60

Figure S35. MS spectrum extracted from DA-GA reaction product chromatogram at retention time 28.2 min. 61

Figure S36. MS spectrum extracted from DA-GA reaction product chromatogram at retention time 28.4 min. 62

Figure S37. MS spectrum extracted from DA-GA reaction product chromatogram at retention time 28.7 min. 63

Figure S38. MS spectrum extracted from DA-GA reaction product chromatogram at retention time 28.8 min. 64

Figure S39. MS spectrum extracted from DA-GA reaction product chromatogram at retention time 28.9 min. 65

Figure S40. MS spectrum extracted from DA-GA reaction product chromatogram at retention time 29.0 min.. 66

Figure S41. MS spectrum extracted from DA-MA reaction product chromatogram at retention time 7.7 min. 69

Figure S42. MS spectrum extracted from DA-MA reaction product chromatogram at retention time 8.7 min.. 70

Figure S43. MS spectrum extracted from DA-MA reaction product chromatogram at retention time 11.4 min. 71

Figure S44. MS spectrum extracted from DA-MA reaction product chromatogram at retention time 12.1 min. 72

Figure S45. MS spectrum extracted from DA-MA reaction product chromatogram at retention time 12.5 min. 73

Figure S46. MS spectrum extracted from DA-MA reaction product chromatogram at retention time 12.8 min.. 74

Figure S47. MS spectrum extracted from DA-MA reaction product chromatogram at retention time 13.2 min.. 75

Figure S48. MS spectrum extracted from DA-MA reaction product chromatogram at retention time 13.4 min.. 76

Figure S49. MS spectrum extracted from DA-MA reaction product chromatogram at retention 13.6 min. 77

Figure S50. MS spectrum extracted from DA-MA reaction product chromatogram at retention time 13.8 min.. 78

Figure S51. MS spectrum extracted from DA-MA reaction product chromatogram at retention time 14.0 min.. 79

Figure S52. MS spectrum extracted from DA-MA reaction product chromatogram at retention time 14.4 min. 80

Figure S53. MS spectrum extracted from DA-MA reaction product chromatogram at retention time 14.7 min. 81

Figure S54. MS spectrum extracted from DA-MA reaction product chromatogram at retention time 14.9 min. 82

Figure S55. MS spectrum extracted from DA-MA reaction product chromatogram at retention time 15.0 min. 83

Figure S56. MS spectrum extracted from DA-MA reaction product chromatogram at retention time 15.3 min. 84

Figure S57. MS spectrum extracted from DA-MA reaction product chromatogram at retention time 15.6 min. 85

Figure S58. MS spectrum extracted from DA-MA reaction product chromatogram at retention time 23.6 min. 86

Figure S59. MS spectrum extracted from DA-MA reaction product chromatogram at retention time 23.9 min. 87

Figure S60. MS spectrum extracted from DA-MA reaction product chromatogram at retention time 24.3 min. 88

Figure S61. MS spectrum extracted from DA-MA reaction product chromatogram at retention time 24.8 min.. 89

Figure S62. MS spectrum extracted from DA-MA reaction product chromatogram at retention 25.5 min. 90

Table S3. Identification of DA-PLA reaction products. 91

Figure S63. MS spectrum extracted from DA-PLA reaction product chromatogram at retention time 23.1 min. 92

Figure S64. MS spectrum extracted from DA-PLA reaction product chromatogram at retention time 26.6 min. 93

Figure S65. MS spectrum extracted from DA-PLA reaction product chromatogram at retention time 29.0 min. 94

Figure S66. MS spectrum extracted from DA-PLA reaction product chromatogram at retention time 30.0 min.. 95

Figure S67. MS spectrum extracted from DA-PLA reaction product chromatogram at retention time 30.4 min.. 96

Figure S68. MS spectrum extracted from DA-PLA reaction product chromatogram at retention time 30.7 min.. 97

Figure S69. MS spectrum extracted from DA-PLA reaction product chromatogram at retention time 31.4 min. 98

Figure S70. MS spectrum extracted from DA-PLA reaction product chromatogram at retention time 32.2 min. 99

Figure S71. MS spectrum extracted from DA-PLA reaction product chromatogram at retention 33.0 min. 100

Figure S72. MS spectrum extracted from DA-PLA reaction product chromatogram at retention time 33.8 min.. 101

Figure S73. MS spectrum extracted from DA-PLA reaction product chromatogram at retention time 34.8 min. 102

Table S4. Identification of DA-LA reaction products. 103

Figure S74. MS spectrum extracted from DA-LA reaction product chromatogram at retention time 12.5 min. 104

Figure S75. MS spectrum extracted from DA-LA reaction product chromatogram at retention time 13.2 min.. 105

Figure S76. MS spectrum extracted from DA-LA reaction product chromatogram at retention time 15.7 min. 106

Figure S77. MS spectrum extracted from DA-LA reaction product chromatogram at retention time 17.8 min. 107

Figure S78. MS spectrum extracted from DA-LA reaction product chromatogram at retention time 19.5 min. 108

Figure S79. MS spectrum extracted from DA-LA reaction product chromatogram at retention time 19.7 min. 109

Figure S80. MS spectrum extracted from DA-LA reaction product chromatogram at retention time 21.0 min.. 110

Figure S81. MS spectrum extracted from DA-LA reaction product chromatogram at retention time 21.2 min. 111

Figure S82. MS spectrum extracted from DA-LA reaction product chromatogram at retention time 22.2 min. 112

Figure S83. MS spectrum extracted from DA-LA reaction product chromatogram at retention time 22.4 min. 113

Figure S84. MS spectrum extracted from DA-LA reaction product chromatogram at retention time 23.3 min. 114

Figure S85. MS spectrum extracted from DA-LA reaction product chromatogram at retention time 23.5 min. 115

Figure S86. MS spectrum extracted from DA-LA reaction product chromatogram at retention time 24.4 min. 116

Figure S87. MS spectrum extracted from DA-LA reaction product chromatogram at retention time 25.2 min. 117

Figure S88. MS spectrum extracted from DA-LA reaction product chromatogram at retention time 25.9 min.. 118

Figure S89. MS spectrum extracted from DA-LA reaction product chromatogram at retention time 26.6 min.. 119

Figure S90. MS spectrum extracted from DA-LA reaction product chromatogram at retention time 27.2 min. 120

Figure S91. MS spectrum extracted from DA-LA reaction product chromatogram at retention time 29.1 min. 121

Table S5. Identification of DA-LA reaction products. 122

Figure S92. MS spectrum extracted from DA-LA reaction product optimized chromatogram at retention time 10.0 min. 123

Figure S93. MS spectrum extracted from DA-LA reaction product optimized chromatogram at retention time 10.5 min. 124

Figure S94. MS spectrum extracted from DA-LA reaction product optimized chromatogram at retention time 11.0 min. 125

Figure S95. MS spectrum extracted from DA-LA reaction product optimized chromatogram at retention time 11.4 min. 126

Figure S96. MS spectrum extracted from DA-LA reaction product optimized chromatogram at retention time 11.8 min. 127

Figure S97. MS spectrum extracted from DA-LA reaction product optimized chromatogram at retention time 12.2 min. 128

Figure S98. MS spectrum extracted from DA-LA reaction product optimized chromatogram at retention time 12.5 min. 129

Figure S99. MS spectrum extracted from DA-LA reaction product optimized chromatogram at retention time 12.8 min. 130

Figure S100. MS spectrum extracted from DA-LA reaction product optimized chromatogram at retention time 13.1 min.. 131

Figure S101. MS spectrum extracted from DA-LA reaction product optimized chromatogram at retention time 13.4 min. 132

Figure S102. MS spectrum extracted from DA-LA reaction product optimized chromatogram at retention time 13.7 min. 133

Figure S103. MS spectrum extracted from DA-LA reaction product optimized chromatogram at retention time 13.9 min. 134

Table S6. Identification of LA reaction products (in the absence of DA). 135

Figure S104. MS spectrum extracted from LA control chromatogram at retention time 12.5 min. 136

Figure S105. MS spectrum extracted from LA control chromatogram at retention time 13.2 min. 137

Figure S106. MS spectrum extracted from LA control chromatogram at retention time 15.6 min. 138

Figure S107. MS spectrum extracted from LA control chromatogram at retention time 17.8 min. 139

Figure S108. MS spectrum extracted from LA control chromatogram at retention time 19.7 min. 140

Figure S109. MS spectrum extracted from LA control chromatogram at retention time 21.0 min. 141

Figure S110. MS spectrum extracted from LA control chromatogram at retention time 21.2 min. 142

Figure S111. MS spectrum extracted from LA control chromatogram at retention time 22.2 min. 143

Figure S112. MS spectrum extracted from LA control chromatogram at retention time 22.4 min. 144

Figure S113. MS spectrum extracted from LA control chromatogram at retention time 23.3 min. 145

Figure S114. MS spectrum extracted from LA control chromatogram at retention time 23.5 min. 146

Figure S115. MS spectrum extracted from LA control chromatogram at retention time 24.4 min. 147

Figure S116. MS spectrum extracted from LA control chromatogram at retention time 24.6 min. 148

Figure S117. MS spectrum extracted from LA control chromatogram at retention time 25.2 min. 149

Figure S118. MS spectrum extracted from LA control chromatogram at retention time 25.7 min. 150

Figure S119. MS spectrum extracted from LA control chromatogram at retention time 25.9 min. 151

Figure S120. MS spectrum extracted from LA control chromatogram at retention time 26.6 min. 152

Figure S121. MS spectrum extracted from LA control chromatogram at retention time 27.1 min. 153

Figure S122. MS spectrum extracted from LA control chromatogram at retention time 27.6 min. 154

Figure S123. MS spectrum extracted from LA control chromatogram at retention time 28.1 min. 155

Figure S124. MS spectrum extracted from LA control chromatogram at retention time 28.5 min. 156

Figure S125. MS spectrum extracted from LA control chromatogram at retention time 28.9 min. 157

Figure S126. MS spectrum extracted from LA control chromatogram at retention time 29.1 min. 158

Table S7. Identification of GA reaction products (in the absence of DA). 159

Figure S127. MS spectrum extracted from GA control chromatogram at retention time 13.1 min. 160

Figure S128. MS spectrum extracted from GA control chromatogram at retention time 14.2 min. 161

Figure S129. MS spectrum extracted from GA control chromatogram at retention time 15.2 min. 162

Figure S130. MS spectrum extracted from GA control chromatogram at retention time 16.2 min. 163

Figure S131. MS spectrum extracted from GA control chromatogram at retention time 17.0 min. 164

Figure S132. MS spectrum extracted from GA control chromatogram at retention time 17.8 min. 165

Figure S133. MS spectrum extracted from GA control chromatogram at retention time 18.5 min. 166

Figure S134. MS spectrum extracted from GA control chromatogram at retention time 19.2 min. 167

Figure S135. MS spectrum extracted from GA control chromatogram at retention time 19.7 min. 168

Figure S136. MS spectrum extracted from GA control chromatogram at retention time 20.2 min. 169

Figure S137. MS spectrum extracted from GA control chromatogram at retention time 20.7 min. 170

Table S8. Identification of MA reaction products (in the absence of DA). 171

Figure S138. MS spectrum extracted from MA control chromatogram at retention time 7.7 min.. 172

Figure S139. MS spectrum extracted from MA control chromatogram at retention time 8.7 min.. 173

Figure S140. MS spectrum extracted from MA control chromatogram at retention time 11.3 min. 174

Figure S141. MS spectrum extracted from MA control chromatogram at retention time 12.5 min. 175

Figure S142. MS spectrum extracted from MA control chromatogram at retention time 12.8 min. 176

Figure S143. MS spectrum extracted from MA control chromatogram at retention time 13.1 min. 177

Figure S144. MS spectrum extracted from MA control chromatogram at retention time 13.4 min. 178

Figure S145. MS spectrum extracted from MA control chromatogram at retention time 13.6 min. 179

Figure S146. MS spectrum extracted from MA control chromatogram at retention time 13.9 min. 180

Figure S147. MS spectrum extracted from MA control chromatogram at retention time 14.1 min. 181

Figure S148. MS spectrum extracted from MA control chromatogram at retention time 14.5 min. 182

Figure S149. MS spectrum extracted from MA control chromatogram at retention time 14.9 min. 183

Figure S150. MS spectrum extracted from MA control chromatogram at retention time 15.0 min. 184

Figure S151. MS spectrum extracted from MA control chromatogram at retention time 15.6 min. 185

Figure S152. MS spectrum extracted from MA control chromatogram at retention time 16.0 min. 186

Table S9. Identification of PLA reaction products (in the absence of DA). 187

Figure S153. MS spectrum extracted from PLA control chromatogram at retention time 23.1 min. 188

Figure S154. MS spectrum extracted from PLA control chromatogram at retention time 26.7 min. ... 189

Figure S155. MS spectrum extracted from PLA control chromatogram at retention time 29.0 min. ... 190

Figure S156. MS spectrum extracted from PLA control chromatogram at retention time 29.8 min. ... 191

Figure S157. MS spectrum extracted from PLA control chromatogram at retention time 30.6 min. ... 192

Figure S158. FTIR spectrum of fresh DA monomers. ... 193

Figure S159. FTIR spectrum of fresh GA monomers. ... 194

Figure S160. FTIR spectrum of fresh LA monomers. ... 195

Figure S161. FTIR spectrum of fresh MA monomers. ... 196

Figure S162. FTIR spectrum of fresh PLA monomers. ... 197

Figure S163. FTIR spectra of DA and GA fresh monomers and reaction products. ... 198

Figure S164. FTIR spectra of DA and LA fresh monomers and reaction products. 199

Figure S165. FTIR spectra of DA and MA fresh monomers and reaction products. F ... 200

Figure S166. FTIR spectra of DA and PLA fresh monomers and reaction products. ... 201

Figure S167. ^{1}H-NMR spectrum of DA fresh monomer in $CDCl_3$. ... 202

Figure S168. ^{1}H-NMR spectrum of GA fresh monomer in D_2O. ... 203

Figure S169. ^{1}H-NMR spectrum of LA fresh monomer in $CDCl_3$. ... 204

Figure S170. ^{1}H-NMR spectrum of MA fresh monomer in D_2O. ... 205

Figure S171. ^{1}H-NMR spectrum of PLA fresh monomer in $CDCl_3$. ... 206

Figure S172. ^{1}H-NMR spectrum of DA:GA reaction product at 1:1 molar ratio. ... 207

Figure S173. ^{1}H-NMR spectrum of DA:GA reaction product at 1:2 molar ratio. ... 208

Figure S174. ^{1}H-NMR spectrum of DA:GA reaction product at 1:4 molar ratio. 209

Figure S175. ^{1}H-NMR spectrum of DA:LA reaction product at 1:1 molar ratio. 210

Figure S176. ^{1}H-NMR spectrum of DA:LA reaction product at 1:2 molar ratio. 211

Figure S177. ^{1}H-NMR spectrum of DA:LA reaction product at 1:4 molar ratio. 212

Figure S178. ^{1}H-NMR spectrum of DA:MA reaction product at 1:1 molar ratio. ... 213

Figure S179. ^{1}H-NMR spectrum of DA:MA reaction product at 1:2 molar ratio. ... 214

Figure S180. 1H-NMR spectrum of DA:MA reaction product at 1:4 molar ratio. .. 215

Figure S181. ^{1}H-NMR spectrum of DA:PLA reaction product at 1:1 molar ratio. . 216

Figure S182. ^{1}H-NMR spectrum of DA:PLA reaction product at 1:2 molar ratio. . 217

Figure S183. ^{1}H-NMR spectrum of DA:PLA reaction product at 1:4 molar ratio. . 218

Figure S184. ^{1}H-NMR spectrum of GA control reaction products. 219

Figure S185. ^{1}H-NMR spectrum of LA control reaction product. 220

Figure S186. ^{1}H-NMR spectrum of MA control reaction product. 221

Figure S187. ^{1}H-NMR spectrum of PLA control reaction product. 222

Figure S188. DA consumption under dry reaction at 85°C for 7 days determined by HPLC. 223

Figure S189. Calibration curve at 259nm constructed for the determination of PLA conversion. 224

Figure S190. PLA consumption under dry reaction at 85°C for 7 days. 225

Figure S191. Visual appearance of rehydrated DA-HA reaction products at 1:1, 1:2, and 1:4 molar ratio. 226

Figure S192. Turbidity meausrments of the rehydrated DA:HAs reaction products at 1:1, 1:2 and 1:4 molar ratio. 227

Figure S193. DA concentration in the resulting rehydrated products. 228

Figure S194. Fluorescent microscopy images of the rehydrated reaction products obtained by the reaction of DA and the tested HAs at different molar ratios. 229

Figure S195. Visual appearance of DA fresh controls and DA:LA reaction products at 1:1, 1:2, and 1:4 molar ratios. 230

Figure S196. Fluorescent microscopy images of DA fresh controls at decreasing concentrations and DA:LA reaction products at 1:1, 1:2, and 1:4 molar ratios. ... 231

Figure S197. Fluorescent microscopy images , bright-field microscopy images, and visual appearance of LA reaction products at 100 mM and 200 mM in the presence and absence of 50 mM DA. ... 232

Figure S198. Cryo-TEM images of fresh monomers and reaction products of DA:LA at 1:1, 1:2, and 1:4 molar ratios. ... 233

Figure S199. Dynamic light scattering correlograms of DA control, DA-LA fresh monomers, and DA-LA reaction products. ... 234

Figure S200. Mean size and polydispersity obtained for DA-LA reaction products and fresh monomers. ... 235

Figure S201. Size distribution obtained for DA:LA reaction products and fresh monomers at a 1:4 molar ratio. ... 236

Figure S202. Vesicle size distribution derived from cryo-TEM measurements. ... 237

Figure S203. Vesicle size distribution derived from cryo-TEM measurements. ... 238

Figure S204. Determination of the critical aggregation concentration of DA for DA control sample. ... 239

Figure S205. Determination of the critical aggregation concentration of DA for DA:LA reaction product at 1:1 molar ratio. ... 240

Figure S206. Determination of the critical aggregation concentration of DA for DA:LA reaction product at 1:2 molar ratio. ... 241

Figure S207. Determination of the critical aggregation concentration of DA for DA:LA reaction product at 1:4 molar ratio. ... 242

Figure S208. Determination of the critical aggregation concentration of DA for DA:LA fresh monomers at 1:2 molar ratio. ... 243

Figure S209. Determination of the critical aggregation concentration of DA for DA:LA fresh monomers at 1:4 molar ratio. ... 244

Figure S210. Determination of the critical aggregation concentration of DA for a mixture of DA and LA rection product at 1:2 molar ratio referring to initial amount of LA. ... 245

Figure S211. Determination of the critical aggregation concentration of DA for a mixture of DA and LA rection product at 1:4 molar ratio referring to initial amount of LA. 246

Figure S212. DA-LA reaction products exhibit greater permeability compared to the corresponding DA-LA fresh monomers and DA control vesicles. 247

Figure S213. Encapsulation of biologically relevant molecules within DA-LA reaction products. 248

Figure S214. HPLC chromatograms obtained for LA homoesters of DA:LA reaction product at a 1:4 molar ratio at pH 6.8 and 40°C over the course of the degradation study. 249

Figure S215. HPLC chromatograms obtained for DA-LA heteroesters of DA:LA reaction product at a 1:4 molar ratio at pH 6.8 and 40°C over the course of the degradation study. 250

Figure S216. Peak area measured for LA monomer, DA monomer, and the 1DA1LA conjugation prodcut in DA:LA reaction sample at a 1:4 molar ratio over the course of the degradation study. 251

Figure S217. HPLC chromatograms obtained for DA:LA reaction product at a 1:4 molar ratio following incubation at 60°C and rehydration in citrate buffer at pH 5.5. 252

Figure S218. HPLC chromatograms obtained for DA:LA reaction product at a 1:4 molar ratio following incubation at 60°C and rehydration in phosphate buffer at pH 6.8. 253

Figure S219. HPLC chromatograms obtained for DA:LA reaction product at a 1:4 molar ratio following incubation at 60°C and rehydration in tris buffer at pH 8.0. 254

Figure S220. HPLC chromatograms obtained for DA:LA reaction product at a 1:4 molar ratio following incubation at 40°C and rehydration in citrate buffer at pH 5.5. 255

Figure S221. HPLC chromatograms obtained for DA:LA reaction product at 1:4 molar ratio following incubation at 40°C and rehydration in phosphate buffer at pH 6.8. 256

Figure S222. HPLC chromatograms obtained for DA:LA reaction product at 1:4 molar ratio following incubation at 40°C and rehydration in tris buffer at pH 8.0. 257

Figure S223. HPLC chromatograms obtained for DA:LA reaction product at 1:4 molar ratio following incubation at room temperature and rehydration in citrate buffer at pH 5.5. 258

Figure S224. HPLC chromatograms obtained for DA:LA reaction product at 1:4 molar ratio following incubation at room temperature and rehydration in phosphate buffer at pH 6.8. 259

Figure S225. HPLC chromatograms obtained for DA:LA reaction product at 1:4 molar ratio following incubation at room temperature and rehydration in tris buffer at pH 8.0. 260

Figure S226. Relative peak area of LA dimer and trimer as a function of incubation period, pH, and temperature. 261

Figure S227. Relative peak area of DA-LA conjugates as a function of storage period, pH and temperature. 262

Figure S228. HPLC chromatograms obtained for DA:LA reaction product at a 1:4 molar ratio at t0 prior to incubation. 263

Figure S229. HPLC chromatograms obtained for 100mM LA reaction products following incubation at room temperature (RT).. 264

Figure S230. HPLC chromatograms obtained for 200mM LA reaction products following incubation at room temperature (RT).. 265

Figure S231. HPLC chromatograms obtained for 100mM LA reaction products in the presence of DA monomer following incubation at room temperature (RT).. 266

Figure S232. HPLC chromatograms obtained for 200mM LA reaction products in the presence of DA monomer following incubation at room temperature (RT).. 267

Figure S233. HPLC chromatograms obtained for OcA:LA reaction product at 1:4 molar ratio following incubation at 40°C and rehydration in phosphate buffer at pH 6.6. 268

Figure S234. Relative peak area of LA and OcA-LA conjugates as a function of storage period. 269

Figure S235. HPLC chromatograms obtained for dDA:LA reaction product at 1:4 molar ratio following incubation at 40°C and rehydration in phosphate buffer at pH 7.4. 270

Figure S236. Relative peak area of LA and dDA-LA conjugates as a function of storage period. 271

Figure S237. HPLC chromatograms obtained for DA:LA:GA reaction product at 1:2:2 molar ratio following incubation at 40°C and rehydration in phosphate buffer at pH 6.8. 272

273

Figure S238. LC-MS chromatograms obtained for DA:LA:GA reaction product at 1:2:2 molar ratio following incubation at 40°C and rehydration in phosphate buffer at pH 6.8. 273

Figure S239. HPLC chromatograms obtained for DA:PLA reaction product at 1:1 molar ratio following incubation at 40°C and rehydration in tris buffer at pH 8.0.. 274

Figure S240. Relative peak area of PLA and DA-PLA conjugates as a function of storage period. 275

Figure S241. OcA and dDA consumption under dry reaction at 85°C for 7 days as determined by HPLC. 276

Figure S242. Determination of the critical aggregation concentration of fresh monomers and reaction products of different fatty acids. 277

Figure S243. Determination of the critical aggregation concentration of OcA for OcA control sample. 278

Figure S244. Determination of the critical aggregation concentration of OcA for OcA:LA reaction product at 1:4 molar ratio. 279

Figure S245. Determination of the critical aggregation concentration of dDA for dDA control sample. 280

Figure S246. Determination of the critical aggregation concentration of dDA for dDA:LA reaction product at 1:4 molar ratio. 281

Figure S247. Determination of the critical aggregation concentration of DA for DA:PLA fresh monomers at 1:1 molar ratio. 282

Figure S248. Determination of the critical aggregation concentration of DA for DA:PLA reaction product at 1:1 molar ratio. 283

Materials and methods

Materials

Decanoic acid (C1875), Dodecanoic acid (L4250), Octanoic acid (O3907) Glycolic acid (124737), L-Lactic acid (199257, purity 85%), L-Malic acid (M1000), 3-L-Phenyllactic acid (113069) and 2-Hydroxycaproic acid (219819) were purchased from Merck. Chloroform-D_3 (99.8%, 300816) and D_2O (99.9%, 301496) were purchased from ZEOtope. Hydrochloric acid 37% (320331), sodium hydroxide (S8045), sodium dihydrogen phosphate monohydrate (1.06346), and citric acid (27109) were purchased from Sigma-Merck. Tris(hydroxymethyl)aminomethane (17-1321-01) was purchased from Cativa., merocyanine 450, (dye content 90%, 323756), rhodamine 6G (dye content 99%, 252433), calcein (C0875), Poly-Lys-FITC labelled (P3069, molecular weight 30,000-70,000, FITC/Lys monomer 0.003-0.010) and Sepharose™ 4B (45-165 µm bead diameter, 4B200) were purchased from Sigma-Merck. 56FAM ssDNA-10-mer (5’-|56-FAM/CGCTAAATCG-3’, Mw 3549.5 g/ mol) was purchased from (IDT, Integrated DNA Technologies). Triton x-100 (X198-07) was purchased from J.T. Baker. Solvents used for LC-MS analyses were of LC-MS grade. Double distilled water (conductivity ≤20µS/cm) was used for structural characterization.

Methods

Single-step dry reactions.

Binary mixtures of decanoic acid (DA) and hydroxy acids (HAs) at 1:1, 1:2, and 1:4 molar ratios were prepared in 7mL scintillation vials. DA’s amount was fixed at 200 µmol and HAs’s amount was adjusted accordingly. All components were weighed except for lactic acid (LA) which was added volumetrically. The mixtures were placed at 85 °C for seven days. Samples of either DA (200 µmol) alone or hydroxy acids (400 µmol) alone were prepared as control. An aliquot of 1M HCl (1 µmol) was added to the DA control. All samples were prepared in triplicates. For binary mixtures of octanoic acid (OcA) and LA, 600µmol OcA and 2400µmol LA were used. For binary mixtures of dodecanoic acid (dDA) and LA, 100µmol dDA and 400µmol LA were used. For ternary mixtures of DA, glycolic acid (GA) and LA, 200µmol DA, 400µmol GA, and 400µmol LA were used.

Poly-Lys encapsulation.

The encapsulation of poly-Lys-FITC was carried out using the reaction products of DA:LA at a 1:4 molar ratio. For sample preparation, the dry reaction products were dissolved in acetonitrile to a final concentration of 100 mM. Then, aliquots of 100 µL (corresponding to 10 µmol DA and 40 µmol LA) were withdrawn, and acetonitrile was removed under vacuum at 30°C. Then, poly-Lys (100 µM solution) was added to the dry DA-LA mixture, followed by the addition of phosphate monobasic and sodium hydroxide. Due to precipitation of poly-Lys, pH was roughly adjusted to 6.8 ± 0.2 using pH indicator strips. Then, the sample was centrifuged at 10,000 rpm for 2 min and the supernatant was examined under a fluorescence microscope (FITC filter, EX: 470/40, EM: 525/50, DM: 495).

High-Performance Liquid Chromatography / LC-MS.

HPLC analyses were conducted using an Agilent 1260 quaternary pump and autosampler (Agilent Technologies, Santa Clara, CA, USA) with a DAD UV-vis detector at 210nm and 259nm. LC-MS data were collected using an Agilent G6135C single quadrupole mass spectrometer with a capillary voltage of 4.0 kV and a source fragmentation voltage of 70 V. Scan range: 50-1500 m/z. Chromatographic separation was achieved using InfinityLab Poroshell 120 EC-C18 column (150 x 3.0 mm, 2.7µm, with a SecurityGuard™ C18 4x2.0mm), at a constant 0.3 mL/min flow rate. Column cell temperature was maintained at 20 °C. Gradient elution was carried out using (A) 0.1% formic acid in water and (B) acetonitrile as follow: 5min 100% A, 20min ramp to 20% A, 10min 100% B.

For the identification of the reaction products obtained with phenyllactic acid (PLA), gradient elution was carried out using (A) 0.1% formic acid in water and (B) 0.1% formic acid in acetonitrile as follow: 5min 100% A, 20min ramp to 20% A, 10min 100% B.

For the identification of the reaction products obtained with decanoic acid and lactic acid, chromatographic separation was achieved using InfinityLab Poroshell 120 EC-C18 column (150 x 3.0 mm, 2.7µm, with a SecurityGuard™ C18 4x2.0mm), at constant 0.5 mL/min flow rate. Column cell temperature was maintained at 30 °C. Gradient elution was carried out using (A) 0.1% formic acid in water and (B) acetonitrile as follows: 3min 90% A, 3min ramp to 20% A, 1.5min 20% A, 7.5min ramp to 100% B, 5min 100% B.

For the identification and qualitative analyses of the reaction products obtained with dodecanoic acid, chromatographic separation was achieved using InfinityLab Poroshell 120 EC-C18 column (150 x 3.0 mm, 2.7µm, with a SecurityGuard™ C18 4x2.0mm), at a constant 0.4 mL/min flow rate. Column cell temperature was maintained at 20 °C. Gradient elution was carried out using (A) 0.1% formic acid in water and (B) acetonitrile as follows: 5min 95% A, 7min ramp to 30% A, 10min ramp to 100% B, 8min 100% B.

For the identification and qualitative analyses of the reaction products obtained with octanoic acid, chromatographic separation was achieved using InfinityLab Poroshell 120 EC-C18 column (150 x 3.0 mm, 2.7µm, with a SecurityGuard™ C18 4x2.0mm), at a constant 0.3 mL/min flow rate. Column cell temperature was maintained at 20 °C. Gradient elution was carried out using (A) 0.1% formic acid in water and (B) acetonitrile as follows: 5min 90% A, 5min ramp to 55% A, 20min ramp to 100% B.

For the identification and qualitative analyses of the reaction products obtained with decanoic acid and α-leucic acid, chromatographic separation was achieved using InfinityLab Poroshell 120 EC-C18 column (150 x 3.0 mm, 2.7µm, with a SecurityGuard™ C18 4x2.0mm), at a constant 0.4 mL/min flow rate. Column cell temperature was maintained at 20 °C. Gradient elution was carried out using (A) 0.1% formic acid in water and (B) acetonitrile as follows: 3min 90% A, 5min ramp to 50% A, 6min ramp to 30% A, 10min ramp to 5% A, 11 min 5%A.

For the hydrolysis study analyses of the reaction products obtained with decanoic acid and phenyllactic acid, chromatographic separation was achieved using InfinityLab Poroshell 120 EC-C18 column (150 x 3.0 mm, 2.7µm, with a SecurityGuard™ C18 4x2.0mm), at a constant 0.5 mL/min flow rate. Column cell temperature was maintained at 20 °C. Gradient elution was carried out using (A) 0.1% formic acid in water and (B) acetonitrile as follows: 3min 40% A, 4min ramp to 100% B, 10min 100% B.

Critical aggregation concentration (CAC) determination.

The critical aggregation concentration of DA in fresh controls and reaction products was determined using the Merocyanine 540 assay. To that end, dilution lines were prepared by diluting stocks of fresh monomers or reaction products (see sample rehydration) with 50 mM phosphate buffer (final concentration) at pH 6.8. The dilution lines were constructed to cover a range of DA concentrations that are below and above the CAC. The

resulting dilutions were mixed with Merocyanine 540 stock of 1mg/mL, resulting in a final Merocyanine 540 concentration of 20μg/mL. 150μL sample were placed in 96-wells black plate. Spectra were recorded on a Synergy H1 plate reader (BioTek Instruments, VT, USA) between 400 nm to 620 nm. The ratio between the absorption at 570 nm and 530 nm was calculated and plotted against DA concentration. The point at which the ratio increases significantly was considered as the CAC. The determined values were extracted from the intersection between the fitting lines at the first two regions of the curves.
The critical aggregation concentration of OcA and dDA was determined in a similar way with one exception of pH and buffer concentration. For OcA, CAC was determined at pH 6.6 and 200mM phosphate buffer and for dDA at pH 7.4 and 50mM phosphate buffer.
For DA:PLA fresh monomers and reaction product at 1:1 molar ratio, CAC was determined at pH 6.8 and 50mM phosphate buffer.

Hydrolysis and degradation experiments.

The hydrolysis and degradation profile of the dried products obtained in the drying reactions was evaluated for the products of DA:LA binary mixture at 1:4 molar ratio and for LA control reaction products. Crude reaction products were rehydrated in water and with either 1M solutions of tris, phosphate monobasic, or citric acid, and the pH of the samples was further adjusted to 8.0, 6.8, or 5.5, respectively. DA and LA concentrations were 50 mM and 200 mM, respectively, referring to initial amounts prior to the reaction. The buffer concentration was 50 mM. The resulting rehydrated samples were dispensed into eight 500 μL aliquots in 1.5 mL microtubes. Each sample was withdrawn at different time points. Samples were stored at various temperatures for different periods of time as follows: at room temperature (23 °C ±3 °C) for 7, 14, 21, 28, 35, 42, and 49 days. At 40 °C for 4, 8, 12, 16, 20, 24, and 28 days. At 60 °C for 2, 4, 6, 8, 10, 12, and 14 days. In addition, t0 samples were withdrawn immediately after sample preparation. All withdrawn samples were stored at -80 °C until being analyzed by HPLC/ LC-MS. Prior to HPLC analyses, the pH of each sample was roughly measured using a pH Indicator Strip, universal Specification (0 - 14.0) (Millipore, Merck). For HPLC analyses, samples were diluted ten times fold in ACN:water 50:50. To confirm that no degradation occurred during the analysis itself, representative samples were injected several times during the course of the analysis.

An additional hydrolysis experiment was conducted by rehydrating the reaction product of DA:LA at a 1:4 molar ratio in water and phosphate buffer at pH 6.8. The resulting rehydrated product was divided into thirteen 300 μL aliquots in 1.5 mL microtube. Microtubes were placed at 40 °C and samples were withdrawn immediately after preparation and after 1, 2, 3, 4, 6, 8, 12, 16, 20, 24, 48, and 72hr. The withdrawn samples were treated as described above.

Control samples of LA reaction products using the initial amount of either 400 μmol or 800 μmol were tested for hydrolysis as well. The dry reaction products were rehydrated in water and phosphate buffer and the pH was adjusted to 6.8. The final concentration of LA was either 100 mM or 200 mM, referring to initial amounts prior to the reaction. The rehydrated products were stored at RT and samples of 800 μL were withdrawn immediately after preparation and after 12hr and 4 days. The withdrawn samples were treated as described above.

The hydrolysis of additional fatty acids and hydroxy acids was carried out as well. Reaction products of OcA:LA, at 1:4 molar ratio were rehydrated in water and phosphate buffer (200mM) at pH 6.6. The concentration of OcA and LA was 150mM and 600mM, respectively, referring to the initial quantity prior to reaction initiation. Reaction products of dDA:LA, at 1:4 molar ratio, were rehydrated in water and phosphate buffer (50mM) at pH 7.4. The concentration of dDA and LA was 20mM and 80mM, respectively, referring to the initial quantity prior to reaction initiation. Reaction products of DA:LA:GA at 1:2:2 molar ratio were rehydrated in water and phosphate buffer (50mM) at pH 6.8. The concentration of DA, LA and GA was 50mM, 100mM and 100mM respectively, referring to initial quantity prior to reaction initiation. The rehydrated products were divided into eleven 350 μL aliquots in 1.5 mL microtube. Microtubes were placed at 40 °C and samples were withdrawn immediately after preparation and after 1, 2, 3, 4, 6, 8, 12, 24, 48, and either 72hr or 96hr. The withdrawn samples were treated as described above. Reaction products of DA:PLA at 1:1 molar ratio were rehydrated in water containing tris buffer (50mM) at pH 8.0. The concentrations of DA and PLA were 50mM, referring to the initial quantity prior to reaction initiation. The rehydrated products were divided into eleven 350 μL aliquots in 1.5 mL microtube. Microtubes were placed at 40 °C and samples were withdrawn immediately after preparation and after 1, 2, 4, 8, 12, 24, 48, 72hr, 120 and 192 hr. The withdrawn samples were treated as described above.

Dynamic light scattering (DLS) measurements.

Mean particle size, polydispersity index, and size distribution were determined based on dynamic light scattering measurements. All measurements were carried out using a Nano-ZS instrument (Malvern Panalytical, Malvern, UK) equipped with a laser at a wavelength of 633 nm and a back scattering detector (173°). All measurements were carried out at 25°C following a 120 sec equilibration step. Five repeat measurements were carried out for each sample. Instrument settings were automatically optimized. All DLS experiments were carried out in triplicates. Data analysis was carried out using Zetasizer software according to the details provided below.

In DLS measurements, the light scattered from particles dispersed in a liquid medium is measured over time (within microseconds). When the dispersed particles undergo Brownian motion, their motion causes fluctuations in the scattered light in a time-dependent manner which can be described by the correlation function:

$$G(\tau) = \langle I(t) \cdot I(t+\tau) \rangle \ (1)$$

Where I is the intensity of light, t is the time and τ is the delay time.

For particles of relatively uniform size the correlation function can be described by a single exponential decay:

$$G_2(\tau) = A\left[1 + B \cdot e^{-2\Gamma\tau+\mu\tau^2}\right] \ (2)$$

Where $\Gamma\tau$ is light scattering decay:

$$\Gamma = D_T q^2 \ (3)$$

The translation diffusion, D_T and Light scattering, q, are described by Stocks-Einstein equation and Bragg's law:

$$D_T = \frac{k_B T}{6\pi\eta R_H} \ (4)$$

$$q = \frac{4\pi n}{\lambda} \sin\left(\frac{\theta}{2}\right) \ (5)$$

Where D_T is the translational diffusion coefficient, k_B is Boltzmann constant, T is the temperature, η is medium viscosity and R_H is particle hydrodynamic radius, n is dispersant refractive index, λ is wavelength and θ is the scattering angle.

The equation is further treated using the Cumulant approach which provides the first Cumulant moment (a_1) and the second Cumulant moment (a_2) that are used to calculate the intensity-weighted Z average mean size (Z-ave) and the polydispersity index (PDI)

$$y(\tau) = \frac{1}{2}\ln[G_2(\tau) - A] \cong \frac{1}{2}\ln[AB] - \langle\Gamma\rangle\tau + \frac{\mu_2}{2}\tau^2 = a_0 - a_1\tau + a_2\tau^2 \quad (6)$$

Combining equations (3),(4),(5) and (6) we obtain:

$$Z_{ave} = \frac{1}{a_1}\frac{k_B T}{3\pi\eta}\left[\frac{4\pi\tilde{n}}{\lambda_0}\sin\left(\frac{\theta}{2}\right)\right]^2$$

$$PDI = \frac{2a_2}{{a_1}^2}$$

The Cumulant analysis described above is a good approximation for samples with correlograms showing a single decay. In our case, all tested samples with one exception met this criterion and therefore were analyzed using the Cumulant algorithm. The only exception was DA:LA reaction products at a 1:4 molar ratio, for which the correlogram exhibited two distinct decays and therefore was analyzed with the distribution algorithm. The distribution analysis provides size distributions based on a non-negative least squares fitting algorithm included in the Zetasizer software. In general, a fitting function (which is the summation of single exponential functions containing a factor Ai, that represent the area under the curve of the individual exponent) is defined to fit the measured data by minimizing the deviation of the fitted data from the measured data. The factor Ai is calculated by the non-negative least squares algorithm which is then plotted against the size to produce the intensity-weighted size distribution. The volume-weighted and number-weighted distributions are then calculated from the intensity-weighted size distribution using Mie theory.

Additional information about the method and calculations is available on https://www.malvernpanalytical.com/en.

Supplementary Figures

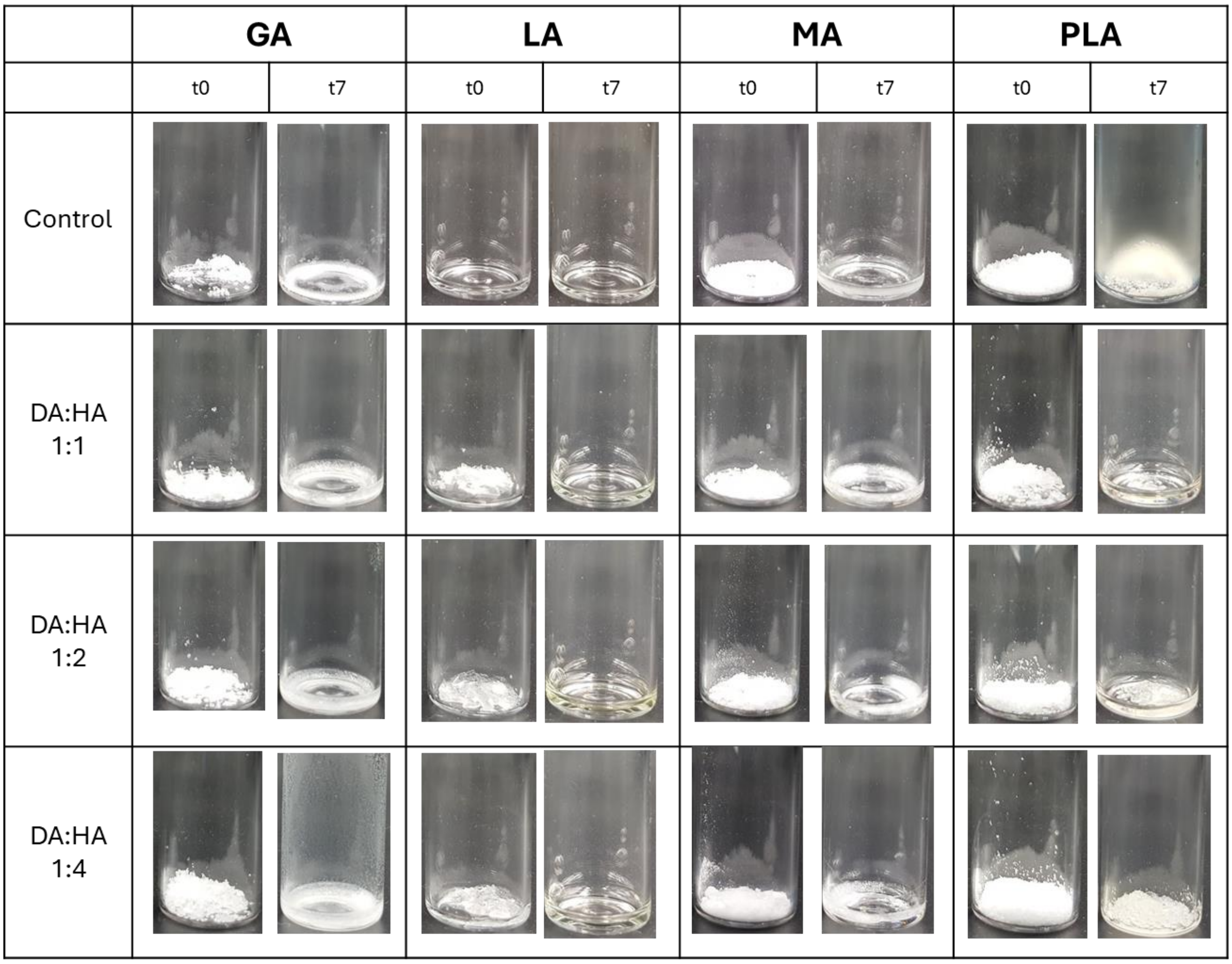

Figure S1. Visual appearance of the binary mixtures of DA and the tested HAs at 1:1, 1:2, and 1:4 molar ratios and HAs control samples. The images on the left and right represent the sample prior to reaction initiation and after reaction termination respectively (t7 – seven days).

A

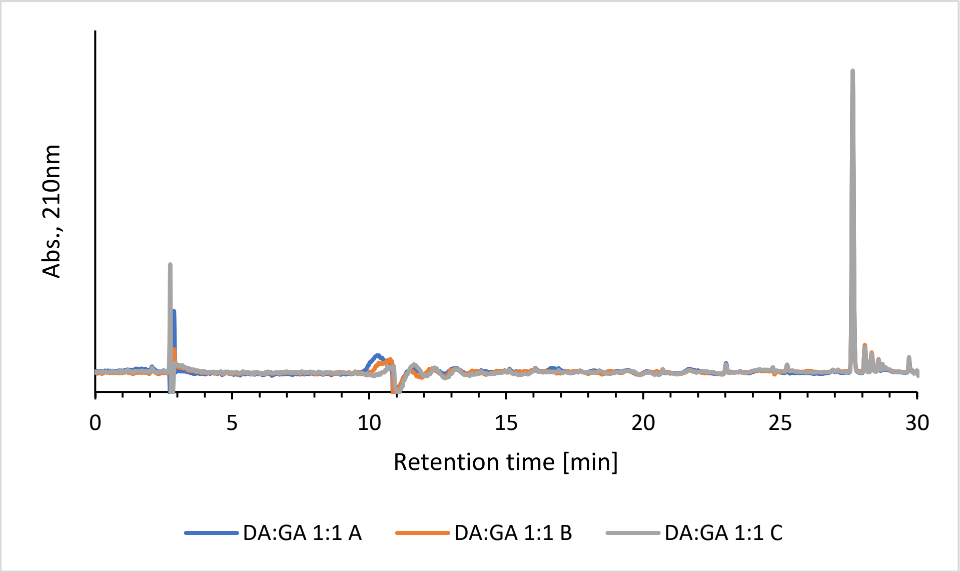

B

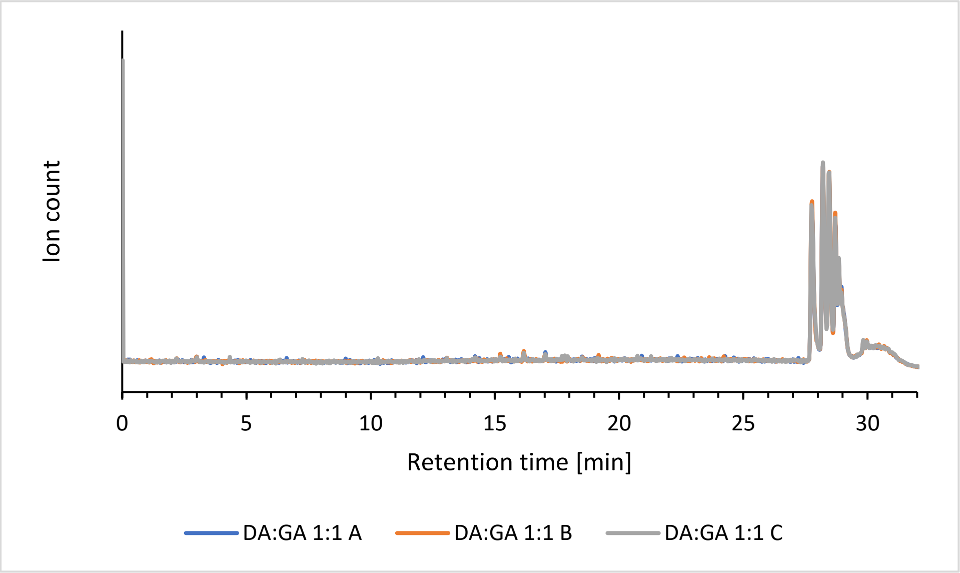

Figure S2. LC-MS analysis confirms the formation of products obtained by the reaction of DA and GA at a 1:1 molar ratio. DA and GA reacted under dry conditions at 85 °C for 7 days and the reaction products were analyzed by HPLC at 210nm (A) and LC-MS operating at negative mode (B). Chromatograms were obtained for three individual preparations.

A

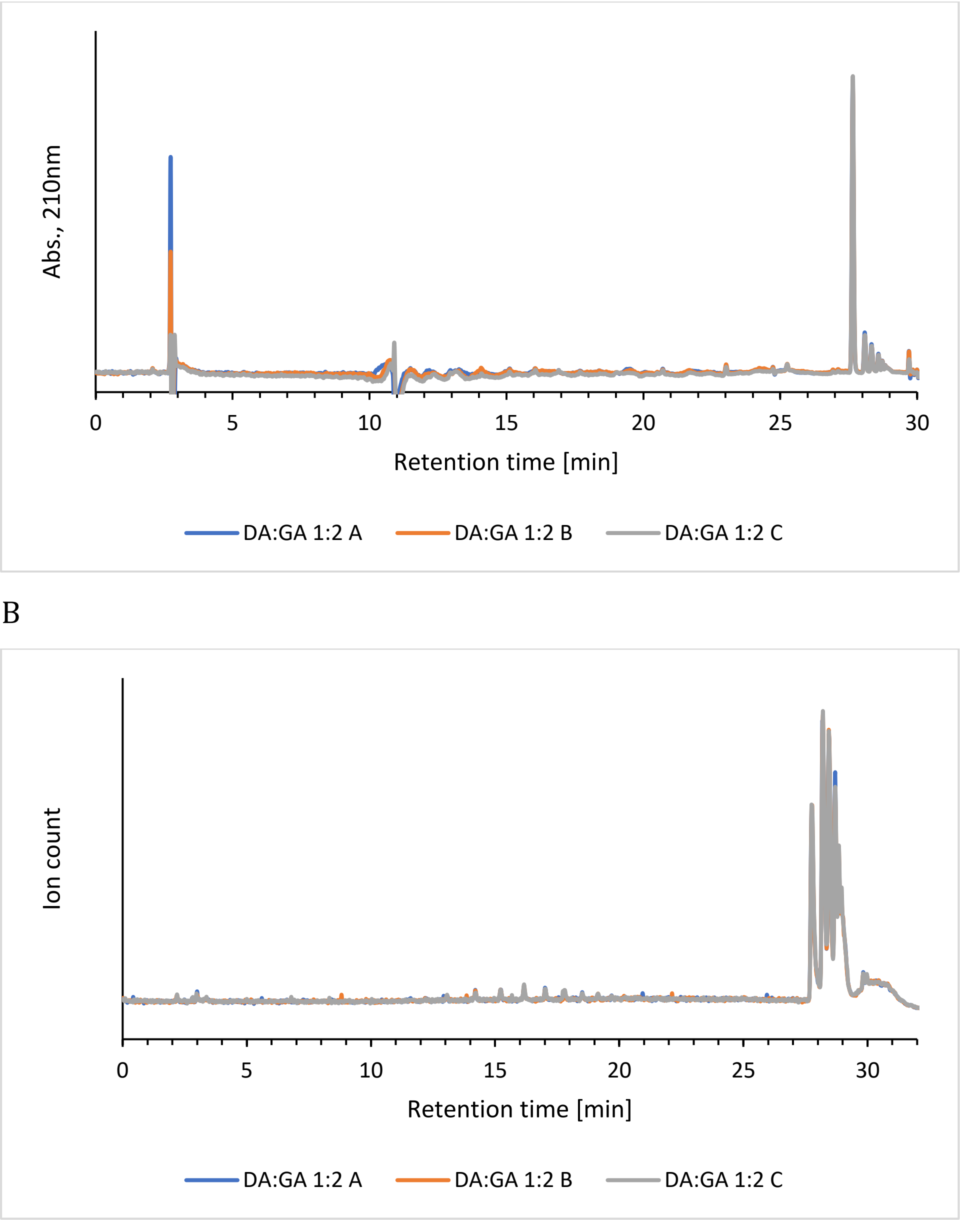

Figure S3. LC-MS analysis confirms the formation of products obtained by the reaction of DA and GA at a 1:2 molar ratio. DA and GA reacted under dry conditions at 85 °C for 7 days and the reaction products were analyzed by HPLC at 210nm (A) and LC-MS operating at negative mode (B). Chromatograms were obtained for three individual preparations.

A

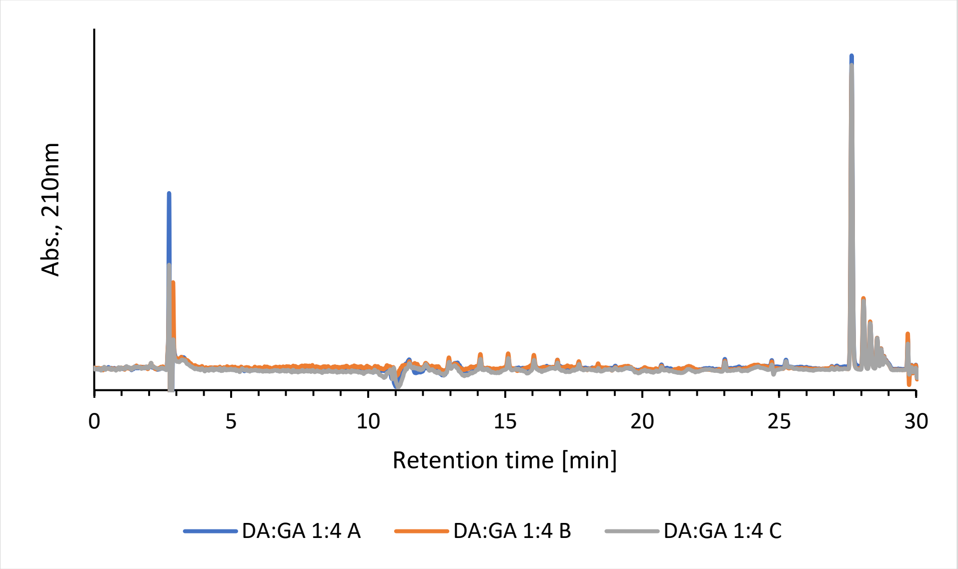

B

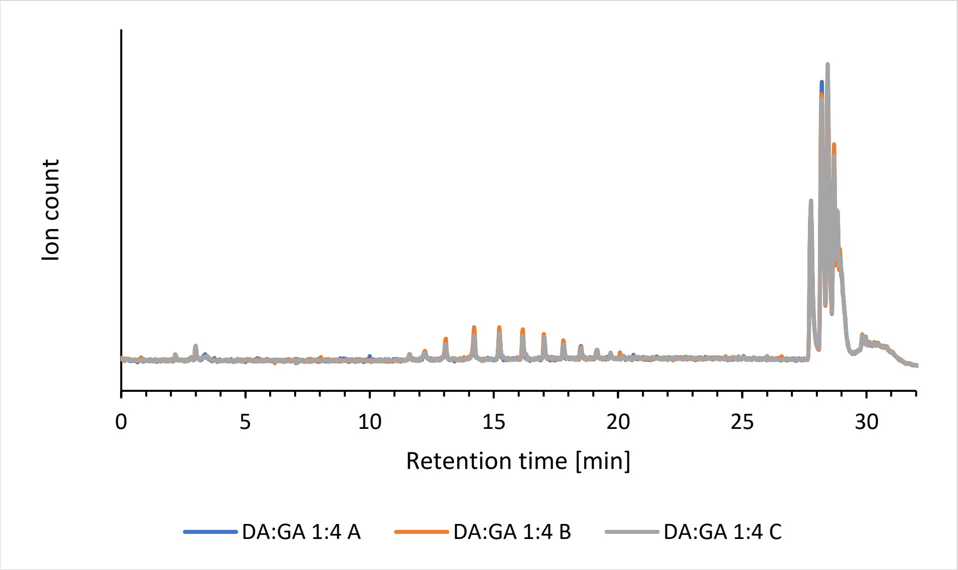

Figure S4. LC-MS analysis confirms the formation of products obtained by the reaction of DA and GA at a 1:4 molar ratio. DA and GA reacted under dry conditions at 85 °C for 7 days and the reaction products were analyzed by HPLC at 210nm (A) and LC-MS operating at negative mode (B). Chromatograms were obtained for three individual preparations.

A

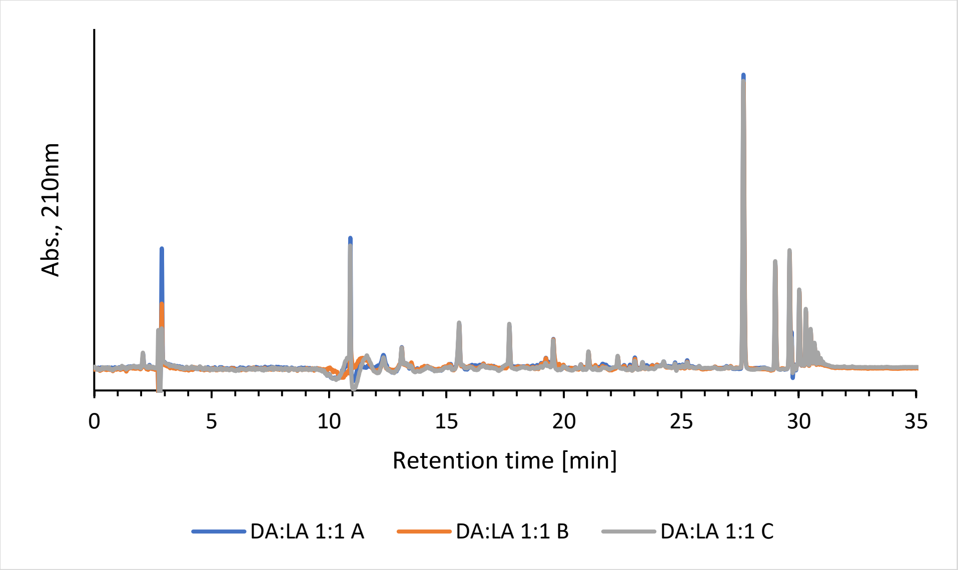

B

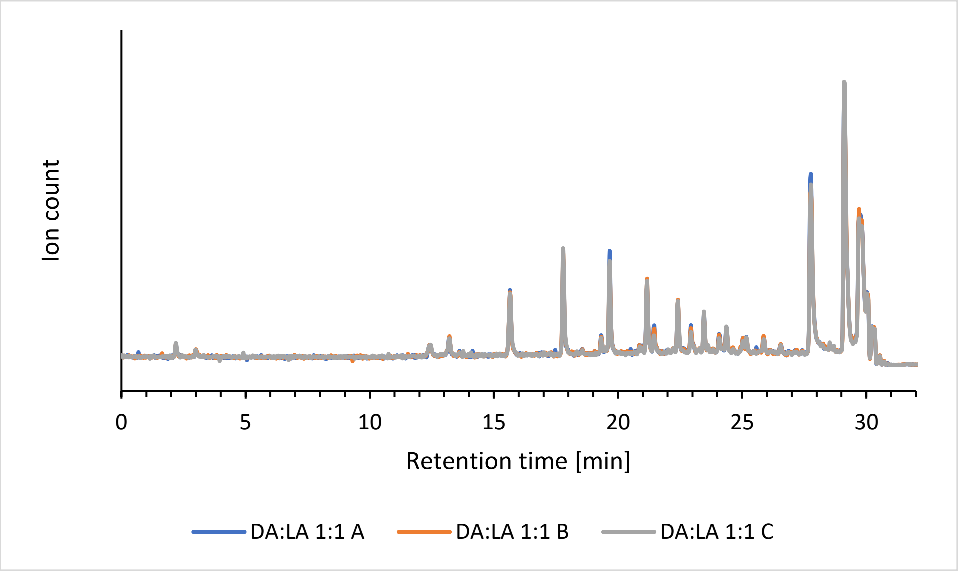

Figure S5. LC-MS analysis confirms the formation of products obtained by the reaction of DA and LA at a 1:1 molar ratio. DA and LA reacted under dry conditions at 85 °C for 7 days and the reaction products were analyzed by HPLC at 210nm (A) and LC-MS operating at negative mode (B). Chromatograms were obtained for three individual preparations.

A

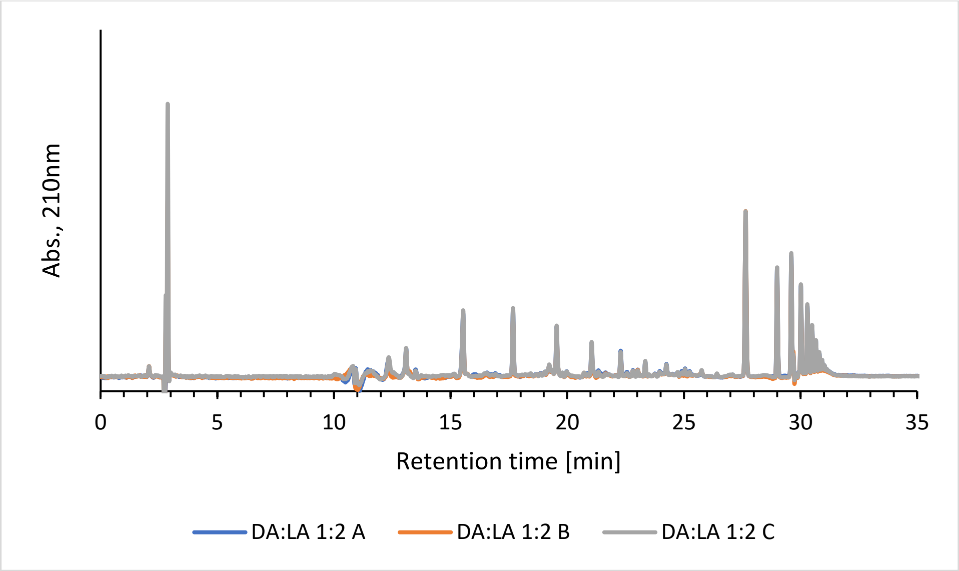

B

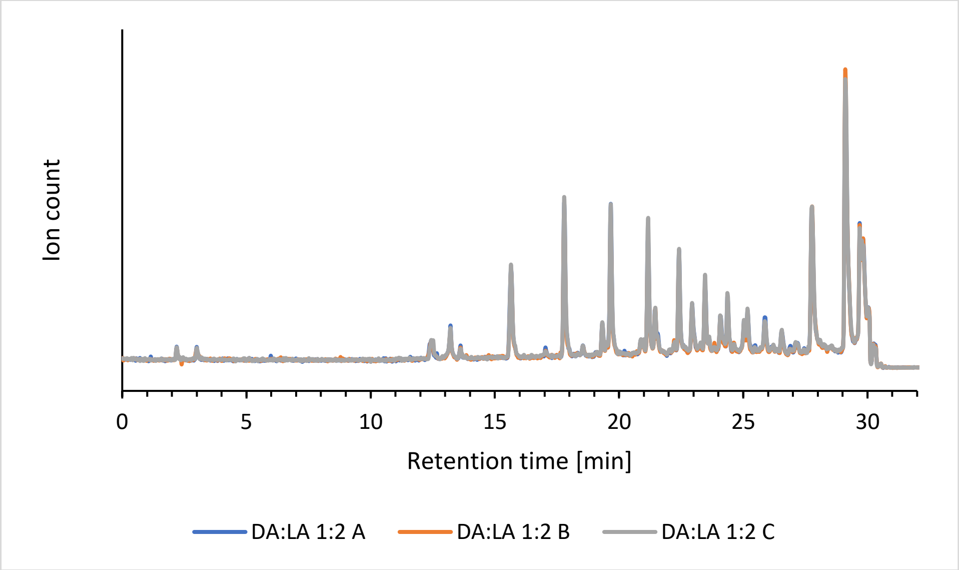

Figure S6. LC-MS analysis confirms the formation of products obtained by the reaction of DA and LA at a 1:2 molar ratio. DA and LA reacted under dry conditions at 85 °C for 7 days and the reaction products were analyzed by HPLC at 210nm (A) and LC-MS operating at negative mode (B). Chromatograms were obtained for three individual preparations.

A

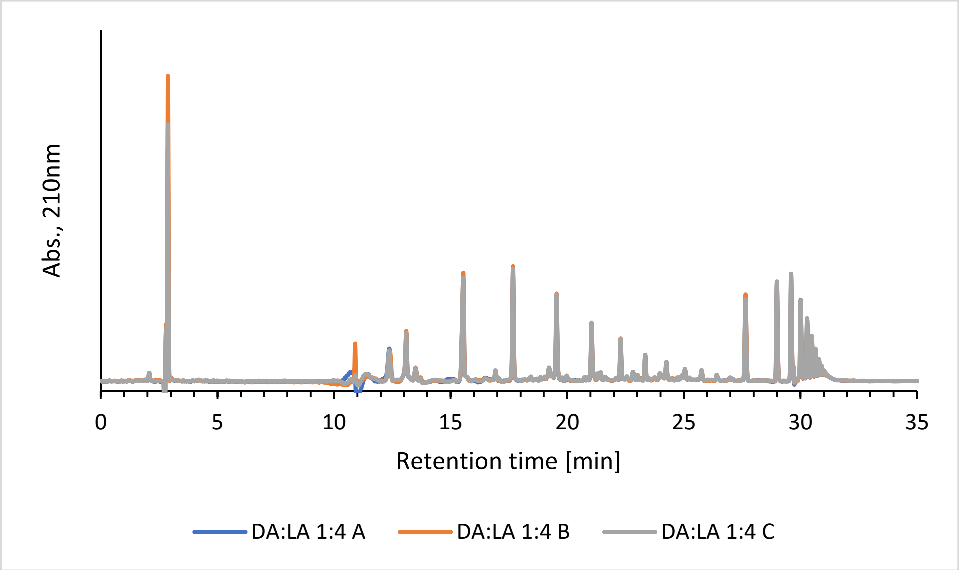

B

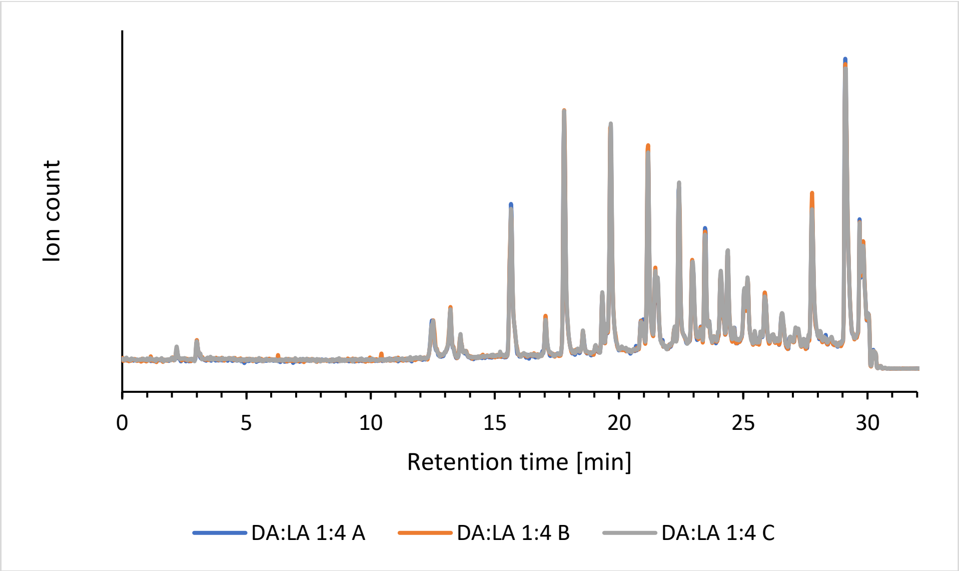

Figure S7. LC-MS analysis confirms the formation of products obtained by the reaction of DA and LA at a 1:4 molar ratio. DA and LA reacted under dry conditions at 85 °C for 7 days and the reaction products were analyzed by HPLC at 210nm (A) and LC-MS operating at negative mode (B). Chromatograms were obtained for three individual preparations.

A

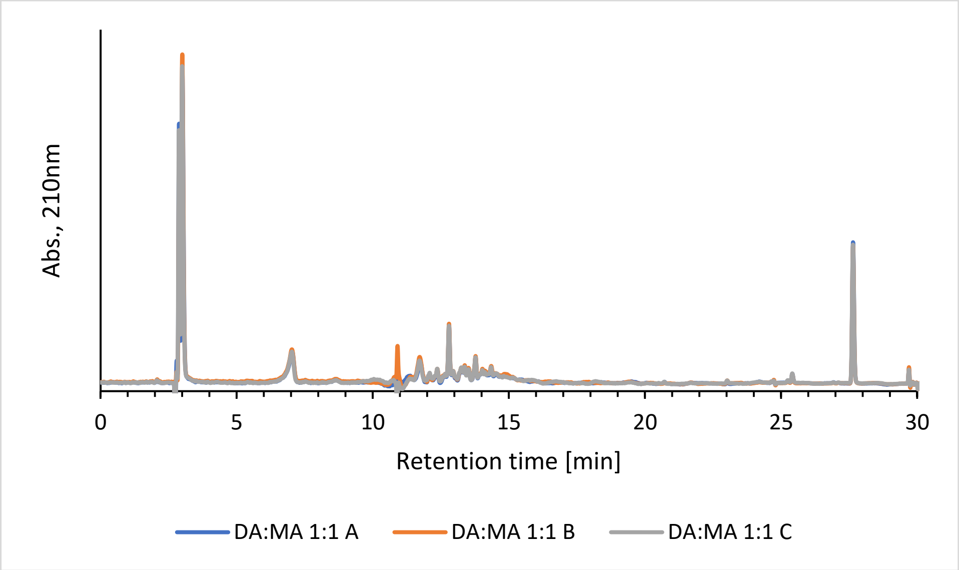

B

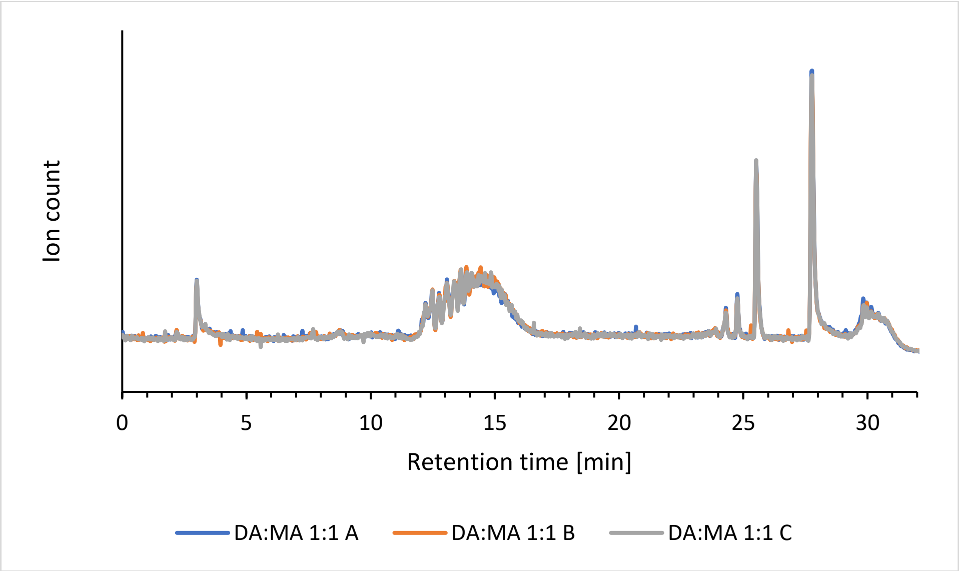

Figure S8. LC-MS analysis confirms the formation of products obtained by the reaction of DA and MA at a 1:1 molar ratio. DA and MA reacted under dry conditions at 85 °C for 7 days and the reaction products were analyzed by HPLC at 210nm (A) and LC-MS operating at negative mode (B). Chromatograms were obtained for three individual preparations.

A

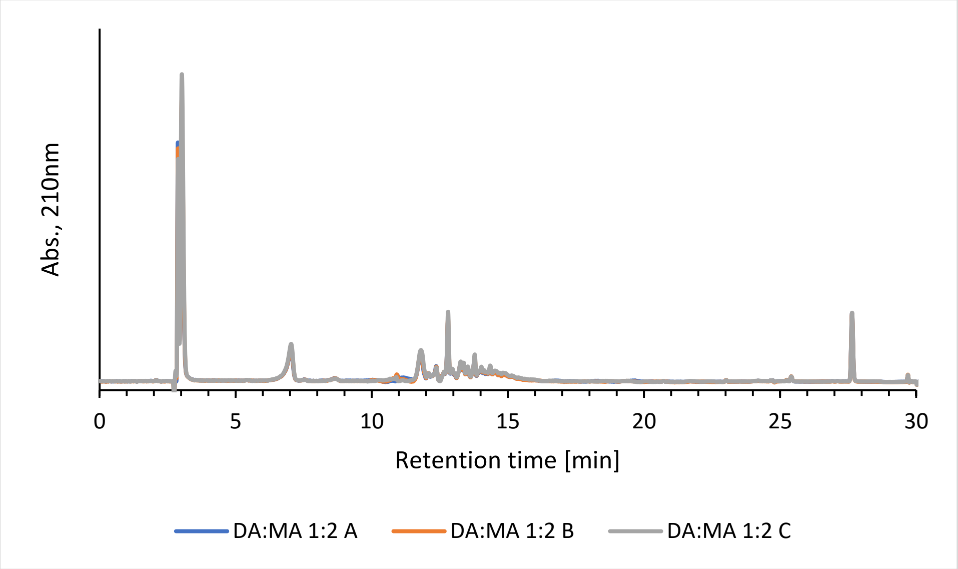

B

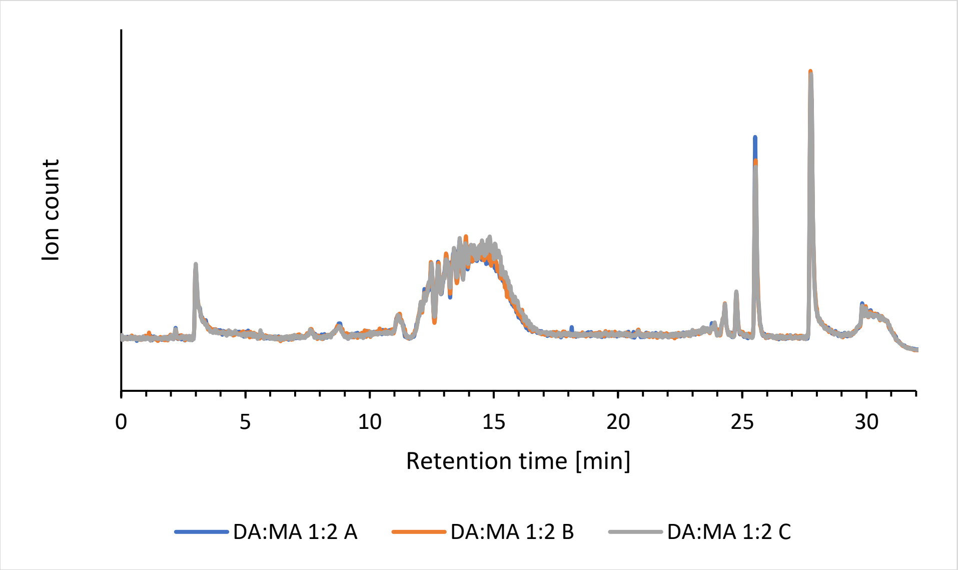

Figure S9. LC-MS analysis confirms the formation of products obtained by the reaction of DA and MA at a 1:2 molar ratio. DA and MA reacted under dry conditions at 85 °C for 7 days and the reaction products were analyzed by HPLC at 210nm (A) and LC-MS operating at negative mode (B). Chromatograms were obtained for three individual preparations.

A

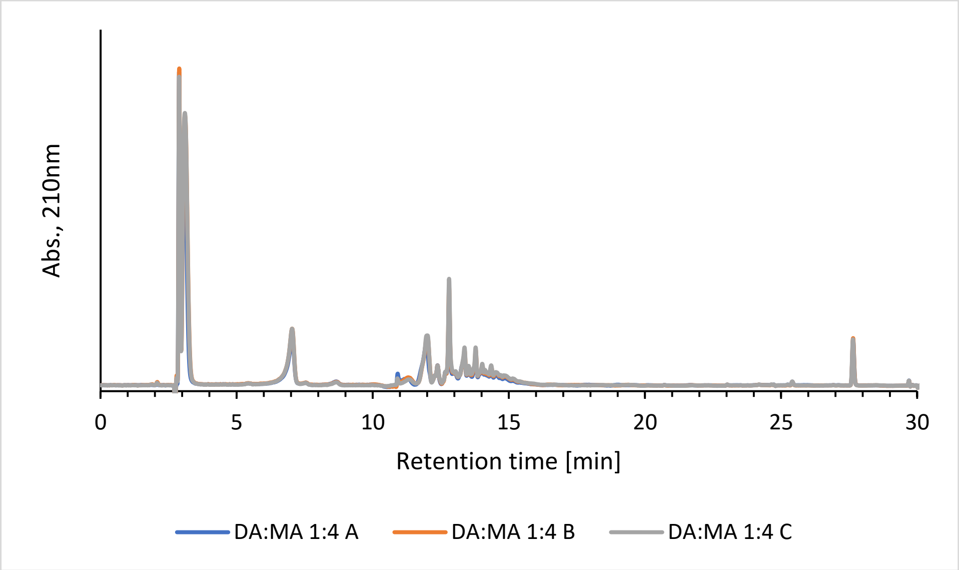

B

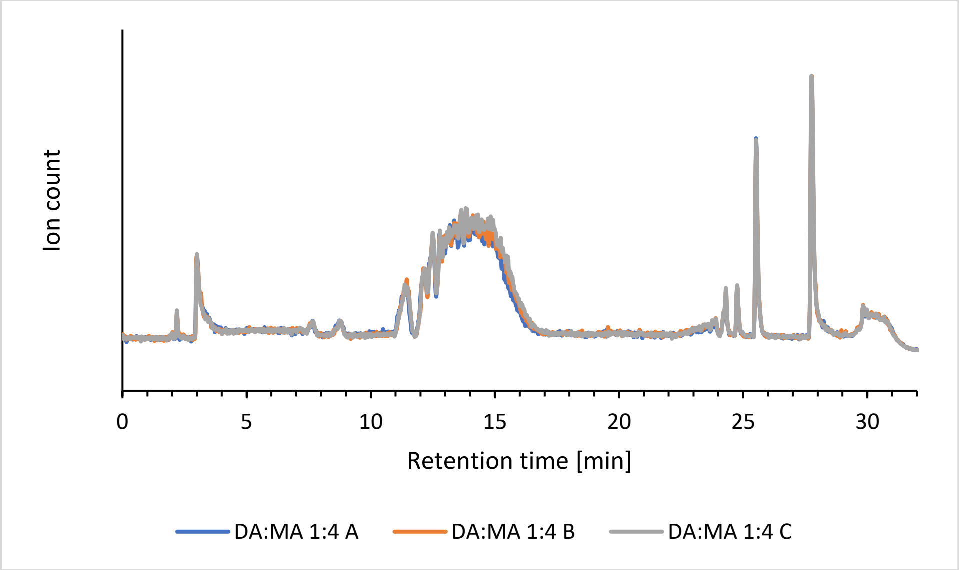

Figure S10. LC-MS analysis confirms the formation of products obtained by the reaction of DA and MA at a 1:4 molar ratio. DA and MA reacted under dry conditions at 85 °C for 7 days and the reaction products were analyzed by HPLC at 210nm (A) and LC-MS operating at negative mode (B). Chromatograms were obtained for three individual preparations.

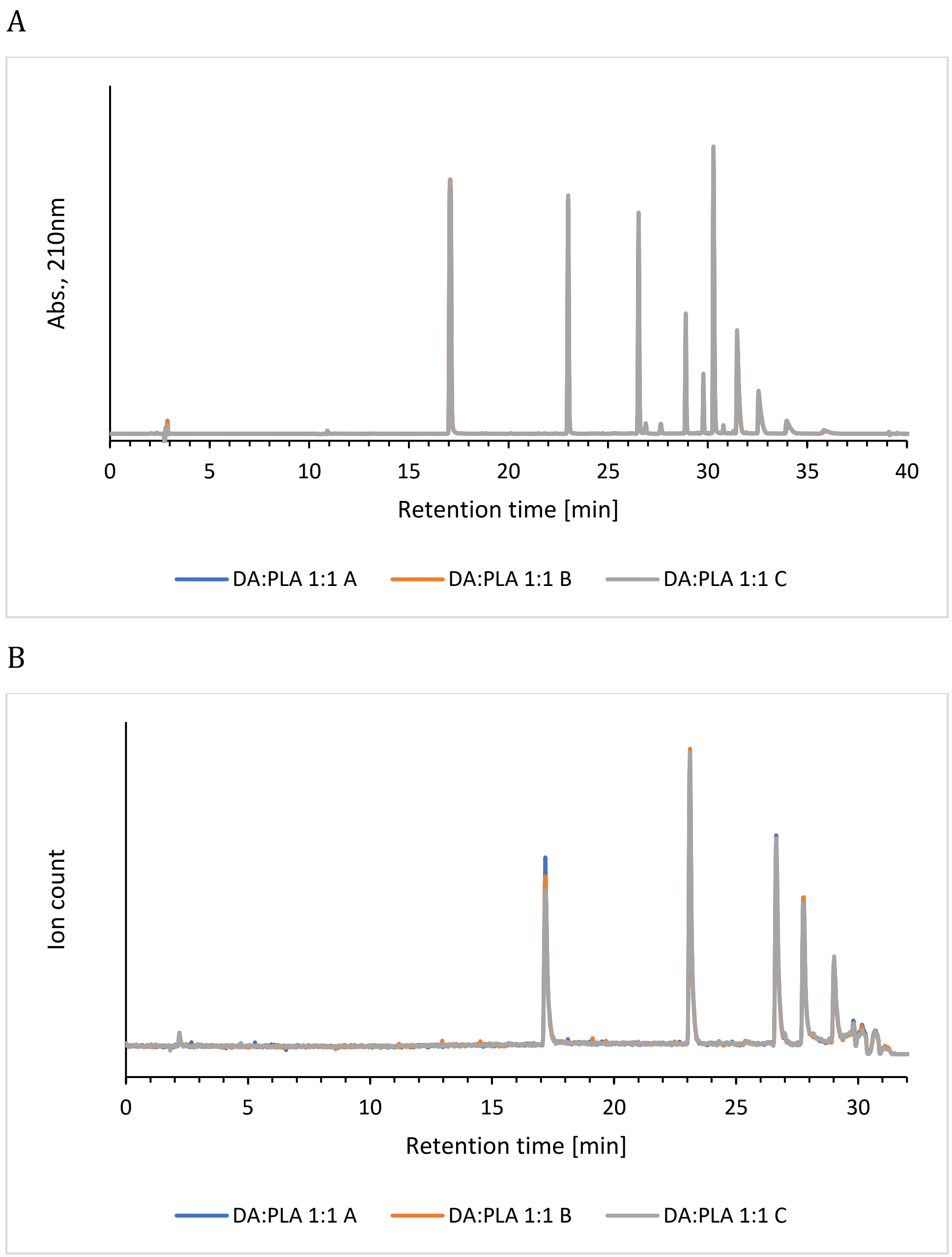

Figure S11. LC-MS analysis confirms the formation of products obtained by the reaction of DA and PLA at a 1:1 molar ratio. DA and PLA reacted under dry conditions at 85 °C for 7 days and the reaction products were analyzed by HPLC at 210nm (A) and LC-MS operating at negative mode (B). Chromatograms were obtained for three individual preparations.

A

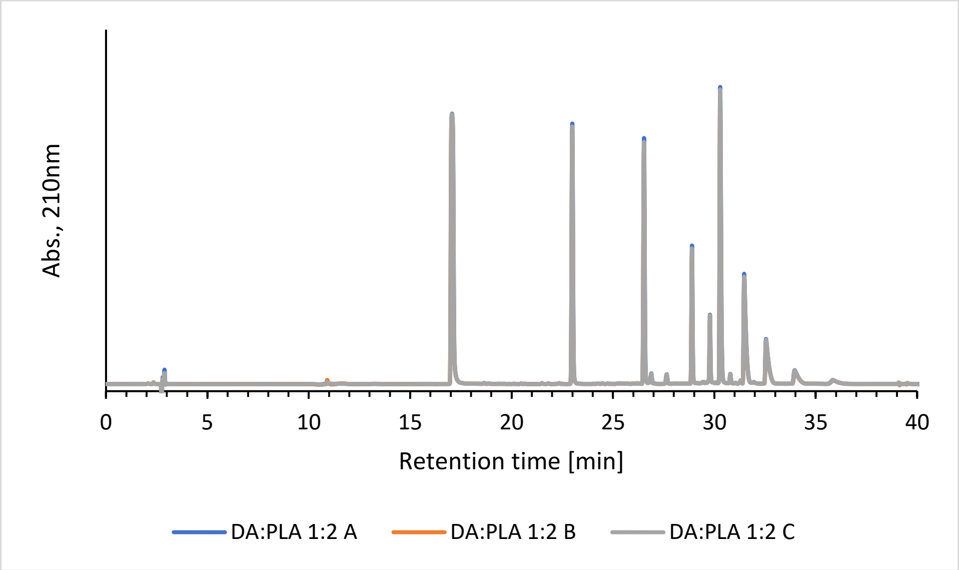

B

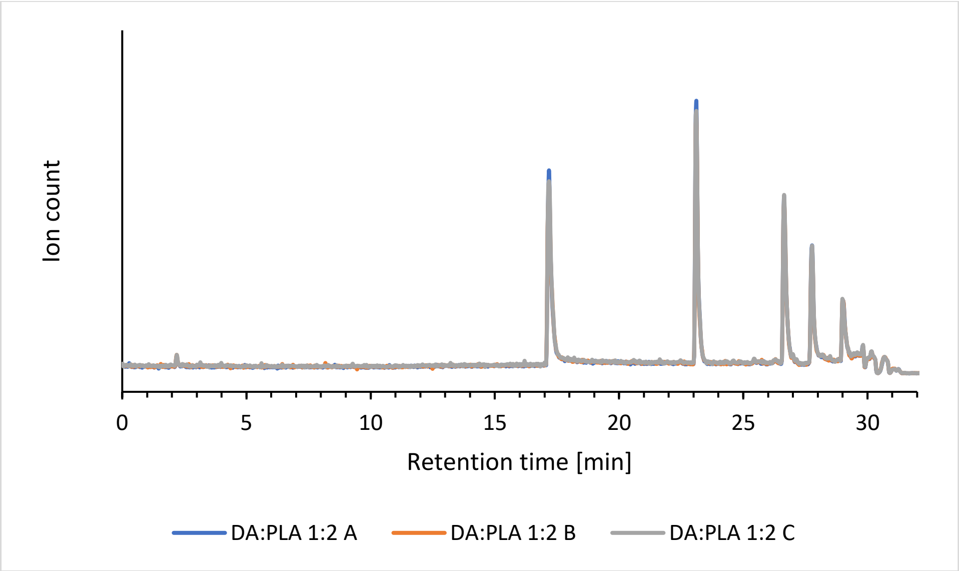

Figure S12. LC-MS analysis confirms the formation of products obtained by the reaction of DA and PLA at a 1:2 molar ratio. DA and PLA reacted under dry conditions at 85 °C for 7 days and the reaction products were analyzed by HPLC at 210nm (A) and LC-MS operating at negative mode (B). Chromatograms were obtained for three individual preparations.

A

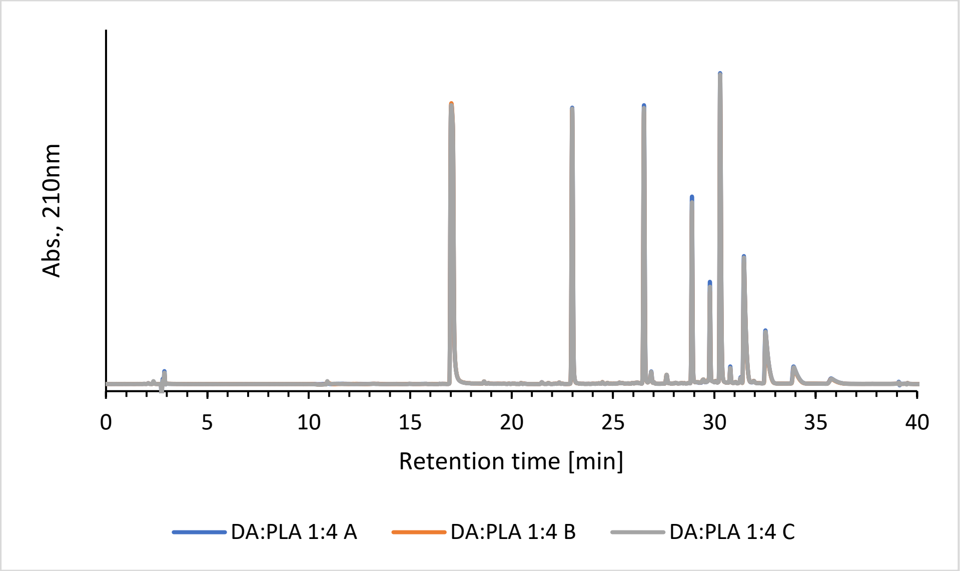

B

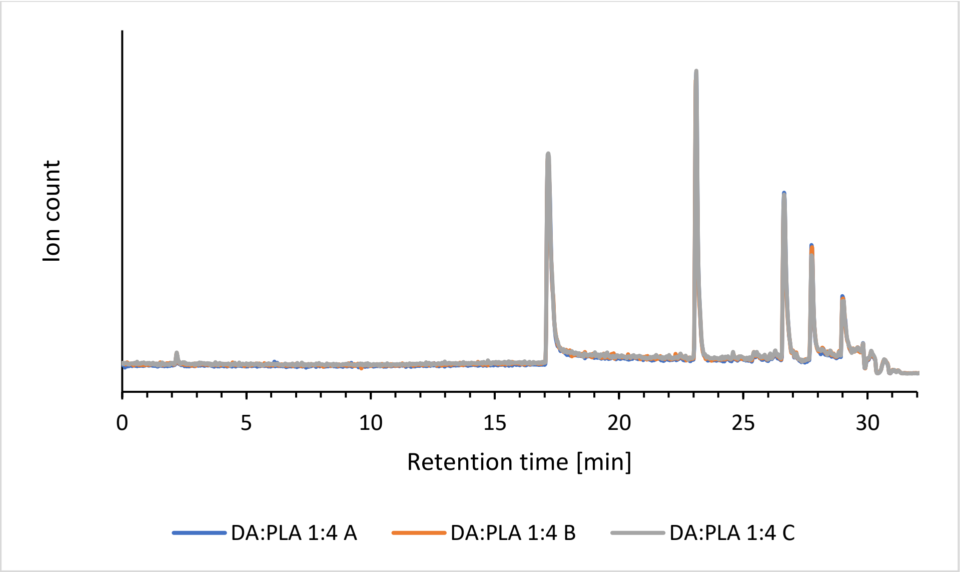

Figure S13: LC analysis confirms the formation of products obtained by the reaction of DA and PLA at a 1:4 molar ratio. DA and PLA reacted under dry conditions at 85 °C for 7 days and the reaction products were analyzed by HPLC at 210nm (A) and LC-MS operating at negative mode (B). Chromatograms were obtained for three individual preparations.

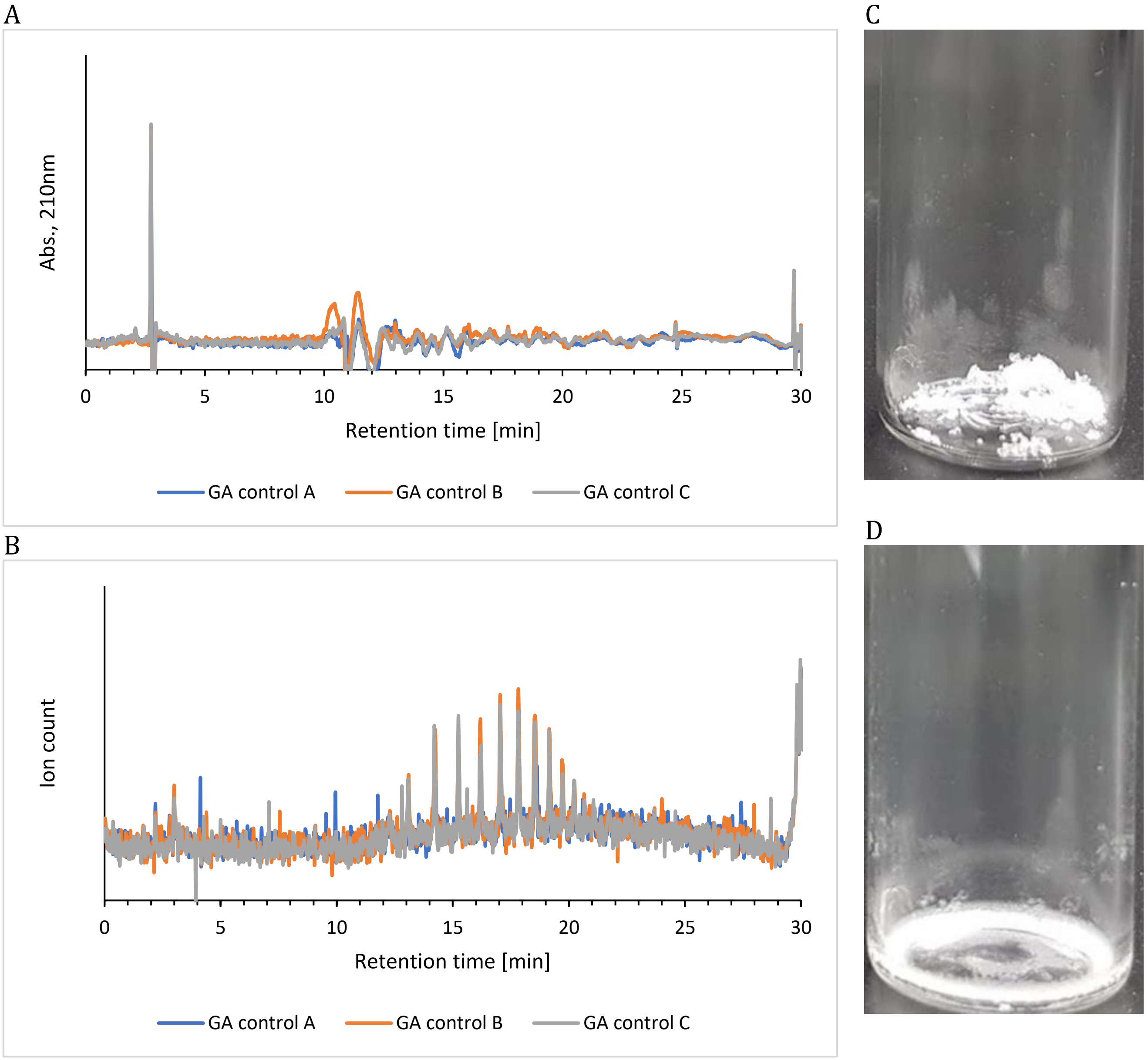

Figure S14. LC-MS analysis confirms the formation of products obtained for GA control in the absence of DA. GA (400μmol) was allowed to react under dry conditions at 85 °C for 7 days and the reaction products were analyzed by HPLC at 210nm (A) and LC-MS operating at negative mode (B). Chromatograms were obtained for three individual preparations. Images of GA sample prior to the reaction initiation (C) and after the reaction termination (D). The image of the resulting product suggests a high extent of GA oligomerization as indicated by the plastic-like appearance which was only partially soluble in the analysis diluent.

A

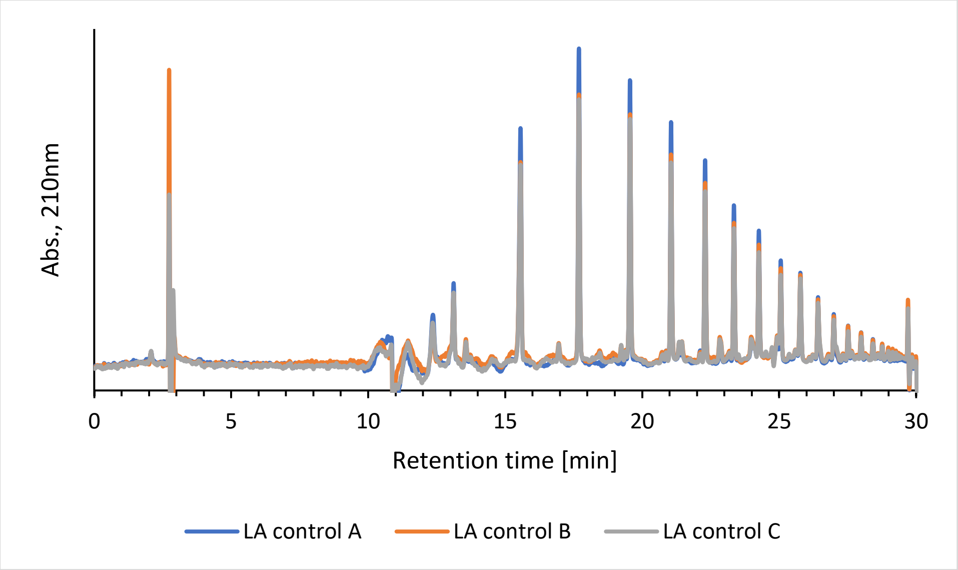

B

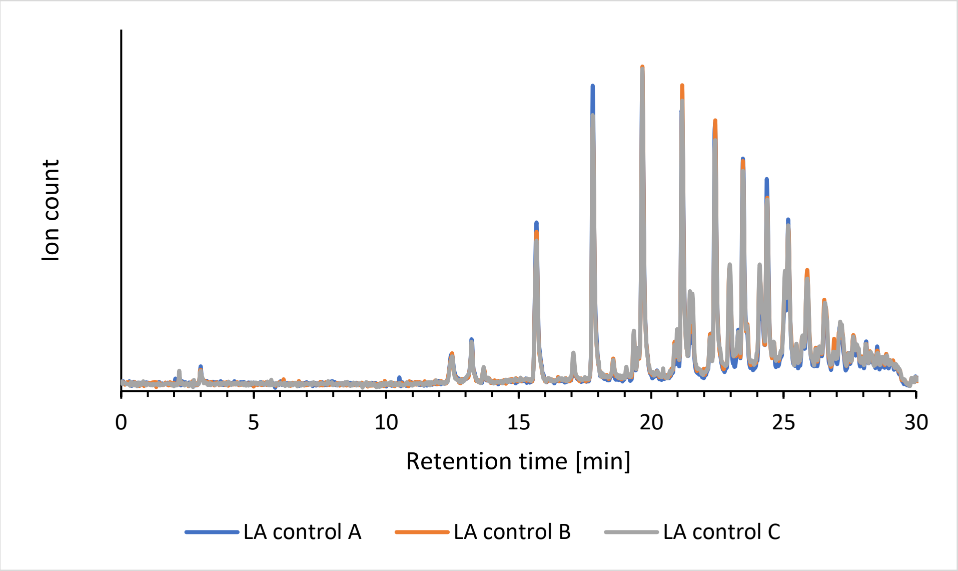

Figure S15. LC-MS analysis confirms the formation of products obtained for LA control in the absence of DA. LA (400µmol) was allowed to react under dry conditions at 85 °C for 7 days and the reaction products were analyzed by HPLC at 210nm (A) and LC-MS operating at negative mode (B). Chromatograms were obtained for three individual preparations.

A

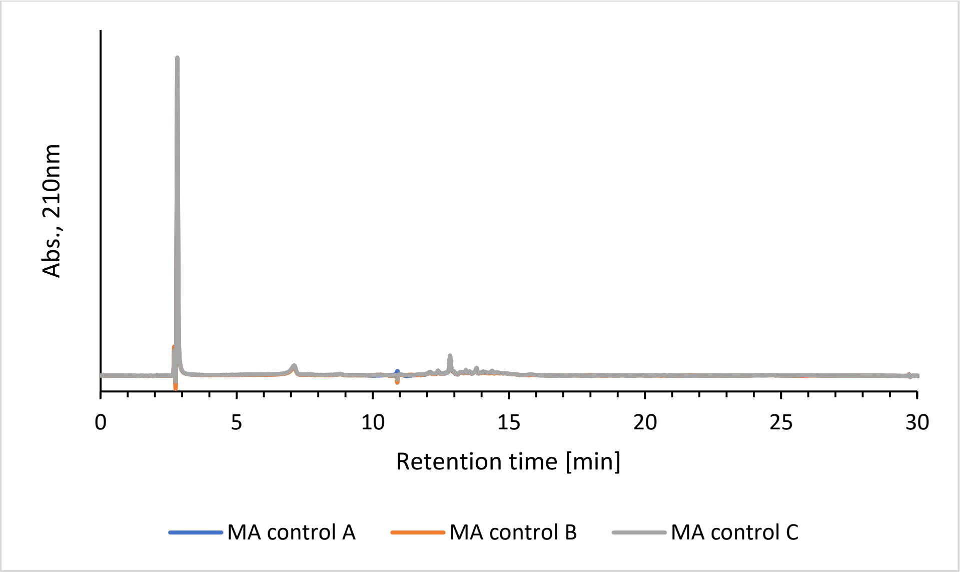

B

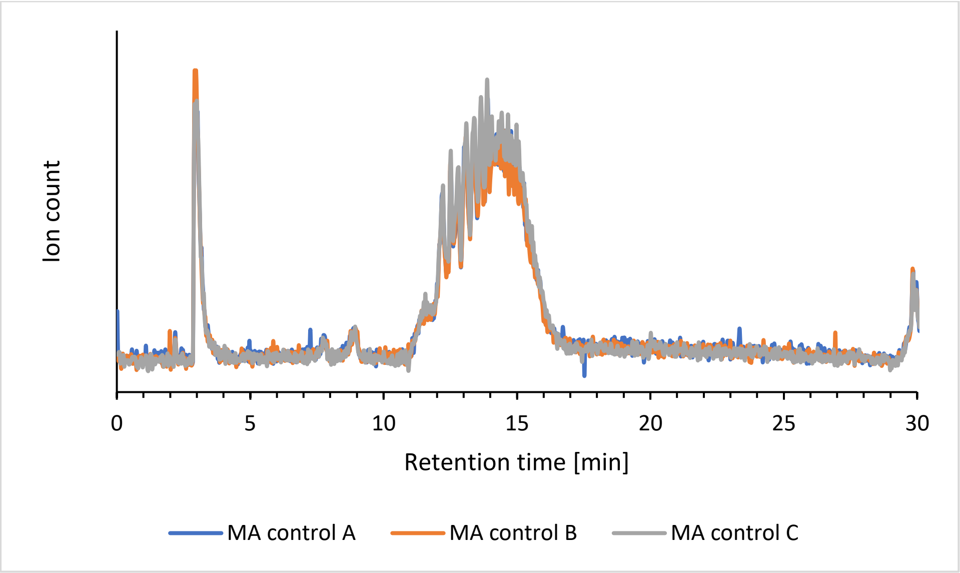

Figure S16. LC-MS analysis confirms the formation of products obtained for MA control in the absence of DA. MA (400μmol) was allowed to react under dry conditions at 85 °C for 7 days and the reaction products were analyzed by HPLC at 210nm (A) and LC-MS operating at negative mode (B). Chromatograms were obtained for three individual preparations.

A

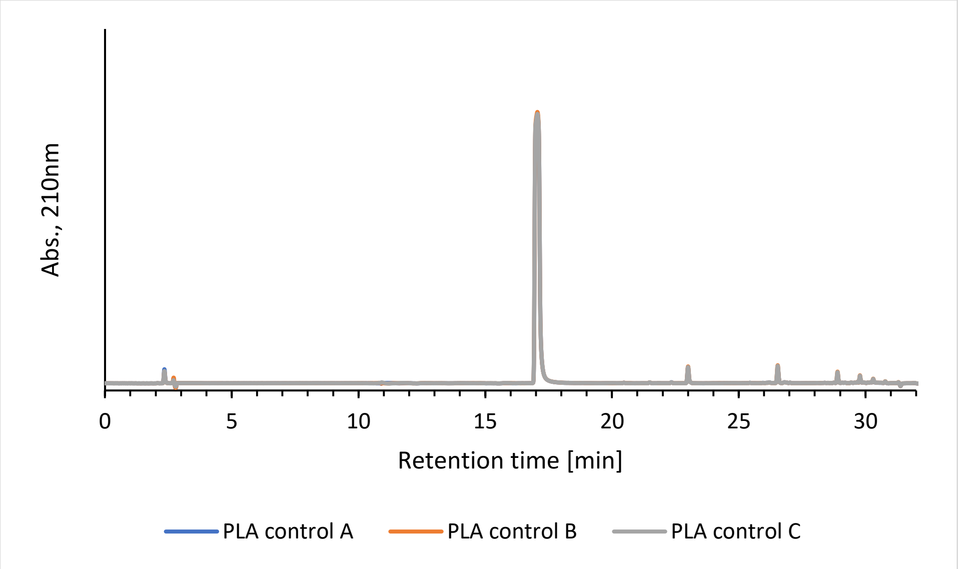

B

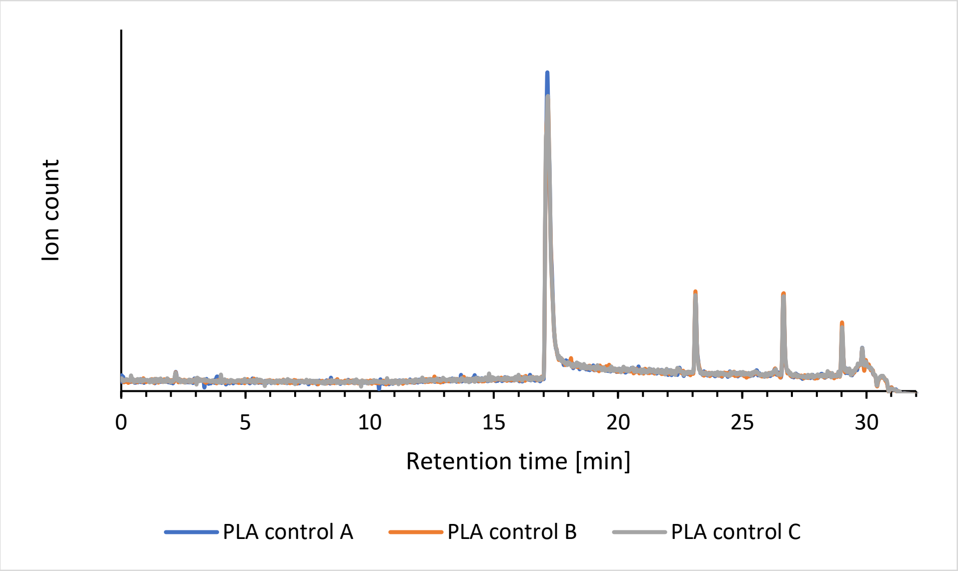

Figure S17. LC-MS analysis confirms the formation of products obtained for PLA control in the absence of DA. PLA (400µmol) was allowed to react under dry conditions at 85 °C for 7 days and the reaction products were analyzed by HPLC at 210nm (A) and LC-MS operating at negative mode (B). Chromatograms were obtained for three individual preparations.

A

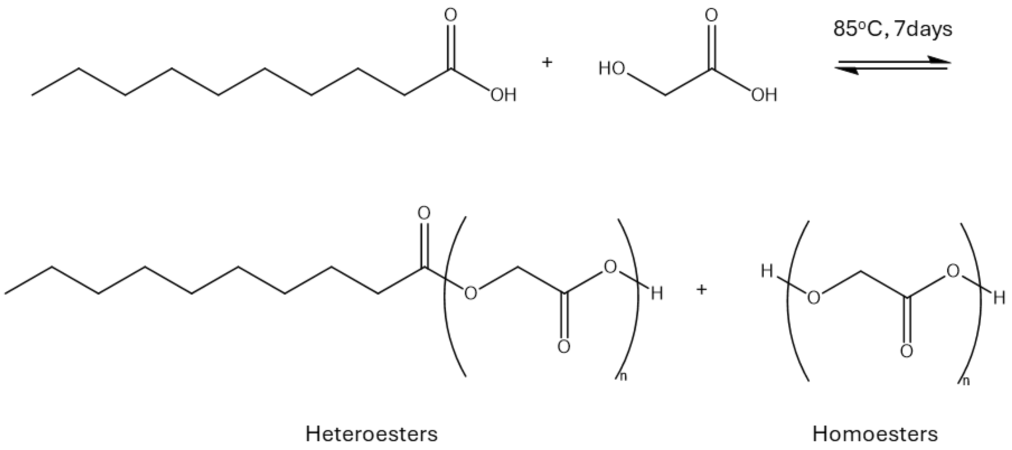

B

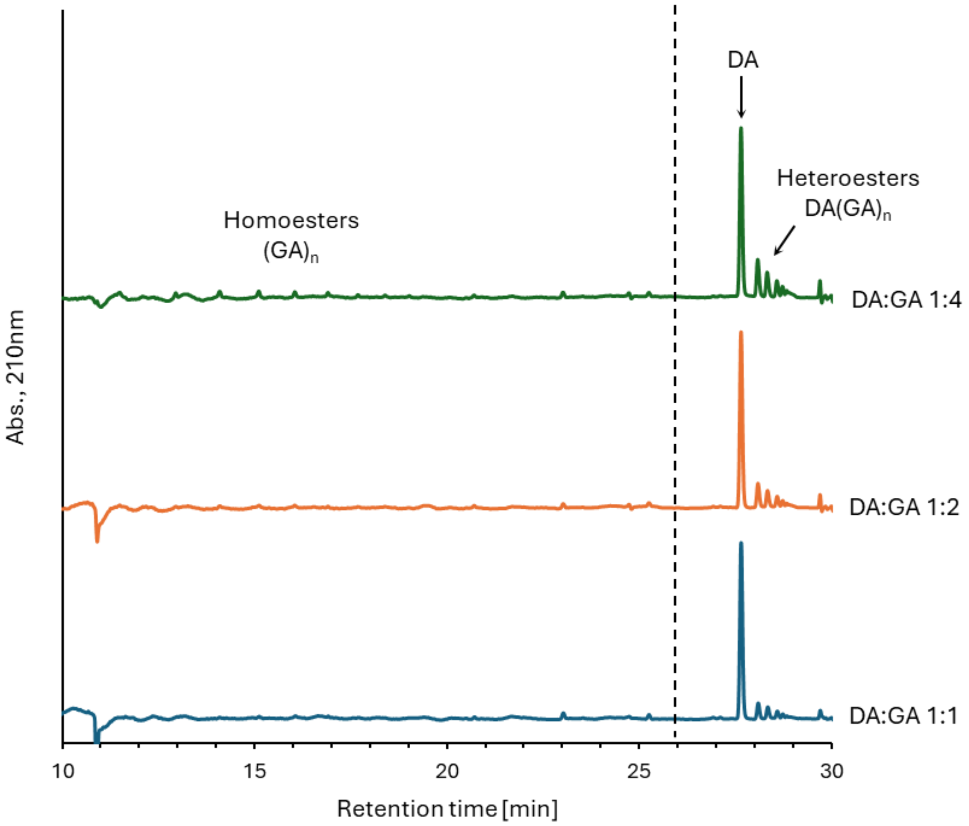

Figure S18. DA and GA react to produce two types of products: homoesters of GA and heteroesters of DA and GA. General esterification reaction that DA and GA undergo at 85 °C for 7 days. The main products obtained by the reaction are homooligomers of GA and cooligomers of single DA molecule covalently linked to GA or GA oligomers (A). HPLC chromatogram of the products obtained by the reaction of DA and GA at 1:1, 1:2, and 1:4 molar ratio (B). The products eluted at earlier retention times correspond to GA homooligomers while products of later retention times correspond to DA-GA cooligomers. A greater excess of GA results in the formation of more products at higher levels.

A

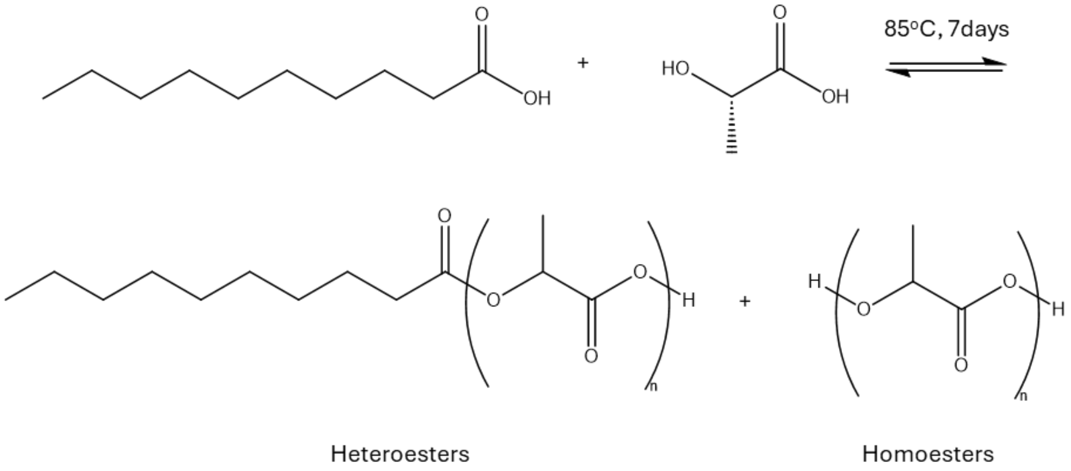

B

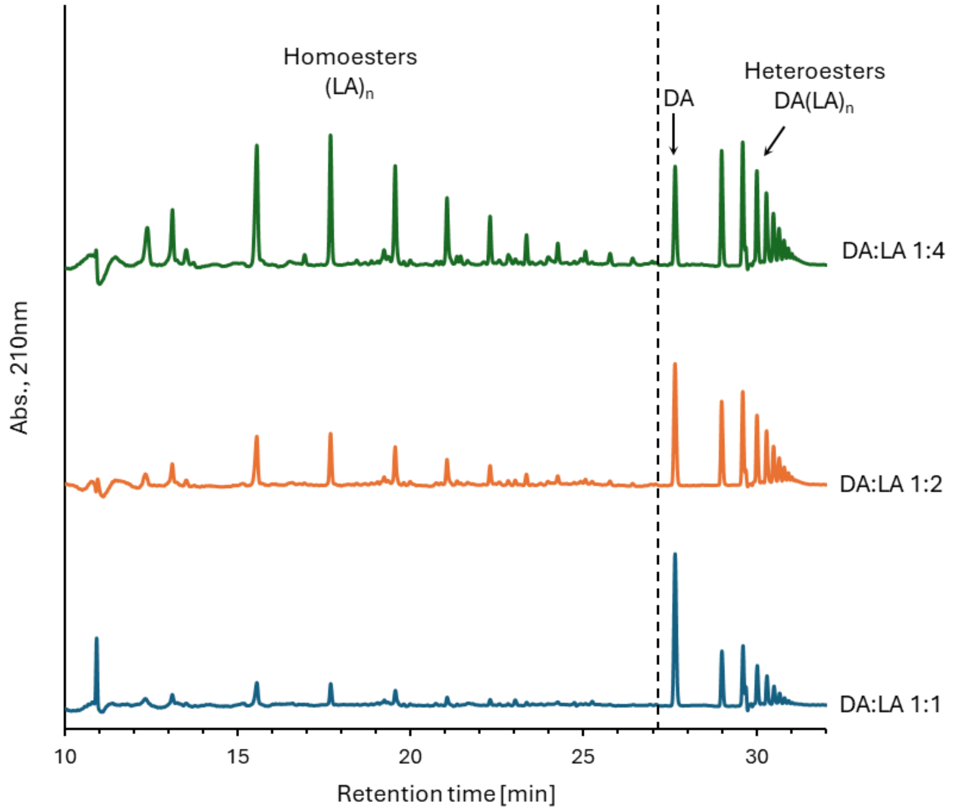

Figure S19. DA and LA react to produce two types of products: homoesters of LA and heteroesters of DA and LA. General esterification reaction that DA and LA undergo at 85 °C for 7 days. The main products obtained by the reaction are homooligomers of LA and cooligomers of single DA molecule covalently linked to LA or LA oligomers (A). HPLC chromatogram of the products obtained by the reaction of DA and LA at 1:1, 1:2, and 1:4 molar ratio (B). The products eluted at earlier retention times correspond to LA homooligomers while products of later retention times correspond to DA-LA cooligomers. A greater excess of LA results in the formation of more products at higher levels.

A

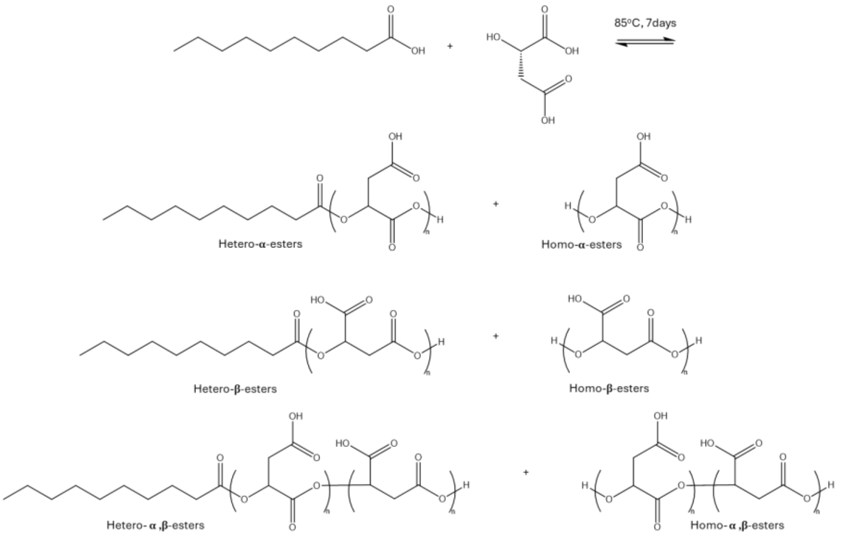

B

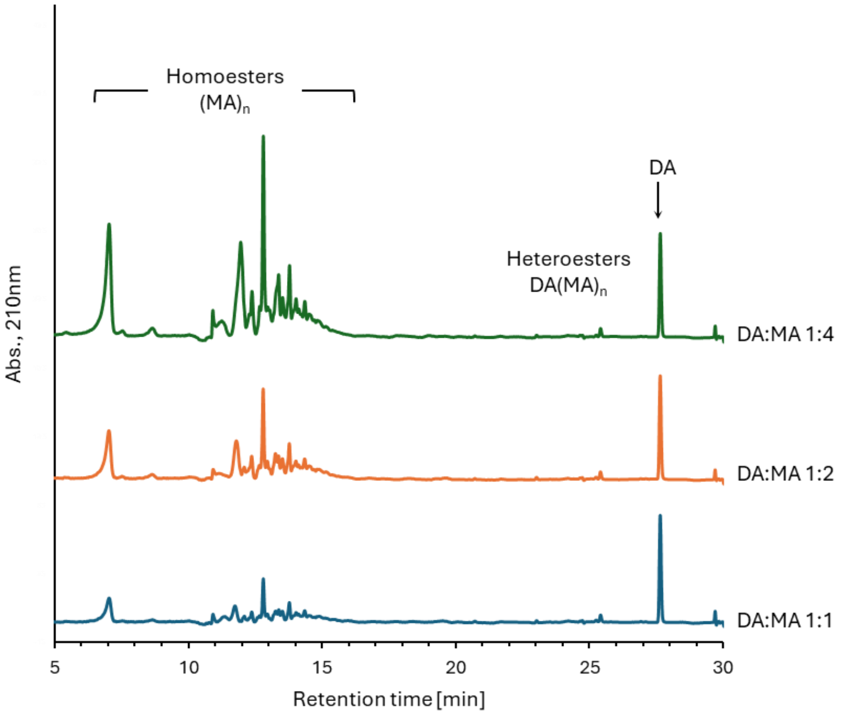

Figure S20. DA and MA react to produce two types of products: homoesters of MA and heteroesters of DA and MA. General esterification reaction that DA and LA undergo at 85 °C for 7 days. The main products obtained by the reaction are homooligomers and cooligomers of either the α-esters, β-esters, or α,β-esters. α and β esters refer to the esterification products at the α- and β- carboxylic acid of MA, respectively (A). HPLC chromatogram of the products obtained by the reaction of DA and MA at 1:1, 1:2, and 1:4 molar ratio (B). The products eluted at earlier retention times correspond to MA homooligomers. The products eluted within 20-26min correspond to DA-MA cooligomers. A greater excess of MA results in the formation of more products at higher levels.

A

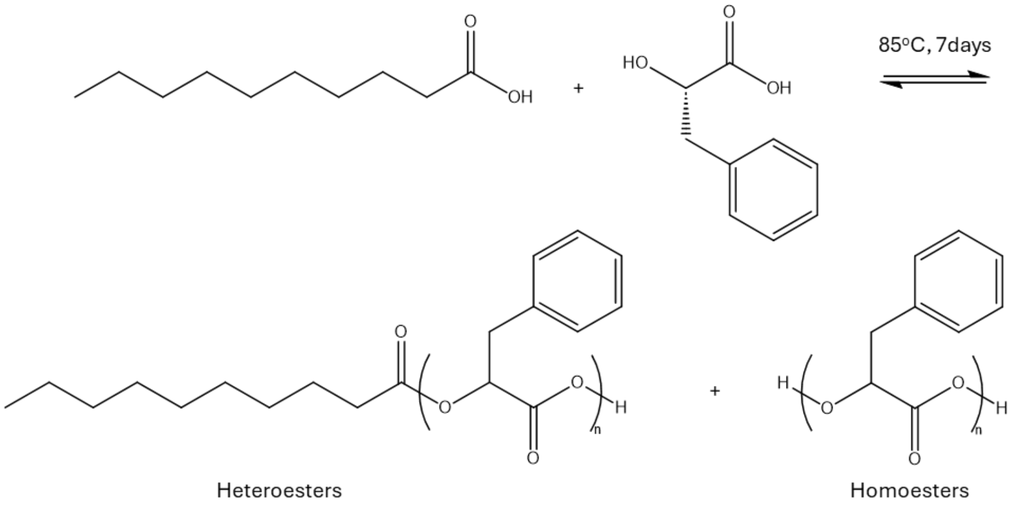

B

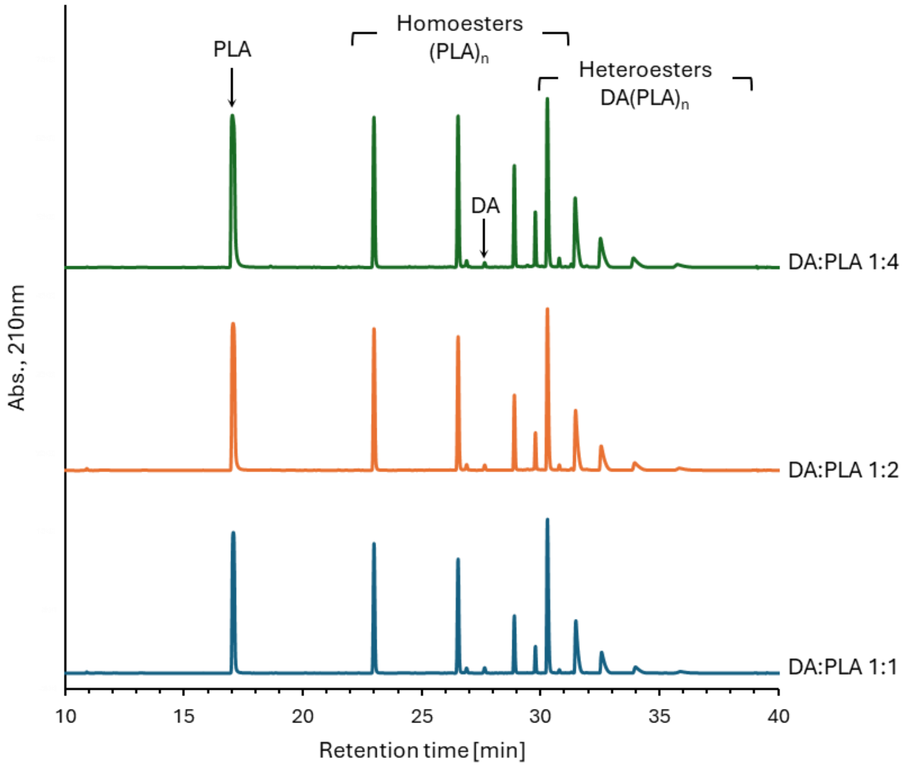

Figure S21. DA and PLA react to produce two types of products: homoesters of PLA and heteroesters of DA and PLA. General esterification reaction that DA and PLA undergo at 85 °C for 7 days. The main products obtained by the reaction are homooligomers of PLA and cooligomers of single DA molecule covalently linked to PLA or LA oligomers (A). HPLC chromatogram of the products obtained by the reaction of DA and PLA at 1:1, 1:2, and 1:4 molar ratio (B). Increasing the levels of PLA did not result in the formation of new products, but increased their concentration.

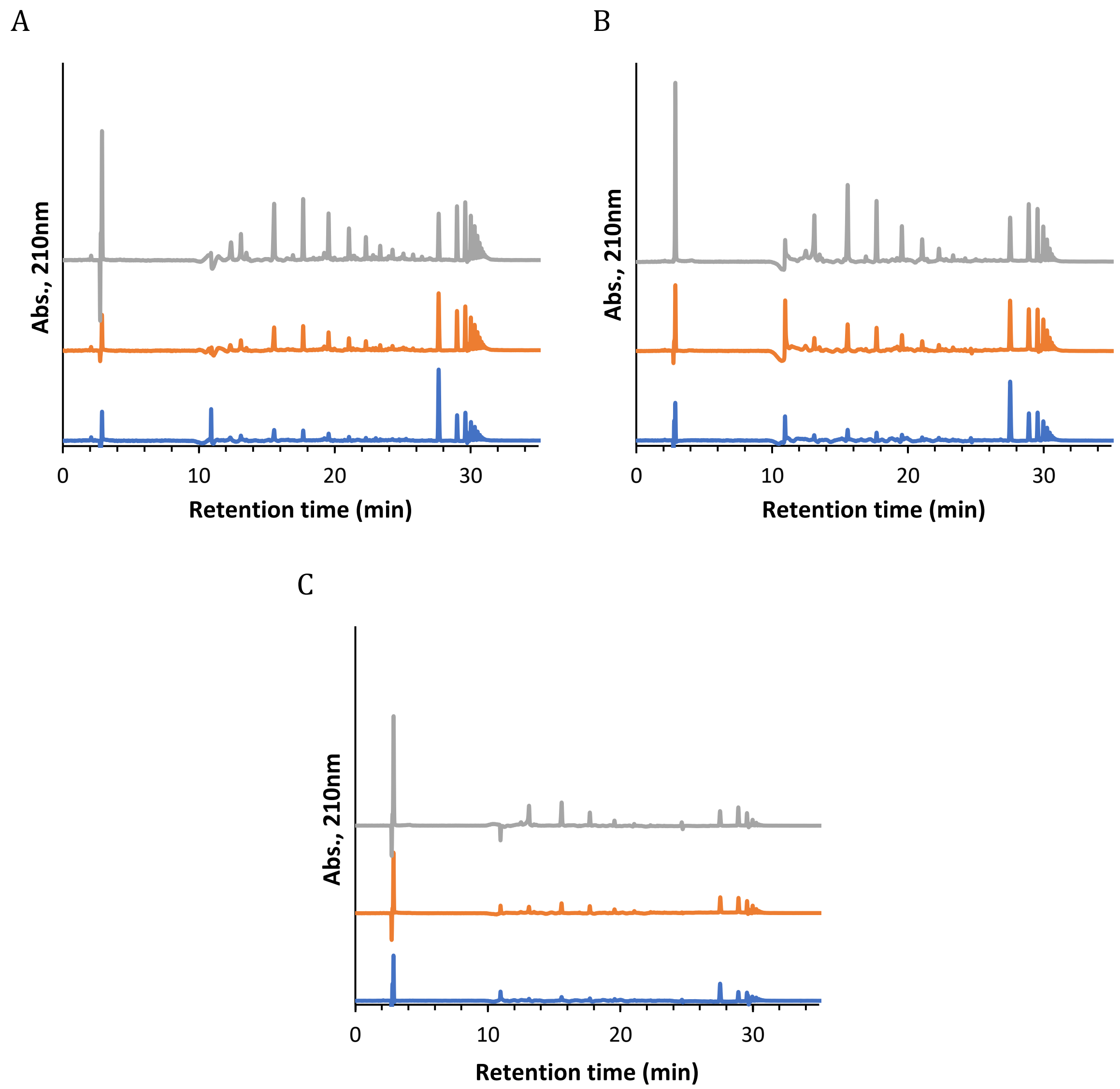

Figure S22. Batch size affects product formation and the concentration of products obtained by the dry-state reaction of DA and LA. HPLC chromatograms obtained for DA-LA reaction products at various batch sizes, including 200 µmol DA (A), 400 µmol DA (B), and 800 µmol DA (C). DA:LA molar ratios of 1:1 (blue lines), 1:2 (orange lines), and 1:4 (grey lines) were examined. As indicated by the figure, as the batch size (i.e., DA mass) increased, the variety of products decreased due to limitations in mass transfer. When the batch size increases, adjustments (such as applying mechanical forces and adjustments of reaction vessels) must be considered in order to compensate for the greater inhomogeneity resulting from this increase.

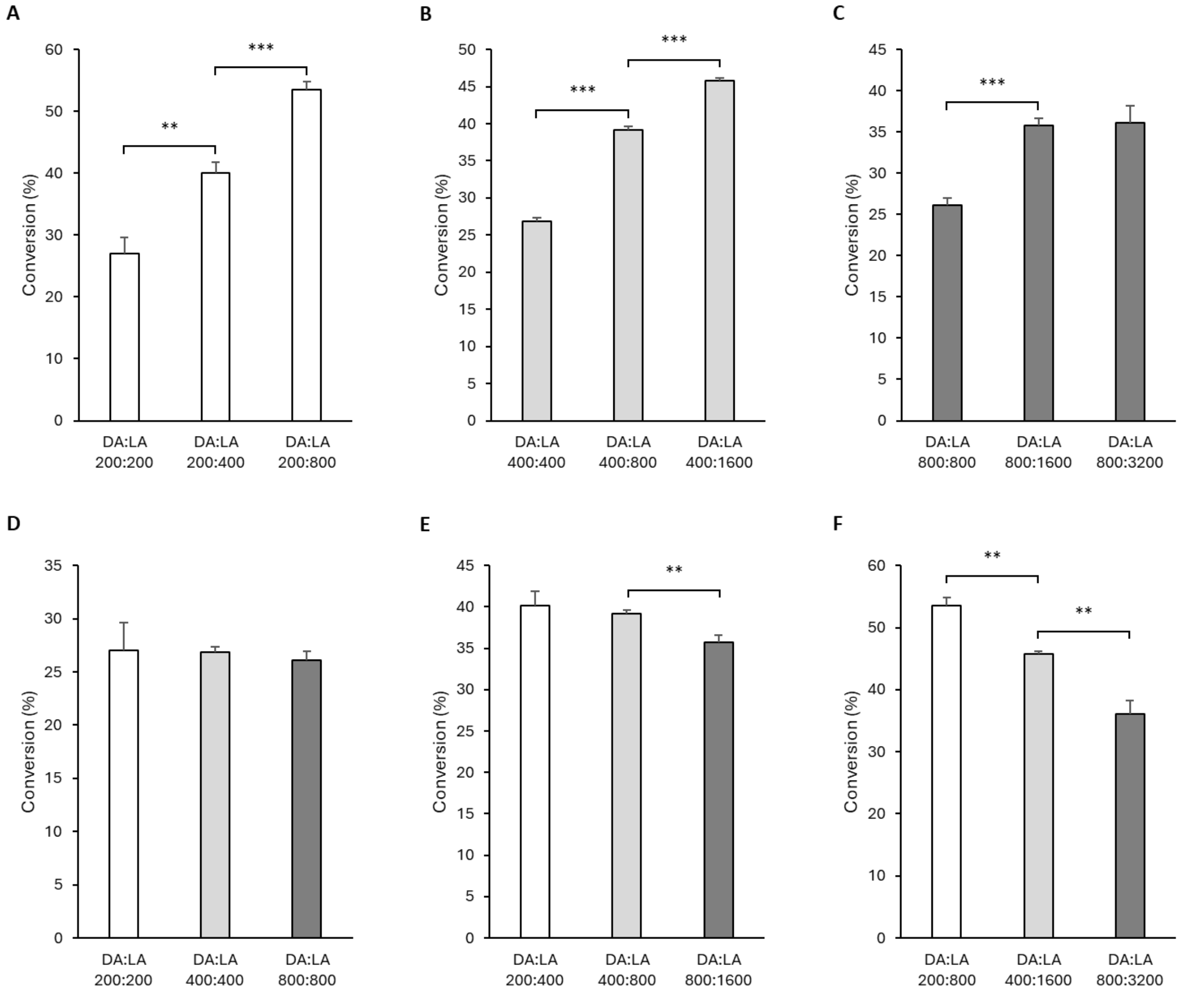

Figure S23. DA conversion obtained for different batch sizes and different molar ratios. The conversion of DA obtained for the reaction of 200 µmol DA with either 200, 400 or 800 µmol LA (A), the conversion of DA obtained for the reaction of 400 µmol DA with either 400, 800 or 1600 µmol LA (B), the conversion of DA obtained for the reaction of 800 µmol DA with either 800, 1600 or 3200 µmol LA (C), the conversion of DA obtained for constant DA:LA 1:1 molar ratio and either 200, 400 or 800 µmol DA (D), the conversion of DA obtained for constant DA:LA 1:2 molar ratio and either 200, 400 or 800 µmol DA (E), and the conversion of DA obtained for constant DA:LA 1:4 molar ratio and either 200, 400 or 800 µmol DA (F). As indicated in panels A-C, increasing batch size does not affect the trend where a greater excess of LA results in an increase of DA conversion. There is only one exception for DA:LA at 800:3200 (µmol:µmol), for which DA conversion was comparable to the conversion at DA:LA 800:1600 (µmol:µmol). Panel D-F shows the effect of increasing batch size on the obtained DA conversion. For DA:LA 1:1 molar ratio, increasing the batch size has no effect on the conversion. At DA:LA 1:2 molar ratio, only a slight reduction in DA conversion was observed for 800 µmol DA. The greatest effect of batch size was observed at a DA:LA 1:4 molar ratio, for which a gradual decrease in DA conversion was observed (55% , 45%, and 35% conversion for 200, 400, and 800 µmol DA, respectively). Error bars represent standard deviation of three independent preparations.

Table S1. Identification of DA-GA reaction products. The detected products based on retention time and their corresponding m/z and ionization pattern as determined by LC-MS.

Retention time (min)	Compound	M (g/mol)	Corresponding m/z (-TIC)	Ionization pattern
**11.6**	3GA	192.1	191.1, 383.2	$[M-H]^-$, $[2M-H]^-$
**12.2**	4GA	250.1	249.0, 499.3	$[M-H]^-$, $[2M-H]^-$
**13.1**	4GA	250.1	249.0, 499.2	$[M-H]^-$, $[2M-H]^-$
**14.2**	5GA	308.2	307.0, 615.3	$[M-H]^-$, $[2M-H]^-$
**15.2**	6GA	366.2	365.2, 731.2	$[M-H]^-$, $[2M-H]^-$
**16.2**	7GA	424.2	423.2, 847.4	$[M-H]^-$, $[2M-H]^-$
**17.0**	8GA	482.3	481.3, 963.4	$[M-H]^-$, $[2M-H]^-$
**17.8**	9GA	540.3	539.2, 1079.4	$[M-H]^-$, $[2M-H]^-$
**18.5**	10GA	598.3	597.3, 1195.5	$[M-H]^-$, $[2M-H]^-$
**19.1**	11GA	656.4	655.1, 1311.7	$[M-H]^-$, $[2M-H]^-$
**19.7**	12GA	714.4	713.3	$[M-H]^-$
**28.2**	1GA1DA	230.3	229.1, 459.4	$[M-H]^-$, $[2M-H]^-$
**28.4**	2GA1DA	288.3	287.2, 575.5	$[M-H]^-$, $[2M-H]^-$
**28.7**	3GA1DA	346.4	345.3, 691.5	$[M-H]^-$, $[2M-H]^-$
**28.8**	4GA1DA	404.4	403.3, 807.5	$[M-H]^-$, $[2M-H]^-$
**28.9**	5GA1DA	462.4	461.4, 923.5	$[M-H]^-$, $[2M-H]^-$
**29.0**	6GA1DA	520.4	519.4, 1039.6	$[M-H]^-$, $[2M-H]^-$

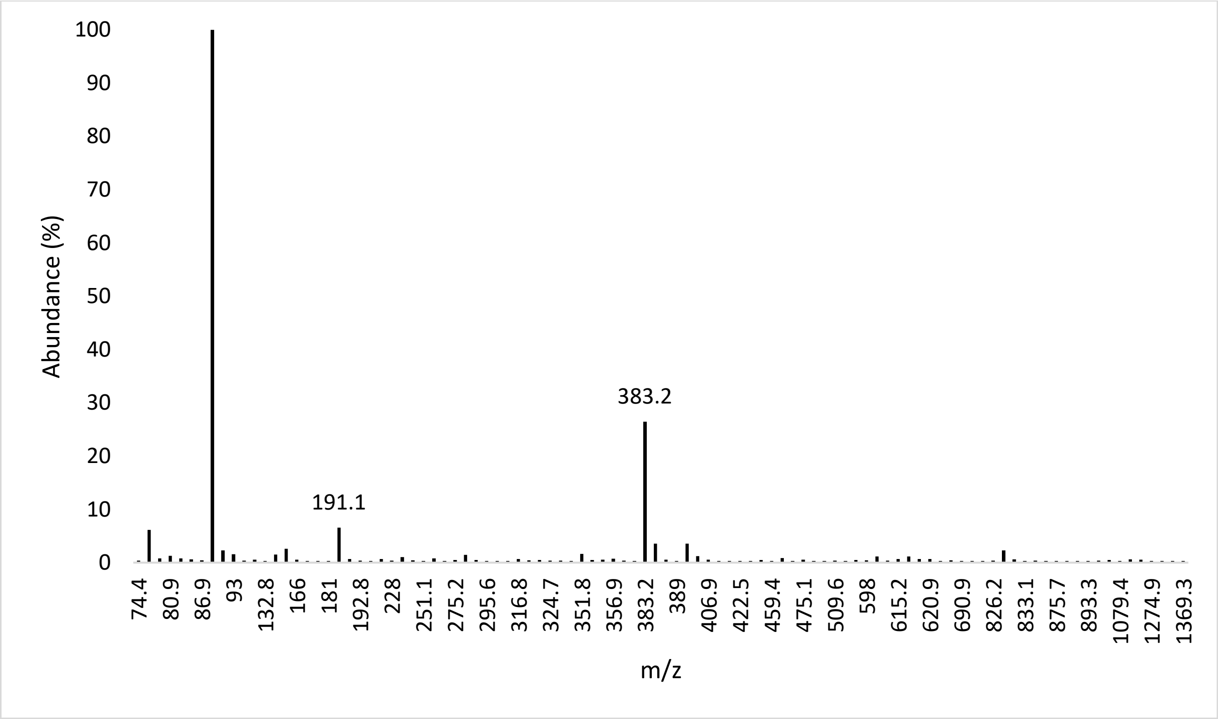

Figure S24. MS spectrum extracted from DA-GA reaction product chromatogram at retention time 11.6 min. The labeled m/z signals represent 3GA.

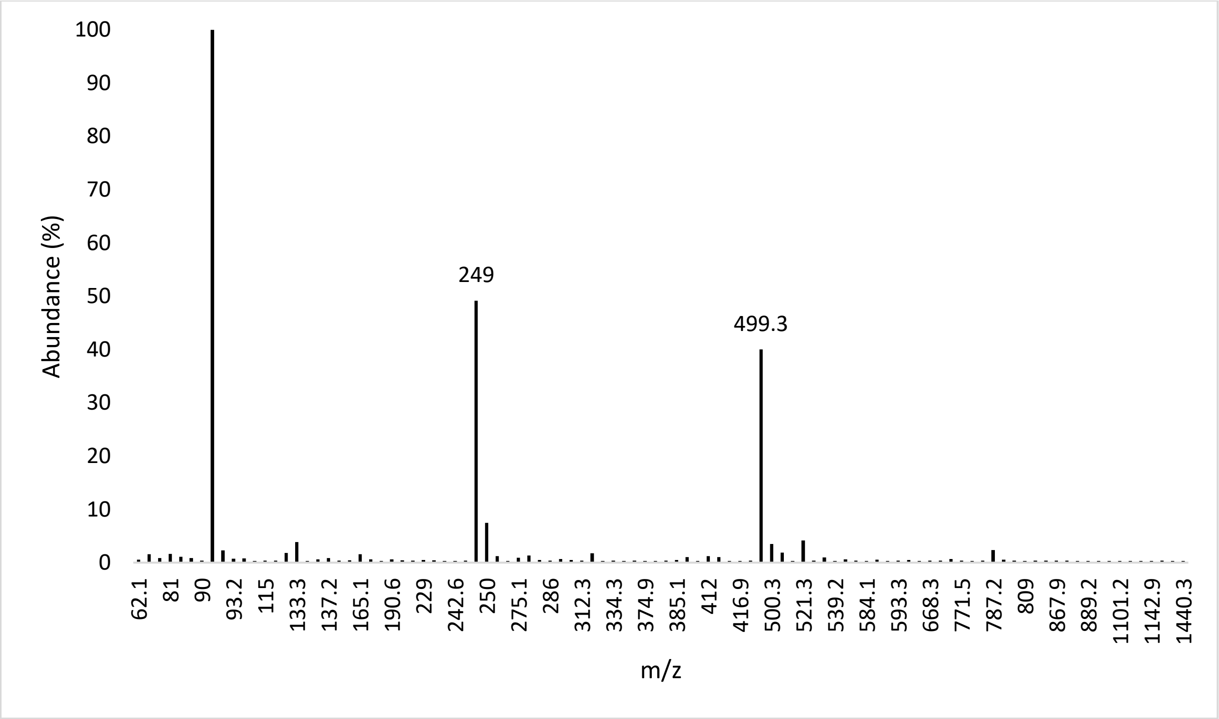

Figure S25. MS spectrum extracted from DA-GA reaction product chromatogram at retention time 12.2 min. The labeled m/z signals represent 4GA.

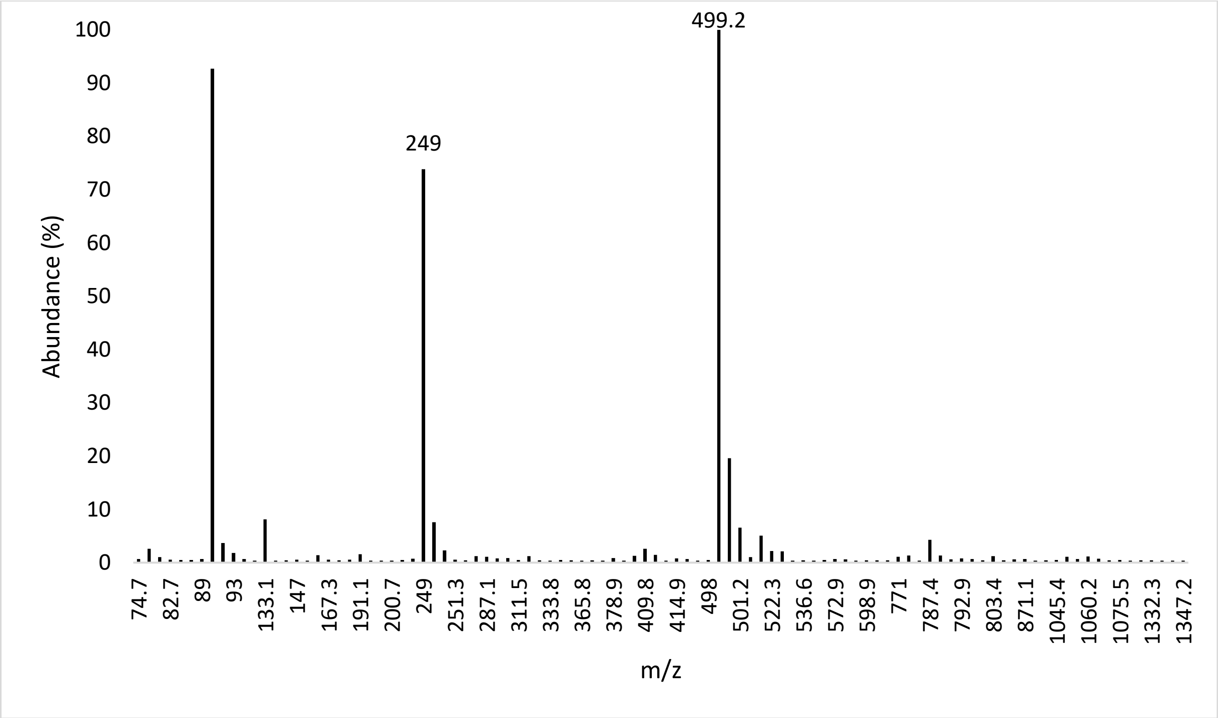

Figure S26. MS spectrum extracted from DA-GA reaction product chromatogram at retention time 13.1 min. The labeled m/z signals represent 4GA.

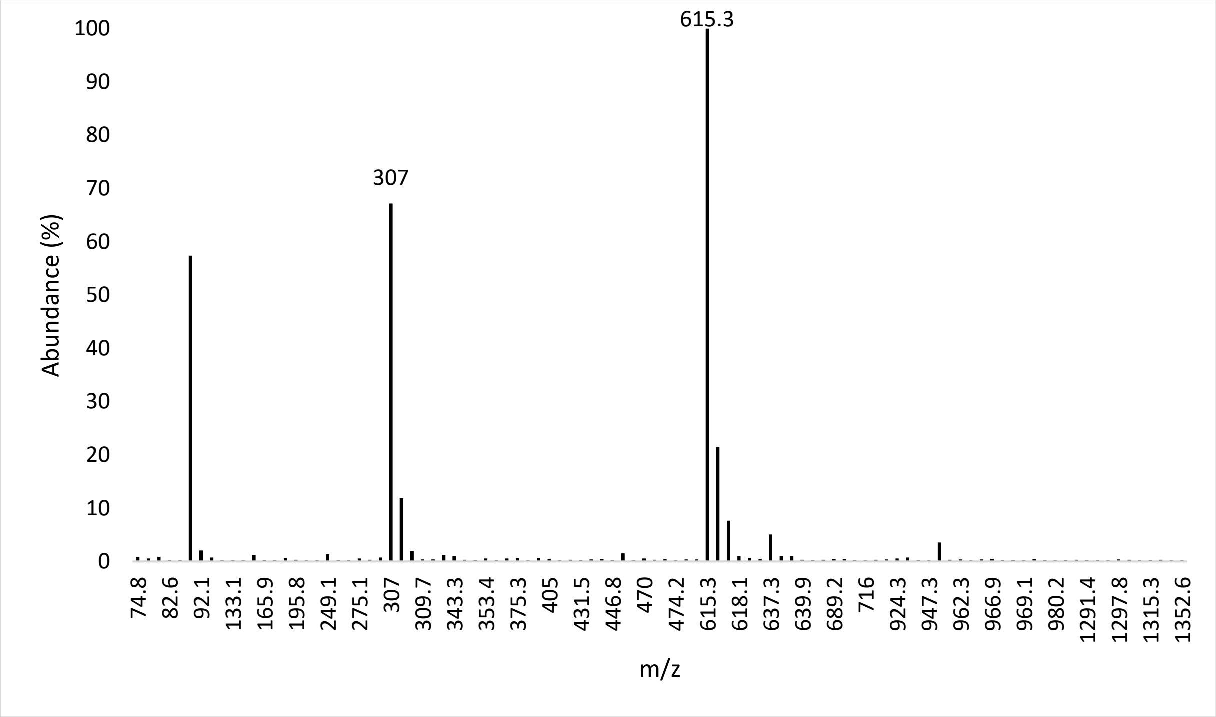

Figure S27. MS spectrum extracted from DA-GA reaction product chromatogram at retention time 14.2 min. The labeled m/z signals represent 5GA.

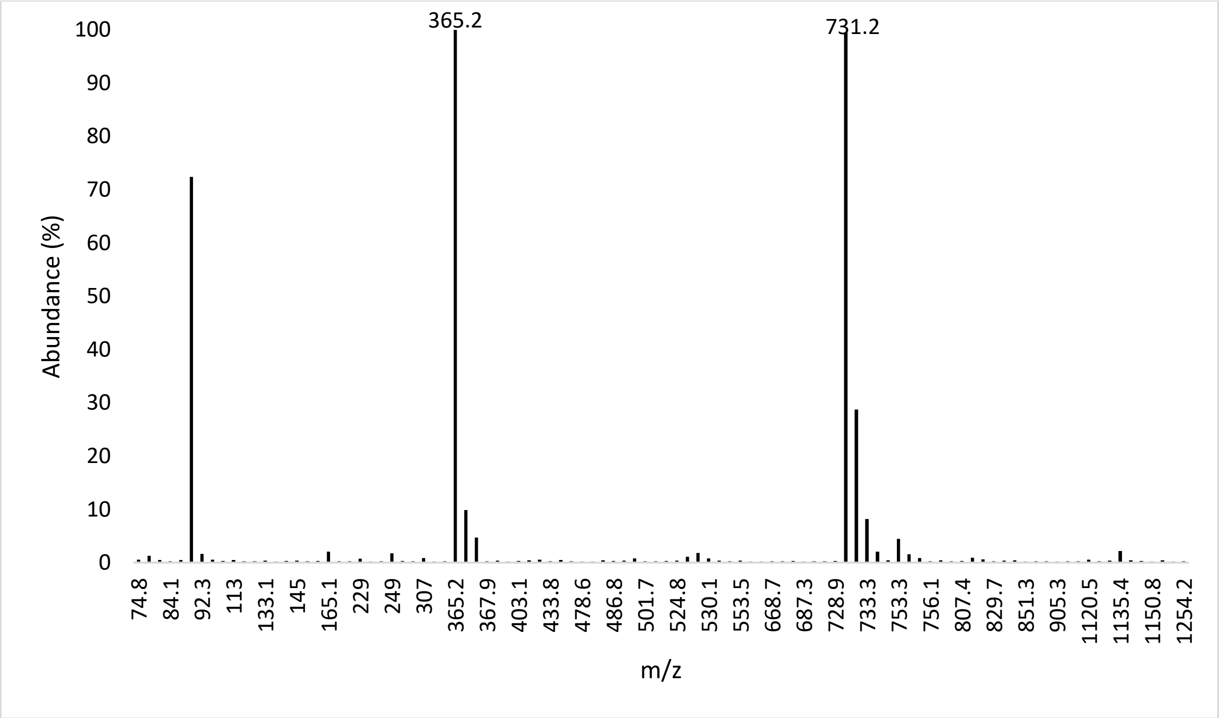

Figure S28. MS spectrum extracted from DA-GA reaction product chromatogram at retention time15.2 min. The labeled m/z signals represent 6GA.

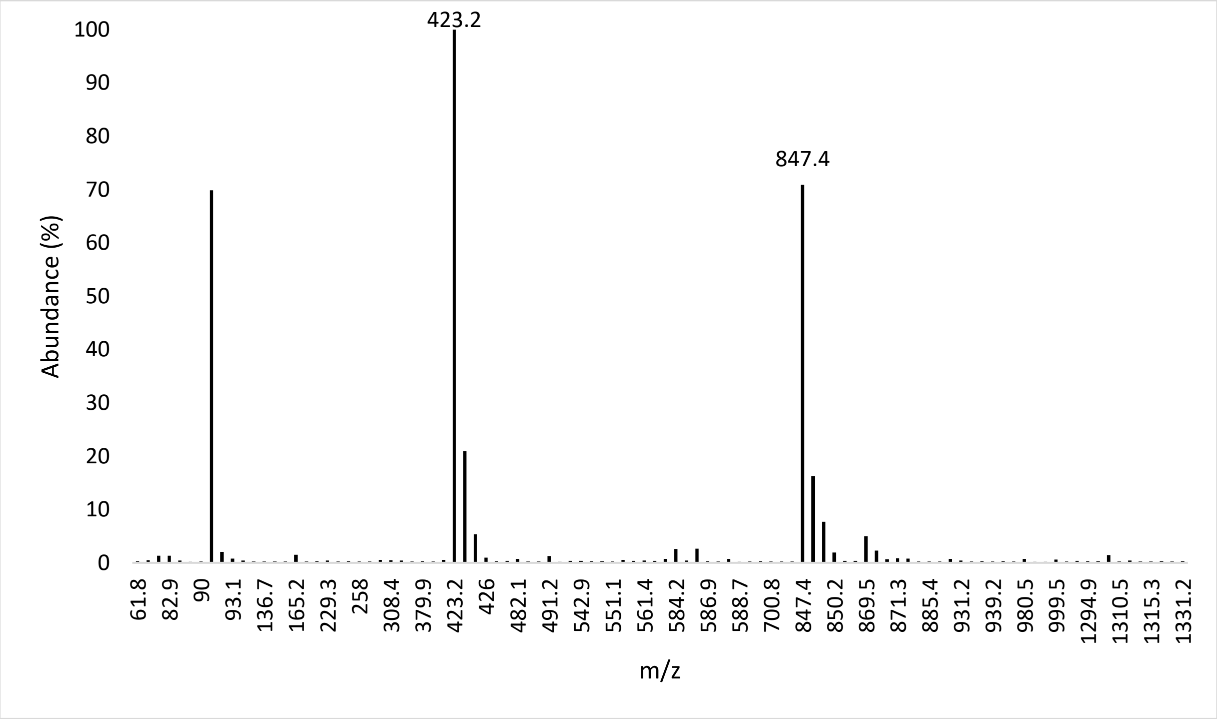

Figure S29. MS spectrum extracted from DA-GA reaction product chromatogram at retention time 16.2 min. The labeled m/z signals represent 7GA.

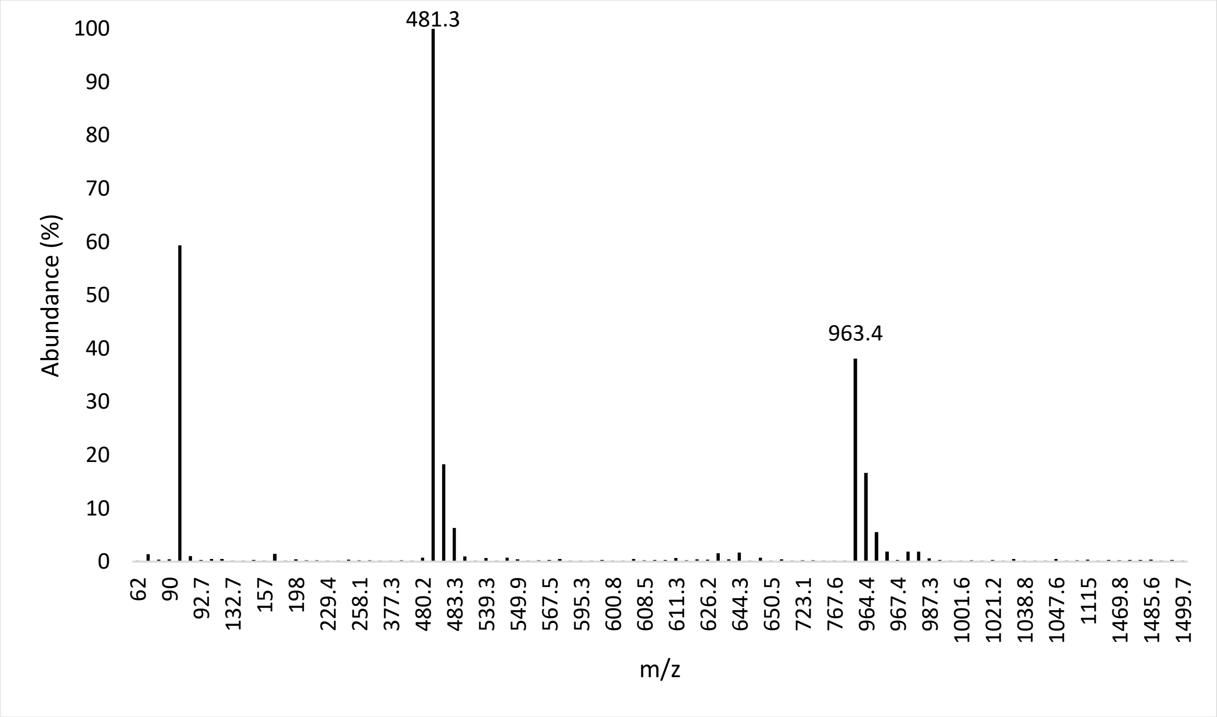

Figure S30. MS spectrum extracted from DA-GA reaction product chromatogram at retention time 17.0 min. The labeled m/z signals represent 8GA.

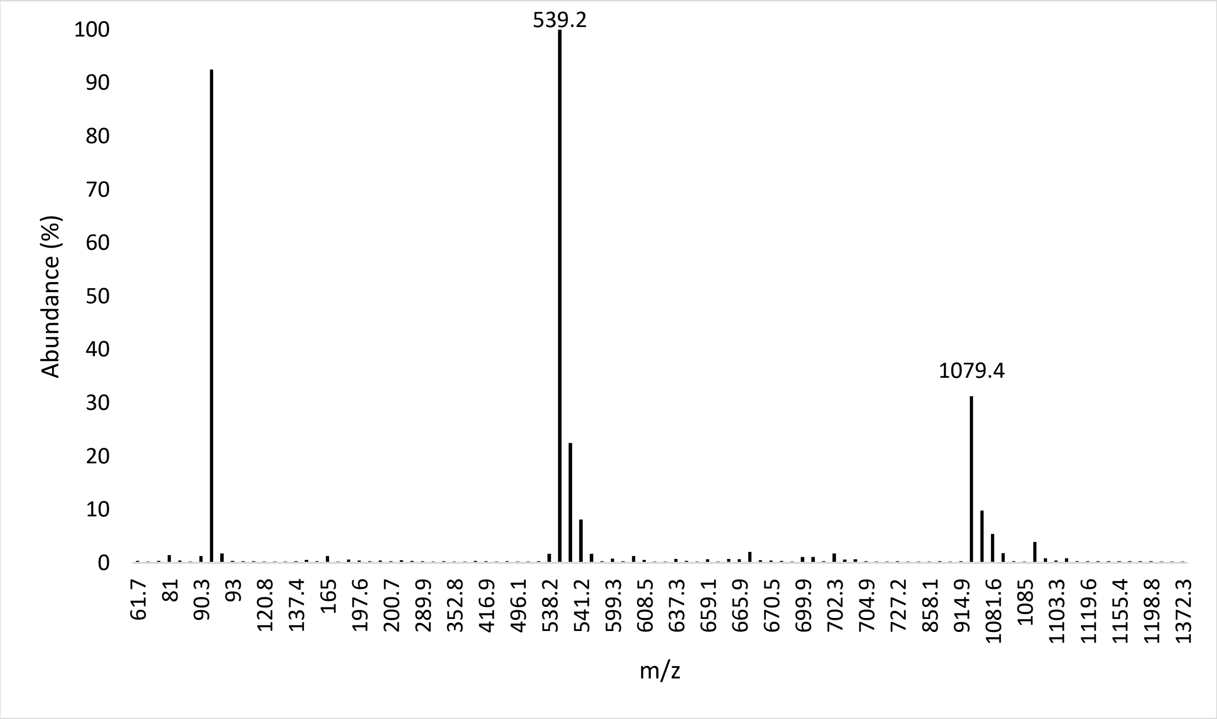

Figure S31. MS spectrum extracted from DA-GA reaction product chromatogram at retention time 17.8 min. The labeled m/z signals represent 9GA.

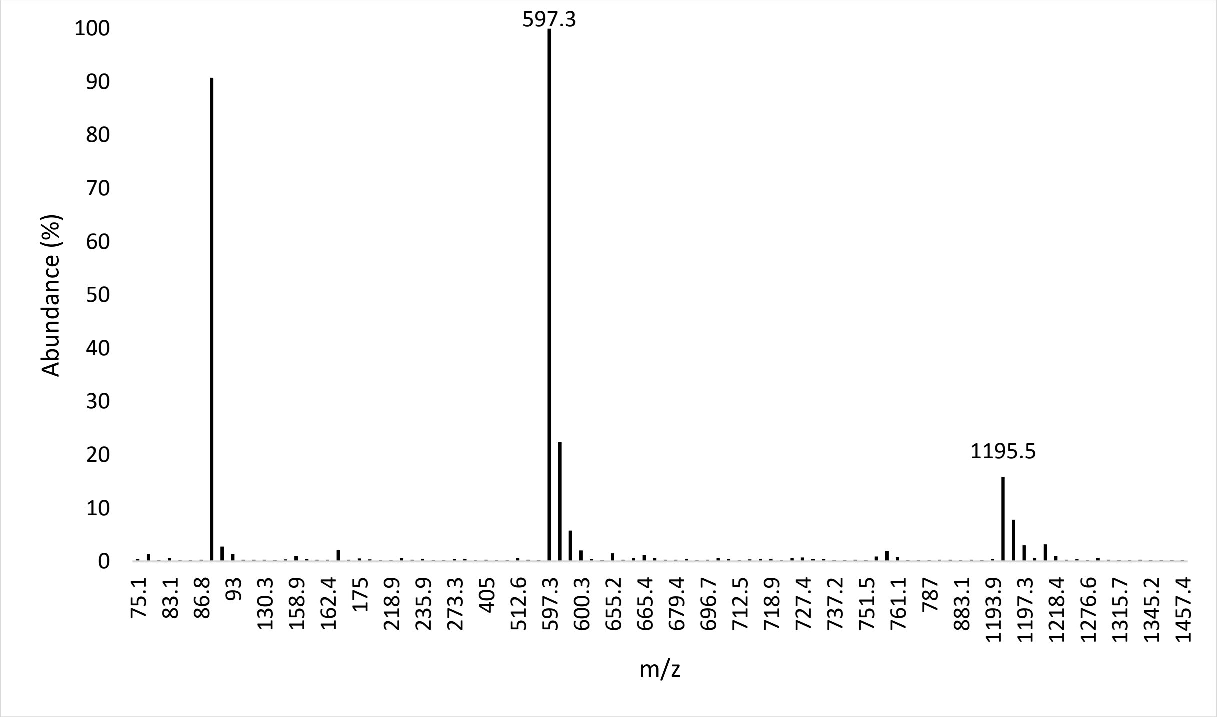

Figure S32. MS spectrum extracted from DA-GA reaction product chromatogram at retention time 18.5 min. The labeled m/z signals represent 10GA.

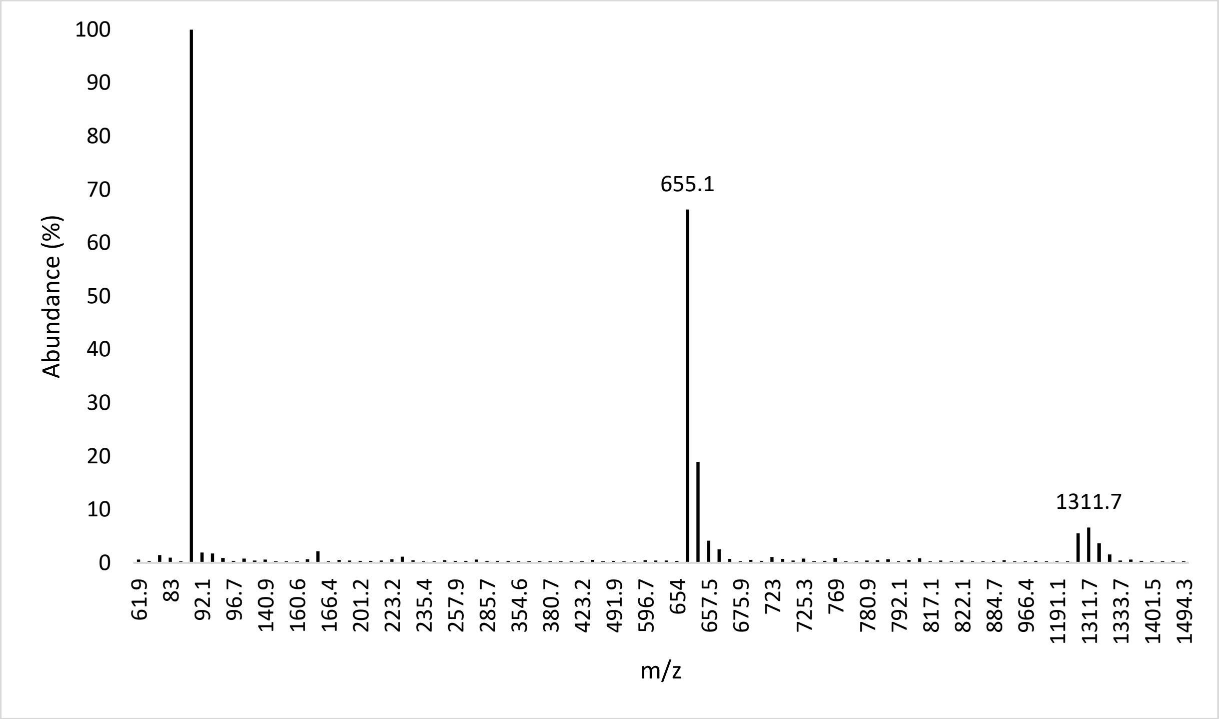

Figure S33. MS spectrum extracted from DA-GA reaction product chromatogram at retention time 19.1 min. The labeled m/z signals represent 11GA.

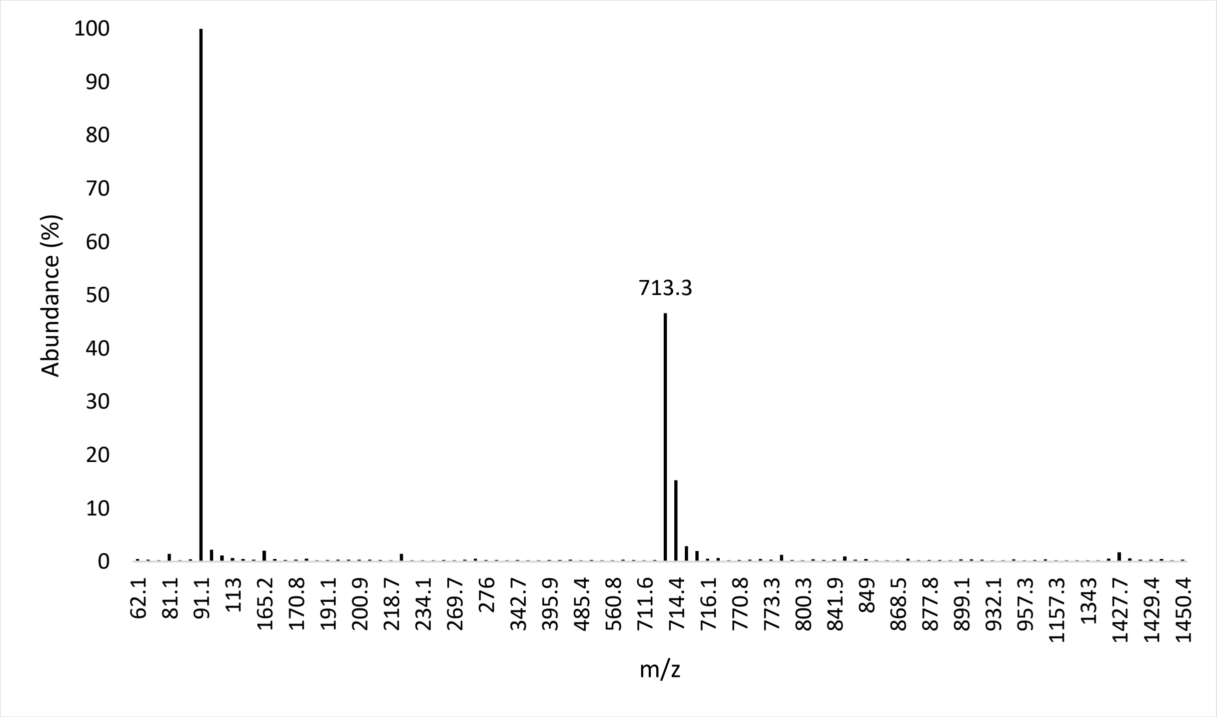

Figure S34. MS spectrum extracted from DA-GA reaction product chromatogram at retention time 19.7 min. The labeled m/z signal represents 12GA.

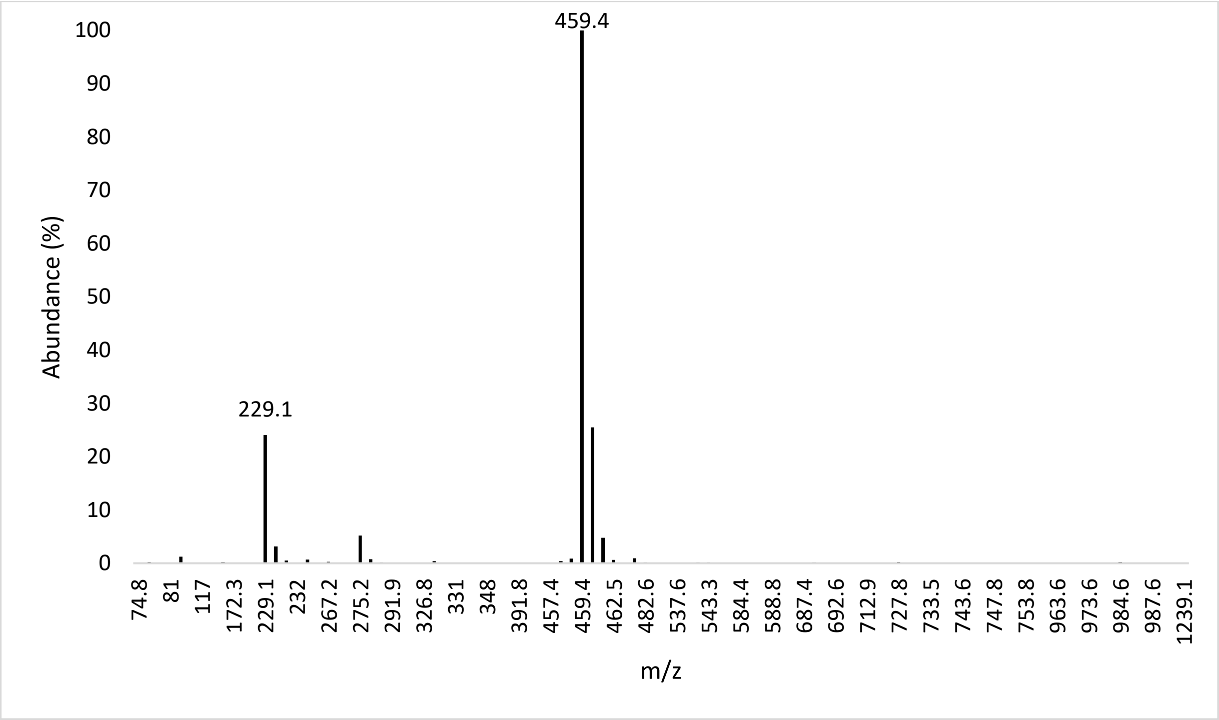

Figure S35. MS spectrum extracted from DA-GA reaction product chromatogram at retention time 28.2 min. The labeled m/z signals represent 1GA1DA.

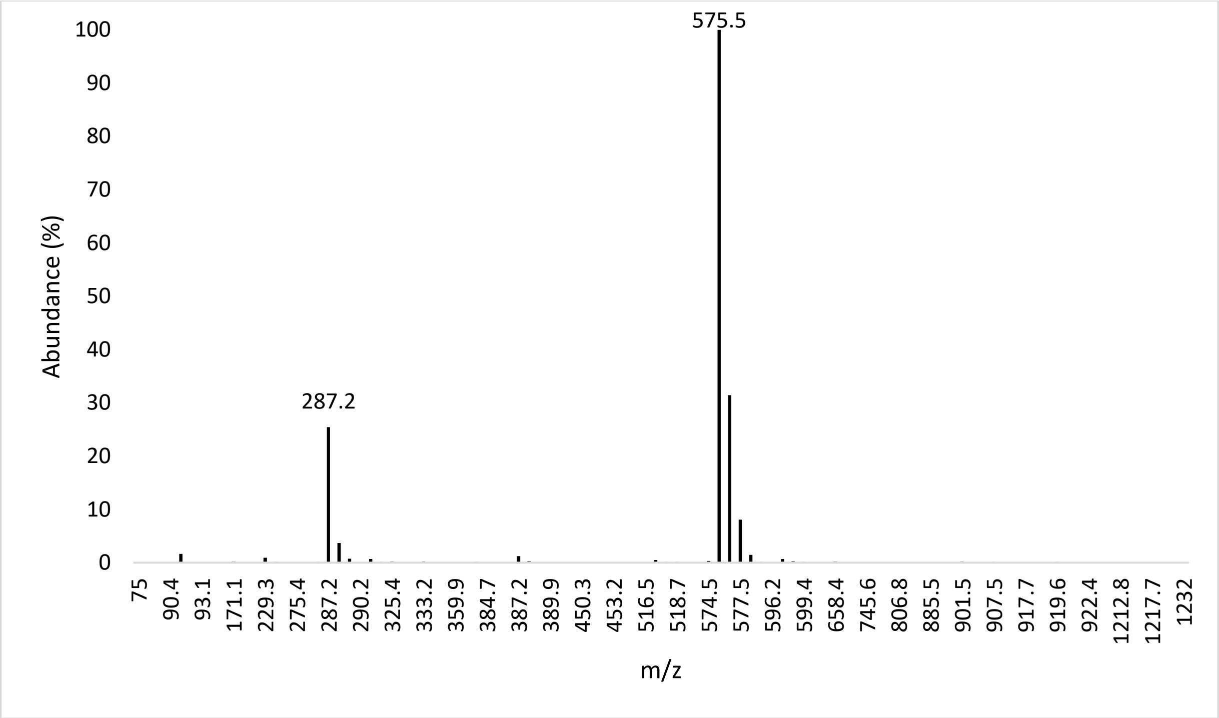

Figure S36. MS spectrum extracted from DA-GA reaction product chromatogram at retention time 28.4 min. The labeled m/z signals represent 2GA1DA.

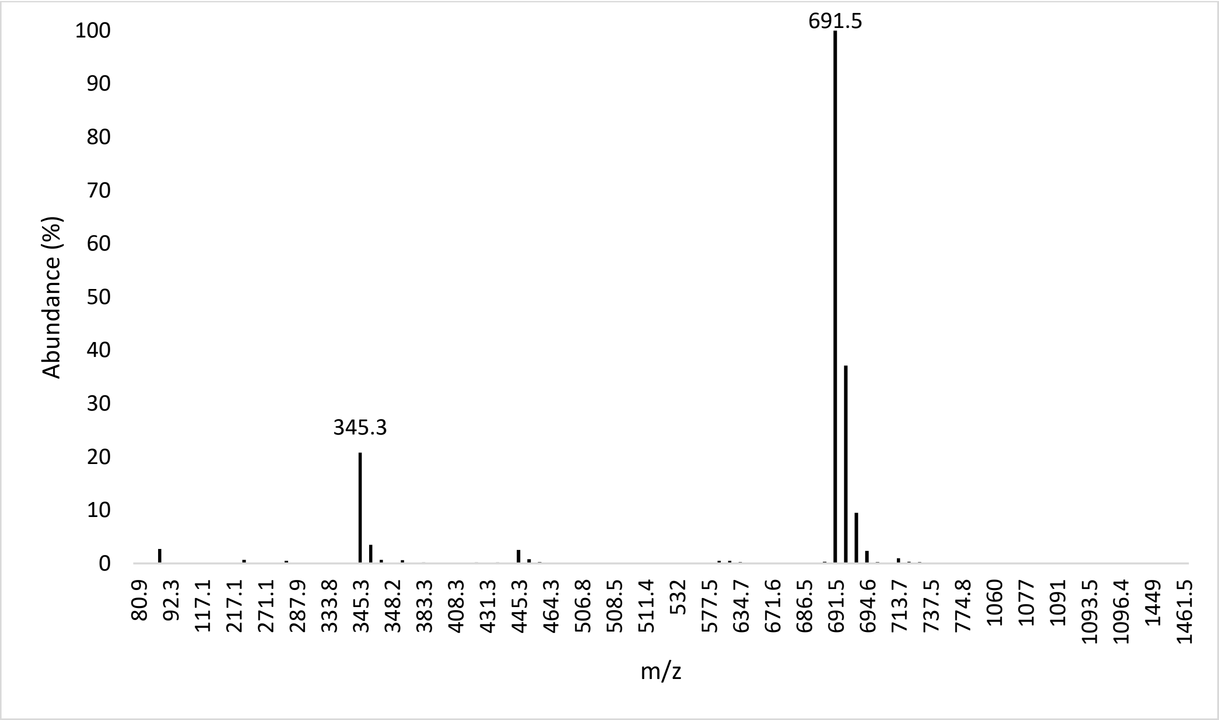

Figure S37. MS spectrum extracted from DA-GA reaction product chromatogram at retention time 28.7 min. The labeled m/z signals represent 3GA1DA.

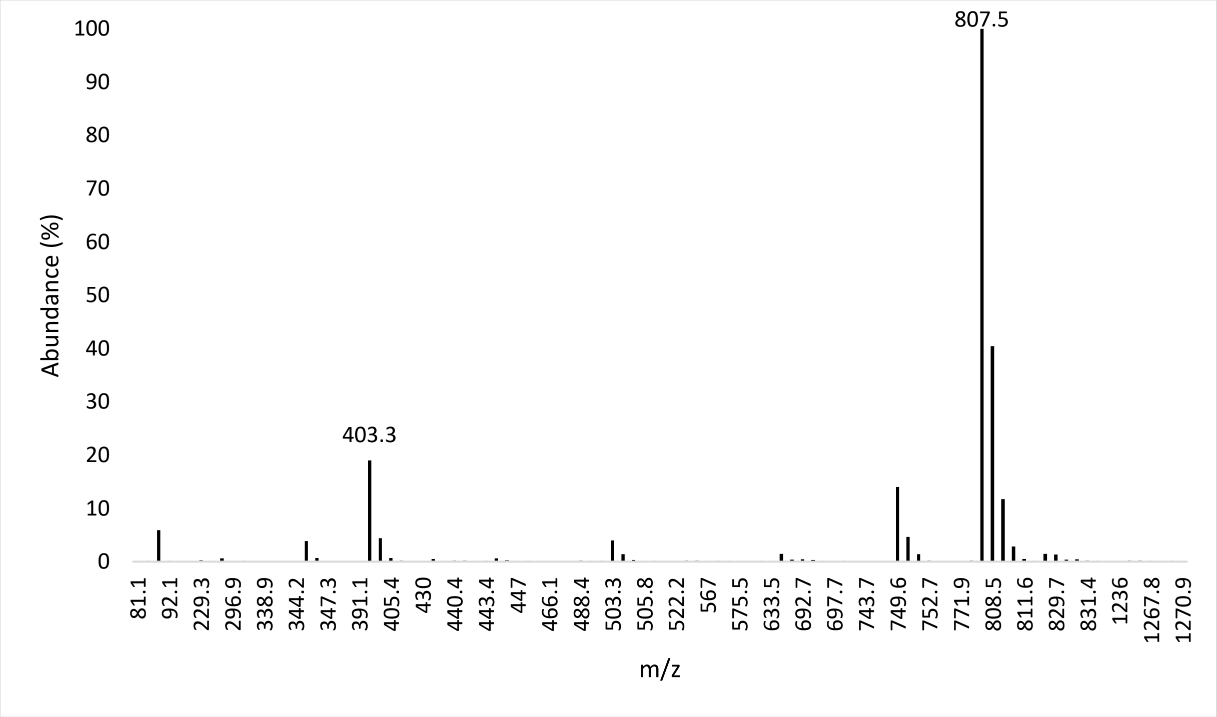

Figure S38. MS spectrum extracted from DA-GA reaction product chromatogram at retention time 28.8 min. The labeled m/z signals represent 4GA1DA.

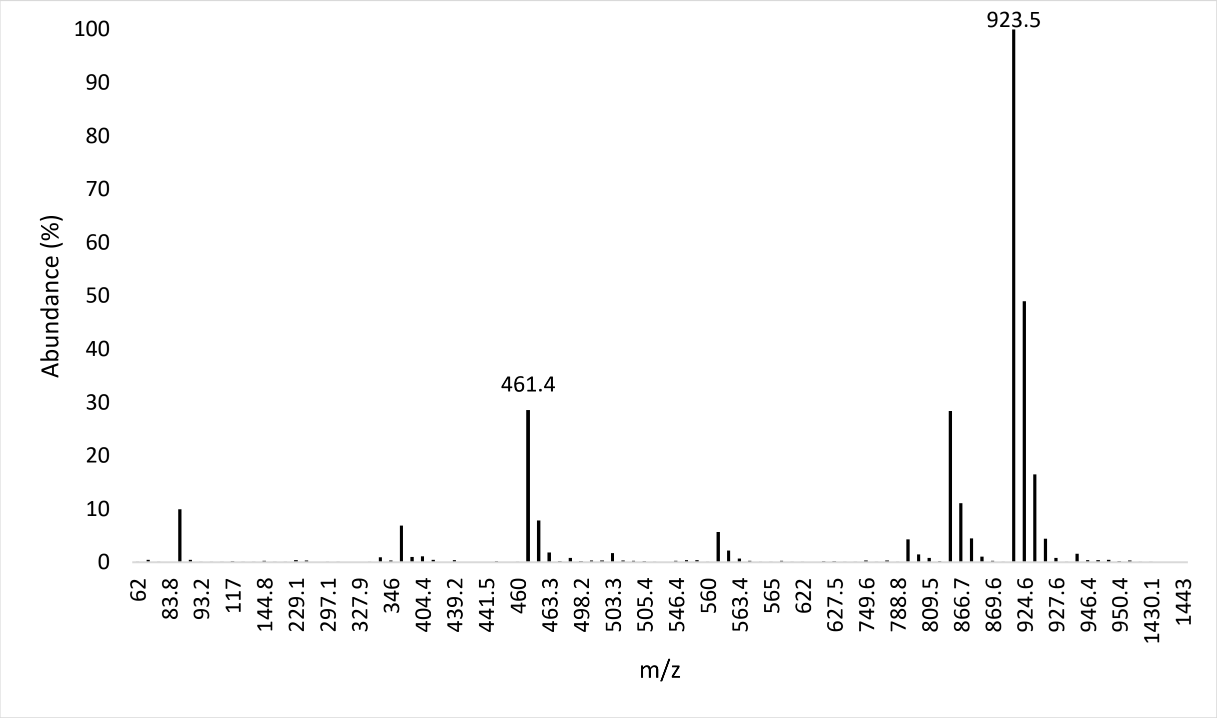

Figure S39. MS spectrum extracted from DA-GA reaction product chromatogram at retention time 28.9 min. The labeled m/z signals represent 5GA1DA.

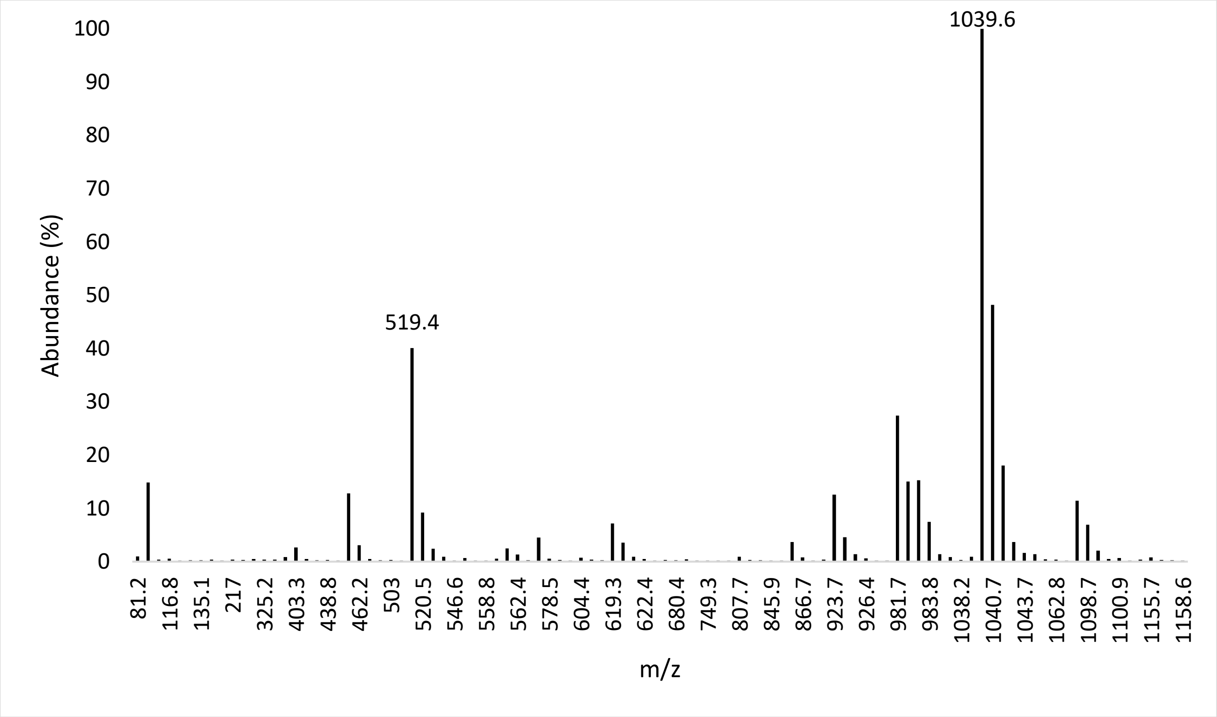

Figure S40. MS spectrum extracted from DA-GA reaction product chromatogram at retention time 29.0 min. The labeled m/z signals represent 6GA1DA.

Table S2. Identification of DA-MA reaction products. The detected products based on retention time and their corresponding m/z and ionization pattern as determined by LC-MS.

Retention time (min)	Compound	M (g/mol)	Corresponding m/z (-TIC)	Ionization pattern
**7.7**	2MA	250.2	249.0	$[M-H]^-$
**8.7**	2MA	250.2	249.1	$[M-H]^-$
**11.4**	3MA	366.2	365.1, 731.4	$[M-H]^-$, $[2M-H]^-$
**12.1**	3MA	366.2	365.2	$[M-H]^-$
	4MA	482.3	481.2	$[M-H]^-$
**12.5**	3MA	366.2	365.1, 731.4	$[M-H]^-$, $[2M-H]^-$
	4MA	482.3	481.2, 963.4	$[M-H]^-$, $[2M-H]^-$
**12.8**	4MA	482.3	481.2, 963.4	$[M-H]^-$, $[2M-H]^-$
	5MA	598.4	597.3	$[M-H]^-$
**13.2**	4MA	482.3	481.2	$[M-H]^-$
	5MA	598.4	597.3, 1195.6	$[M-H]^-$, $[2M-H]^-$
	6MA	714.4	713.4	$[M-H]^-$
**13.4**	4MA	482.3	481.2	$[M-H]^-$
	5MA	598.4	597.3, 1195.6, 298.2	$[M-H]^-$, $[2M-H]^-$, $[M-2H]^{2-}$
	6MA	714.4	713.3, 356.3	$[M-H]^-$, $[M-2H]^{2-}$
**13.6**	5MA	598.4	597.2, 1195.7, 297.9	$[M-H]^-$, $[2M-H]^-$, $[M-2H]^{2-}$
	6MA	714.4	713.4, 356.2	$[M-H]^-$, $[M-2H]^{2-}$
	7MA	830.5	829.5, 414.3	$[M-H]^-$, $[M-2H]^{2-}$
**13.8**	5MA	598.4	597.3	$[M-H]^-$
	6MA	714.4	713.3, 1427.5, 356.1	$[M-H]^-$, $[2M-H]^-$, $[M-2H]^{2-}$
	7MA	830.5	829.4, 414.2	$[M-H]^-$, $[M-2H]^{2-}$
**14.0**	6MA	714.4	713.4, 356.3	$[M-H]^-$, $[M-2H]^{2-}$
	7MA	830.5	829.3, 414.3	$[M-H]^-$, $[M-2H]^{2-}$
	8MA	946.6	945.3,472.3	$[M-H]^-$, $[M-2H]^{2-}$
**14.4**	7MA	830.5	829.3, 414.2	$[M-H]^-$, $[M-2H]^{2-}$
	8MA	946.6	945.5, 472.3	$[M-H]^-$, $[M-2H]^{2-}$
	9MA	1062.7	1061.5, 530.3	$[M-H]^-$, $[M-2H]^{2-}$
	10MA	1178.7	1177.2, 588.5	$[M-H]^-$, $[M-2H]^{2-}$
**14.7**	8MA	946.6	945.3, 472.4	$[M-H]^-$, $[M-2H]^{2-}$
	9MA	1062.7	1061.5, 530.3	$[M-H]^-$, $[M-2H]^{2-}$
	10MA	1178.7	1177.5, 588.4	$[M-H]^-$, $[M-2H]^{2-}$
	11MA	1294.8	1293.6, 646.4	$[M-H]^-$, $[M-2H]^{2-}$
**14.9**	9MA	1062.7	1061.5, 530.4	$[M-H]^-$, $[M-2H]^{2-}$
	10MA	1178.7	1177.4, 588.4	$[M-H]^-$, $[M-2H]^{2-}$
	11MA	1294.8	1293.7, 646.4	$[M-H]^-$, $[M-2H]^{2-}$
	12MA	1410.9	1409.6, 704.6	$[M-H]^-$, $[M-2H]^{2-}$
	13MA	1526.9	762.6	$[M-2H]^{2-}$
**15.0**	9MA	1062.7	1061.5, 530.1	$[M-H]^-$, $[M-2H]^{2-}$
	10MA	1178.7	1177.5, 588.4	$[M-H]^-$, $[M-2H]^{2-}$
	11MA	1294.8	1293.6, 646.5	$[M-H]^-$, $[M-2H]^{2-}$
	12MA	1410.9	1409.6, 704.3	$[M-H]^-$, $[M-2H]^{2-}$
	13MA	1526.9	762.8	$[M-2H]^{2-}$
**15.3**	10MA	1178.7	588.5	$[M-2H]^{2-}$
	11MA	1294.8	646.4	$[M-2H]^{2-}$
	12MA	1410.9	1409.7, 704.5	$[M-H]^-$, $[M-2H]^{2-}$
	13MA	1526.9	762.4	$[M-2H]^{2-}$
	14MA	1643.0	820.6	$[M-2H]^{2-}$
	15MA	1759.1	878.6	$[M-2H]^{2-}$
**15.6**	12MA	1410.9	704.7	$[M-2H]^{2-}$

	13MA	1526.9	762.4	$[M-2H]^{2-}$
	14MA	1643.0	820.2	$[M-2H]^{2-}$
	15MA	1759.1	878.5	$[M-2H]^{2-}$
	16MA	1875.1	936.6	$[M-2H]^{2-}$
**23.6**	3MA1DA	520.5	519.4	$[M-H]^{-}$
	4MA1DA	636.5	635.5	$[M-H]^{-}$
	5MA1DA	752.6	751.5	$[M-H]^{-}$
**23.9**	3MA1DA	520.5	519.4	$[M-H]^{-}$
	4MA1DA	636.5	635.4	$[M-H]^{-}$
**24.3**	2MA1DA	404.4	403.3, 807.8	$[M-H]^{-}$, $[2M-H]^{-}$
	3MA1DA	520.5	519.4	$[M-H]^{-}$
**24.8**	2MA1DA	404.4	403.2	$[M-H]^{-}$
**25.5**	1MA1DA	288.3	287.2, 575.5	$[M-H]^{-}$, $[2M-H]^{-}$

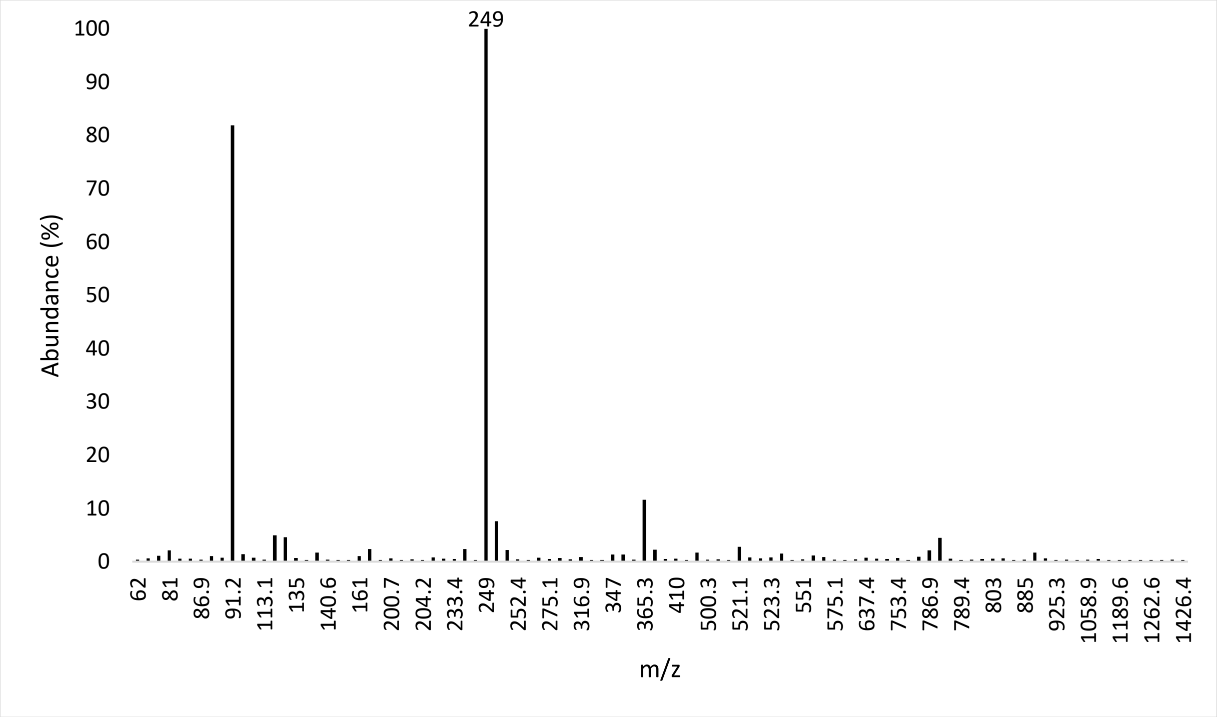

Figure S41. MS spectrum extracted from DA-MA reaction product chromatogram at retention time 7.7 min. The labeled m/z signal represents 2MA.

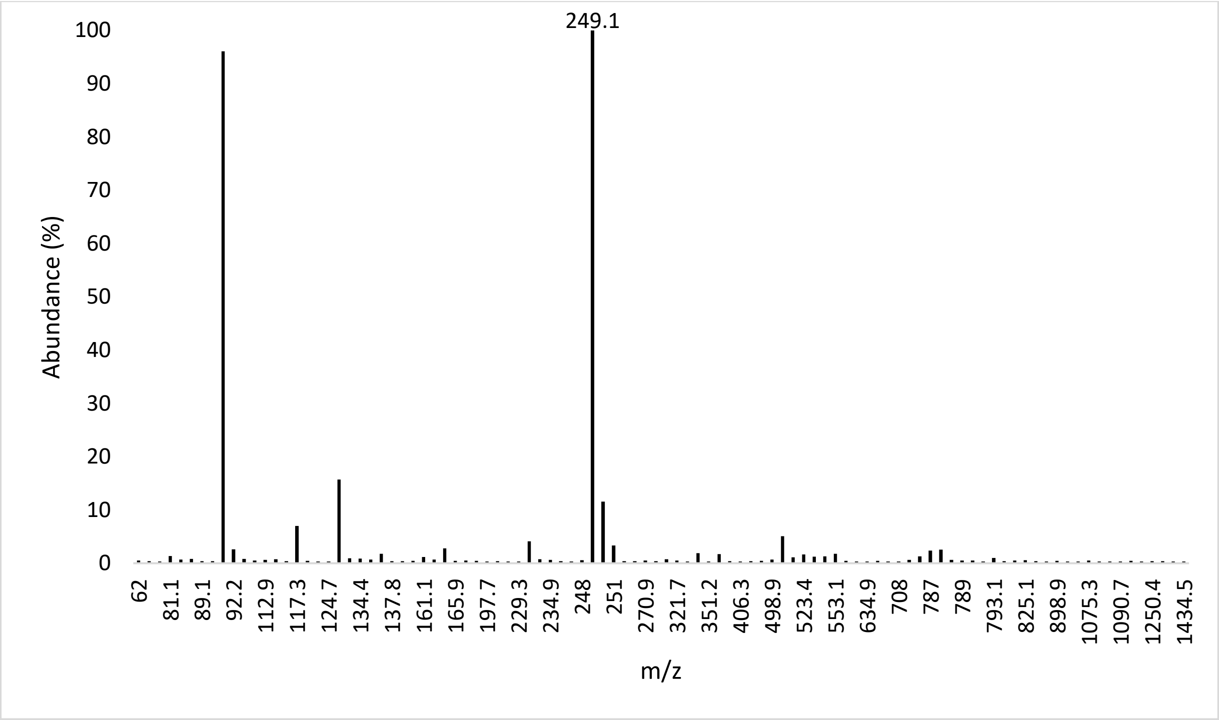

Figure S42. MS spectrum extracted from DA-MA reaction product chromatogram at retention time 8.7 min. The labeled m/z signal represents 2MA.

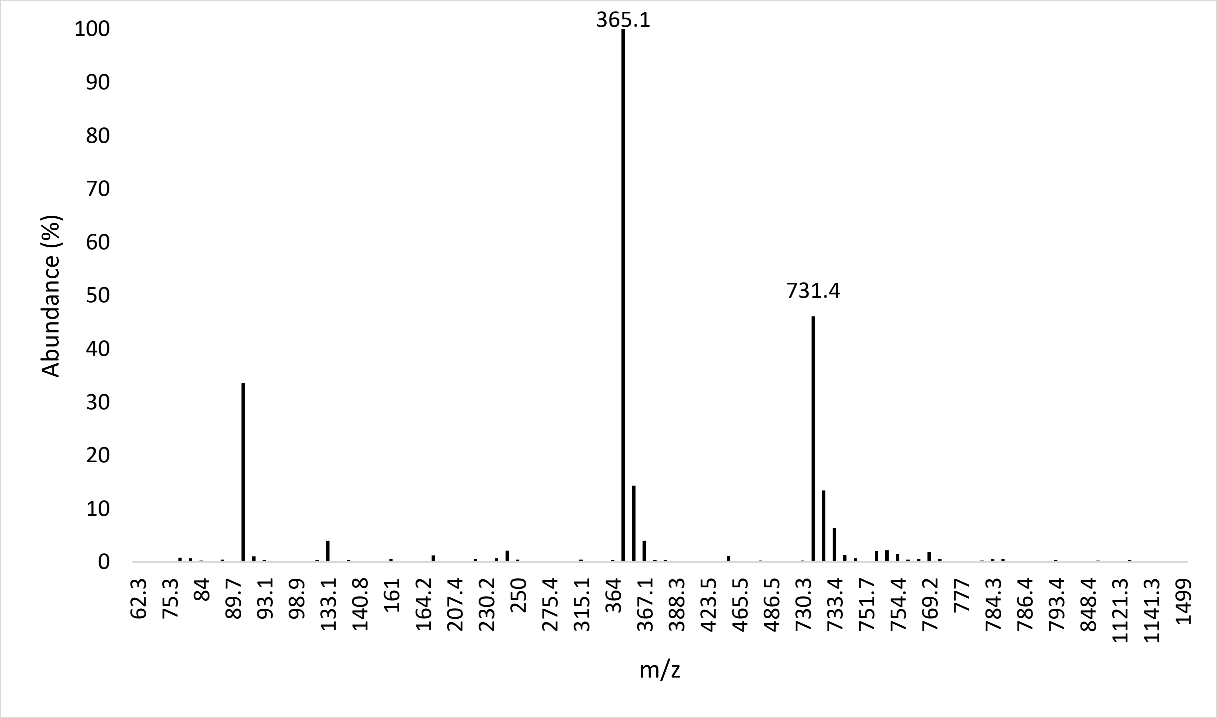

Figure S43. MS spectrum extracted from DA-MA reaction product chromatogram at retention time 11.4 min. The labeled m/z signals represent 3MA.

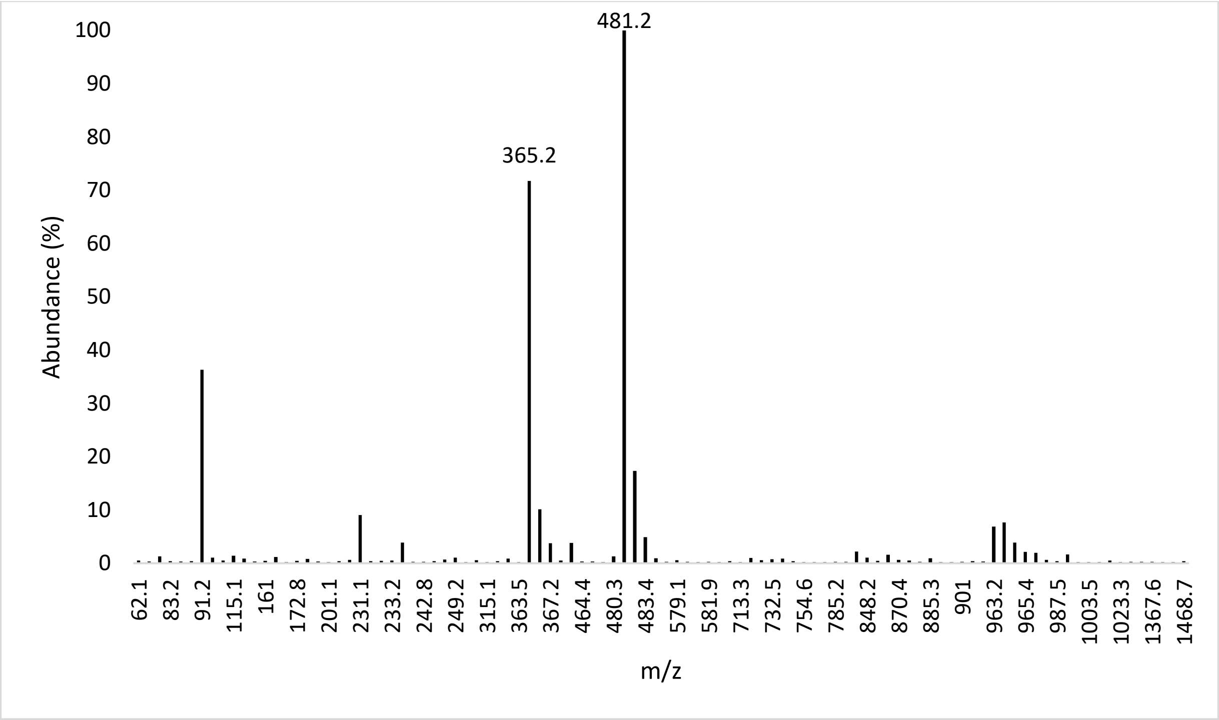

Figure S44. MS spectrum extracted from DA-MA reaction product chromatogram at retention time 12.1 min. The labeled m/z signals represent 3MA and 4MA.

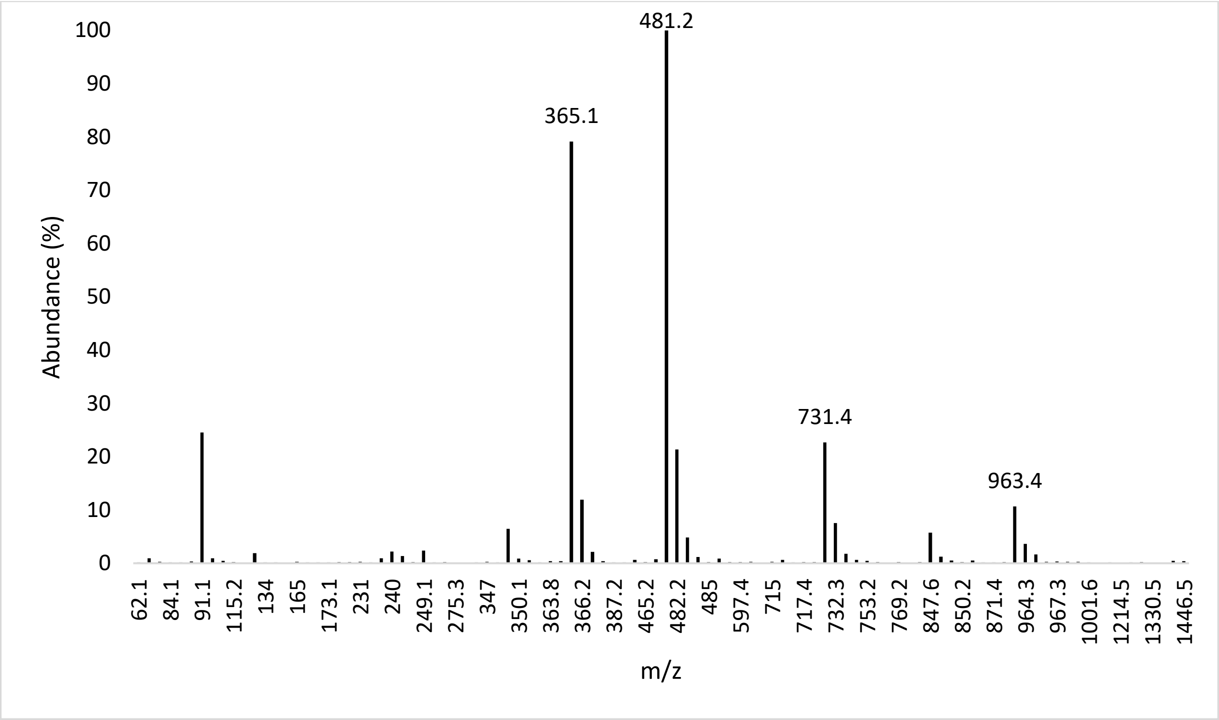

Figure S45. MS spectrum extracted from DA-MA reaction product chromatogram at retention time 12.5 min. The labeled m/z signals represent 3MA and 4MA.

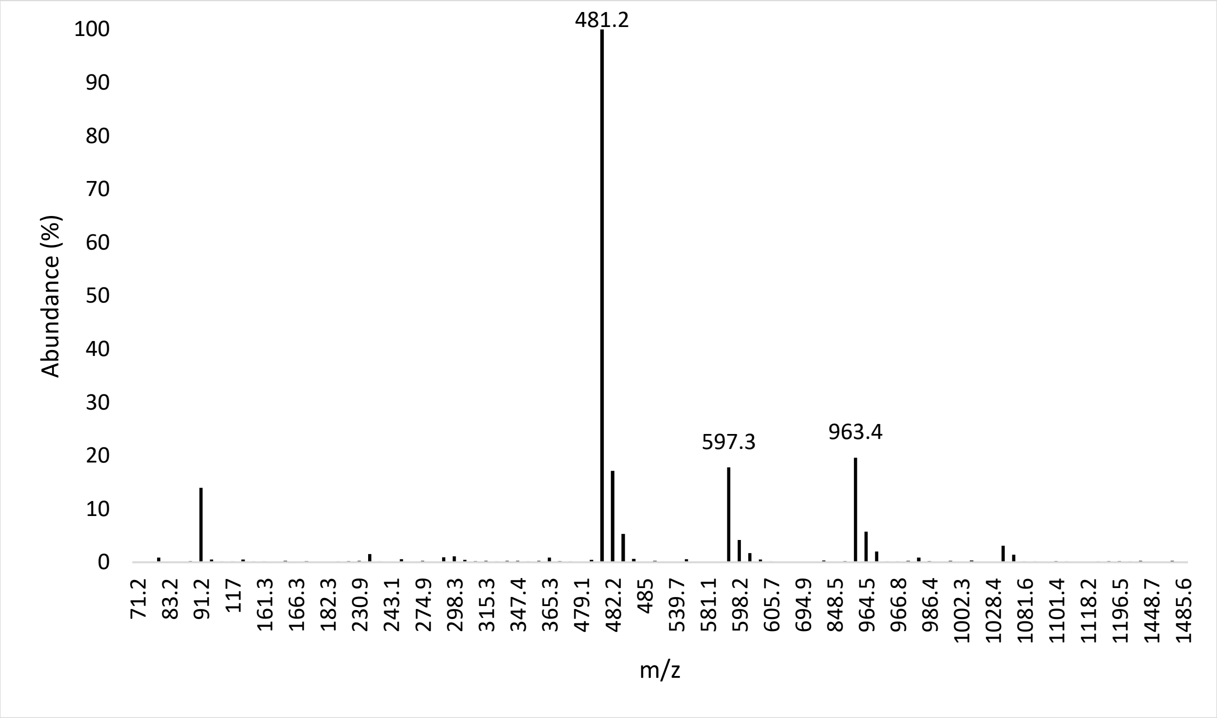

Figure S46. MS spectrum extracted from DA-MA reaction product chromatogram at retention time 12.8 min. The labeled m/z signals represent 4MA and 5MA.

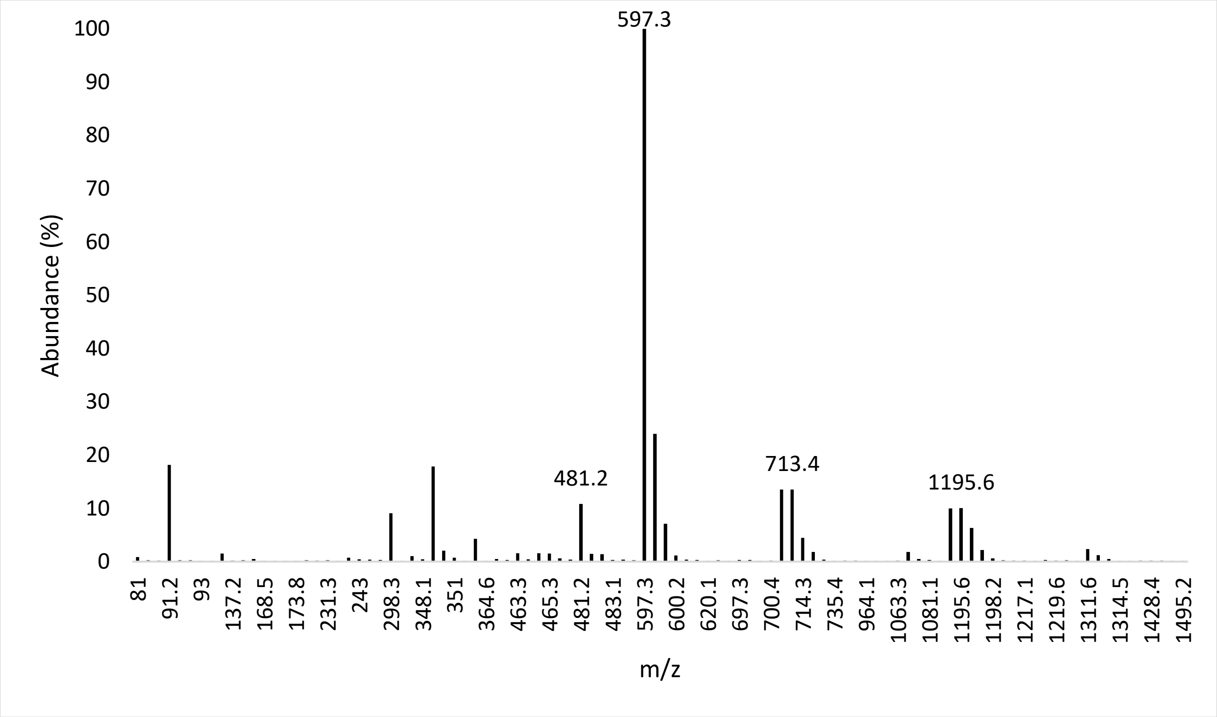

Figure S47. MS spectrum extracted from DA-MA reaction product chromatogram at retention time 13.2 min. The labeled m/z signals represent 4MA, 5MA and 6MA.

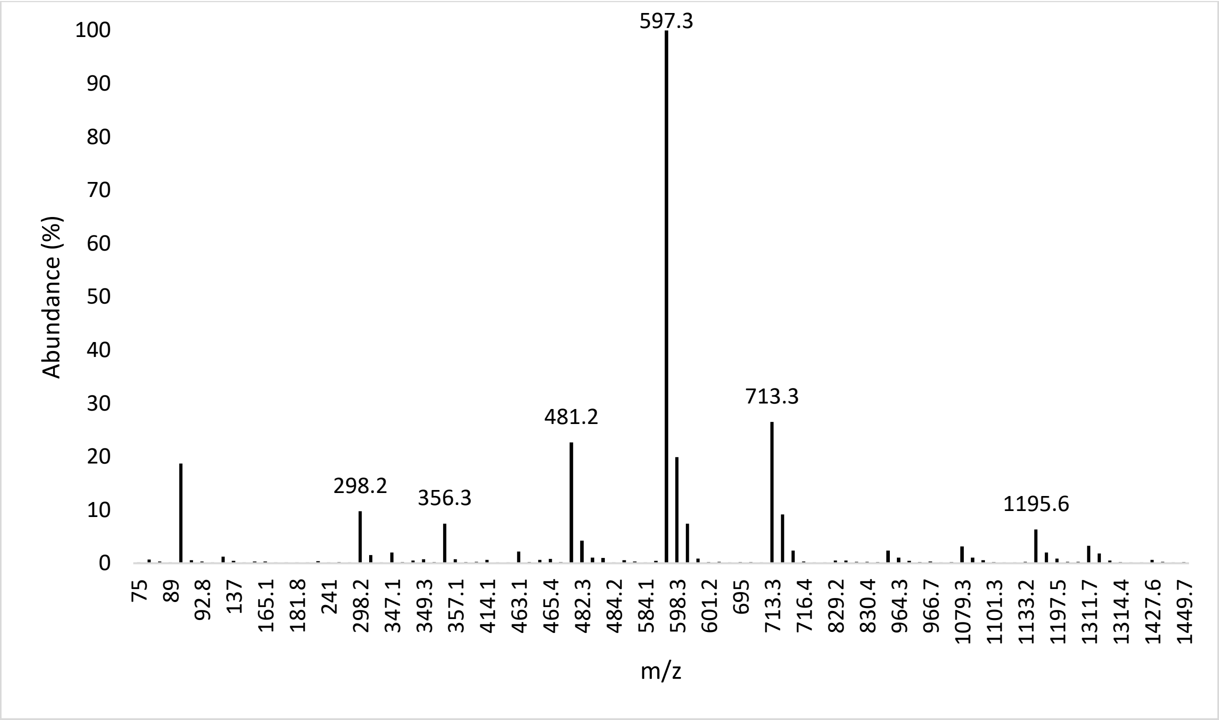

Figure S48. MS spectrum extracted from DA-MA reaction product chromatogram at retention time 13.4 min. The labeled m/z signals represent 4MA, 5MA and 6MA.

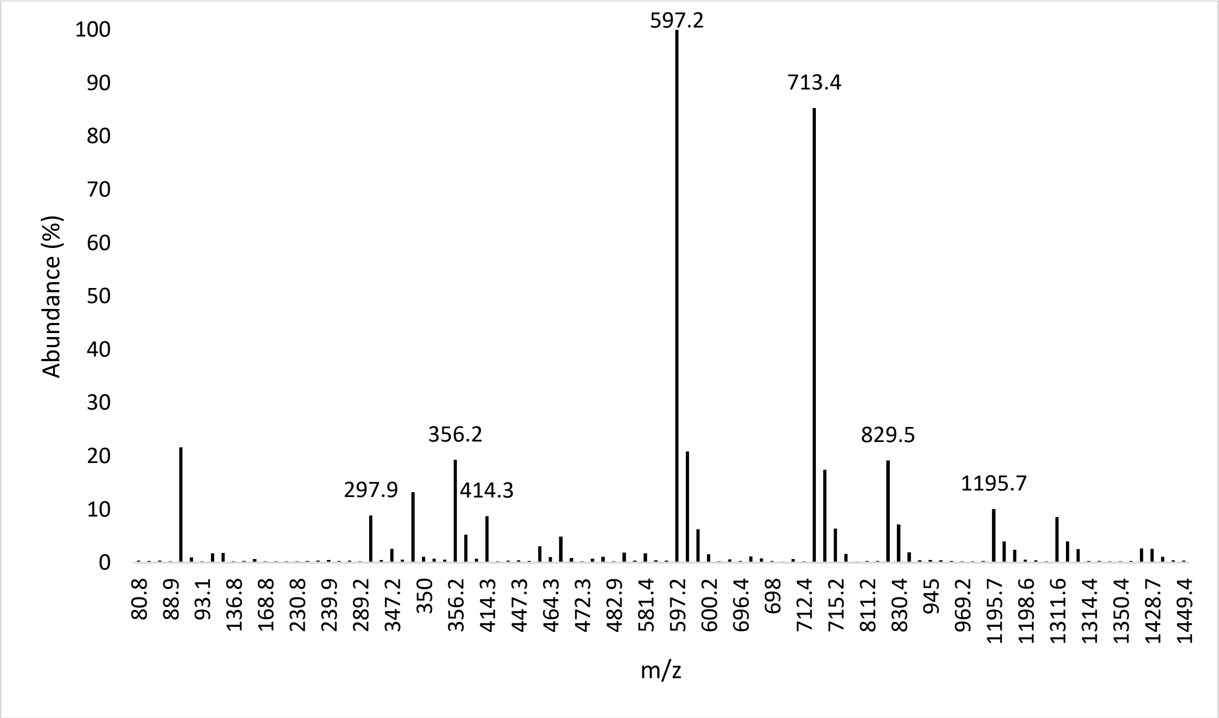

Figure S49. MS spectrum extracted from DA-MA reaction product chromatogram at retention 13.6 min. The labeled m/z signals represent 5MA, 6MA and 7MA.

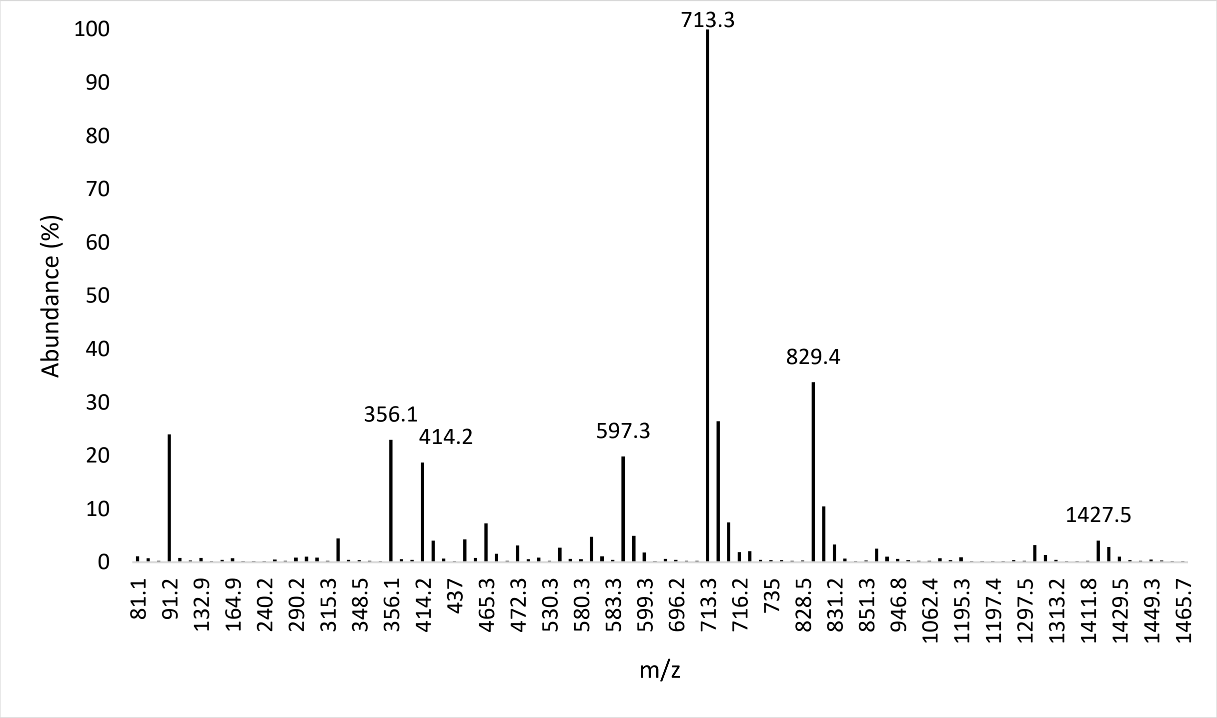

Figure S50. MS spectrum extracted from DA-MA reaction product chromatogram at retention time 13.8 min. The labeled m/z signals represent 5MA, 6MA and 7MA.

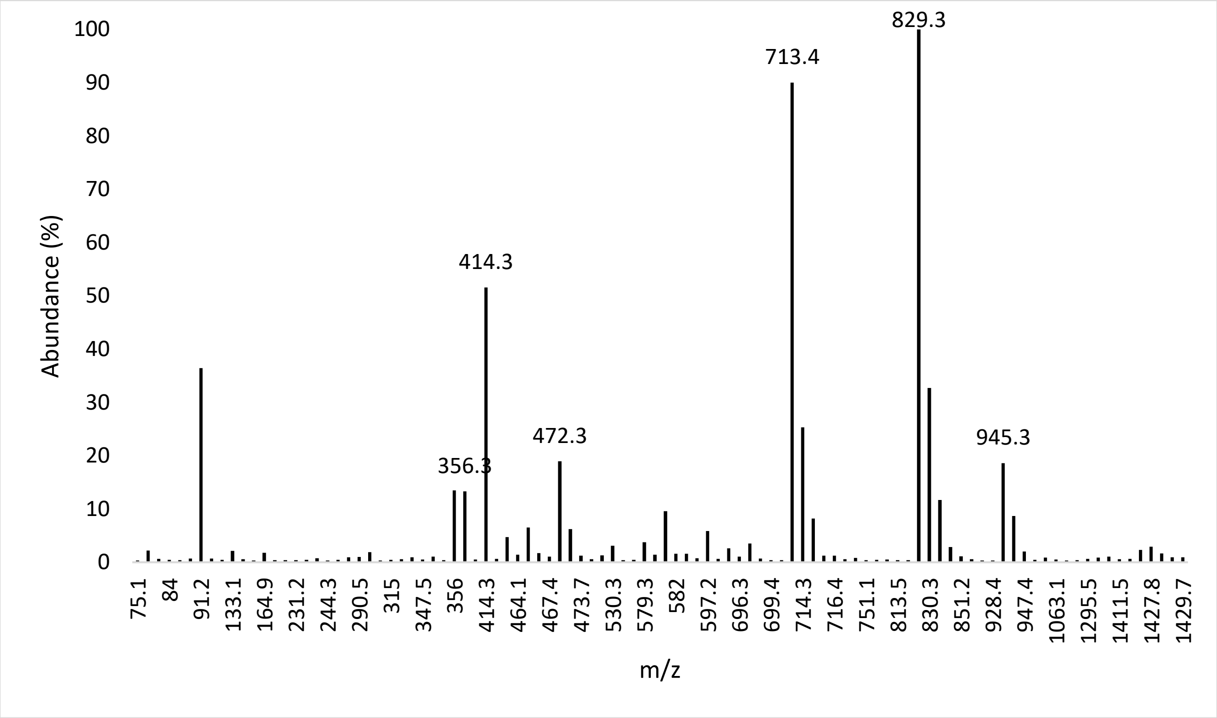

Figure S51. MS spectrum extracted from DA-MA reaction product chromatogram at retention time 14.0 min. The labeled m/z signals represent 6MA, 7MA and 8MA.

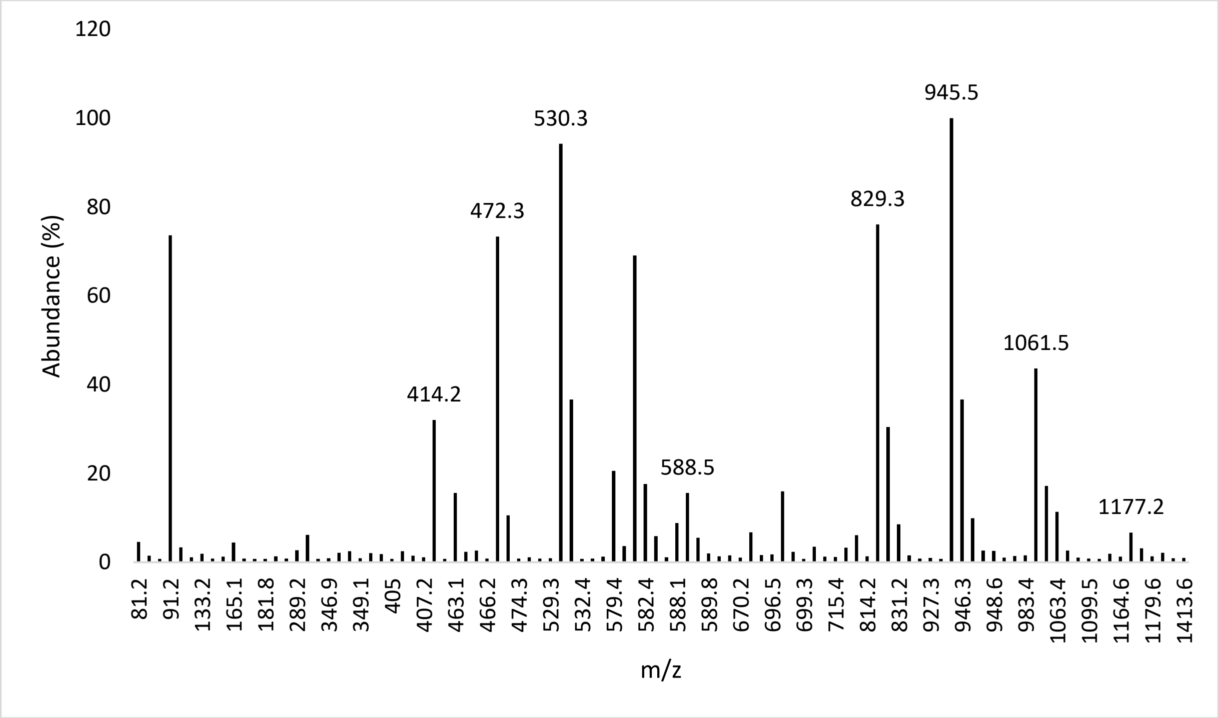

Figure S52. MS spectrum extracted from DA-MA reaction product chromatogram at retention time 14.4 min. The labeled m/z signals represent 7MA, 8MA, 9MA and 10MA.

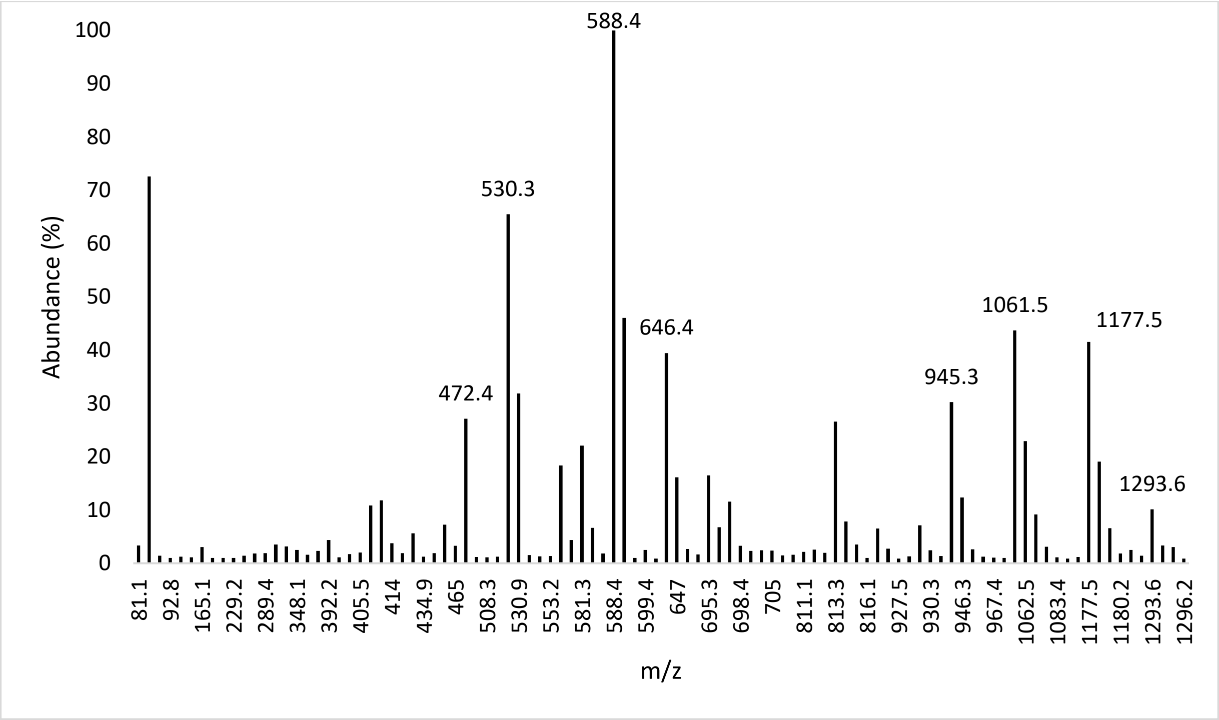

Figure S53. MS spectrum extracted from DA-MA reaction product chromatogram at retention time 14.7 min. The labeled m/z signals represent 8MA, 9MA, 10MA and 11MA.

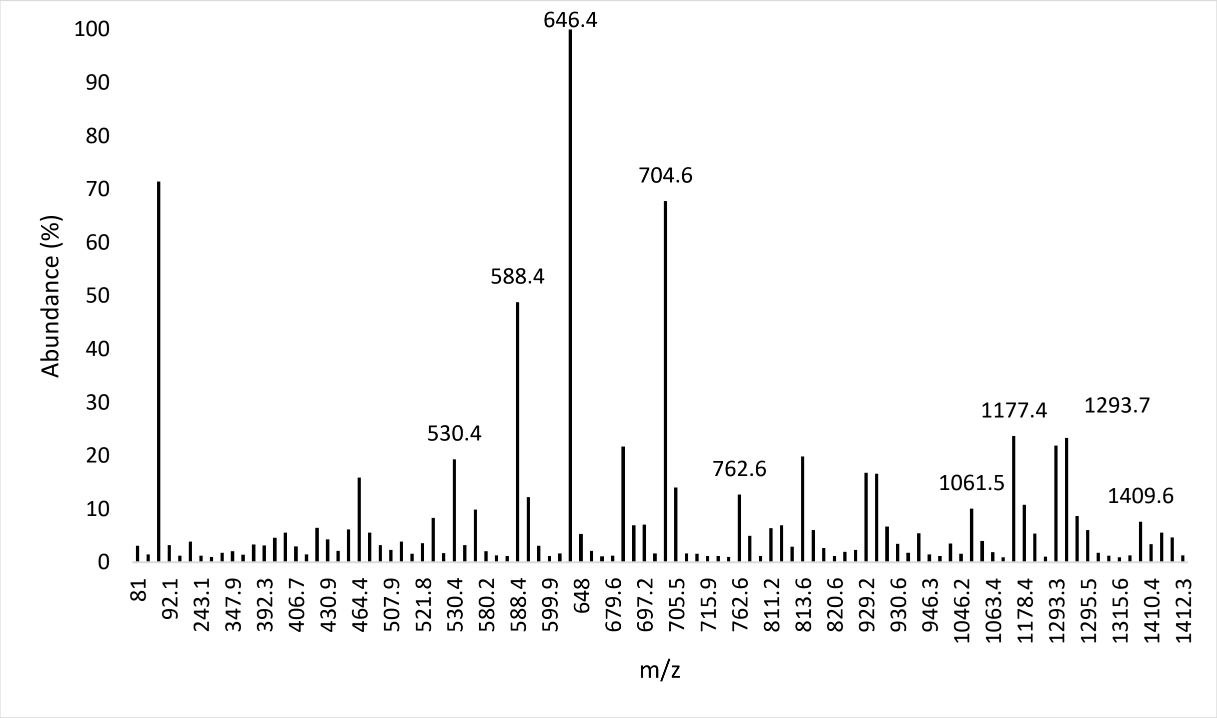

Figure S54. MS spectrum extracted from DA-MA reaction product chromatogram at retention time 14.9 min. The labeled m/z signals represent 7MA, 8MA, 9MA and 10MA.

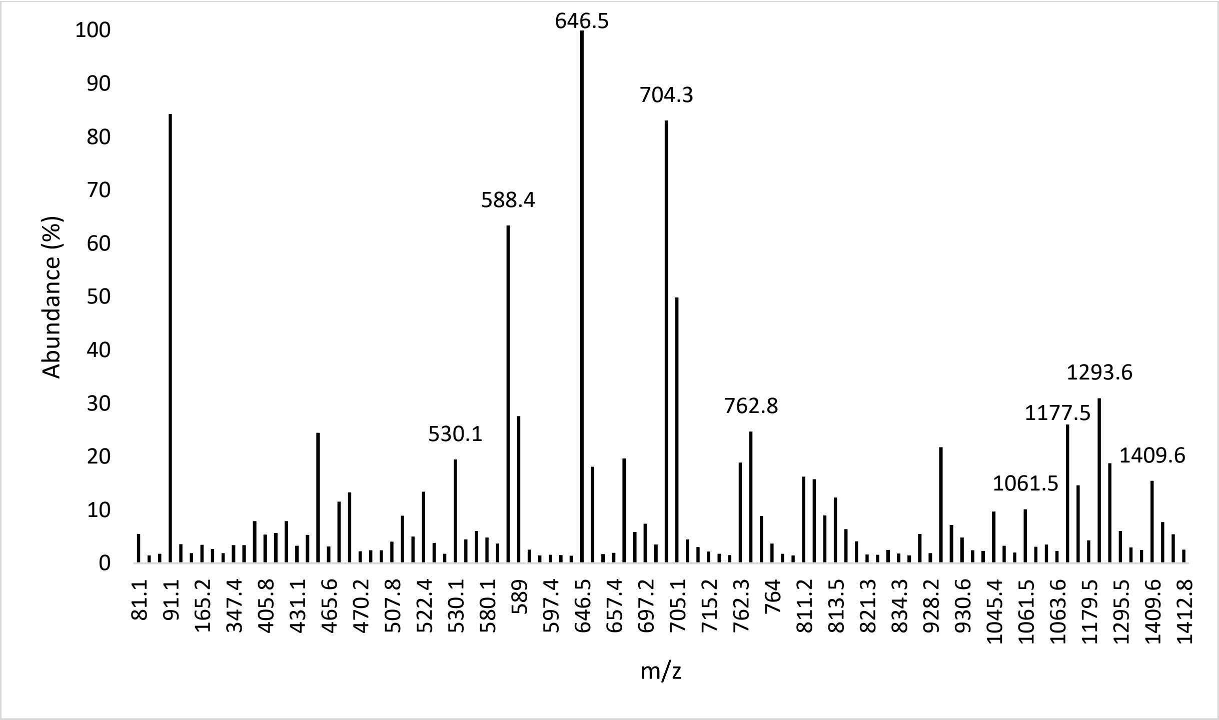

Figure S55. MS spectrum extracted from DA-MA reaction product chromatogram at retention time 15.0 min. The labeled m/z signals represent 9MA, 10MA, 11MA, 12MA and 13MA.

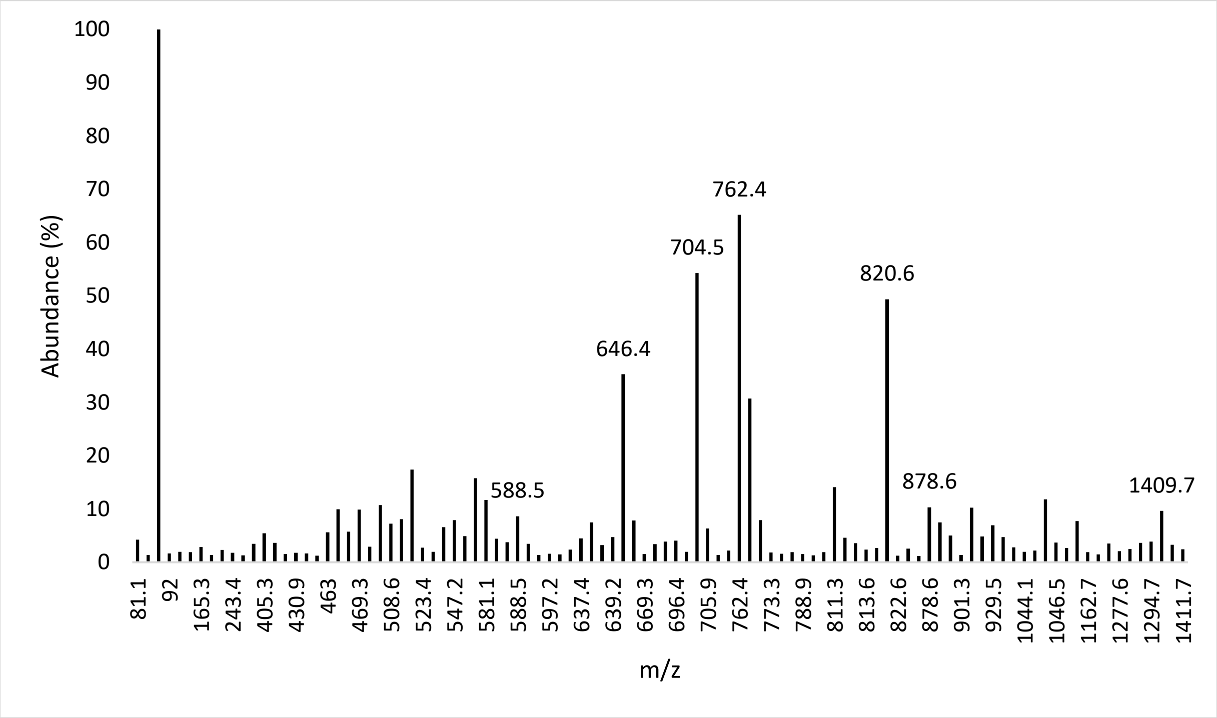

Figure S56. MS spectrum extracted from DA-MA reaction product chromatogram at retention time 15.3 min. The labeled m/z signals represent 10MA, 11MA, 12MA, 13MA, 14MA and 15MA.

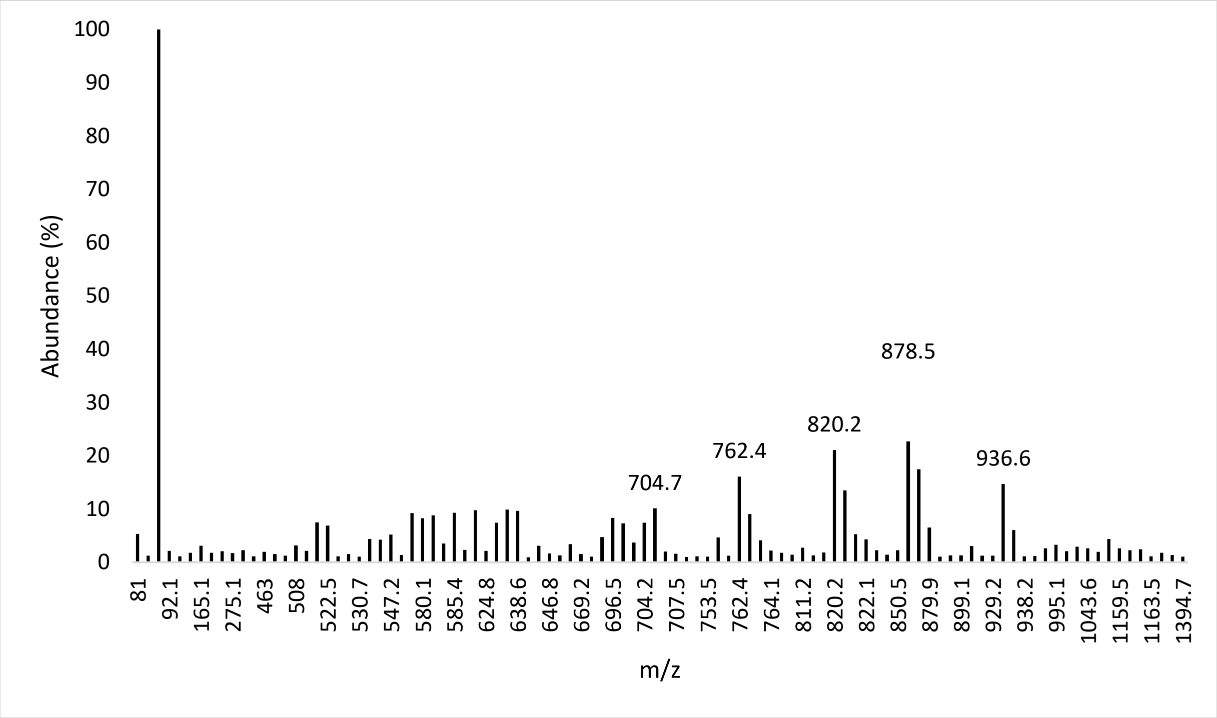

Figure S57. MS spectrum extracted from DA-MA reaction product chromatogram at retention time 15.6 min. The labeled m/z signals represent 12MA, 13MA, 14MA, 15MA and 16MA.

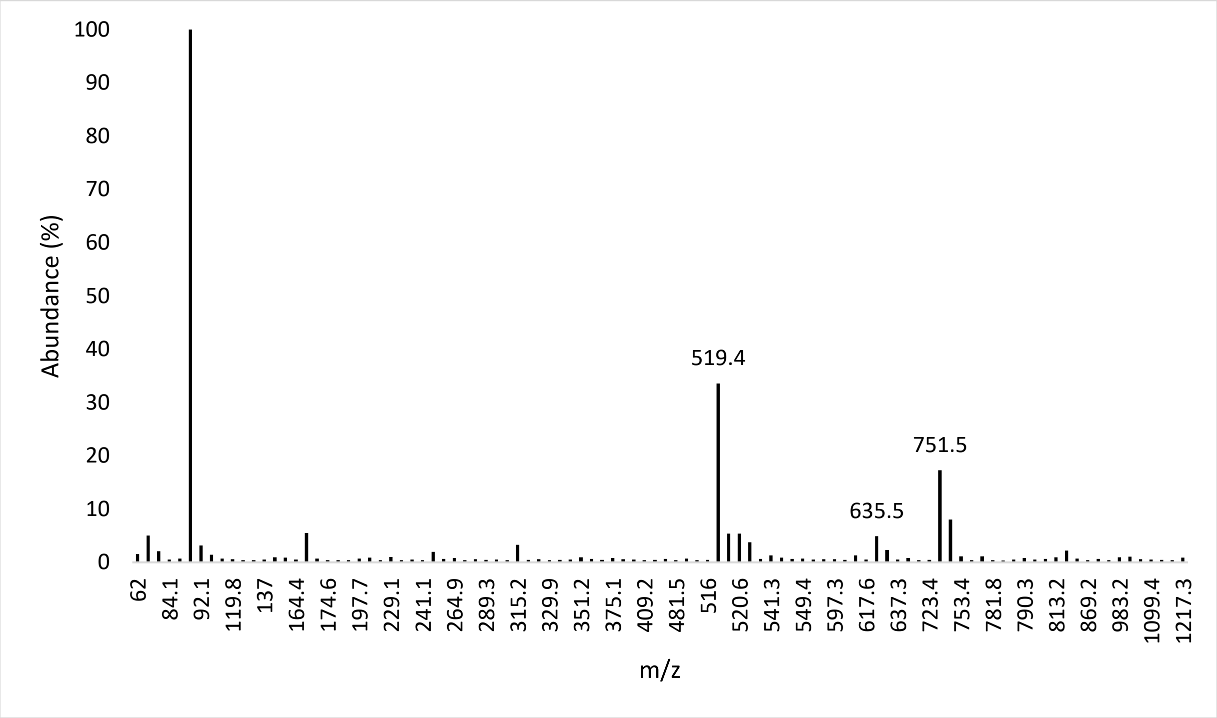

Figure S58. MS spectrum extracted from DA-MA reaction product chromatogram at retention time 23.6 min. The labeled m/z signals represent 3MA1DA, 4MA1DA and 5MA1DA.

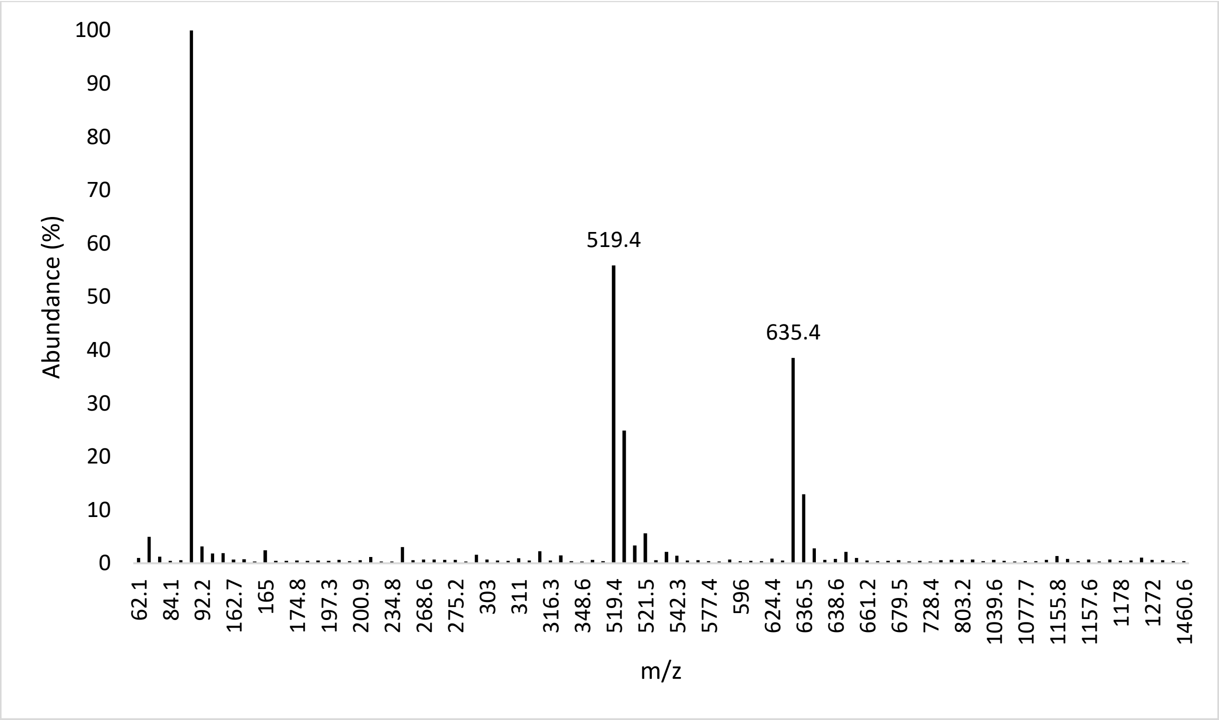

Figure S59. MS spectrum extracted from DA-MA reaction product chromatogram at retention time 23.9 min. The labeled m/z signals represent 3MA1DA, and 4MA1DA.

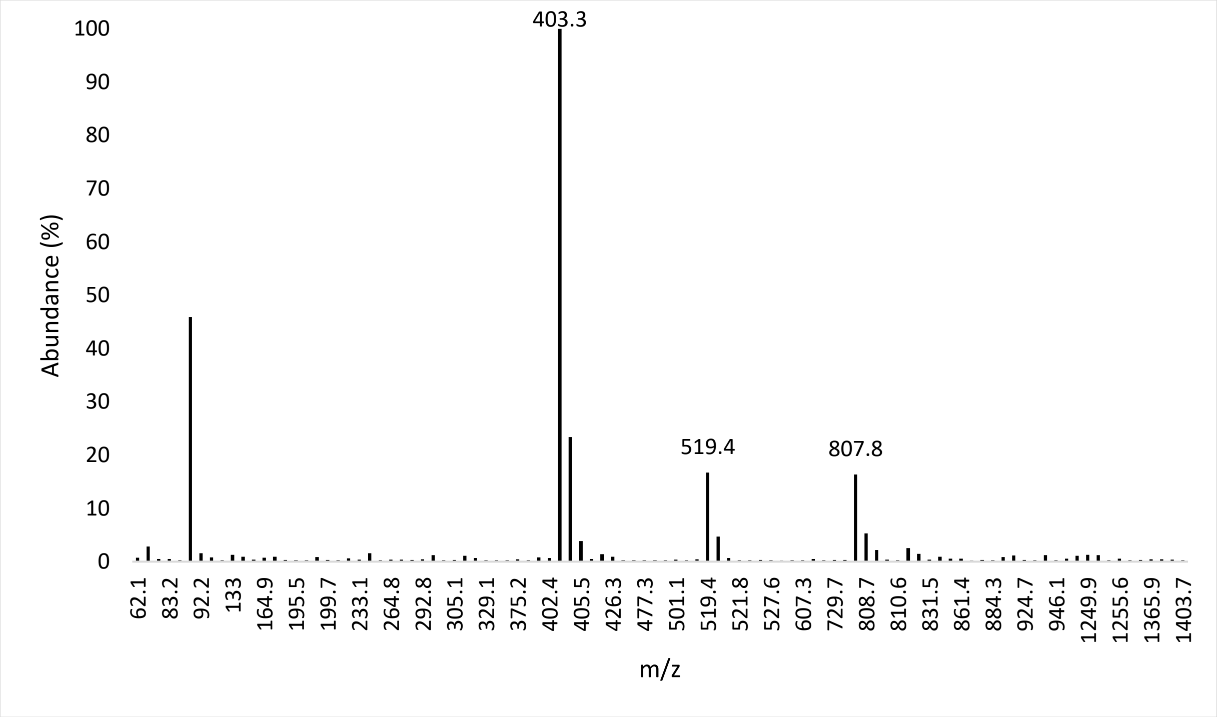

Figure S60. MS spectrum extracted from DA-MA reaction product chromatogram at retention time 24.3 min. The labeled m/z signals represent 2MA1DA, and 3MA1DA.

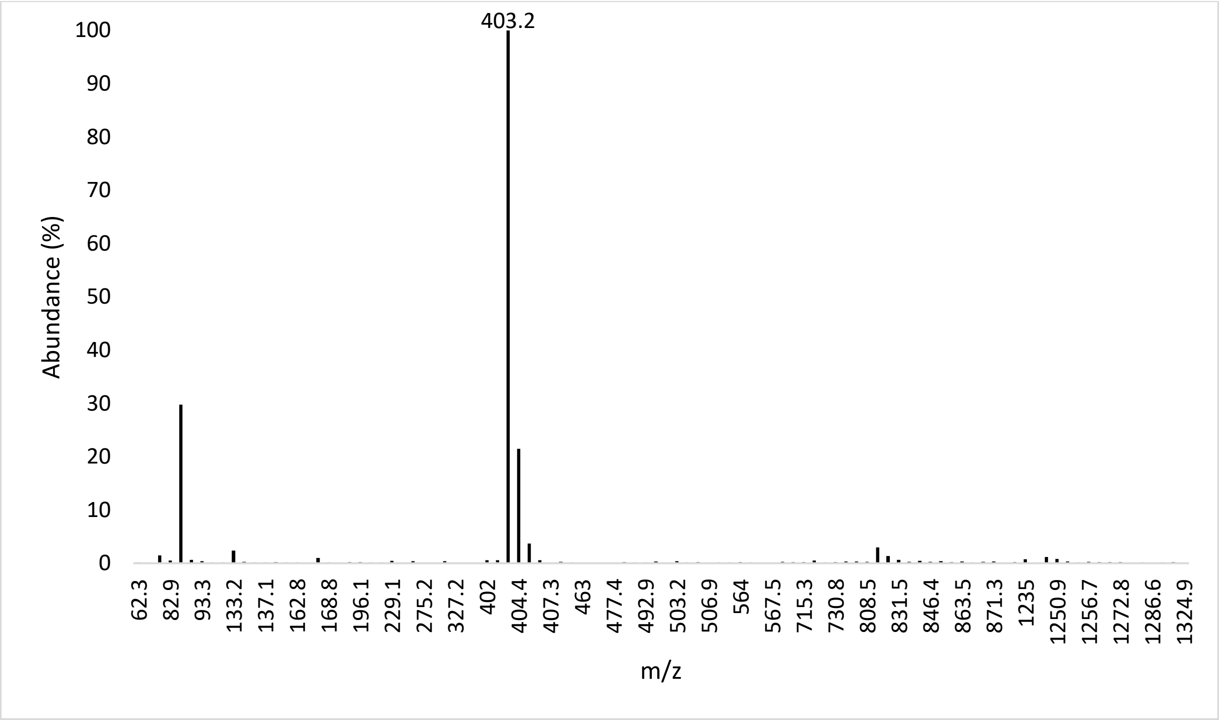

Figure S61. MS spectrum extracted from DA-MA reaction product chromatogram at retention time 24.8 min. The labeled m/z signal represents 2MA1DA.

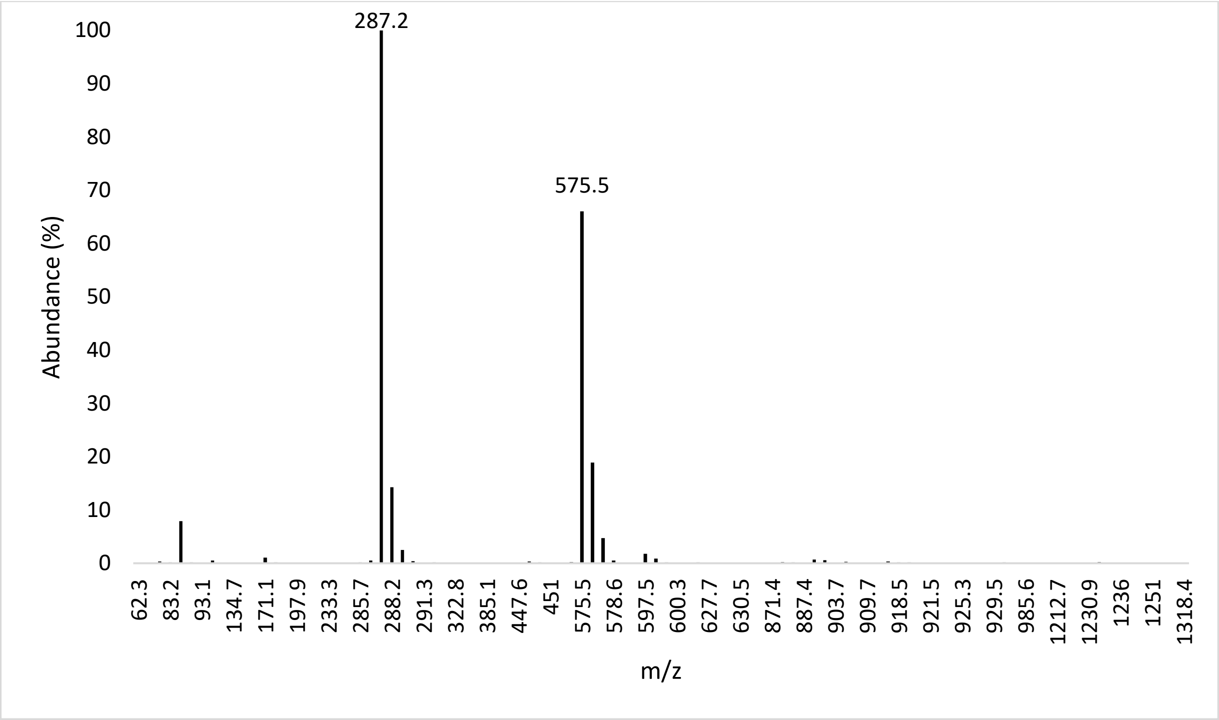

Figure S62. MS spectrum extracted from DA-MA reaction product chromatogram at retention 25.5 min. The labeled m/z signals represent 1MA1DA.

Table S3. Identification of DA-PLA reaction products. The detected products based on retention time and their corresponding m/z and ionization pattern as determined by LC-MS.

Retention time (min)	Compound	M (g/mol)	Corresponding m/z (-TIC)	Ionization pattern
**23.1**	2PLA	314.3	313.1, 627.5	$[M-H]^-$, $[2M-H]^-$
**26.6**	3PLA	462.5	461.3, 923.5	$[M-H]^-$, $[2M-H]^-$
**29.0**	4PLA	610.6	609.4, 1219.7	$[M-H]^-$, $[2M-H]^-$
**30.0**	5PLA	758.8	757.6	$[M-H]^-$
**30.4**	6PLA	906.9	905.5	$[M-H]^-$
	1PLA1DA	320.4	319.3, 639.7	$[M-H]^-$, $[2M-H]^-$
**30.7**	7PLA	1055.1	1053.7	$[M-H]^-$
	1PLA1DA	320.4	319.4, 639.7	$[M-H]^-$, $[2M-H]^-$
**31.4**	2PLA1DA	468.6	467.6, 936.6	$[M-H]^-$, $[2M-H]^-$
**32.2**	3PLA1DA	616.7	615.6, 1232.8	$[M-H]^-$, $[2M-H]^-$
**33.0**	4PLA1DA	764.9	763.6	$[M-H]^-$
**33.8**	5PLA1DA	913.0	911.7	$[M-H]^-$
**34.8**	6PLA1DA	1061.2	1059.6	$[M-H]^-$

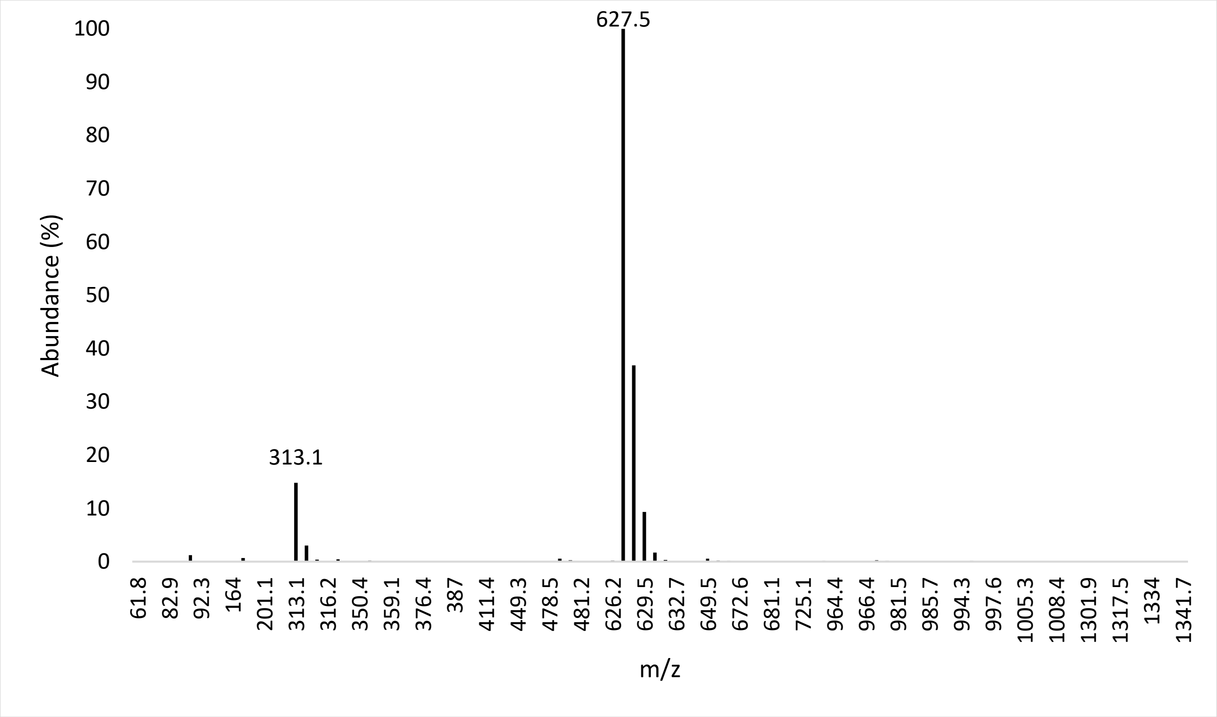

Figure S63. MS spectrum extracted from DA-PLA reaction product chromatogram at retention time 23.1 min. Labeled m/z signals represent 2PLA.

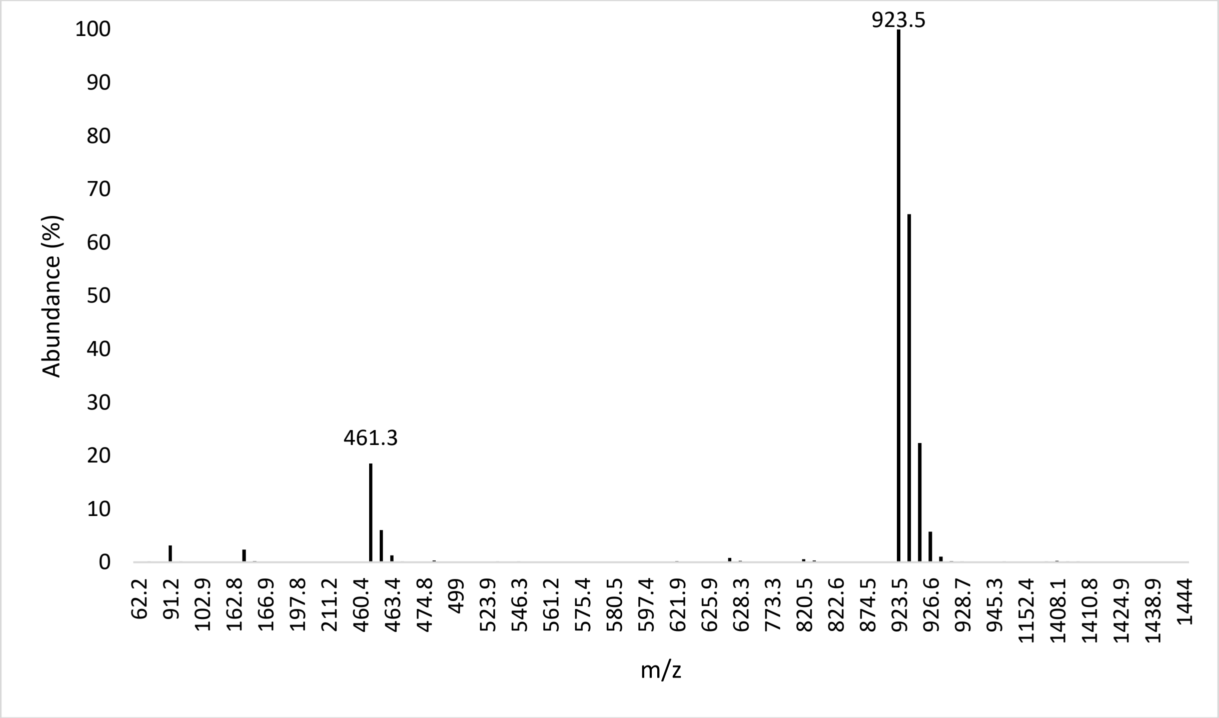

Figure S64. MS spectrum extracted from DA-PLA reaction product chromatogram at retention time 26.6 min. Labeled m/z signals represent 3PLA.

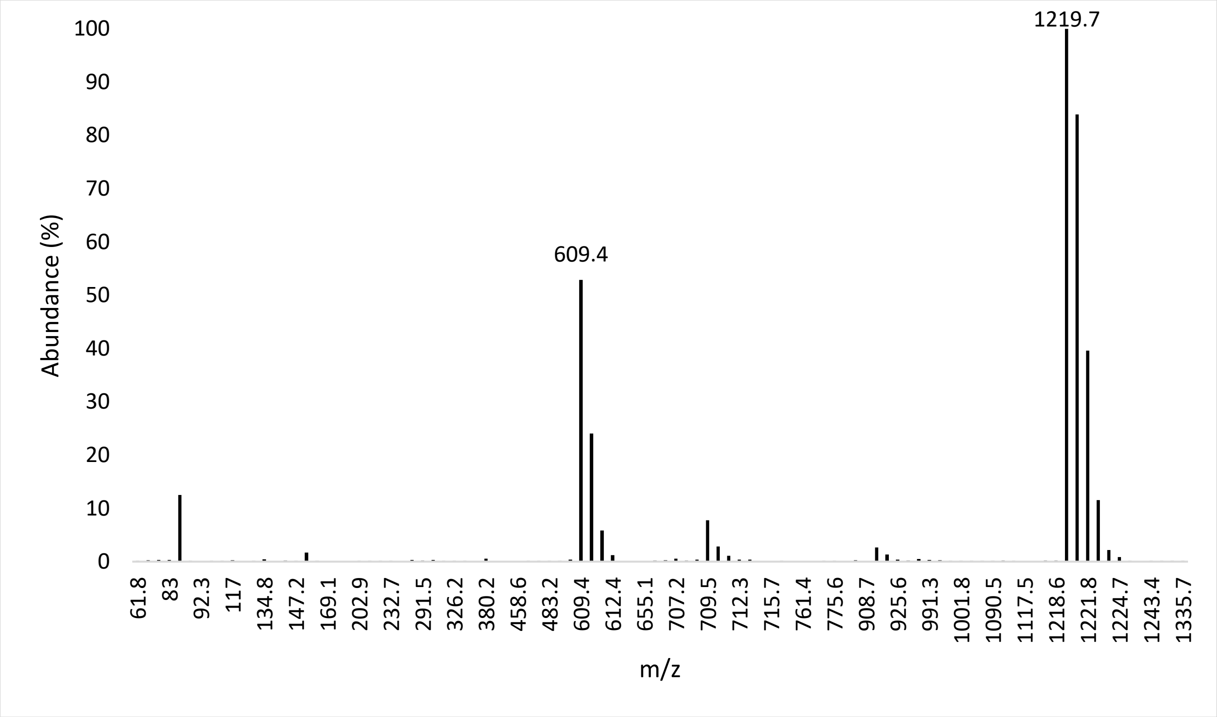

Figure S65. MS spectrum extracted from DA-PLA reaction product chromatogram at retention time 29.0 min. Labeled m/z signals represent 4PLA.

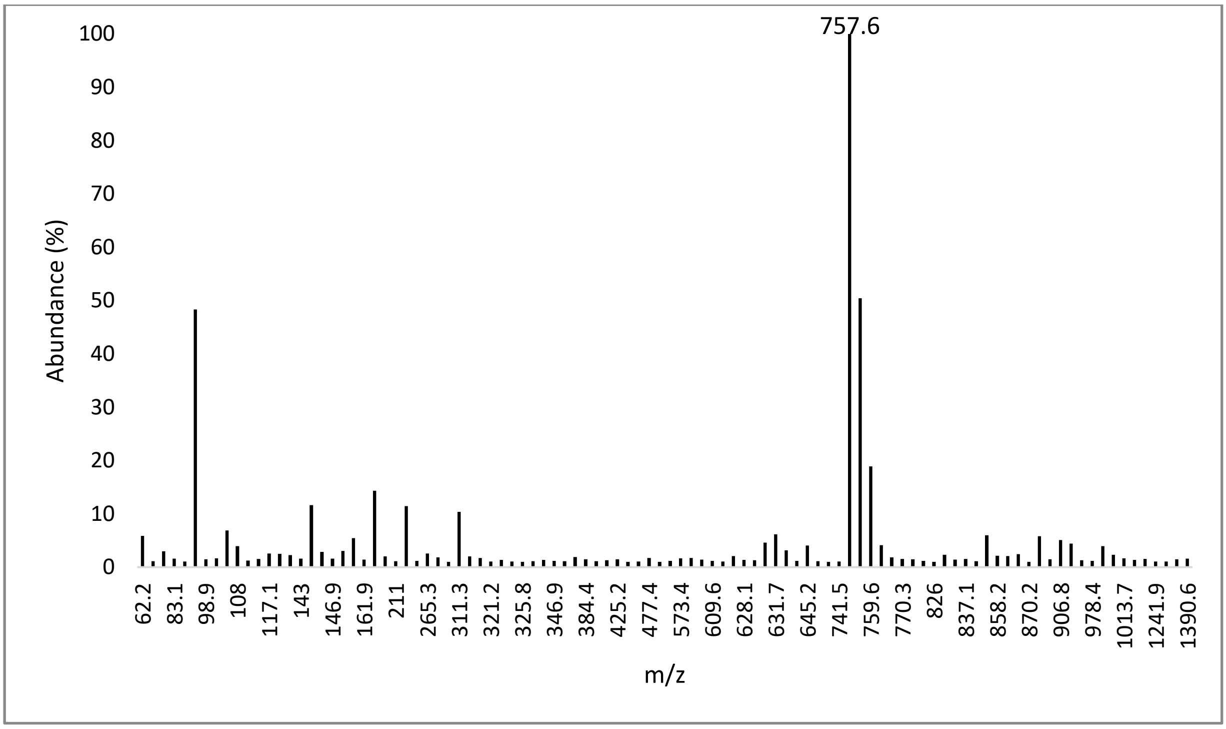

Figure S66. MS spectrum extracted from DA-PLA reaction product chromatogram at retention time 30.0 min. The labeled m/z signal represents 5PLA.

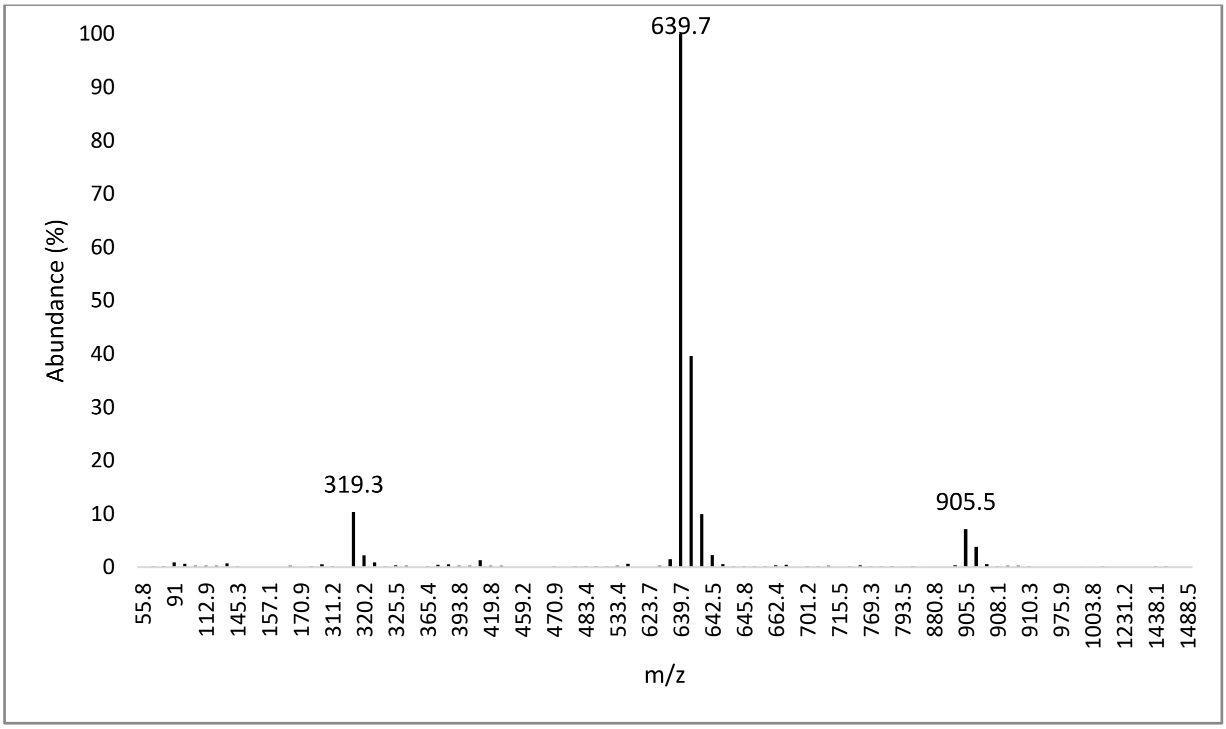

Figure S67. MS spectrum extracted from DA-PLA reaction product chromatogram at retention time 30.4 min. Labeled m/z signals represent 6PLA and 1PLA1DA.

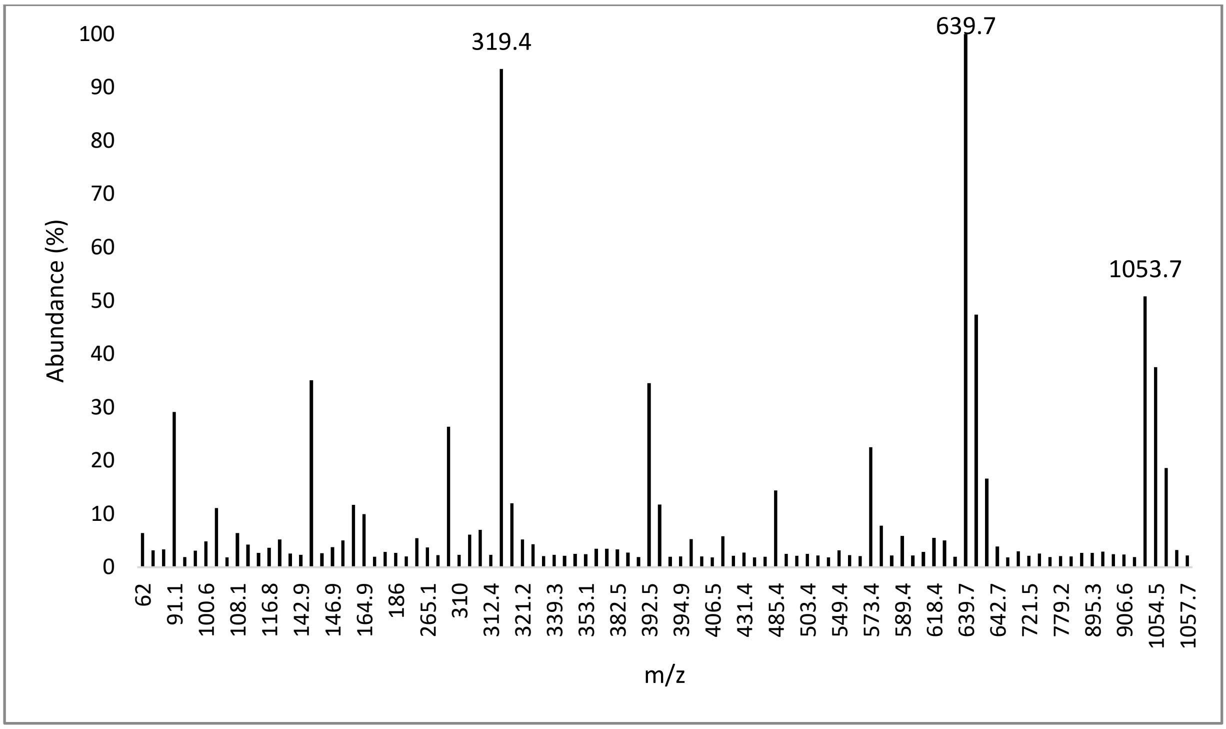

Figure S68. MS spectrum extracted from DA-PLA reaction product chromatogram at retention time 30.7 min. Labeled m/z signals represent 7PLA and 1PLA1DA.

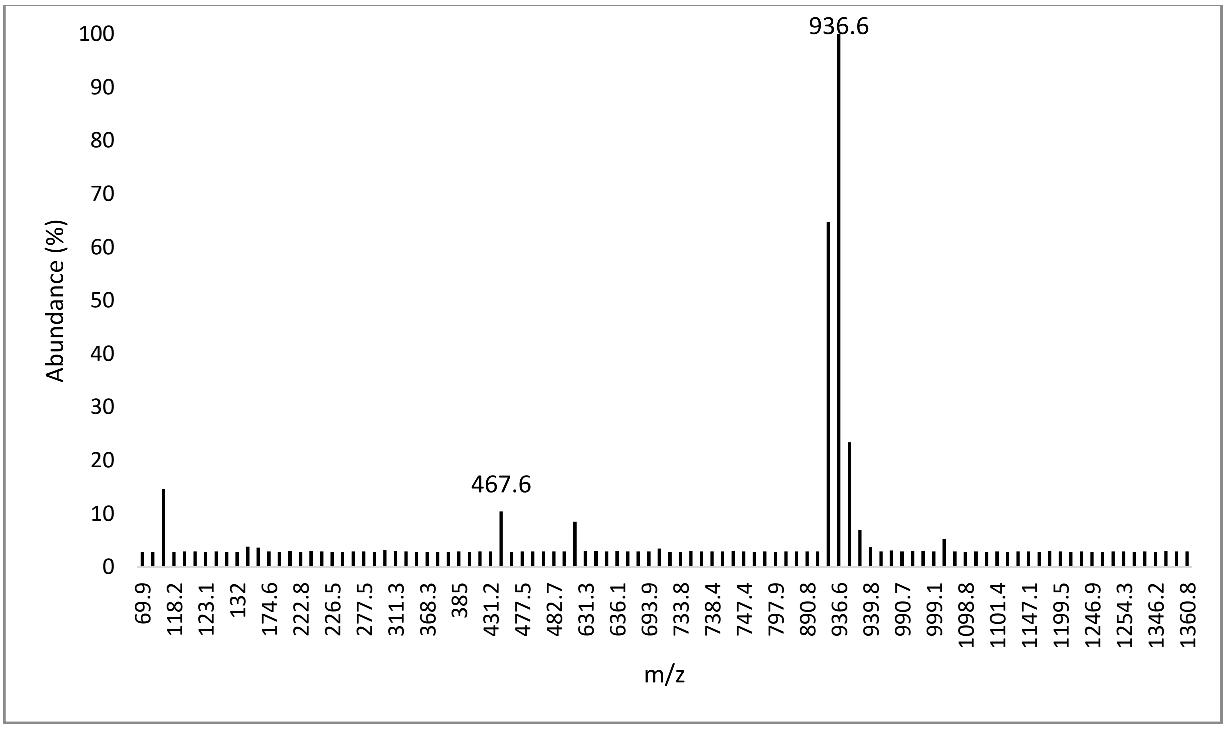

Figure S69. MS spectrum extracted from DA-PLA reaction product chromatogram at retention time 31.4 min. Labeled m/z signals represent 2PLA1DA.

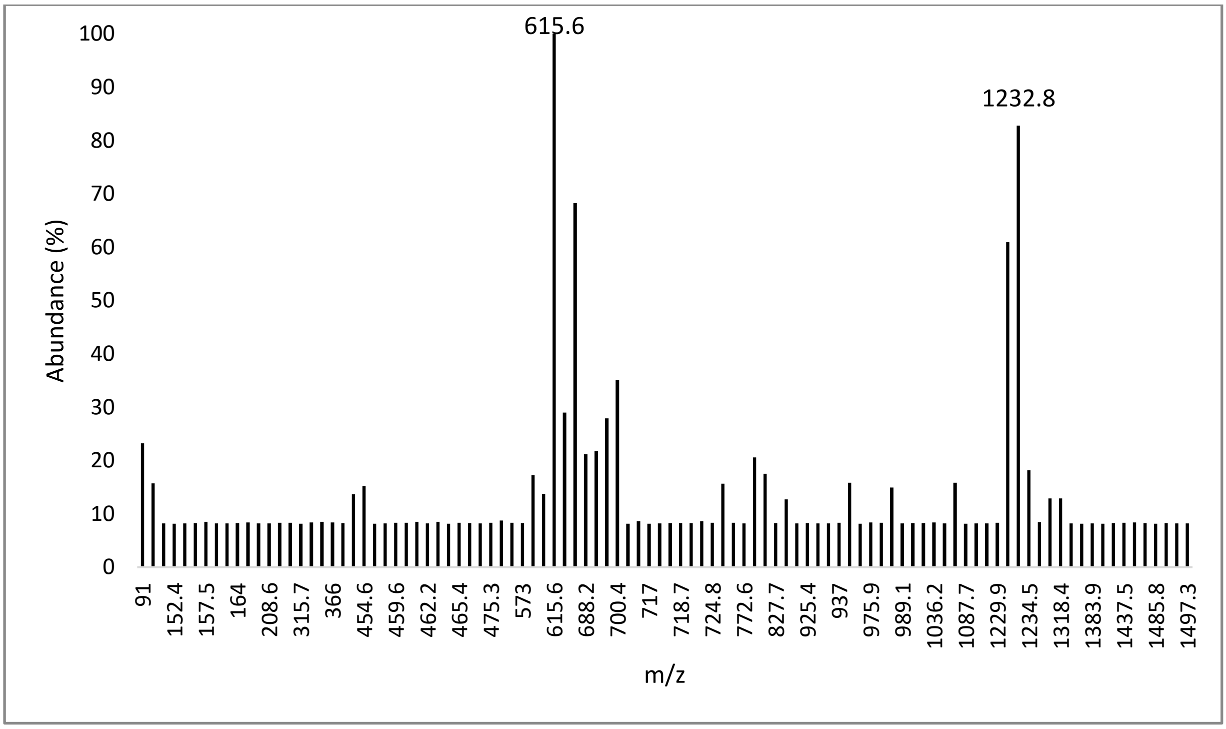

Figure S70. MS spectrum extracted from DA-PLA reaction product chromatogram at retention time 32.2 min. Labeled m/z signals represent 3PLA1DA.

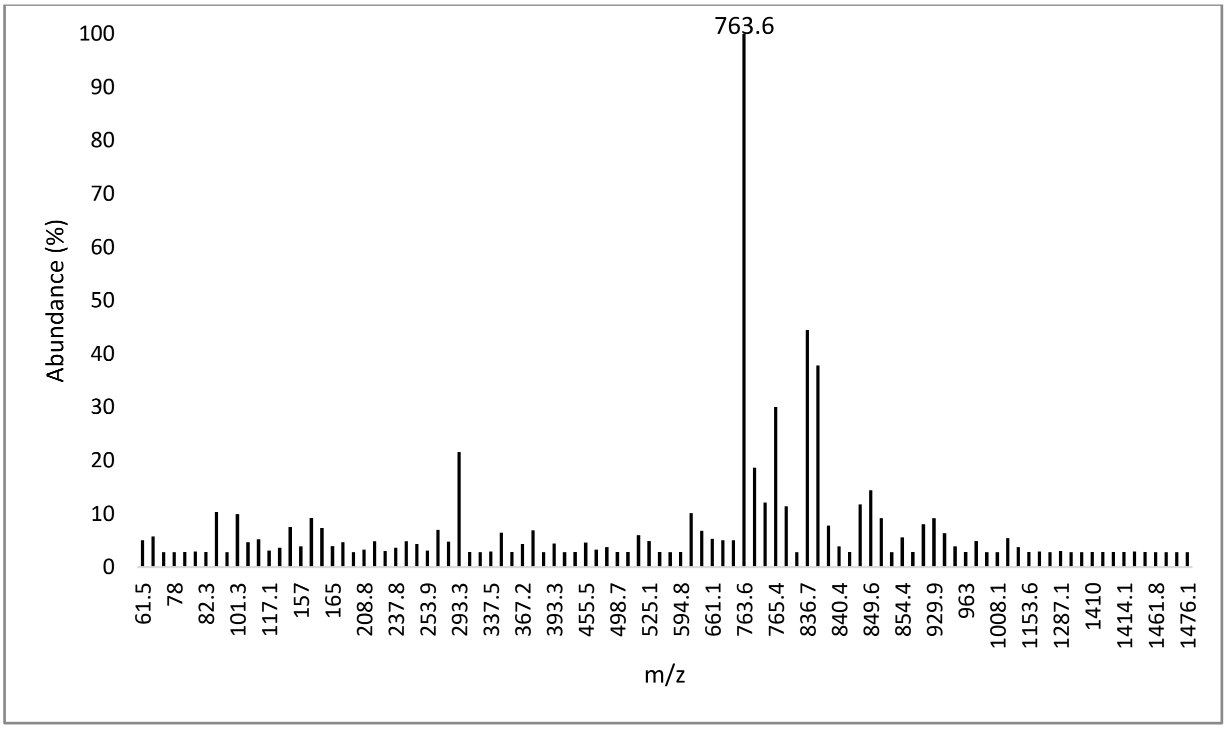

Figure S71. MS spectrum extracted from DA-PLA reaction product chromatogram at retention 33.0 min. The labeled m/z signal represents 4PLA1DA.

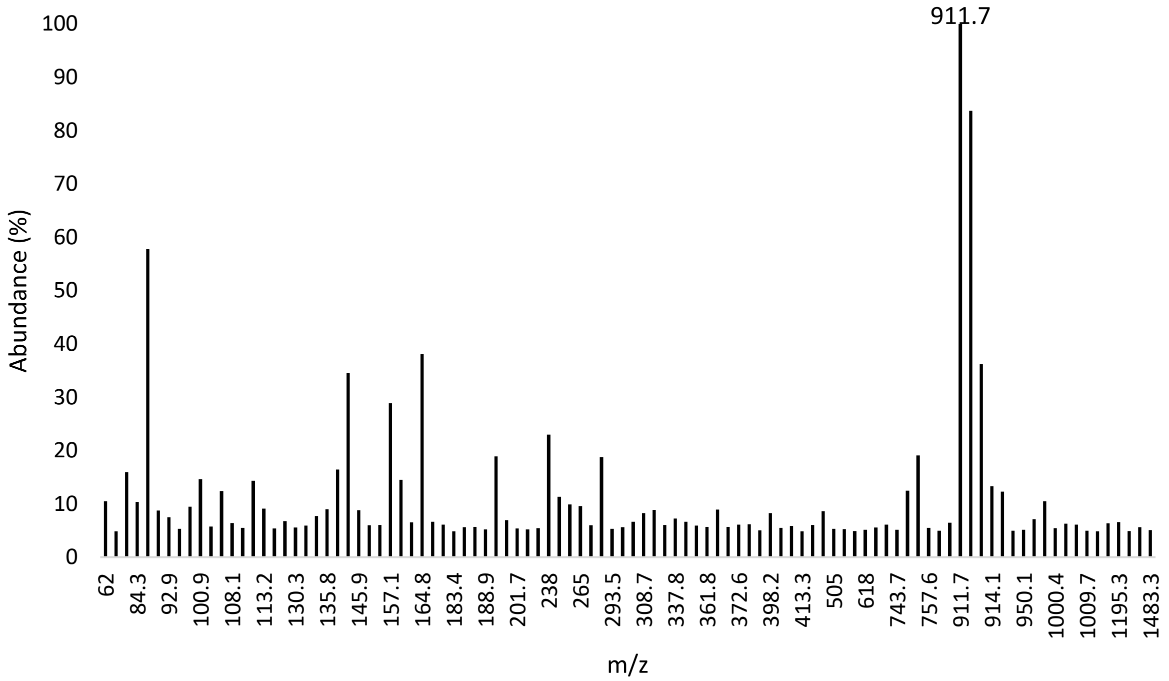

Figure S72. MS spectrum extracted from DA-PLA reaction product chromatogram at retention time 33.8 min. The labeled m/z signal represents 5PLA1DA.

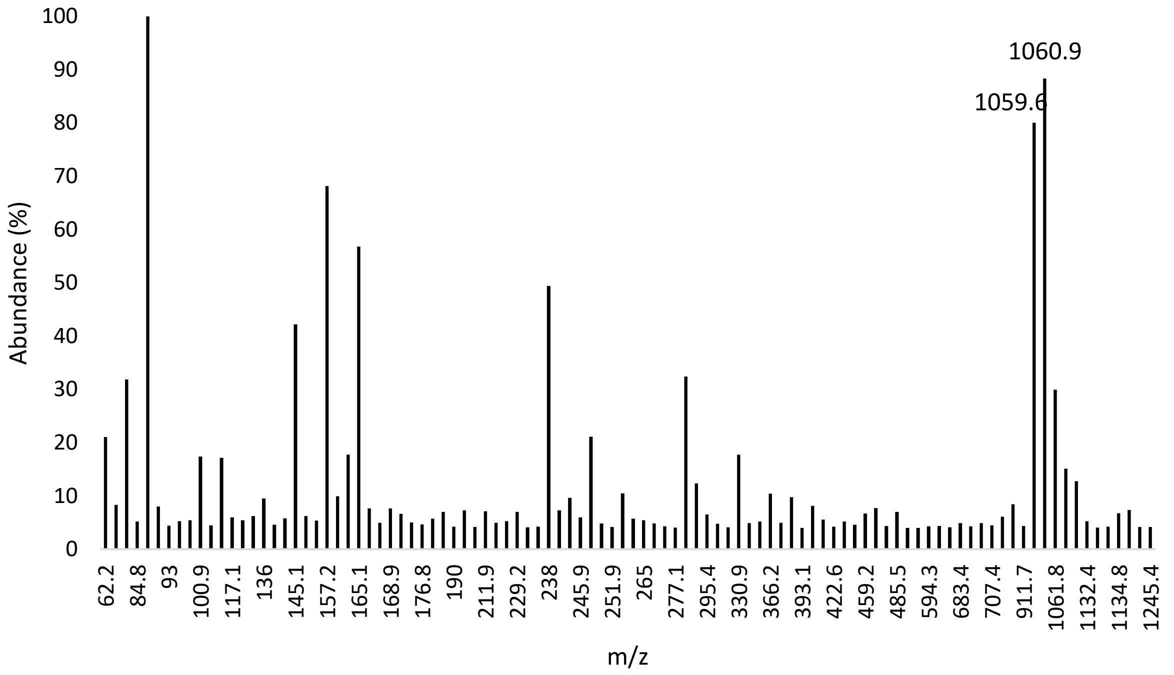

Figure S73. MS spectrum extracted from DA-PLA reaction product chromatogram at retention time 34.8 min. The labeled m/z signal represents 6PLA1DA.

Table S4. Identification of DA-LA reaction products. The detected products based on retention time and their corresponding m/z and ionization pattern as determined by LC-MS.

Retention time (min)	Compound	M (g/mol)	Corresponding m/z (-TIC)	Ionization pattern
**12.5**	2LA	162.1	161.1, 323.2	$[M-H]^-$, $[2M-H]^-$
**13.2**	2LA	162.1	161.2, 323.2	$[M-H]^-$, $[2M-H]^-$
**15.7**	3LA	234.2	233.1, 467.3	$[M-H]^-$, $[2M-H]^-$
**17.8**	4LA	306.3	305.1, 611.4	$[M-H]^-$, $[2M-H]^-$
**19.5**	5LA	378.3	377.2, 755.4	$[M-H]^-$, $[2M-H]^-$
**19.7**	5LA	378.3	377.2, 755.4	$[M-H]^-$, $[2M-H]^-$
**21.0**	6LA	450.4	449.3, 899.5	$[M-H]^-$, $[2M-H]^-$
**21.2**	6LA	450.4	449.2, 899.4	$[M-H]^-$, $[2M-H]^-$
**22.2**	7LA	522.4	521.3, 1043.7	$[M-H]^-$, $[2M-H]^-$
**22.4**	7LA	522.4	521.4, 1043.6	$[M-H]^-$, $[2M-H]^-$
**23.3**	8LA	594.5	593.4, 1187.8	$[M-H]^-$, $[2M-H]^-$
**23.5**	8LA	594.5	593.4, 1187.6	$[M-H]^-$, $[2M-H]^-$
**24.4**	9LA	666.6	665.5, 1331.8	$[M-H]^-$, $[2M-H]^-$
**25.2**	10LA	738.6	737.5, 1475.9	$[M-H]^-$, $[2M-H]^-$
**25.9**	11LA	810.7	809.4	$[M-H]^-$
**26.6**	12LA	882.7	881.6	$[M-H]^-$
**27.2**	13LA	954.8	953.6	$[M-H]^-$
**29.1**	1LA1DA	244.3	243.3, 487.5	$[M-H]^-$, $[2M-H]^-$

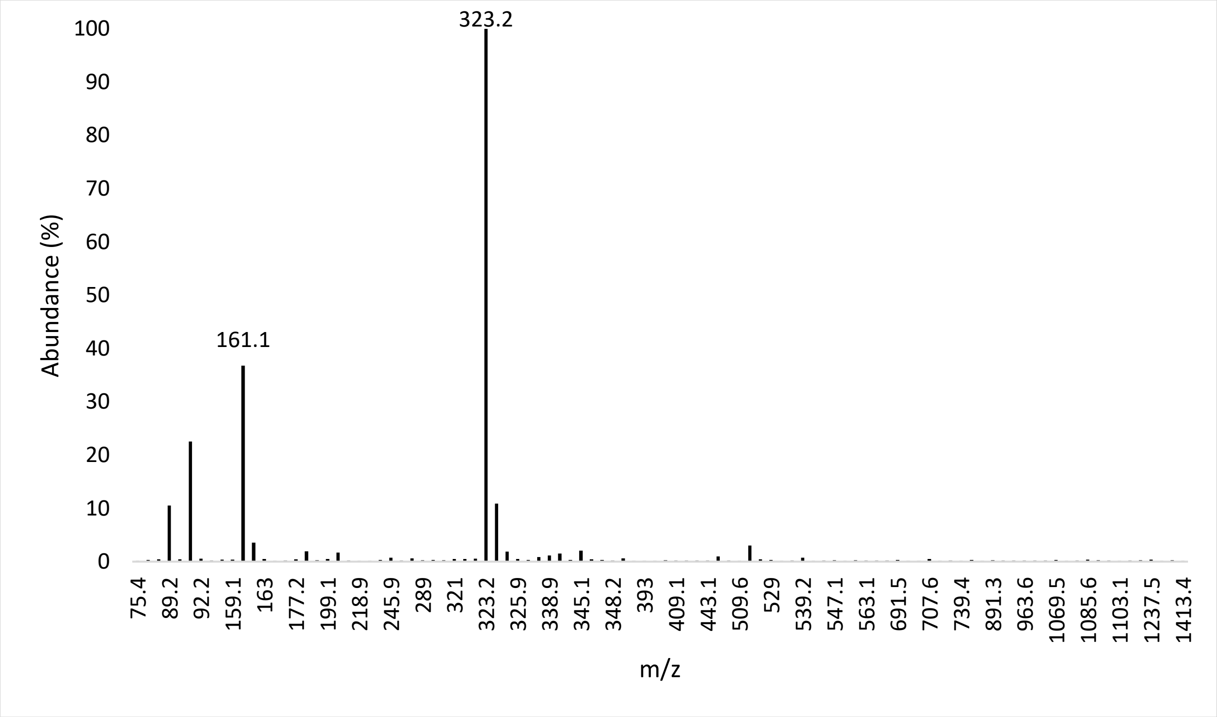

Figure S74. MS spectrum extracted from DA-LA reaction product chromatogram at retention time 12.5 min. Labeled m/z signals represent 2LA.

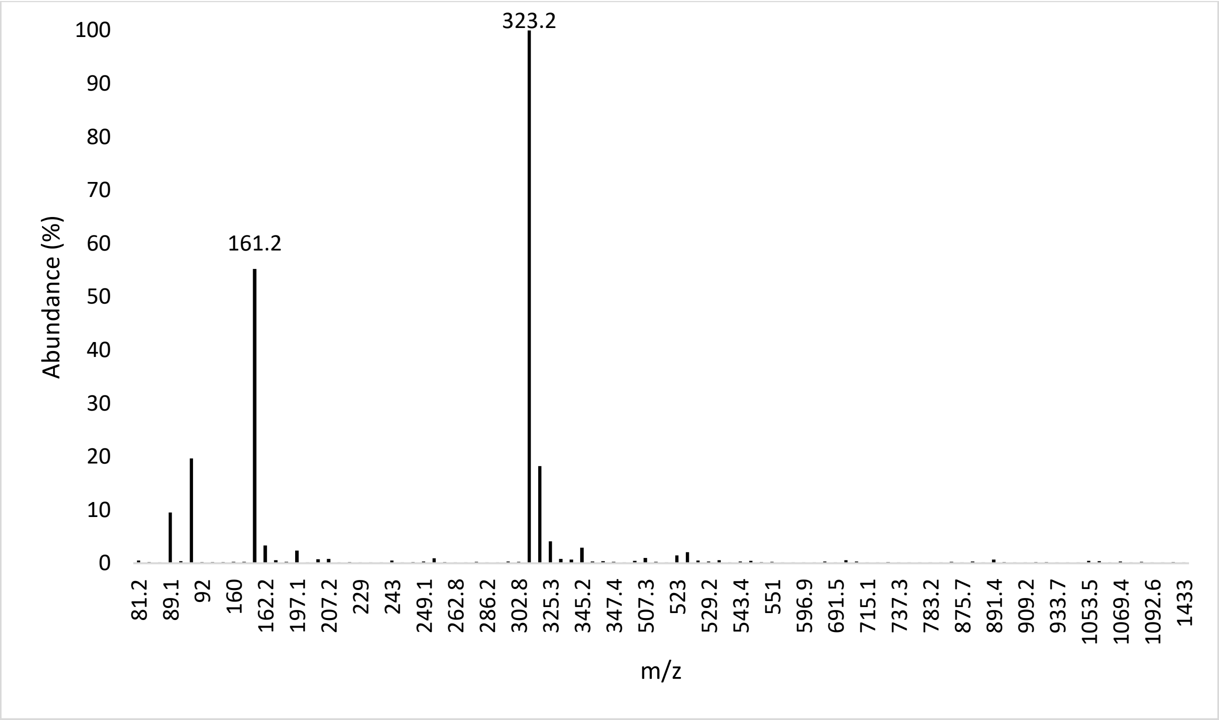

Figure S75. MS spectrum extracted from DA-LA reaction product chromatogram at retention time 13.2 min. Labeled m/z signals represent 2LA.

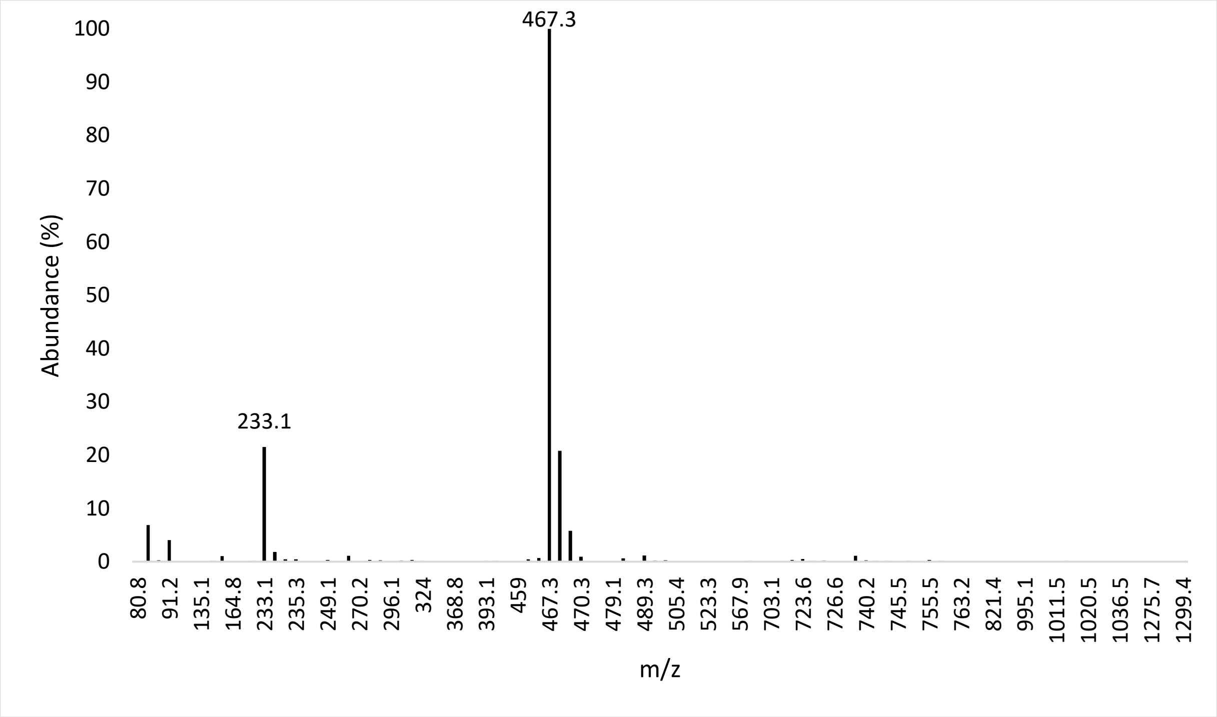

Figure S76. MS spectrum extracted from DA-LA reaction product chromatogram at retention time 15.7 min. Labeled m/z signals represent 3LA.

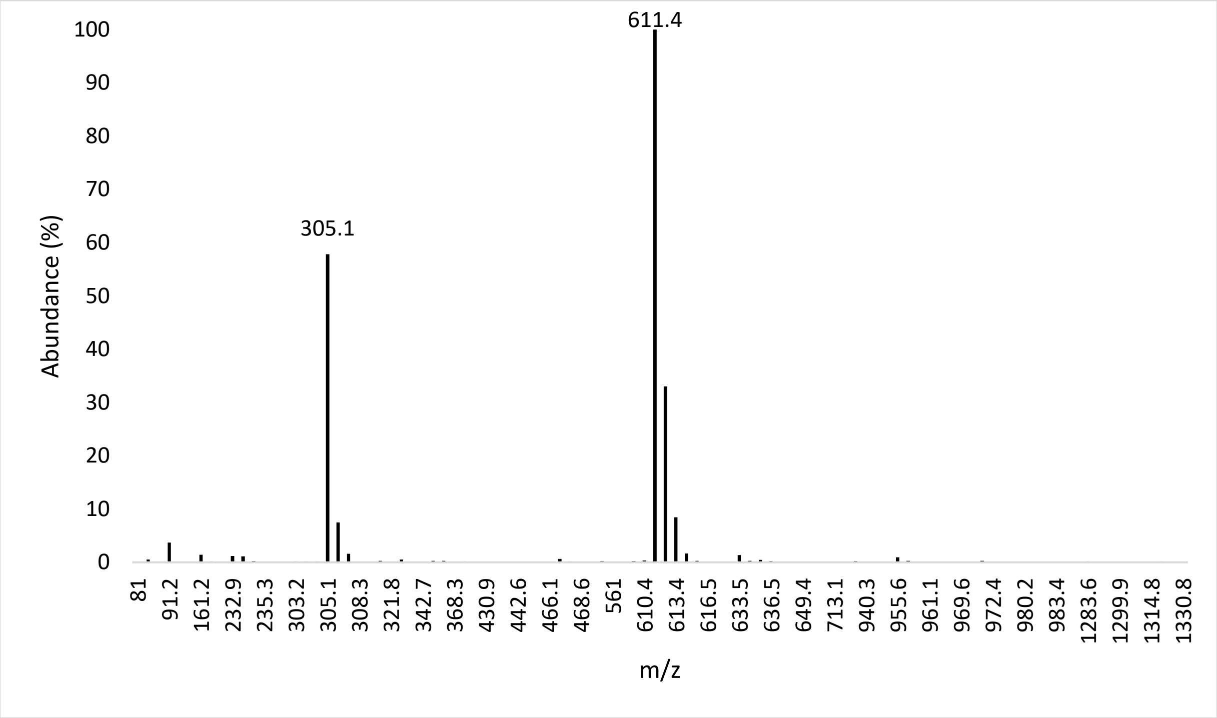

Figure S77. MS spectrum extracted from DA-LA reaction product chromatogram at retention time 17.8 min. Labeled m/z signals represent 4LA.

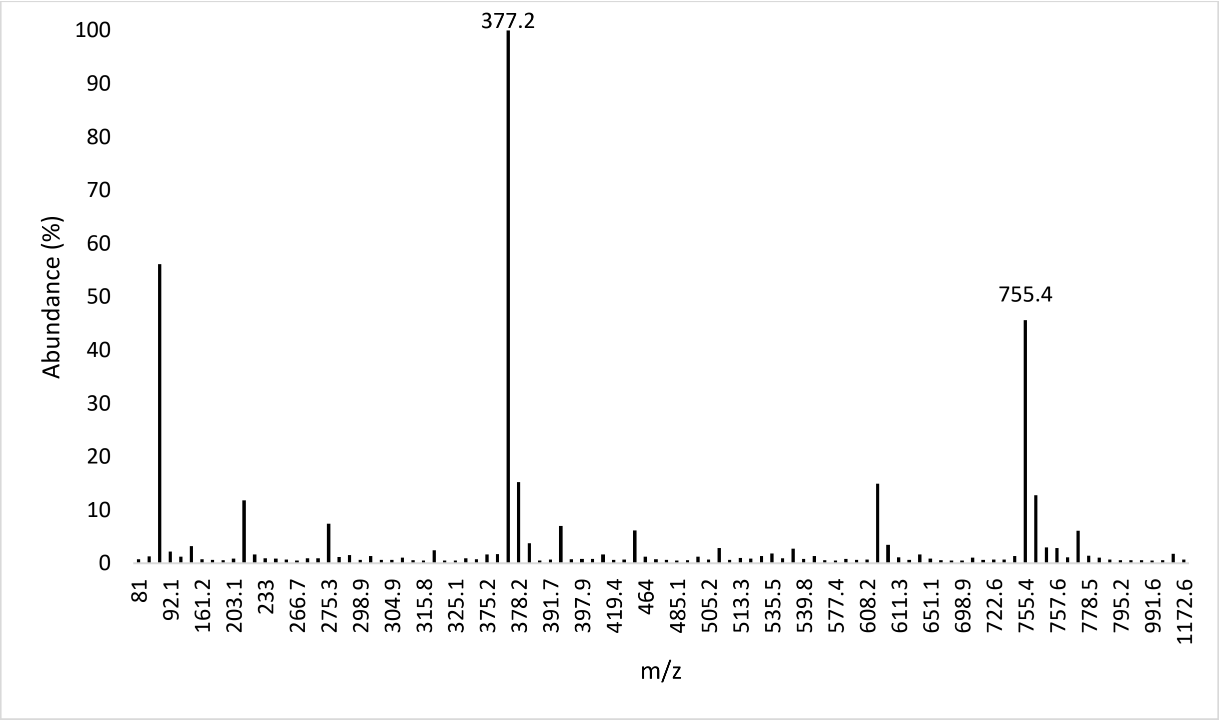

Figure S78. MS spectrum extracted from DA-LA reaction product chromatogram at retention time 19.5 min. Labeled m/z signals represent 5LA.

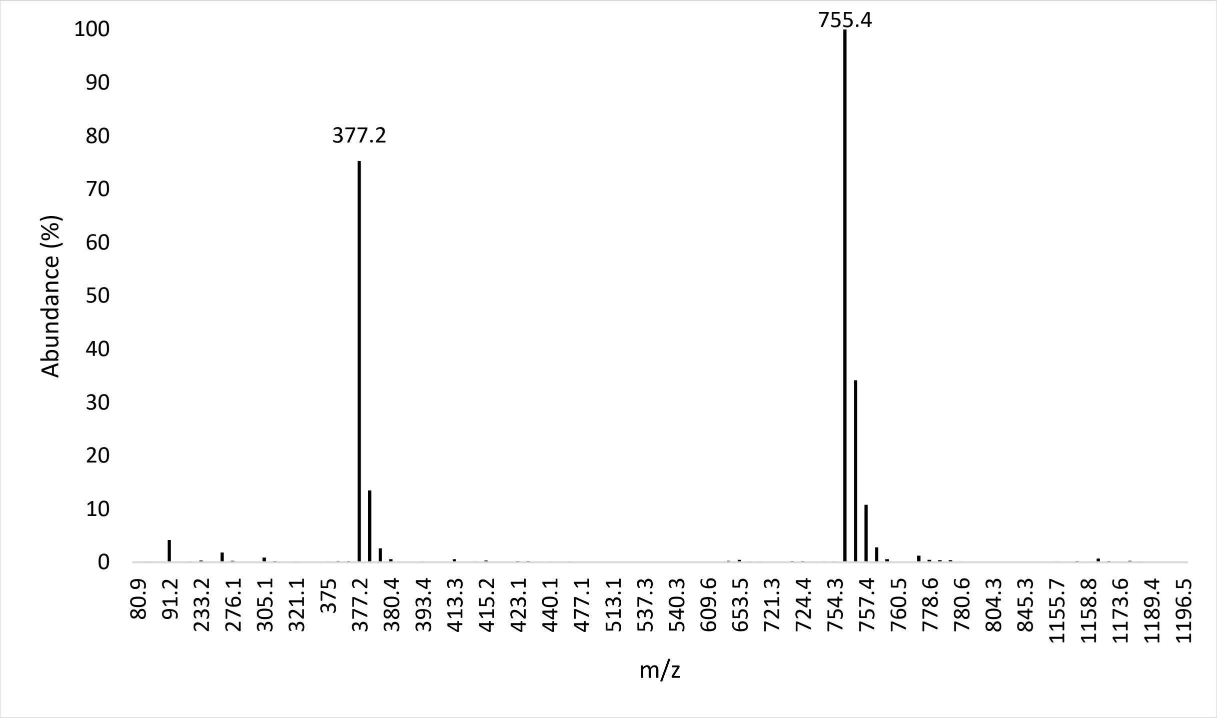

Figure S79. MS spectrum extracted from DA-LA reaction product chromatogram at retention time 19.7 min. Labeled m/z signals represent 5LA.

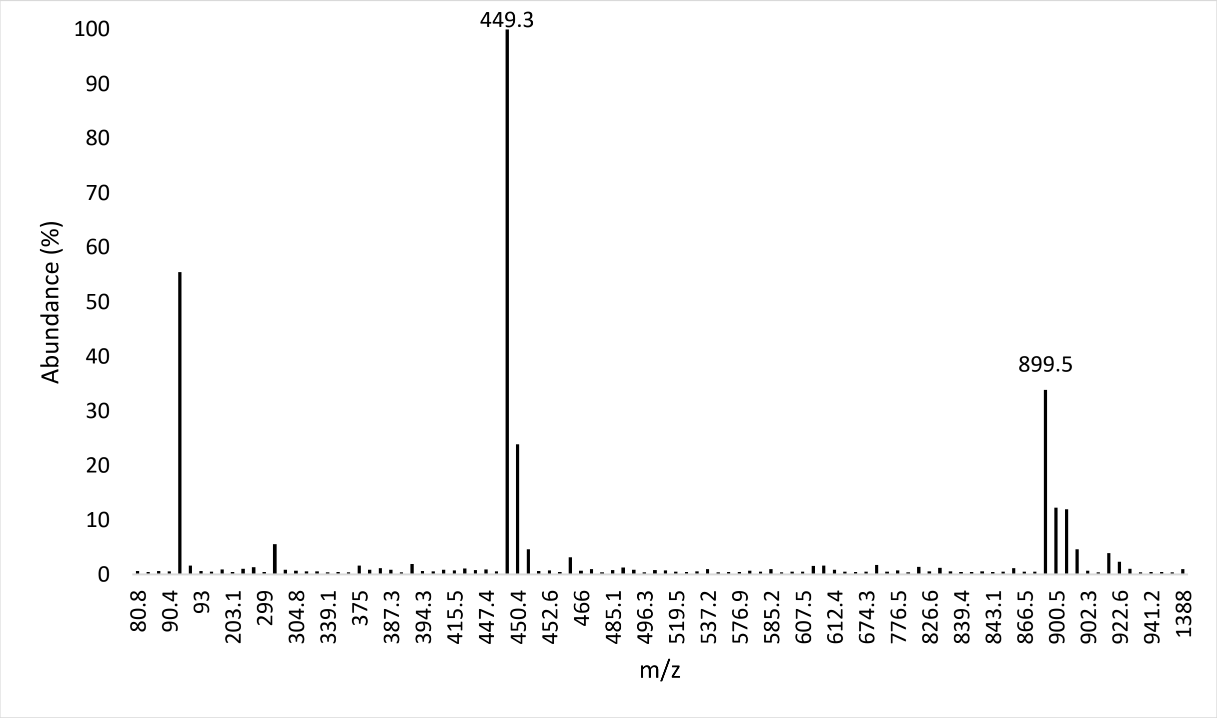

Figure S80. MS spectrum extracted from DA-LA reaction product chromatogram at retention time 21.0 min. Labeled m/z signals represent 6LA.

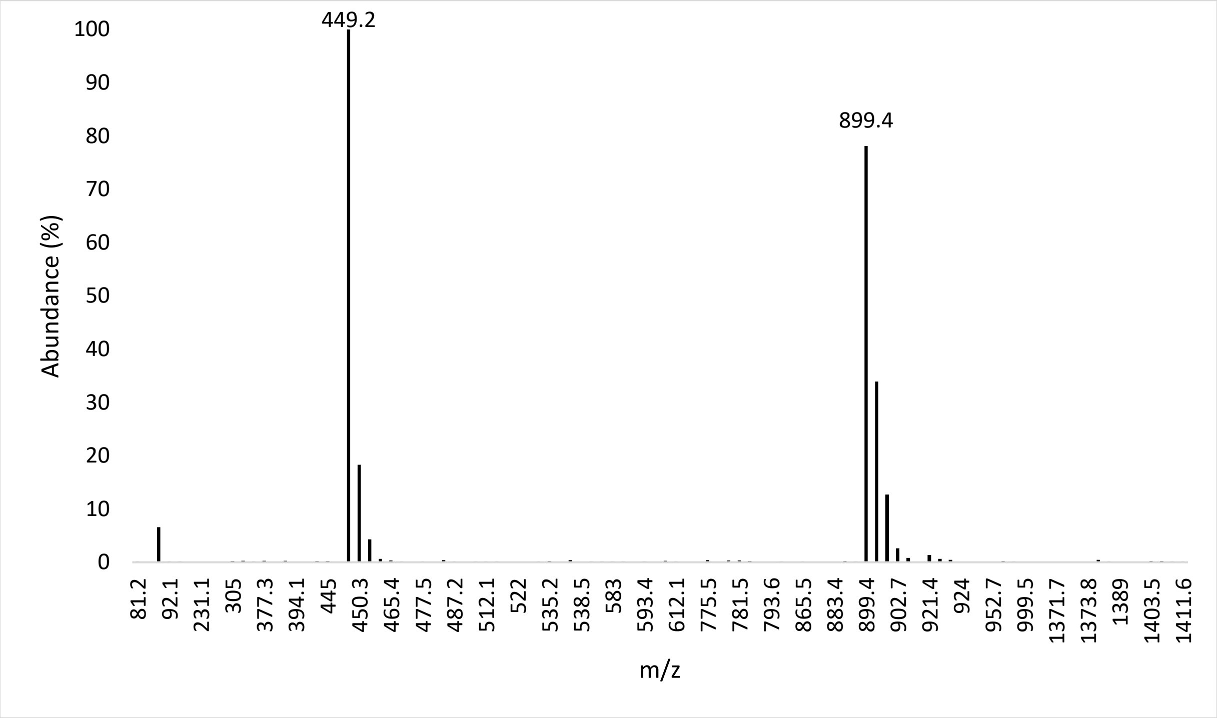

Figure S81. MS spectrum extracted from DA-LA reaction product chromatogram at retention time 21.2 min. Labeled m/z signals represent 6LA.

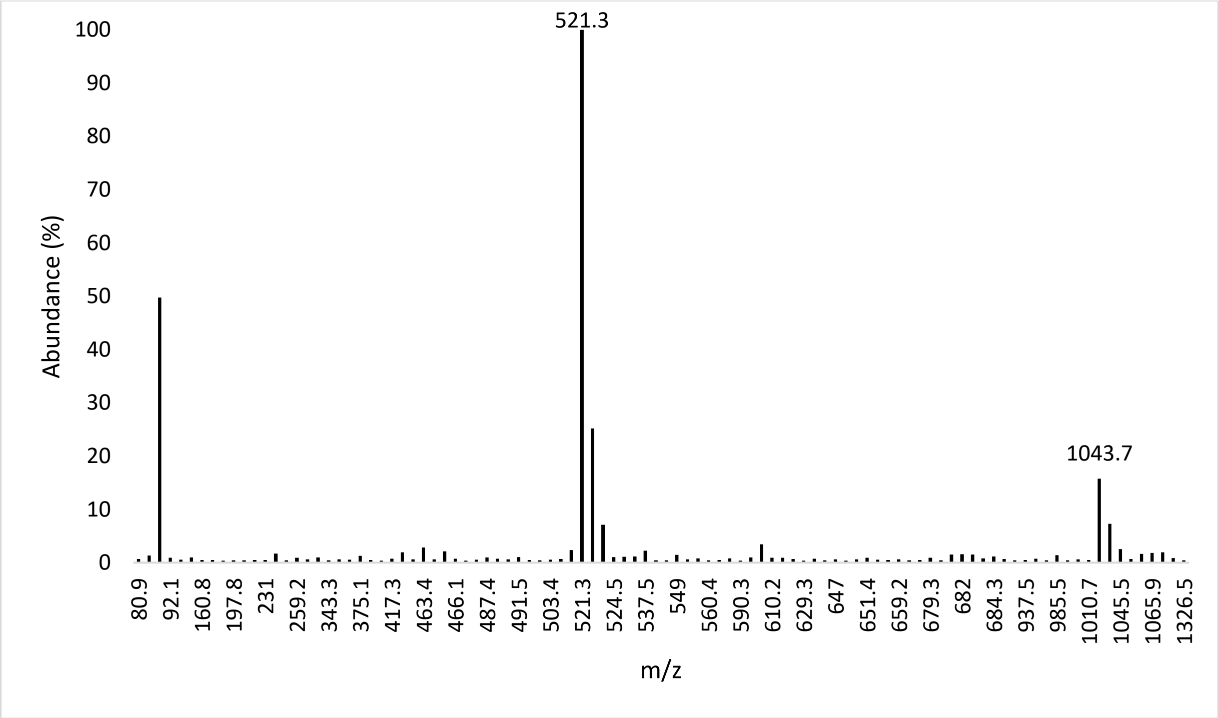

Figure S82. MS spectrum extracted from DA-LA reaction product chromatogram at retention time 22.2 min. Labeled m/z signals represent 7LA.

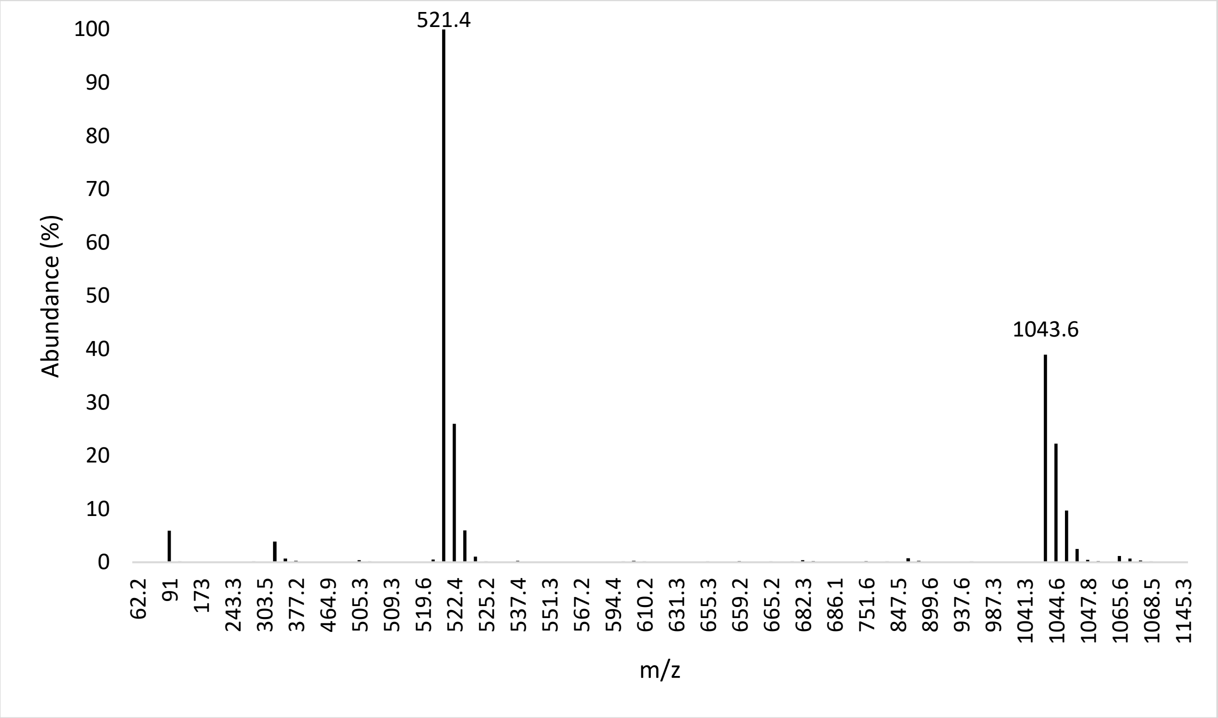

Figure S83. MS spectrum extracted from DA-LA reaction product chromatogram at retention time 22.4 min. Labeled m/z signals represent 7LA.

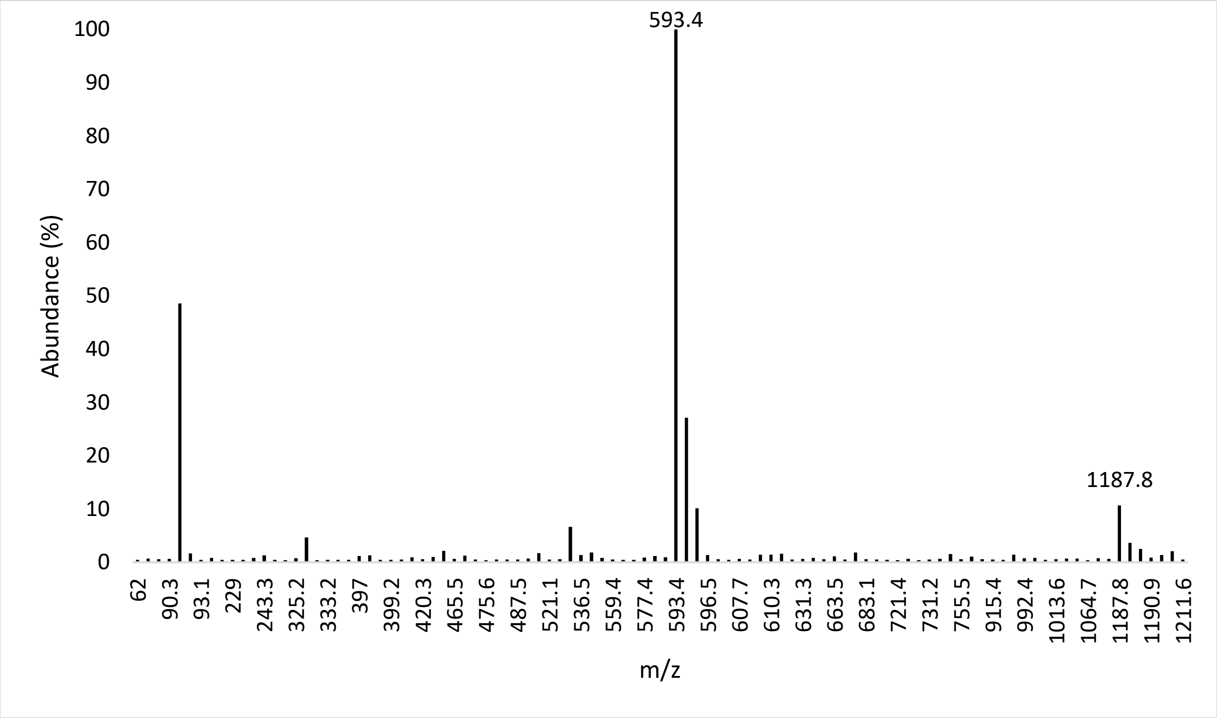

Figure S84. MS spectrum extracted from DA-LA reaction product chromatogram at retention time 23.3 min. Labeled m/z signals represent 8LA.

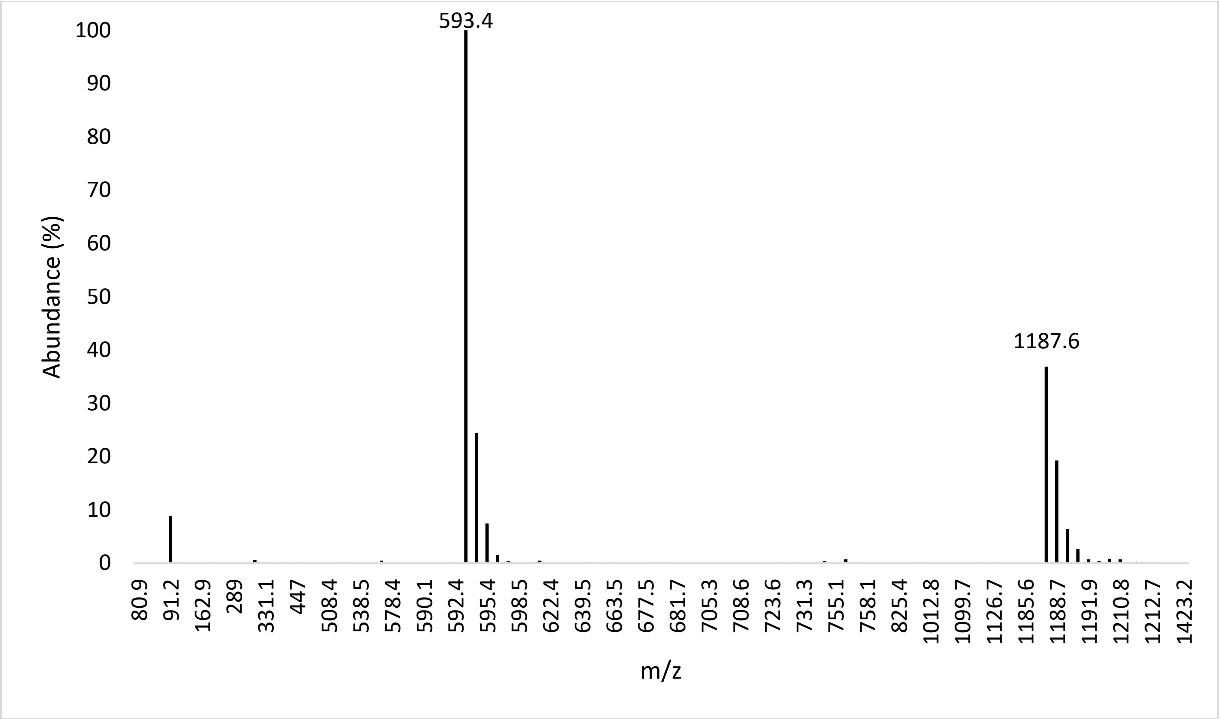

Figure S85. MS spectrum extracted from DA-LA reaction product chromatogram at retention time 23.5 min. Labeled m/z signals represent 8LA.

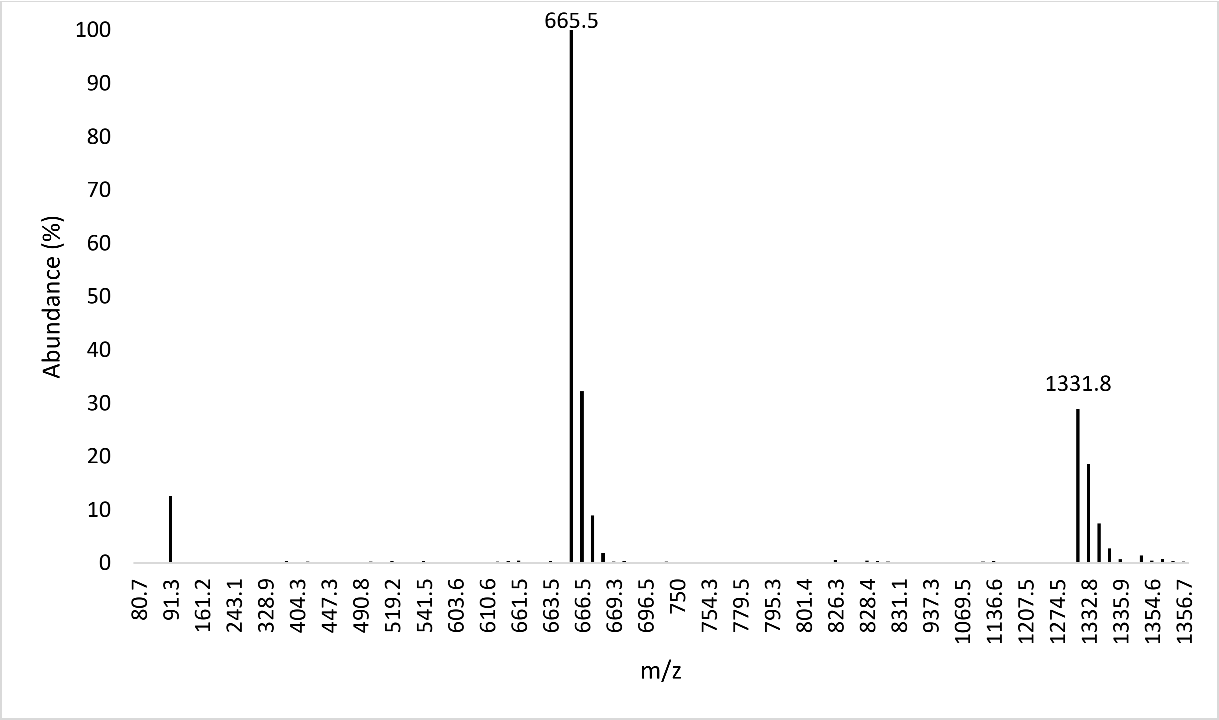

Figure S86. MS spectrum extracted from DA-LA reaction product chromatogram at retention time 24.4 min. Labeled m/z signals represent 9LA.

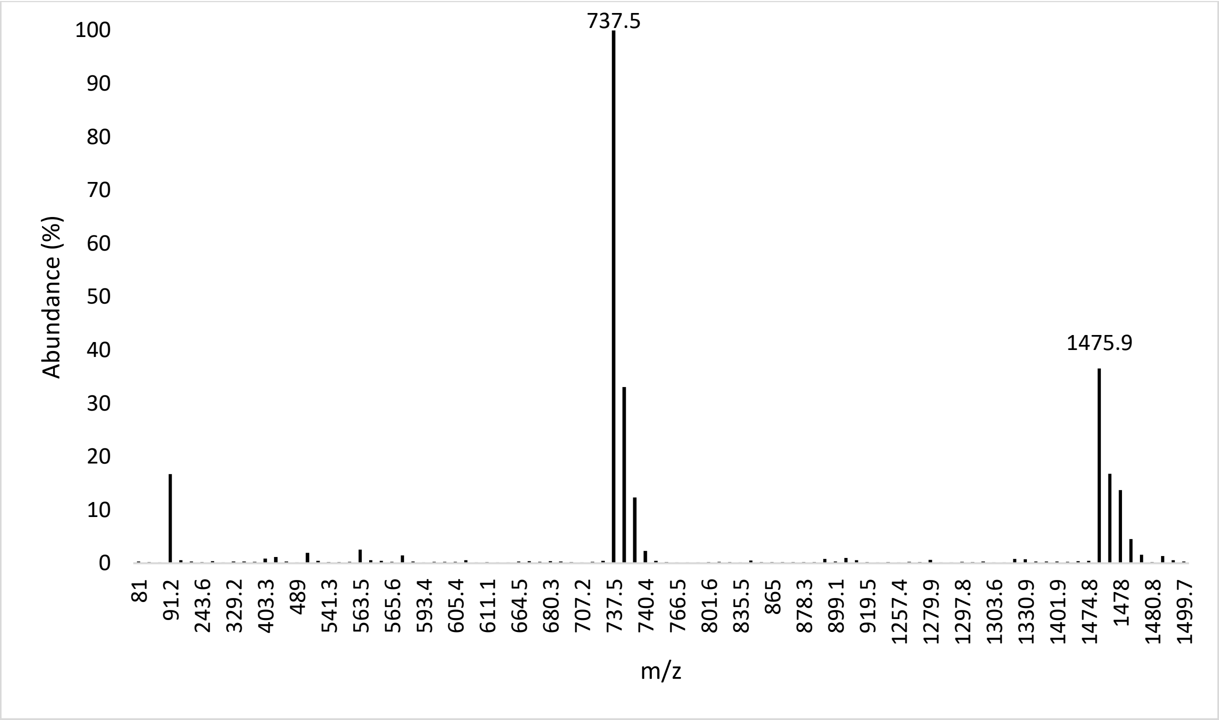

Figure S87. MS spectrum extracted from DA-LA reaction product chromatogram at retention time 25.2 min. Labeled m/z signals represent 10LA.

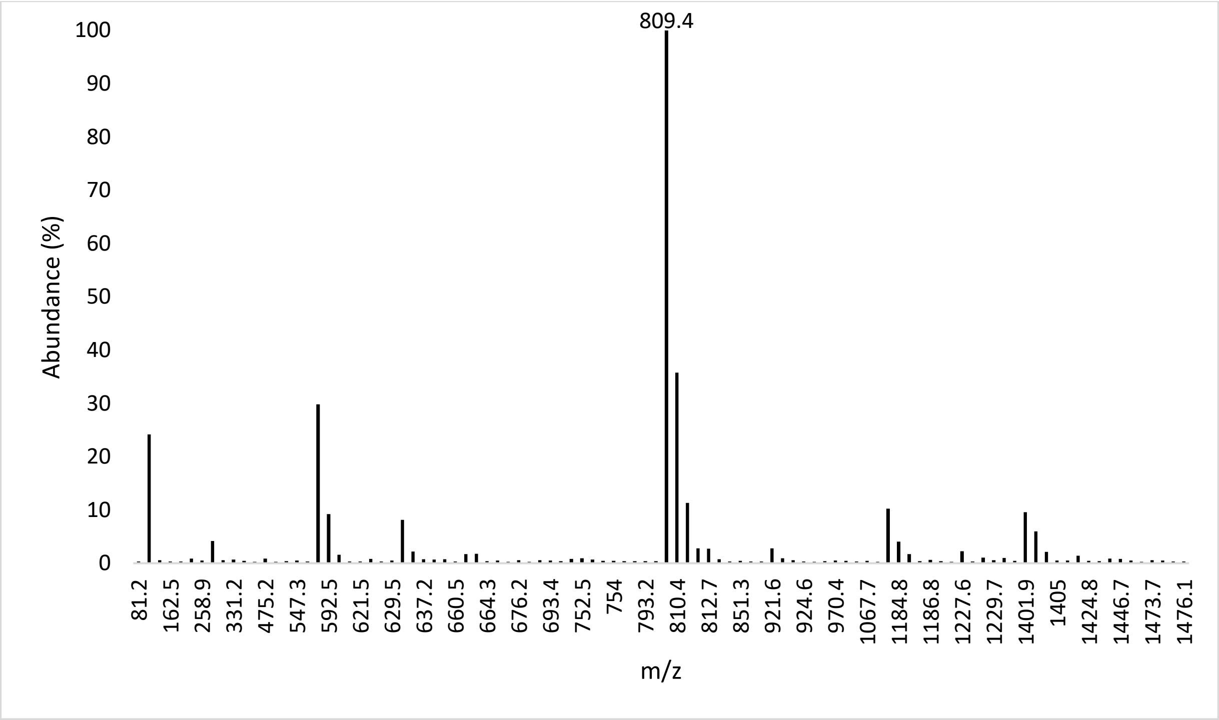

Figure S88. MS spectrum extracted from DA-LA reaction product chromatogram at retention time 25.9 min. The labeled m/z signal represents 11LA.

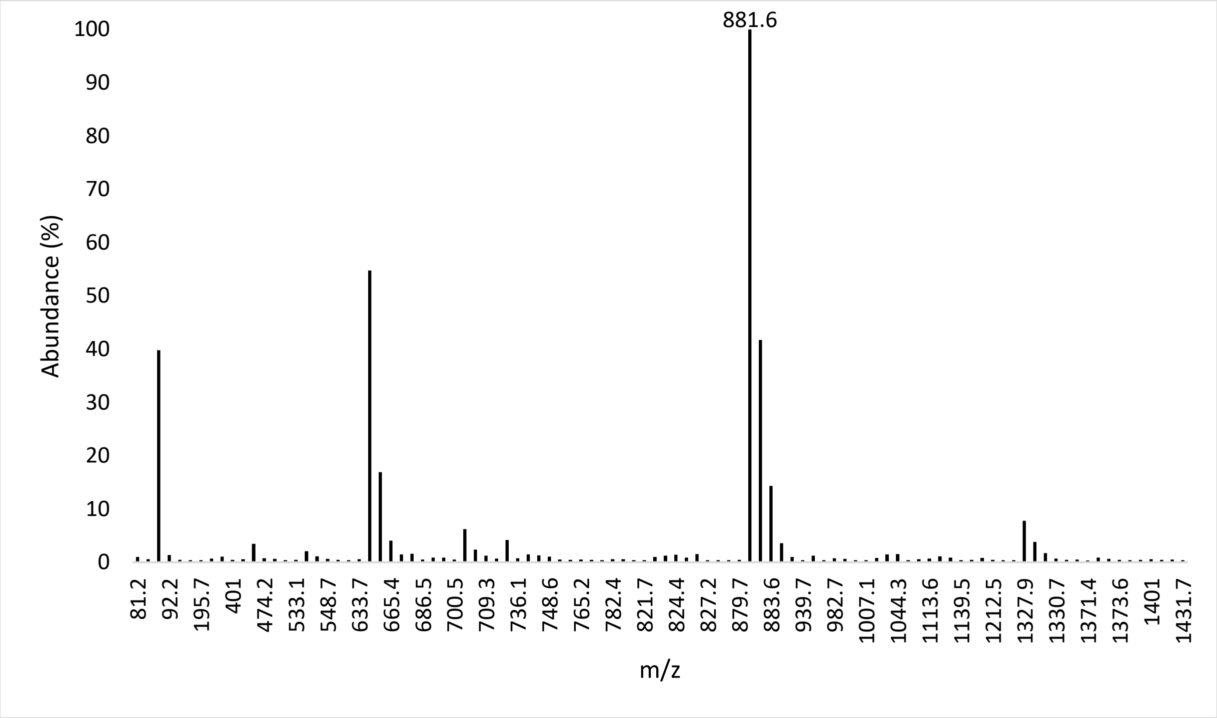

Figure S89. MS spectrum extracted from DA-LA reaction product chromatogram at retention time 26.6 min. The labeled m/z signal represents 12LA.

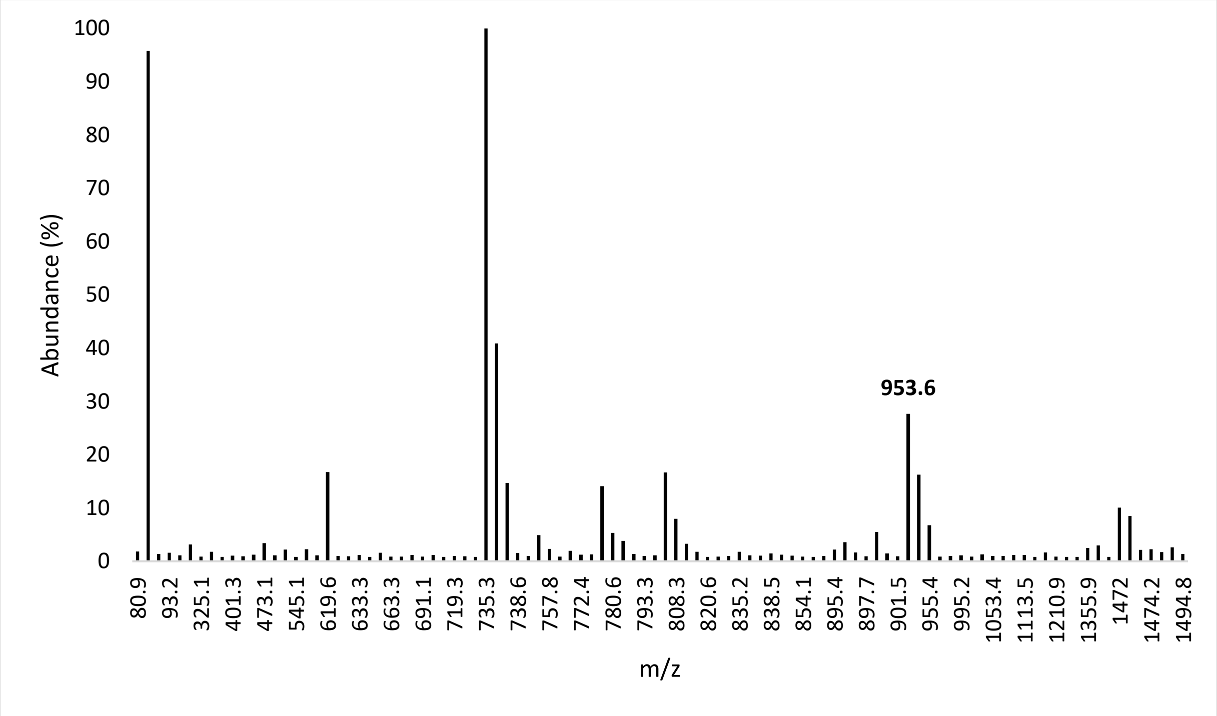

Figure S90. MS spectrum extracted from DA-LA reaction product chromatogram at retention time 27.2 min. The labeled m/z signal represents 13LA.

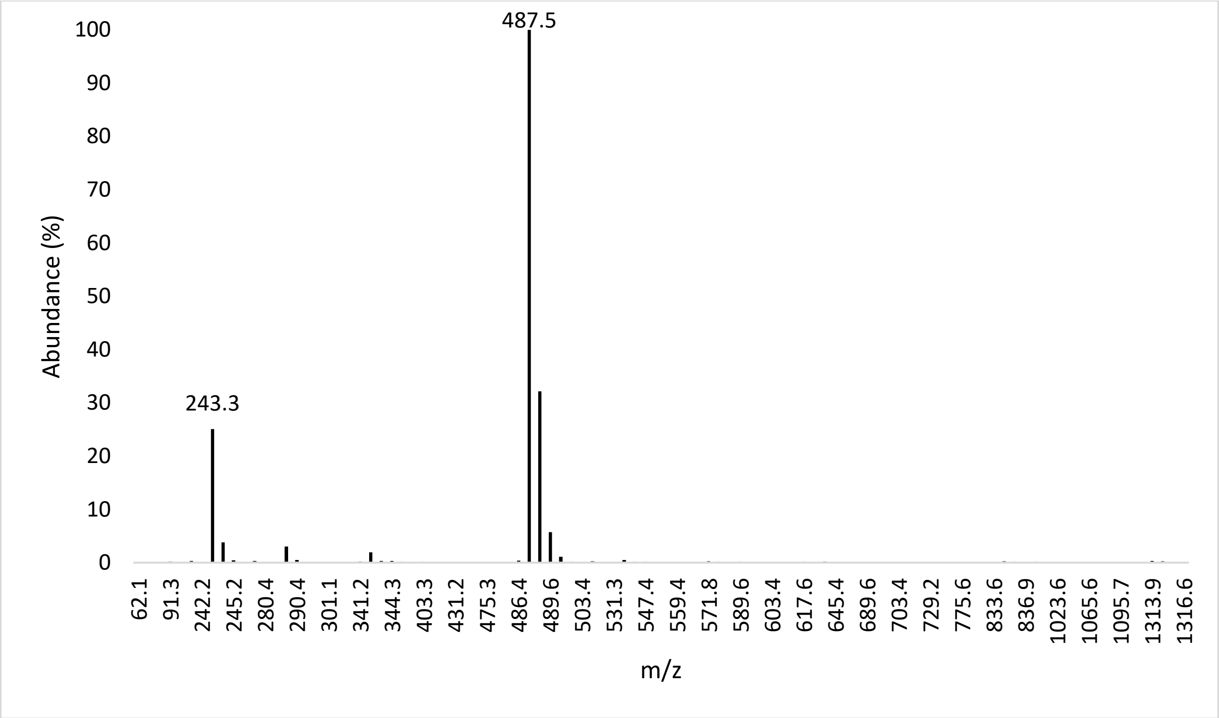

Figure S91. MS spectrum extracted from DA-LA reaction product chromatogram at retention time 29.1 min. Labeled m/z signals represent 1LA1DA.

Table S5. Identification of DA-LA reaction products. The detected products based on retention time and their corresponding m/z and ionization pattern as determined by optimized LC-MS. method

Retention time (min)	Compound	M (g/mol)	Corresponding m/z (-TIC)	Ionization pattern
**10.0**	1LA1DA	244.3	243.2, 487.5	$[M-H]^-$, $[2M-H]^-$
**10.5**	2LA1DA	316.4	315.2, 631.5	$[M-H]^-$, $[2M-H]^-$
**11.0**	3LA1DA	388.4	387.3, 775.6	$[M-H]^-$, $[2M-H]^-$
**11.4**	4LA1DA	460.5	459.4, 919.6	$[M-H]^-$, $[2M-H]^-$
**11.8**	5LA1DA	532.6	531.5, 1063.8	$[M-H]^-$, $[2M-H]^-$
**12.2**	6LA1DA	604.6	603.4, 1207.8	$[M-H]^-$, $[2M-H]^-$
**12.5**	7LA1DA	676.7	675.5, 1351.9	$[M-H]^-$, $[2M-H]^-$
**12.8**	8LA1DA	748.7	747.5, 1496.1	$[M-H]^-$, $[2M-H]^-$
**13.1**	9LA1DA	820.8	819.6	$[M-H]^-$
**13.4**	10LA1DA	892.9	891.6	$[M-H]^-$
**13.7**	11LA1DA	964.9	963.6	$[M-H]^-$
**13.9**	12LA1DA	1037.0	1035.7	$[M-H]^-$

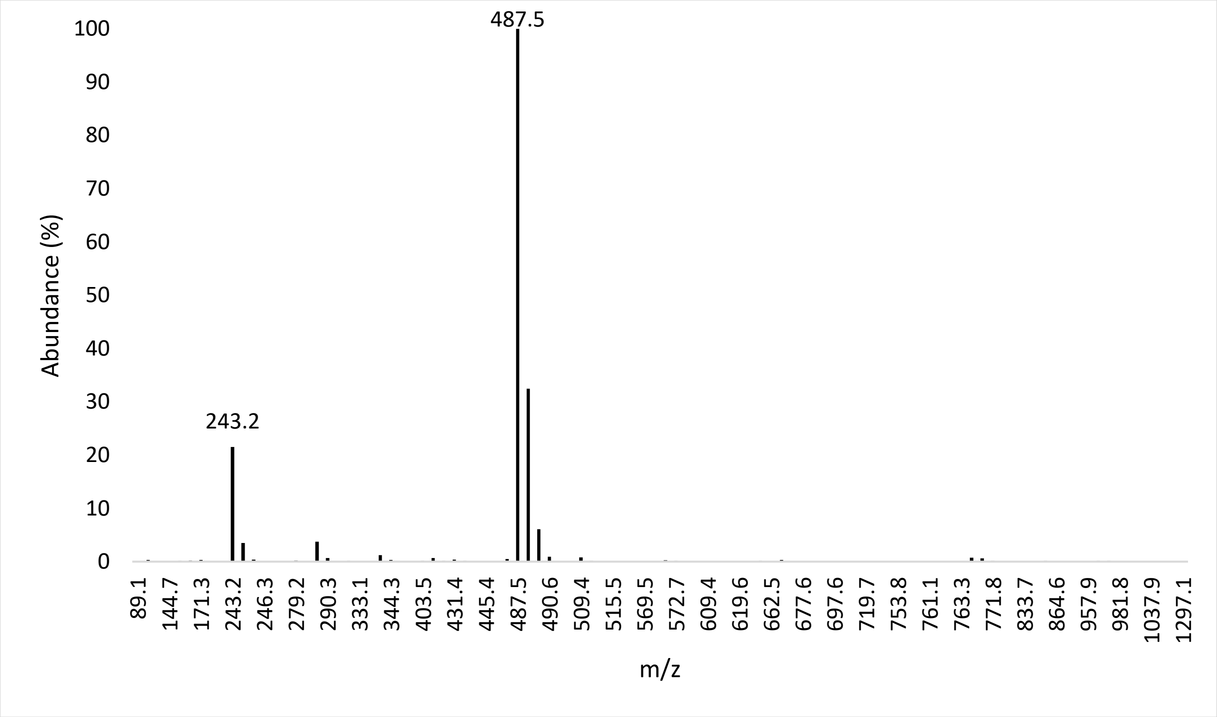

Figure S92. MS spectrum extracted from DA-LA reaction product optimized chromatogram at retention time 10.0 min. Labeled m/z signals represent 1LA1DA.

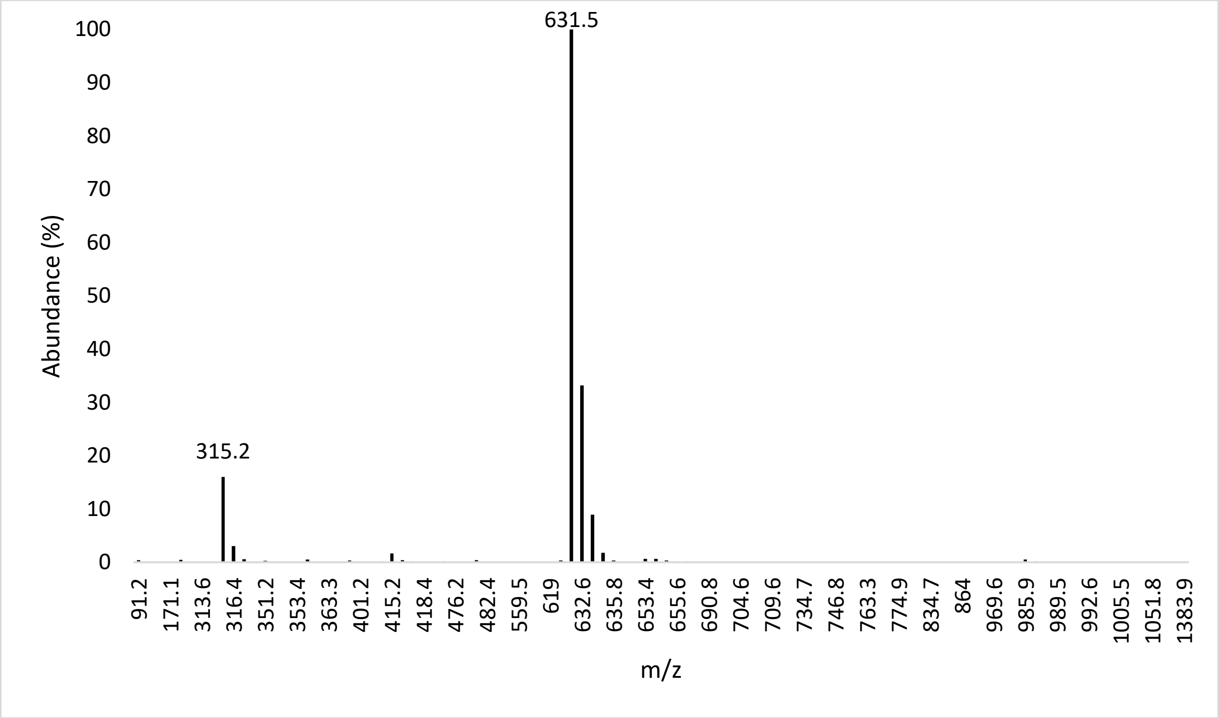

Figure S93. MS spectrum extracted from DA-LA reaction product optimized chromatogram at retention time 10.5 min. Labeled m/z signals represent 2LA1DA.

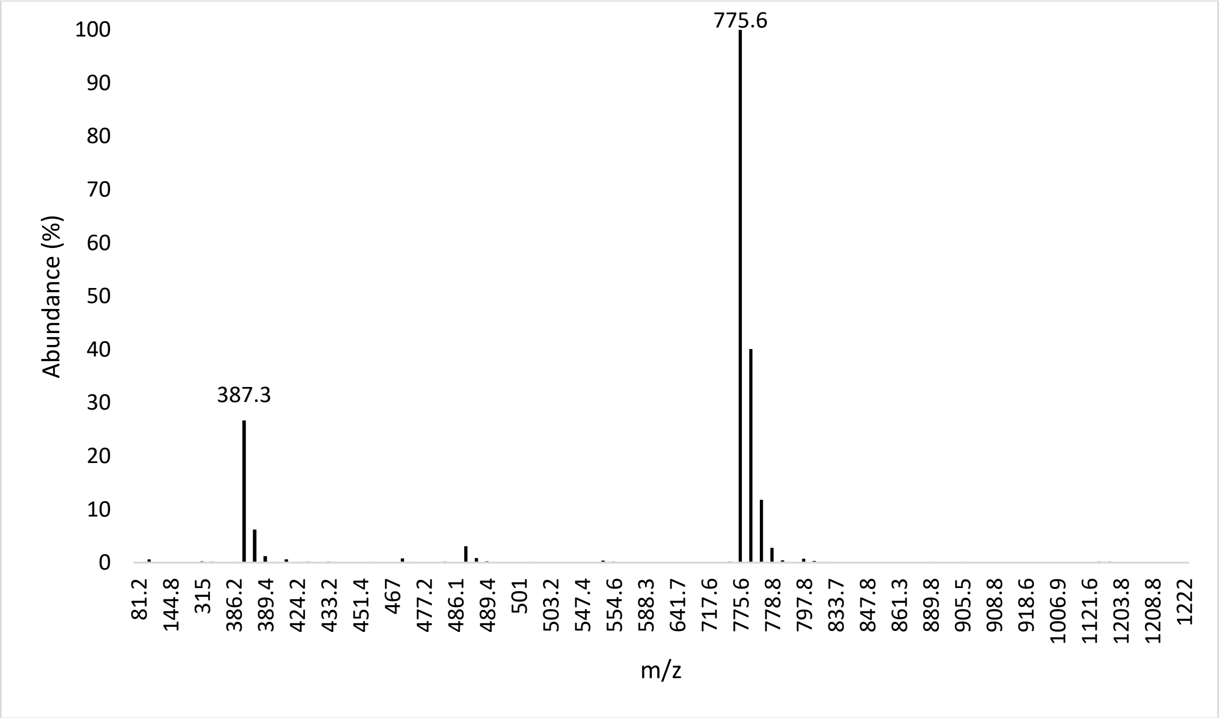

Figure S94. MS spectrum extracted from DA-LA reaction product optimized chromatogram at retention time 11.0 min. Labeled m/z signals represent 3LA1DA.

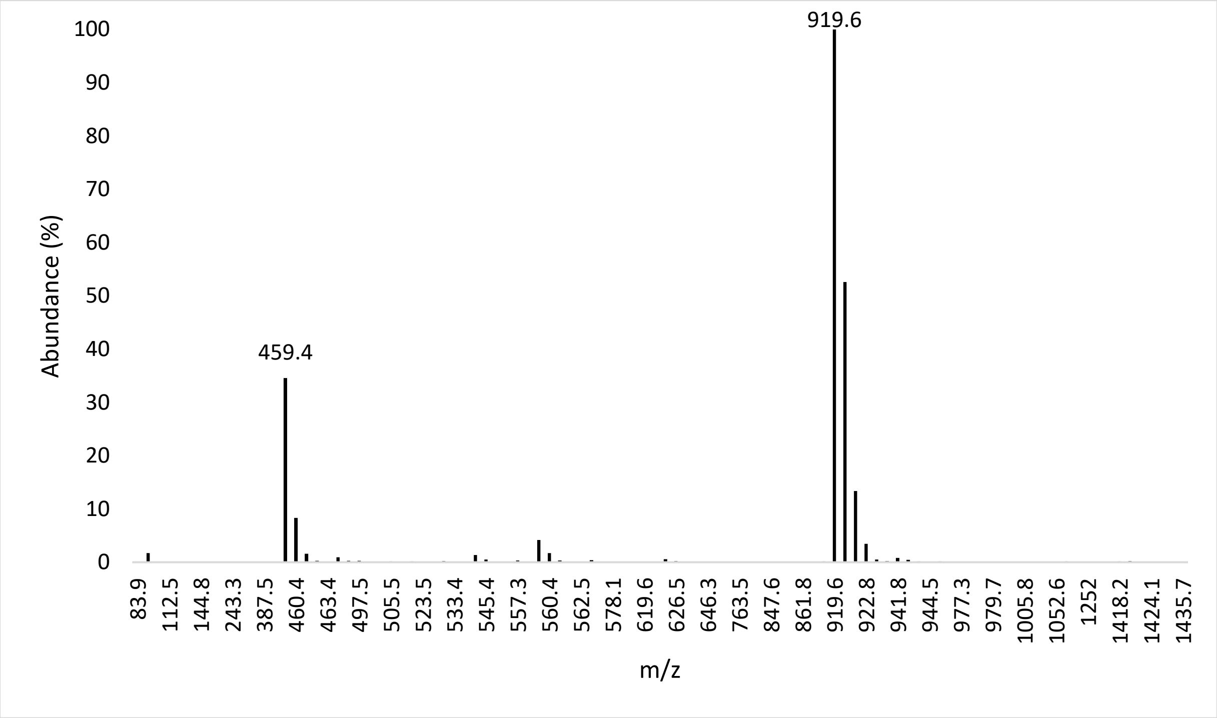

Figure S95. MS spectrum extracted from DA-LA reaction product optimized chromatogram at retention time 11.4 min. Labeled m/z signals represent 4LA1DA.

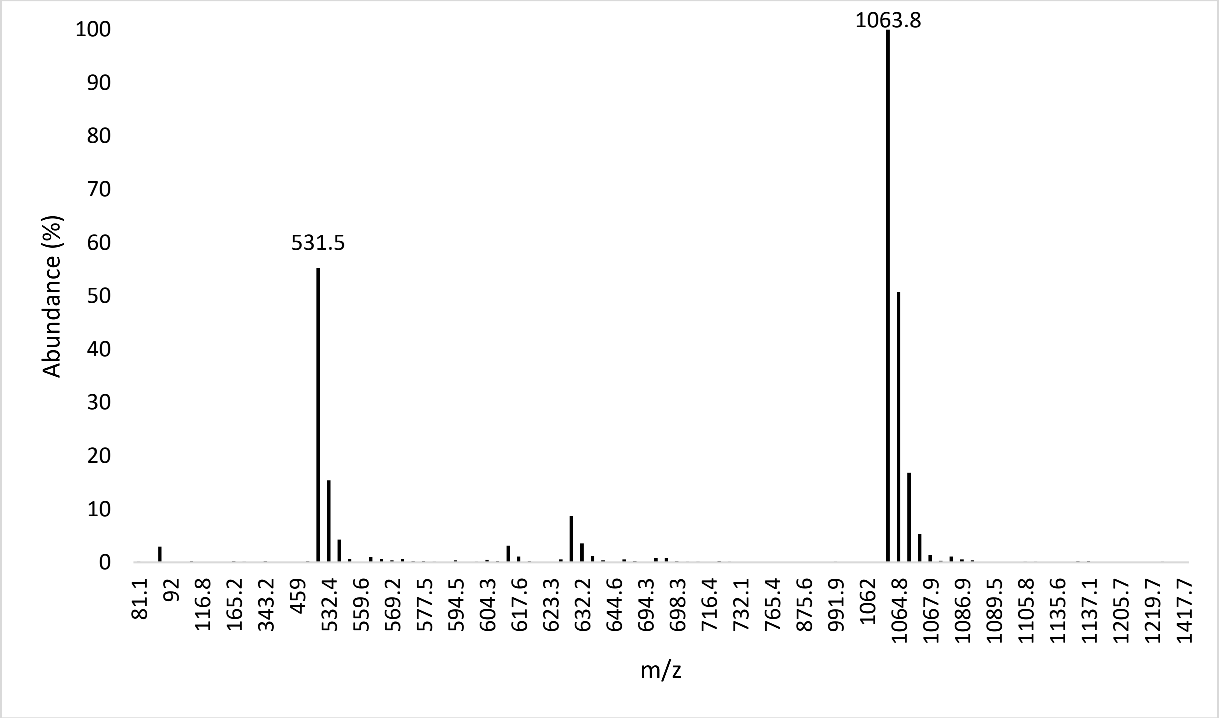

Figure S96. MS spectrum extracted from DA-LA reaction product optimized chromatogram at retention time 11.8 min. Labeled m/z signals represent 5LA1DA.

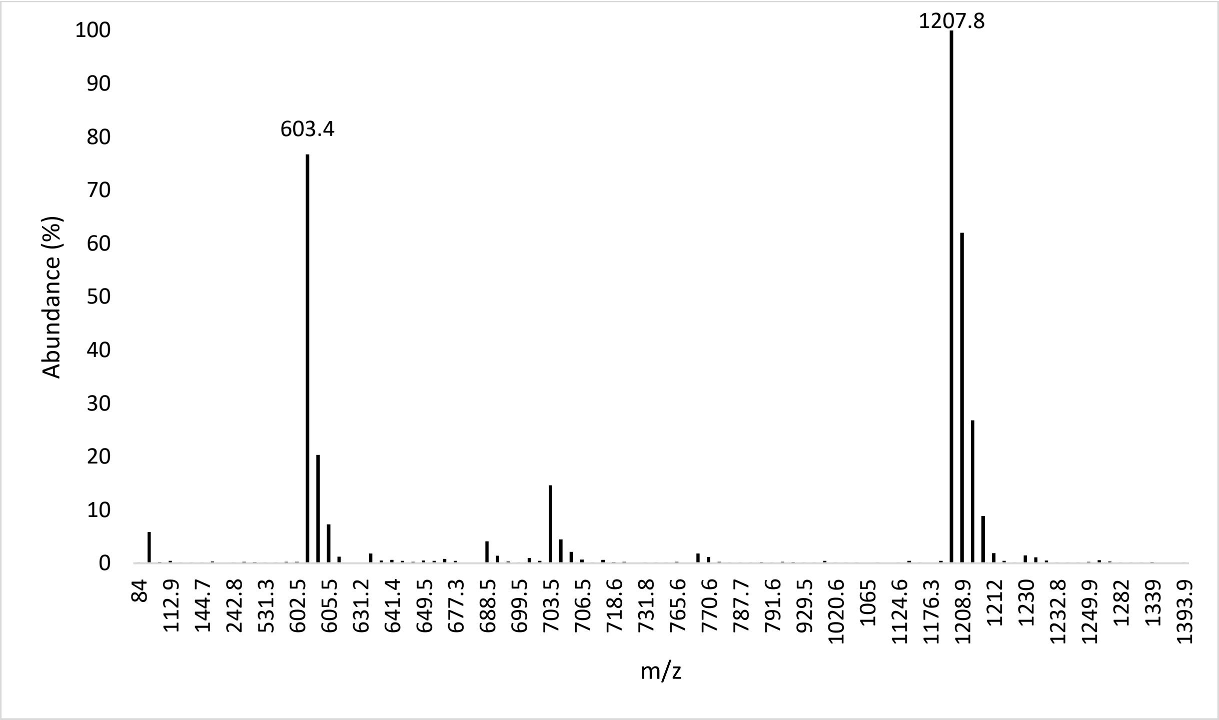

Figure S97. MS spectrum extracted from DA-LA reaction product optimized chromatogram at retention time 12.2 min. Labeled m/z signals represent 6LA1DA.

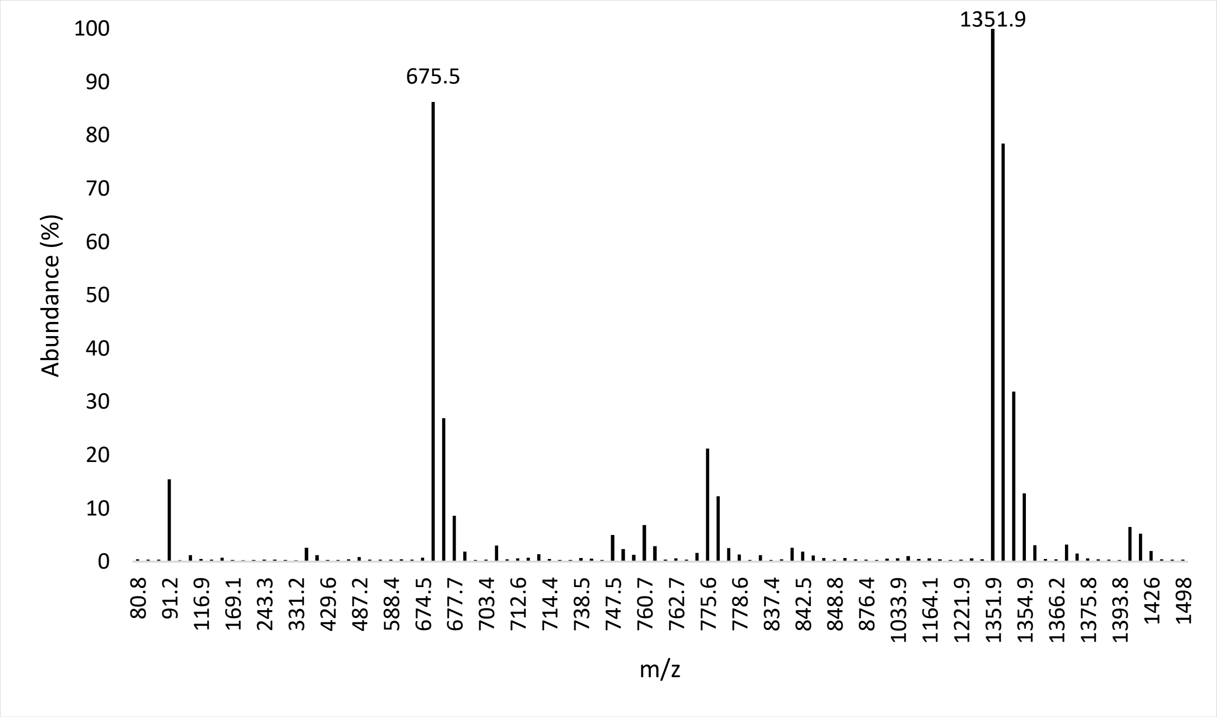

Figure S98. MS spectrum extracted from DA-LA reaction product optimized chromatogram at retention time 12.5 min. Labeled m/z signals represent 7LA1DA.

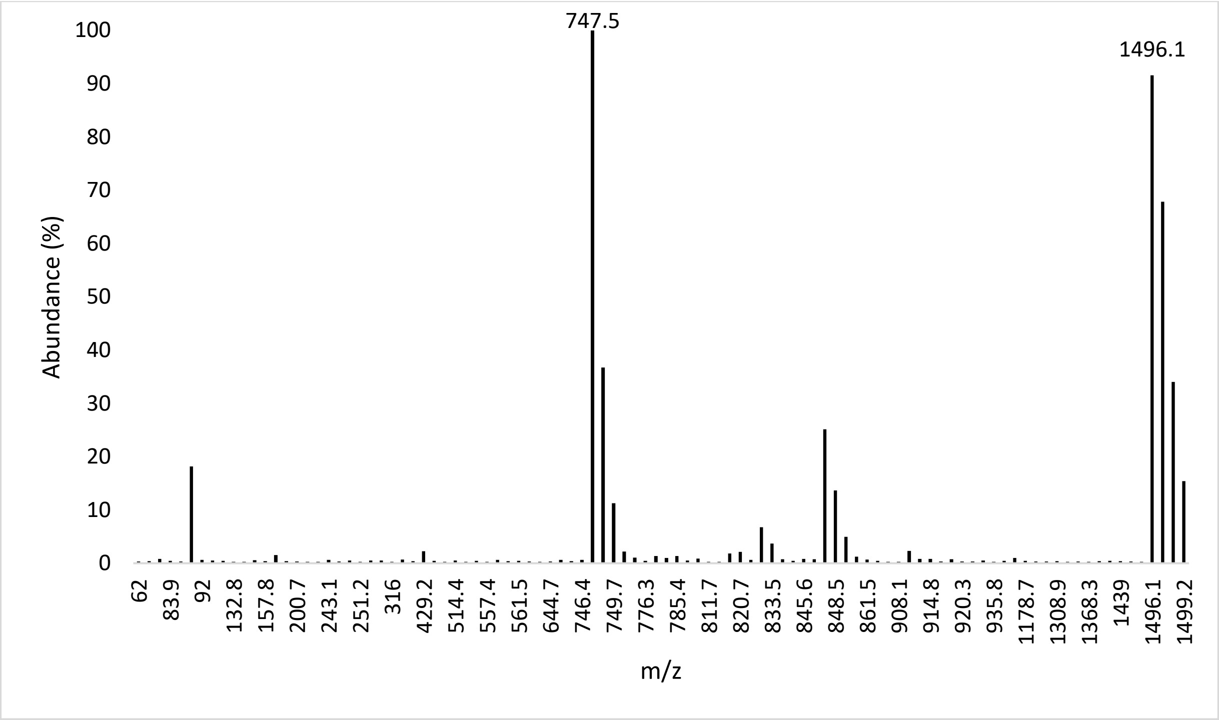

Figure S99. MS spectrum extracted from DA-LA reaction product optimized chromatogram at retention time 12.8 min. Labeled m/z signals represent 8LA1DA.

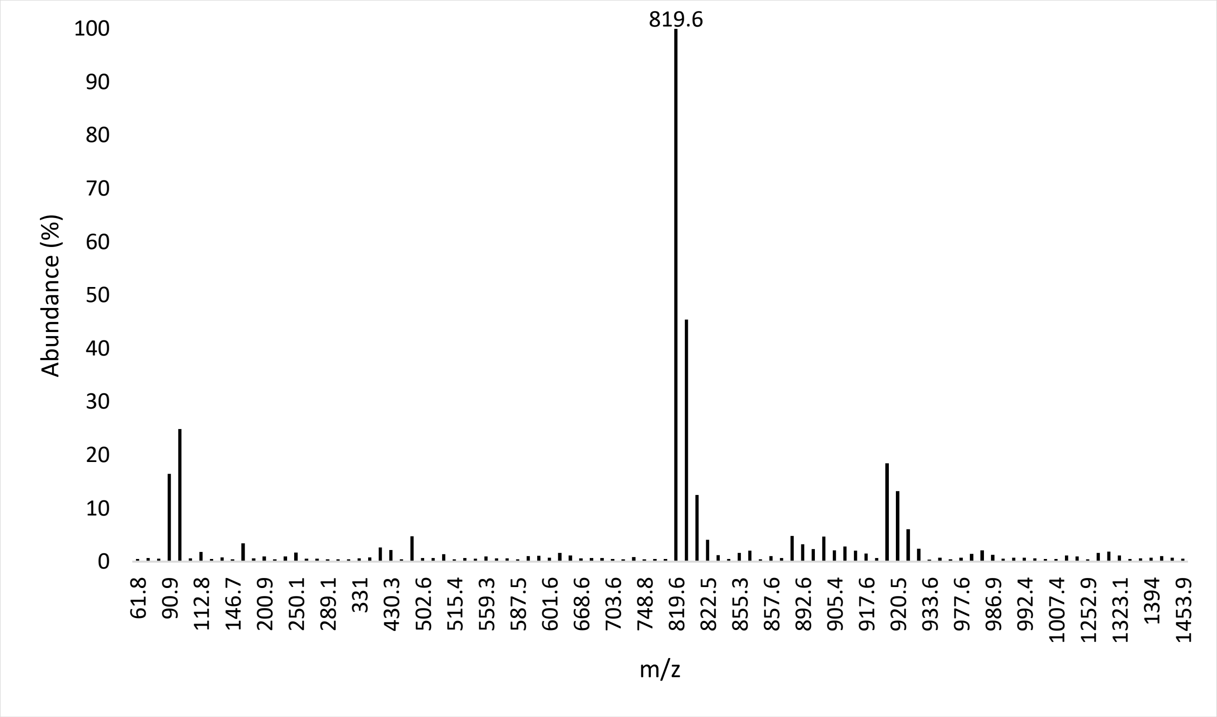

Figure S100. MS spectrum extracted from DA-LA reaction product optimized chromatogram at retention time 13.1 min. The labeled m/z signal represents 9LA1DA.

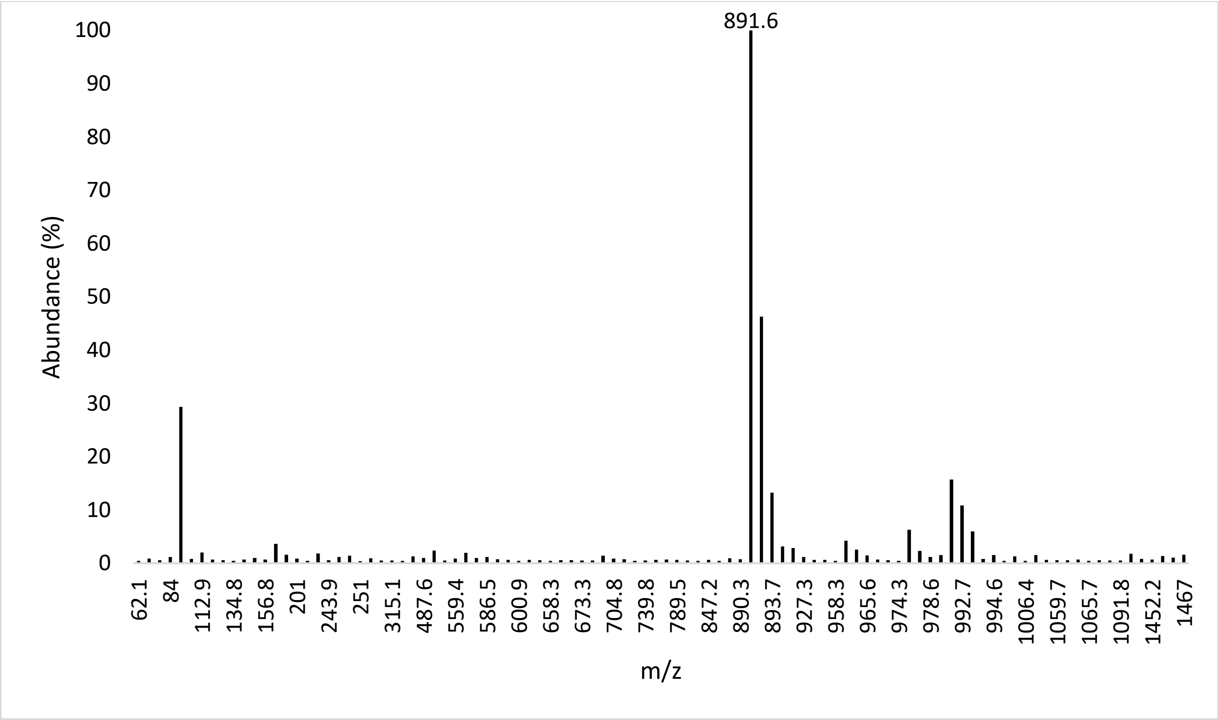

Figure S101. MS spectrum extracted from DA-LA reaction product optimized chromatogram at retention time 13.4 min. The labeled m/z signal represents 10LA1DA.

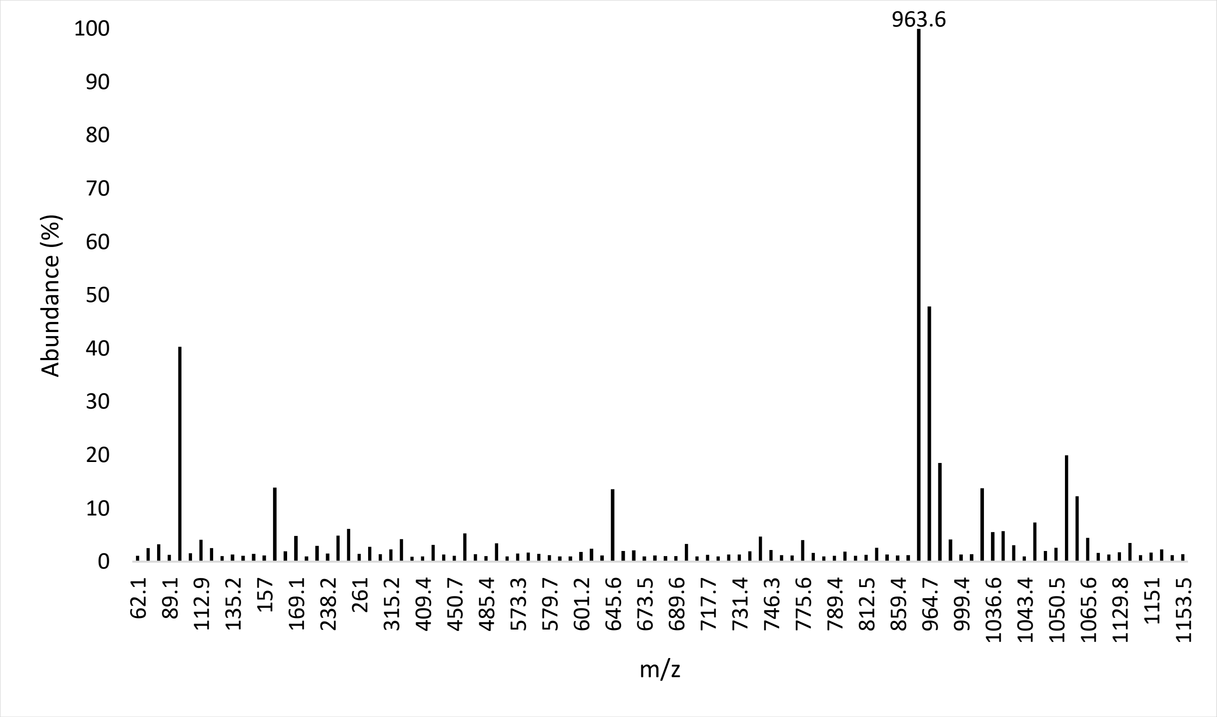

Figure S102. MS spectrum extracted from DA-LA reaction product optimized chromatogram at retention time 13.7 min. The labeled m/z signal represents 11LA1DA.

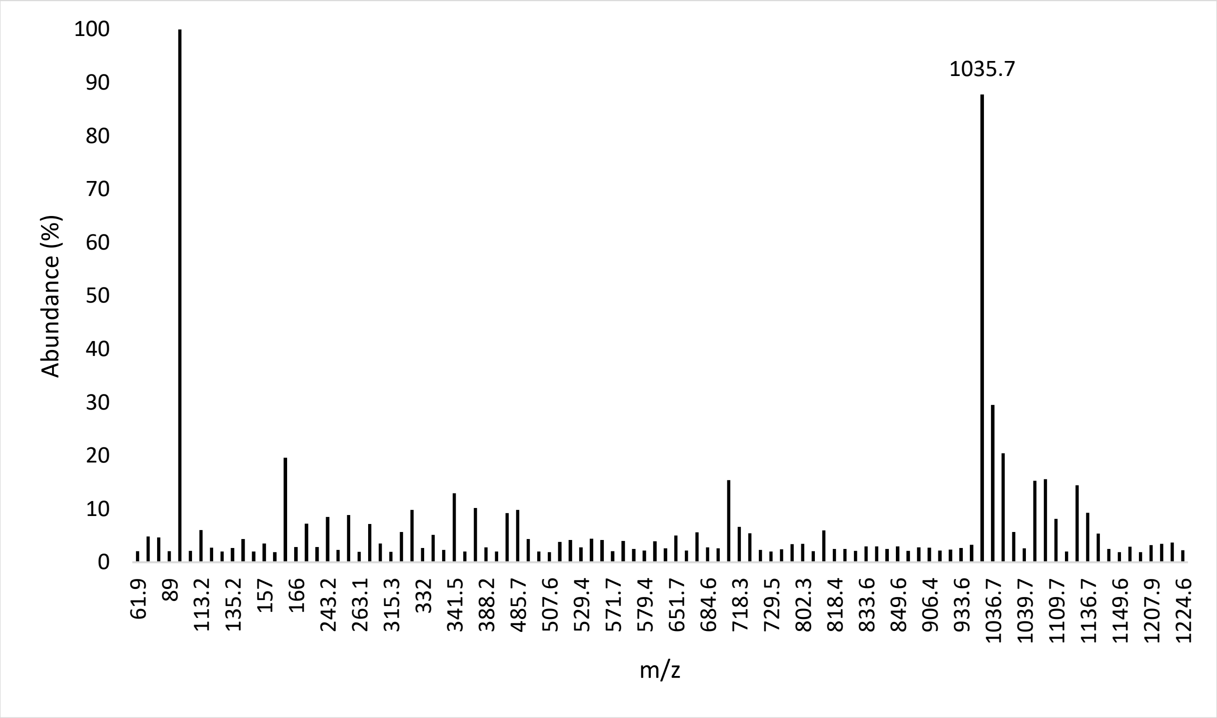

Figure S103. MS spectrum extracted from DA-LA reaction product optimized chromatogram at retention time 13.9 min. The labeled m/z signal represents 12LA1DA.

Table S6. Identification of LA reaction products (in the absence of DA). The detected products based on retention time and their corresponding m/z and ionization pattern as determined by LC-MS.

Retention time (min)	Compound	M (g/mol)	Corresponding m/z (-TIC)	Ionization pattern
**12.5**	2LA	162.1	161.1, 323.2	$[M-H]^-$, $[2M-H]^-$
**13.2**	2LA	162.1	161.3, 323.2	$[M-H]^-$, $[2M-H]^-$
**15.6**	3LA	234.2	233.2, 467.3	$[M-H]^-$, $[2M-H]^-$
**17.8**	4LA	306.3	305.2, 611.4	$[M-H]^-$, $[2M-H]^-$
**19.7**	5LA	378.3	377.2, 755.3	$[M-H]^-$, $[2M-H]^-$
**21.0**	6LA	450.4	449.2, 899.6	$[M-H]^-$, $[2M-H]^-$
**21.2**	6LA	450.4	449.3, 899.4	$[M-H]^-$, $[2M-H]^-$
**22.2**	7LA	522.4	521.3, 1043.6	$[M-H]^-$, $[2M-H]^-$
**22.4**	7LA	522.4	521.4, 1043.7	$[M-H]^-$, $[2M-H]^-$
**23.3**	8LA	594.5	593.4, 1187.7	$[M-H]^-$, $[2M-H]^-$
**23.5**	8LA	594.5	593.4, 1187.8	$[M-H]^-$, $[2M-H]^-$
**24.4**	9LA	666.6	665.4, 1331.9	$[M-H]^-$, $[2M-H]^-$
**24.6**	9LA	666.6	665.5	$[M-H]^-$
**25.2**	10LA	738.6	737.4, 1475.9	$[M-H]^-$, $[2M-H]^-$
**25.7**	11LA	810.7	809.4	$[M-H]^-$
**25.9**	11LA	810.7	809.4	$[M-H]^-$
**26.6**	12LA	882.7	881.6	$[M-H]^-$
**27.1**	13LA	954.8	953.6	$[M-H]^-$
**27.6**	14LA	1026.9	1025.6	$[M-H]^-$
**28.1**	15LA	1098.9	1097.7	$[M-H]^-$
**28.5**	16LA	1171.0	1169.7	$[M-H]^-$
**28.9**	17LA	1243.0	1241.8	$[M-H]^-$
**29.1**	18LA	1315.1	1313.8	$[M-H]^-$

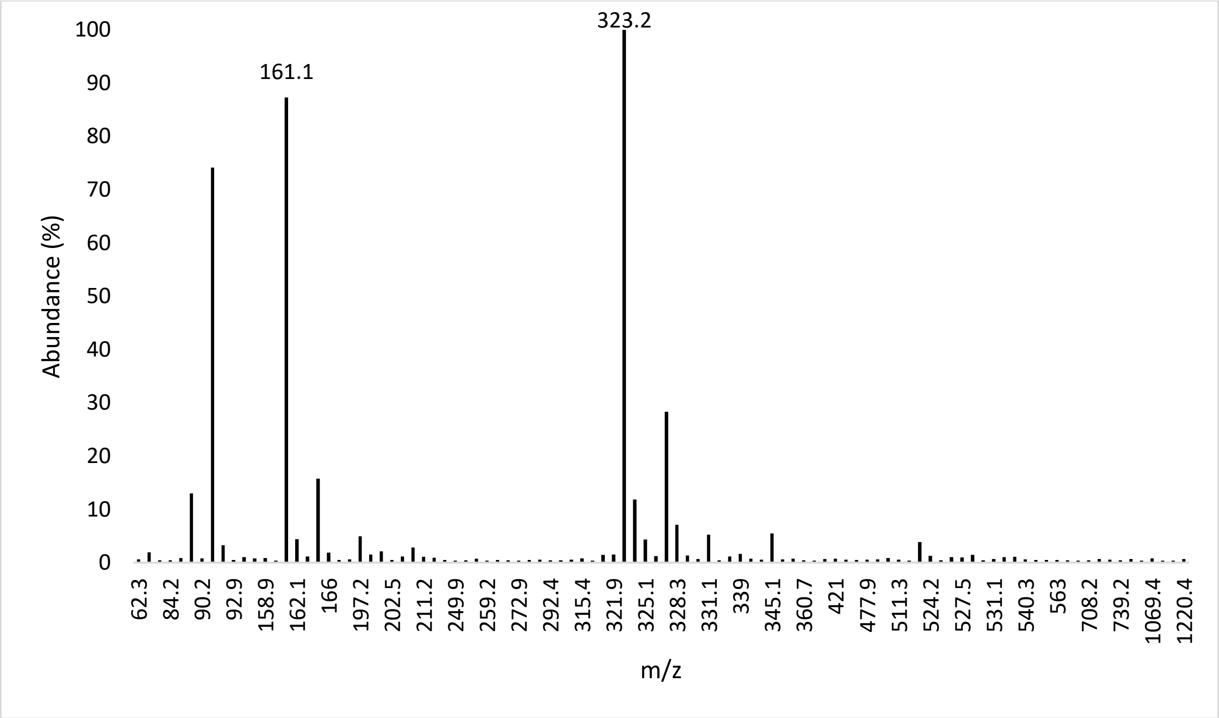

Figure S104. MS spectrum extracted from LA control chromatogram at retention time 12.5 min. Labeled m/z signals represent 2LA.

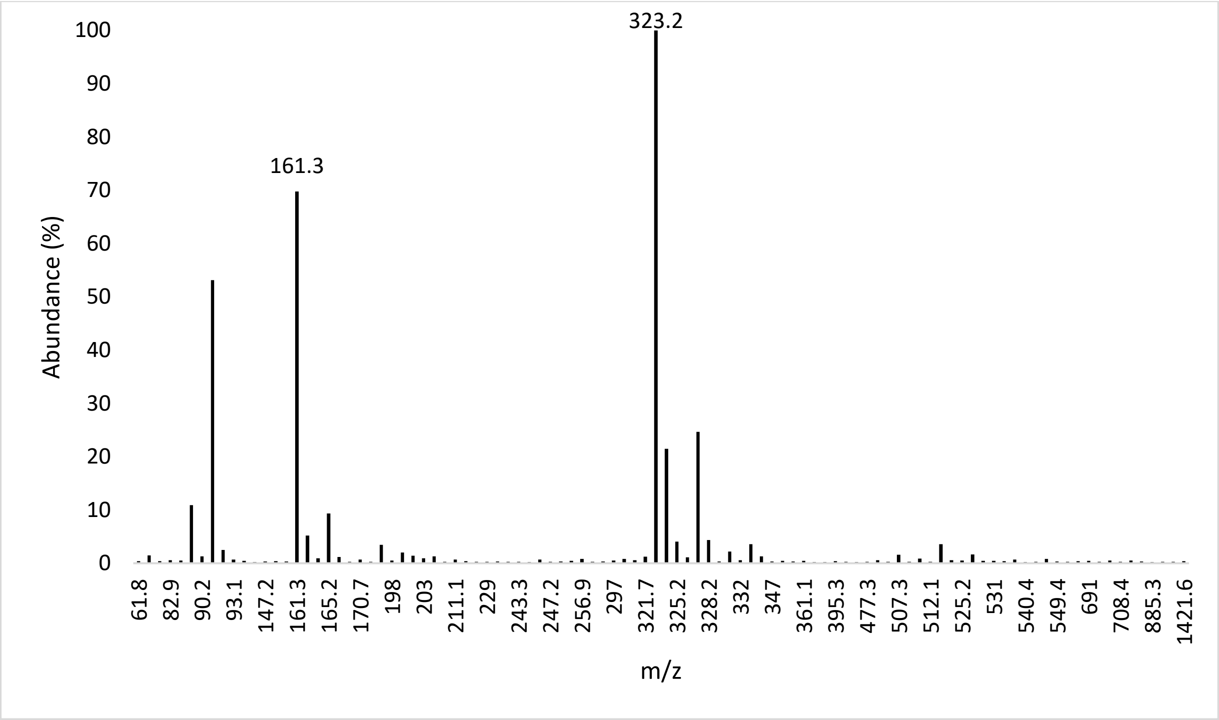

Figure S105. MS spectrum extracted from LA control chromatogram at retention time 13.2 min. Labeled m/z signals represent 2LA.

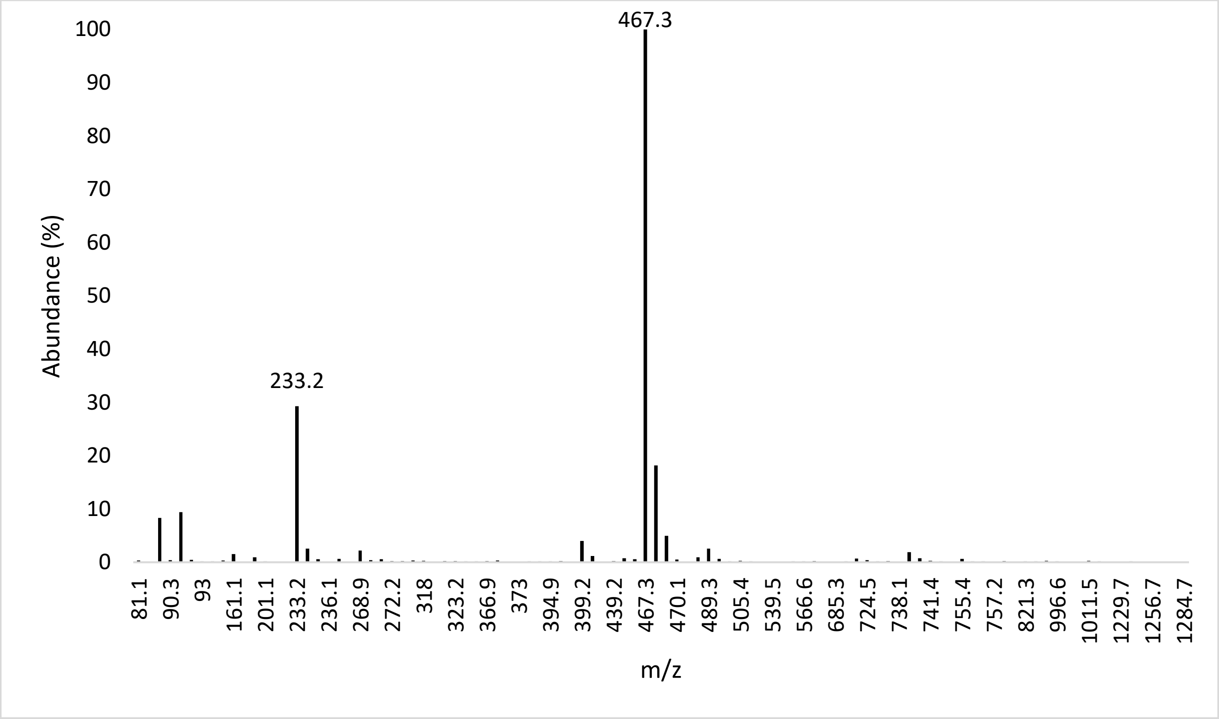

Figure S106. MS spectrum extracted from LA control chromatogram at retention time 15.6 min. Labeled m/z signals represent 3LA.

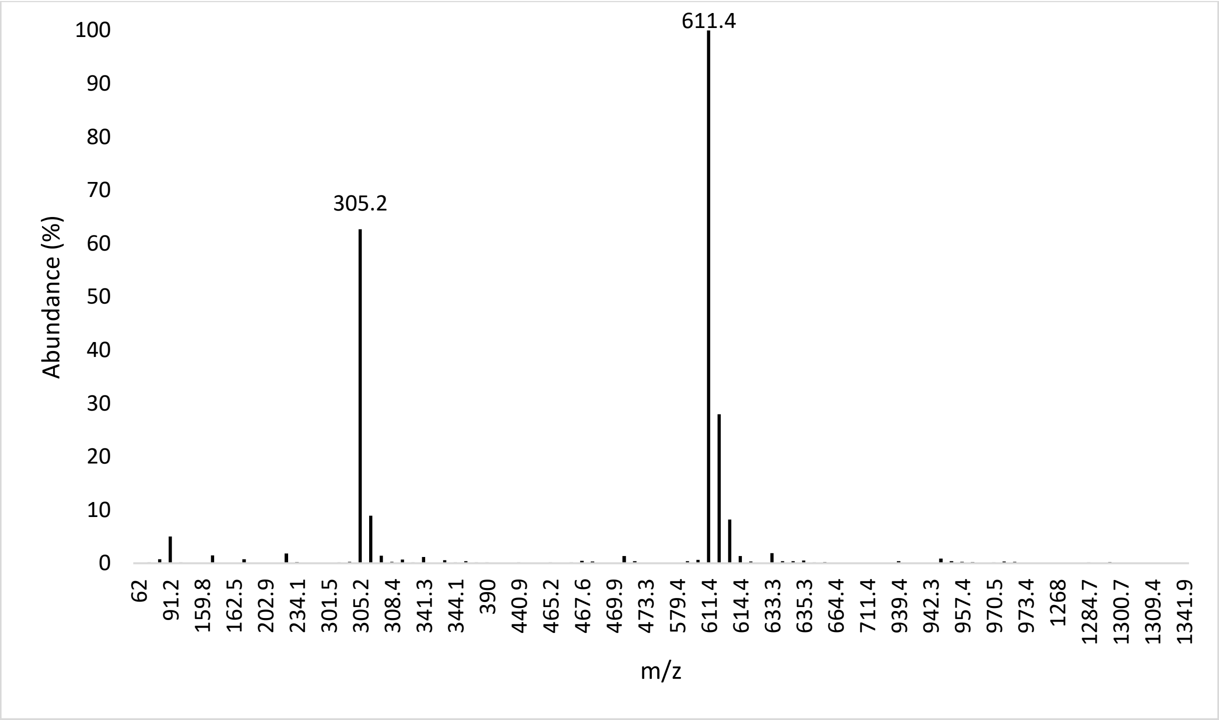

Figure S107. MS spectrum extracted from LA control chromatogram at retention time 17.8 min. Labeled m/z signals represent 4LA.

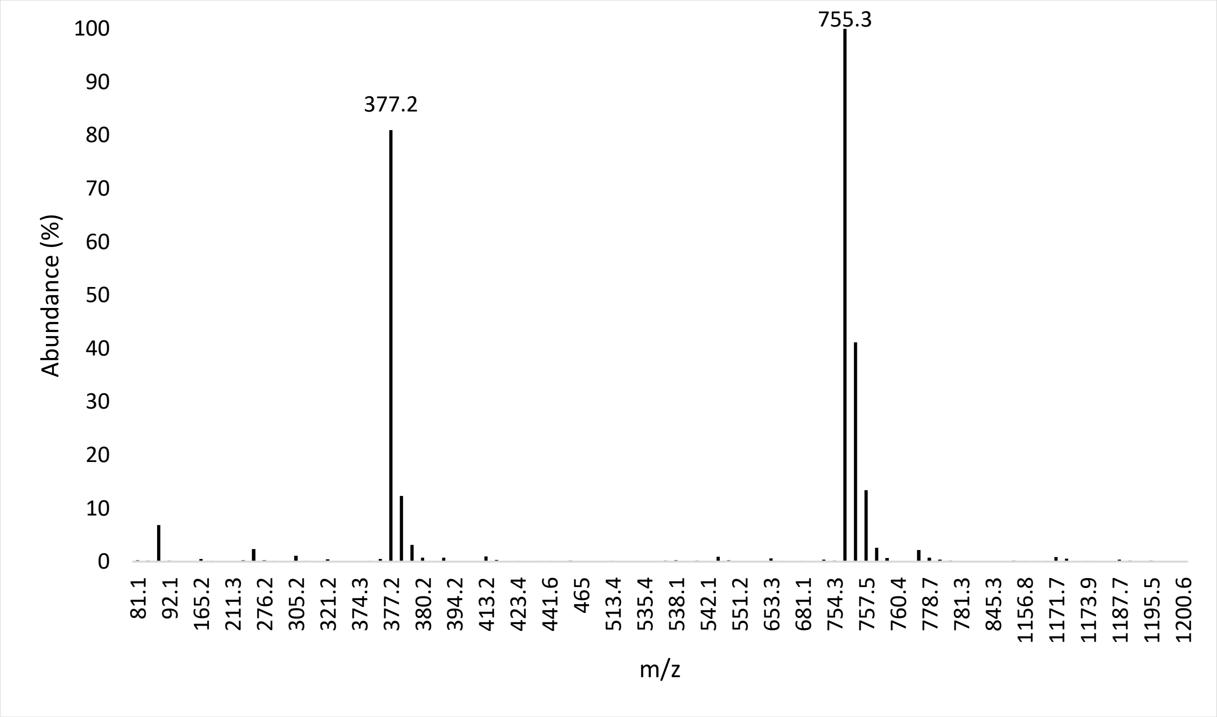

Figure S108. MS spectrum extracted from LA control chromatogram at retention time 19.7 min. Labeled m/z signals represent 5LA.

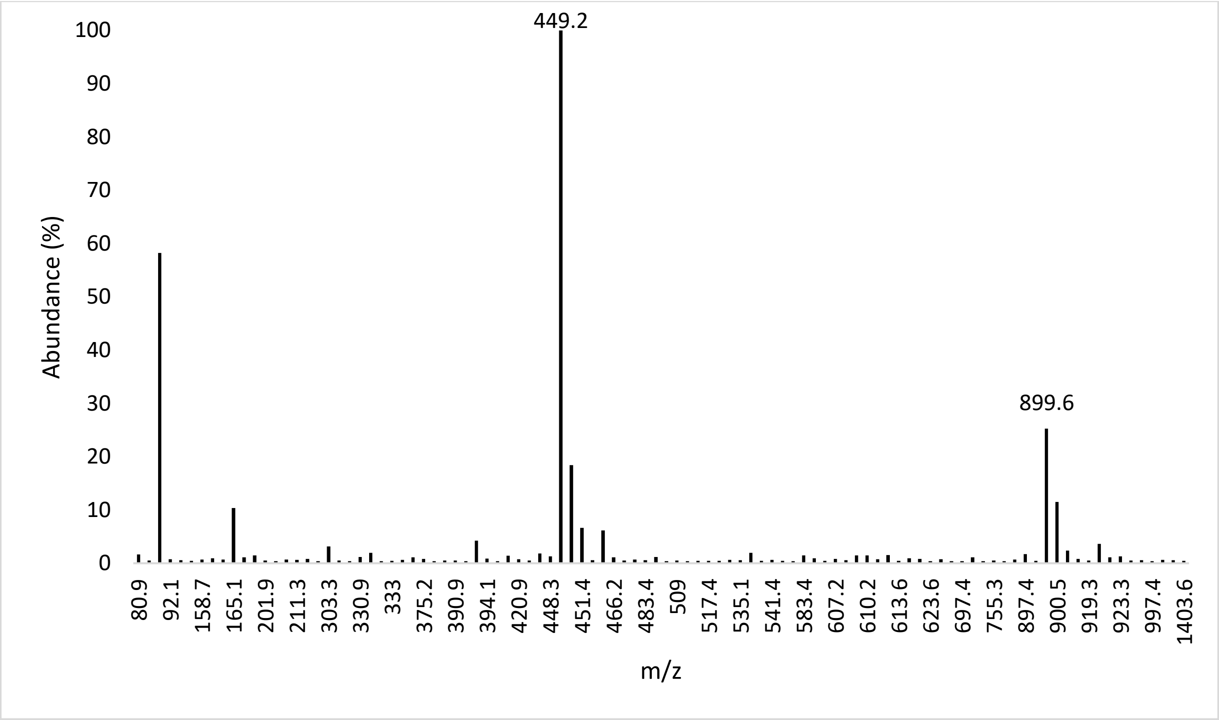

Figure S109. MS spectrum extracted from LA control chromatogram at retention time 21.0 min. Labeled m/z signals represent 6LA.

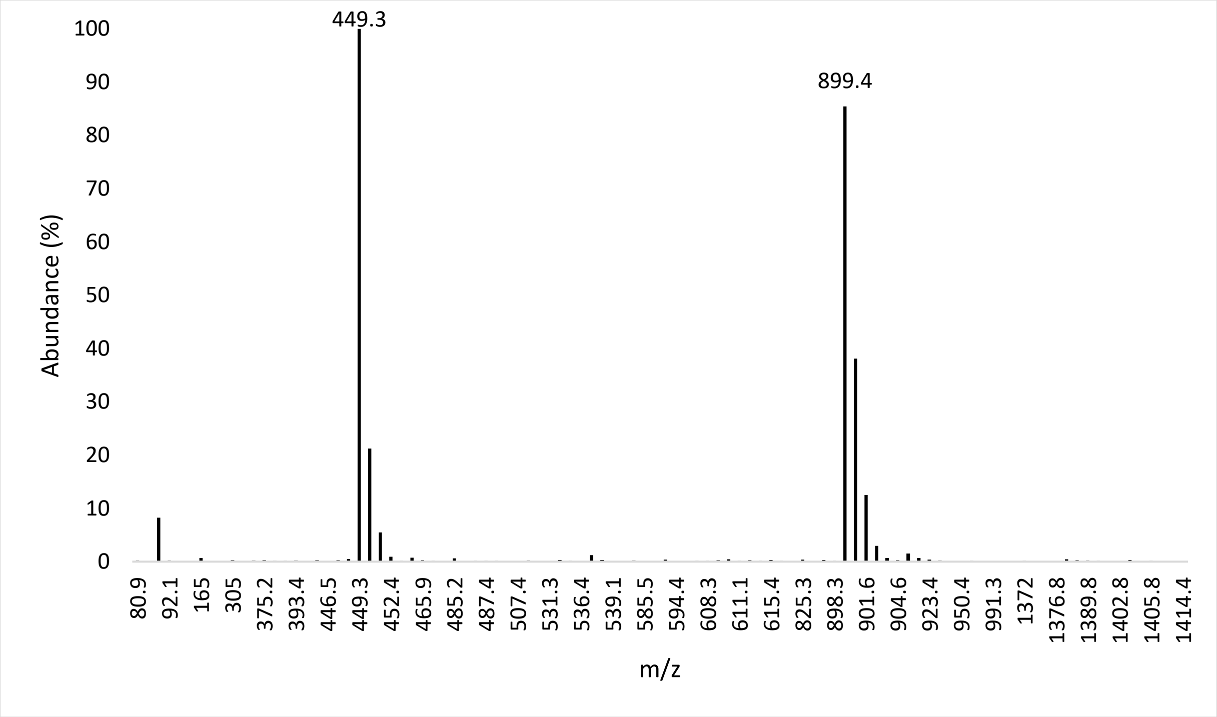

Figure S110. MS spectrum extracted from LA control chromatogram at retention time 21.2 min. Labeled m/z signals represent 6LA.

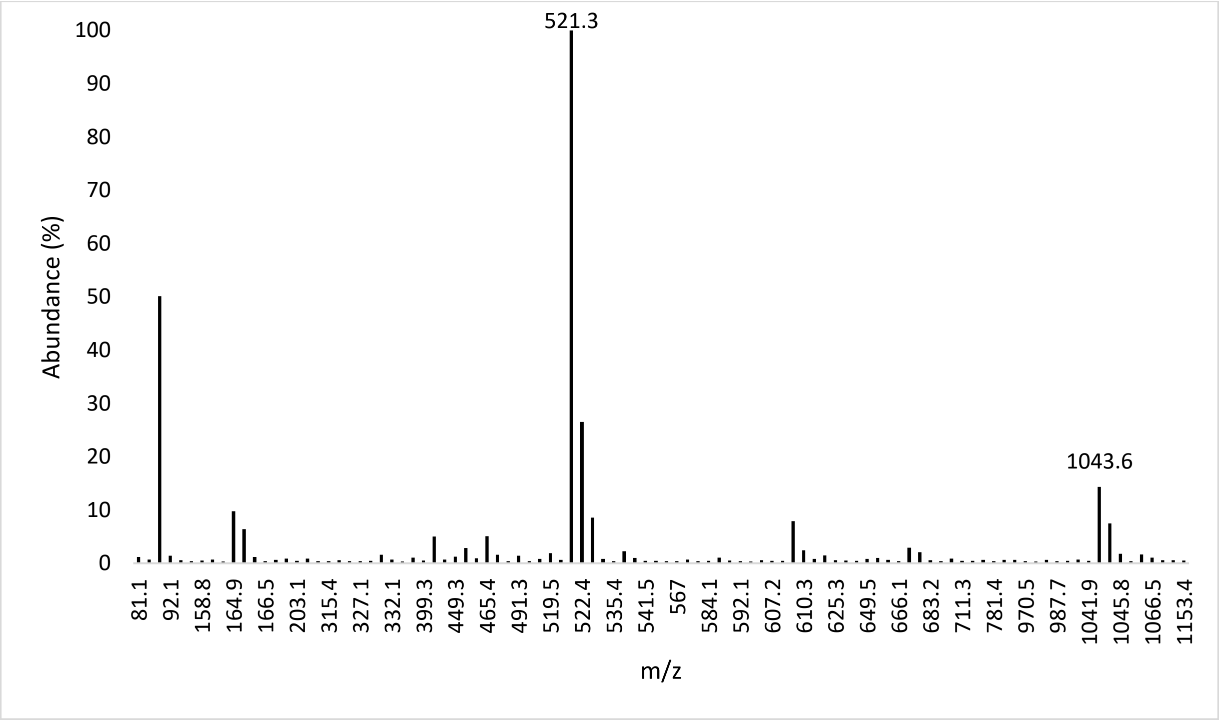

Figure S111. MS spectrum extracted from LA control chromatogram at retention time 22.2 min. Labeled m/z signals represent 7LA.

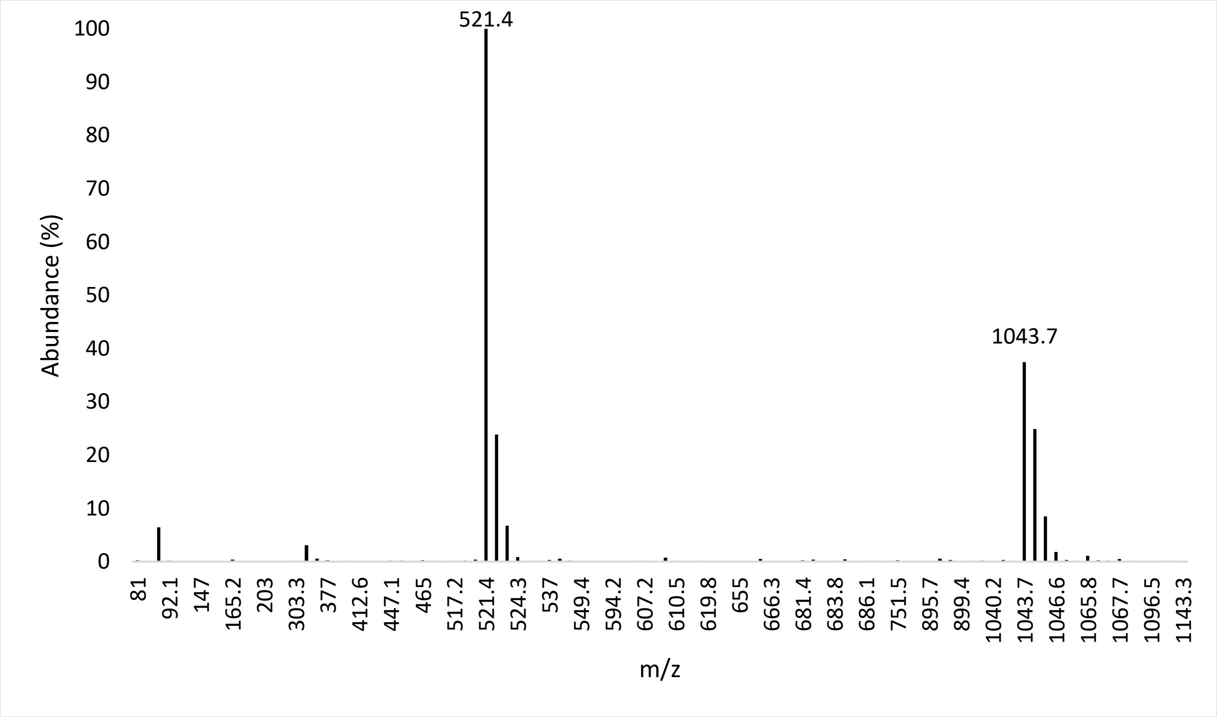

Figure S112. MS spectrum extracted from LA control chromatogram at retention time 22.4 min. Labeled m/z signals represent 7LA.

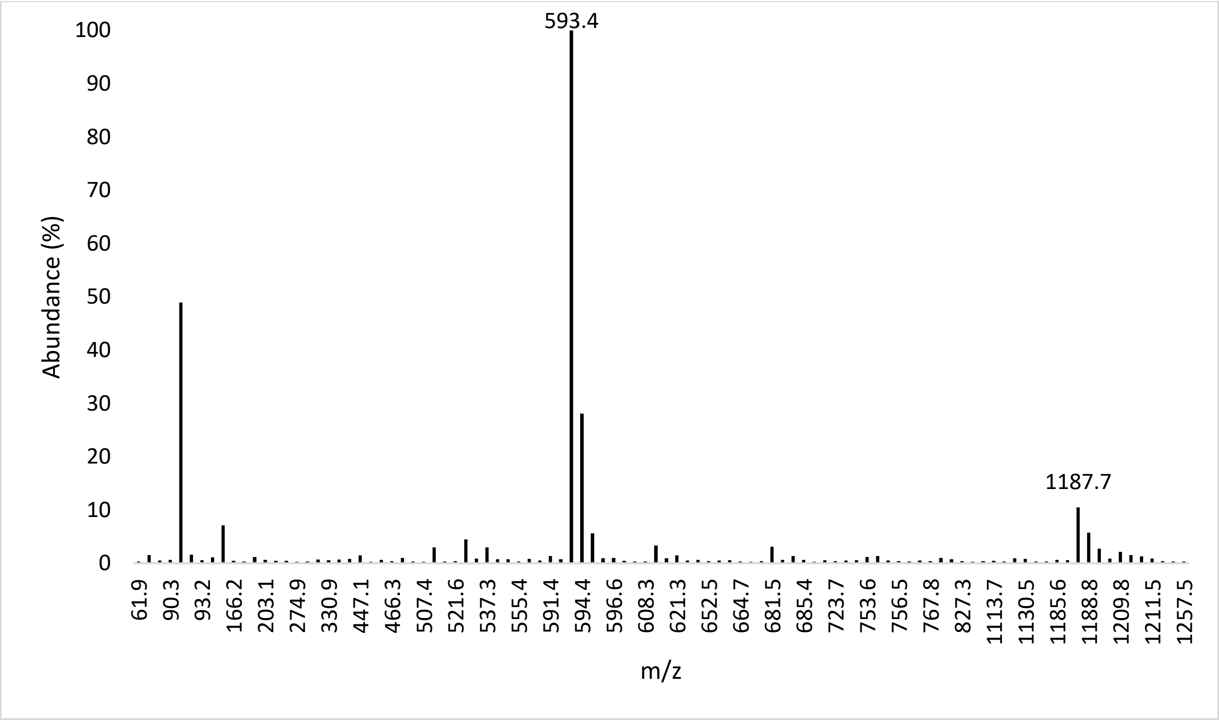

Figure S113. MS spectrum extracted from LA control chromatogram at retention time 23.3 min. Labeled m/z signals represent 8LA.

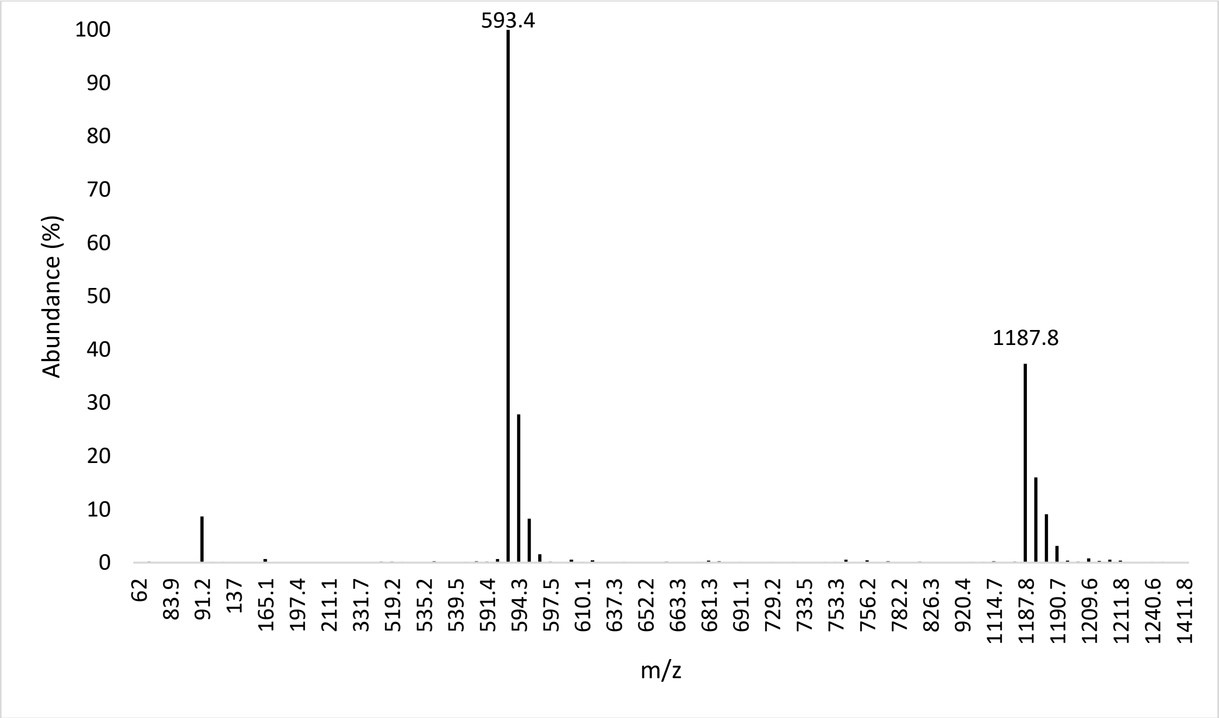

Figure S114. MS spectrum extracted from LA control chromatogram at retention time 23.5 min. Labeled m/z signals represent 8LA.

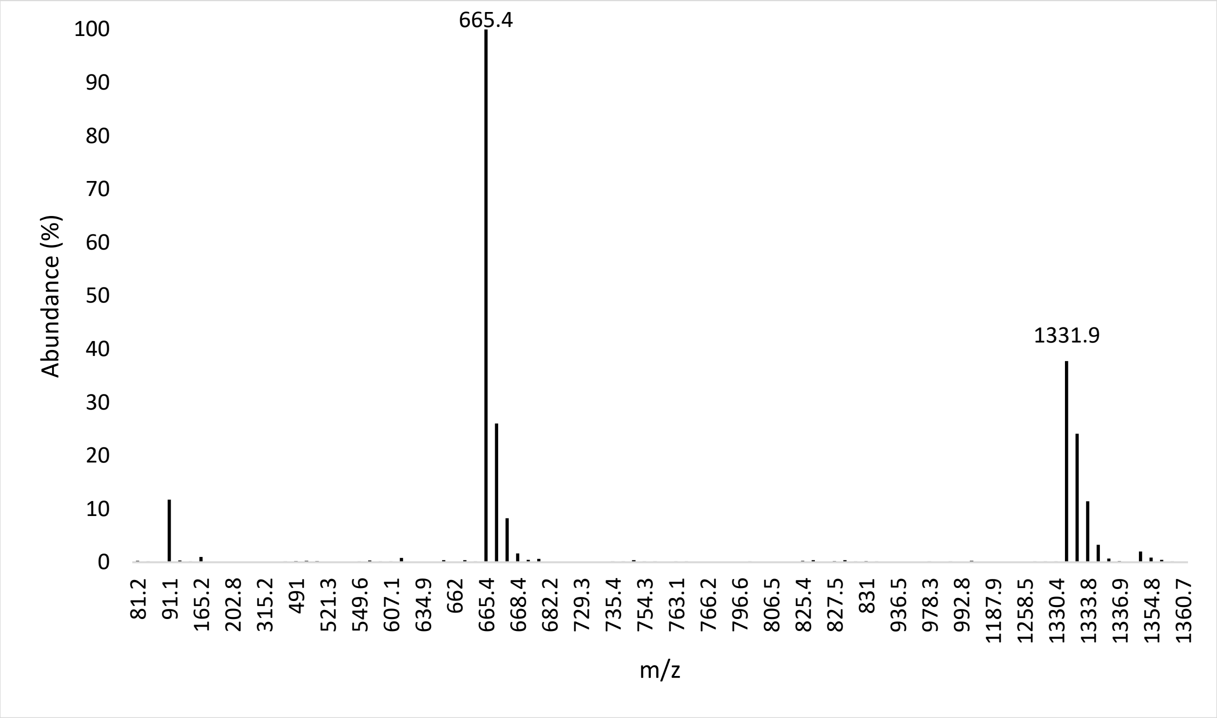

Figure S115. MS spectrum extracted from LA control chromatogram at retention time 24.4 min. Labeled m/z signals represent 9LA.

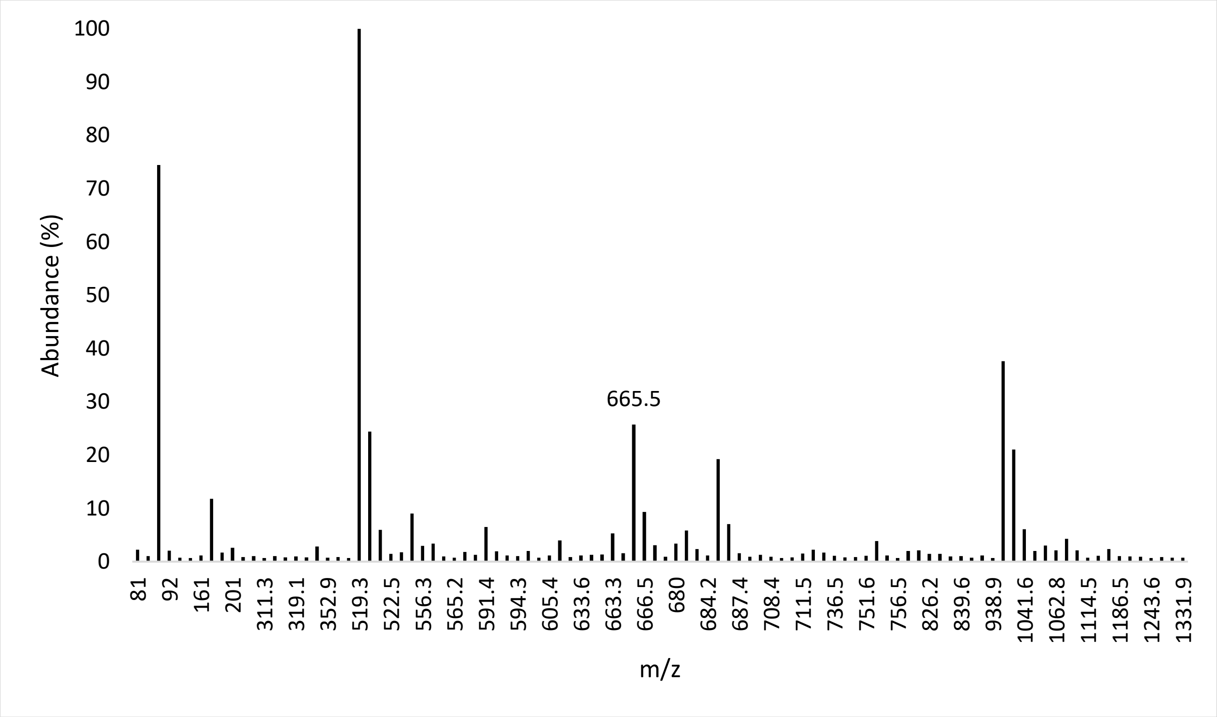

Figure S116. MS spectrum extracted from LA control chromatogram at retention time 24.6 min. Labeled m/z signals represent 9LA.

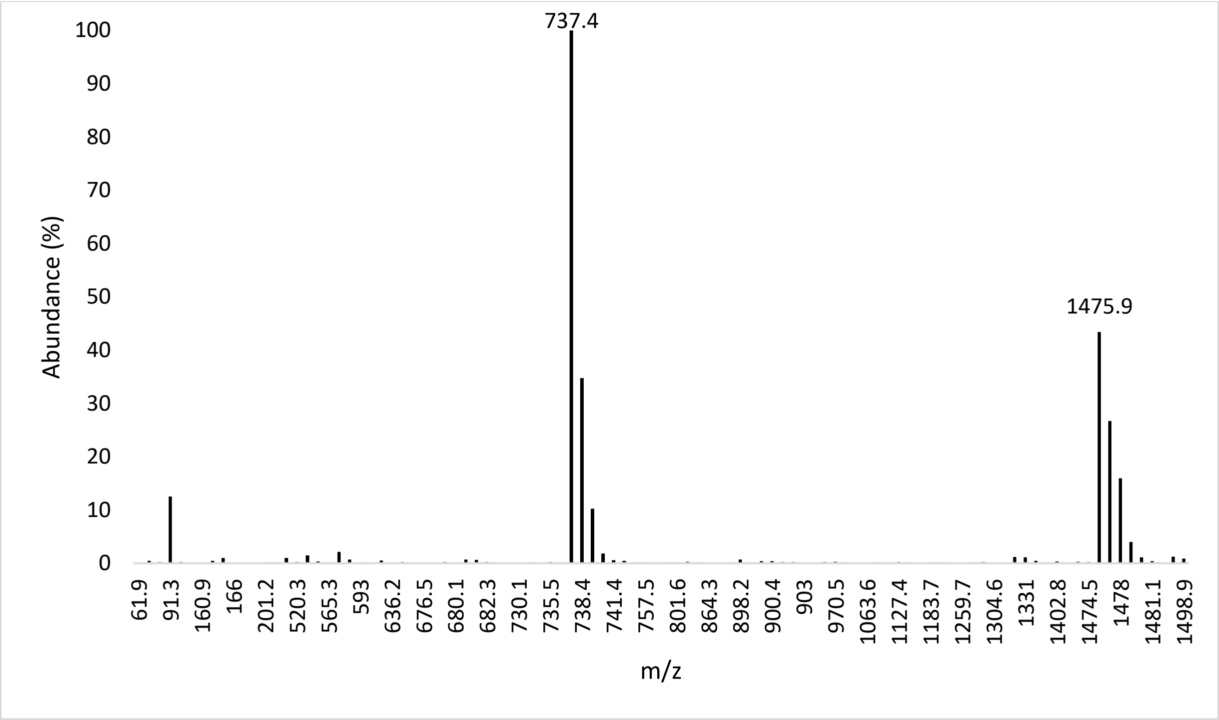

Figure S117. MS spectrum extracted from LA control chromatogram at retention time 25.2 min. Labeled m/z signals represent 10LA.

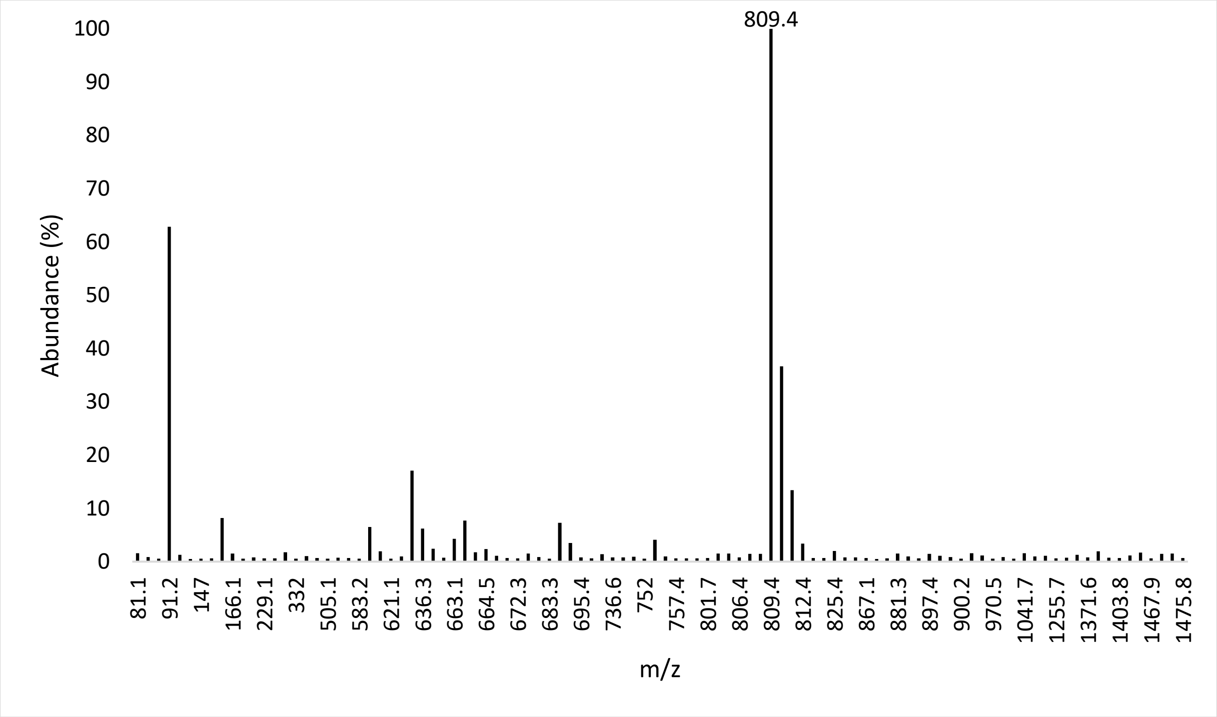

Figure S118. MS spectrum extracted from LA control chromatogram at retention time 25.7 min. Labeled m/z signal represents 11LA.

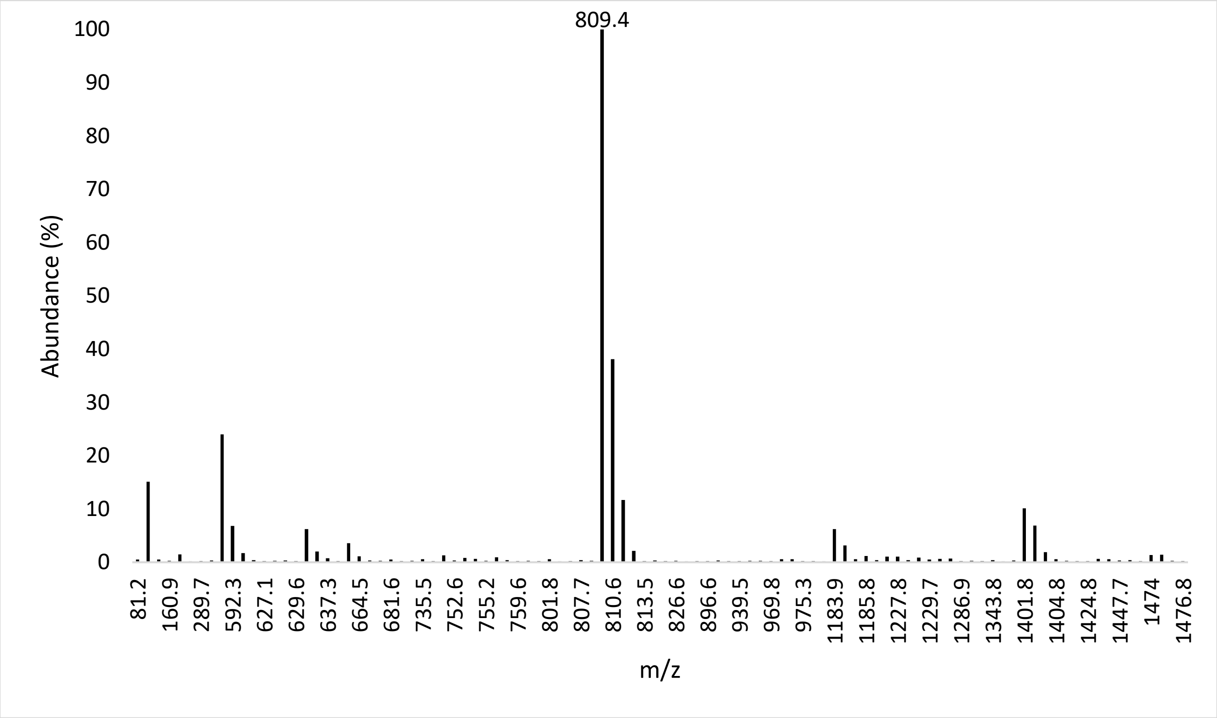

Figure S119. MS spectrum extracted from LA control chromatogram at retention time 25.9 min. Labeled m/z signal represents 11LA.

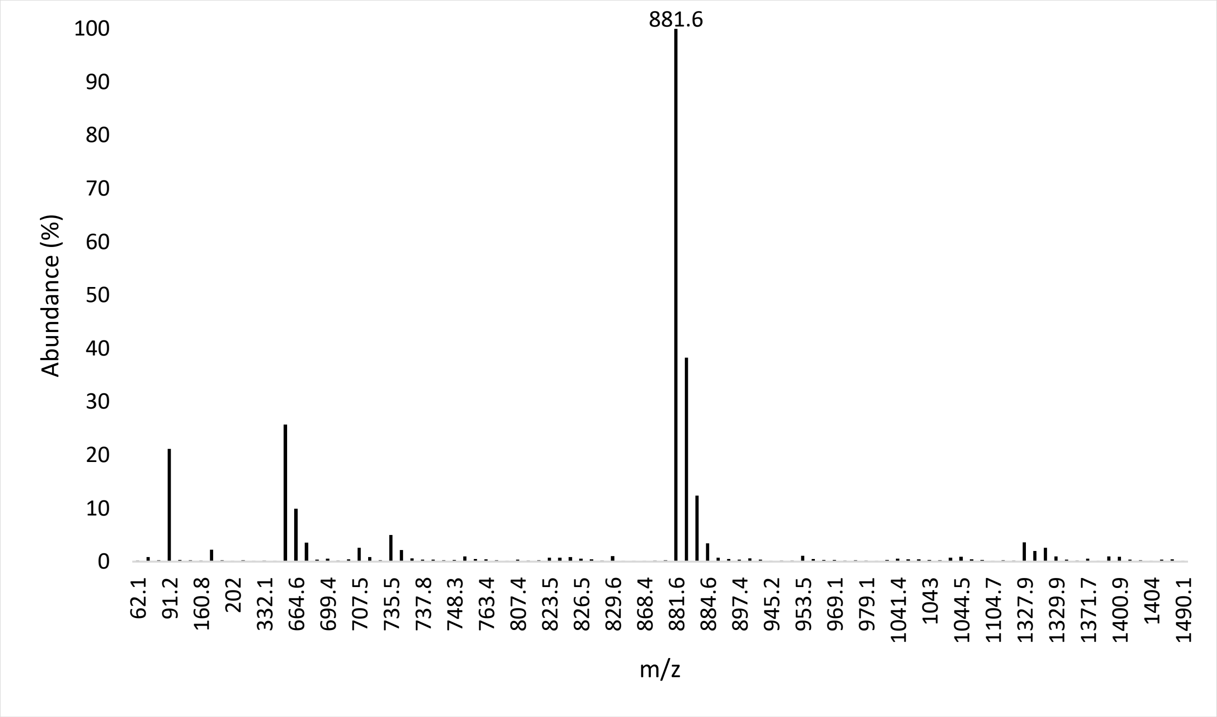

Figure S120. MS spectrum extracted from LA control chromatogram at retention time 26.6 min. Labeled m/z signal represents 12LA.

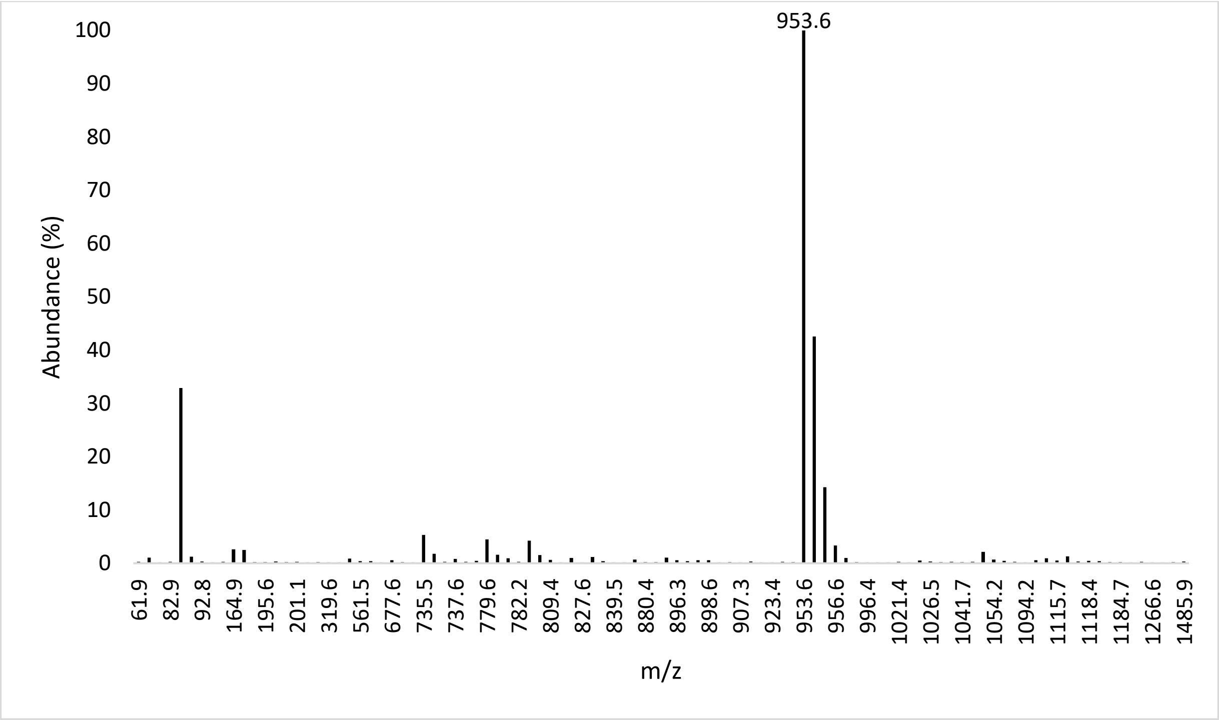

Figure S121. MS spectrum extracted from LA control chromatogram at retention time 27.1 min. Labeled m/z signal represents 13LA.

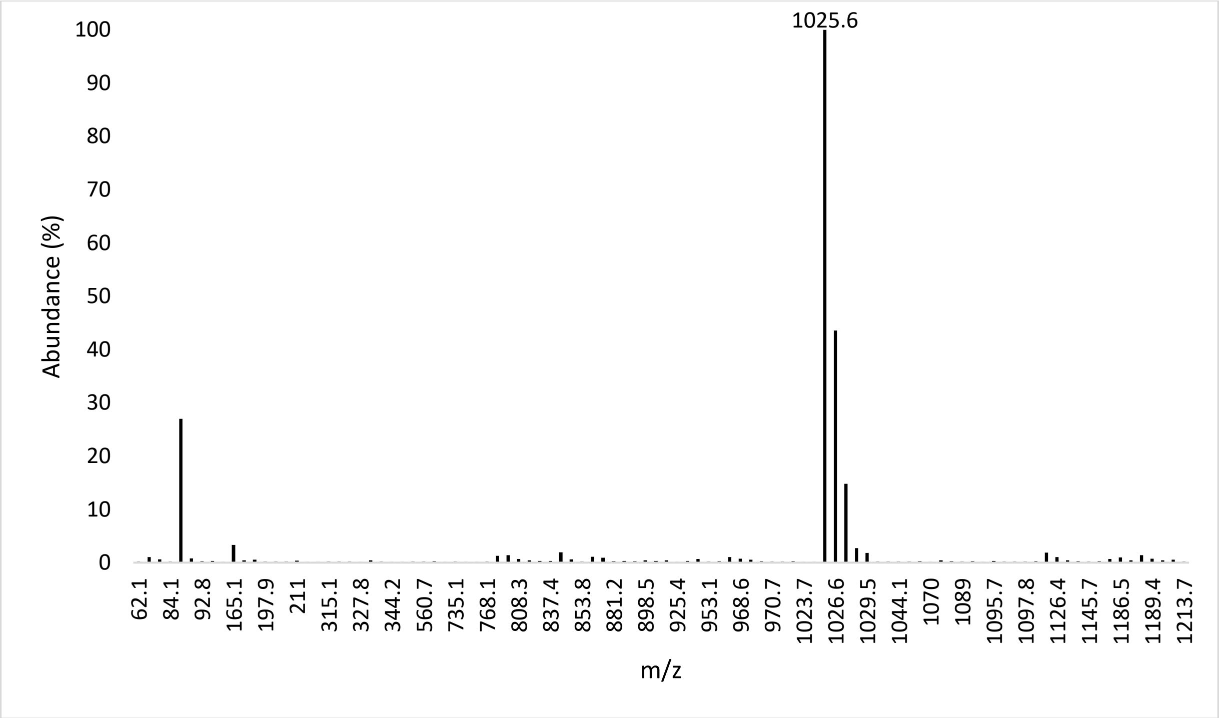

Figure S122. MS spectrum extracted from LA control chromatogram at retention time 27.6 min. Labeled m/z signal represents 14LA.

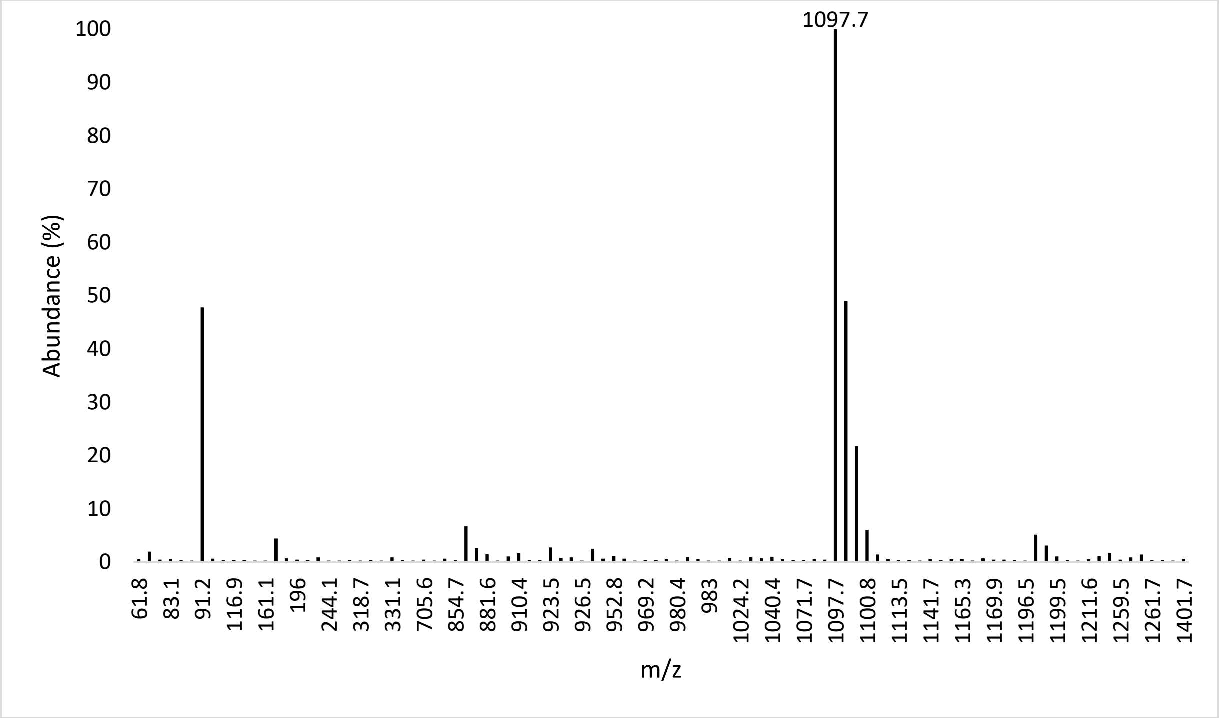

Figure S123. MS spectrum extracted from LA control chromatogram at retention time 28.1 min. Labeled m/z signal represents 15LA.

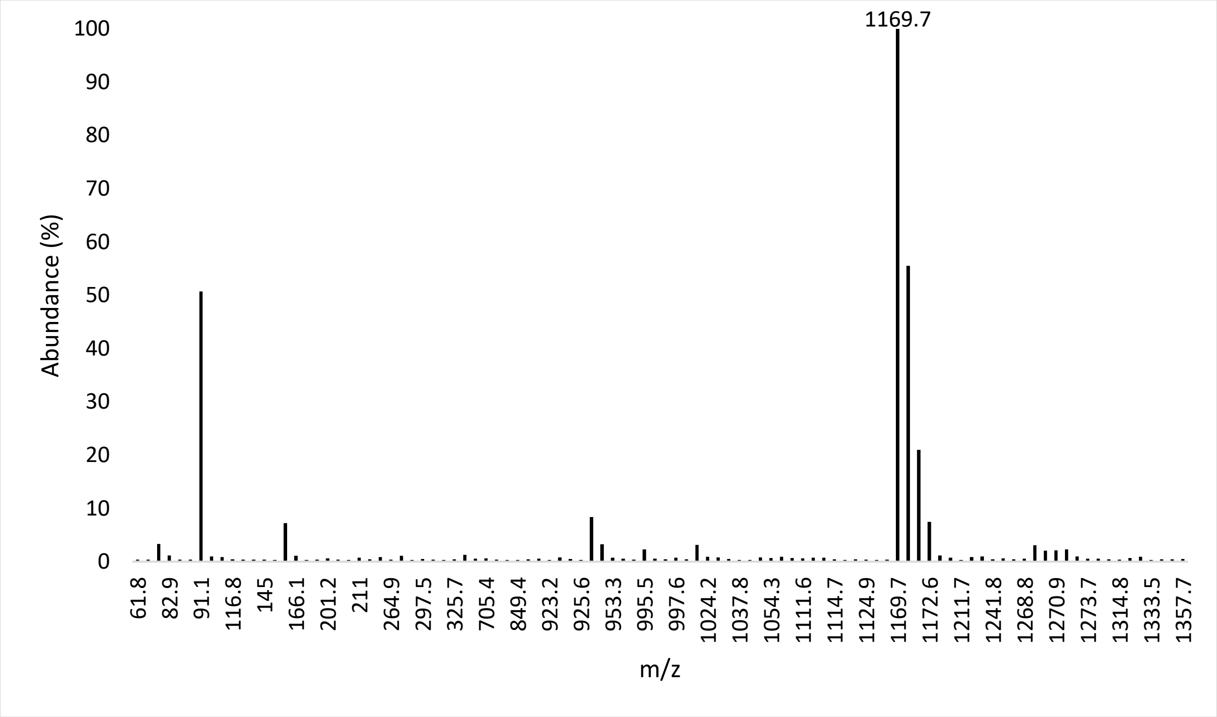

Figure S124. MS spectrum extracted from LA control chromatogram at retention time 28.5 min. Labeled m/z signal represents 16LA.

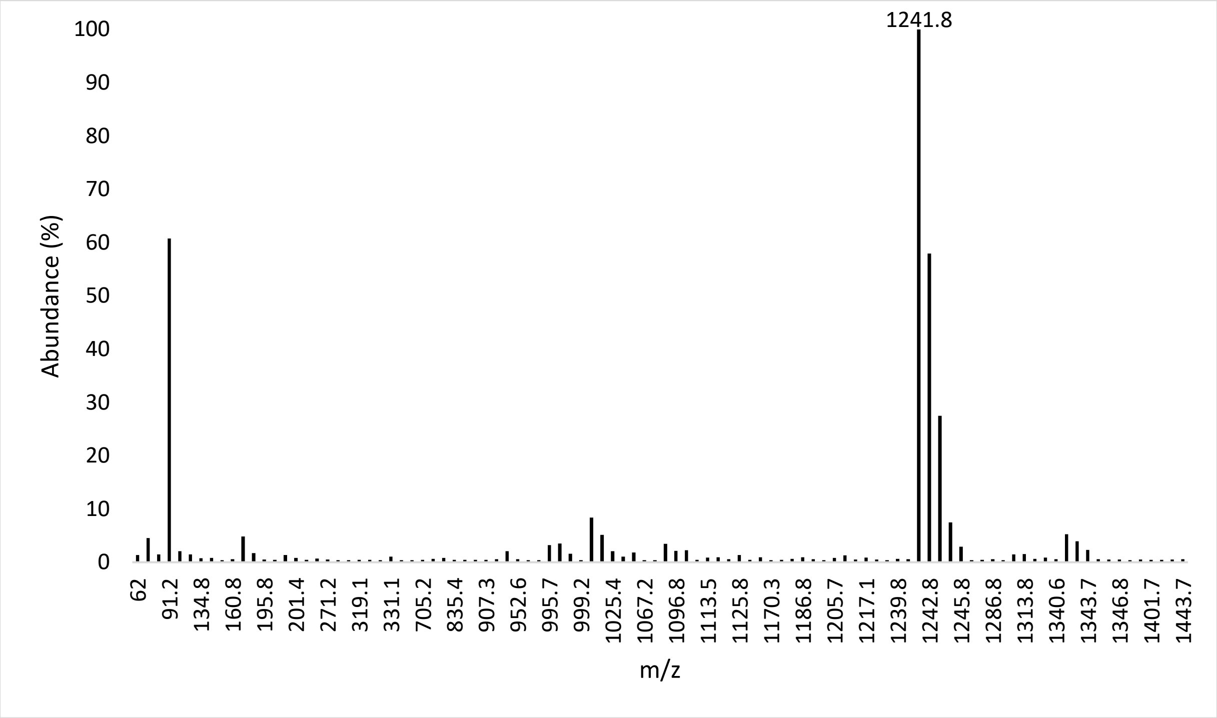

Figure S125. MS spectrum extracted from LA control chromatogram at retention time 28.9 min. Labeled m/z signal represents 17LA.

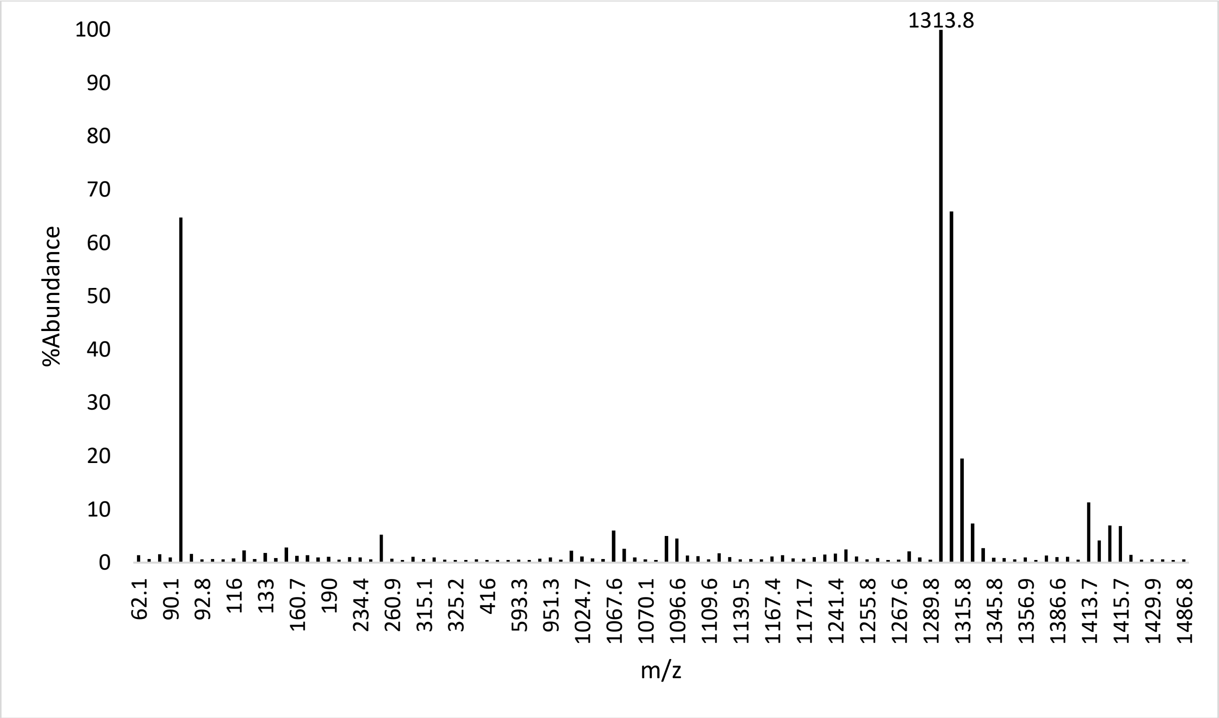

Figure S126. MS spectrum extracted from LA control chromatogram at retention time 29.1 min. Labeled m/z signal represents 18LA.

Table S7. Identification of GA reaction products (in the absence of DA). The detected products based on retention time and their corresponding m/z and ionization pattern as determined by LC-MS.

Retention time (min)	Compound	M (g/mol)	Corresponding m/z (-TIC)	Ionization pattern
**13.1**	4GA	250.1	249.1, 499.1	$[M-H]^-$, $[2M-H]^-$
**14.2**	5GA	308.2	307.1, 615.3	$[M-H]^-$, $[2M-H]^-$
**15.2**	6GA	366.2	365.1, 731.4	$[M-H]^-$, $[2M-H]^-$
**16.2**	7GA	424.2	423.2, 847.3	$[M-H]^-$, $[2M-H]^-$
**17.0**	8GA	482.3	481.2, 963.5	$[M-H]^-$, $[2M-H]^-$
**17.8**	9GA	540.3	539.2, 1079.5	$[M-H]^-$, $[2M-H]^-$
**18.5**	10GA	598.3	597.2, 1195.6	$[M-H]^-$, $[2M-H]^-$
**19.2**	11GA	656.4	655.3, 1311.7	$[M-H]^-$, $[2M-H]^-$
**19.7**	12GA	714.4	713.1, 1427.5	$[M-H]^-$, $[2M-H]^-$
**20.2**	13GA	772.4	771.1	$[M-H]^-$
**20.7**	14GA	830.4	829.3	$[M-H]^-$

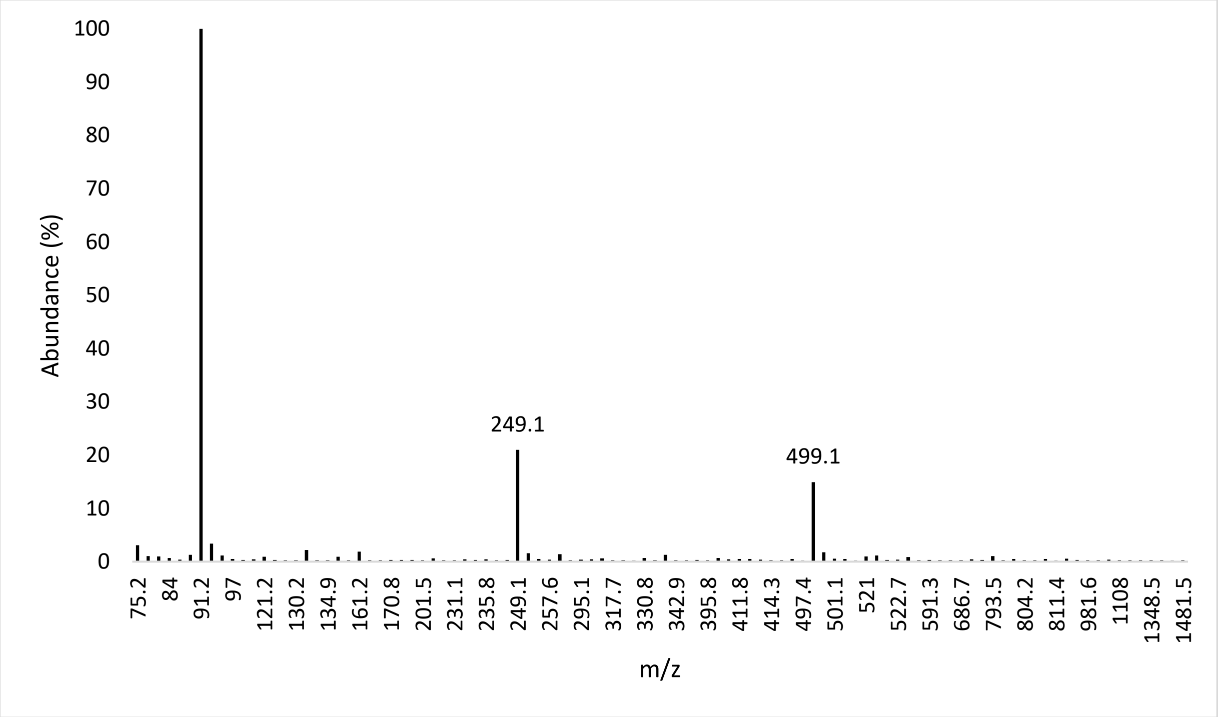

Figure S127. MS spectrum extracted from GA control chromatogram at retention time 13.1 min. Labeled m/z signals represent 4GA.

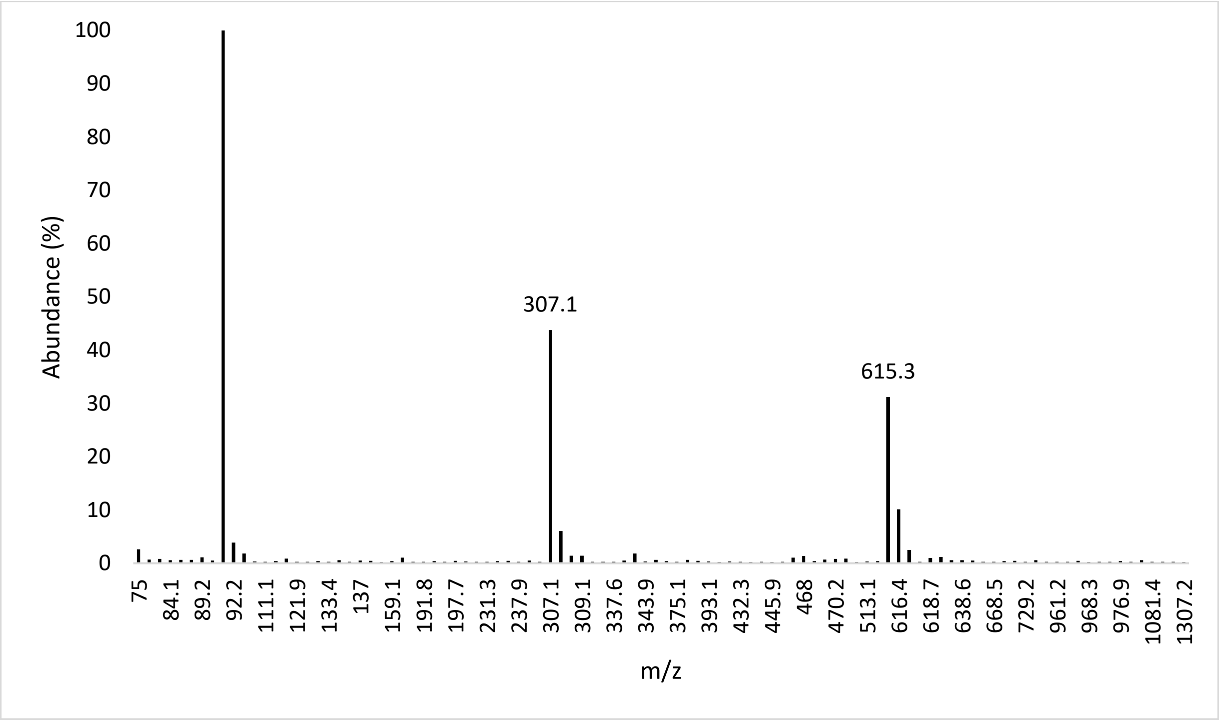

Figure S128. MS spectrum extracted from GA control chromatogram at retention time 14.2 min. Labeled m/z signals represent 5GA.

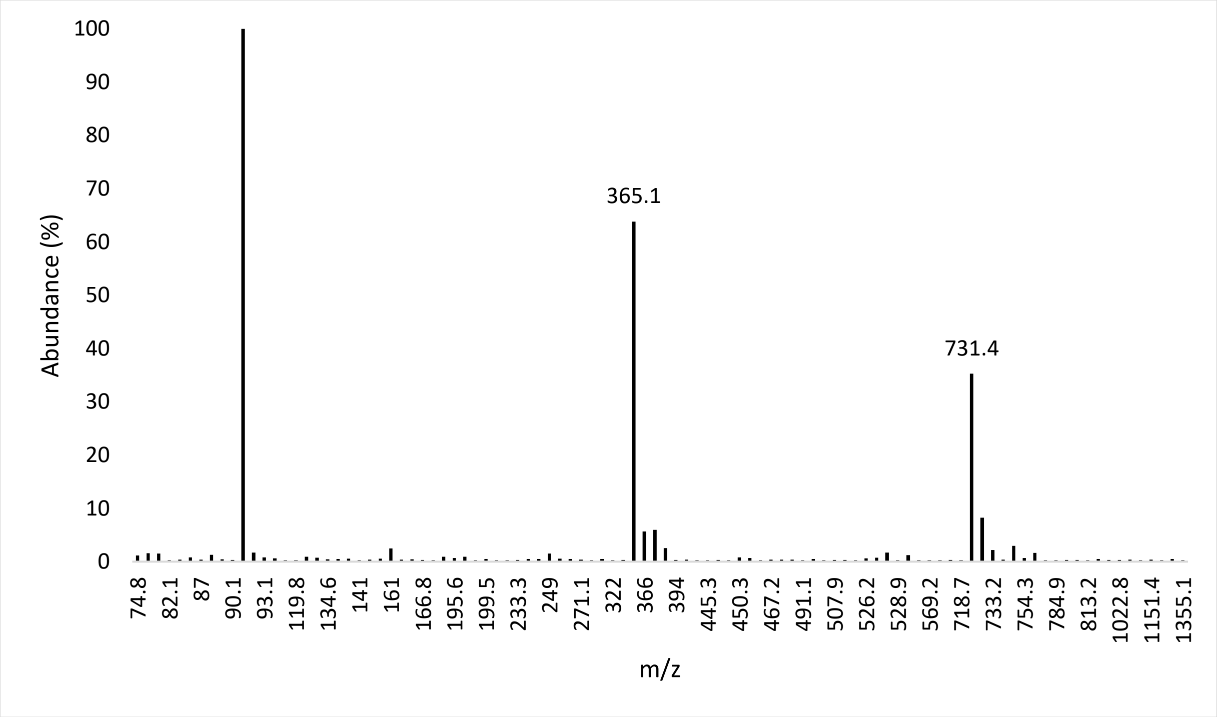

Figure S129. MS spectrum extracted from GA control chromatogram at retention time 15.2 min. Labeled m/z signals represent 6GA.

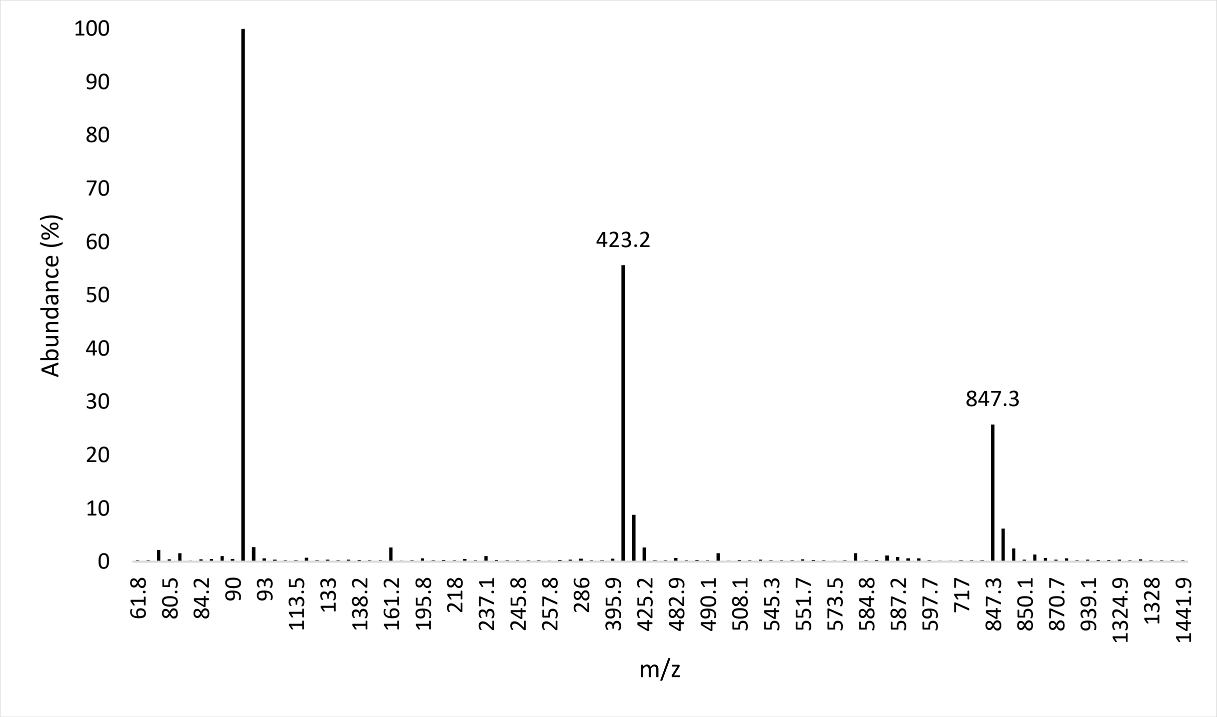

Figure S130. MS spectrum extracted from GA control chromatogram at retention time 16.2 min. Labeled m/z signals represent 7GA.

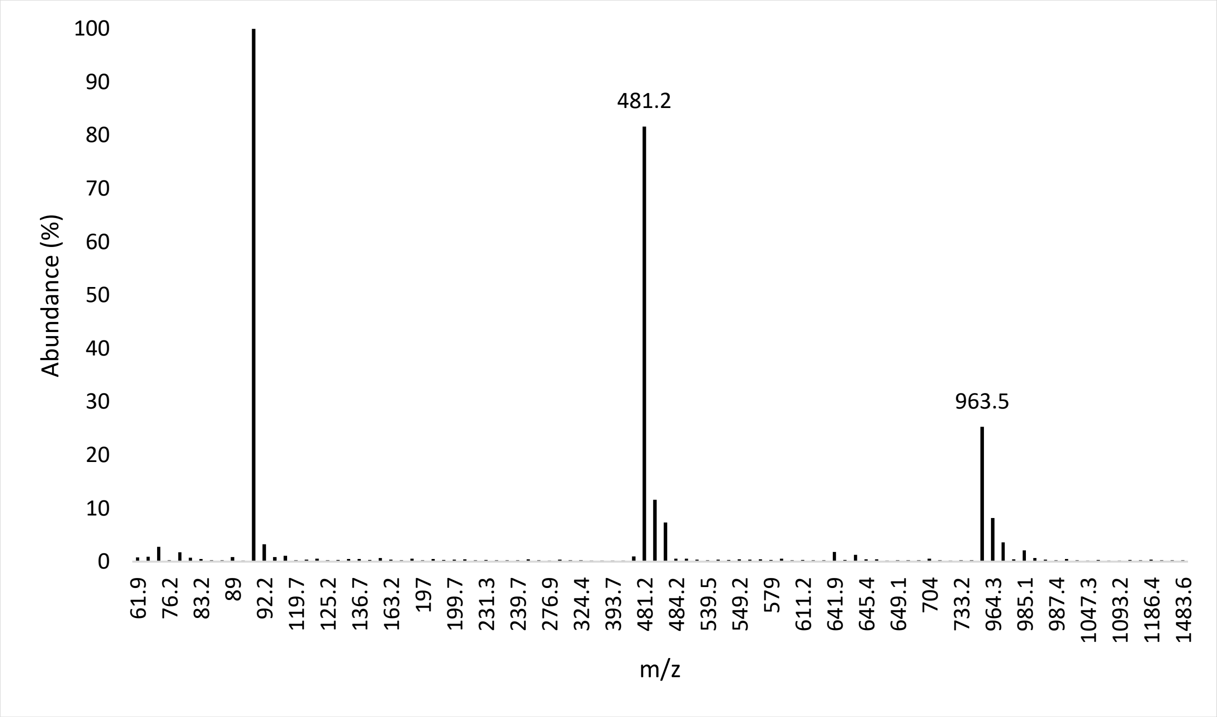

Figure S131. MS spectrum extracted from GA control chromatogram at retention time 17.0 min. Labeled m/z signals represent 8GA.

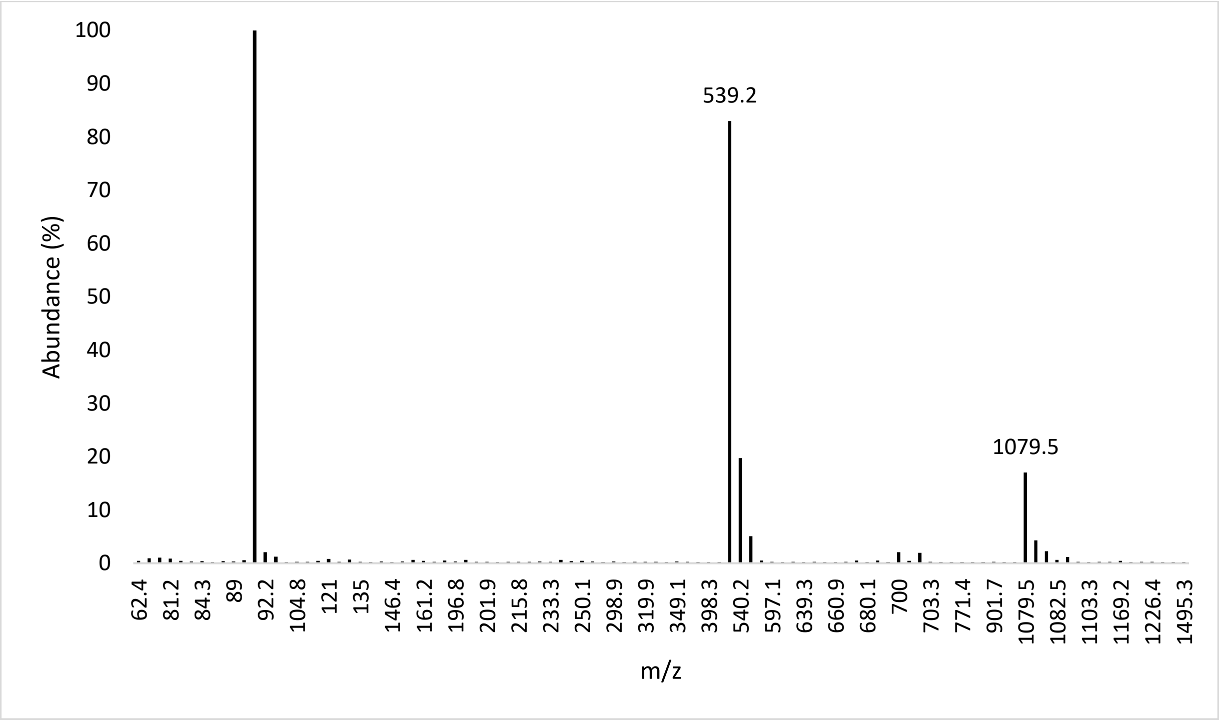

Figure S132. MS spectrum extracted from GA control chromatogram at retention time 17.8 min. Labeled m/z signals represent 9GA.

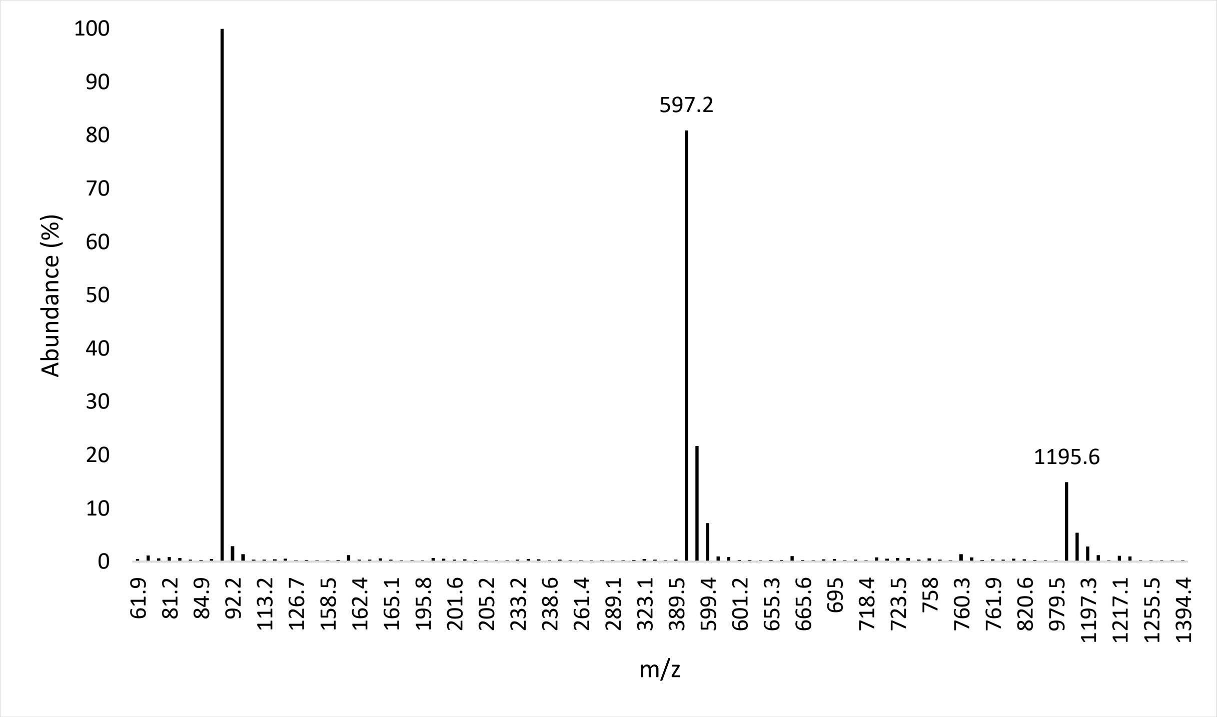

Figure S133. MS spectrum extracted from GA control chromatogram at retention time 18.5 min. Labeled m/z signals represent 10GA.

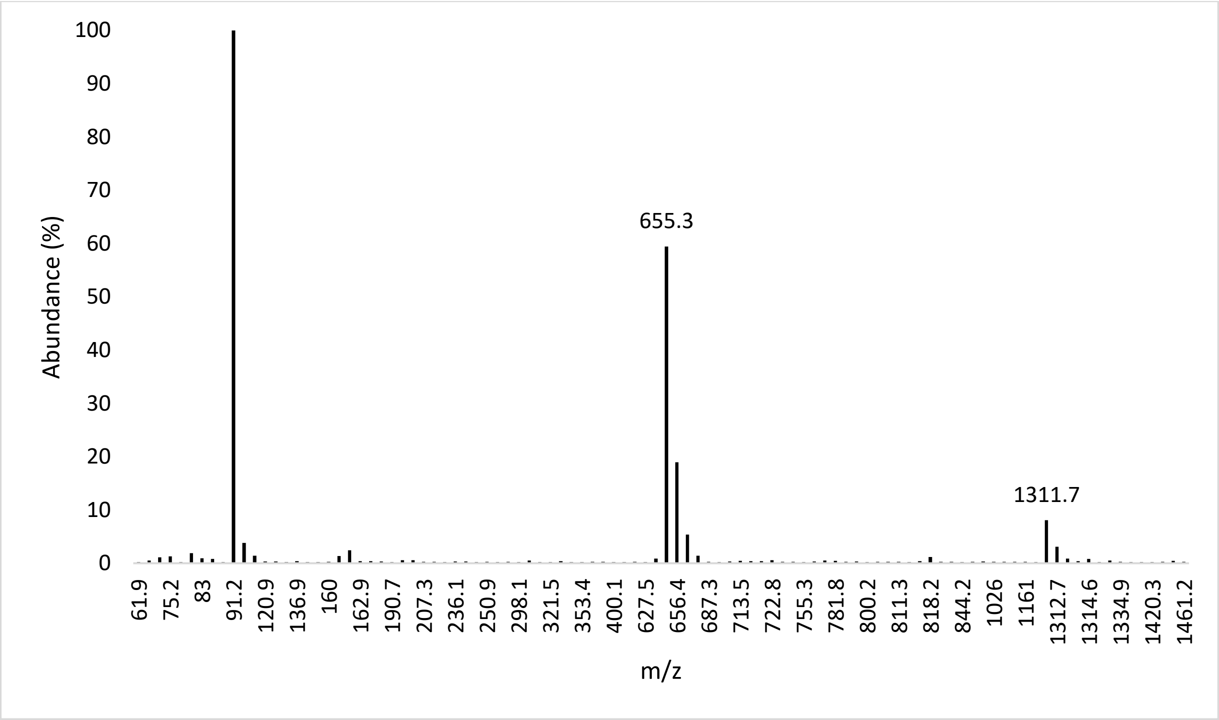

Figure S134. MS spectrum extracted from GA control chromatogram at retention time 19.2 min. Labeled m/z signals represent 11GA.

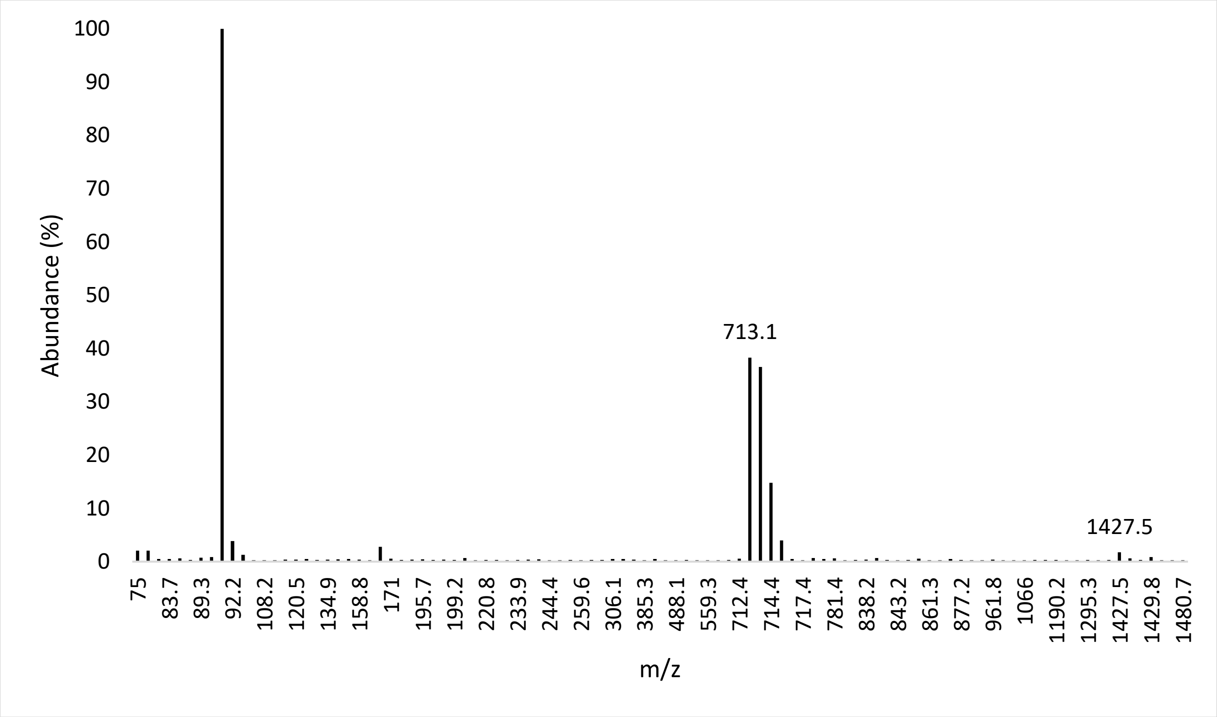

Figure S135. MS spectrum extracted from GA control chromatogram at retention time 19.7 min. Labeled m/z signals represent 12GA.

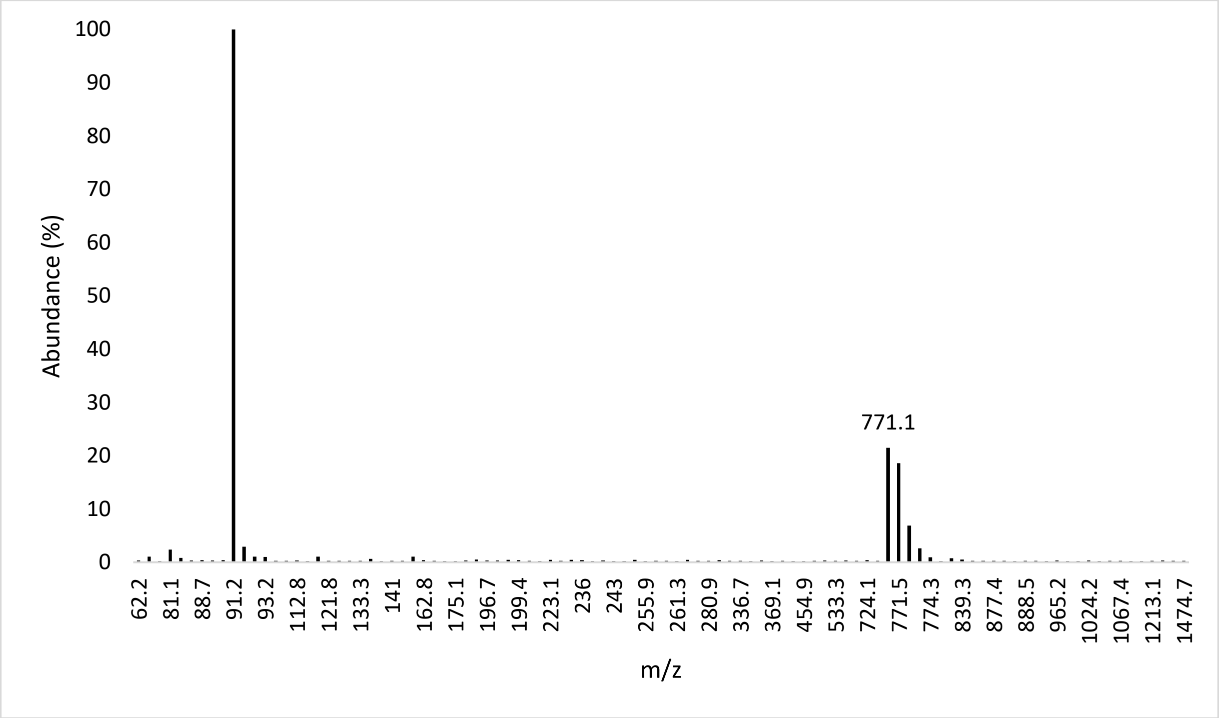

Figure S136. MS spectrum extracted from GA control chromatogram at retention time 20.2 min. Labeled m/z signal represents 13GA.

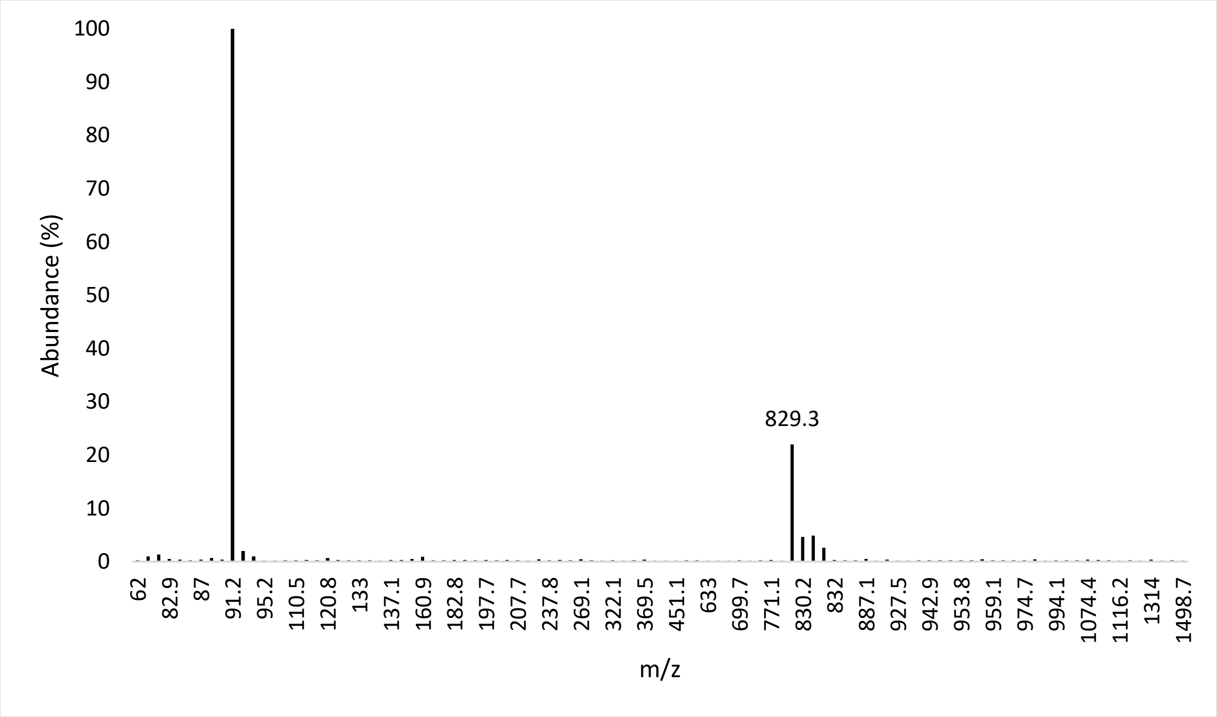

Figure S137. MS spectrum extracted from GA control chromatogram at retention time 20.7 min. Labeled m/z signal represents 14GA.

Table S8. Identification of MA reaction products (in the absence of DA). The detected products based on retention time and their corresponding m/z and ionization pattern as determined by LC-MS.

Retention time (min)	Compound	M (g/mol)	Corresponding m/z (-TIC)	Ionization pattern
**7.7**	2MA	250.2	249.0	$[M-H]^-$
**8.7**	2MA	250.2	249.1	$[M-H]^-$
**11.3**	3MA	366.2	365.2, 731.1	$[M-H]^-$, $[2M-H]^-$
**12.5**	3MA	366.2	365.2, 731.3	$[M-H]^-$, $[2M-H]^-$
	4MA	482.3	481.3, 963.4	$[M-H]^-$, $[2M-H]^-$
**12.8**	4MA	482.3	481.2, 963.4	$[M-H]^-$, $[2M-H]^-$
	5MA	598.4	597.4	$[M-H]^-$
**13.1**	4MA	482.3	481.3, 963.3	$[M-H]^-$, $[2M-H]^-$
	5MA	598.4	597.2	$[M-H]^-$
**13.4**	4MA	482.3	481.3	$[M-H]^-$
	5MA	598.4	597.2, 1195.4	$[M-H]^-$, $[2M-H]^-$
	6MA	714.4	713.2	$[M-H]^-$
**13.6**	5MA	598.4	597.3, 1195.5	$[M-H]^-$, $[2M-H]^-$
	6MA	714.4	713.4, 356.2	$[M-H]^-$, $[M-2H]^{2-}$
**13.9**	5MA	598.4	597.3	$[M-H]^-$
	6MA	714.4	713.3, 356.2	$[M-H]^-$, $[M-2H]^{2-}$
	7MA	830.5	829.3	$[M-H]^-$
**14.1**	6MA	714.4	713.3, 356.2	$[M-H]^-$, $[M-2H]^{2-}$
	7MA	830.5	829.3, 414.2	$[M-H]^-$, $[M-2H]^{2-}$
	8MA	946.6	945.5, 472.2	$[M-H]^-$, $[M-2H]^{2-}$
**14.5**	7MA	830.5	829.3, 414.0	$[M-H]^-$, $[M-2H]^{2-}$
	8MA	946.6	945.4, 472.2	$[M-H]^-$, $[M-2H]^{2-}$
	9MA	1062.7	1061.5, 530.3	$[M-H]^-$, $[M-2H]^{2-}$
	10MA	1178.7	1177.5, 588.5	$[M-H]^-$, $[M-2H]^{2-}$
**14.9**	8MA	946.6	945.5, 472.2	$[M-H]^-$, $[M-2H]^{2-}$
	9MA	1062.7	1061.4, 530.3	$[M-H]^-$, $[M-2H]^{2-}$
	10MA	1178.7	1177.5, 588.1	$[M-H]^-$, $[M-2H]^{2-}$
	11MA	1294.8	1293.5, 646.2	$[M-H]^-$, $[M-2H]^{2-}$
	12MA	1410.9	704.4	$[M-2H]^{2-}$
**15.0**	9MA	1062.7	530.2	$[M-2H]^{2-}$
	10MA	1178.7	1177.5, 588.3	$[M-H]^-$, $[M-2H]^{2-}$
	11MA	1294.8	1293.4, 646.3	$[M-H]^-$, $[M-2H]^{2-}$
	12MA	1410.9	704.4	$[M-2H]^{2-}$
	13MA	1526.9	762.4	$[M-2H]^{2-}$
**15.6**	12MA	1410.9	704.8	$[M-2H]^{2-}$
	13MA	1526.9	762.6	$[M-2H]^{2-}$
	14MA	1643	820.2	$[M-2H]^{2-}$
	15MA	1759.1	878.6	$[M-2H]^{2-}$
	16MA	1875.1	936.5	$[M-2H]^{2-}$
**16.0**	16MA	1875.1	963.5	$[M-2H]^{2-}$
	17MA	1991.2	994.1	$[M-2H]^{2-}$

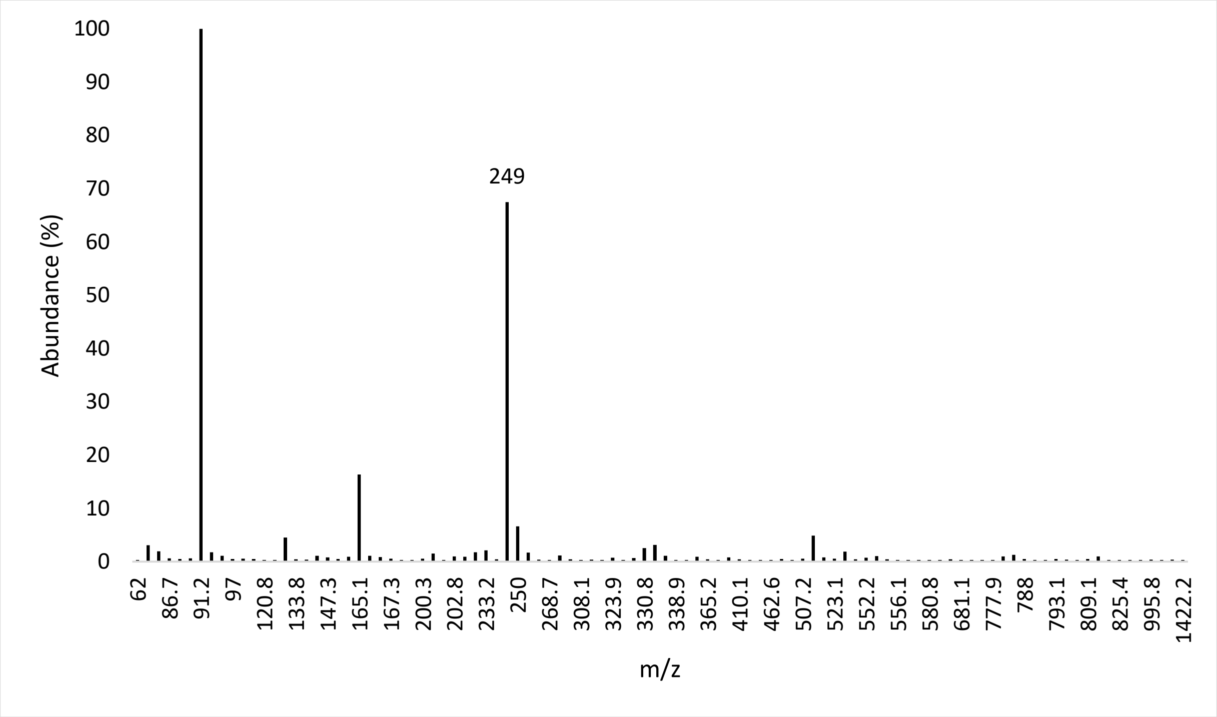

Figure S138. MS spectrum extracted from MA control chromatogram at retention time 7.7 min. Labeled m/z signal represents 2MA.

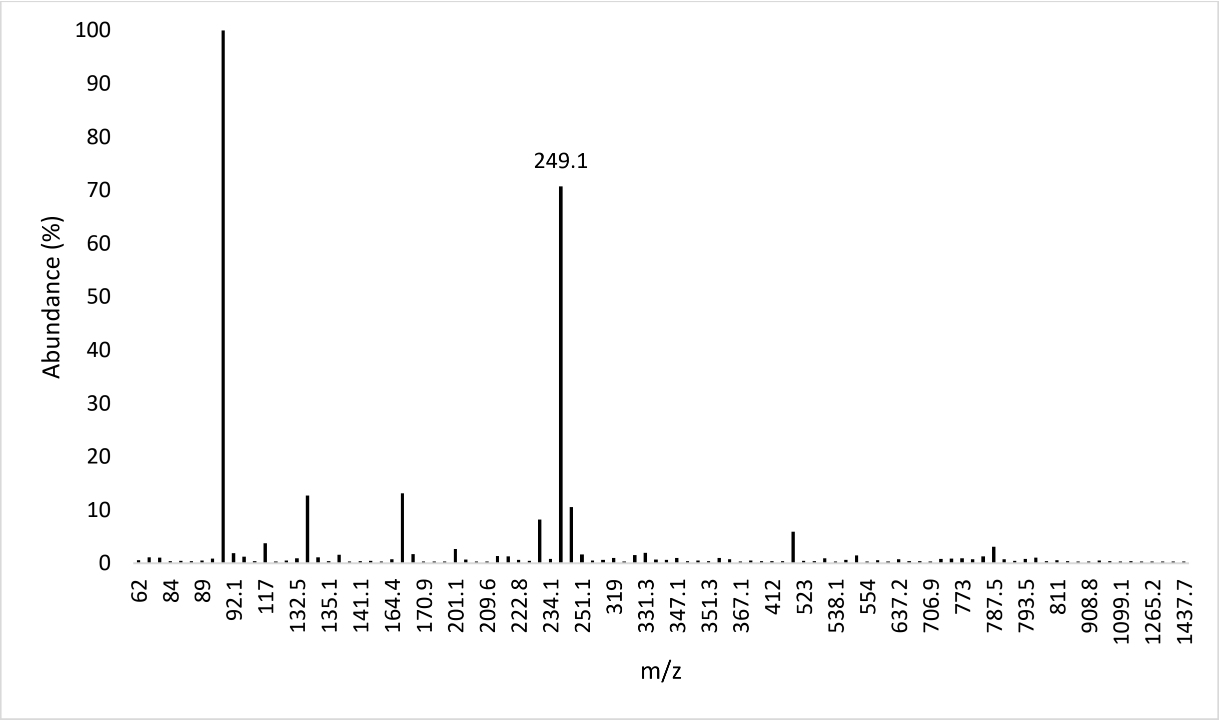

Figure S139. MS spectrum extracted from MA control chromatogram at retention time 8.7 min. Labeled m/z signal represents 2MA.

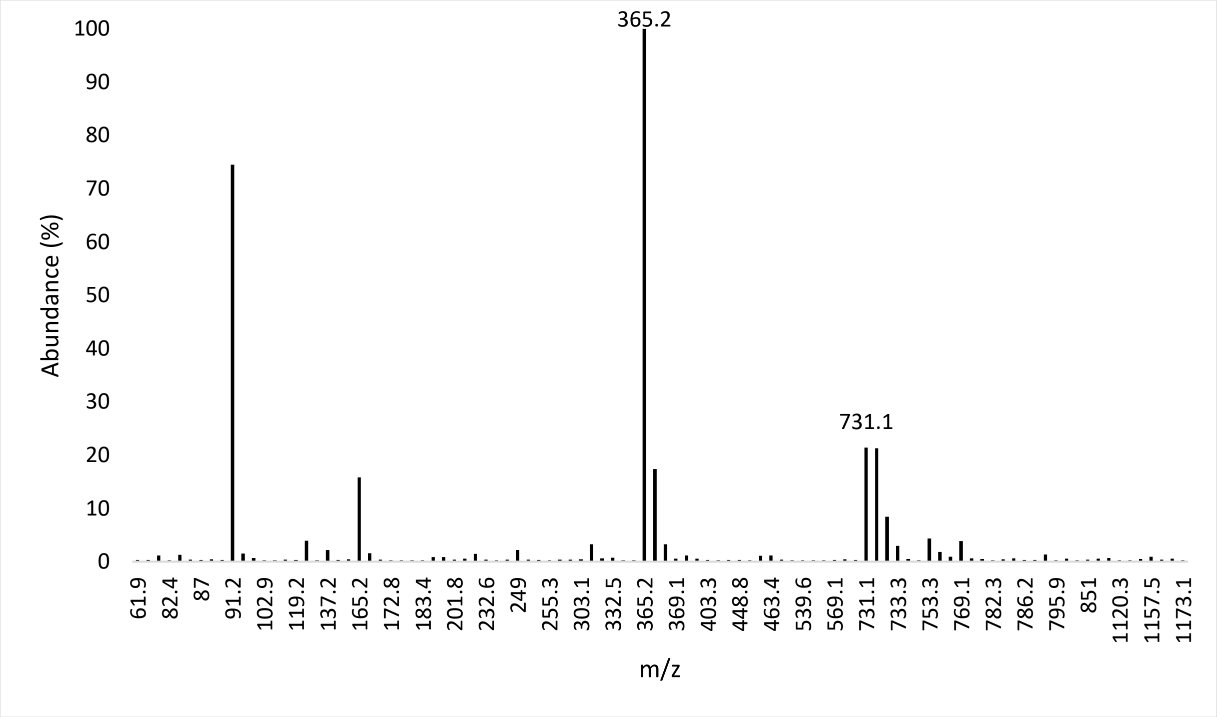

Figure S140. MS spectrum extracted from MA control chromatogram at retention time 11.3 min. Labeled m/z signals represent 3MA.

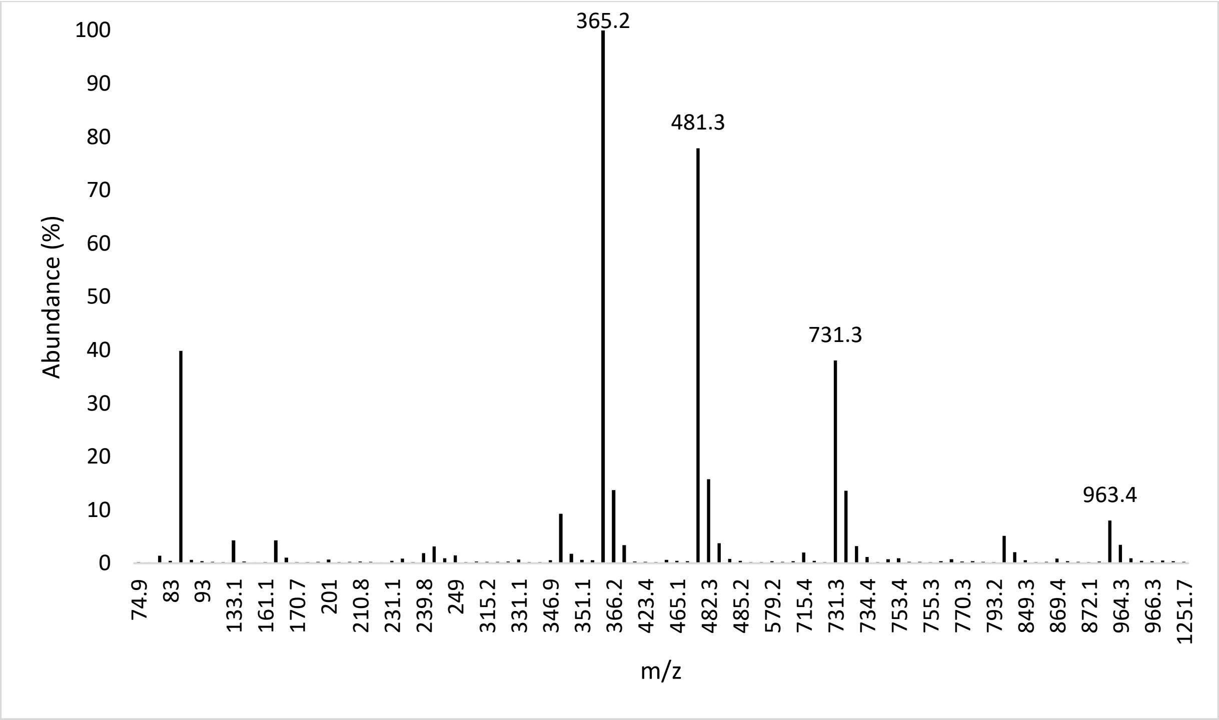

Figure S141. MS spectrum extracted from MA control chromatogram at retention time 12.5 min. Labeled m/z signals represent 3MA and 4MA.

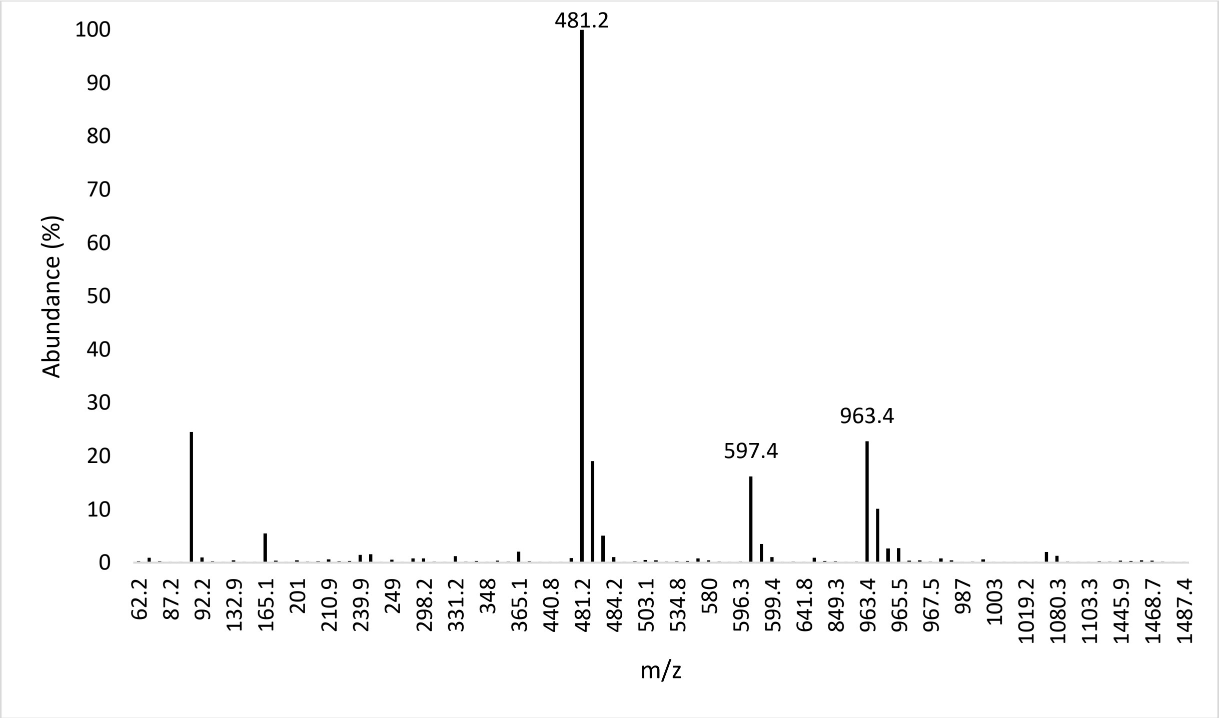

Figure S142. MS spectrum extracted from MA control chromatogram at retention time 12.8 min. Labeled m/z signals represent 4MA and 5MA.

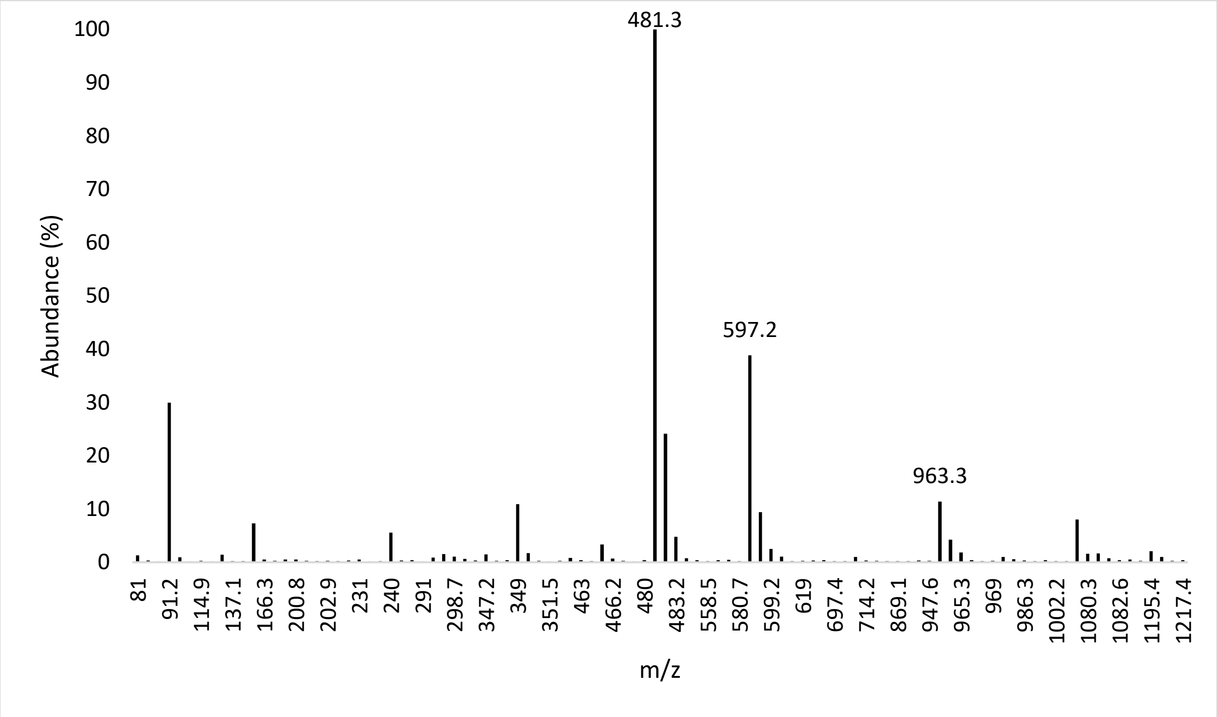

Figure S143. MS spectrum extracted from MA control chromatogram at retention time 13.1 min. Labeled m/z signals represent 4MA and 5MA.

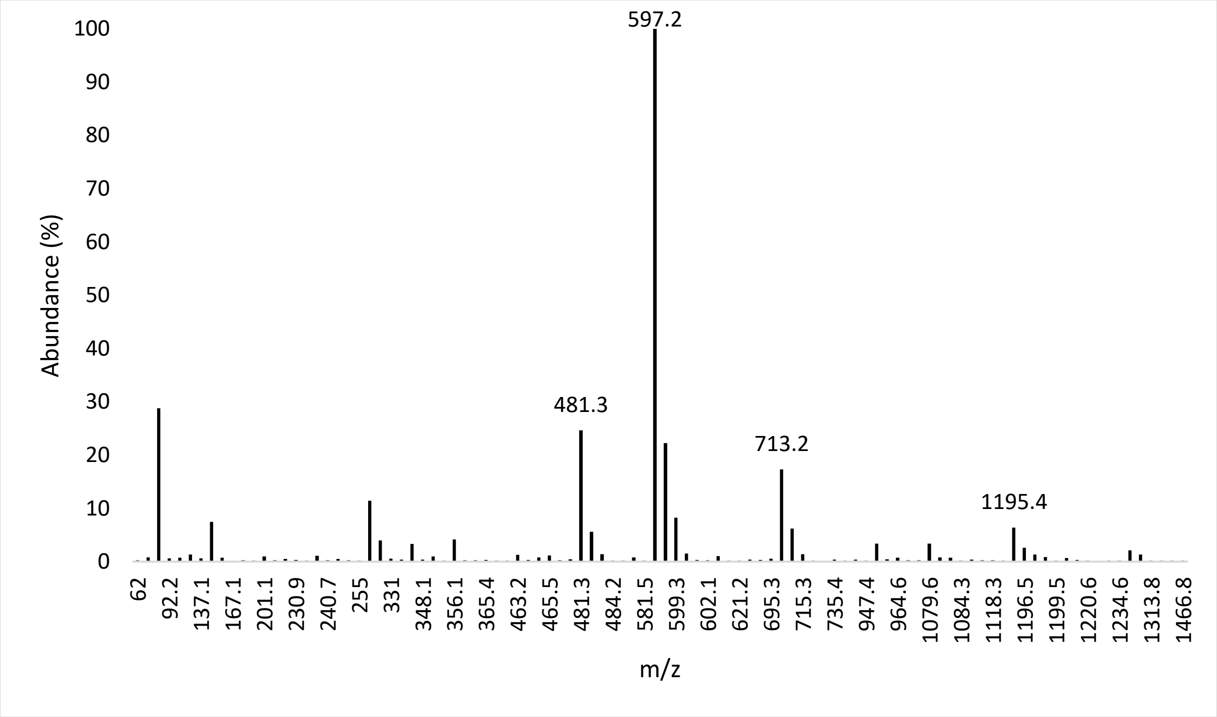

Figure S144. MS spectrum extracted from MA control chromatogram at retention time 13.4 min. Labeled m/z signals represent 4MA, 5MA and 6MA.

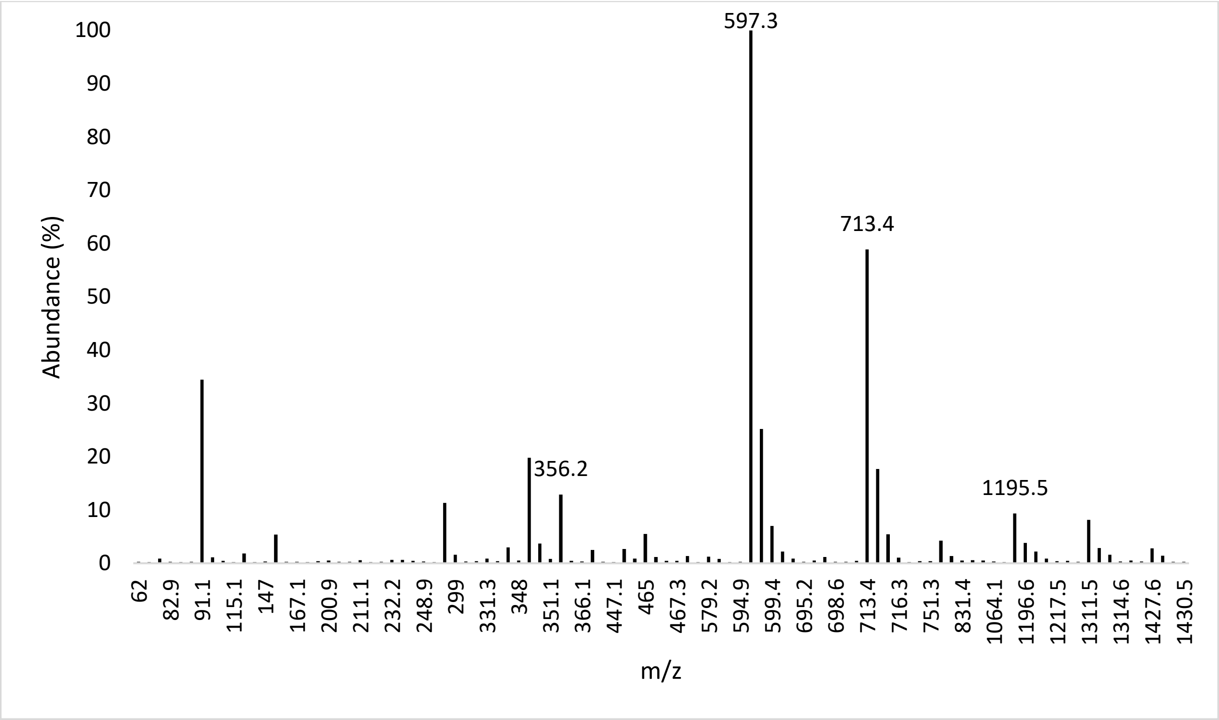

Figure S145. MS spectrum extracted from MA control chromatogram at retention time 13.6 min. Labeled m/z signals represent 5MA and 6MA.

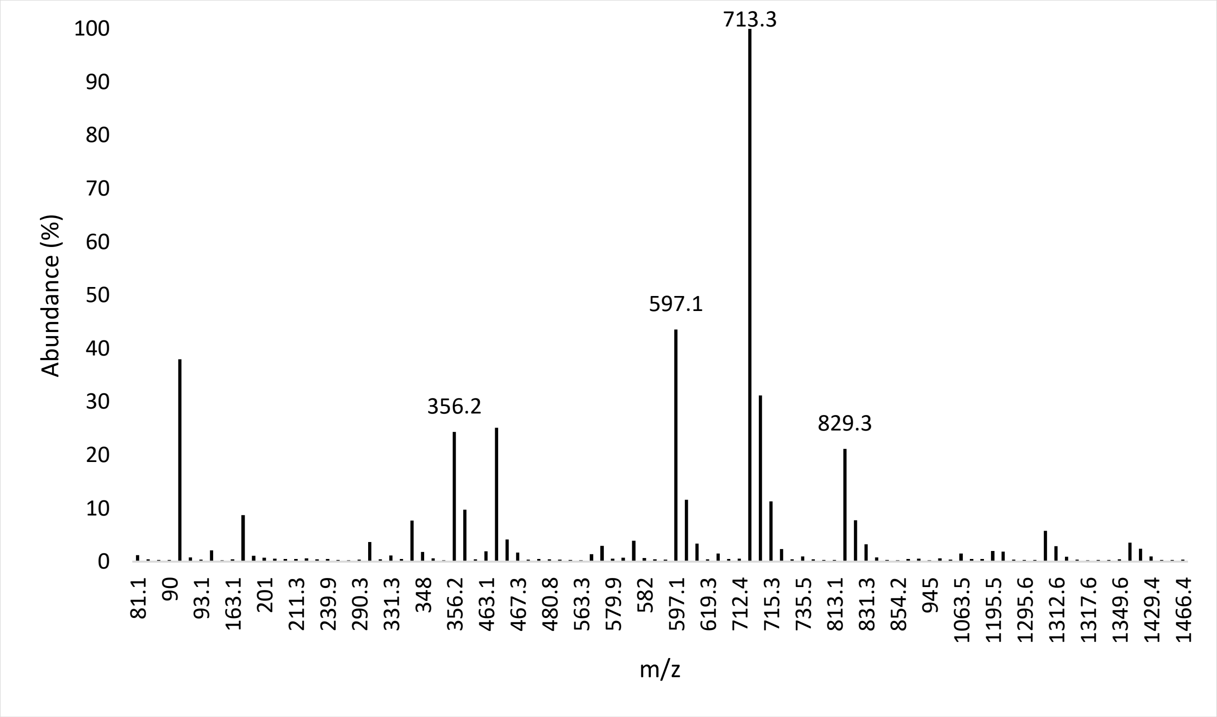

Figure S146. MS spectrum extracted from MA control chromatogram at retention time 13.9 min. Labeled m/z signals represent 5MA, 6MA and 7MA.

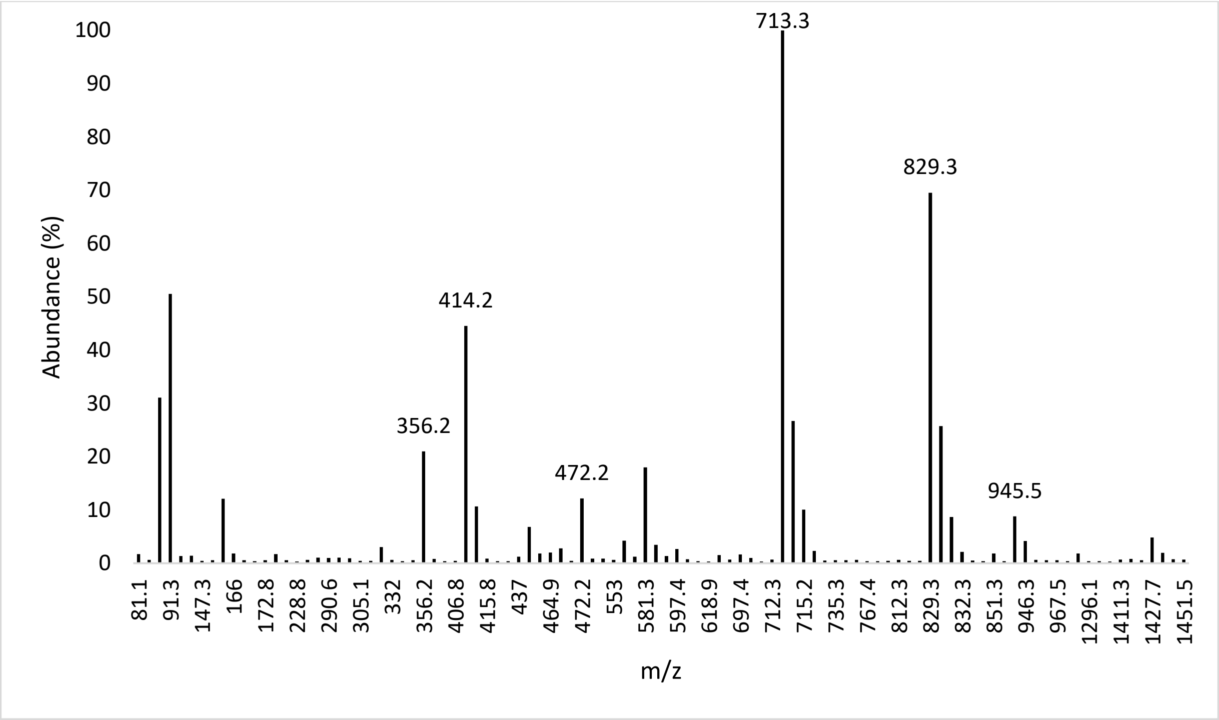

Figure S147. MS spectrum extracted from MA control chromatogram at retention time 14.1 min. Labeled m/z signals represent 6MA, 7MA and 8MA.

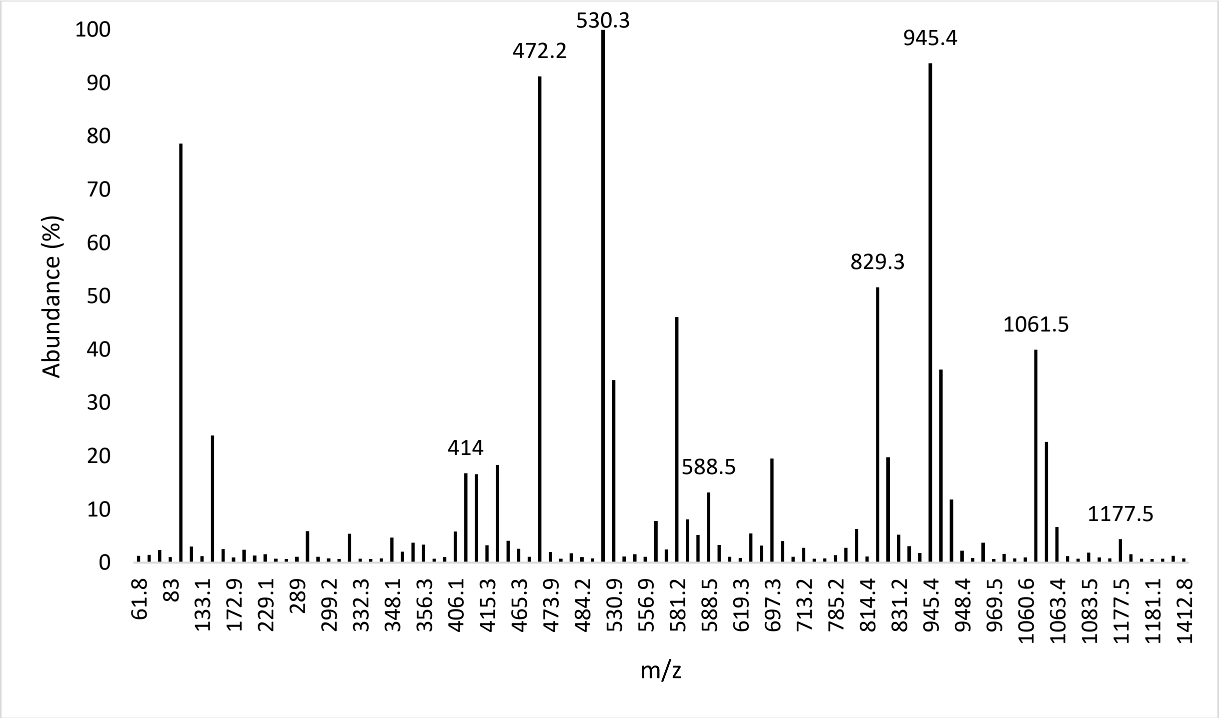

Figure S148. MS spectrum extracted from MA control chromatogram at retention time 14.5 min. Labeled m/z signals represent 7MA, 8MA, 9MA and 10MA.

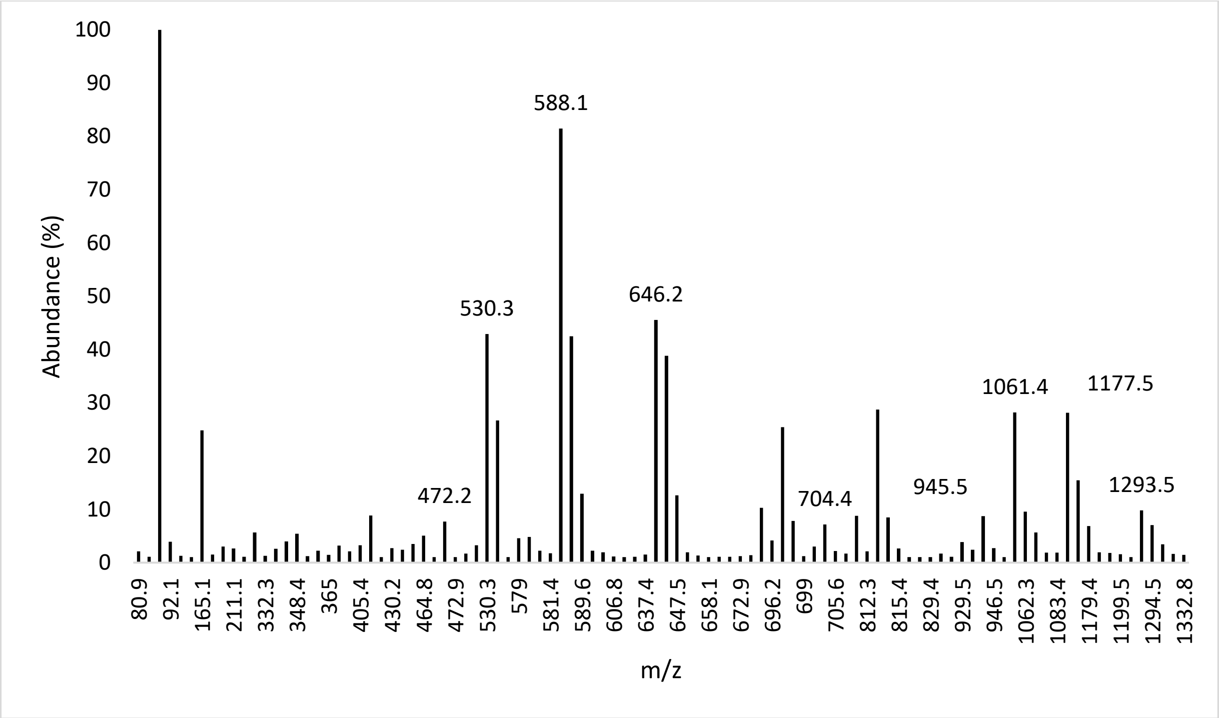

Figure S149. MS spectrum extracted from MA control chromatogram at retention time 14.9 min. Labeled m/z signals represent 8MA, 9MA, 10MA, 11MA and 12MA**.**

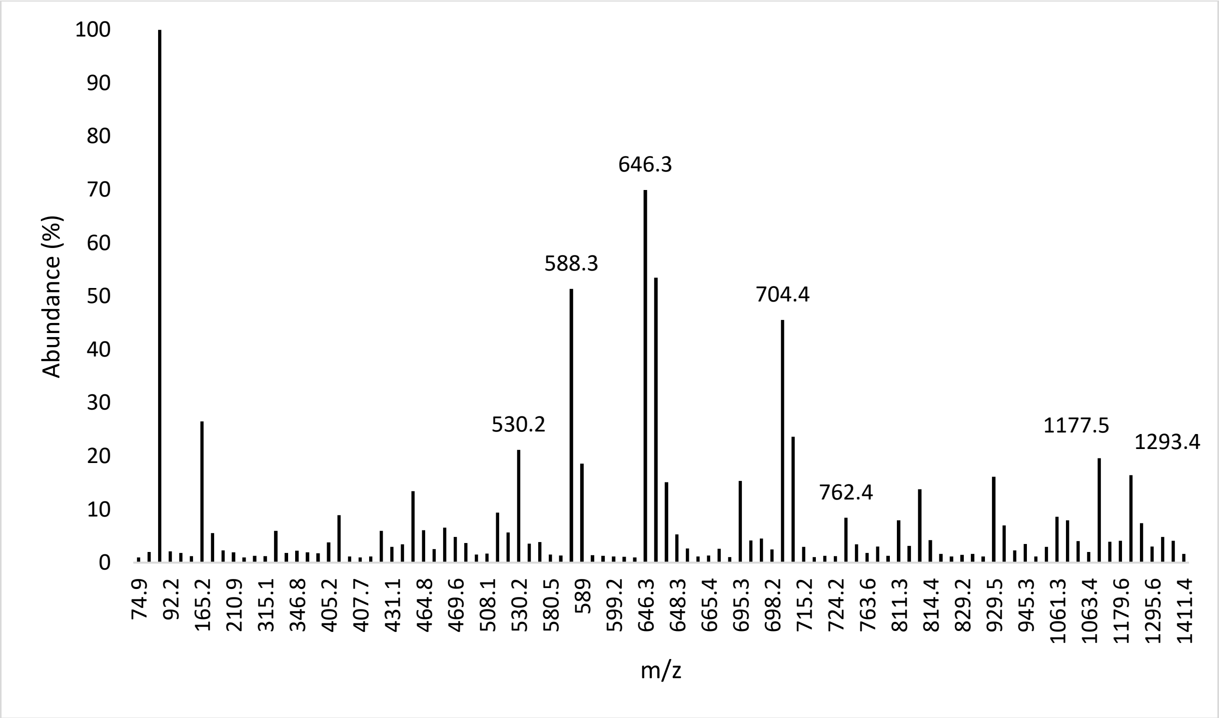

Figure S150. MS spectrum extracted from MA control chromatogram at retention time 15.0 min. Labeled m/z signals represent 9MA, 10MA, 11MA, 12MA and 13MA.

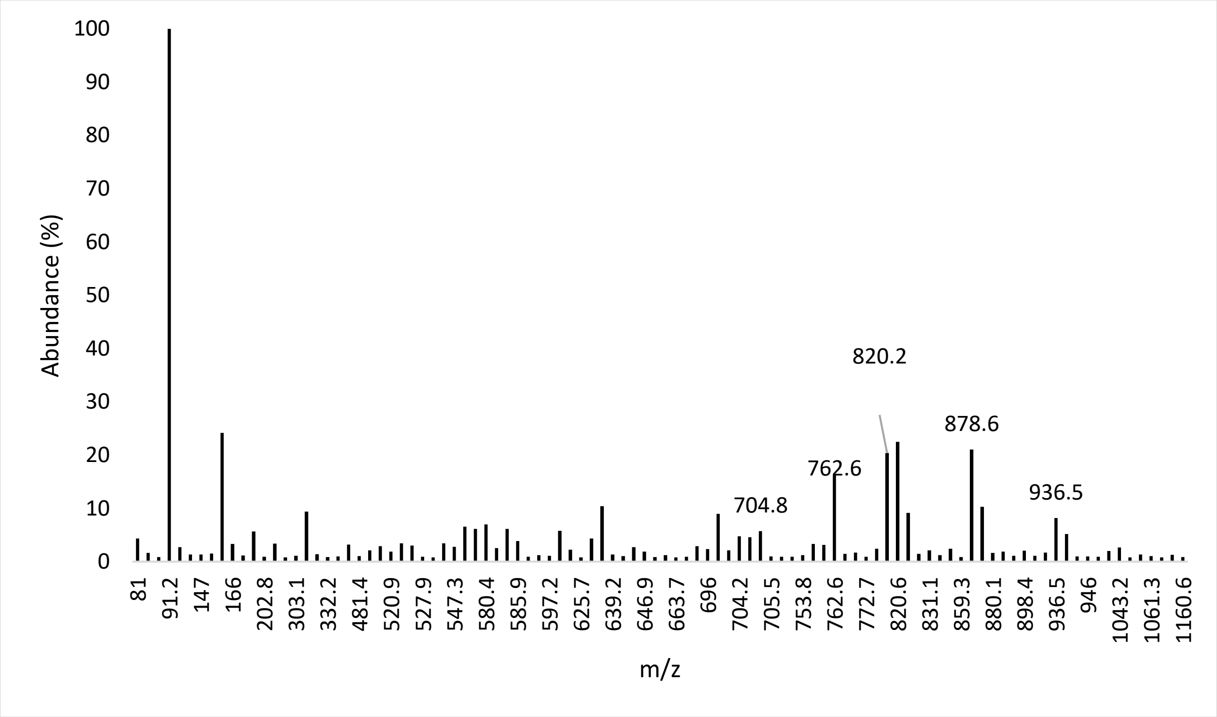

Figure S151. MS spectrum extracted from MA control chromatogram at retention time 15.6 min. Labeled m/z signals represent 12MA, 13MA, 14MA, 15MA and 16MA.

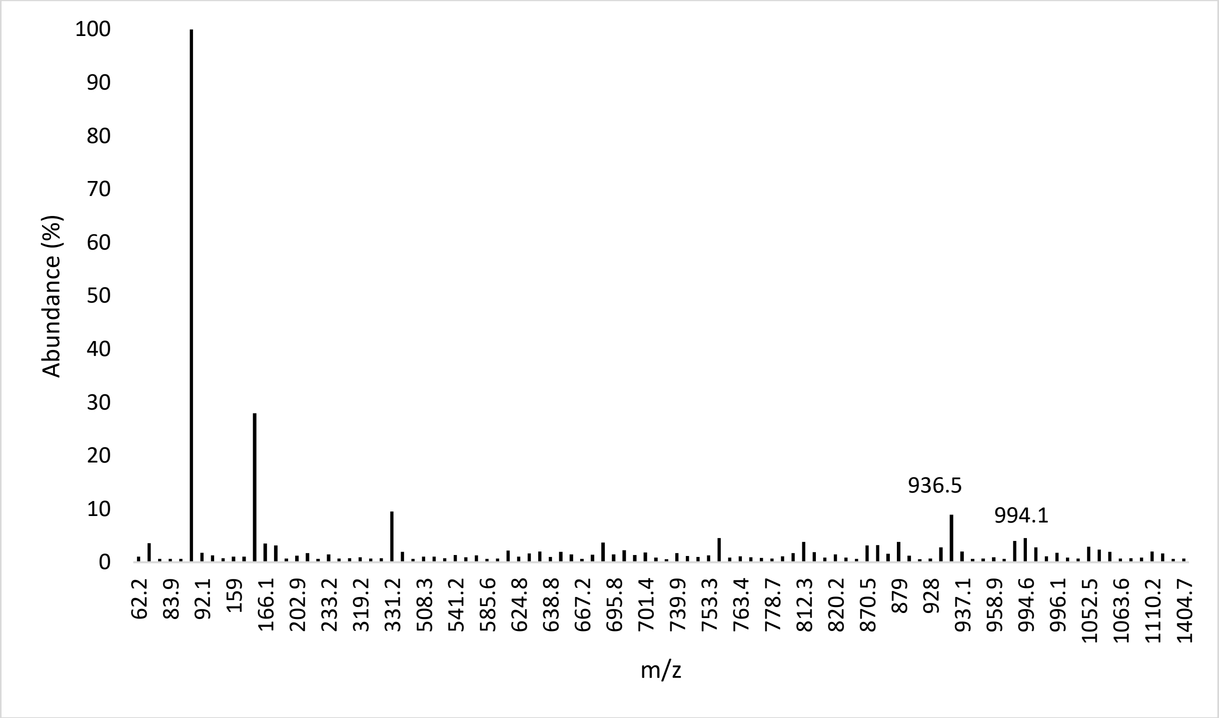

Figure S152. MS spectrum extracted from MA control chromatogram at retention time 16.0 min. Labeled m/z signals represent 16MA and 17MA.

Table S9. Identification of PLA reaction products (in the absence of DA). The detected products based on retention time and their corresponding m/z and ionization pattern as determined by LC-MS.

Retention time (min)	Compound	M (g/mol)	Corresponding m/z (-TIC)	Ionization pattern
**23.1**	2PLA	314.3	313.2, 627.4	$[M-H]^-$, $[2M-H]^-$
**26.7**	3PLA	462.5	461.3, 923.5	$[M-H]^-$, $[2M-H]^-$
**29.0**	4PLA	610.6	609.5, 1219.8	$[M-H]^-$, $[2M-H]^-$
**29.8**	5PLA	758.8	757.4	$[M-H]^-$
**30.6**	6PLA	906.9	905.7	$[M-H]^-$
	7PLA	1055.1	1053.9	$[M-H]^-$

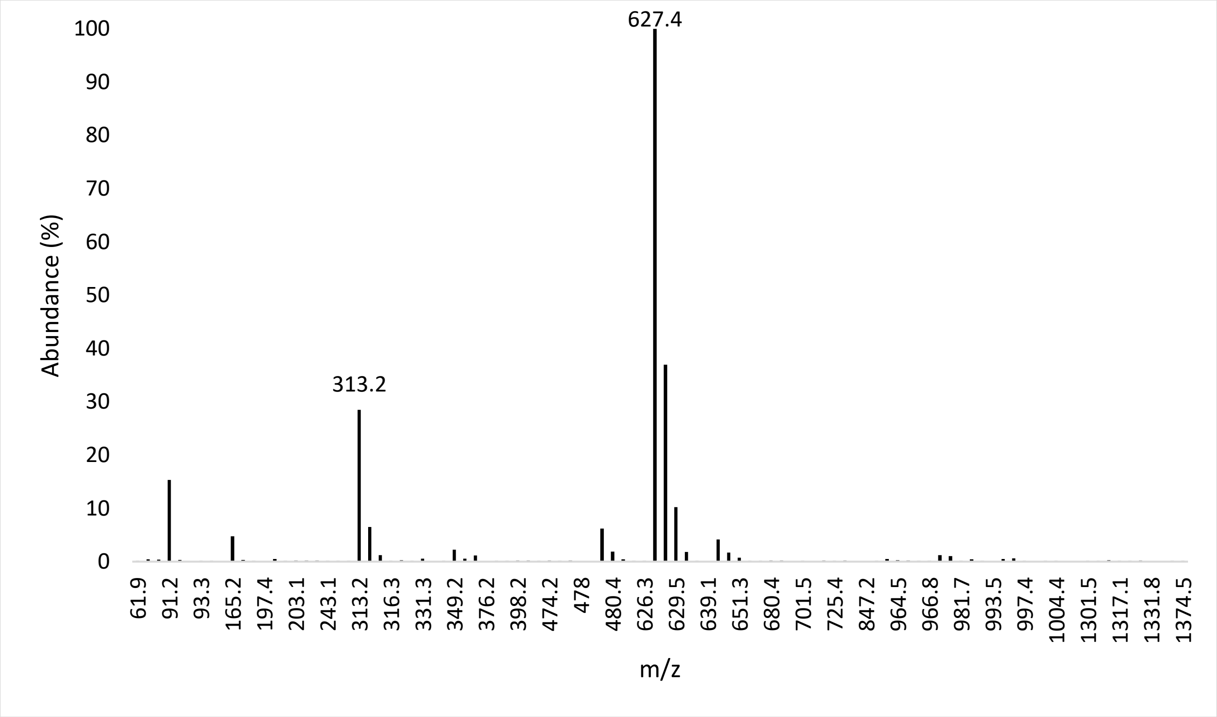

Figure S153. MS spectrum extracted from PLA control chromatogram at retention time 23.1 min. Labeled m/z signals represent 2PLA.

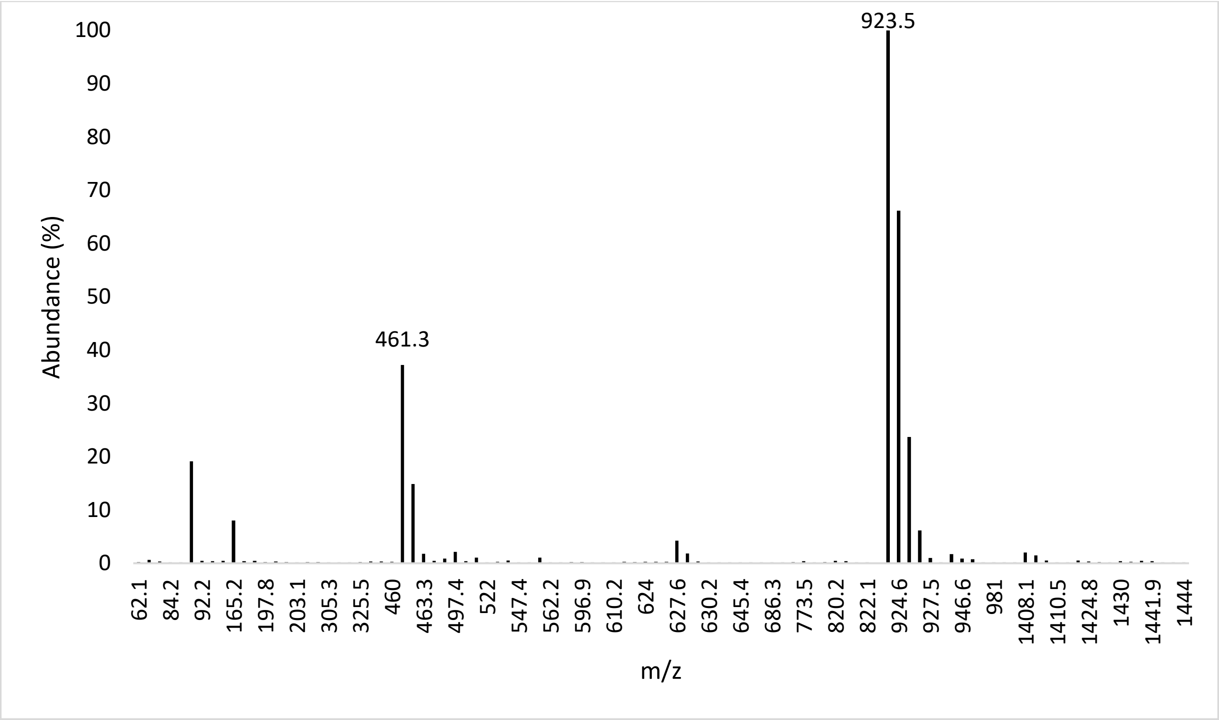

Figure S154. MS spectrum extracted from PLA control chromatogram at retention time 26.7 min. Labeled m/z signals represent 3PLA.

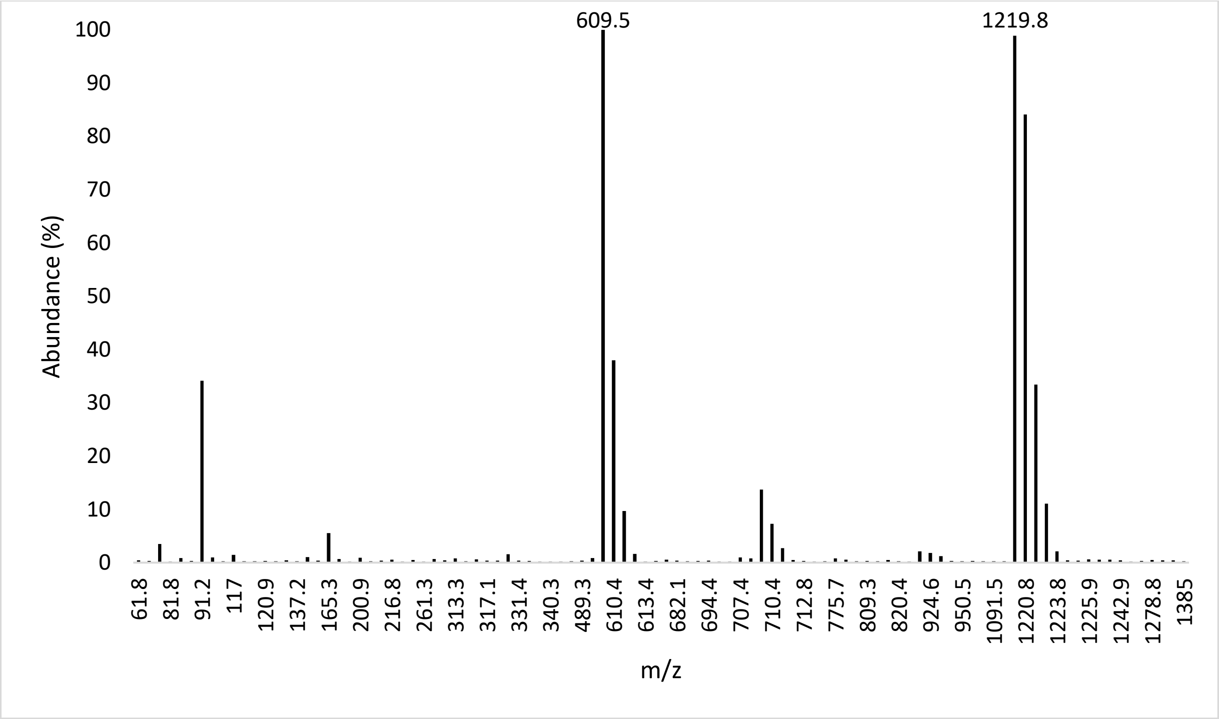

Figure S155. MS spectrum extracted from PLA control chromatogram at retention time 29.0 min. Labeled m/z signals represent 4PLA.

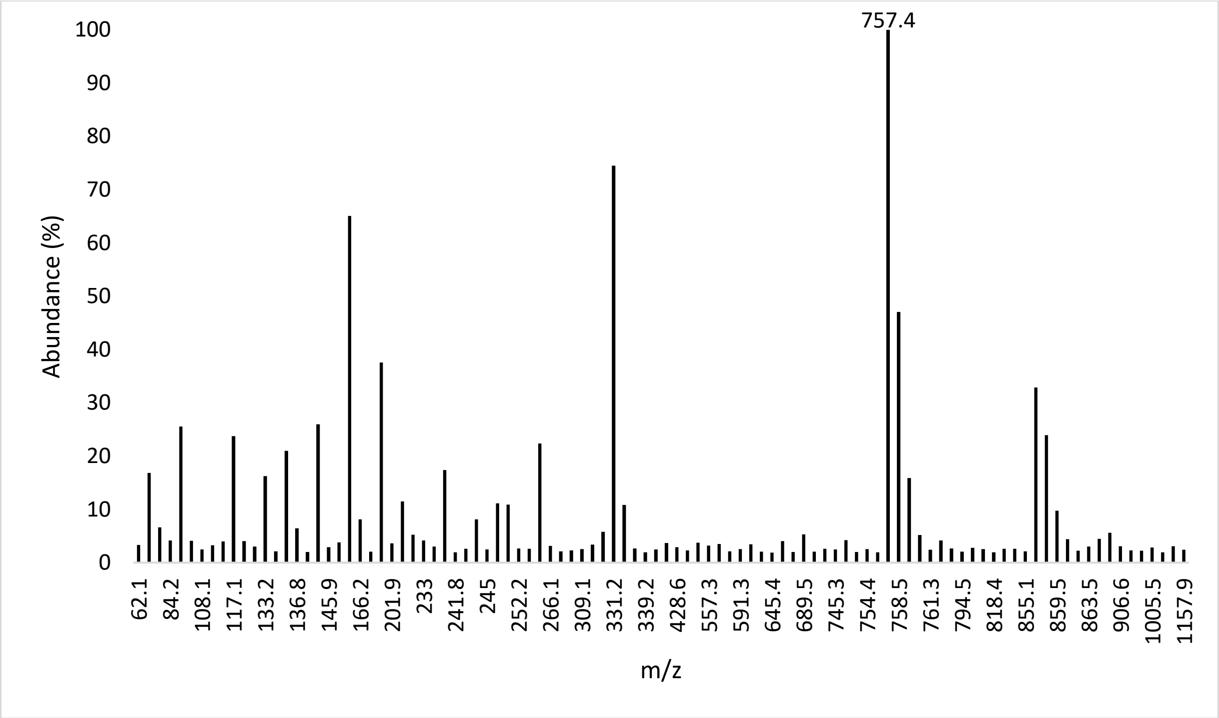

Figure S156. MS spectrum extracted from PLA control chromatogram at retention time 29.8 min. Labeled m/z signal represents 5PLA.

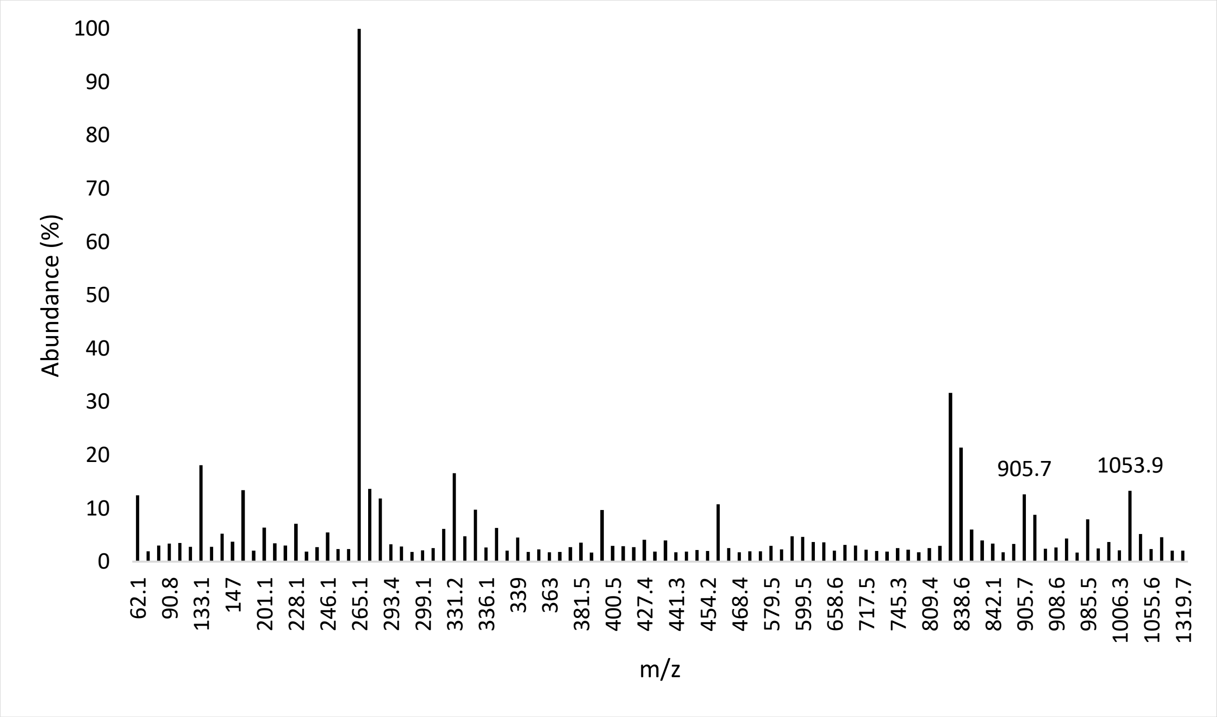

Figure S157. MS spectrum extracted from PLA control chromatogram at retention time 30.6 min. Labeled m/z signals represent 6PLA and 7PLA.

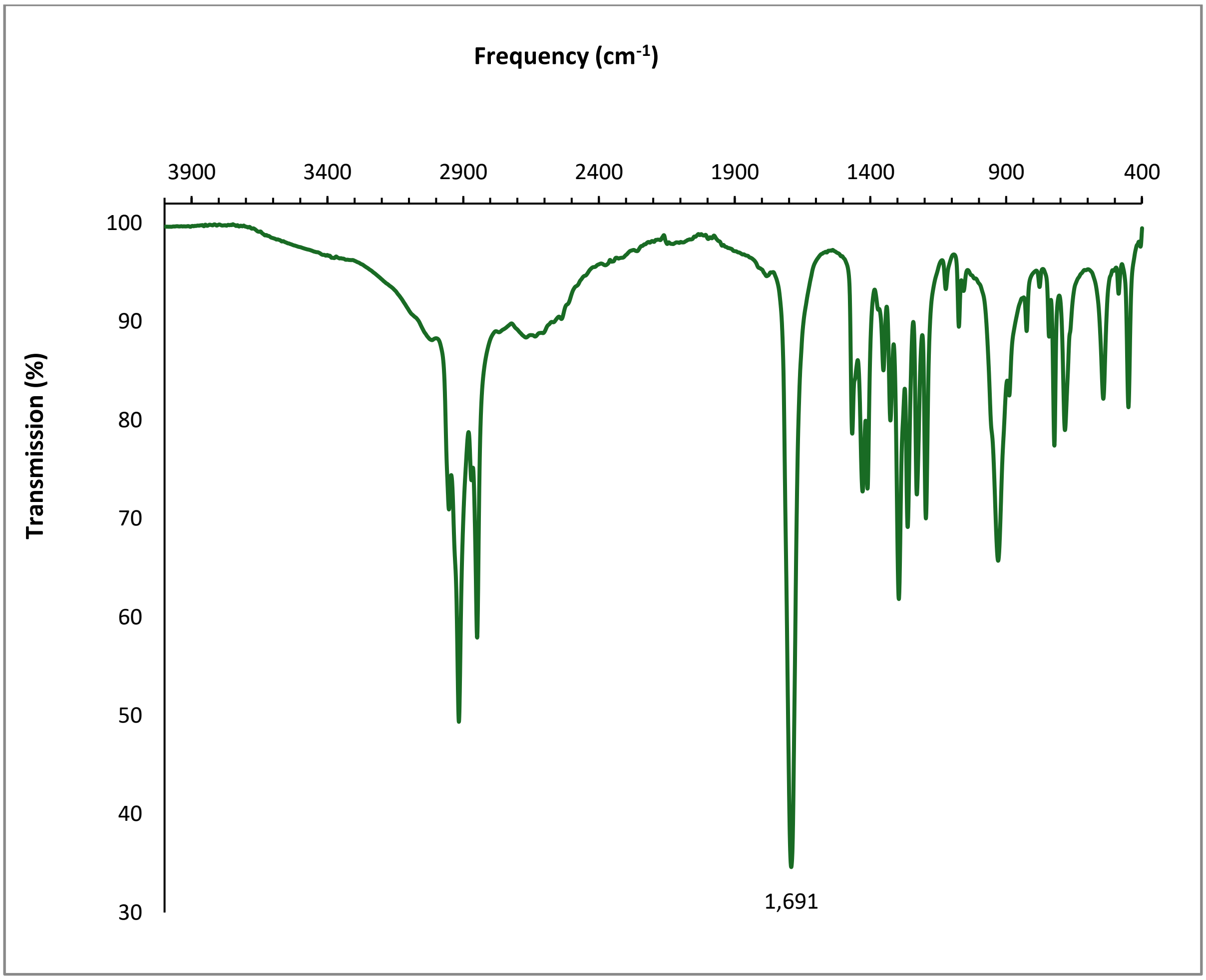

Figure S158. FTIR spectrum of fresh DA monomers. Indicative C=O stretching of the carboxylic acid is assigned at 1691cm^{-1}.

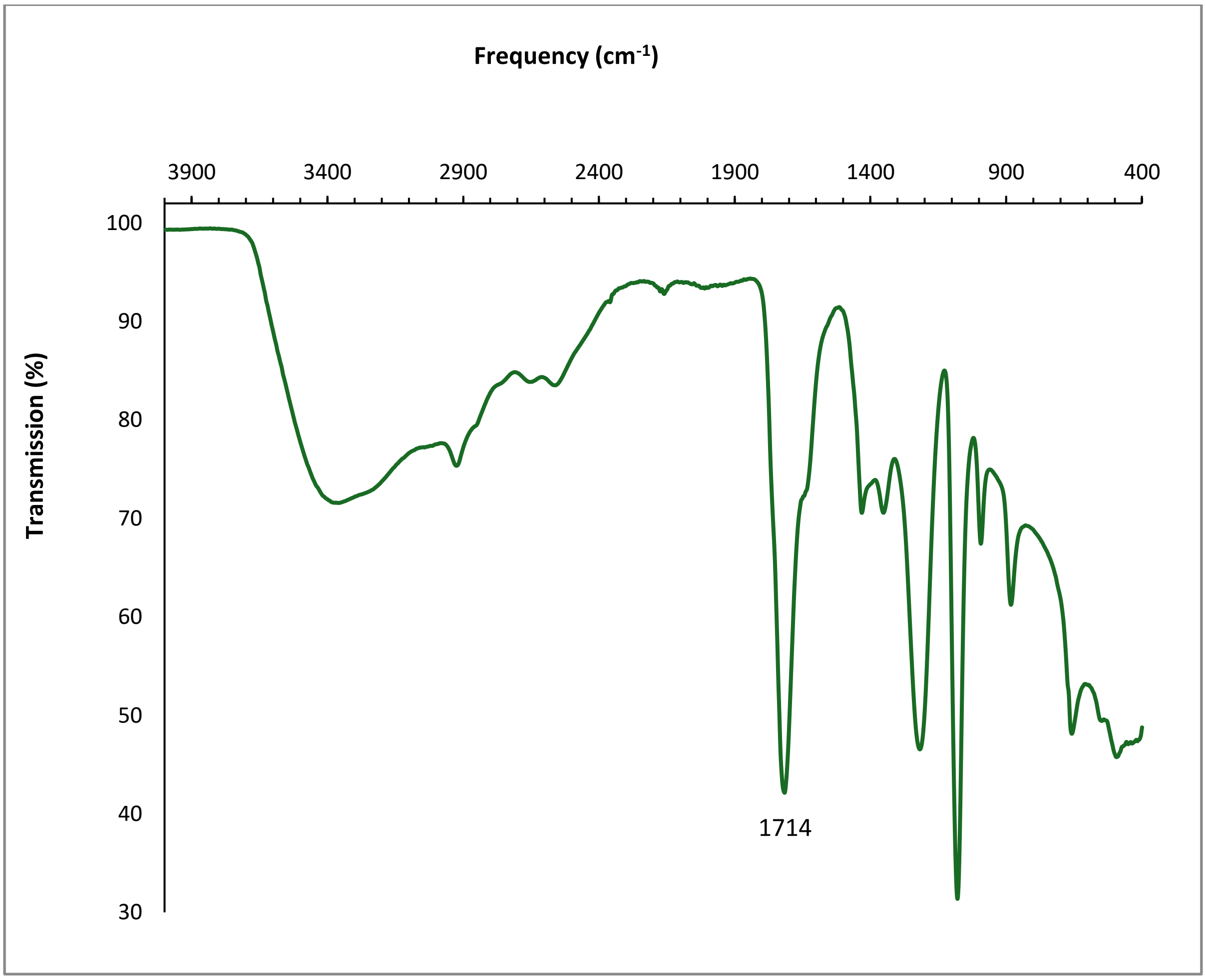

Figure S159. FTIR spectrum of fresh GA monomers. Indicative C=O stretching of the carboxylic acid is assigned at 1714cm^{-1}.

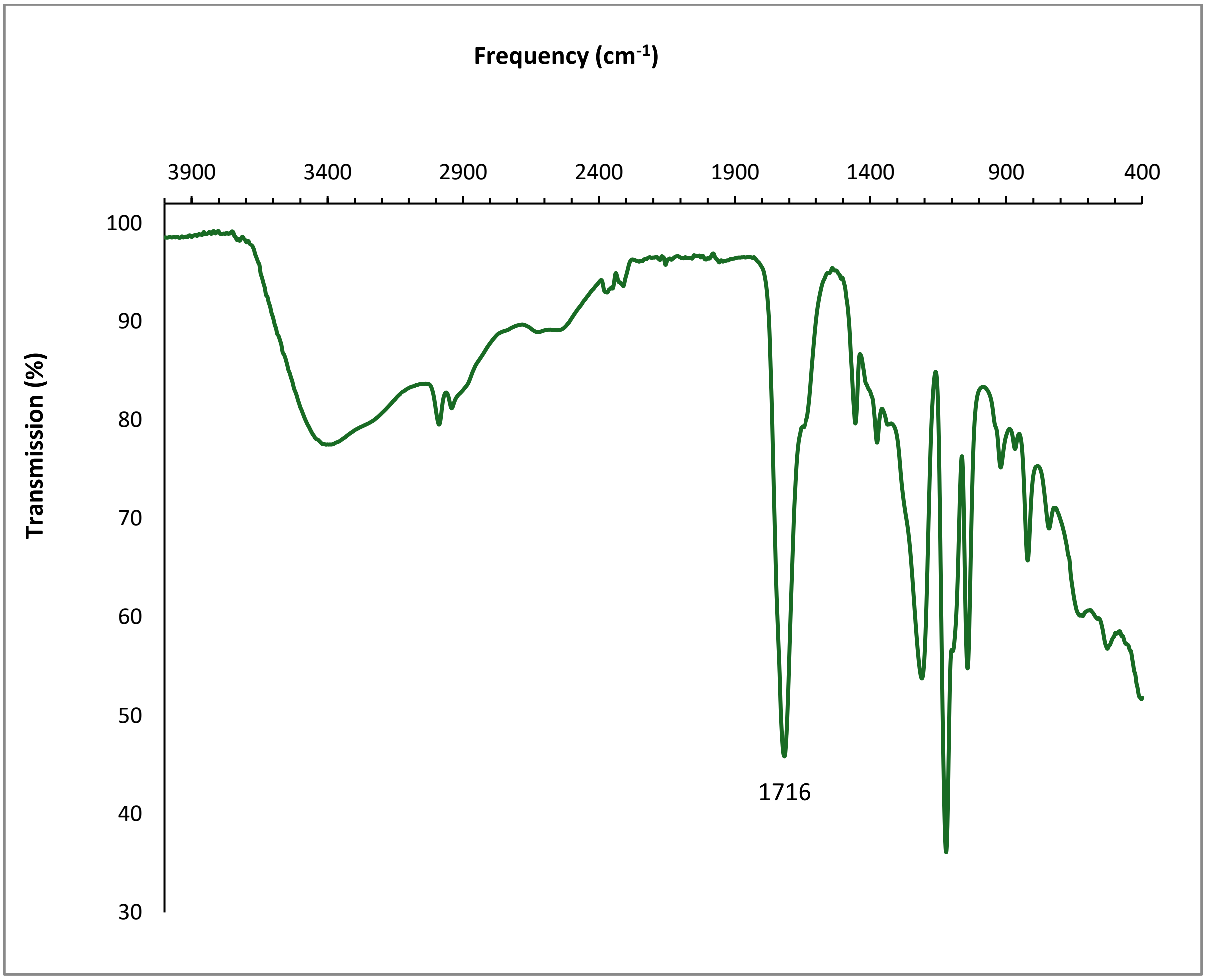

Figure S160. FTIR spectrum of fresh LA monomers. Indicative C=O stretching of the carboxylic acid is assigned at 1716cm^{-1}.

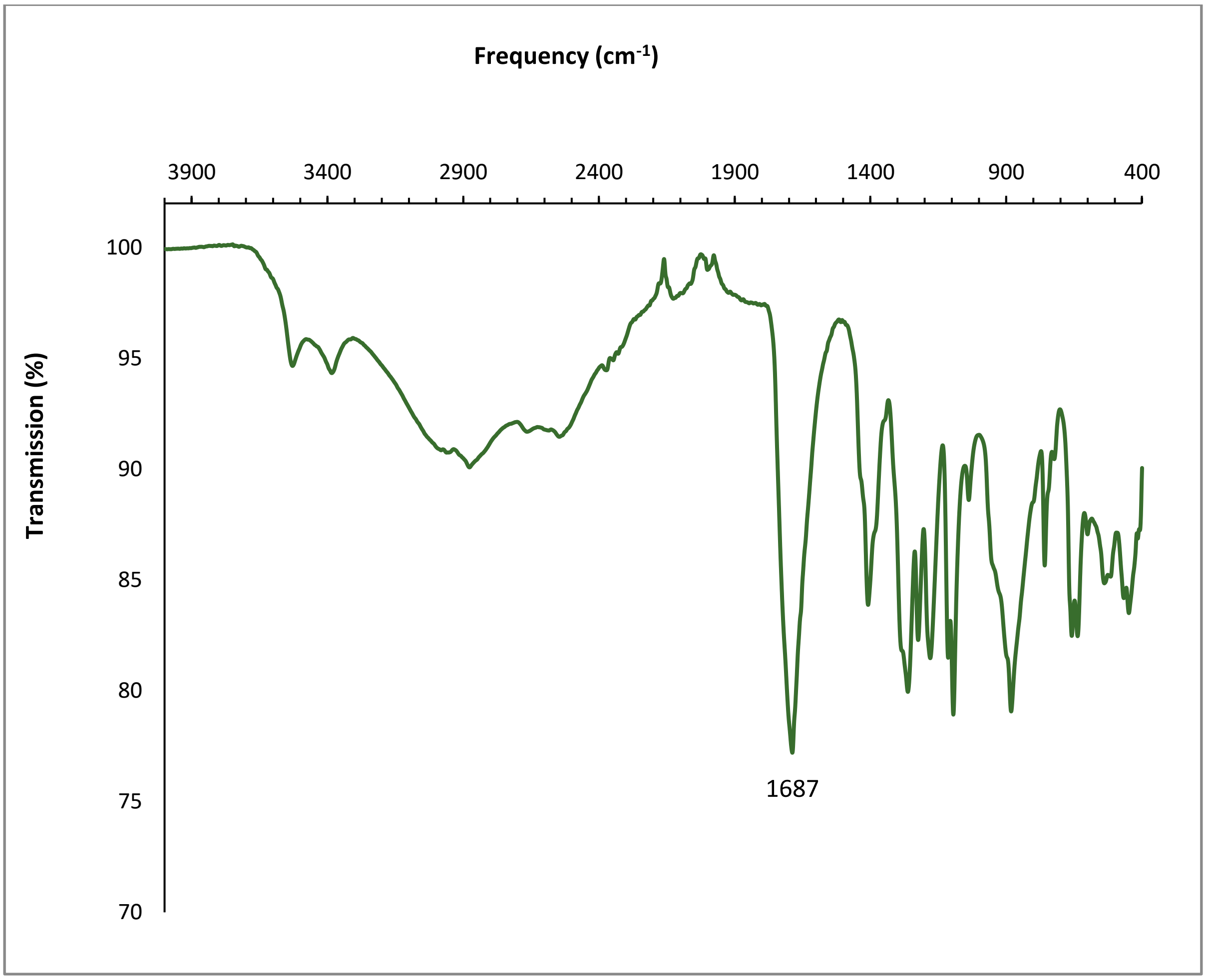

Figure S161. FTIR spectrum of fresh MA monomers. Indicative C=O stretching of the carboxylic acid is assigned at 16876cm^{-1}.

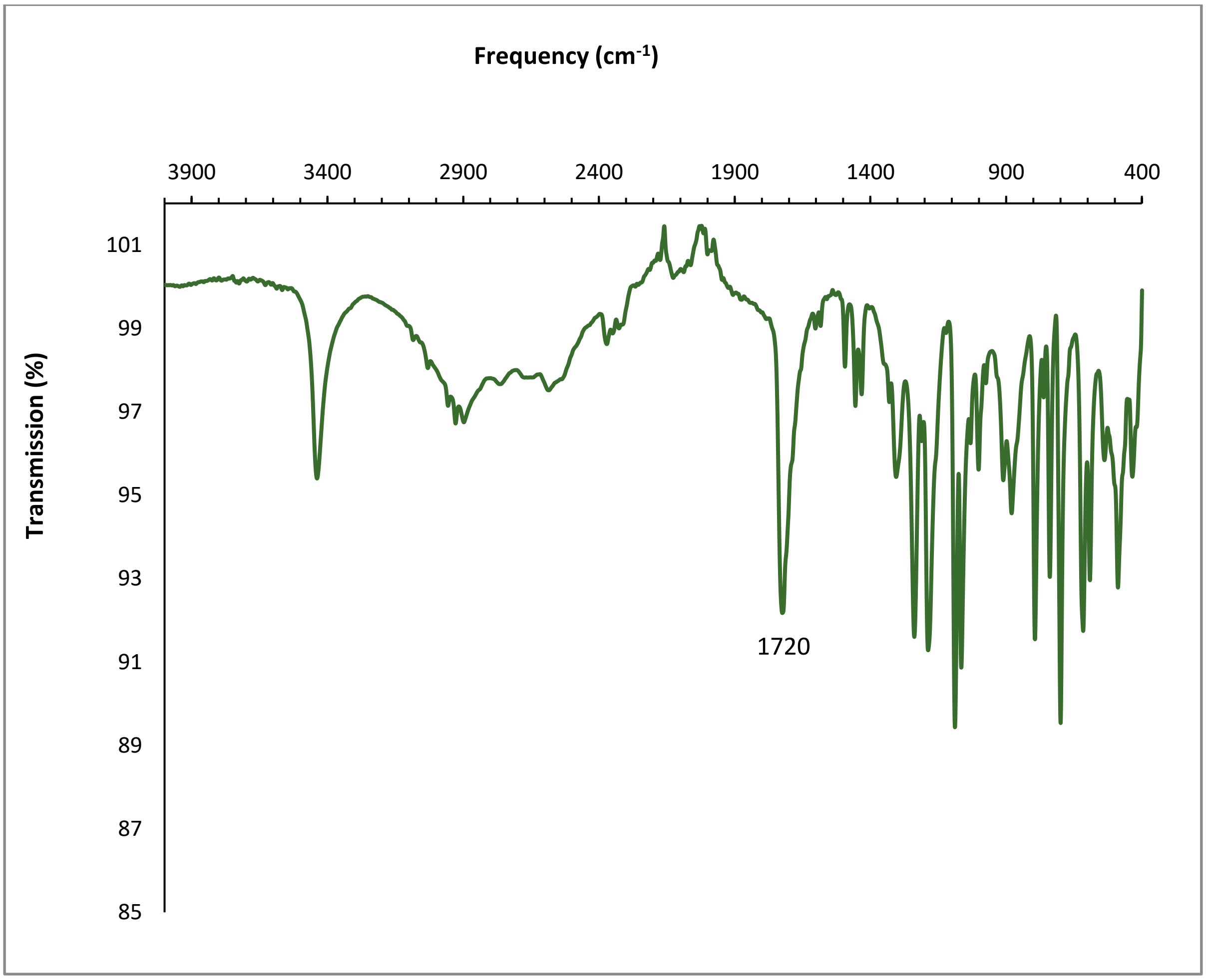

Figure S162. FTIR spectrum of fresh PLA monomers. Indicative C=O stretching of the carboxylic acid is assigned at 1720cm^{-1}.

A

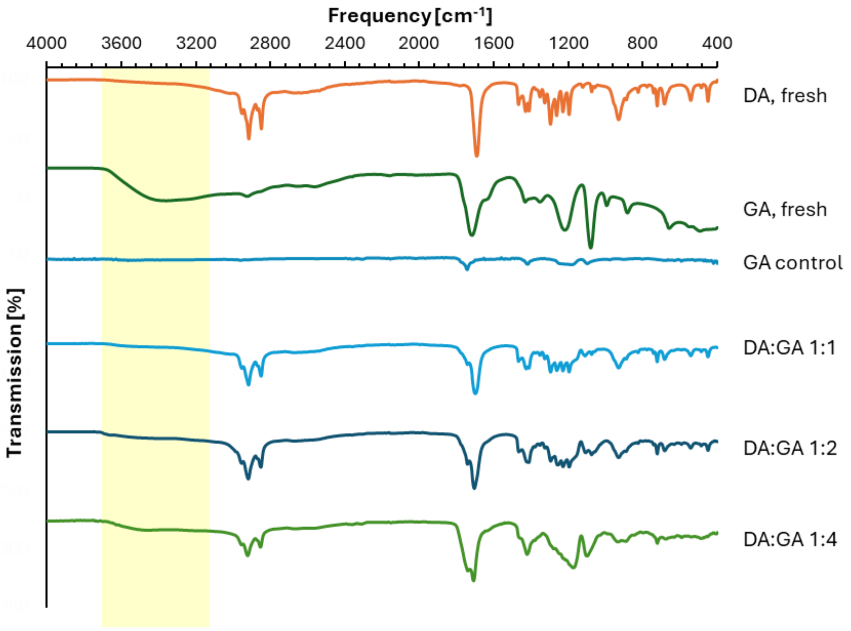

B

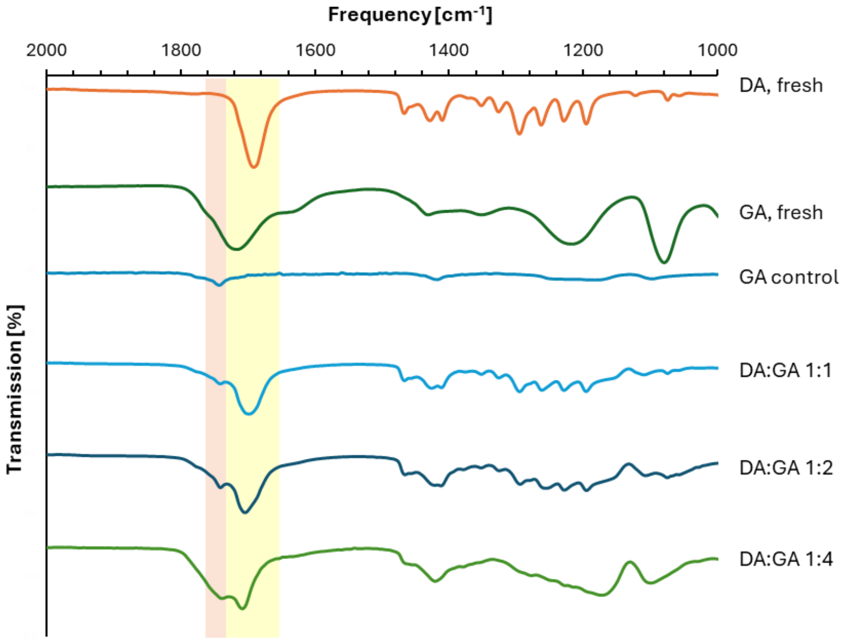

Figure S163. FTIR spectra of DA and GA fresh monomers and reaction products. Full-scale spectra within frequency range of 400-4000cm^{-1} (A) and zoom-in spectra of the frequency range indicative to the carboxylic acid to ester bonds shift (B). As indicated by the spectra, upon GA reaction, the O-H stretching signal of GA at about 3400cm^{-1} is lost. As GA and DA oligomerization proceeded, the intensity of the C=O stretching of ester bond at 1740cm^{-1} increased as highlighted in the red region in the spectra. The yellow region indicates the C=O stretching of the carboxylic group.

A

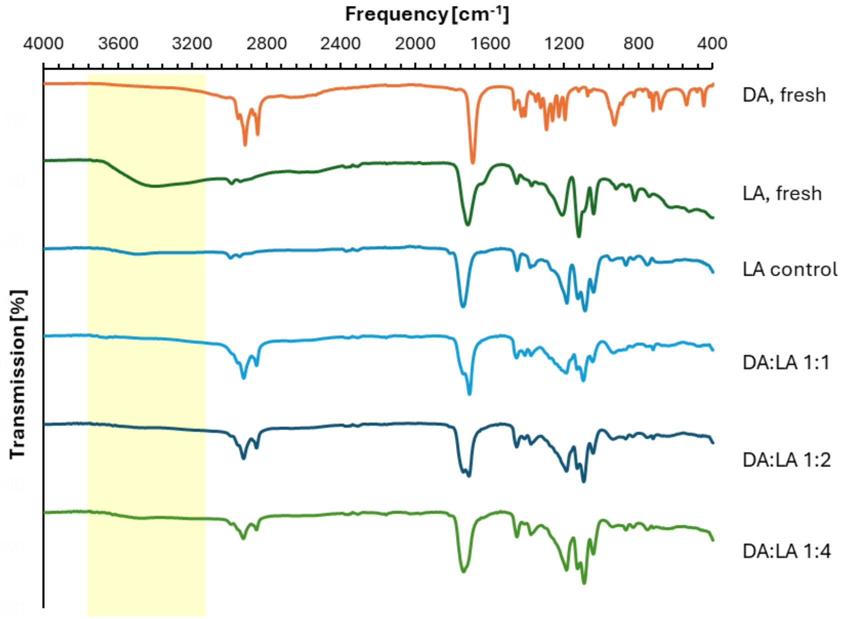

B

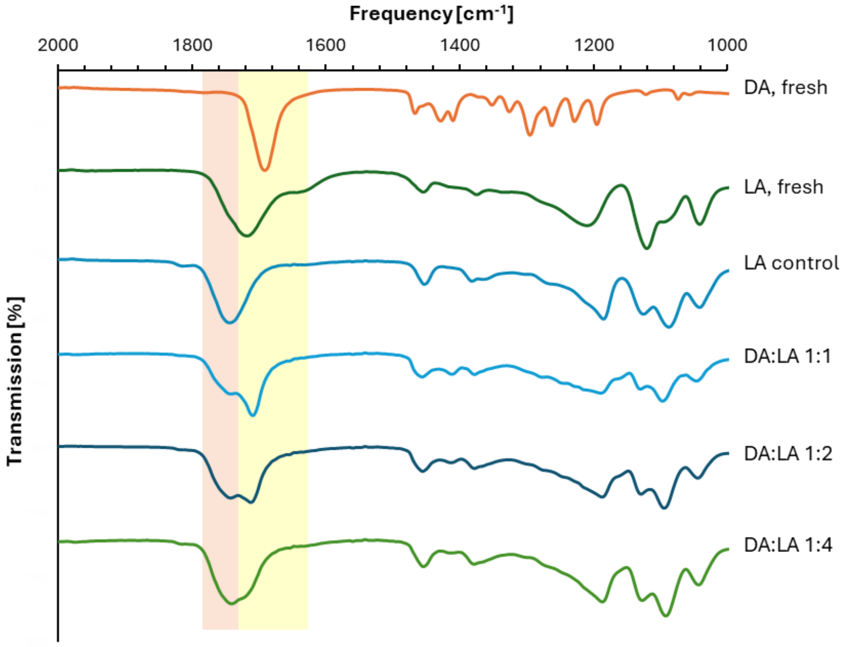

Figure S164. FTIR spectra of DA and LA fresh monomers and reaction products. Full-scale spectra within frequency range of 400-4000cm^{-1} (A) and zoom-in spectra of the frequency range indicative to the carboxylic acid to ester bonds shift (B). As indicated by the spectra, upon LA reaction, the O-H stretching signal of LA at about 3400cm^{-1} is lost. As LA and DA oligomerization proceeded, the intensity of C=O stretching of ester bond at about 1740-1745cm^{-1} increased as highlighted in the red region in the spectra. The Yellow region indicates the C=O stretching of the carboxylic group.

A

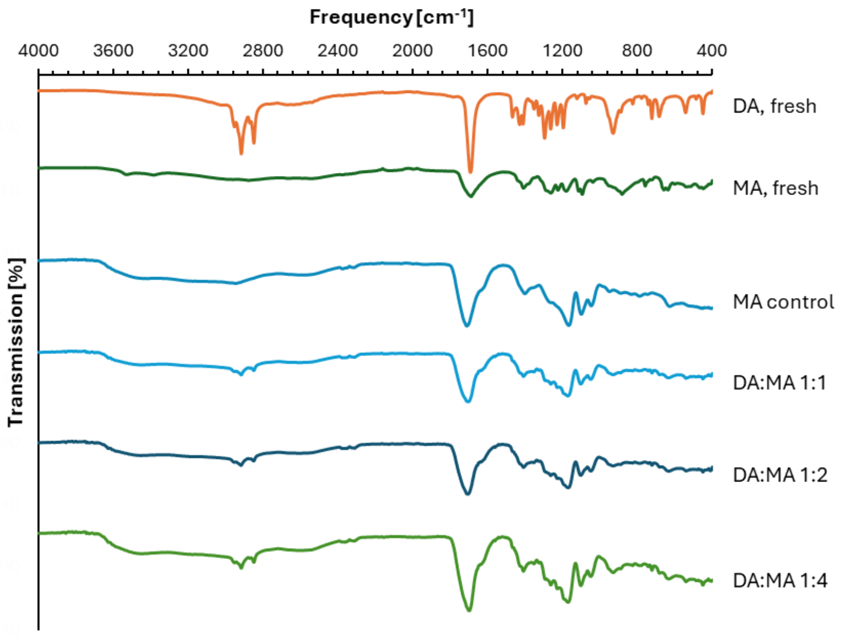

B

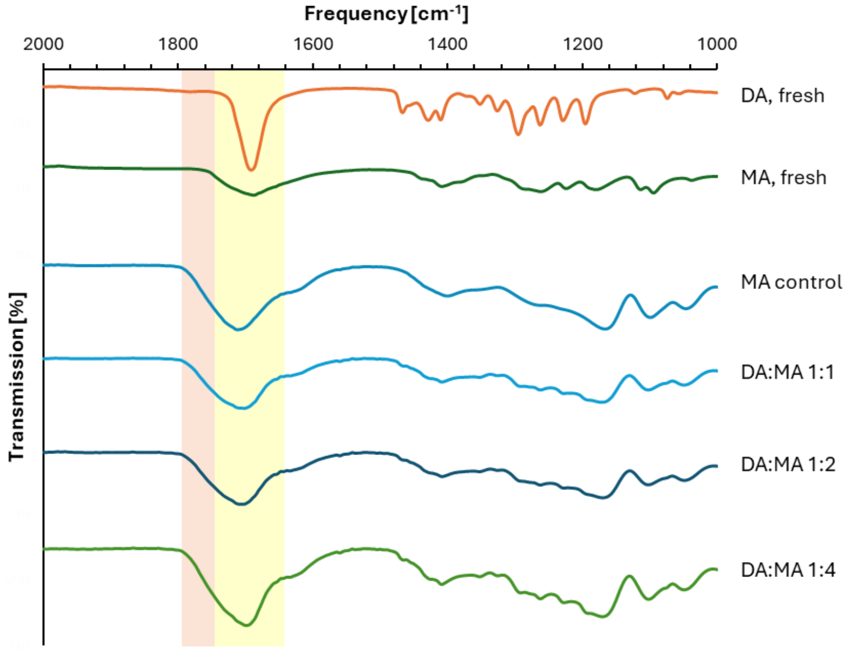

Figure S165. FTIR spectra of DA and MA fresh monomers and reaction products. Full-scale spectra within frequency range of 400-4000cm^{-1} (A) and zoom-in spectra of the frequency range indicative to the carboxylic acid to ester bonds shift (B). As indicated by the red region in the spectra, upon MA and DA oligomerization, the signal corresponding to C=O stretching becomes broaden with signal offsets shifted from ca 1730 cm^{-1} to about 1765-1770cm^{-1}.

A

B

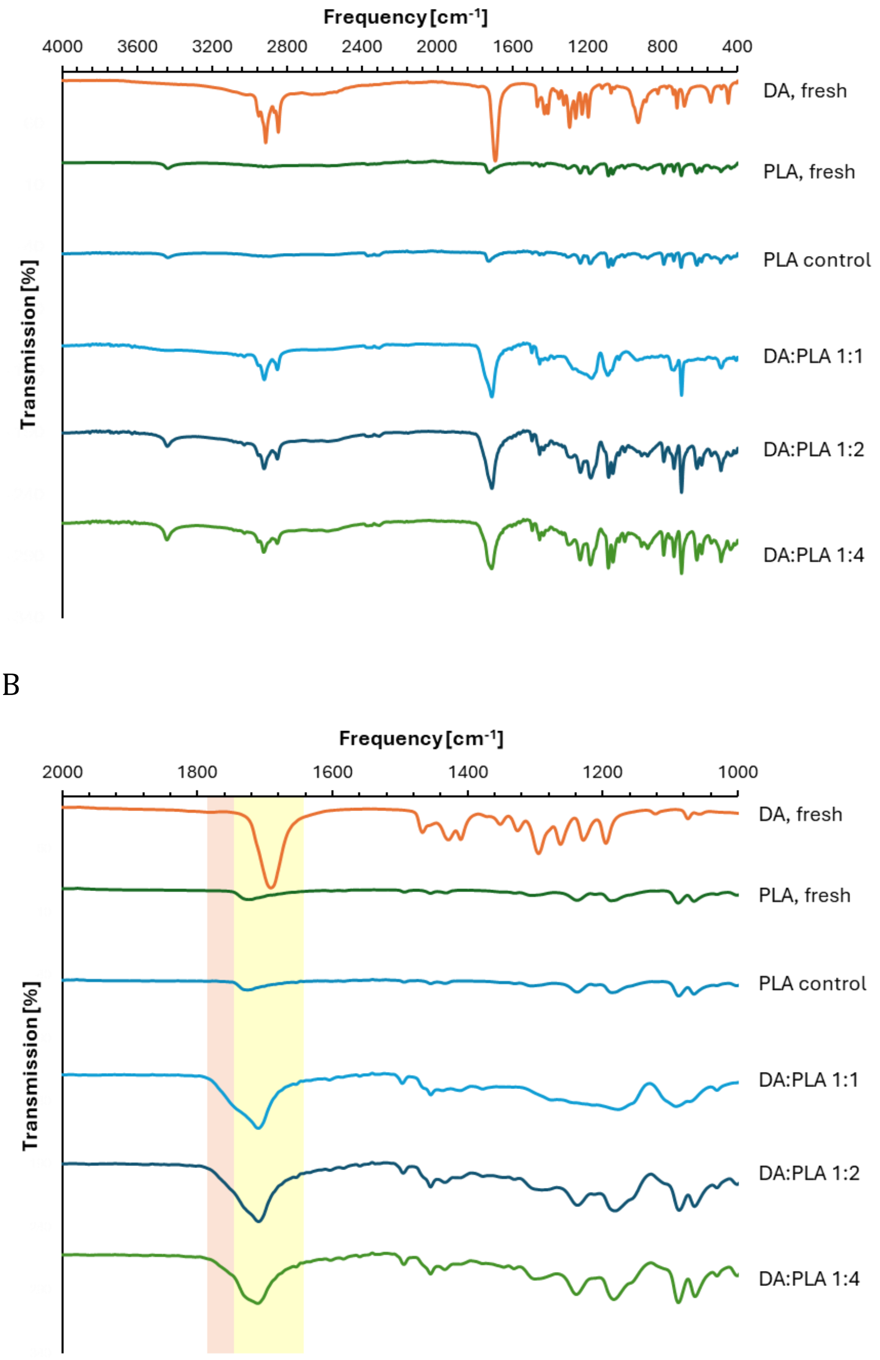

Figure S166. FTIR spectra of DA and PLA fresh monomers and reaction products. Full-scale spectra within frequency range of 400-4000cm^{-1} (A) and zoom-in spectra of the frequency range indicative to the carboxylic acid to ester bonds shift (B). As indicated by the red region in the spectra, upon PLA and DA oligomerization, a shoulder-like signal at around 1750-1760cm^{-1} is detected corresponding to C=O ester stretching.

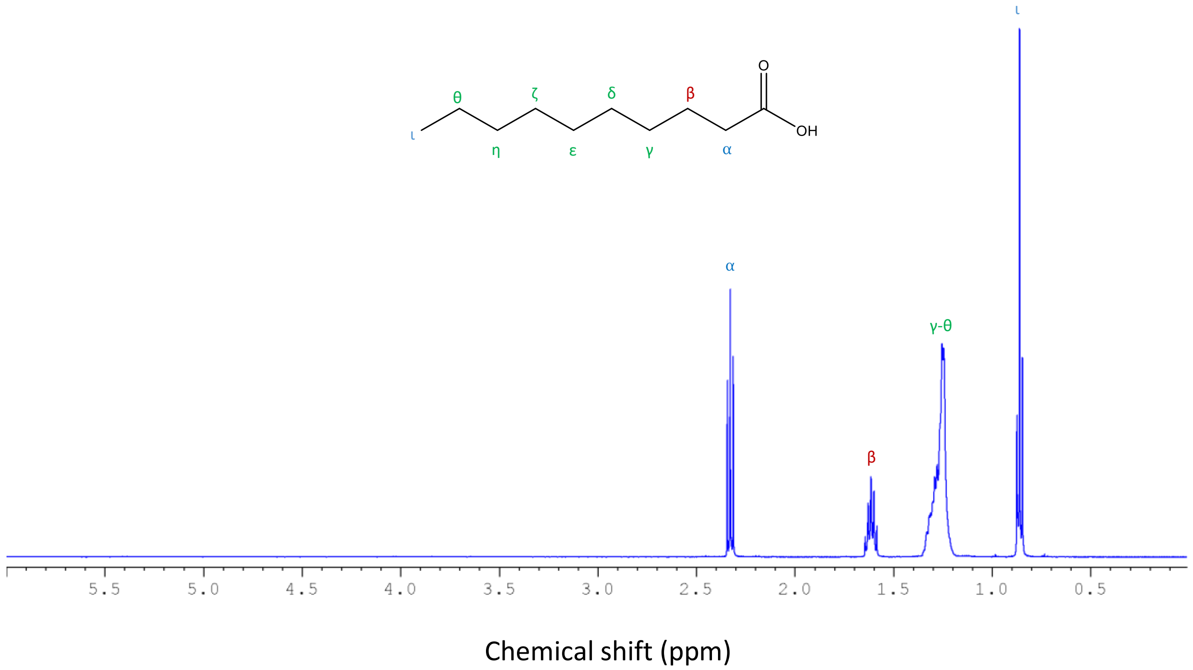

Figure S167. ^{1}H-NMR spectrum of DA fresh monomer in $CDCl_3$.

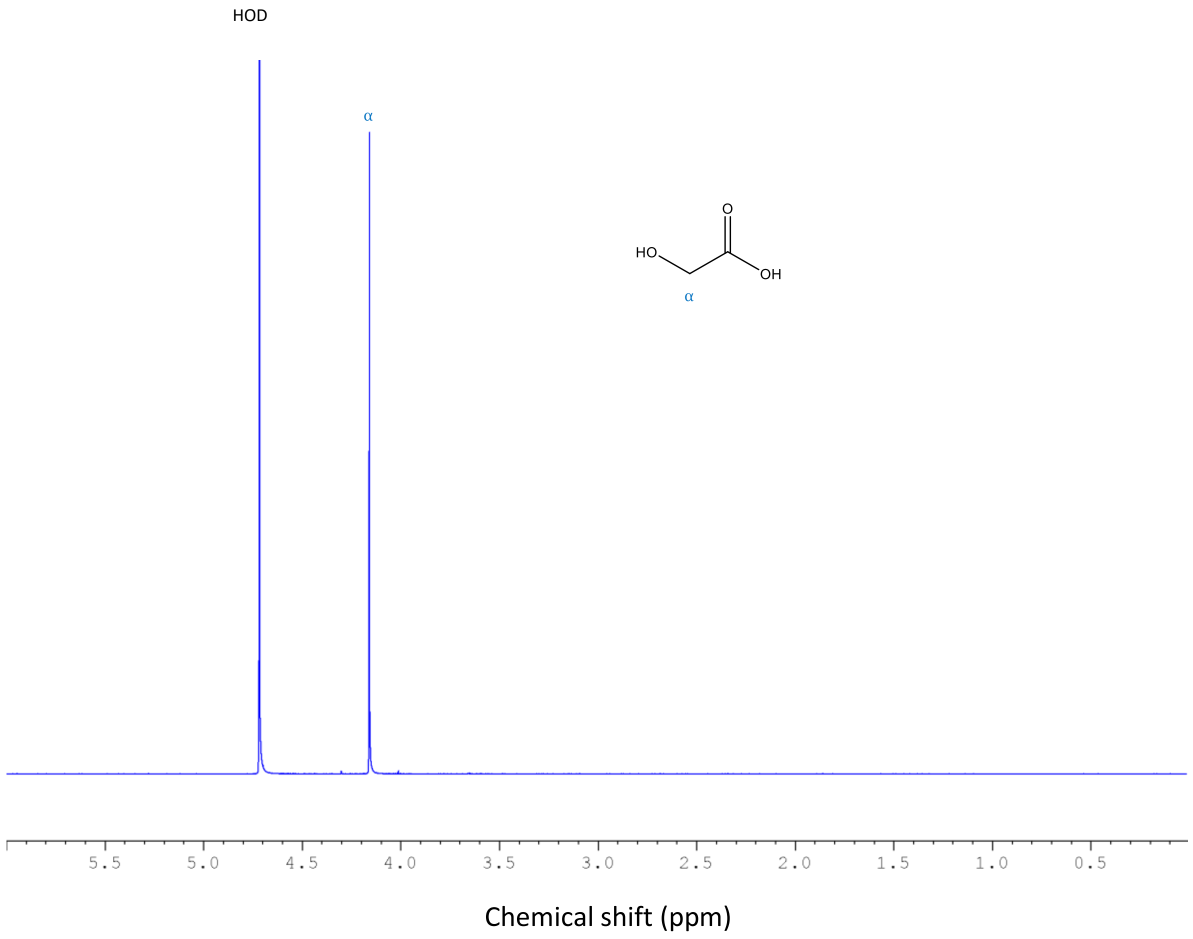

Figure S168. ^{1}H-NMR spectrum of GA fresh monomer in D_2O.

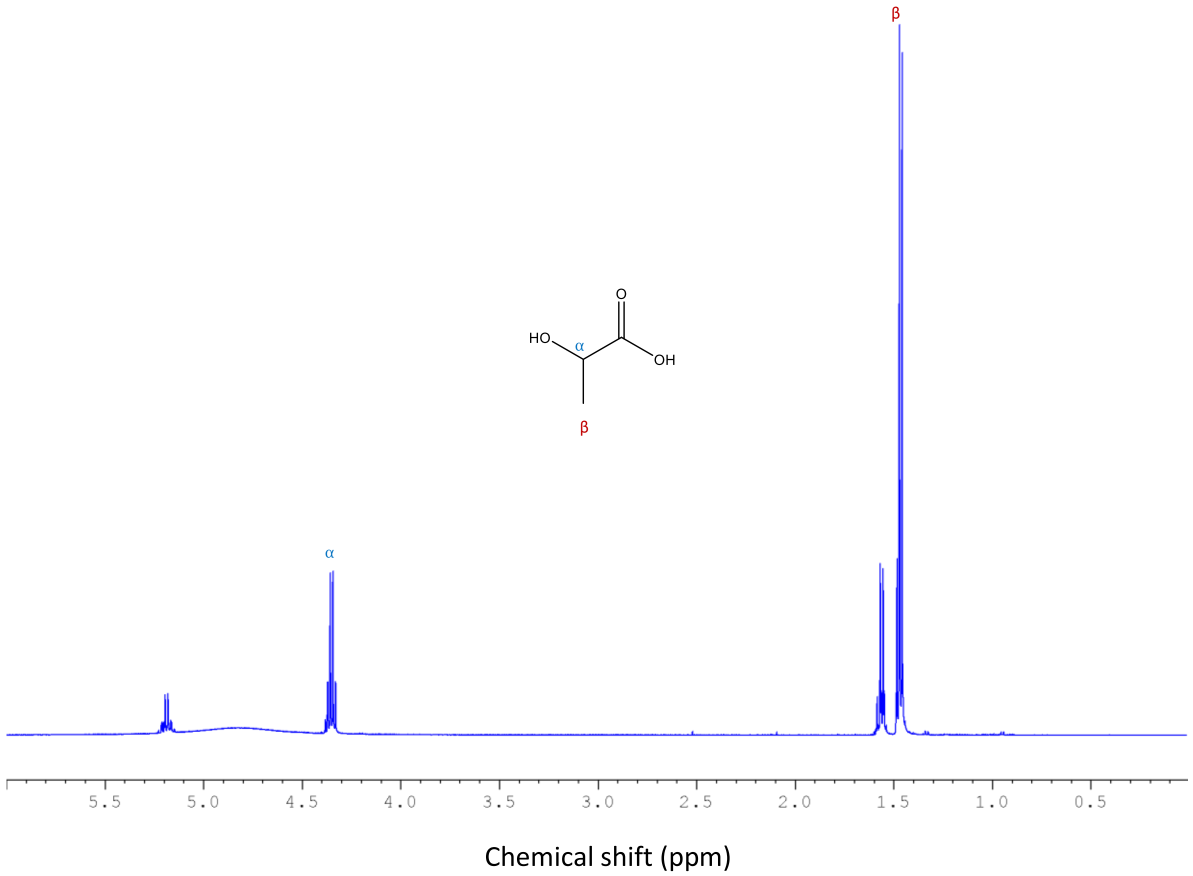

Figure S169. ^{1}H-NMR spectrum of LA fresh monomer in $CDCl_3$. The stock solution contains 2LA.

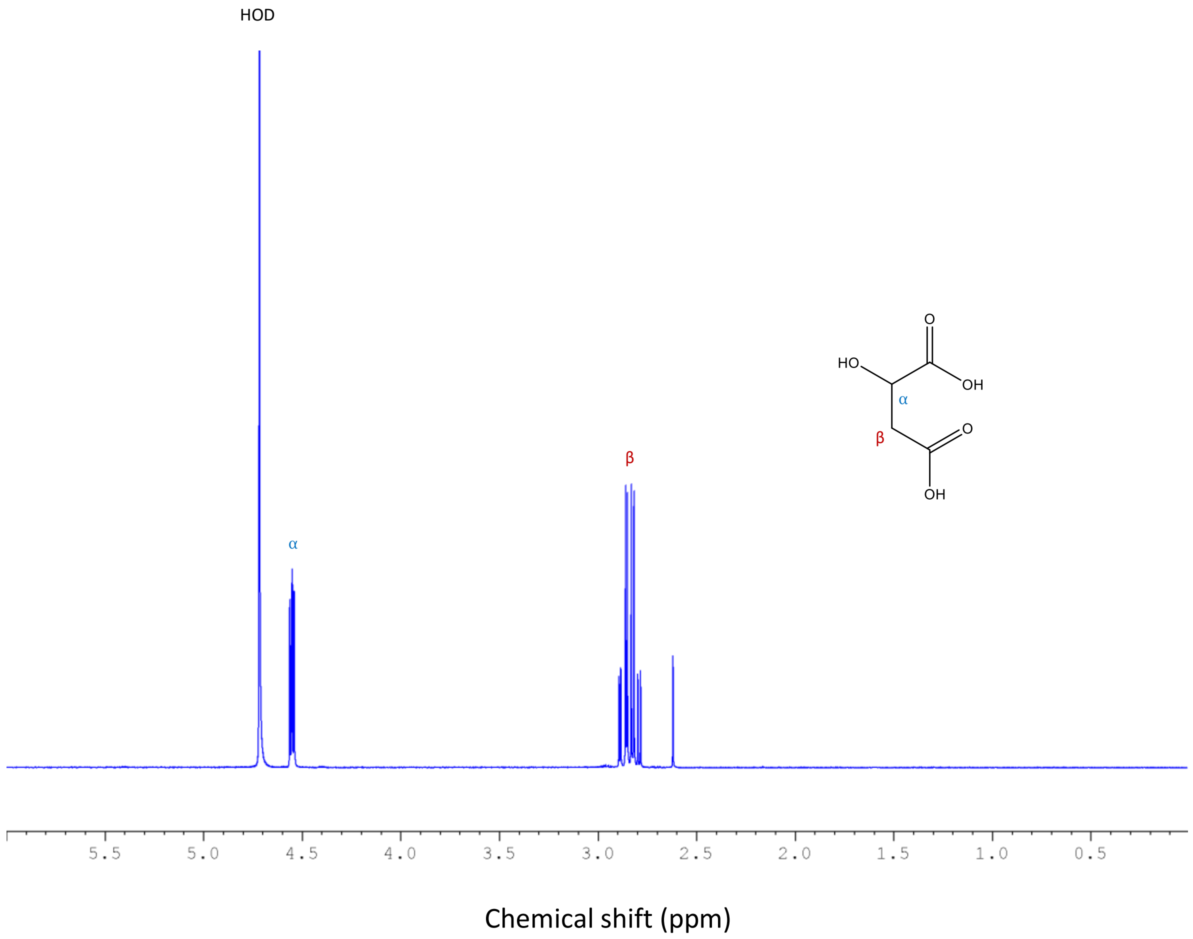

Figure S170. ¹H-NMR spectrum of MA fresh monomer in D_2O.

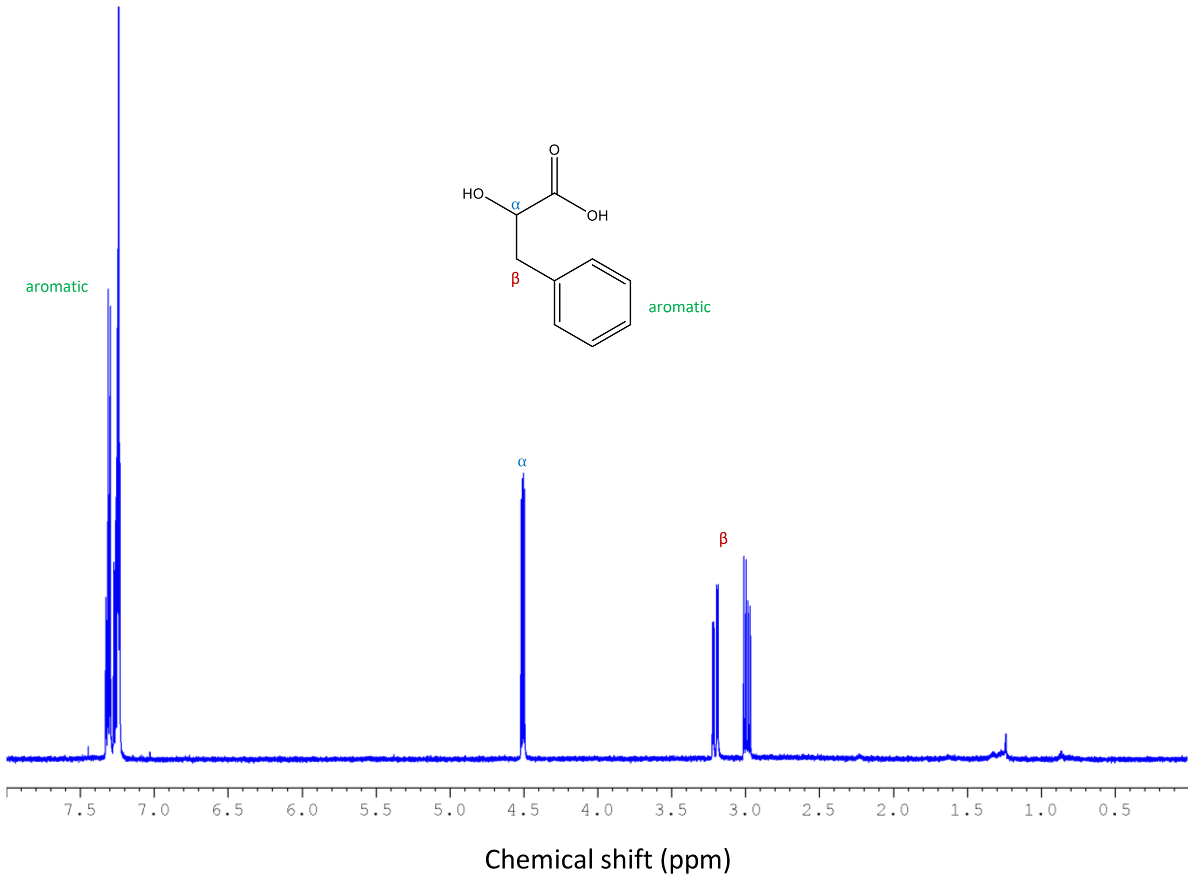

Figure S171. ^{1}H-NMR spectrum of PLA fresh monomer in $CDCl_3$.

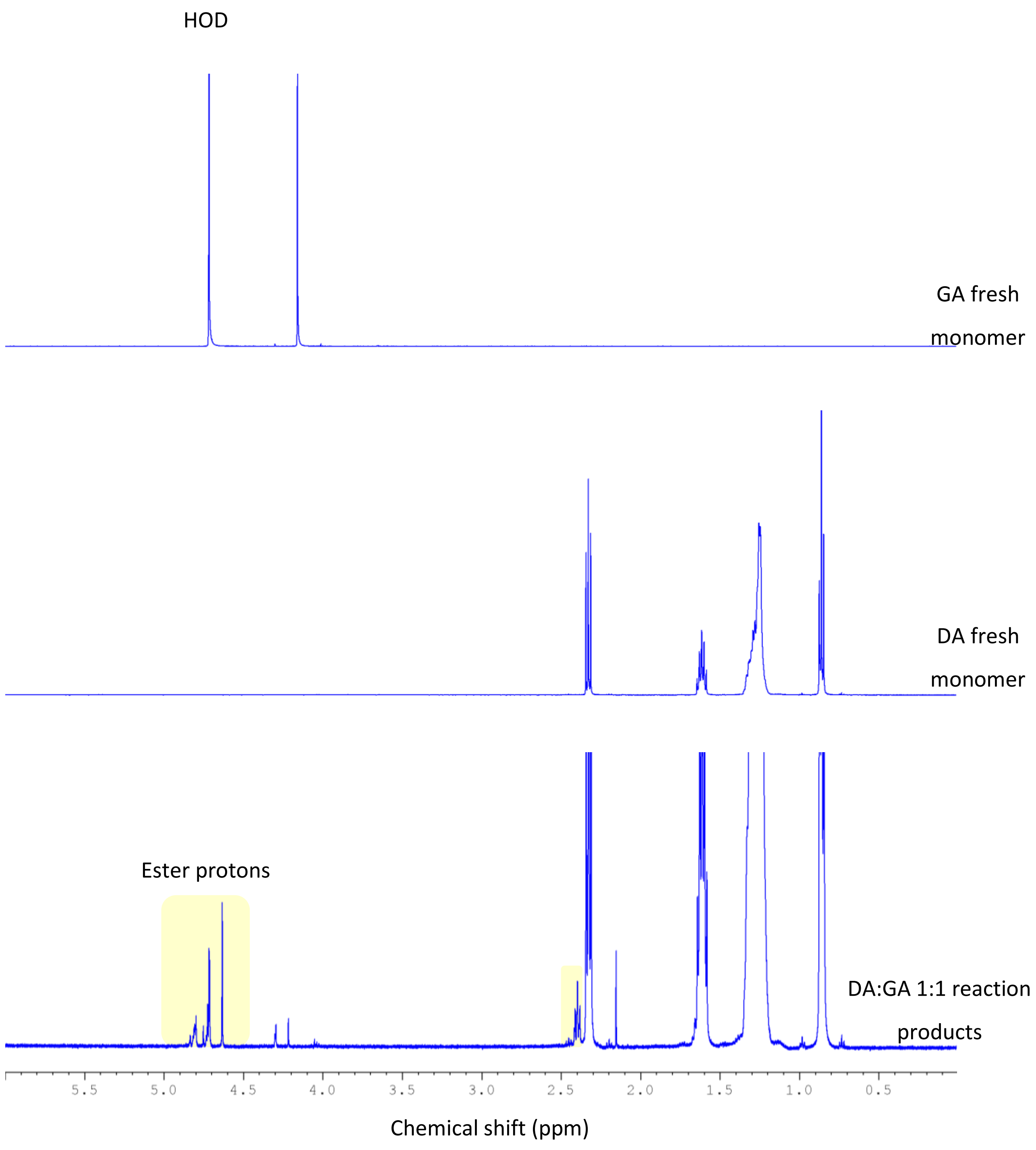

Figure S172. ^{1}H-NMR spectrum of DA:GA reaction product at 1:1 molar ratio. The dry reaction product was suspended in $CDCl_3$ and analyzed by ^{1}H-NMR. DA monomers in $CDCl_3$ and GA monomer in D_2O are presented for reference. Alpha protons shift downfield indicates the formation of ester bonds (highlighted in yellow).

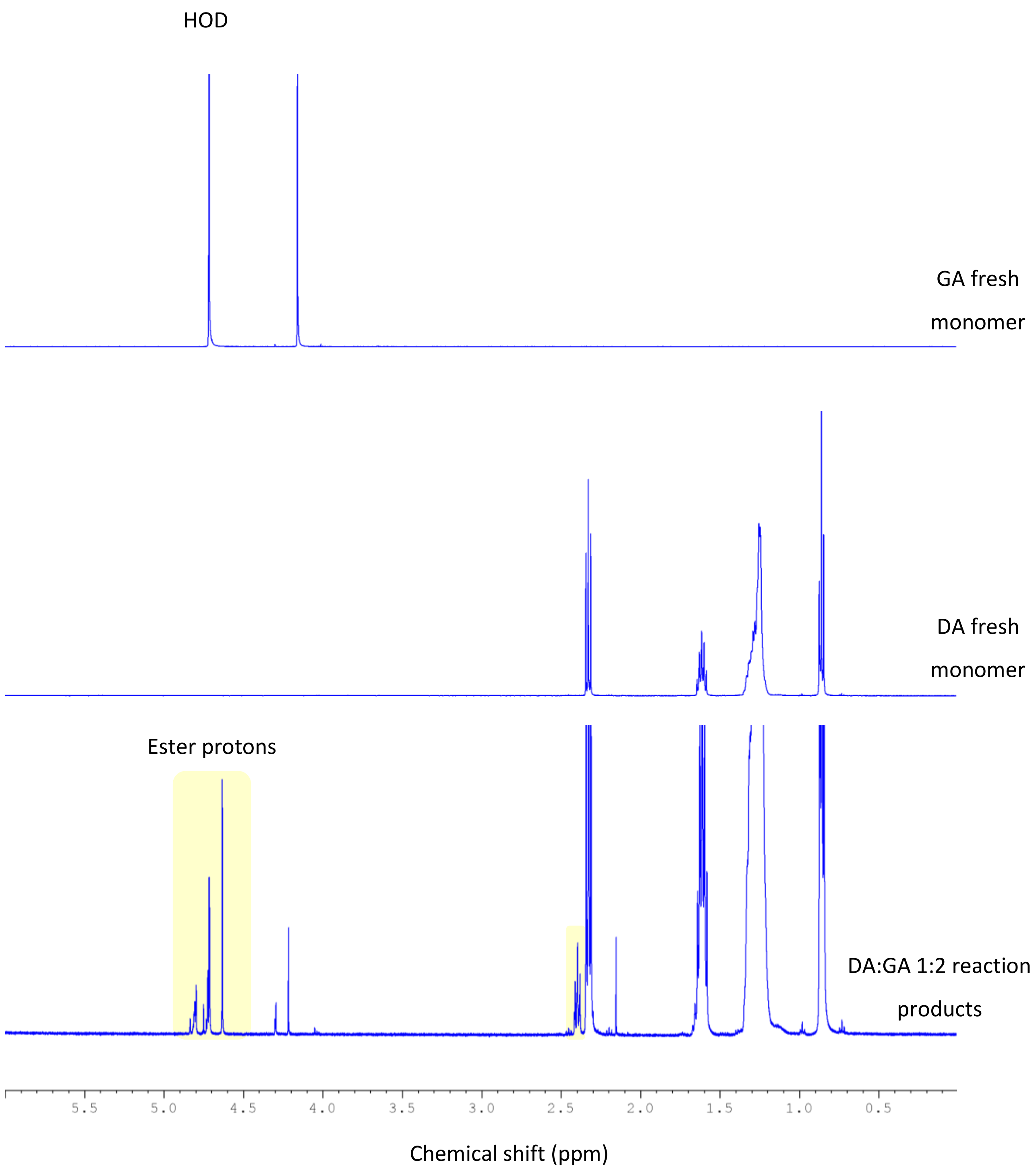

Figure S173. ^{1}H-NMR spectrum of DA:GA reaction product at 1:2 molar ratio. The dry reaction product was suspended in $CDCl_3$ and analyzed by ^{1}H-NMR. DA monomers in $CDCl_3$ and GA monomer in D_2O are presented for reference. Alpha protons shift downfield indicates the formation of ester bonds (highlighted in yellow).

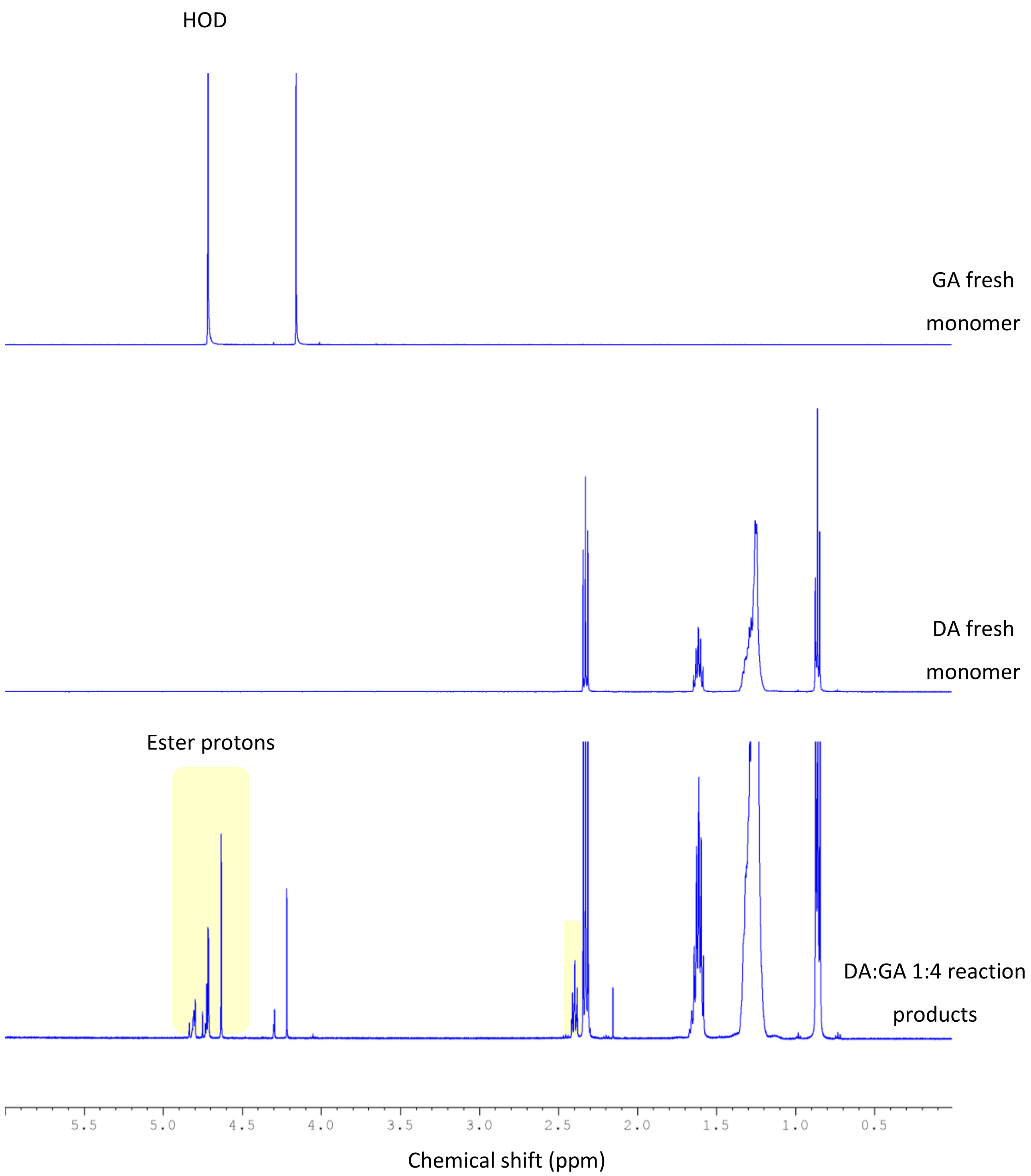

Figure S174. ^{1}H-NMR spectrum of DA:GA reaction product at 1:4 molar ratio. The dry reaction product was suspended in $CDCl_3$ and analyzed by ^{1}H-NMR. DA monomers in $CDCl_3$ and GA monomer in D_2O are presented for reference. Alpha protons shift downfield indicates the formation of ester bonds (highlighted in yellow).

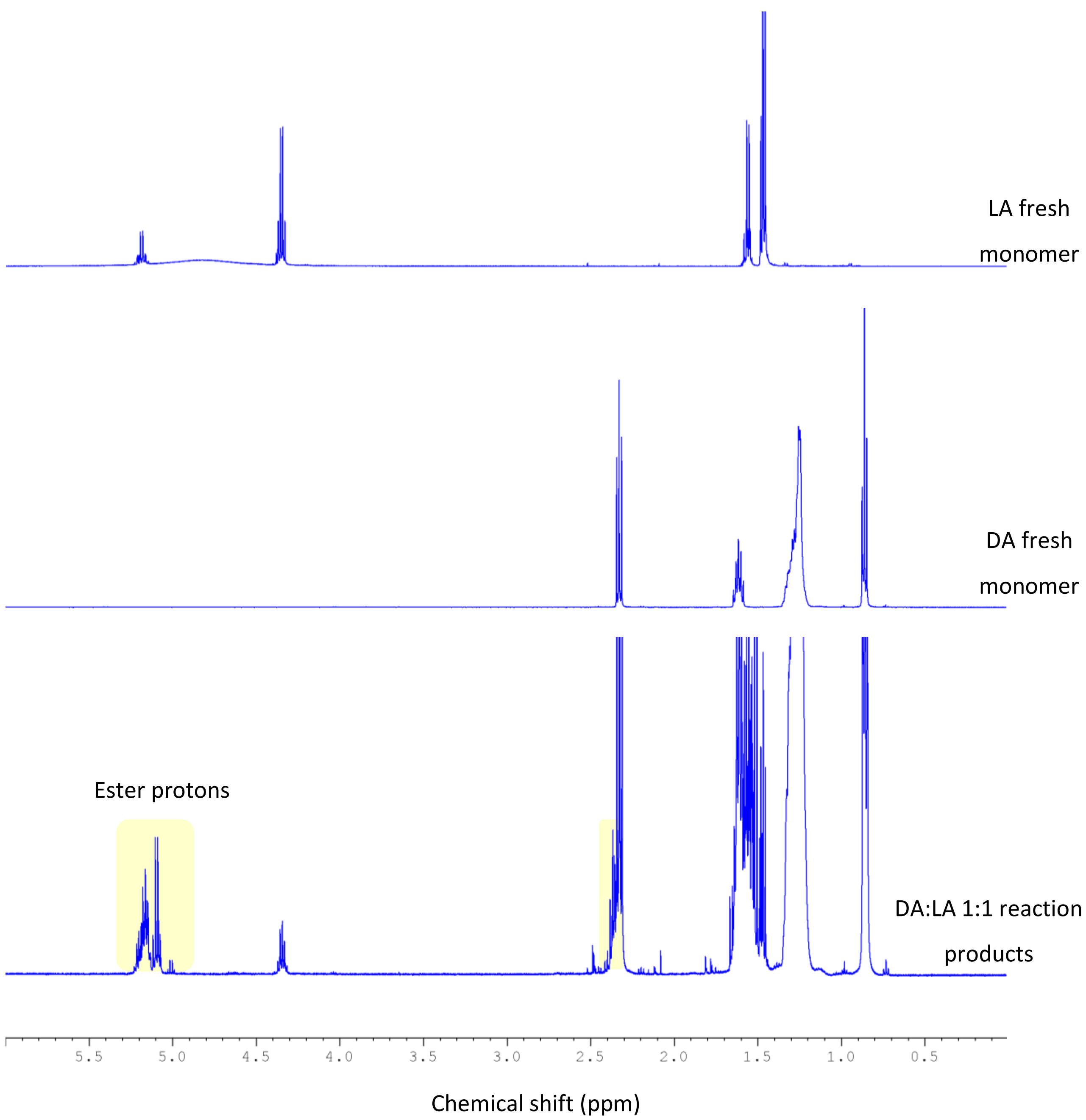

Figure S175. ^{1}H-NMR spectrum of DA:LA reaction product at 1:1 molar ratio. The dry reaction product was suspended in $CDCl_3$ and analyzed by ^{1}H-NMR. DA and LA monomers in $CDCl_3$ are presented for reference. Alpha protons shift downfield indicates the formation of ester bonds (highlighted in yellow).

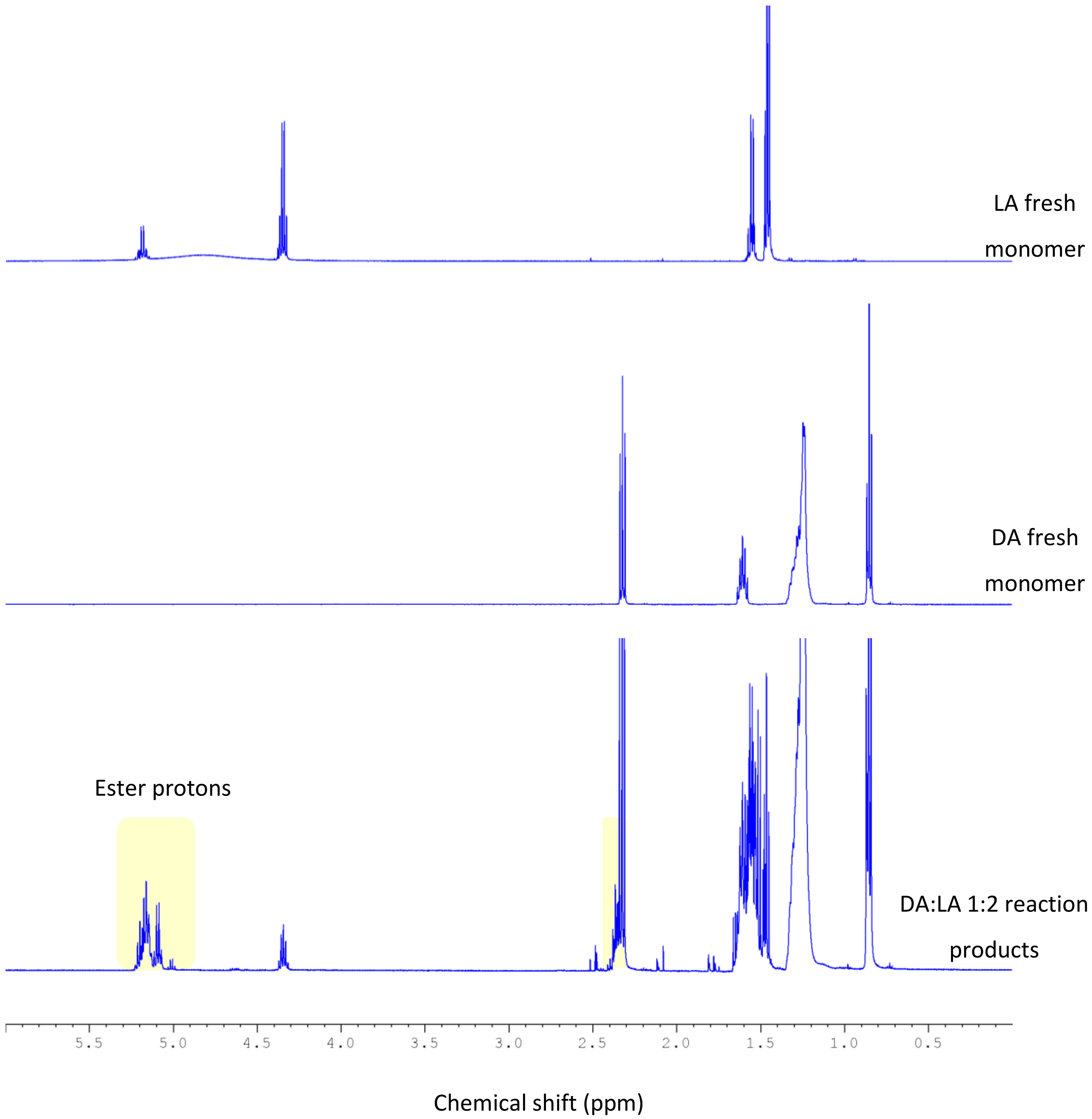

Figure S176. ¹H-NMR spectrum of DA:LA reaction product at 1:2 molar ratio. The dry reaction product was suspended in $CDCl_3$ and analyzed by ¹H-NMR. DA and LA monomers in $CDCl_3$ are presented for reference. Alpha protons shifts downfield indicates the formation of ester bonds (highlighted in yellow).

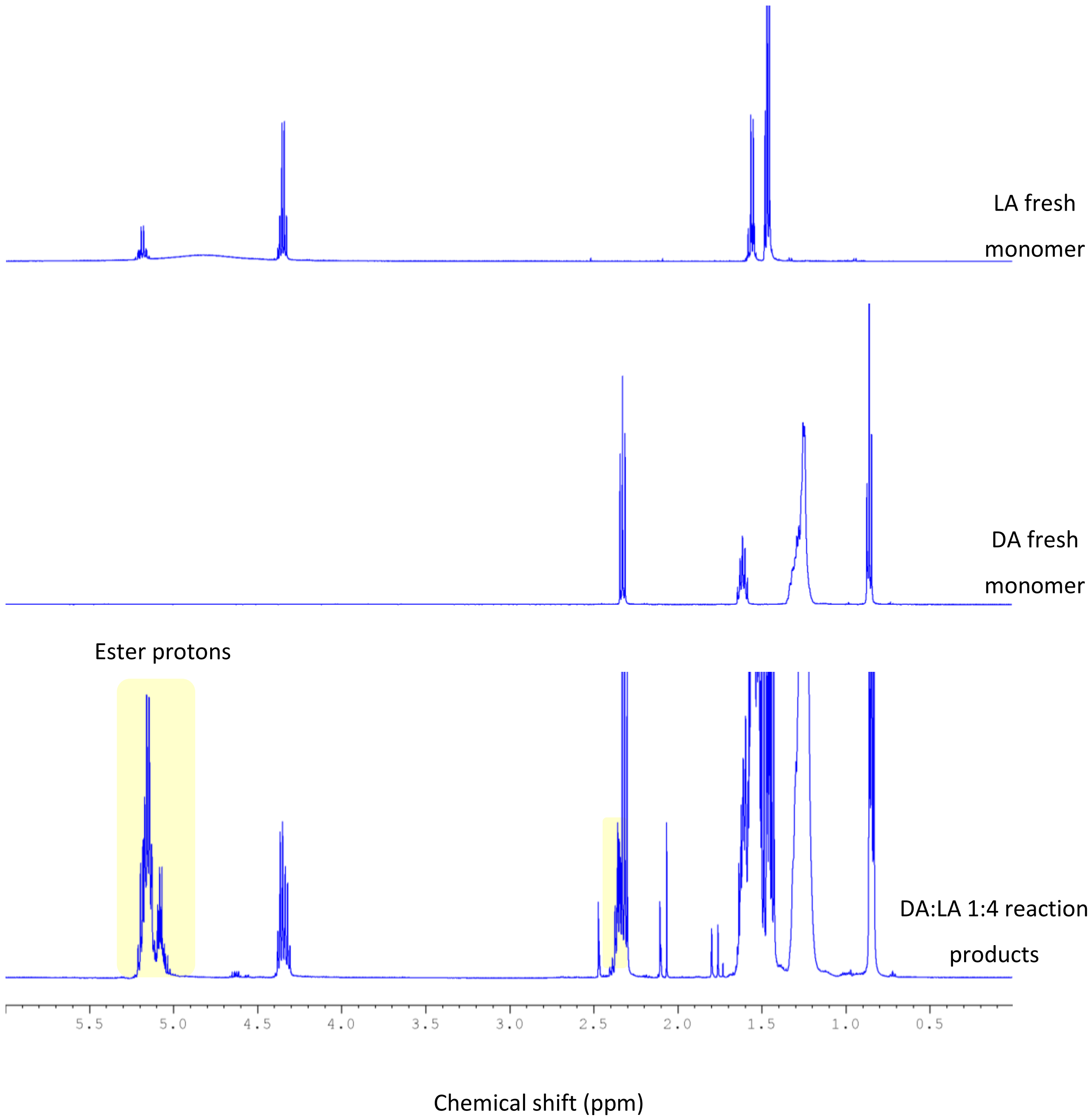

Figure S177. ^{1}H-NMR spectrum of DA:LA reaction product at 1:4 molar ratio. The dry reaction product was suspended in $CDCl_3$ and analyzed by ^{1}H-NMR. DA and LA monomers in $CDCl_3$ are presented for reference. Alpha protons shift downfield indicates the formation of ester bonds (highlighted in yellow).

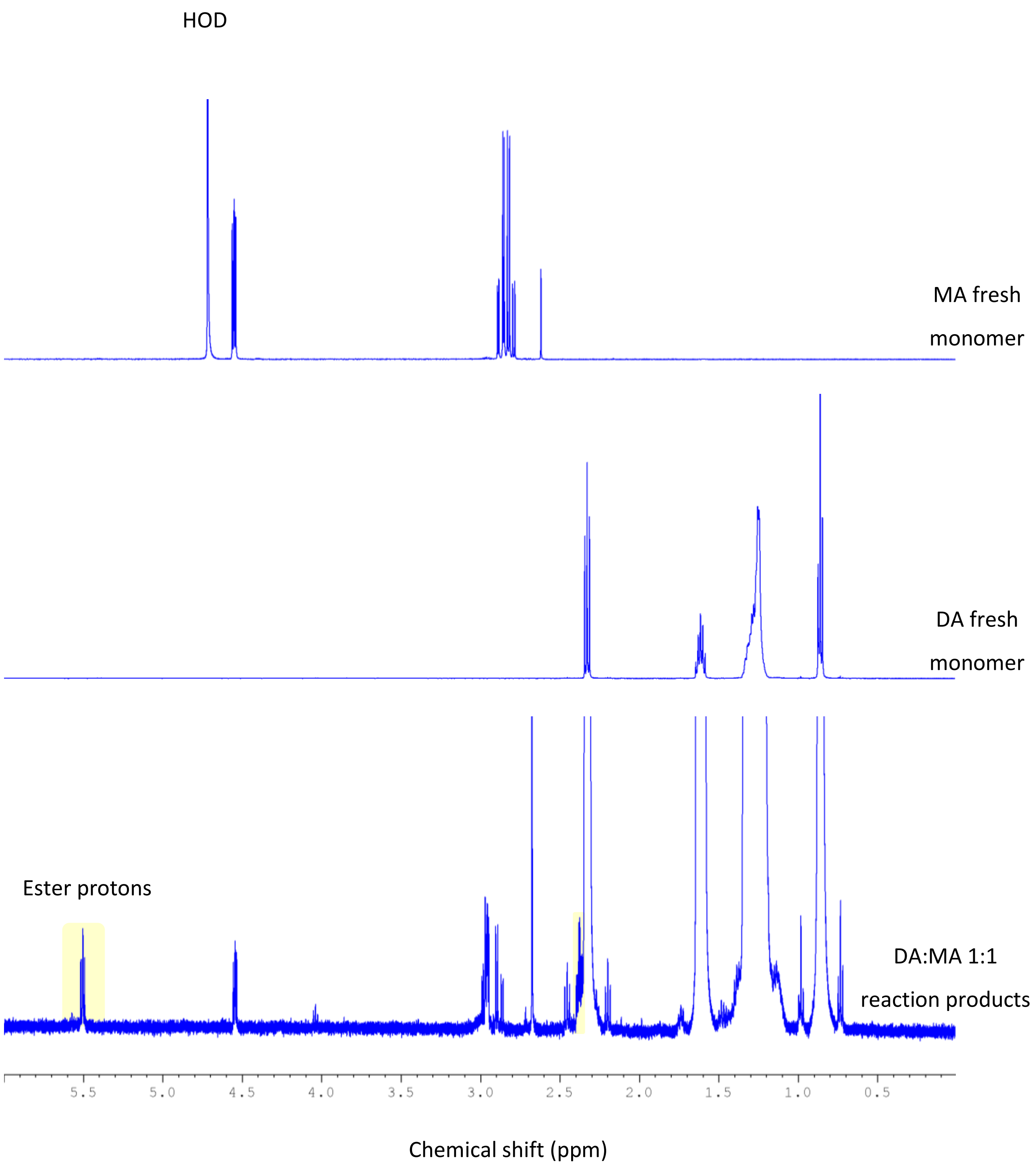

Figure S178. ^{1}H-NMR spectrum of DA:MA reaction product at 1:1 molar ratio. The dry reaction product was suspended in $CDCl_3$ and analyzed by ^{1}H-NMR. DA monomers in $CDCl_3$ and MA monomer in D_2O are presented for reference. Alpha protons shift downfield indicates the formation of ester bonds (highlighted in yellow).

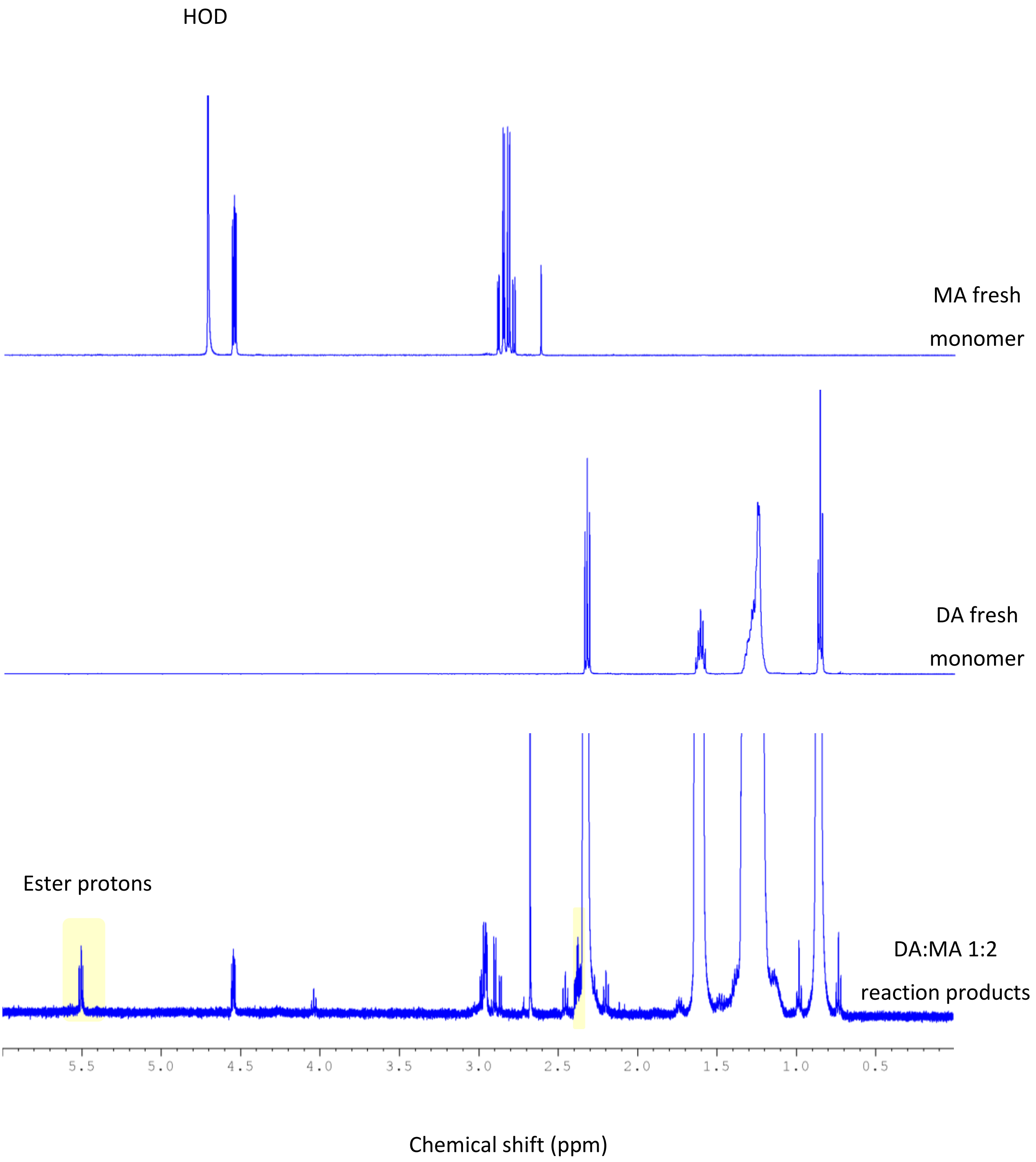

Figure S179. ^{1}H-NMR spectrum of DA:MA reaction product at 1:2 molar ratio. The dry reaction product was suspended in $CDCl_3$ and analyzed by ^{1}H-NMR. DA monomers in $CDCl_3$ and MA monomer in D_2O are presented for reference. Alpha protons shift downfield indicates the formation of ester bonds (highlighted in yellow).

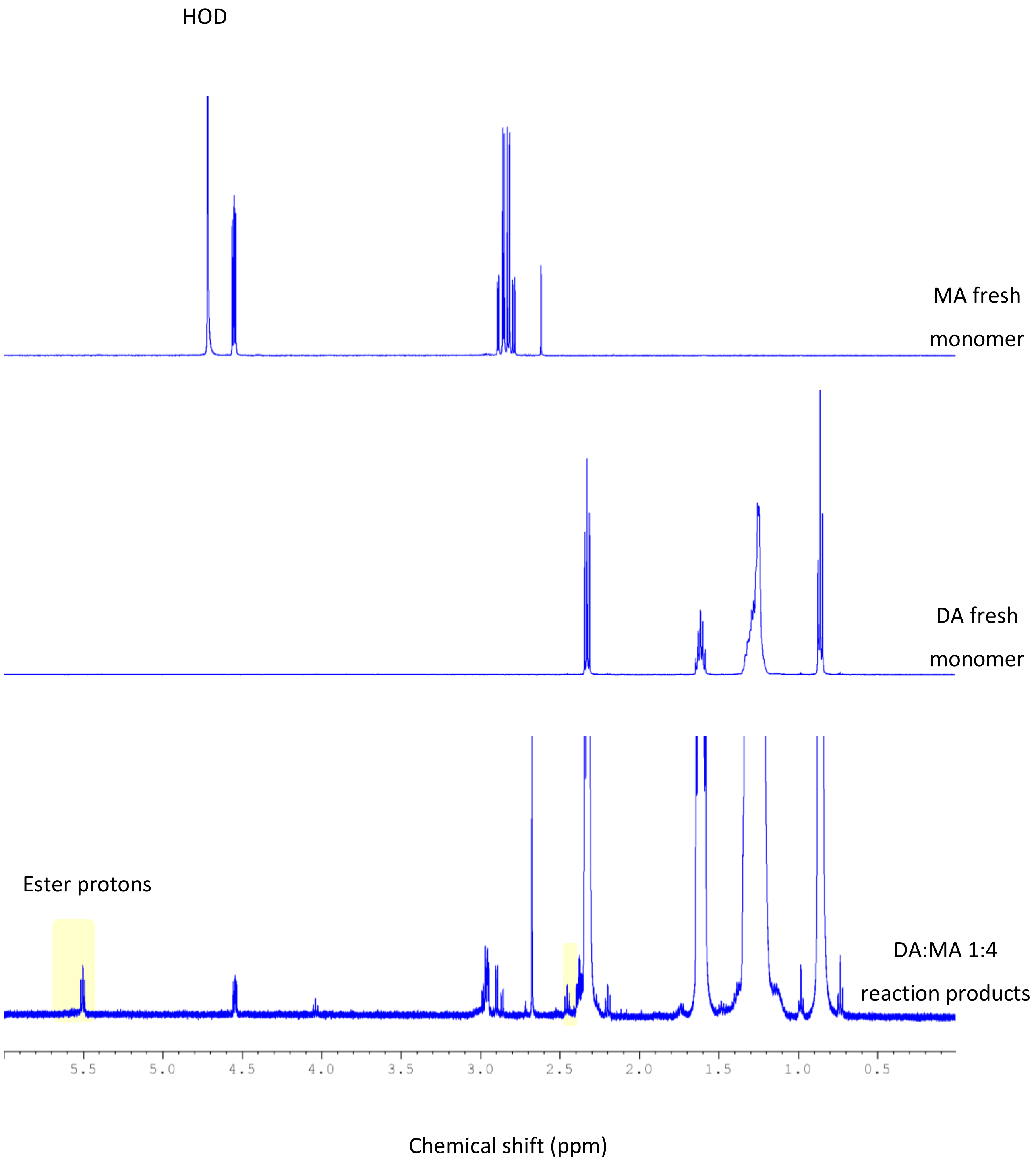

Figure S180. 1H-NMR spectrum of DA:MA reaction product at 1:4 molar ratio. The dry reaction product was suspended in $CDCl_3$ and analyzed by ^1H-NMR. DA monomers in $CDCl_3$ and MA monomer in D_2O are presented for reference. Alpha protons shift downfield indicates the formation of ester bonds (highlighted in yellow).

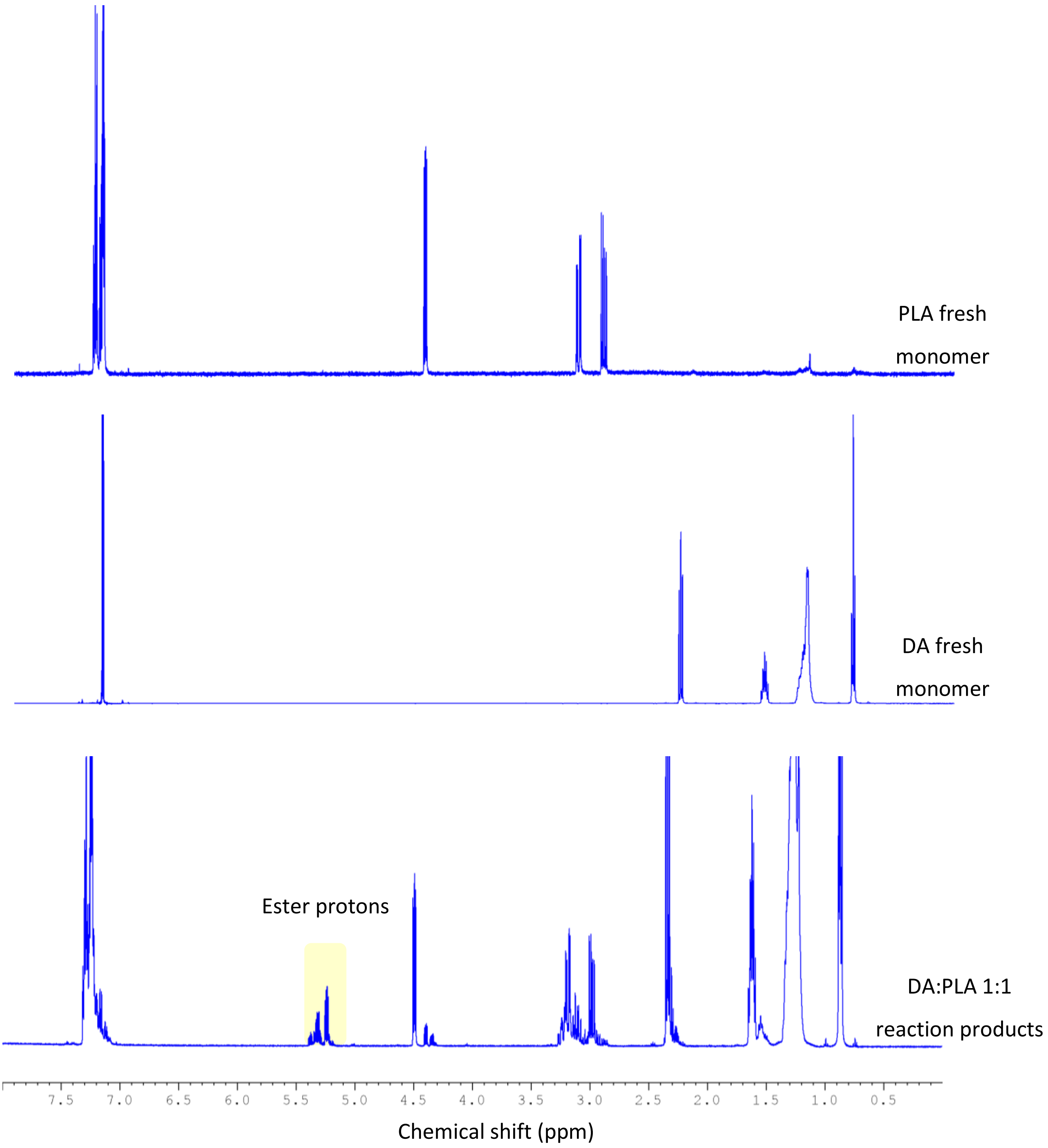

Figure S181. ^{1}H-NMR spectrum of DA:PLA reaction product at 1:1 molar ratio. The dry reaction product was suspended in $CDCl_3$ and analyzed by ^{1}H-NMR. DA and PLA monomers in $CDCl_3$ are presented for reference. Alpha protons shift downfield indicates the formation of ester bonds (highlighted in yellow).

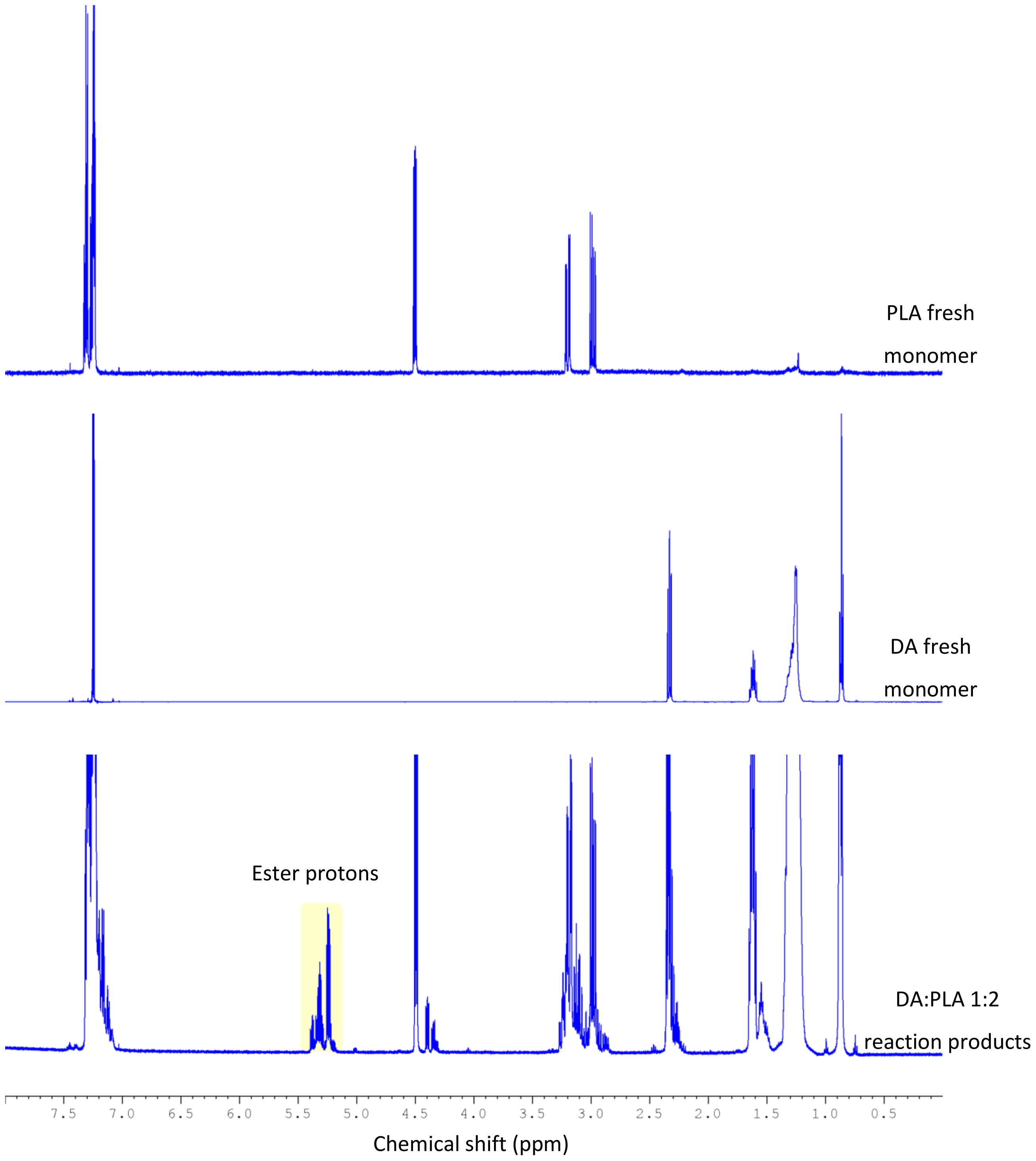

Figure S182. ^{1}H-NMR spectrum of DA:PLA reaction product at 1:2 molar ratio. The dry reaction product was suspended in $CDCl_3$ and analyzed by ^{1}H-NMR. DA and PLA monomers in $CDCl_3$ are presented for reference. Alpha protons shift downfield indicates the formation of ester bonds (highlighted in yellow).

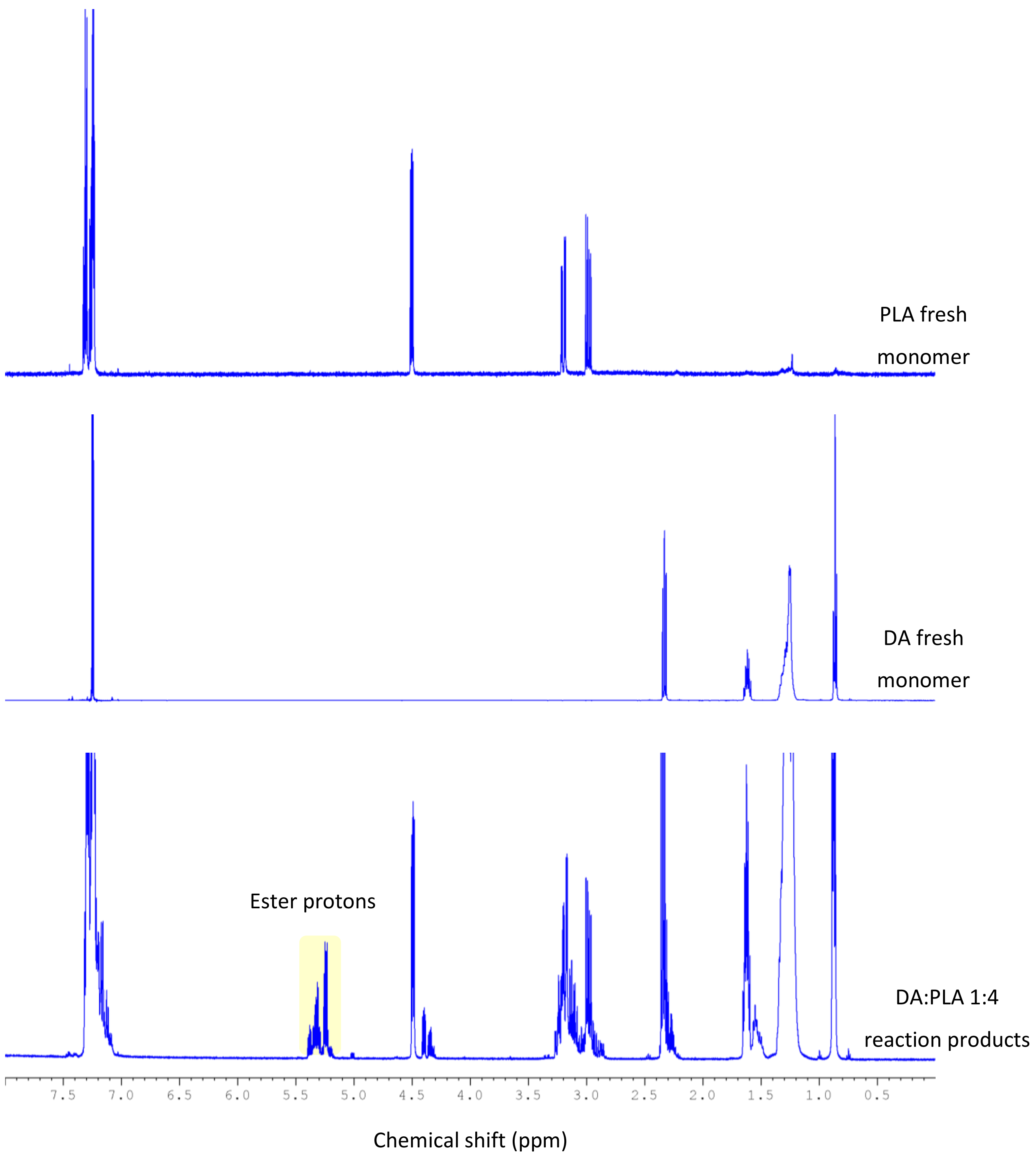

Figure S183. ^{1}H-NMR spectrum of DA:PLA reaction product at 1:4 molar ratio. The dry reaction product was suspended in $CDCl_3$ and analyzed by ^{1}H-NMR. DA and PLA monomers in $CDCl_3$ are presented for reference. Alpha protons shift downfield indicates the formation of ester bonds (highlighted in yellow).for reference.

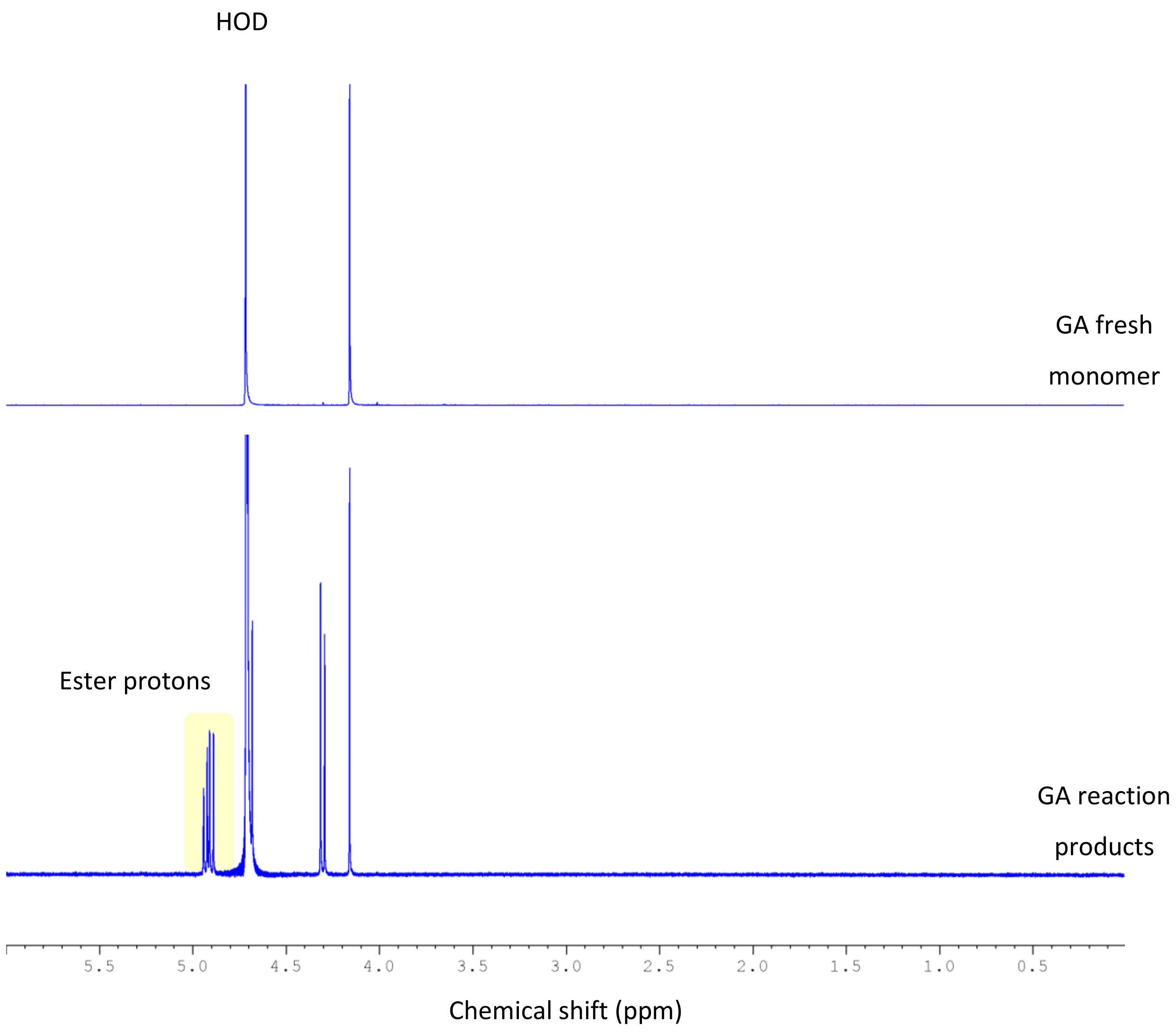

Figure S184. ^{1}H-NMR spectrum of GA control reaction products. The dry reaction product was suspended in D_2O and analyzed by ^{1}H-NMR. GA monomer in D_2O is presented for reference. Downfield shift of the alpha protons indicates the formation of ester bonds (highlighted in yellow).

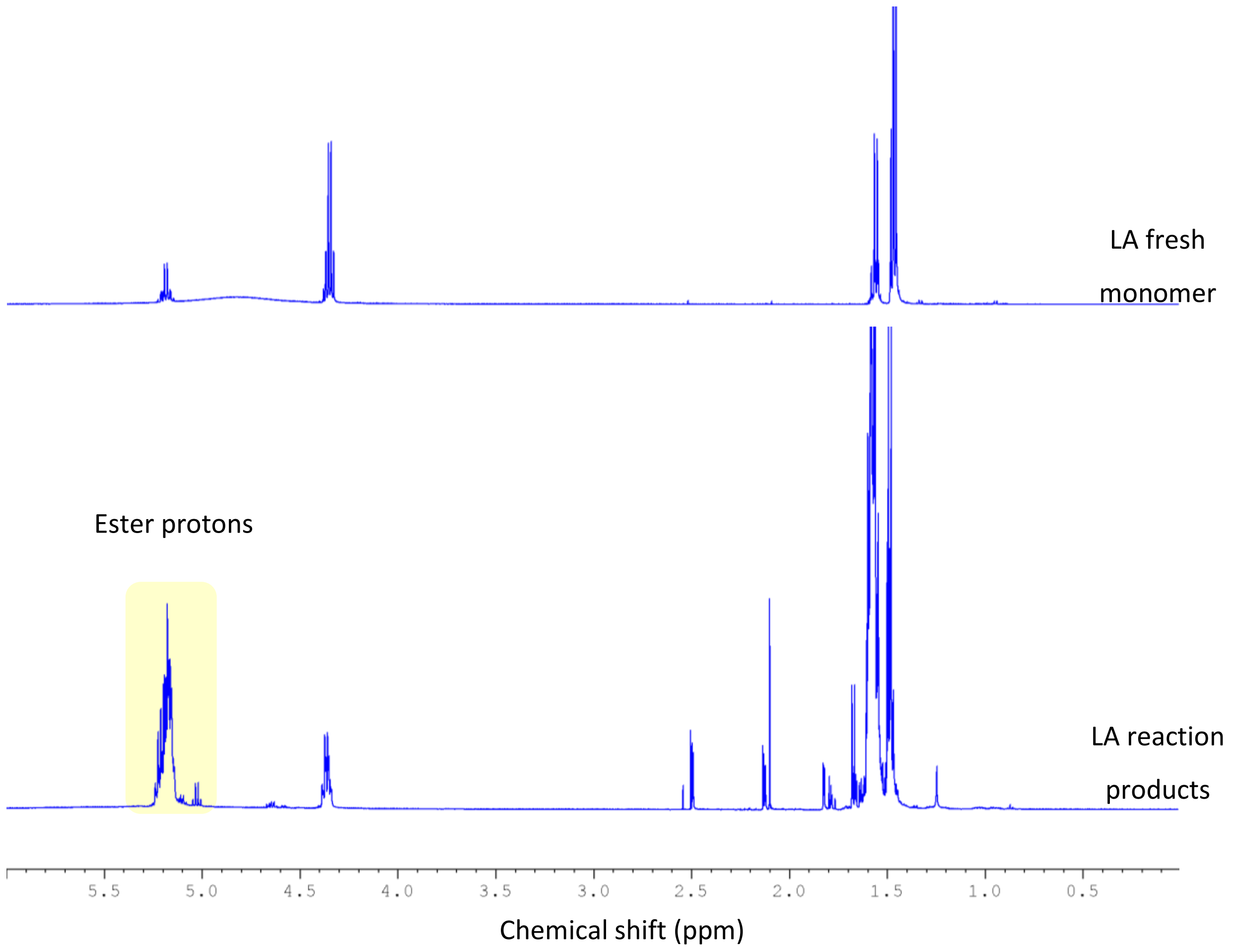

Figure S185. ^{1}H-NMR spectrum of LA control reaction product. The dry reaction product was suspended in $CDCl_3$ and analyzed by ^{1}H-NMR. LA monomer in $CDCl_3$ is presented for reference. Downfield shift of the alpha proton indicates the formation of ester bonds (highlighted in yellow).

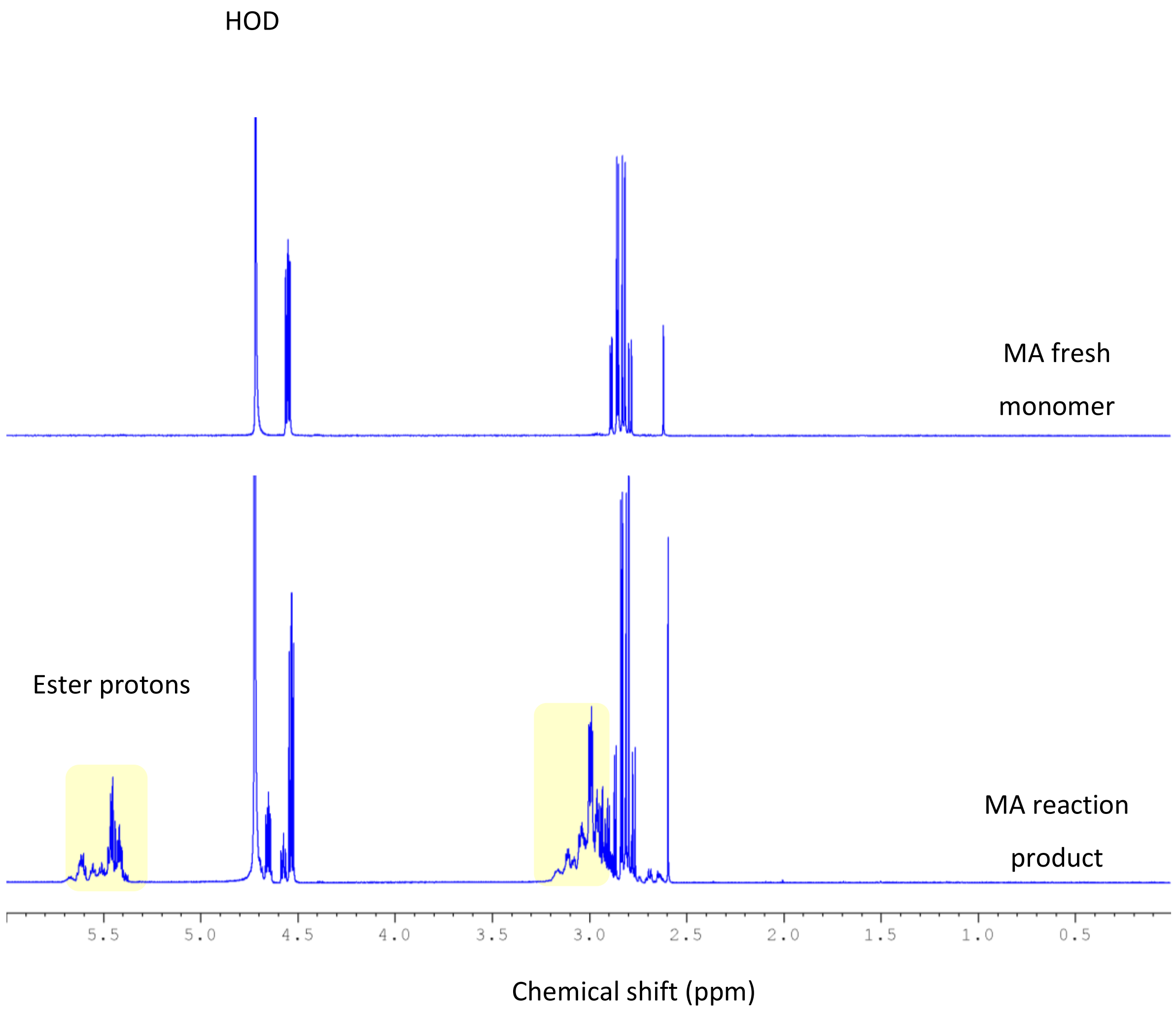

Figure S186. ^{1}H-NMR spectrum of MA control reaction product. The dry reaction product was suspended in D_2O and analyzed by ^{1}H-NMR. MA monomer in D_2O is presented for reference. Downfield shift of the alpha proton indicates the formation of ester bonds (highlighted in yellow).

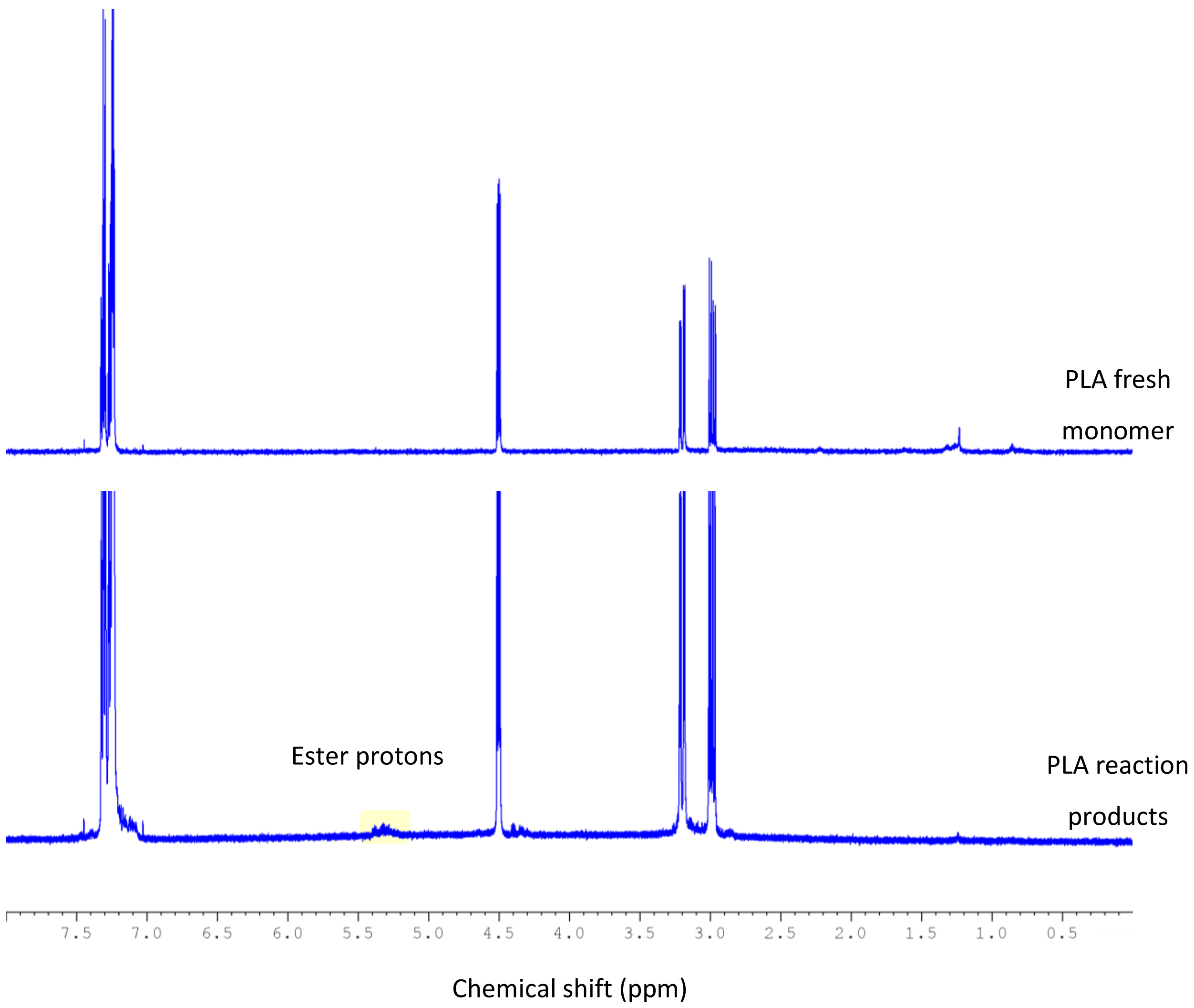

Figure S187. ^{1}H-NMR spectrum of PLA control reaction product. The dry reaction product was suspended in $CDCl_3$ and analyzed by ^{1}H-NMR. PLA monomer in $CDCl_3$ is presented for reference. Downfield shift of the alpha proton indicates the formation of ester bonds (highlighted in yellow).

A

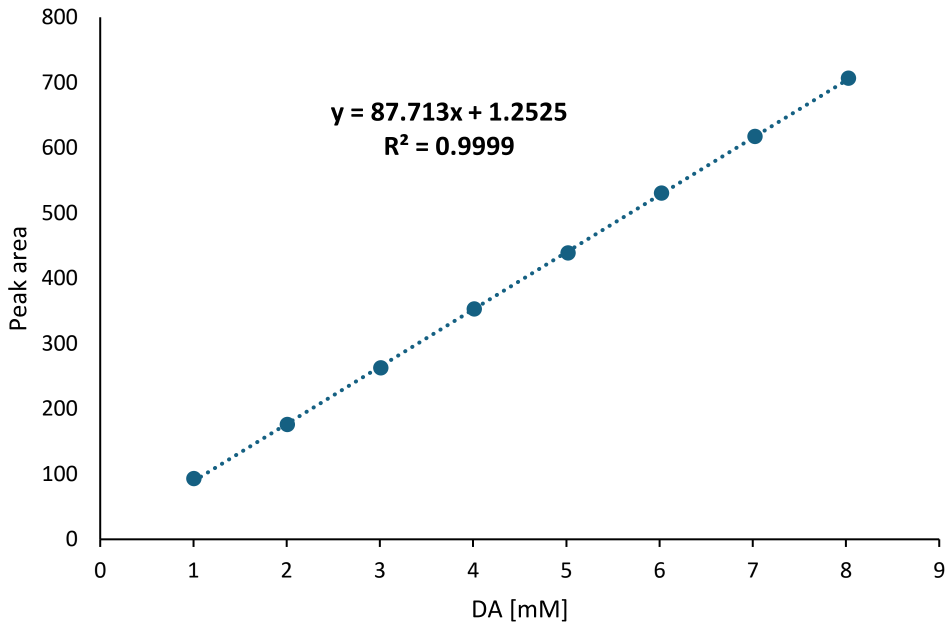

B

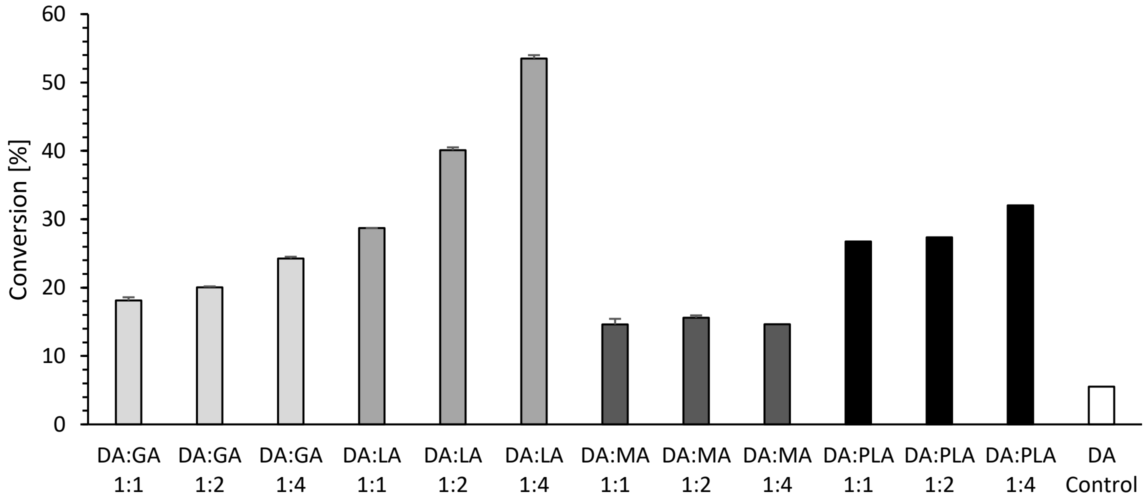

Figure S188. DA consumption under dry reaction at 85°C for 7 days determined by HPLC. Calibration curve was constructed at 210nm for the determination of DA conversion (A) .DA has been consumed when introduced to all HAs under the reaction conditions (B). Error bars represent standard deviation of three independent preparations.

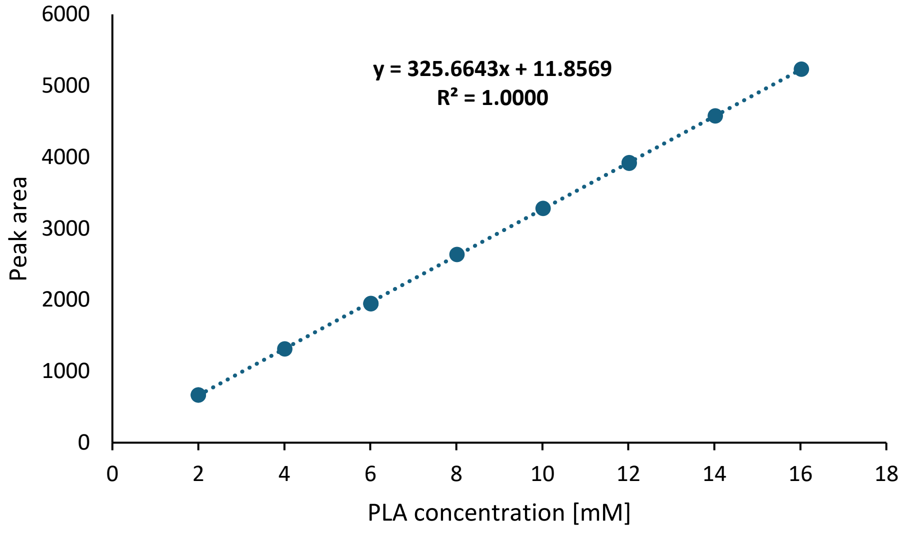

Figure S189. Calibration curve at 259nm constructed for the determination of PLA conversion.

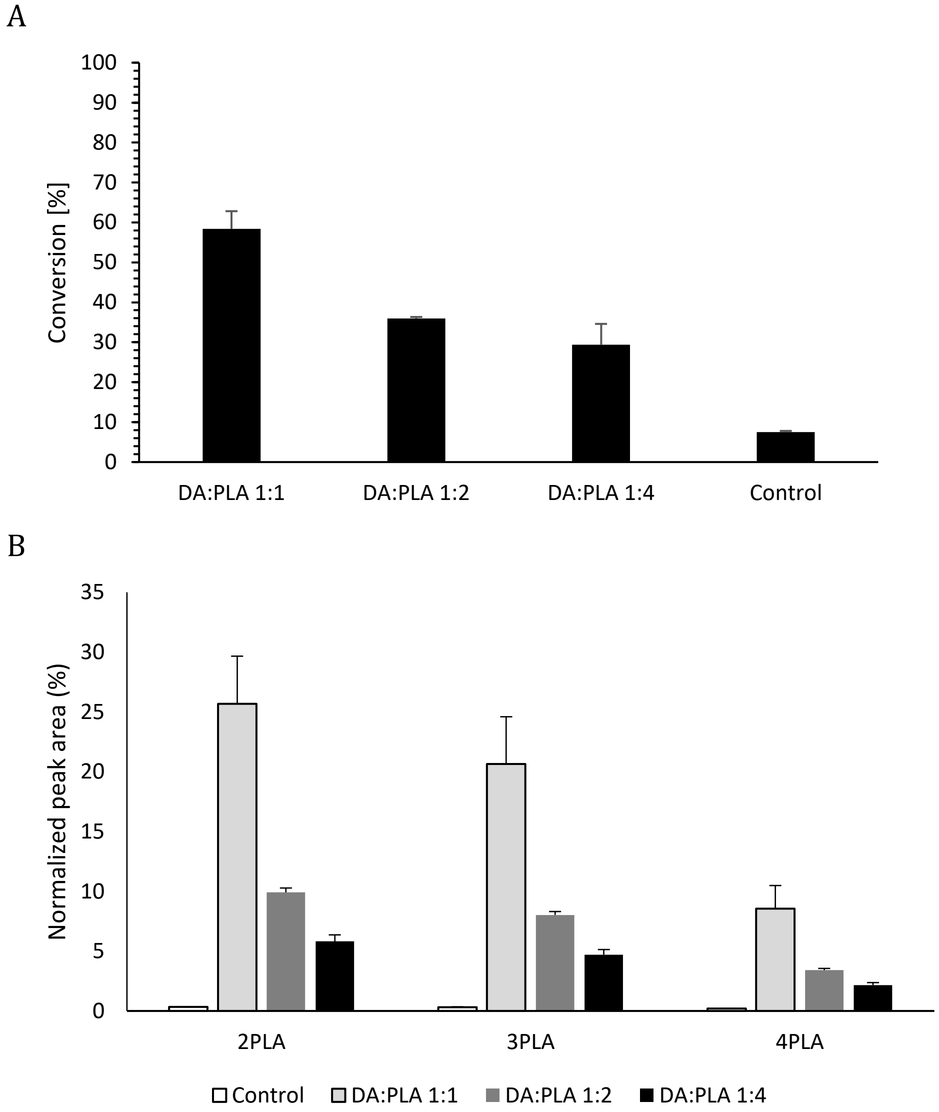

Figure S190. PLA consumption under dry reaction at 85°C for 7 days. The conversion of PLA following the reaction in the presence and absence ('control') of DA at PLA:DA 1:1, 1:2, and 1:4 molar ratios (A). The relative abundance of PLA dimer (2PLA), trimer (3PLA), and tetramer (4PLA) obtained by the reaction of PLA in the presence and absence of DA (B). The relative abundance was calculated as the ratio of homoesters peak area to PLA peak area at 259 nm. The presence of DA resulted in significant increase in the conversion of PLA into products. The formation of PLA homoesters in the presence of DA was also enhanced. The enhancement was inversely proportional to DA:PLA molar ratio exhibiting the highest PLA conversion and PLA homoesters formation at 1:1 molar ratio, following by 1:2 and 1:4 molar ratio. This effect is likely attributed to increased mobility of PLA molecules in the presence of DA molecules which are probably partially solubilizing PLA molecules. Error bars represent standard deviation of three independent preparations.

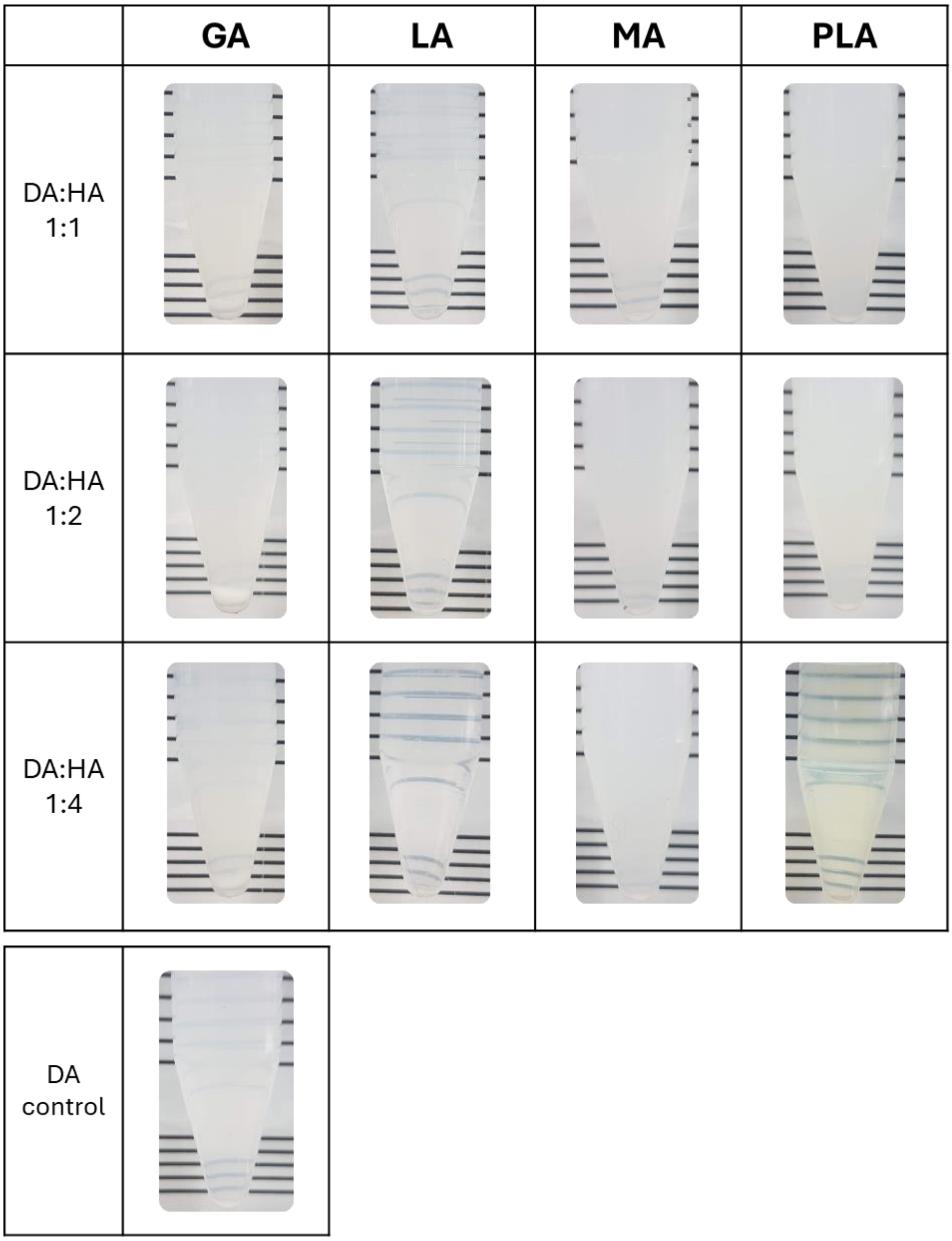

Figure S191. Visual appearance of rehydrated DA-HA reaction products at 1:1, 1:2, and 1:4 molar ratio. All samples were rehydrated in phosphate buffer (50mM) and their pH was adjusted to 6.8. The concentration of DA and the HAs were 50 mM and either 50, 100, or 200mM referring to initial amounts prior to the reaction. Reaction products of DA and GA contained insoluble GA oligomers as indicated in the figure. DA-LA reaction products exhibited increasing transparency as LA concentration was increased. DA-MA reaction products exhibited the opposite trend with increasing turbidity as MA concentration increased. DA-PLA products were turbid with one exception of DA:PLA at 1:4 molar ratio for which the rehydrated product was highly unstable and underwent phase separation into two liquid phases almost immediately.

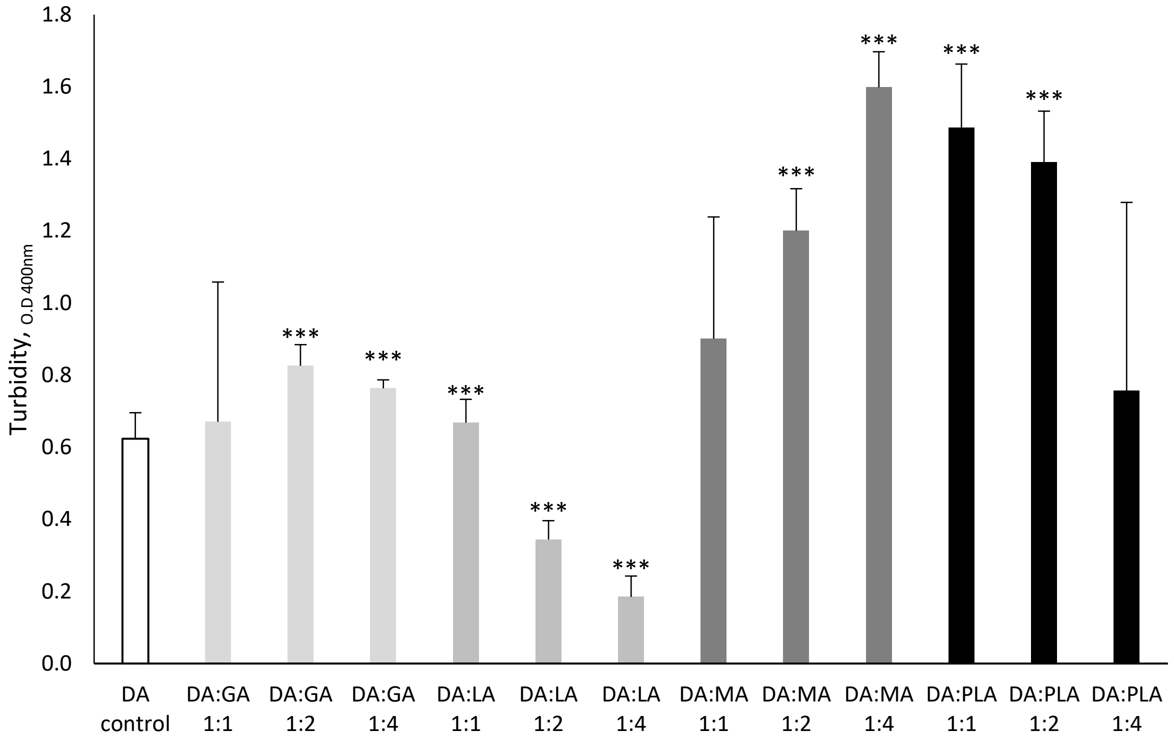

Figure S192. Turbidity meausrments of the rehydrated DA:HAs reaction products at 1:1, 1:2 and 1:4 molar ratio. All samples were rehydrated in phosphate buffer (50 mM) and their pH was adjusted to 6.8. The concentration of DA and the HAs were 50 mM and either 50, 100, or 200 mM, referring to initial amounts prior to the reaction. The turbidity (measured as the optical density at 400 nm) of the rehydrated reaction products is ratio-dependent. For GA and LA, the turbidity decreased as the DA:HA ratio increased. For MA the opposite trend was observed. In the case of PLA, a steep decrease in the turbidity was observed at DA:PLA 1:4 molar ratio, which is due to the rapid phase separation into two transparent liquids as also indicated in the visual assessment. Statisical significance was determined compared to DA control. Error bars represent standard deviation of three independent preparations.

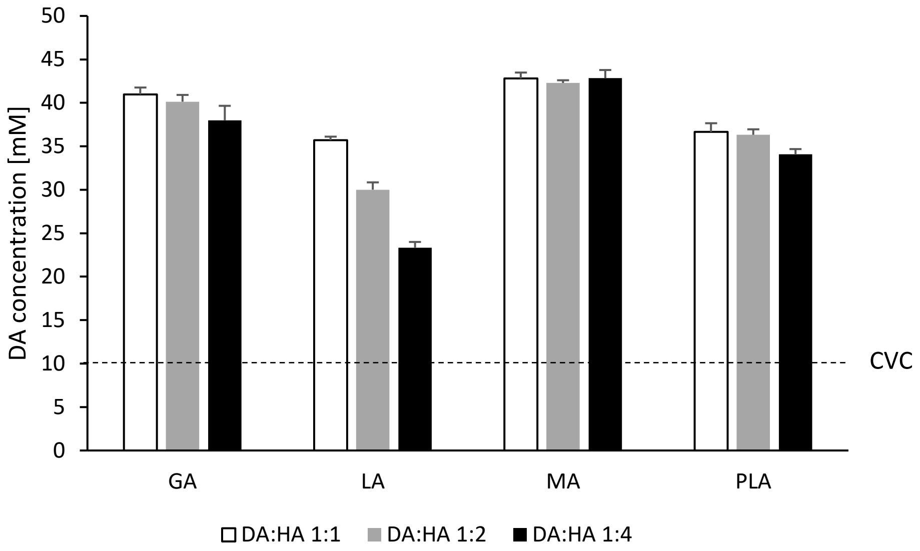

Figure S193. DA concentration in the resulting rehydrated products. All products were rehydrated in phosphate buffer (50 mM) and their pH was adjusted 6.8. The concentrations were calculated based on the measured conversion of DA in the reaction. The horizontal line at 10 mM represents the critical vesicular concentration of DA at the tested pH. Error bars represent standard deviation.

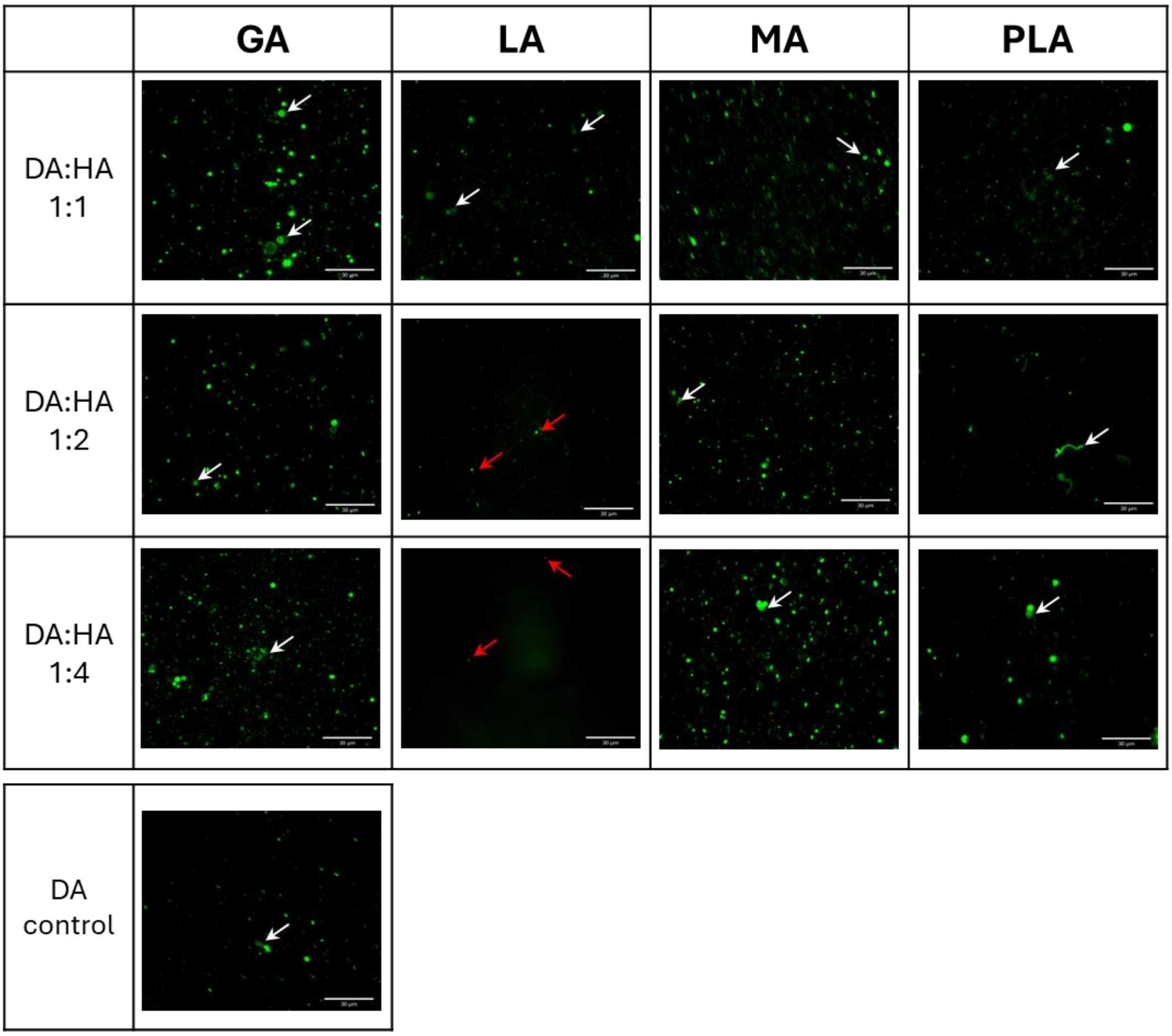

Figure S194. Fluorescent microscopy images of the rehydrated reaction products obtained by the reaction of DA and the tested HAs at different molar ratios. All samples were prepared in phosphate buffer (50 mM) at pH 6.8. The concentration of DA and the HAs were 50 mM and either 50, 100, or 200 mM, referring to the initial amounts prior to the reaction. Rhodamine 6G was used as a fluorescent probe. Structures were formed in all tested samples, as indicated by the images. Vesicles, indicated by the dying of the membrane (labeled with white arrows), were detected in all samples with the exceptions of DA:LA at 1:2 and 1:4 molar ratios, for which no clear membranous structures were detected. Nonetheless, small spherical structures were observed (labeled with red arrows). Scale bar is 30μm (x60 magnification).

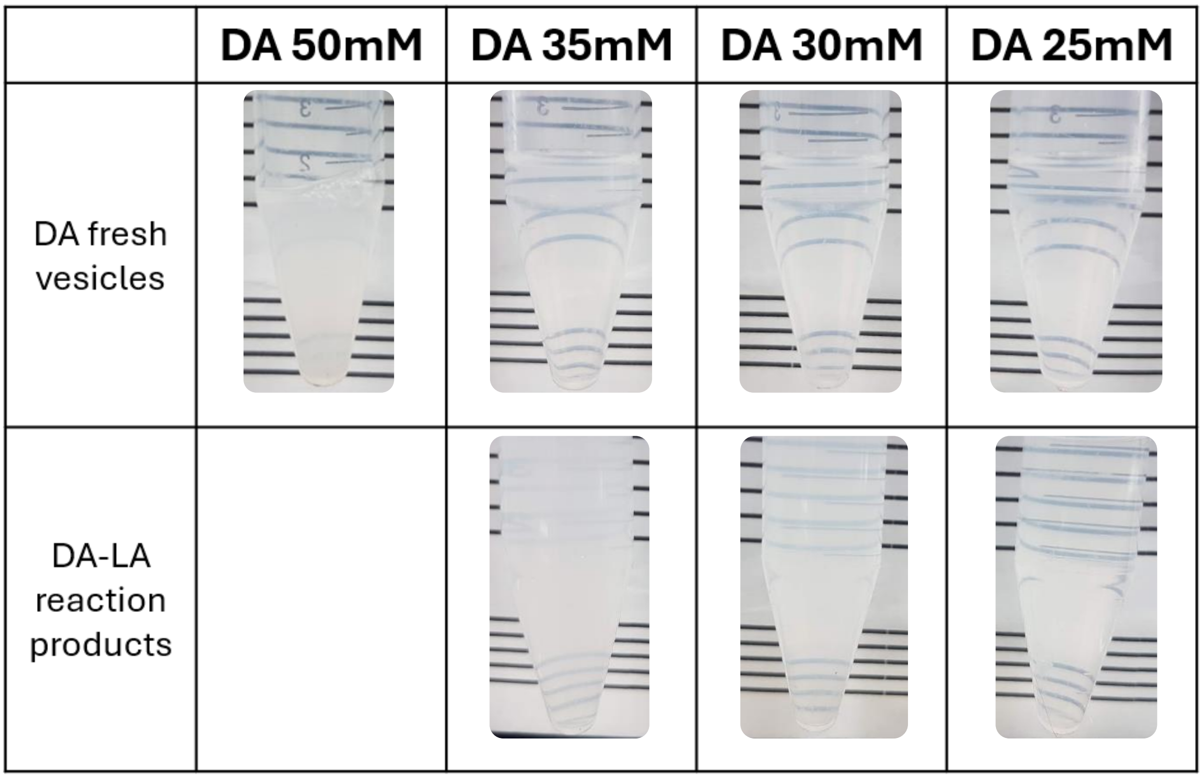

Figure S195. Visual appearance of DA fresh controls and DA:LA reaction products at 1:1, 1:2, and 1:4 molar ratios. DA fresh controls were prepared at either 50 mM, 35 mM, 30 mM, or 25 mM in phosphate buffer (50 mM) at pH 6.8. DA-LA reaction products were rehydrated in phosphate buffer (50 mM) at pH 6.8. DA final concentration in the resulting rehydrated products corresponds to 35 mM, 30 mM, and 25 mM on average for DA:LA at 1:1, 1:2, and 1:4 molar ratios, respectively. As indicated by the images, for DA fresh controls, the turbidity decreases as DA concentration decreases. For the reaction products, a similar trend was observed. While at 35 mM and 25 mM DA the reaction products appeared more turbid than the fresh controls, at 25 mM DA the turbidity of both fresh control and reaction products is comparable.

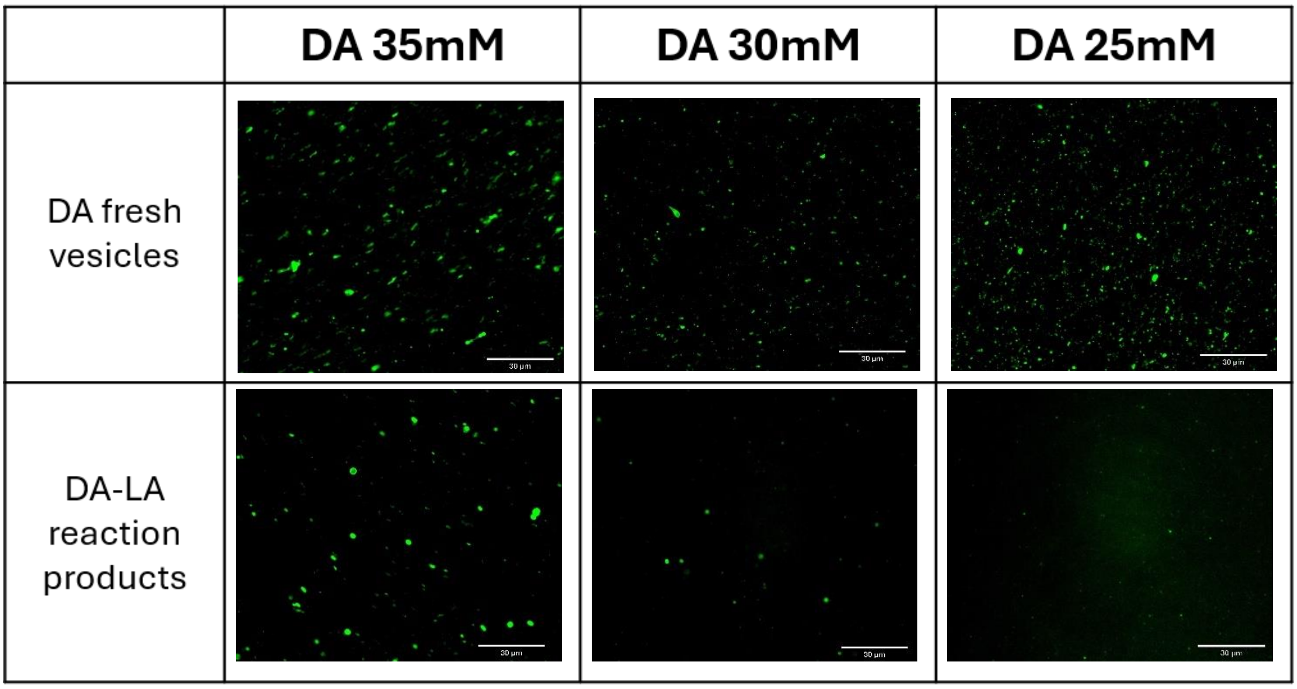

Figure S196. Fluorescent microscopy images of DA fresh controls at decreasing concentrations and DA:LA reaction products at 1:1, 1:2, and 1:4 molar ratios. DA fresh controls were prepared at either 35 mM, 30 mM, or 25mM in phosphate buffer (50 mM) at pH 6.8. DA-LA reaction products were rehydrated in phosphate buffer (50 mM) at pH 6.8. DA final concentration in the resulting rehydrated products corresponds to 35 mM, 30 mM, and 25 mM on average for DA:LA at 1:1, 1:2, and 1:4 molar ratios, respectively. Structures were observed in all samples. The extent of structures formation as indicated by the microscopy observation was relatively similar in the case of the samples of 35mM DA. However, at DA final concentrations of 30 mM and 25 mM, the distribution and number of structures was reduced in the reaction products compared to the fresh controls, suggesting that the aggregation properties of the DA-LA products mixture are different than those of DA. Scale bar is 30µm (x60 magnification).

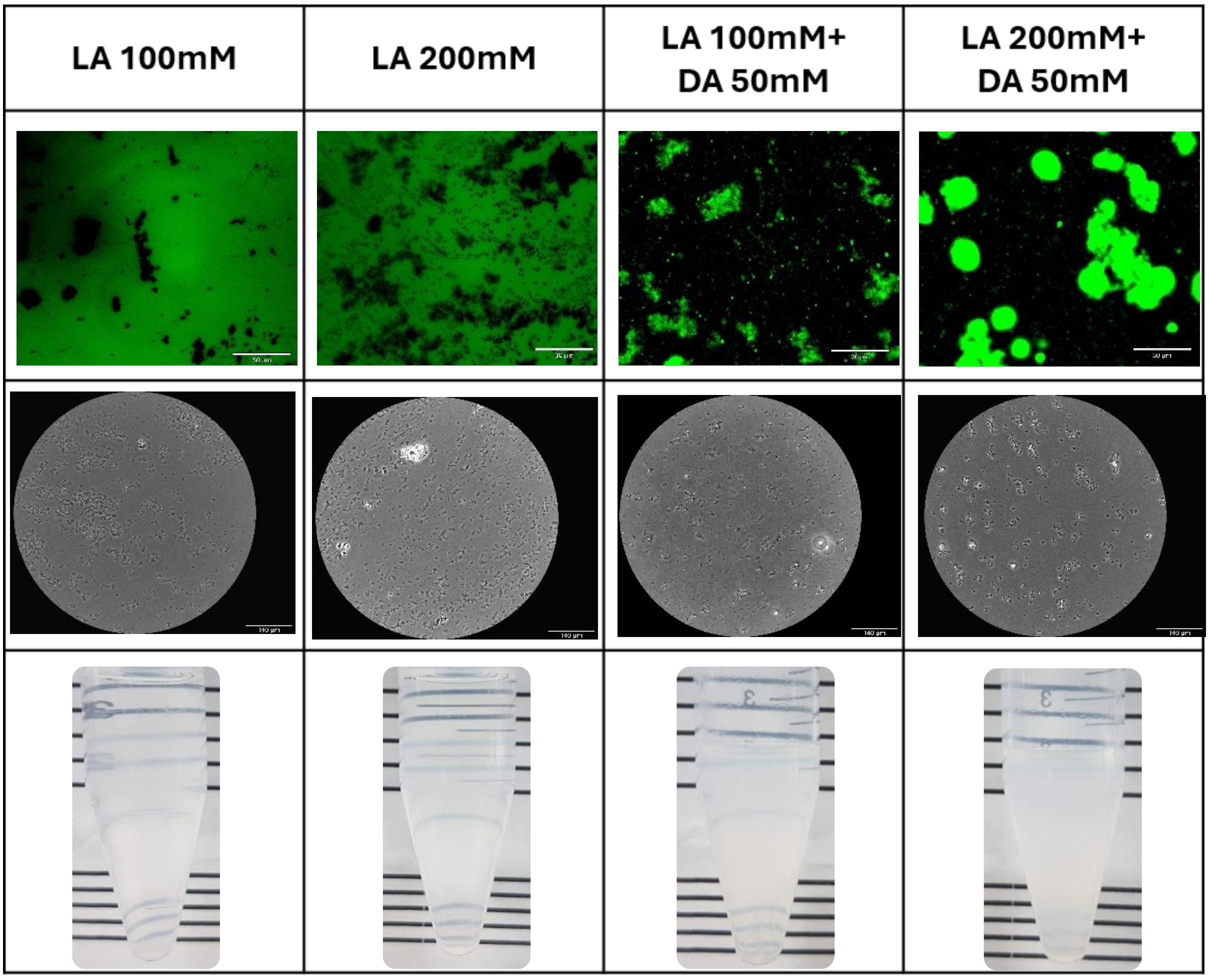

Figure S197. Fluorescent microscopy images , bright-field microscopy images, and visual appearance of LA reaction products at 100 mM and 200 mM in the presence and absence of 50 mM DA. For the samples in the absence of DA, LA reaction products were rehydrated in phosphate buffer (50 mM) at pH 6.8. In the presence of DA, LA reaction products were rehydrated in DA stock solution and phosphate buffer and pH was adjusted to 6.8. The concentration of LA was either 100 mM or 200 mM, referring to the initial amount prior to the reaction. When present, DA concentration was 50 mM. LA reaction products formed aggregates as indicated by the microscopy images. In the absence of DA, aggregates were not dyed by rhodamine 6G. When DA was added, vesicles were observed and aggregates formed by LA oligomers were dyed by the probe suggesting that DA adsorbed at the aggregates surface. Scale bar is 30μm (x60 magnification) for fluorescent microscopy images and is 140μm (x20 magnification) for brigh-field microscopy images.

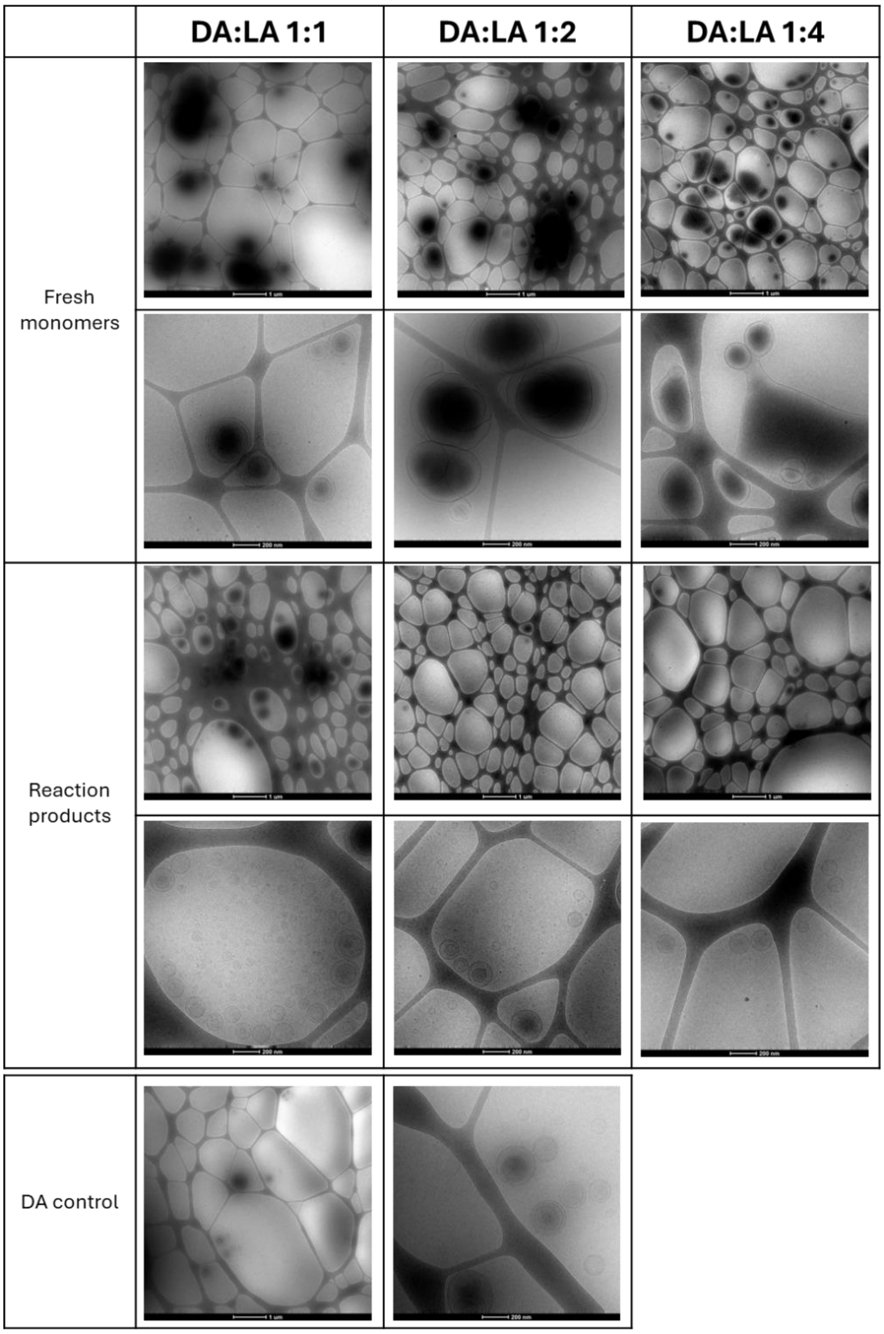

Figure S198. Cryo-TEM images of fresh monomers and reaction products of DA:LA at 1:1, 1:2, and 1:4 molar ratios. All samples were prepared in phosphate buffer (50 mM) at pH 6.8. The concentration of DA was 50mM, and the concentration of LA was either 50, 100, or 200 mM. In the case of the reaction products, DA and LA concentrations refer to the initial amounts prior to the reaction. The micrographs confirm the presence of vesicles in all samples. In the case of the fresh monomers, as the concentration of LA increased above 50mM, the average size of the observed vesicles increased, and the vesicles became less spherical and more deformed. In addition, the inner aqueous phase of the vesicles appears darker, possibly due to the presence of phosphate ions. In the case of the reaction products, at DA:LA molar ratios of 1:1 and 1:2 small vesicles of less than 100nm were abundant compared to vesicles of larger diameter. Remarkably, vesicles observed in DA:LA at 1:4 molar ratio were mostly unilamellar within 100-200nm. In general, the number of vesicles was signifcantly lower at DA:LA 1:4 molar ratio In this sample micelles of about 4-6nm were also observed. Note that the scale bar is the same (top raw is 1μm and bottom raw is 200nm) in images that are in the same row. For DA control sample the scale bars are 1μm and 200nm respectively.

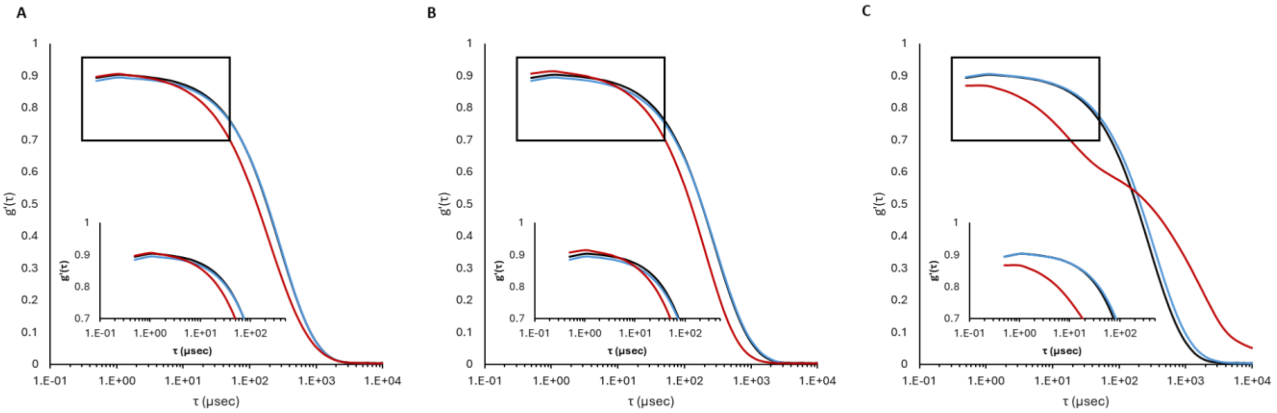

Figure S199. Dynamic light scattering correlograms of DA control, DA-LA fresh monomers, and DA-LA reaction products. Autocorrelation function, g'(τ) obtained for the DA control samples (black lines), DA-LA fresh monomers (blue lines), and DA-LA reaction products (red lines). A comparison of the autocorrelation functions obtained for DA control, DA:LA fresh monomers at a 1:1 molar ratio and DA:LA reaction products at a 1:1 molar ratio (A), a comparison of the autocorrelation functions obtained for DA control, DA:LA fresh monomers at a 1:2 molar ratio and DA:LA reaction products at a 1:2 molar ratio (B), and a comparison of the autocorrelation functions obtained for DA control, DA:LA fresh monomers at a 1:4 molar ratio and DA:LA reaction products at 1:4 molar ratio (C). As seen in panels A and B, the correlograms obtained for DA control and DA:LA fresh monomers at both 1:1 and 1:2 molar ratios almost entirely overlap, indicating that for both ratios, the fresh monomer samples and DA control are relatively similar in terms of both average size and polydispersity. The corresponding DA:LA reaction products at 1:1 and 1:2 molar ratios are however, distinct. For both reaction products, the decay starts earlier (i.e., smaller τ), suggesting that the average size is smaller compared to DA control and the corresponding DA-LA fresh monomers. The rate of decay (decay's slope) in the case of DA:LA at a 1:1 molar ratio is milder compared to DA control and DA-LA fresh monomers, indicating that the reaction products at a 1:1 molar ratio are more polydisperse. However, in the case of DA:LA reaction product at a 1:2 molar ratio, the decay is only slightly steeper, suggesting that at a 1:2 molar ratio, the reaction products are slightly less polydisperse compared to DA control and the corresponding fresh monomers. The trends observed for DA:LA samples at a 1:4 molar ratio are presented in panel C. All three samples exhibit different correlograms with the correlogram of DA-LA reaction products being indisputably distinct. When comparing the correlograms of DA control and DA:LA fresh monomers at a 1:4 molar ratio, one can see that for DA control, the decay begins earlier and has a slightly steeper slope compared to DA-LA fresh monomers, suggesting that both average size and polydispersity are smaller in DA control compared to DA-LA fresh monomers. As for the reaction products, two decays appear in the correlogram, indicating that there are two significantly different size populations. The first decay is consistent with a population of smaller structures, and the second decay represents a population of larger structures.

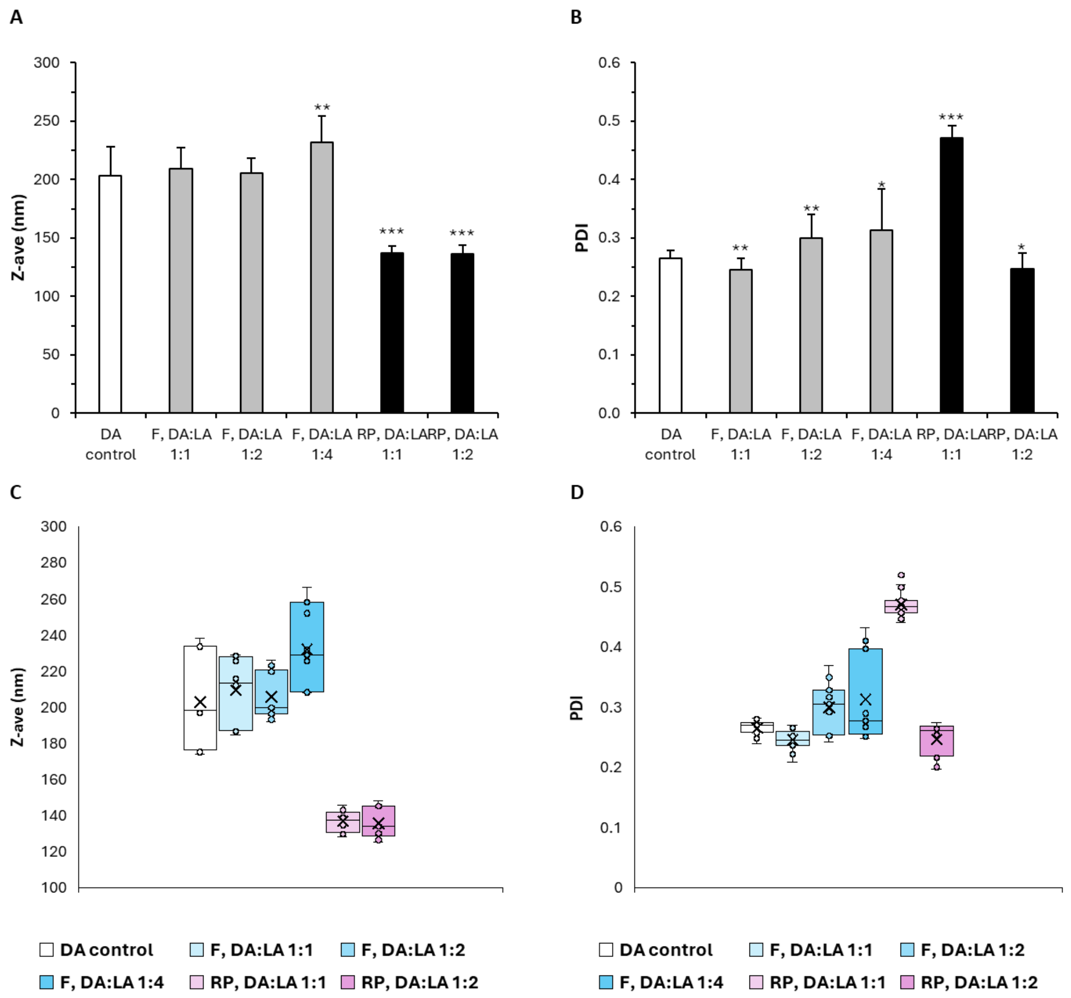

Figure S200. Mean size and polydispersity obtained for DA-LA reaction products and fresh monomers. Averaged Z-ave (A) and PDI (B) calculated from the cumulant approximation for DA control, DA:LA fresh monomers at 1:1, 1:2 and 1:4 molar ratios, and DA:LA reaction products at 1:1 and 1:2 molar ratios. Error bars represent standard deviation of three independent preparations. Boxplot representations of Z-ave (C) and PDI (D) obtained for all individual measured values (N=15). As indicated by panel A, while DA:LA fresh monomers at 1:1 and 1:2 molar ratios have a comparable size to DA control, for the 1:4 molar ratio, the Z-ave is significantly larger. In contrast, DA:LA reaction products at 1:1 and 1:2 molar ratios have a smaller Z-ave, indicating that the presence of the DA-conjugated oligoesters resulted in a decrease in vesicle size. The PDI values (panel B), which describe the degree of polydispersity, presents a slightly different trend. When comparing the fresh monomers to DA control sample, one can find that for the DA:LA at a 1:1 Molar ratio, the PDI slightly decreases, while for the higher molar ratios it increases. Interestingly, the polydispersity of DA:LA reaction products at a 1:1 molar ratio was greater compared to DA control, while at a 1:2 molar ratio the polydispersity significantly decreased. Assuming that each sample can be approximated as a single population of particles, the plotbox representation provides information on the distribution of the measured parameter. In general, the distribution of individual measurements obtained for the reaction products was significantly narrower compared to both DA control and DA-LA fresh monomers. This indicates that the results obtained for reaction product samples are more reproducible compared to their corresponding control samples. In contrast, DA-LA fresh monomers, mostly DA:LA at the 1:4 molar ratio, showed wider distribution in both Z-ave and PDI values. Given that all samples were not treated by any means (i.e., filtration, extrusion etc.), one can expect greater variability in terms of size and consequently also in terms of polydispersity.

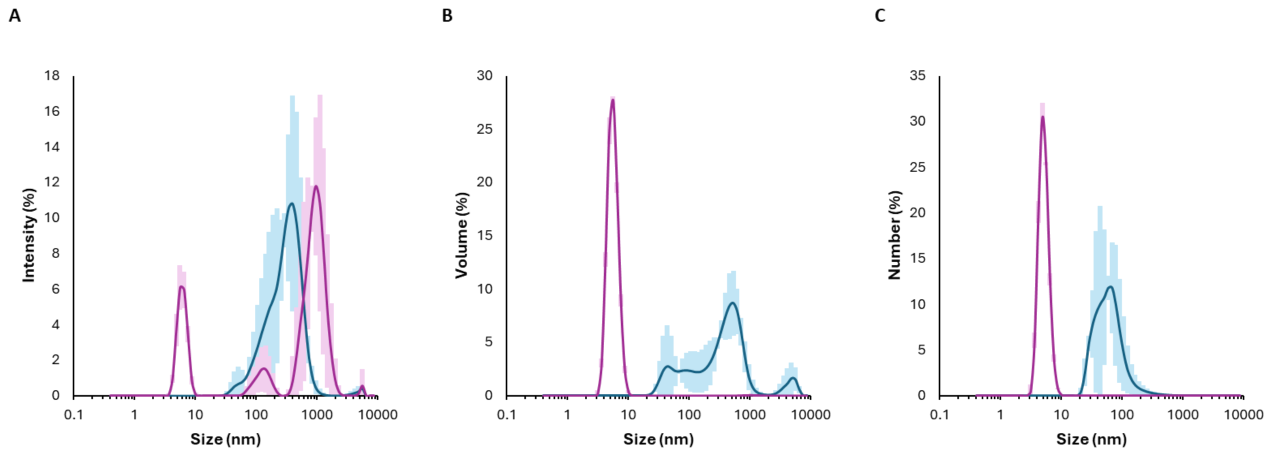

Figure S201. Size distribution obtained for DA:LA reaction products and fresh monomers at a 1:4 molar ratio. Intensity-weighted (A), volume-weighted (B) and number-weighted (C) distributions obtained for the fresh monomers (blue lines) and reaction products (purple lines) of DA:LA at a 1:4 molar ratio. The distributions are represented as the average of independent measurements. Vertical shaded bars represent standard deviation. As demonstrated by the intensity distribution, the reaction products exhibited three distinct populations, the two major populations are on the nanometric scale (ca 6 nm) and the micron scale (ca 1 µm). The fresh monomers contain a broad single peak with a maximum at around 350 nm. As the intensity-weighted distribution is sensitive to large particles and aggregates, it does not intuitively reflect the ratio between the different populations, and therefore volume and number distribution are commonly used. In the case of the reaction products, the translation of the intensity distribution into volume- and number-weighted distributions suggests that the major population in the samples is the nanometric one. In practice, this means that the number and volume occupied by the small particles are significantly greater than those of the larger population. This is in agreement with cryo-TEM images that confirm the presence of micelles. In the case of the fresh monomers, the volume and number distributions also suggest that smaller particles account for the majority of the total particles. However, in the fresh monomers, the smaller particles are mostly within the submicron region, which is not indicative of micelles but of small unilamellar vesicles (as also observed in cryo-TEM measurements).

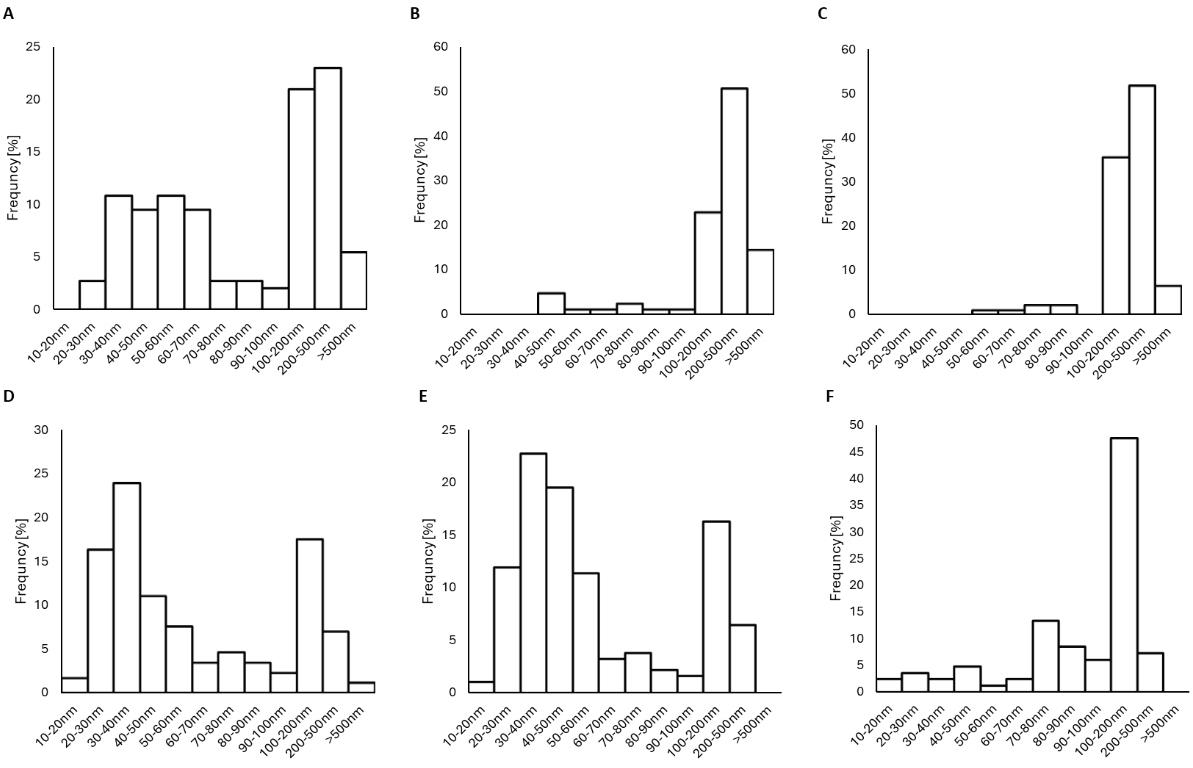

Figure S202. Vesicle size distribution derived from cryo-TEM measurements. Size distributions of vesicular structures obtained for fresh monomers of DA:LA at a 1:1 (n=148) (A), a 1:2 (n=83) (B) and a 1:4 (n=93) (C) molar ratios, and for reaction products of DA:LA at a 1:1 (n=172) (D), a 1:2 (185) (E) and a 1:4 (n=82) (F) molar ratios. For DA-LA fresh monomers, as the DA:LA molar ratio increased in favor of LA, fewer vesicles with diameters less than 100 nm were observed. In the case of DA:LA reaction products at 1:1 and 1:2 molar ratios, the majority of the observed vesicles were smaller than 100 nm, mostly in the range of 30-50 nm. In the case of DA:LA reaction products at a 1:4 molar ratio, the percentage of vesicles with a diameter larger than 100nm increased. However, in these samples, micelles were also observed, suggesting that micelle formation could have been at the expense of the smaller vesicles.

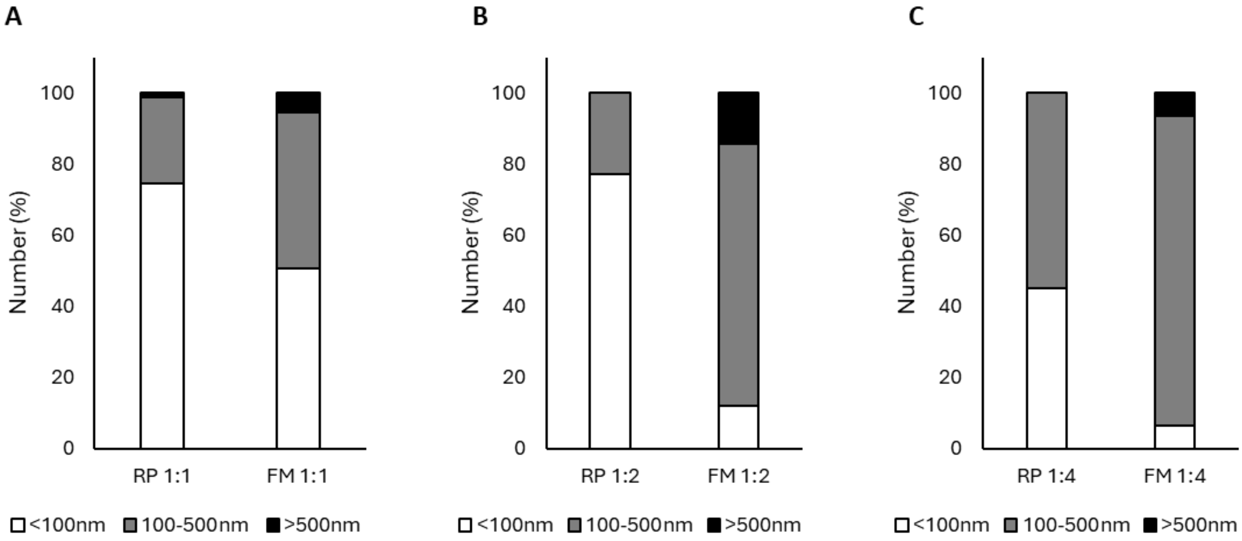

Figure S203. Vesicle size distribution derived from cryo-TEM measurements. Vesicle populations observed in DA-LA fresh monomers (FM) and reaction products (RP) at a 1:1 molar ratio (A), a 1:2 molar ratio (B), and a 1:4 molar ratio (C). Vesicle populations were classified into three groups of vesicles with diameters less than 100 nm (white box), within 100 to 500 nm (grey box) and above 500 nm (black box). For all molar ratios, the fraction of vesicles smaller than 100 nm is greater for the reaction products compared to the fresh monomers. The presence of vesicles larger than 500 nm was negligible for DA-LA reaction products. In the case of DA-LA fresh monomers, the vesicle population with a diameter larger than 500 nm accounted for 6-14%, which in some cases may be an underestimation since deformed vesicles, which were typically larger than 300 nm, were not included.

A

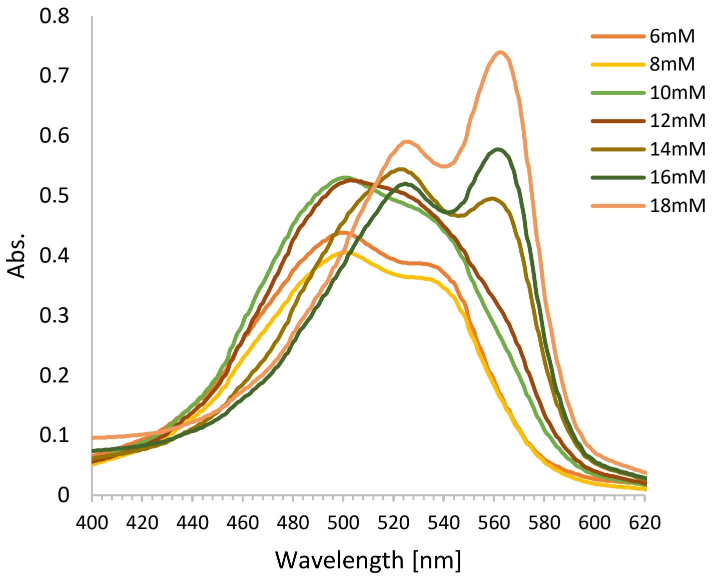

B

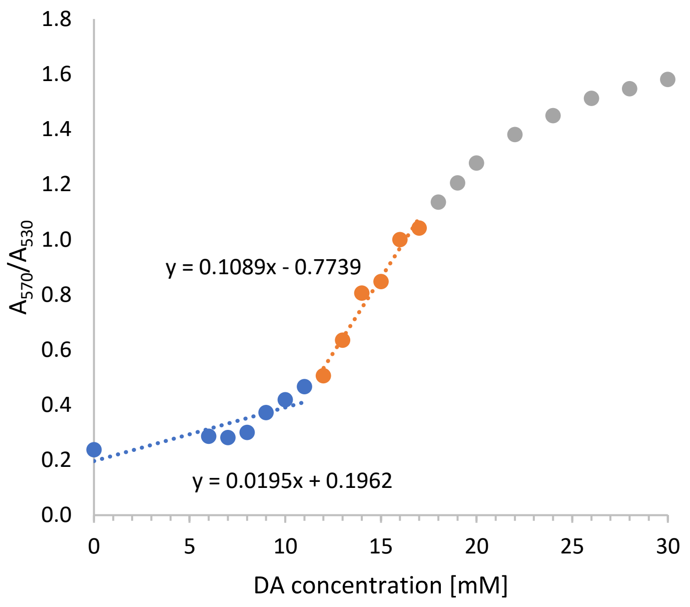

Figure S204. Determination of the critical aggregation concentration of DA for DA control sample. Absorption spectra of merocyanine 540 in the presence of increasing concentrations of DA obtained for DA control sample at pH 6.8 (A). Absorption ratio at 570 nm and 530 nm as a function of DA concentration. The intersection between the two fitting lines represents the CAC of DA – ca. 10 mM (B).

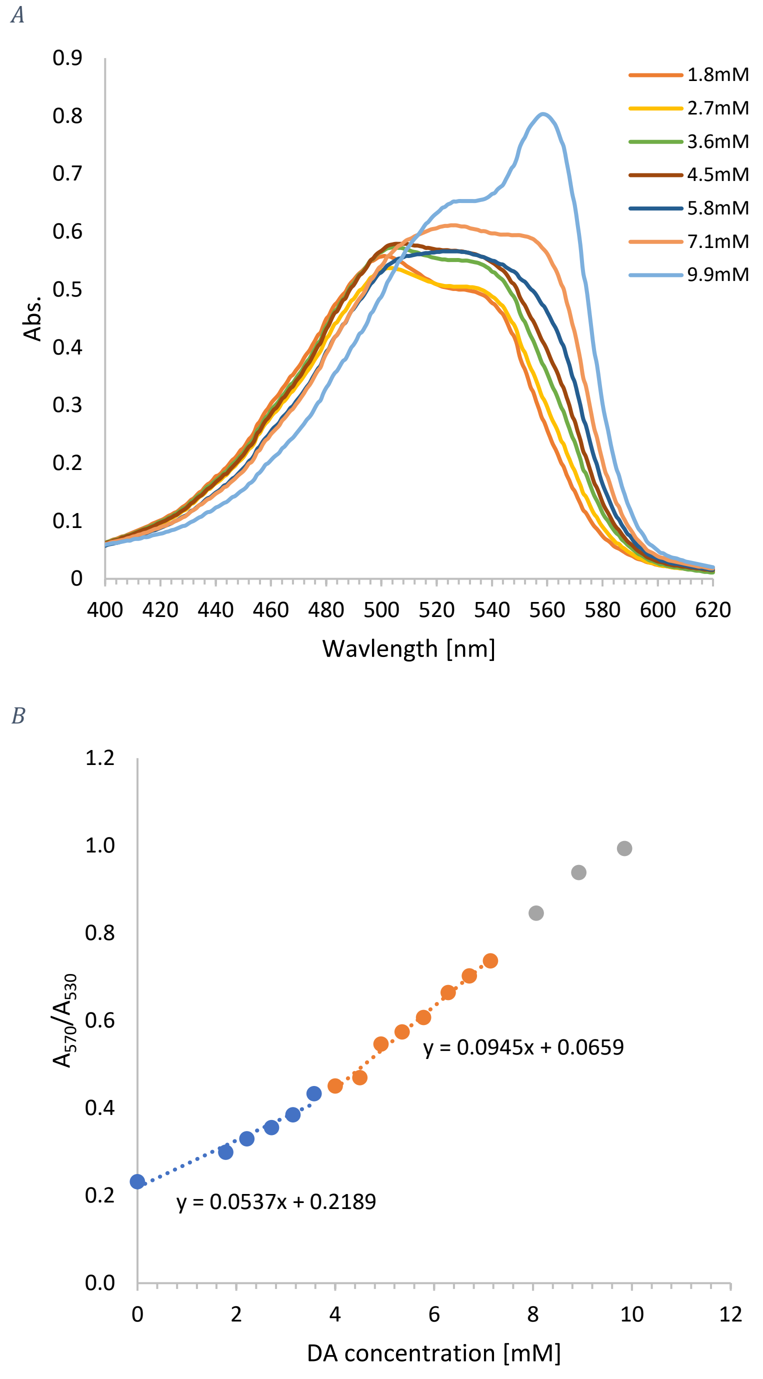

Figure S205. Determination of the critical aggregation concentration of DA for DA:LA reaction product at 1:1 molar ratio. Absorption spectra of merocyanine 540 in the presence of increasing concentrations of DA obtained for the reaction product of DA:LA at 1:1 molar ratio at pH 6.8 (A). Absorption ratio at 570 nm and 530 nm as a function of DA concentration. The intersection between the two fitting lines represents the CAC of DA – ca. 4 mM (B).

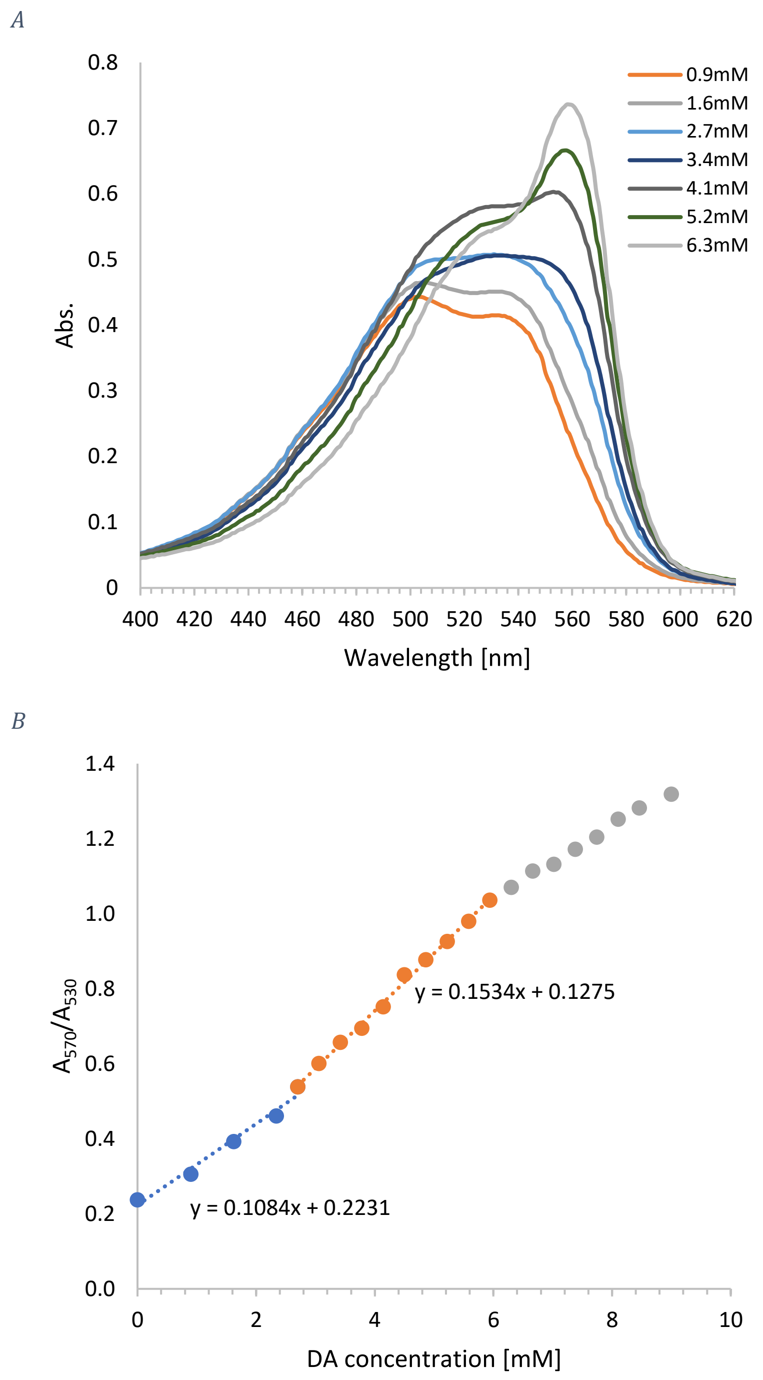

Figure S206. Determination of the critical aggregation concentration of DA for DA:LA reaction product at 1:2 molar ratio. Absorption spectra of merocyanine 540 in the presence of increasing concentrations of DA obtained for the reaction product of DA:LA at a 1:2 molar ratio at pH 6.8 (A). Absorption ratio at 570 nm and 530 nm as a function of DA concentration. The intersection between the two fitting lines represents the CAC of DA – ca. 2 mM (B).

A

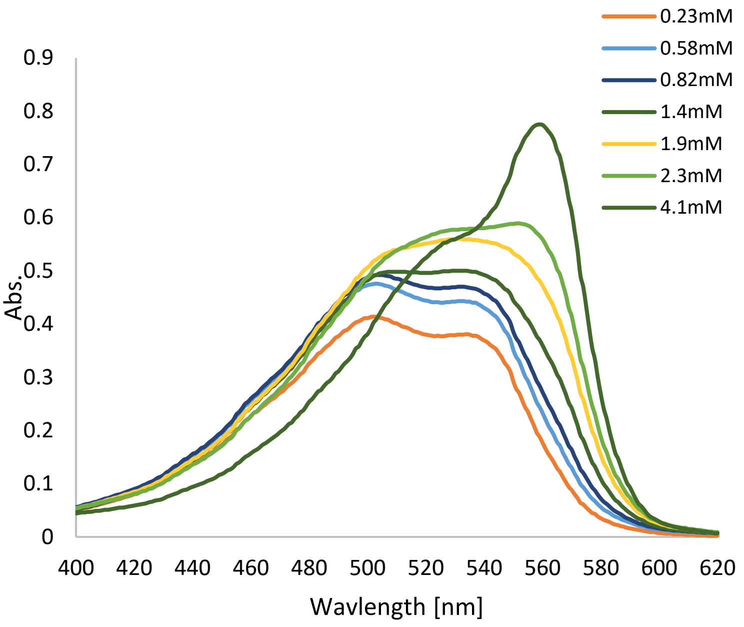

B

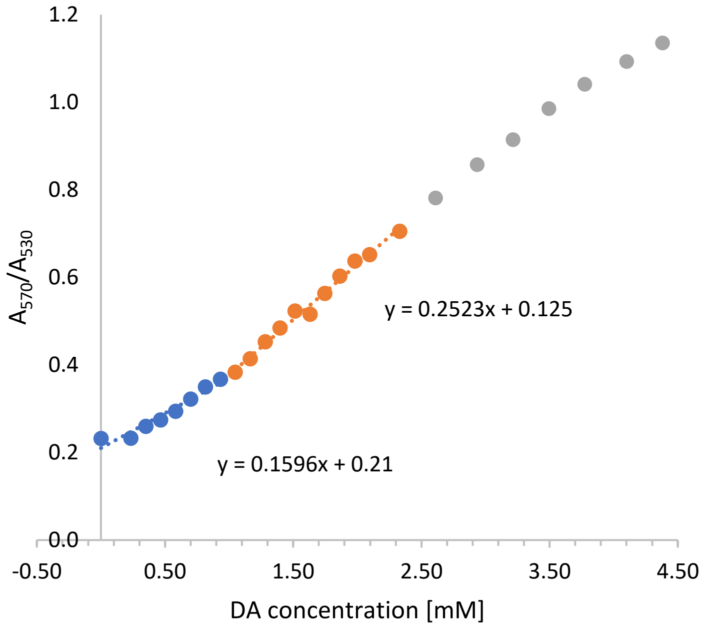

Figure S207. Determination of the critical aggregation concentration of DA for DA:LA reaction product at 1:4 molar ratio. Absorption spectra of merocyanine 540 in the presence of increasing concentrations of DA obtained for the reaction product of DA:LA at a 1:4 molar ratio at pH 6.8 (A). Absorption ratio at 570 nm and 530 nm as a function of DA concentration. The intersection between the two fitting lines represents the CAC of DA – ca 1 mM (B).

A

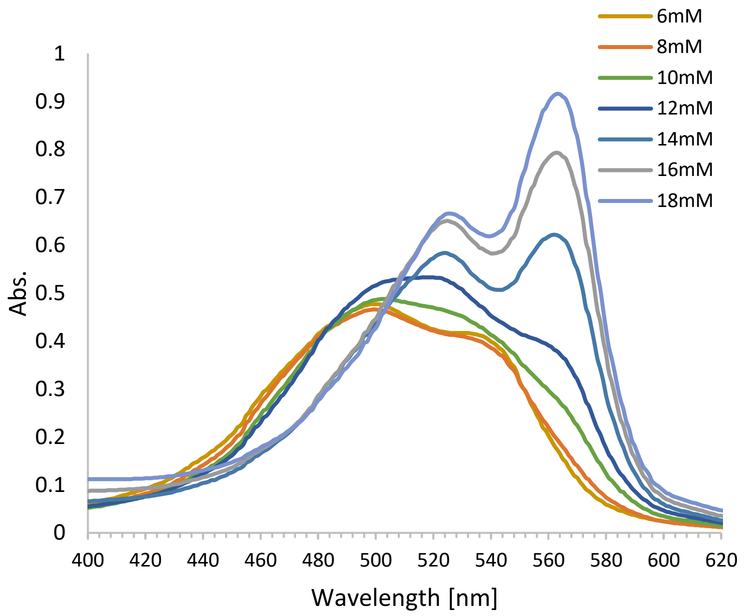

B

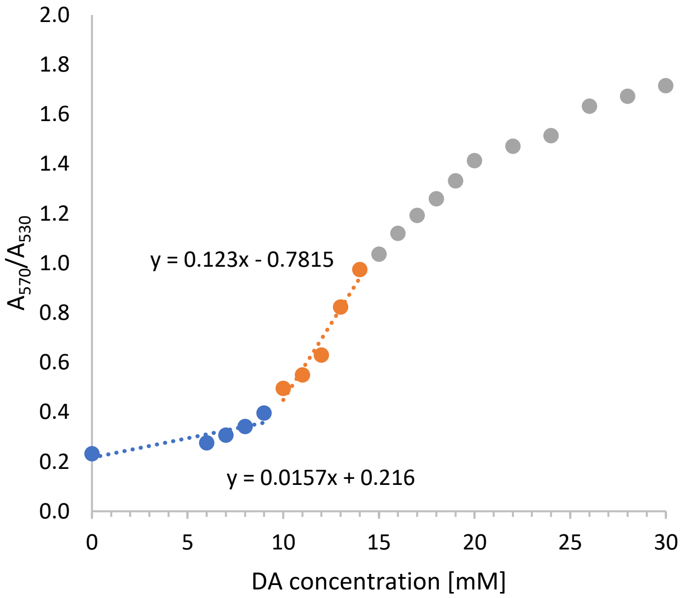

Figure S208. Determination of the critical aggregation concentration of DA for DA:LA fresh monomers at 1:2 molar ratio. Absorption spectra of merocyanine 540 in the presence of increasing concentrations of DA obtained for fresh monomers of DA:LA at 1:2 molar ratio at pH 6.8 (A). Absorption ratio at 570nm and 530nm as a function of DA concentration. The intersection between the two fitting lines represents the CAC of DA – ca. 10 mM (B).

A

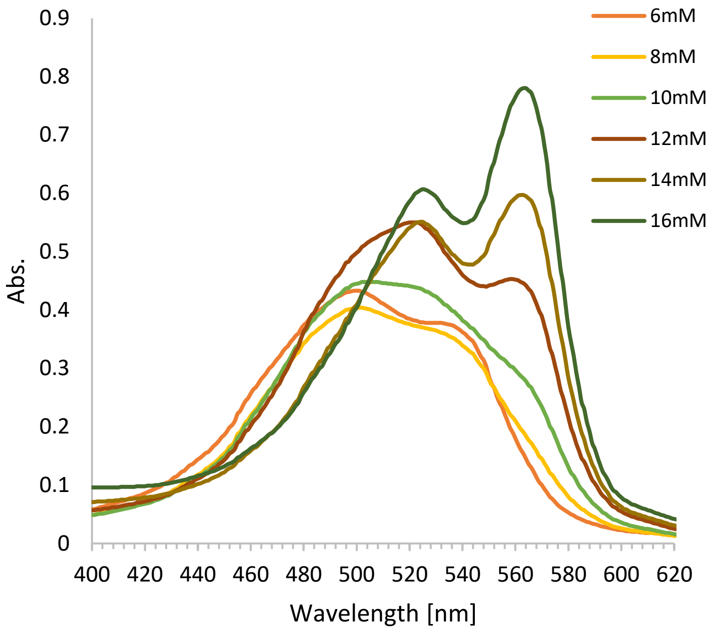

B

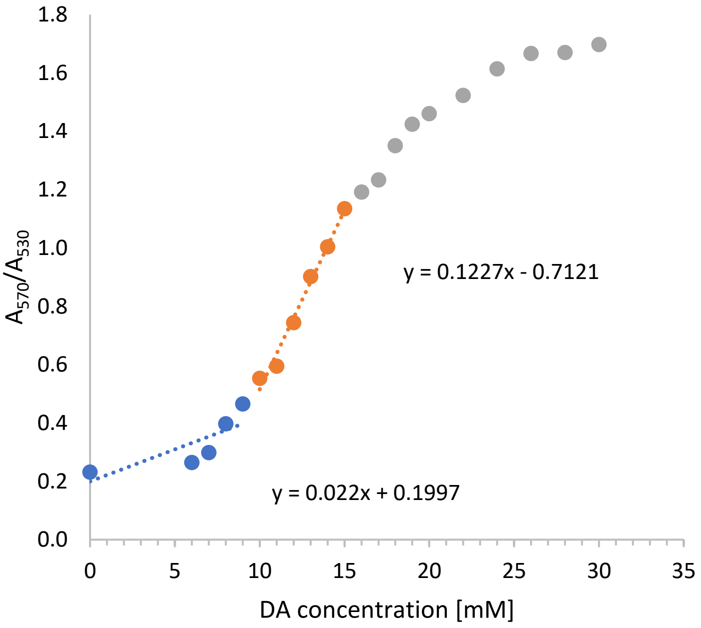

Figure S209. Determination of the critical aggregation concentration of DA for DA:LA fresh monomers at 1:4 molar ratio. Absorption spectra of merocyanine 540 in the presence of increasing concentrations of DA obtained for fresh monomers of DA:LA at 1:4 molar ratio at pH 6.8 (A). Absorption ratio at 570nm and 530nm as a function of DA concentration. The intersection between the two fitting lines represents the CAC of DA – ca. 9 mM (B).

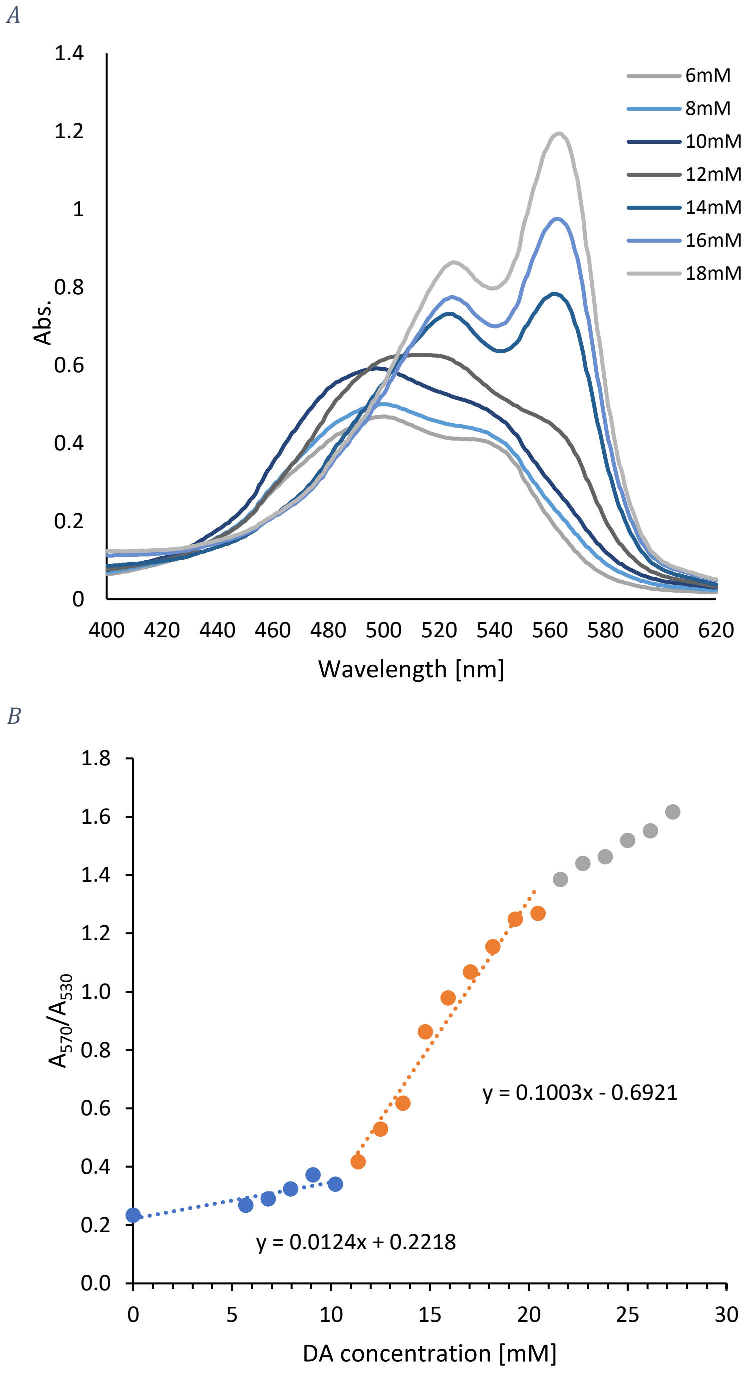

Figure S210. Determination of the critical aggregation concentration of DA for a mixture of DA and LA rection product at 1:2 molar ratio referring to initial amount of LA. Absorption spectra of merocyanine 540 in the presence of increasing concentrations of DA obtained for the reaction product LA into which DA was added at pH 6.8. The ratio between DA and LA was 1:2 referring to the initial amount of LA prior to the reaction (A). Absorption ratio at 570nm and 530nm as a function of DA concentration. The intersection between the two fitting lines represents the CAC of DA – ca. 10 mM (B).

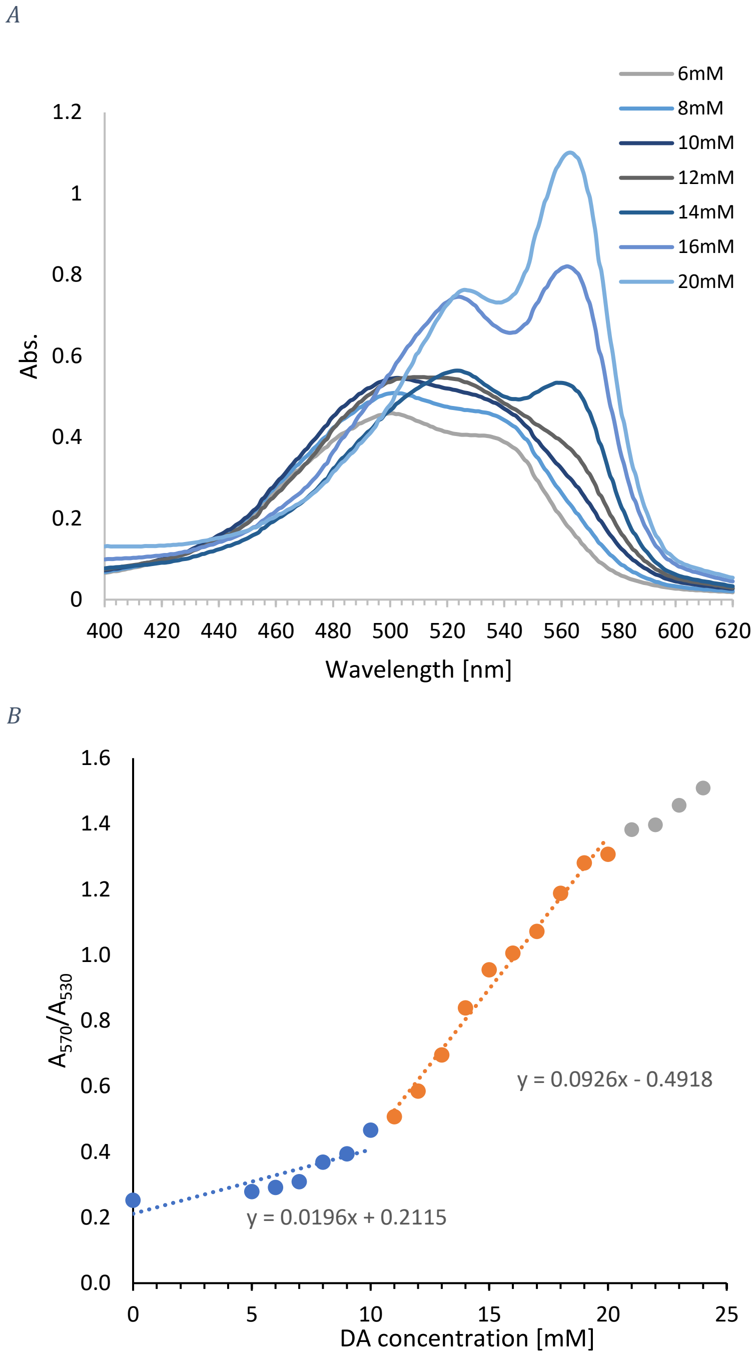

Figure S211. Determination of the critical aggregation concentration of DA for a mixture of DA and LA rection product at 1:4 molar ratio referring to initial amount of LA. Absorption spectra of merocyanine 540 in the presence of increasing concentrations of DA obtained for the reaction product LA into which DA was added at pH 6.8. The ratio between DA and LA was 1:4 referring to the initial amount of LA prior to the reaction (A). Absorption ratio at 570nm and 530nm as a function of DA concentration. The intersection between the two fitting lines represents the CAC of DA – ca. 10 mM (B).

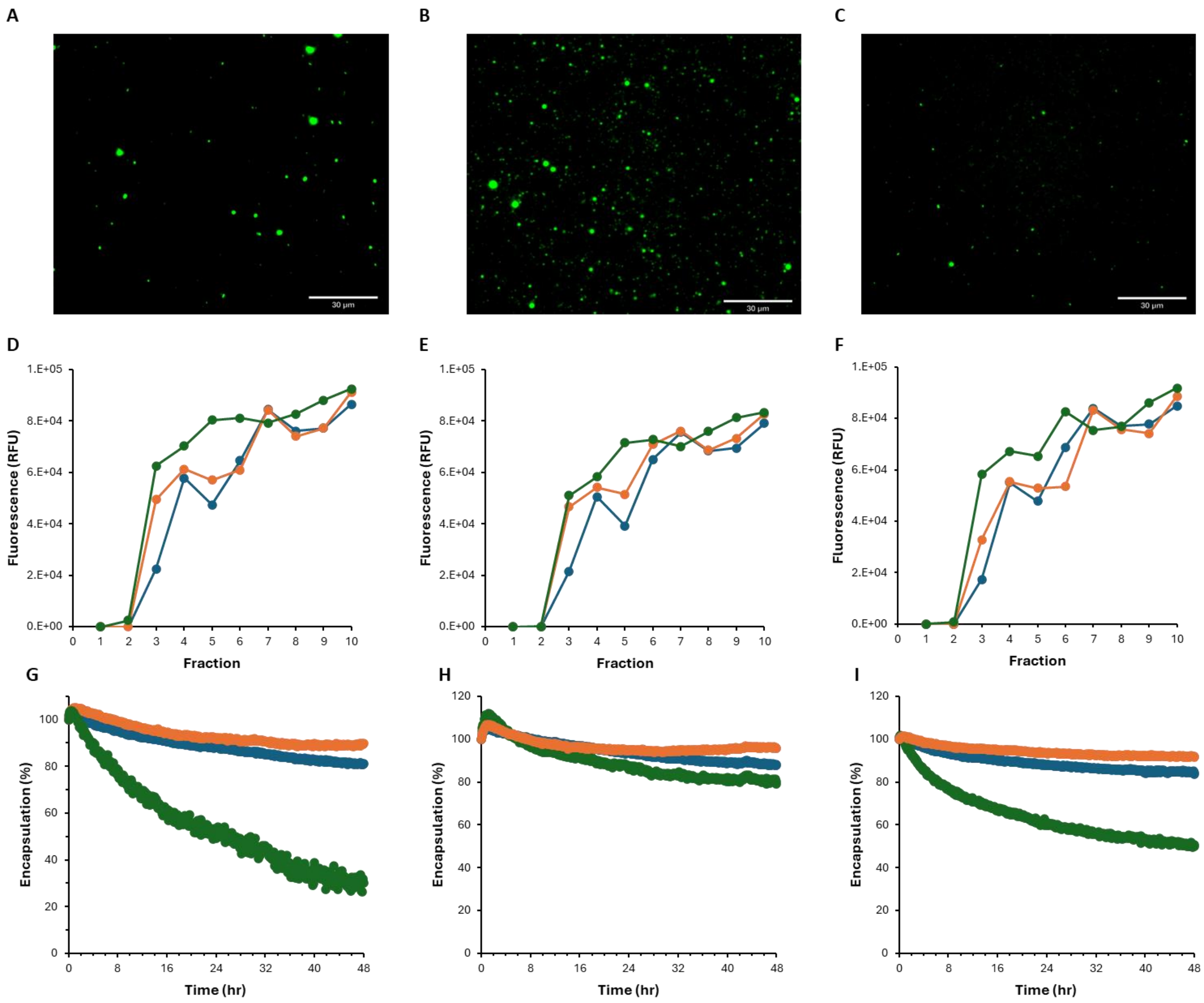

Figure S212. DA-LA reaction products exhibit greater permeability compared to the corresponding DA-LA fresh monomers and DA control vesicles. Fluorescence microscopy images of calcein-encapsulated vesicles obtained for DA control (A), fresh monomers of DA:LA at 1:4 molar ratio (B), and reaction products of DA:LA at a 1:4 molar ratio (C). As demonstrated in panels A-C, calcein was encapsulated within all tested samples. Fluorescence measurement of the fractions obtained by size exclusion chromatography (SEC) of three independent preparations of DA control (blue), DA-LA fresh monomers (orange) and DA-LA reaction products (green) (D-F). As indicated by panels D-F, fractions 3-5 represent the encapsulated calcein. Calcein encapsulation percentage obtained for three independent preparations of DA control (blue), DA-LA fresh monomers (orange) and DA-LA reaction products (green). The encapsulation percentage profiles suggest that calcian leakage is greater from DA-LA reaction products compared to both DA-LA fresh monomers and DA control. These results indicate that vesicles formed from DA-LA reaction products exhibit greater permeability, consistent with their smaller particle size. When isolating the calcein-encapsulated fractions from the reaction products, we could not rule out the possibility that micelles were also eluted. If micelles were present, they could alter membrane packing and facilitate calcein leakage.

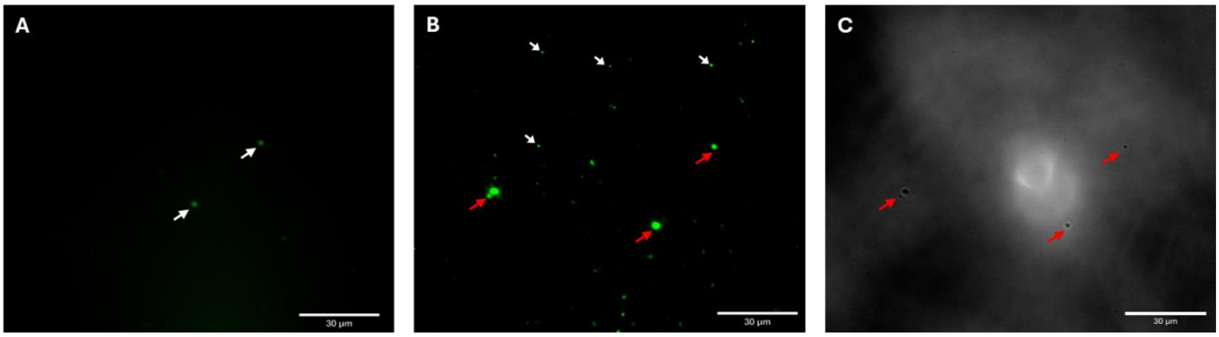

Figure S213. Encapsulation of biologically relevant molecules within DA-LA reaction products. Fluorescence microscopy images of FAM-labelled ssDNA (A) and FITC-labelled Poly-Lys (B) following their encapsulation within DA:LA reaction products at a 1:4 molar ratio. The corresponding brightfield image of FITC-labelled Poly-Lys following its encapsulation within DA:LA reaction products is shown (C). Encapsulation was examined at 50 mM DA, 200 mM LA (referring to their initial concentration prior to reaction initiation) and 50 mM phosphate buffer. For ssDNA, the pH was 6.8, while for poly-Lys, the pH was within the range of 6.7-7.0. As indicated in panel A, ssDNA was successfully encapsulated within the DA-LA reaction products. In the case of poly-Lys, precipitation was observed upon pH adjustment. To remove poly-Lys precipitates, the sample was centrifuged at 10,000 rpm for 2 min, and the supernatant was examined. Aggregates, likely resulting from poly-Lys precipitation, were observed (red arrows) along with spherical structures (white arrows). Due to the large size (~42 kDa) and the strong positive charge of poly-Lys, it is possible that it did not undergo encapsulation but rather adsorbed onto the outer surface of the vesicles due to electrostatic interactions with the negatively charged lipids.

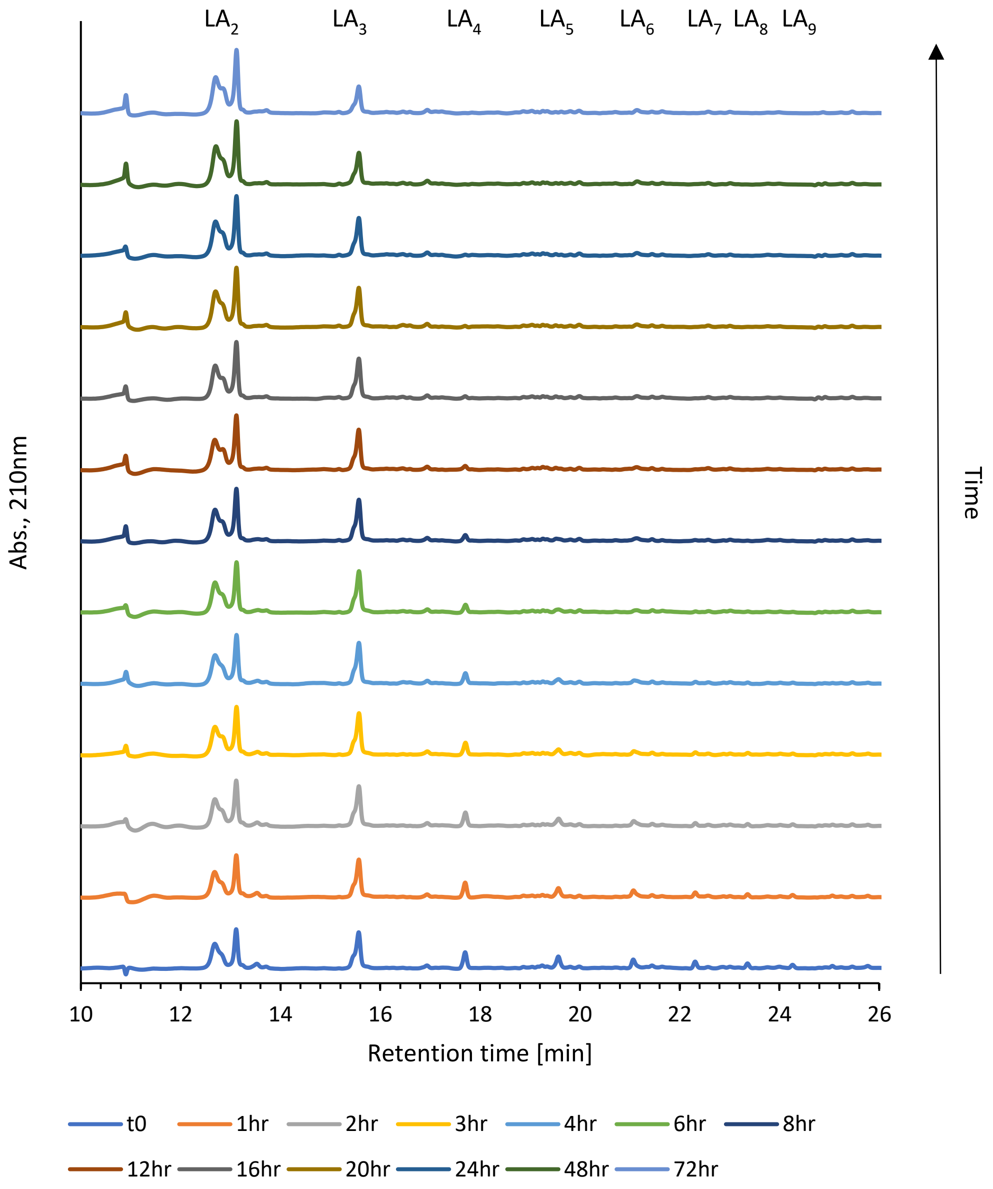

Figure S214. HPLC chromatograms obtained for LA homoesters of DA:LA reaction product at a 1:4 molar ratio at pH 6.8 and 40°C over the course of the degradation study. The region in the chromatograms that corresponds to the elution of LA homoesters is shown. As indicated by the chromatograms, the longer LA oligomers (LA5 to LA9) underwent degradation faster and to a greater extent compared to the shorter oligomers (LA2 to LA4). By the end of the incubation period (72 hours), only dimers and trimers were detected.

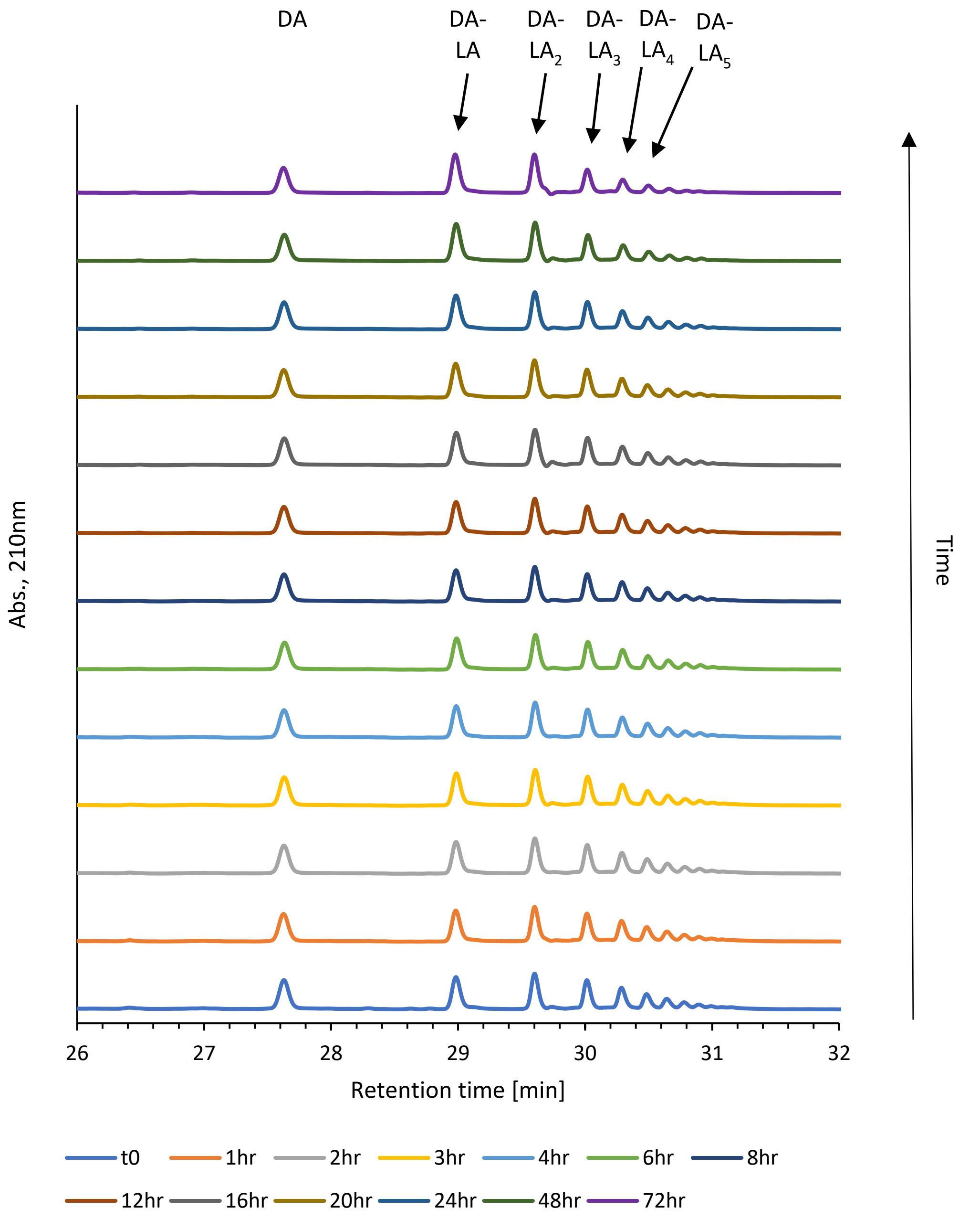

Figure S215. HPLC chromatograms obtained for DA-LA heteroesters of DA:LA reaction product at a 1:4 molar ratio at pH 6.8 and 40°C over the course of the degradation study. The region in the chromatograms that corresponds to the elution of DA-LA heteroesters is shown. As indicated by the chromatograms, during the course of the incubation period most of the products remained and were detected even after 72hr. Only the longest oligomers of more than 7-mers were significantly degraded.

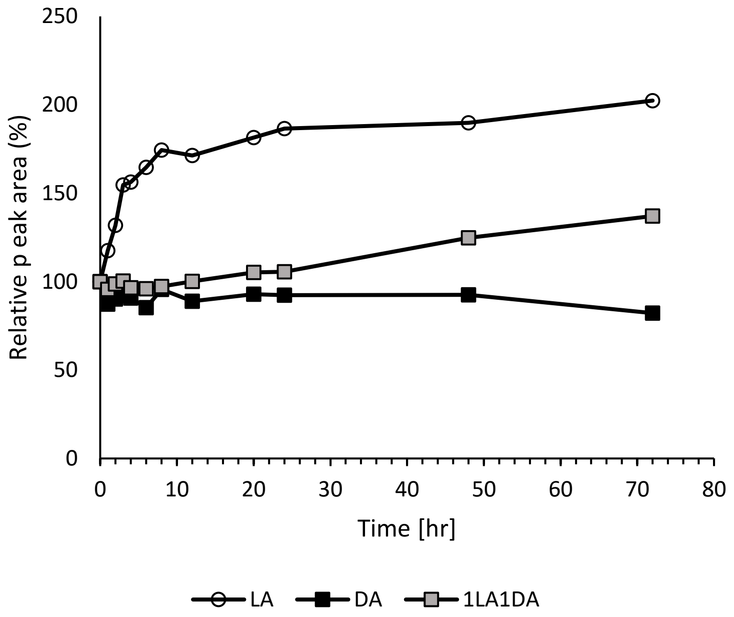

Figure S216. Peak area measured for LA monomer, DA monomer, and the 1DA1LA conjugation prodcut in DA:LA reaction sample at a 1:4 molar ratio over the course of the degradation study. The peak areas of LA, DA, and 1DA1LA conjugation product obtained for DA:LA reaction product at a 1:4 molar ratio were measured over the course of the hydrolysis at pH 6.8 and 40 °C. As indicated in the figure, LA accumulated over the incubation period, exhibiting an increase of about 1.5-fold due to hydrolysis of the different oligomer products. DA concentration remained approximately constant for most of the incubation period, and towards the last time point at 72 hr it decreased, probably due to volatilization. The concentration of 1DA1LA reaction product gradually increased during the incubation, reaching approximately 130% of its initial concentration. This increase in concentration is in accordance with the hydrolysis of longer DA-conjugated oligoesters.

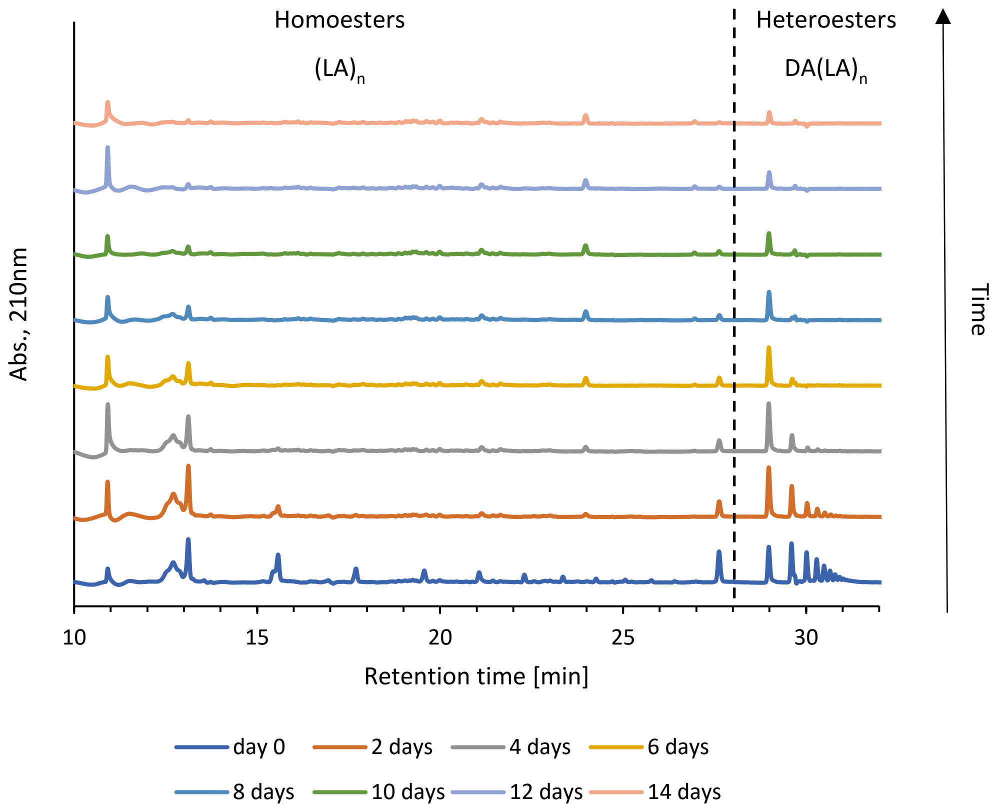

Figure S217. HPLC chromatograms obtained for DA:LA reaction product at a 1:4 molar ratio following incubation at 60°C and rehydration in citrate buffer at pH 5.5.

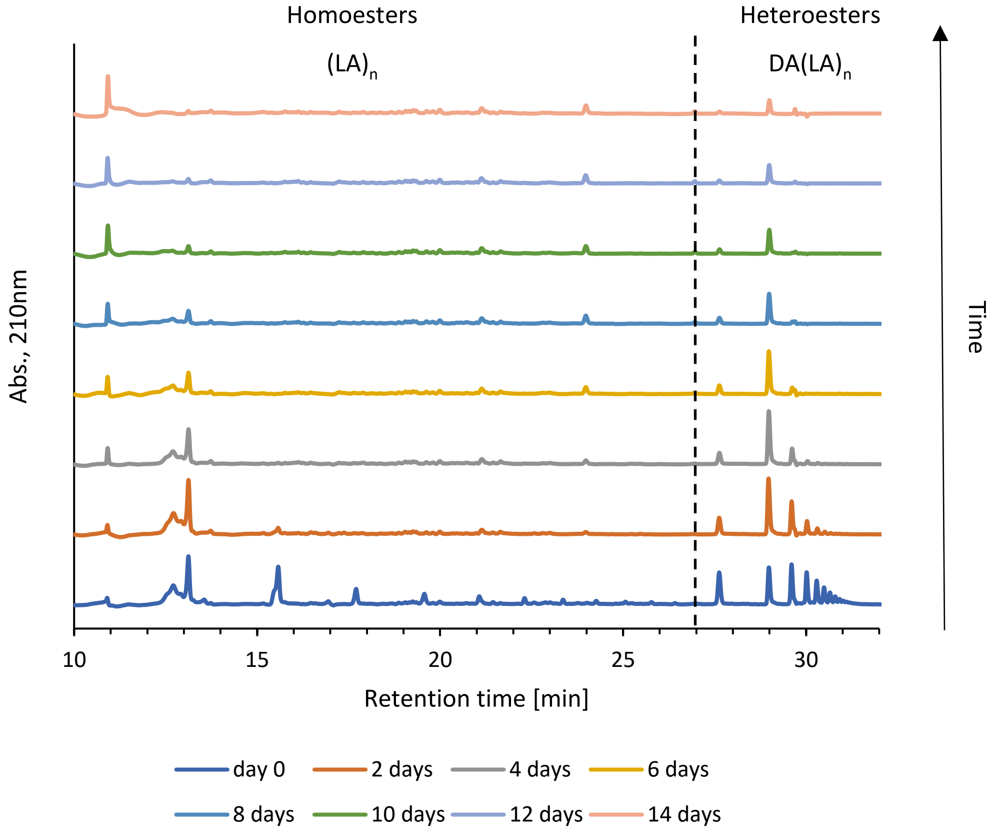

Figure S218. HPLC chromatograms obtained for DA:LA reaction product at a 1:4 molar ratio following incubation at 60°C and rehydration in phosphate buffer at pH 6.8.

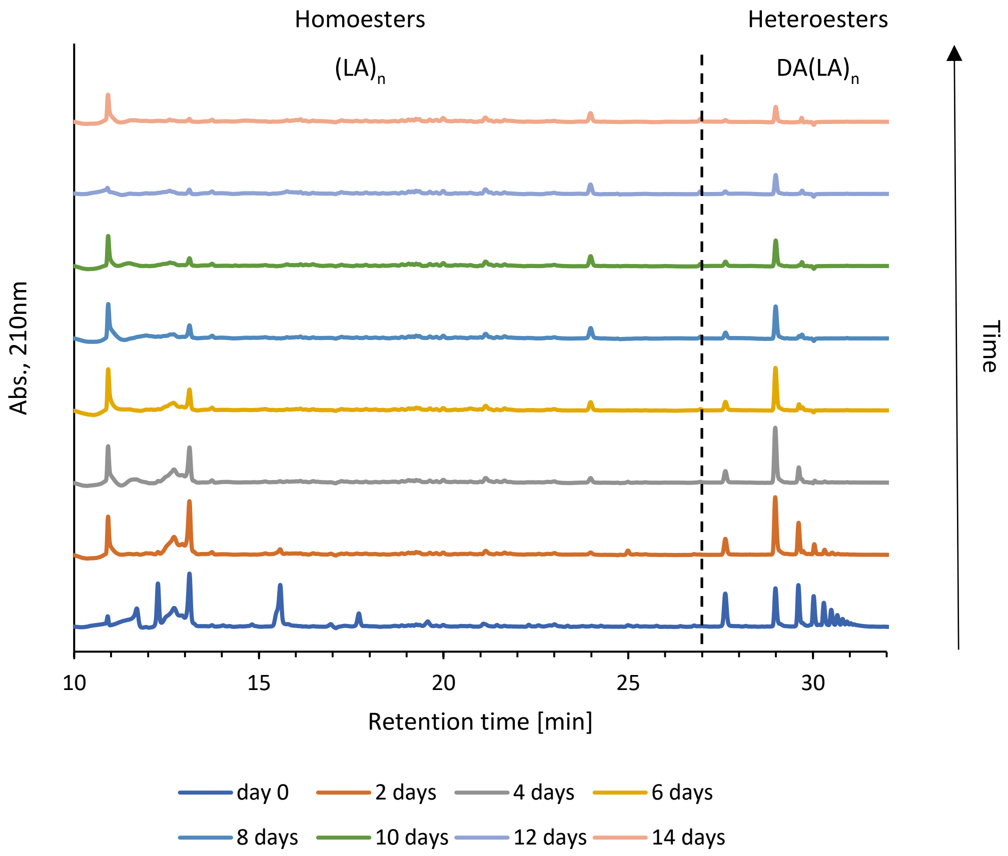

Figure S219. HPLC chromatograms obtained for DA:LA reaction product at a 1:4 molar ratio following incubation at 60°C and rehydration in tris buffer at pH 8.0.

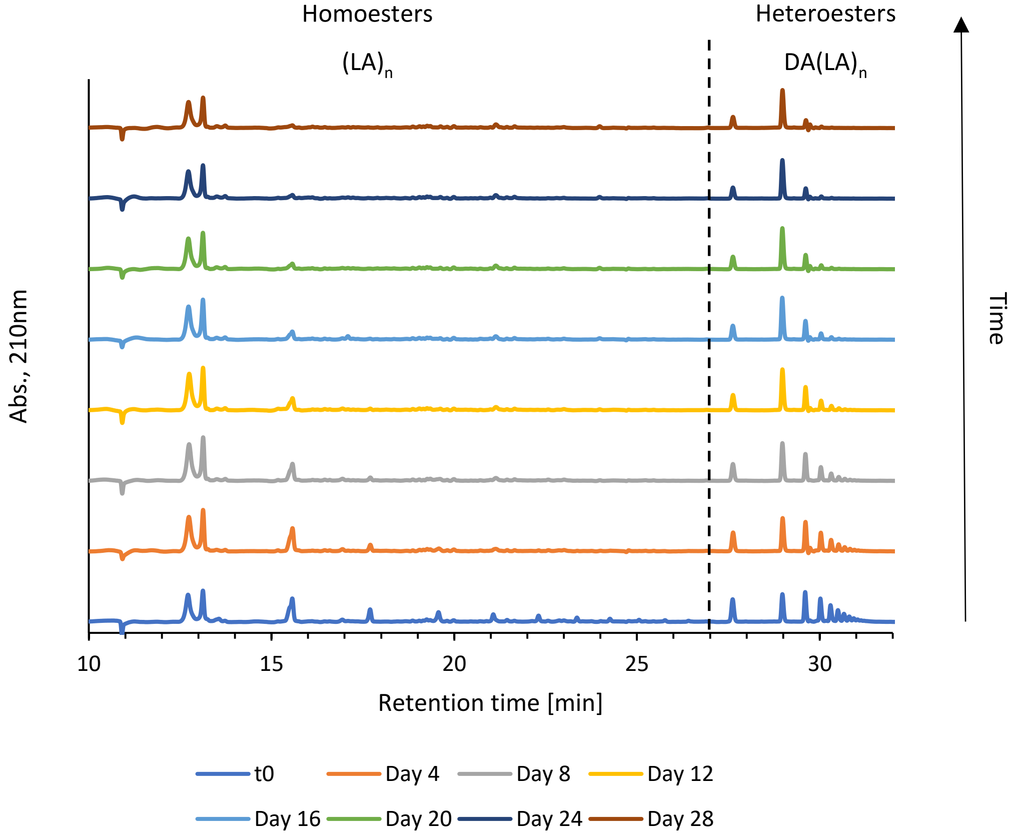

Figure S220. HPLC chromatograms obtained for DA:LA reaction product at a 1:4 molar ratio following incubation at 40°C and rehydration in citrate buffer at pH 5.5.

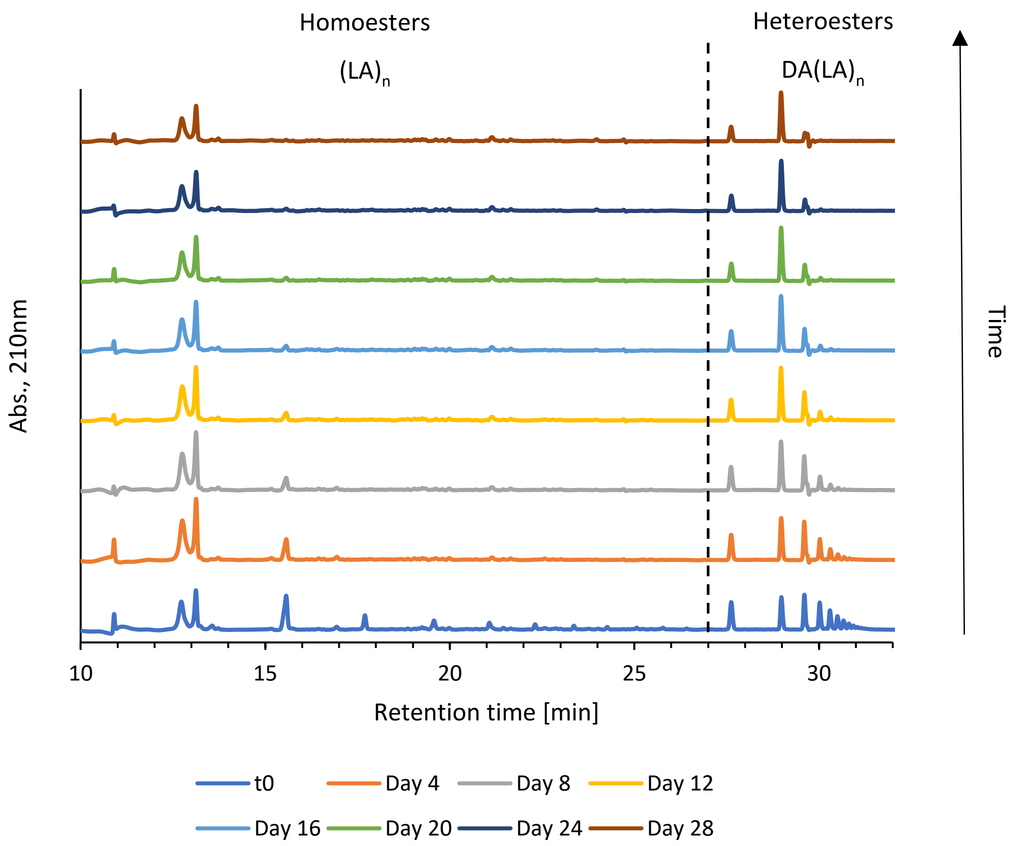

Figure S221. HPLC chromatograms obtained for DA:LA reaction product at 1:4 molar ratio following incubation at 40°C and rehydration in phosphate buffer at pH 6.8.

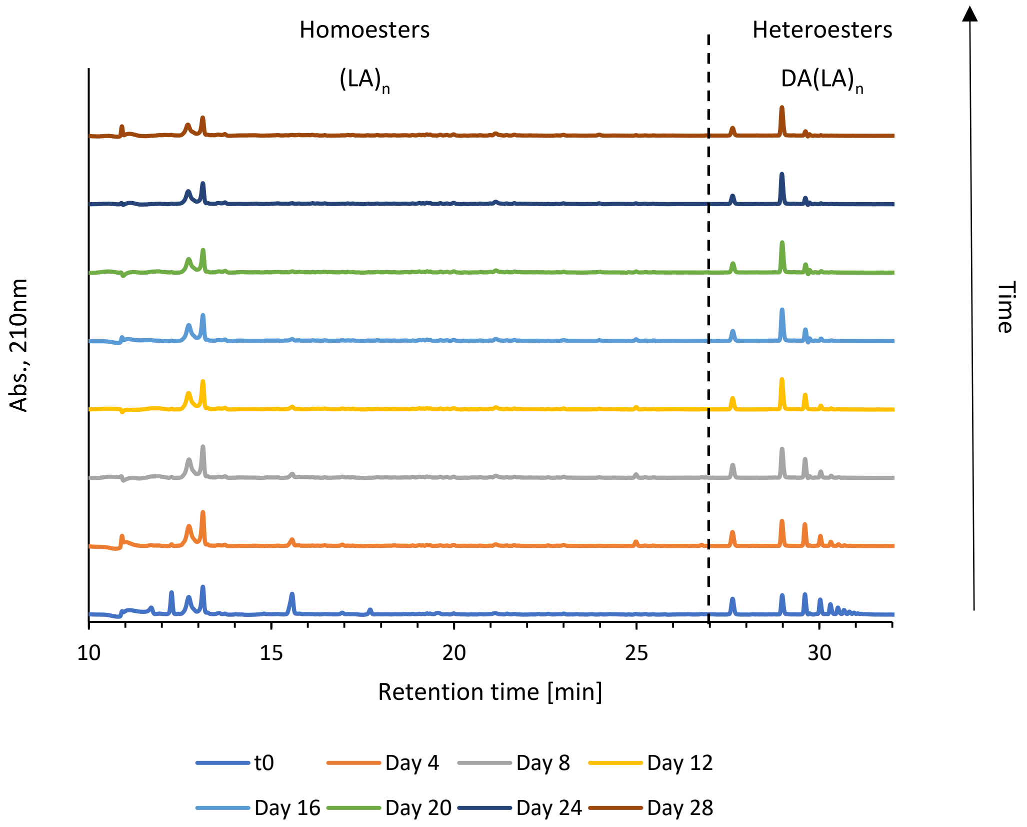

Figure S222. HPLC chromatograms obtained for DA:LA reaction product at 1:4 molar ratio following incubation at 40°C and rehydration in tris buffer at pH 8.0.

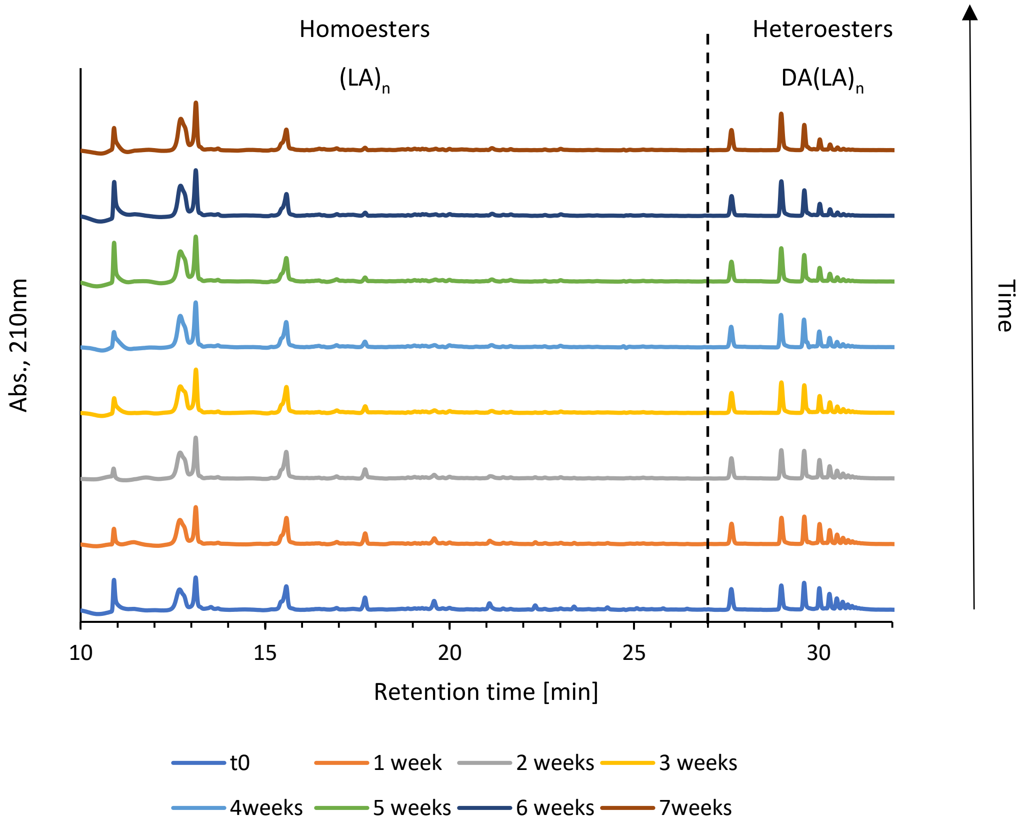

Figure S223. HPLC chromatograms obtained for DA:LA reaction product at 1:4 molar ratio following incubation at room temperature and rehydration in citrate buffer at pH 5.5.

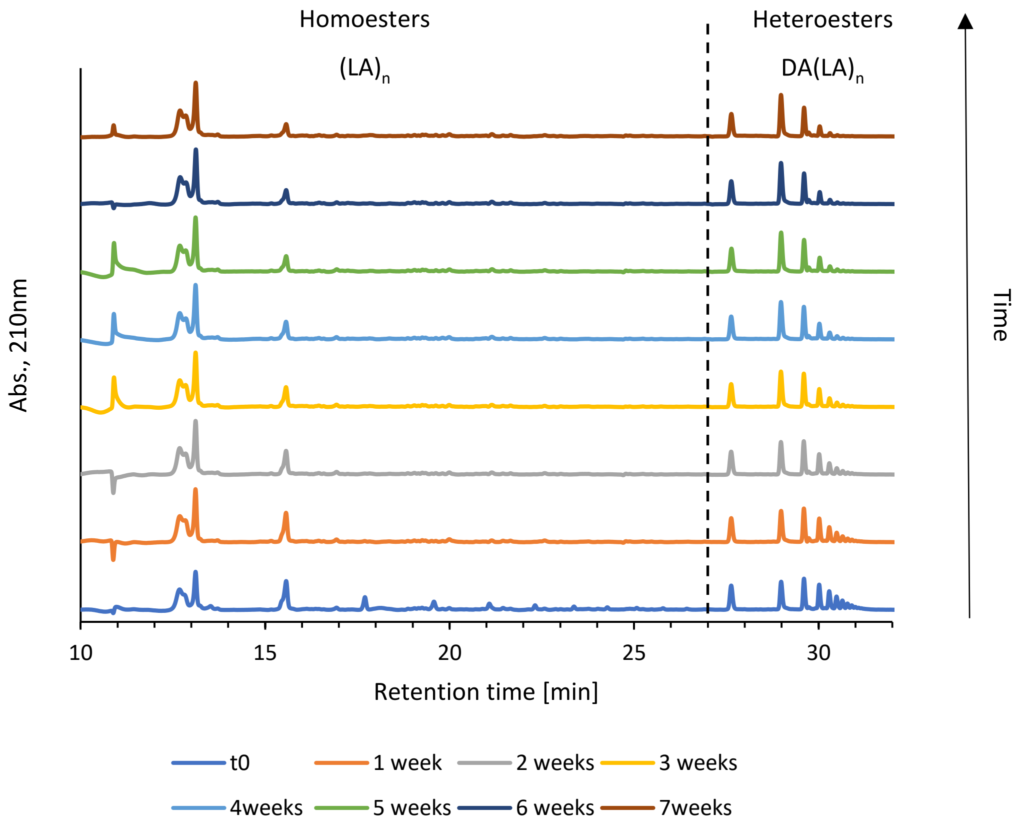

Figure S224. HPLC chromatograms obtained for DA:LA reaction product at 1:4 molar ratio following incubation at room temperature and rehydration in phosphate buffer at pH 6.8.

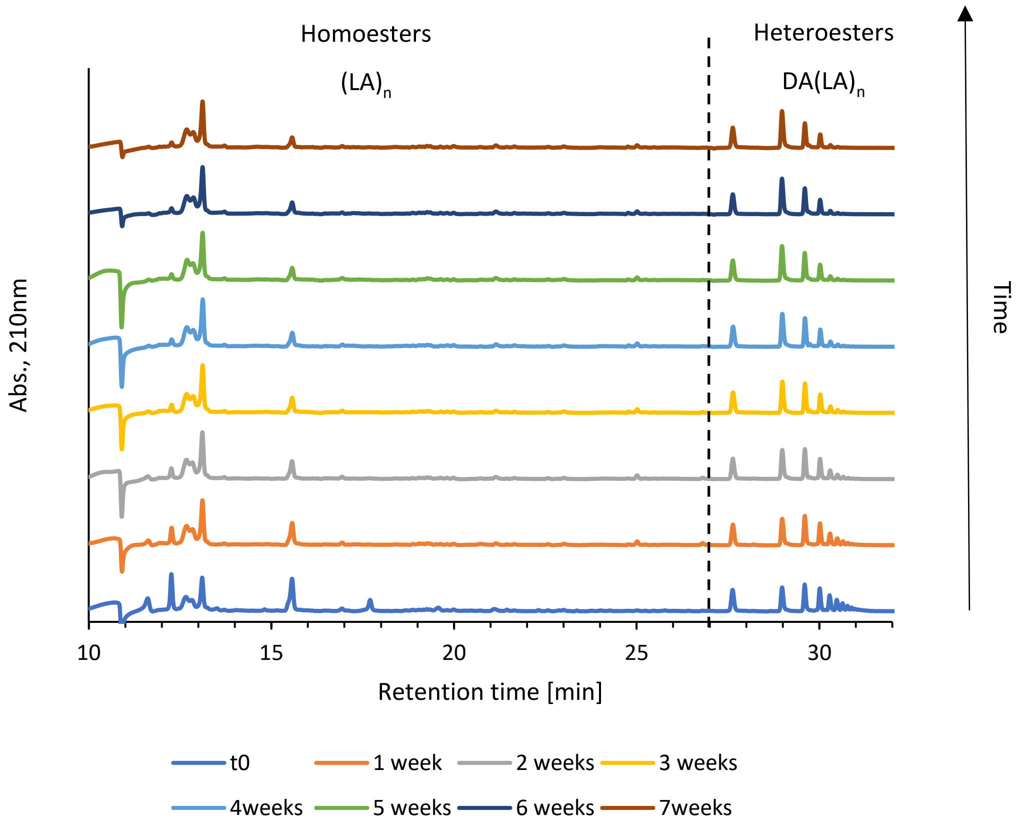

Figure S225. HPLC chromatograms obtained for DA:LA reaction product at 1:4 molar ratio following incubation at room temperature and rehydration in tris buffer at pH 8.0.

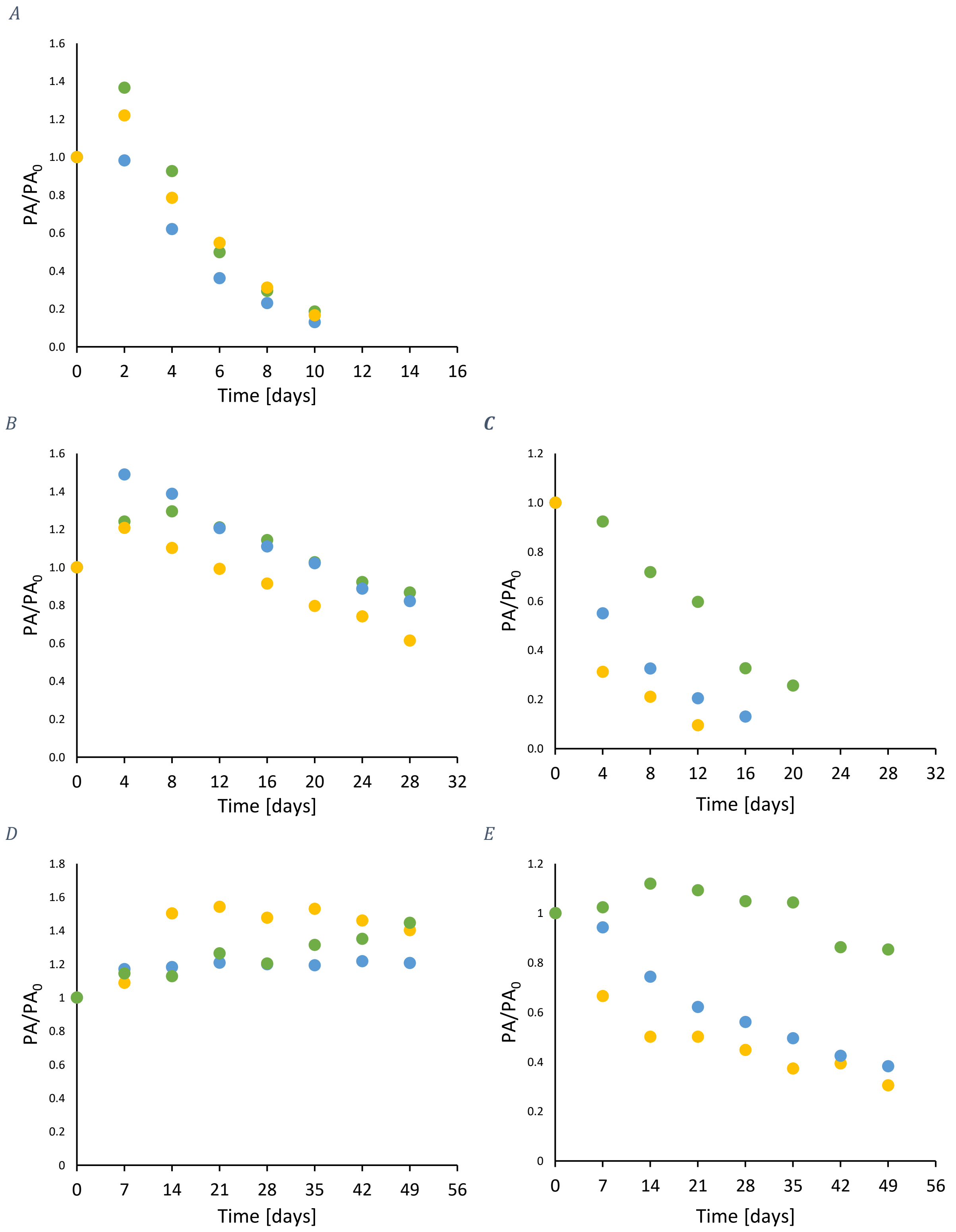

Figure S226. Relative peak area of LA dimer and trimer as a function of incubation period, pH, and temperature. Relative peak area of LA dimers obtained at 60°C and pH levels of 5.5 (green), 6.8 (blue), and 8.0 (yellow) (A). Relative peak area of LA dimers obtained at 40°C and pH levels of 5.5 (green), 6.8 (blue), and 8.0 (yellow) (B). Relative peak area of LA trimers obtained at 40°C and pH levels of 5.5 (green), 6.8 (blue), and 8.0 (yellow) (C). Relative peak area of LA dimers obtained at room temperature (RT) and pH levels of 5.5 (green), 6.8 (blue), and 8.0 (yellow) (D). Relative peak area of LA trimers obtained at room temperature and pH levels of 5.5 (green), 6.8 (blue), and 8.0 (yellow) (E). The effect of pH on the degradation rate of LA oligomers is pronounced at 40°C and at RT.

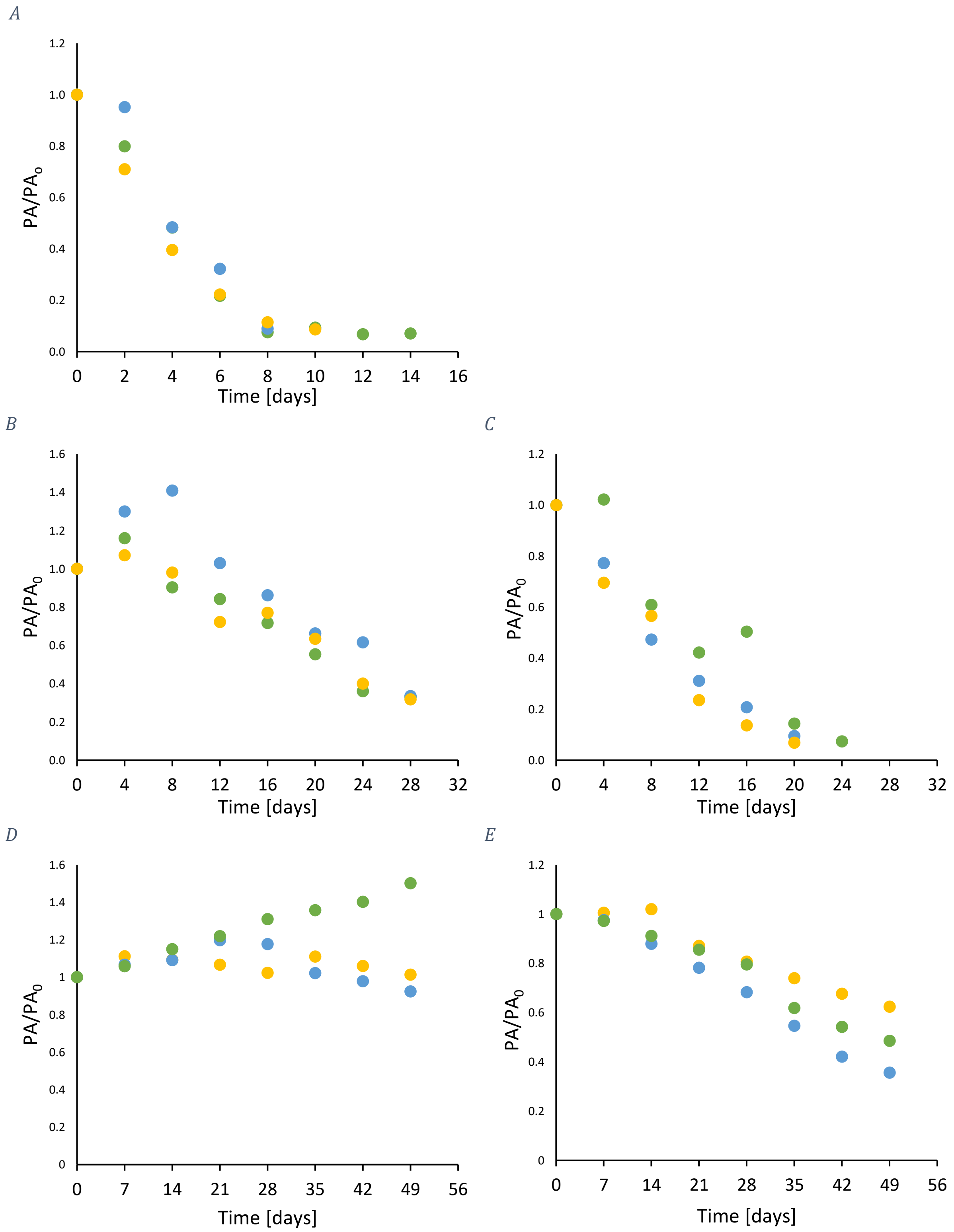

Figure S227. Relative peak area of DA-LA conjugates as a function of storage period, pH and temperature. Relative peak area of DA-LA2 obtained at 60°C and pH levels 5.5 (green), 6.8 (blue) and 8.0 (yellow) (A). Relative peak area of DA-LA2 obtained at 40°C and pH levels 5.5 (green), 6.8 (blue) and 8.0 (yellow) (B). Relative peak area of DA-LA3 obtained at 40°C and pH levels 5.5 (green), 6.8 (blue) and 8.0 (yellow) (C). Relative peak area of DA-LA2 obtained at room temperature (RT) and pH levels 5.5 (green), 6.8 (blue) and 8.0 (yellow) (D). Relative peak area of DA-LA3 obtained at room temperature and pH levels 5.5 (green), 6.8 (blue) and 8.0 (yellow) (E). The effect of pH on the degradation rate of DA-LA conjugates is relatively negligible, except for RT, at which degradation was only slightly faster at pH 6.8 and 8.0 in the case of DA-LA2.

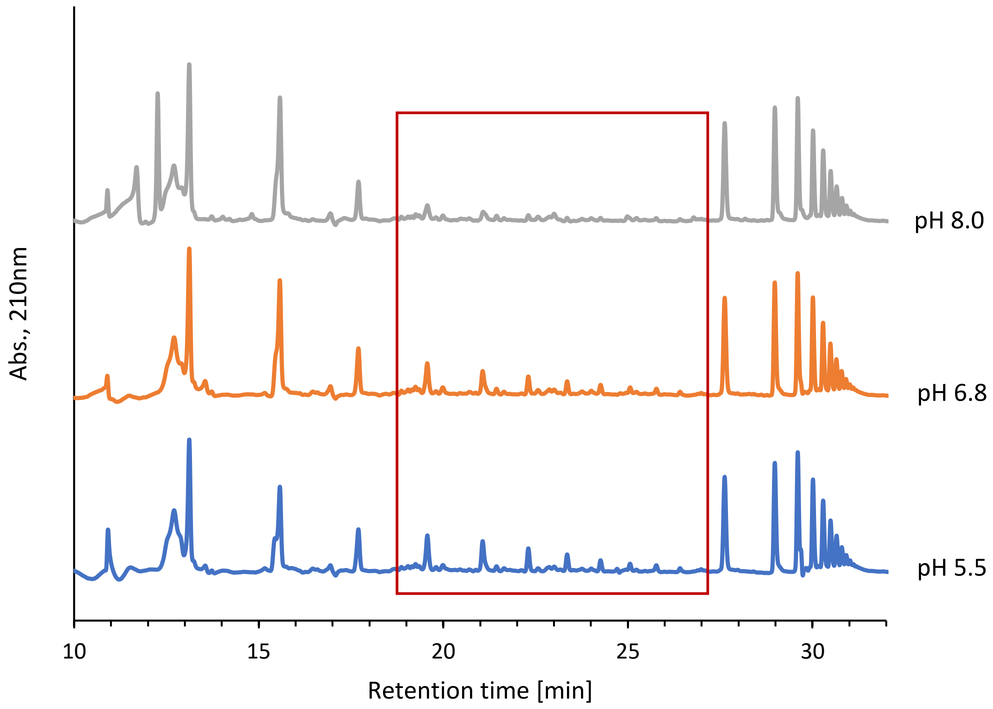

Figure S228. HPLC chromatograms obtained for DA:LA reaction product at a 1:4 molar ratio at t0 prior to incubation. Rection products were rehydrated in either citrate buffer, phosphate buffer, or tris buffer, at pH 5.5, 6.8, or 8.0, respectively. Already at t0, the peaks corresponding to LA oligomers are significantly reduced at pH 8.0 compared to lower pH levels.

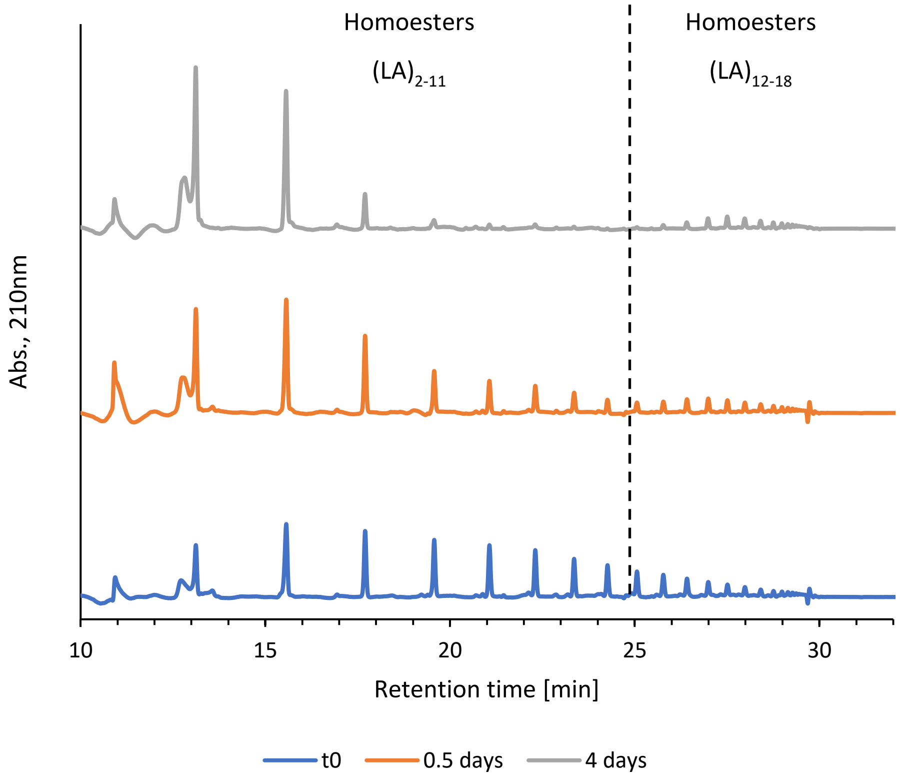

Figure S229. HPLC chromatograms obtained for 100mM LA reaction products following incubation at room temperature (RT). Reaction product was rehydrated in phosphate buffer (50 mM) at pH 6.8 and kept at RT. The initial amount of LA was 400 µmol, corresponding to LA monomer introduced at DA:LA 1:2 molar ratio. As indicated by the chromatograms, already after 4 days at RT, most of the medium-chain oligomers were hydrolyzed while the longer oligomers remained in the system.

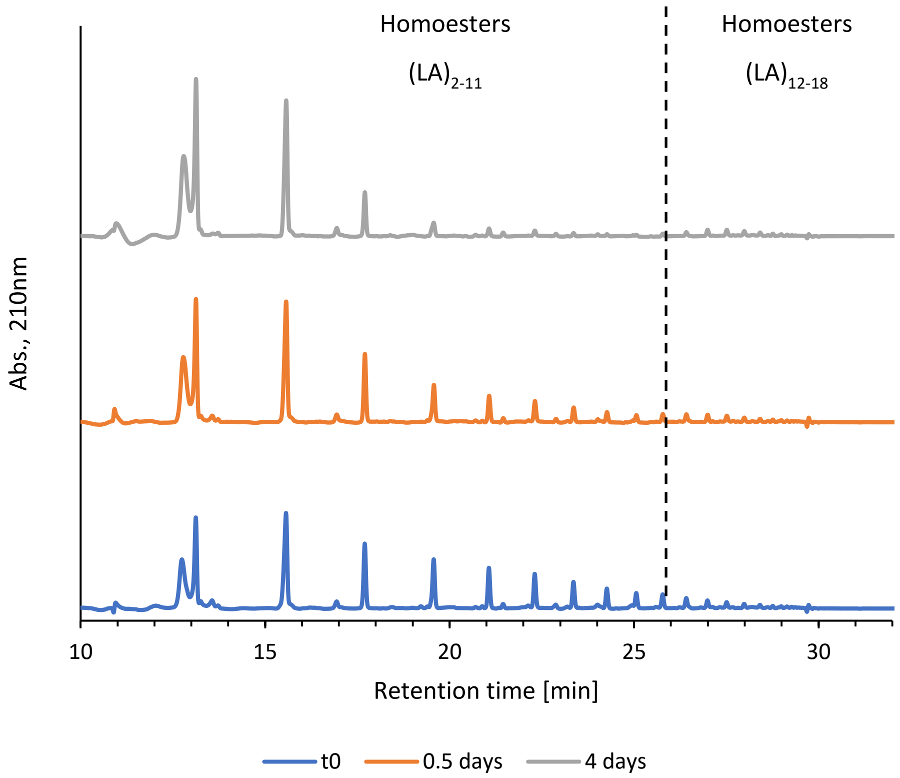

Figure S230. HPLC chromatograms obtained for 200mM LA reaction products following incubation at room temperature (RT). Reaction product was rehydrated in phosphate buffer (50mM) at pH 6.8 and kept at RT. The initial amount of LA was 800µmol, corresponding to LA amount introduced at DA:LA 1:4 molar ratio. As indicated by the chromatograms, already after 4 days at RT, most of the medium chain oligomers were hydrolyzed while the longer oligomers remained at the system.

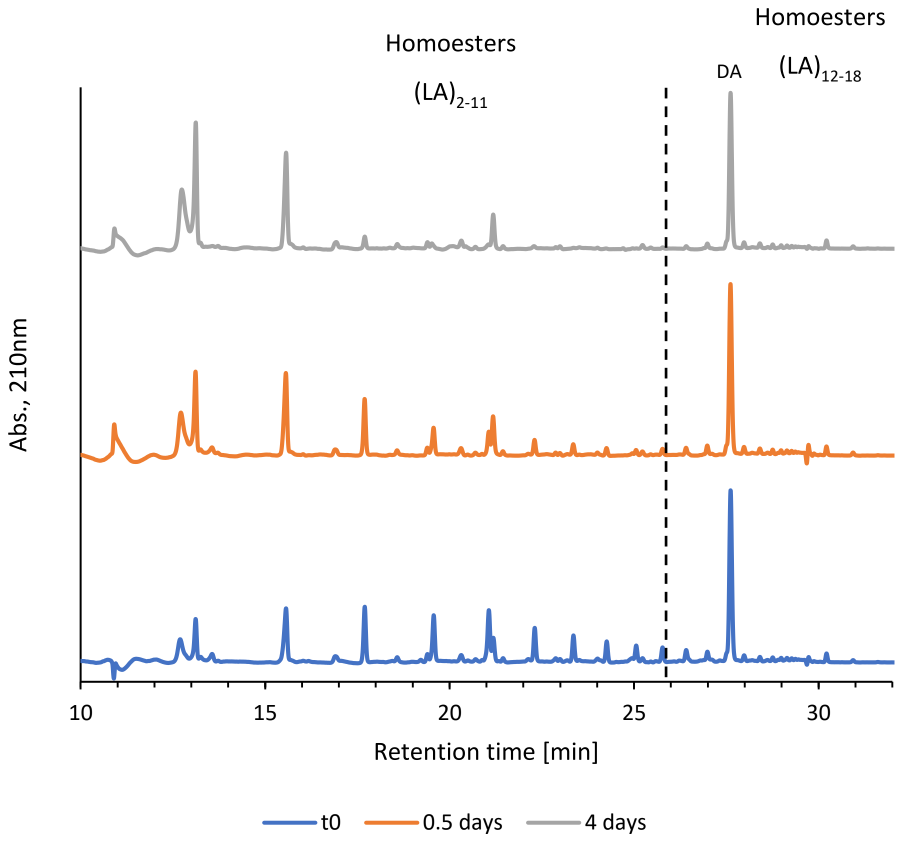

Figure S231. HPLC chromatograms obtained for 100mM LA reaction products in the presence of DA monomer following incubation at room temperature (RT). LA reaction product was rehydrated in DA monomers stock (50mM) and phosphate buffer (50mM) at pH 6.8 and kept at RT. The initial amount of LA was 400μmol, corresponding to LA amount introduced at DA:LA 1:2 molar ratio. As indicated by the chromatograms, already after 4 days at RT, most of the medium chain oligomers were hydrolyzed while the longer oligomers remained at the system.

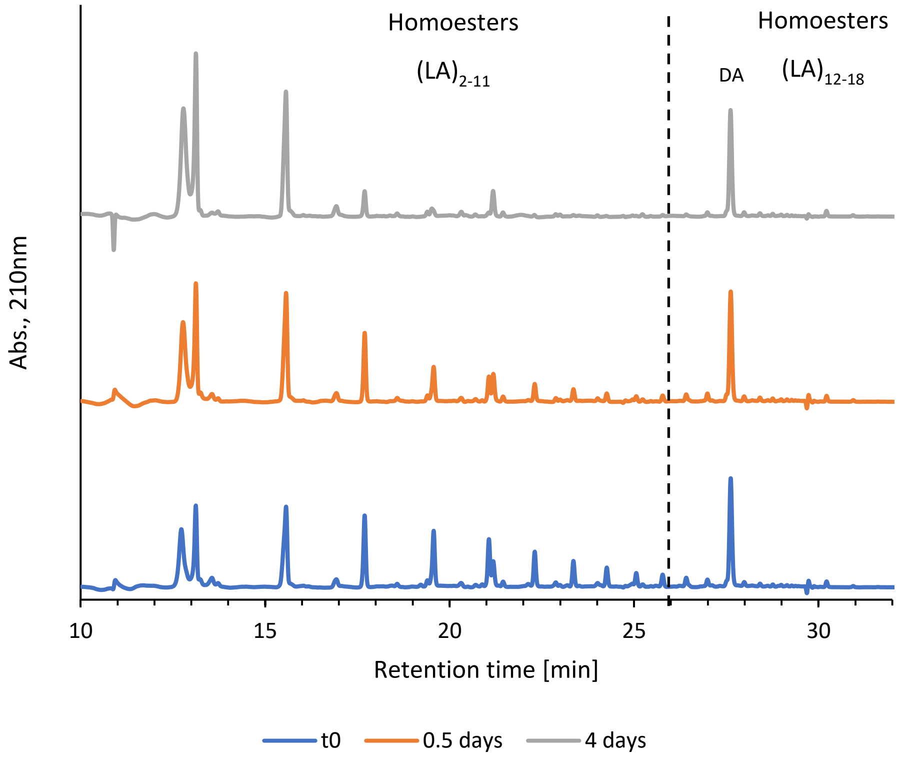

Figure S232. HPLC chromatograms obtained for 200mM LA reaction products in the presence of DA monomer following incubation at room temperature (RT). LA reaction product was rehydrated in DA monomers stock (50mM) and phosphate buffer (50mM) at pH 6.8 and kept at RT. The initial amount of LA was 800μmol, corresponding to LA amount introduced at DA:LA 1:4 molar ratio. As indicated by the chromatograms, already after 4 days at RT, most of the medium chain oligomers were hydrolyzed while the longer oligomers remained at the system.

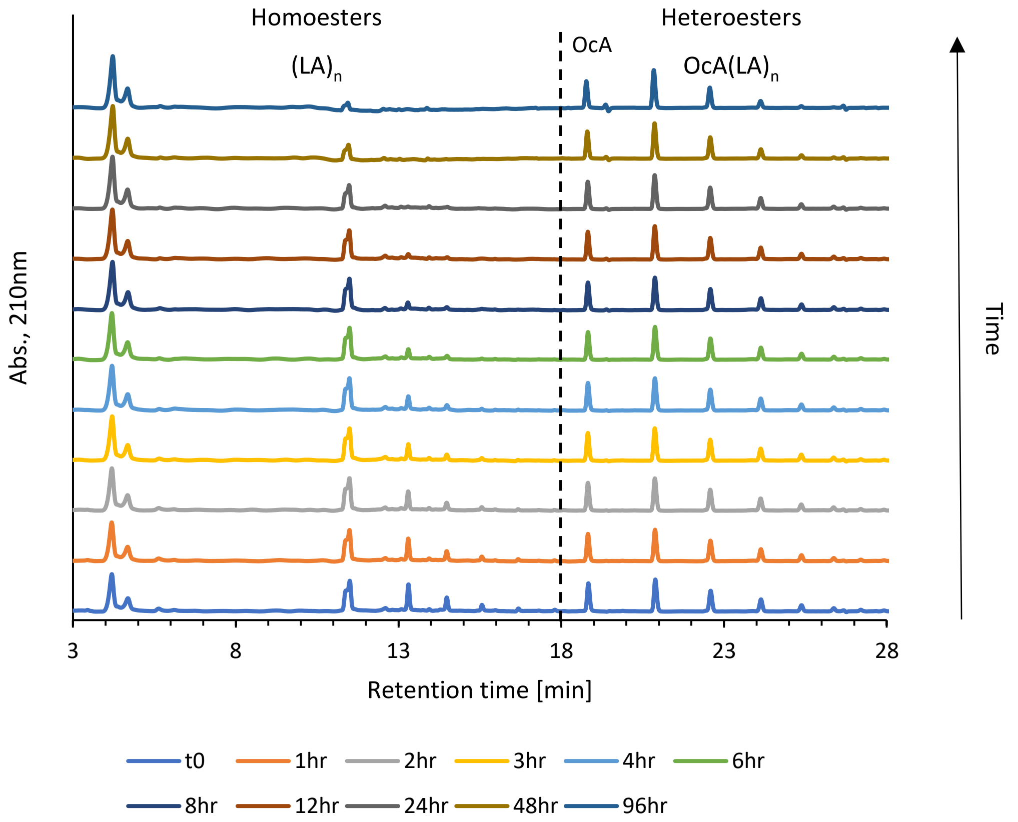

Figure S233. HPLC chromatograms obtained for OcA:LA reaction product at 1:4 molar ratio following incubation at 40°C and rehydration in phosphate buffer at pH 6.6. OcA:LA reaction product at 1:4 molar ratio was rehydrated in phosphate buffer (200mM) at pH 6.6 and stored at 40°C for up to 4 days. OcA and LA concentrations were 150mM and 600mM, respectively, referring to the initial amount prior to the reaction molar ratio. As indicated by the chromatograms, the hydrolysis of LA homoesters was significantly faster compared to the hydrolysis of the corresponding heteroesters.

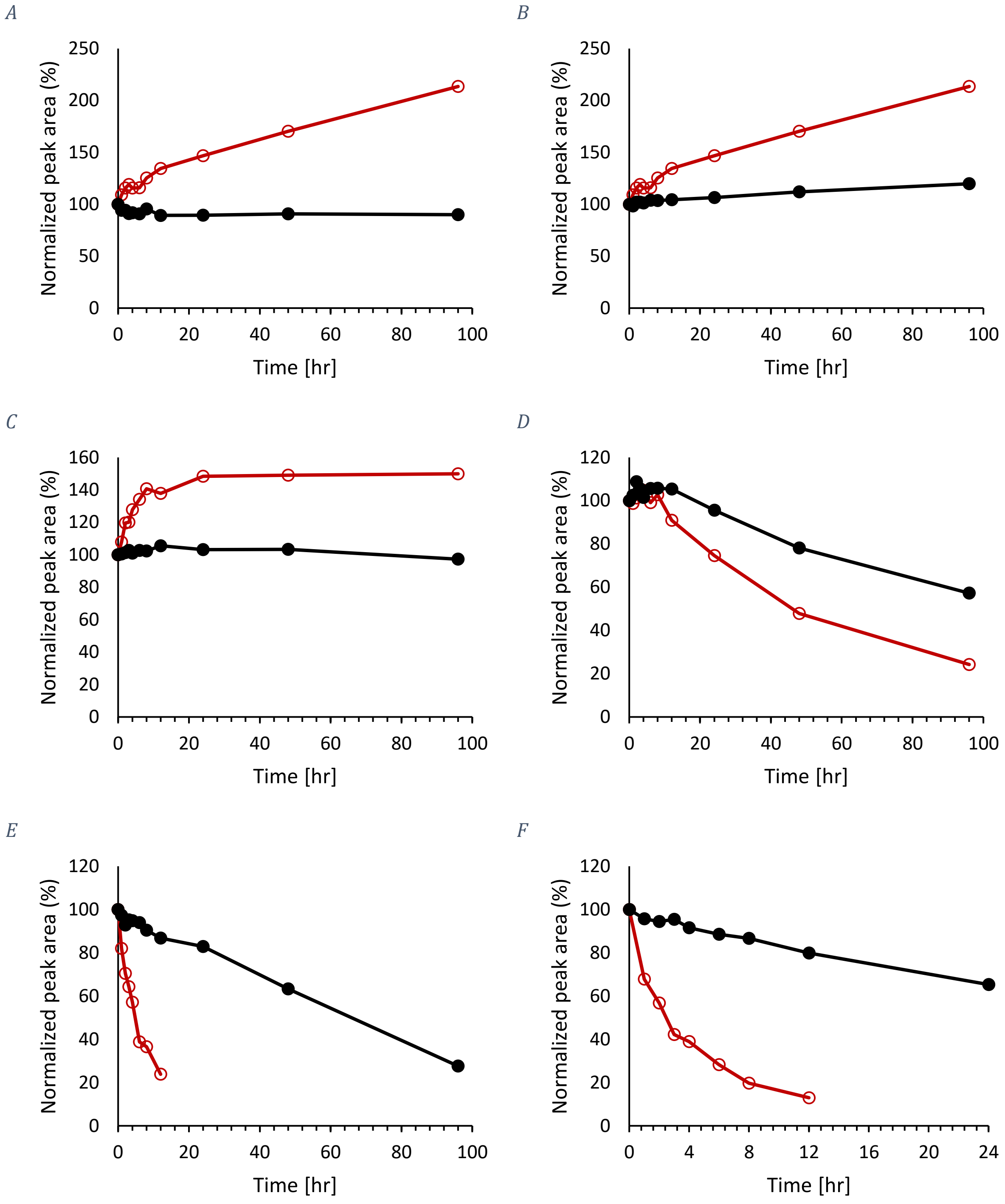

Figure S234. Relative peak area of LA and OcA-LA conjugates as a function of storage period. Relative peak area of OcA (black) and LA (red) monomers obtained at 40°C and pH 6.6. (A). Relative peak area of OcA-LA (black) and LA (red) obtained at 40°C and pH 6.6. (B). Relative peak area of OcA-2LA (black) and 2LA (red) obtained at 40°C and pH 6.6. (C). Relative peak area of OcA-3LA (black) and 3LA (red) obtained at 40°C and pH 6.6. (D). Relative peak area of OcA-4LA (black) and 4LA (red) obtained at 40°C and pH 6.6. (E). Relative peak area of OcA-5LA (black) and 5LA (red) obtained at 40°C and pH 6.6 (F). LA oligomers conjugated to OcA were hydrolyzed to a lesser extent compared to non-conjugated LA oligomers.

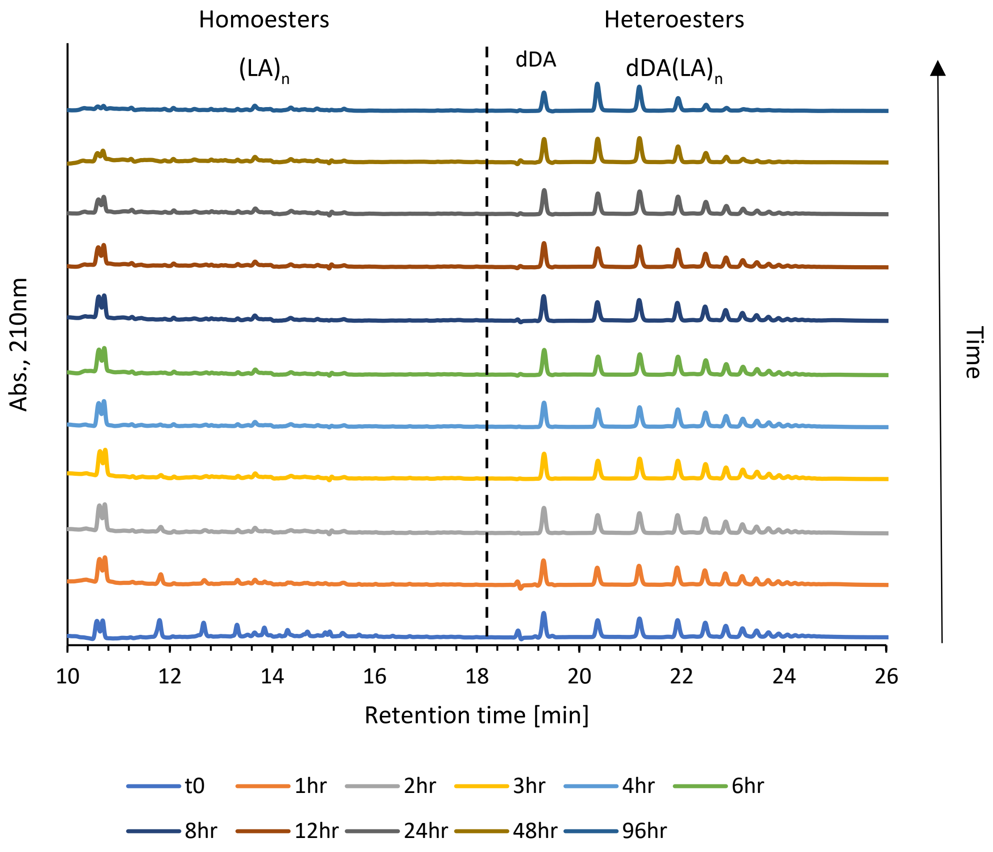

Figure S235. HPLC chromatograms obtained for dDA:LA reaction product at 1:4 molar ratio following incubation at 40°C and rehydration in phosphate buffer at pH 7.4. dDA:LA reaction product at 1:4 molar ratio was rehydrated in phosphate buffer (50mM) at pH 7.4 and stored at 40°C for up to 4 days. dDA and LA concentrations were 20mM and 80mM, respectively, referring to the initial amount prior to the reaction molar ratio. As indicated by the chromatograms, the hydrolysis of LA homoesters was significantly faster compared to the hydrolysis of the corresponding heteroesters.

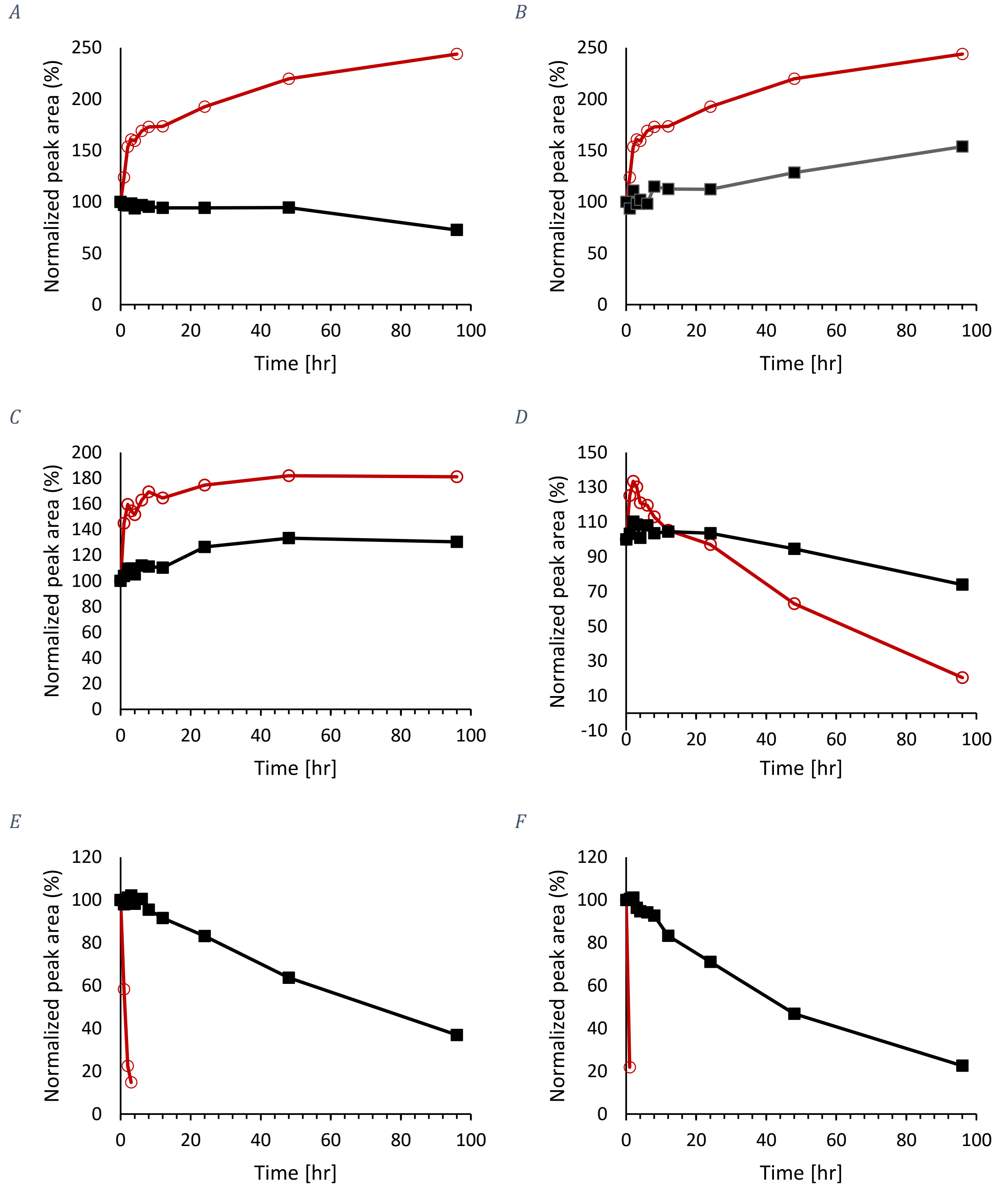

Figure S236. Relative peak area of LA and dDA-LA conjugates as a function of storage period. Relative peak area of dDA (black) and LA (red) monomers obtained at 40°C and pH 7.4. (A). Relative peak area of dDA-LA (black) and LA (red) obtained at 40°C and pH 7.4 (B). Relative peak area of dDA-2LA (black) and 2LA (red) obtained at 40°C and pH 7.4 (C). Relative peak area of dDA-3LA (black) and 3LA (red) obtained at 40°C and pH 7.4 (D). Relative peak area of dDA-4LA (black) and 4LA (red) obtained at 40°C and pH 7.4 (E). Relative peak area of dDA-5LA (black) and 5LA (red) obtained at 40°C and pH 7.4 (F). LA oligomers conjugated to dDA were hydrolyzed to a lesser extent compared to non-conjugated LA oligomers.

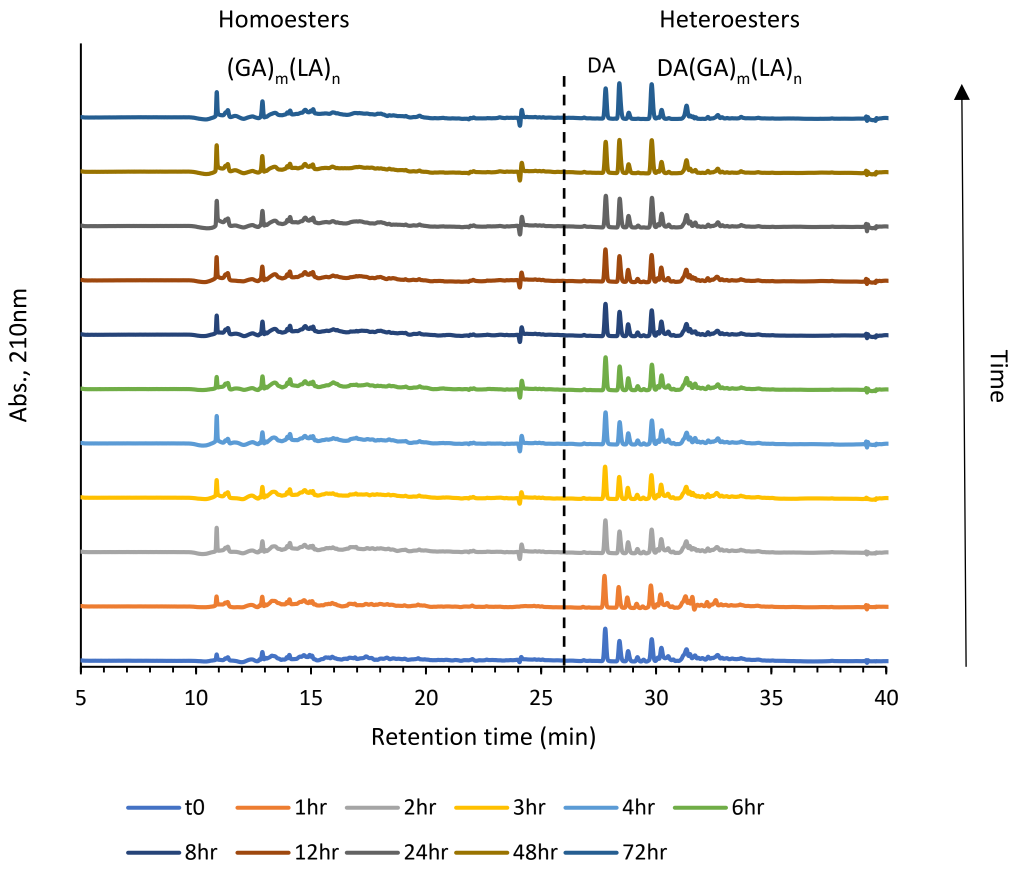

Figure S237. HPLC chromatograms obtained for DA:LA:GA reaction product at 1:2:2 molar ratio following incubation at 40°C and rehydration in phosphate buffer at pH 6.8. DA:LA:GA reaction product at 1:2:2 molar ratio was rehydrated in phosphate buffer (50mM) at pH 6.8 and stored at 40°C for up to 4 days. DA, LA and GA concentrations were 50mM, 100mM and 100mM, respectively, referring to the initial amount prior to the reaction molar ratio.

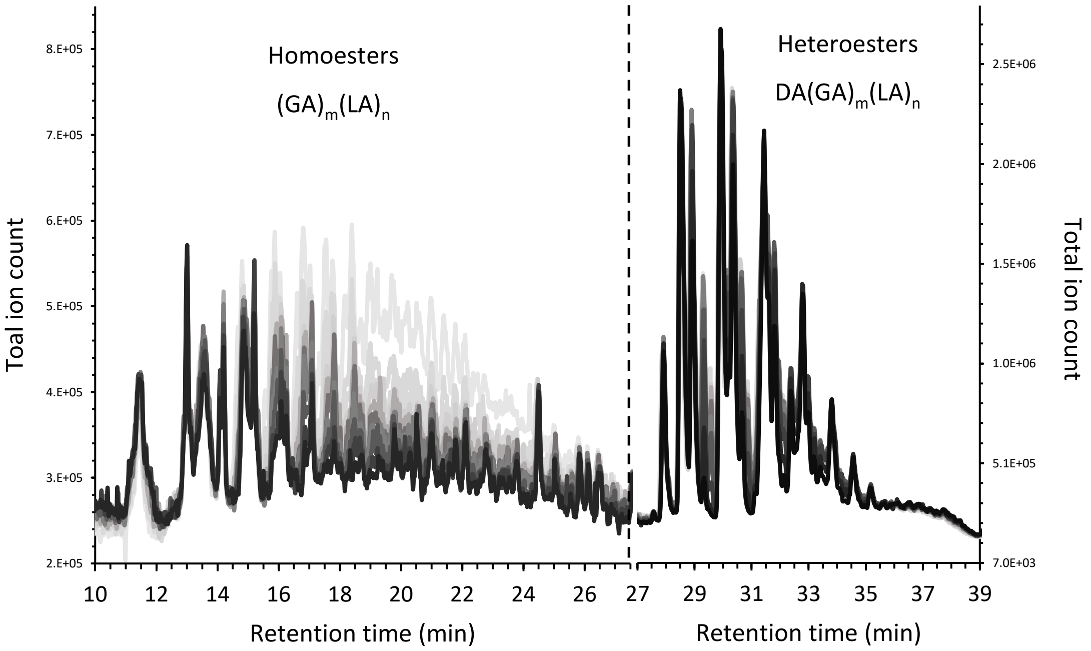

Figure S238. LC-MS chromatograms obtained for DA:LA:GA reaction product at 1:2:2 molar ratio following incubation at 40°C and rehydration in phosphate buffer at pH 6.8. DA:LA:GA reaction product at 1:2:2 molar ratio was rehydrated in phosphate buffer (50mM) at pH 6.8 and stored at 40°C for up to 4 days. DA, LA and GA concentrations were 50mM, 100mM and 100mM, respectively, referring to initial amount prior to the reaction. Time points are represented by color gradient. Light grey line represents t0 and the black line represents 72hr. As indicated in the chromatogram, GA-LA homoesters were hydrolyzed to a greater extent compared to DA-GA-LA heteroesters.

A

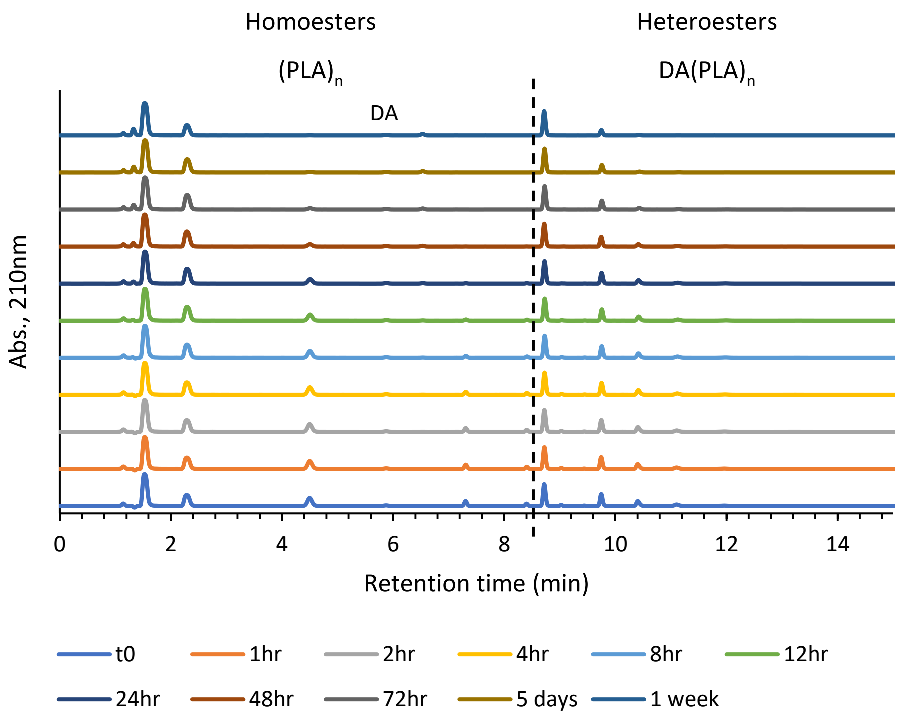

B

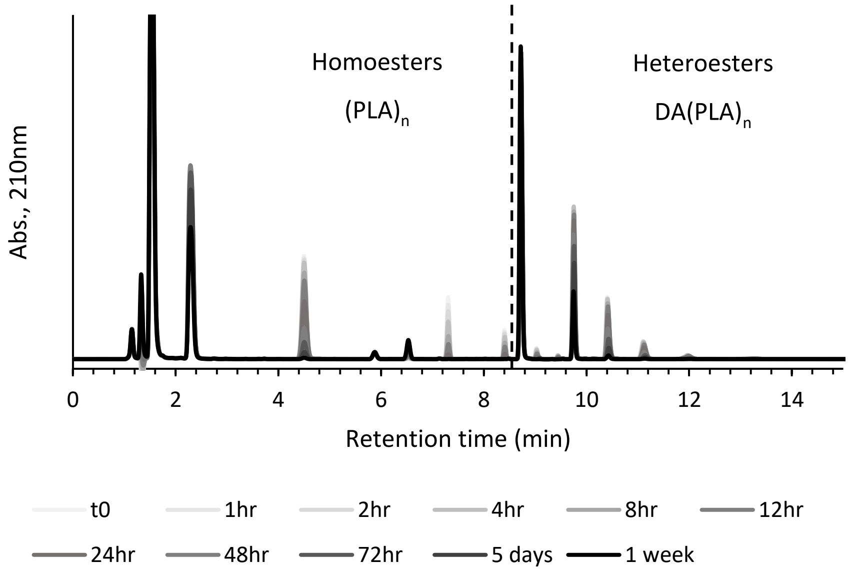

Figure S239. HPLC chromatograms obtained for DA:PLA reaction product at 1:1 molar ratio following incubation at 40°C and rehydration in tris buffer at pH 8.0. DA:PLA reaction product at 1:1 molar ratio was rehydrated in tris buffer (50mM) at pH 8.0 and stored at 40°C for up to 1 week. DA and PLA concentration was 50mM, referring to the initial amount prior to the reaction. Stack representation of the chromatograms obtained for the different time points (A) and overlay representation of the obtained chromatograms (B). Time points are represented by color gradient. The light grey line represents t0 and black line represents 72hr. Loger oligomers were hydrolyzed more slowly when conjugated to DA.

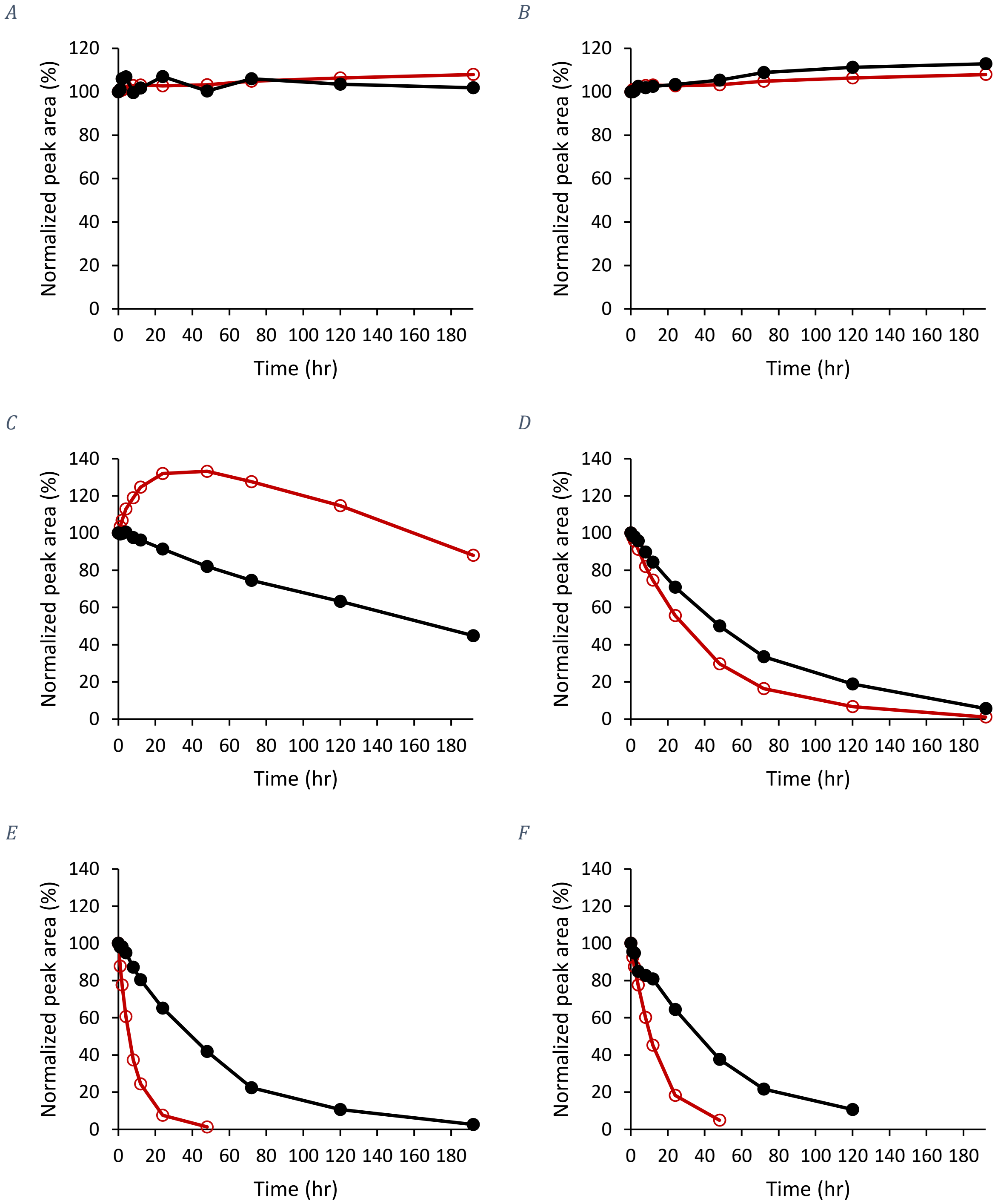

Figure S240. Relative peak area of PLA and DA-PLA conjugates as a function of storage period. Relative peak area of DA (black) and PLA (red) monomers obtained at 40°C and pH 8.0. (A). Relative peak area of DA-PLA (black) and PLA (red) obtained at 40°C and pH 8.0. (B). Relative peak area of DA-2PLA (black) and 2PLA (red) obtained at 40°C and pH 8.0. (C). Relative peak area of DA-3PLA (black) and 3PLA (red) obtained at 40°C and pH 8.0. (D). Relative peak area of DA-4PLA (black) and 4PLA (red) obtained at 40°C and pH 8.0. (E). Relative peak area of DA-5PLA (black) and 5PLA (red) obtained at 40°C and pH 8.0 (F). The longest PLA oligomers conjugated to DA were hydrolyzed to a lesser extent compared to non-conjugated PLA oligomers.

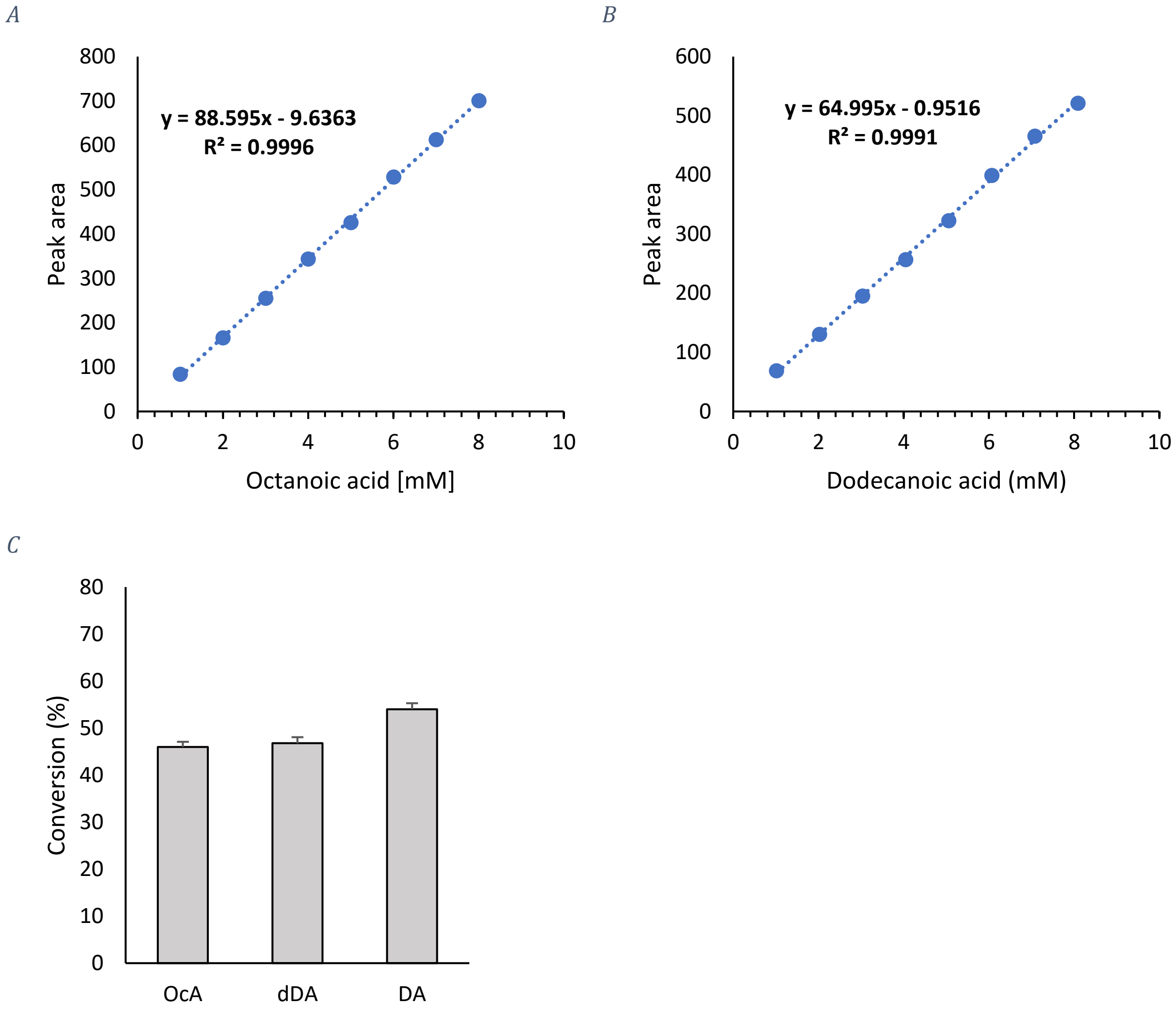

Figure S241. OcA and dDA consumption under dry reaction at 85°C for 7 days as determined by HPLC. A calibration curve was constructed at 210nm for the determination of the conversion of OcA when reacted with LA at 1:4 molar ratio in the favor of LA (A) . Calibration curve was constructed at 210nm for the determination the conversion of dDA when reacted with LA at 1:4 molar ratio in the favor of LA (B). Conversion of different fatty acids when reacted with LA at 1:4 molar ratio in the favor of LA (C). The measured conversion for OcA, dDA and DA was 46%, 47% and 54% respectively. Error bars represent standard deviation of three independent preparations.

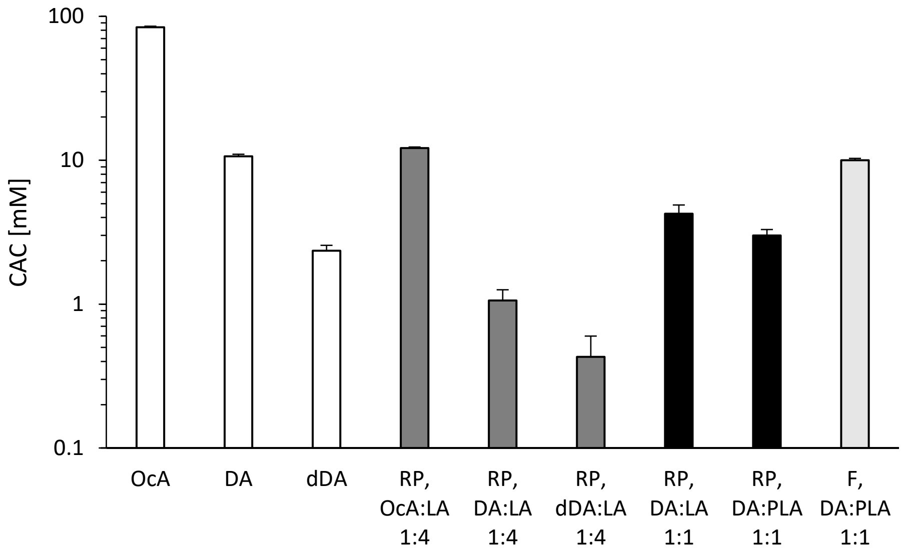

Figure S242. Determination of the critical aggregation concentration of fresh monomers and reaction products of different fatty acids. CAC of fresh OcA, DA and dDA at pH 6.6, 6.8 and 8.0 respectively (white columns), FA:LA reaction products (RP) at 1:4 molar ration (grey columns), DA:HA reaction products at 1:1 molar ratio (black columns) and fresh DA:PLA monomers at 1:1 molar ratio (light grey column). As indicated in the figure, the CAC of all tested fatty acids was significantly reduced by about one order of magnitude in the presence of FA-LA conjugates. DA:PLA reaction product at 1:1 molar ratio exhibited similar effect as the corresponding DA-LA mixture; a reduction of about 3-fold. Error bars represent standard deviation of two independent preparations.

A

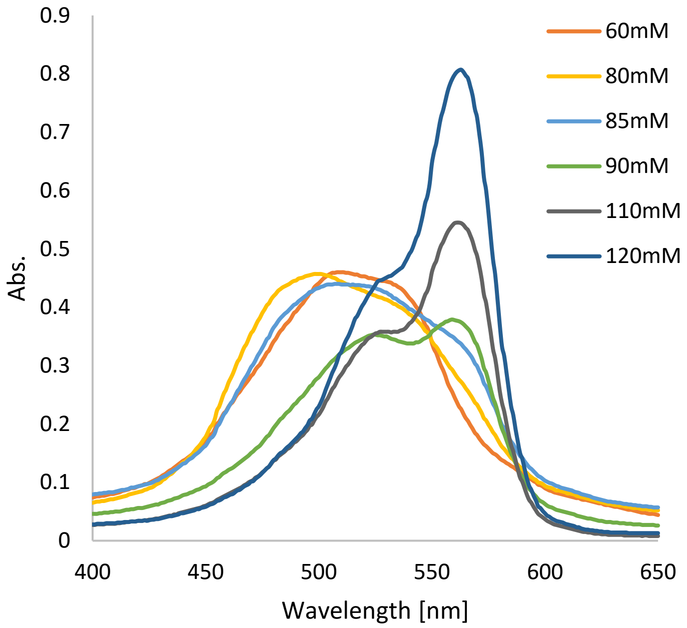

B

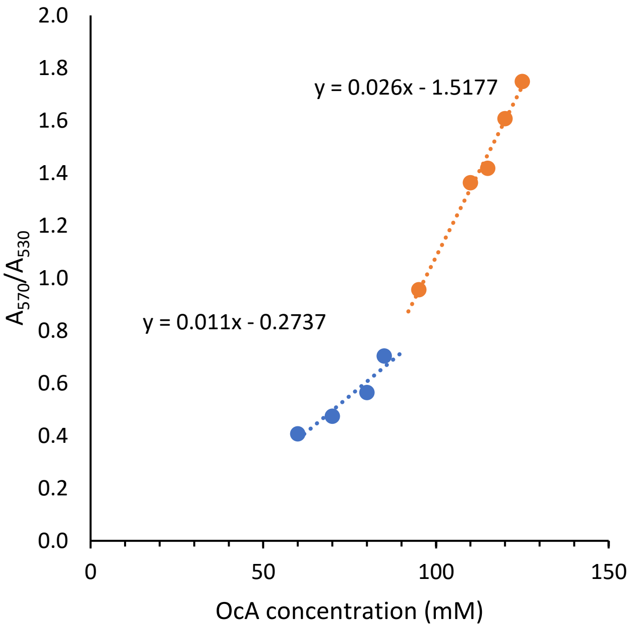

Figure S243. Determination of the critical aggregation concentration of OcA for OcA control sample. Absorption spectra of merocyanine 540 in the presence of increasing concentrations of OcA obtained for OcA control sample at pH 6.6 (A). Absorption ratio at 570 nm and 530 nm as a function of OcA concentration. The intersection between the two fitting lines represents the CAC of OcA – ca. 80 mM (B).

A

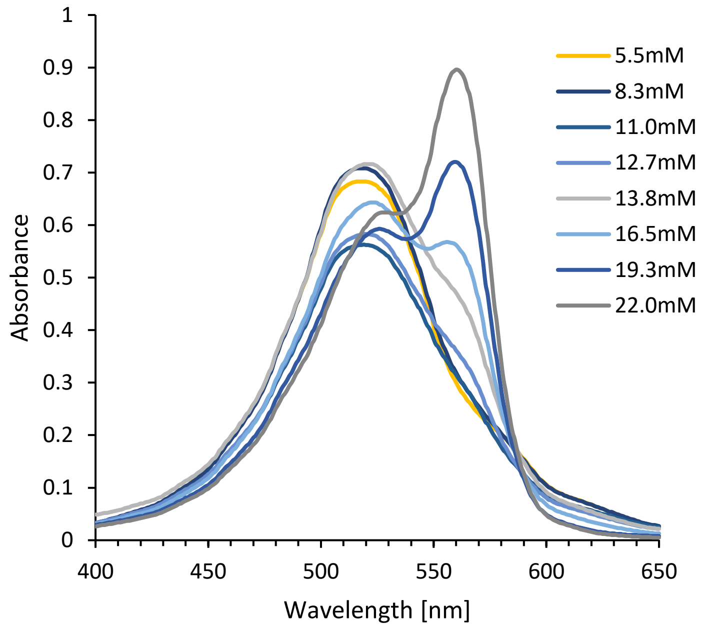

B

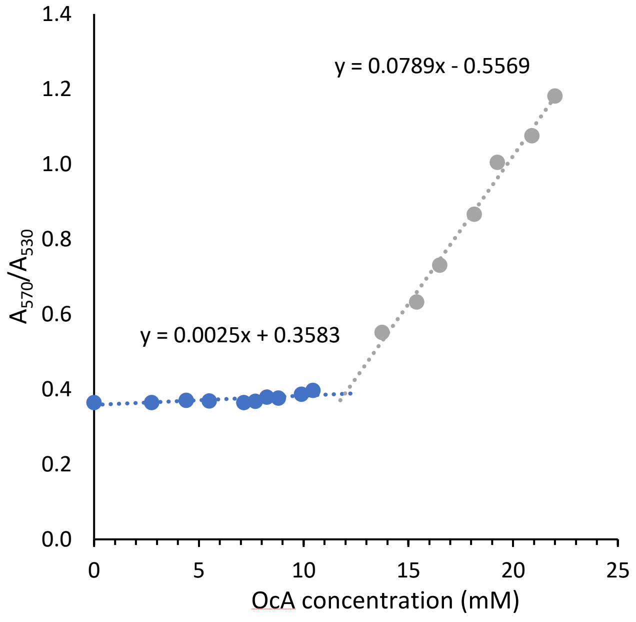

Figure S244. Determination of the critical aggregation concentration of OcA for OcA:LA reaction product at 1:4 molar ratio. Absorption spectra of merocyanine 540 in the presence of increasing concentrations of OcA obtained for the reaction product of OcA:LA at a 1:4 molar ratio at pH 6.6 (A). Absorption ratio at 570 nm and 530 nm as a function of OcA concentration. The intersection between the two fitting lines represents the CAC of OcA – ca 12 mM (B).

A

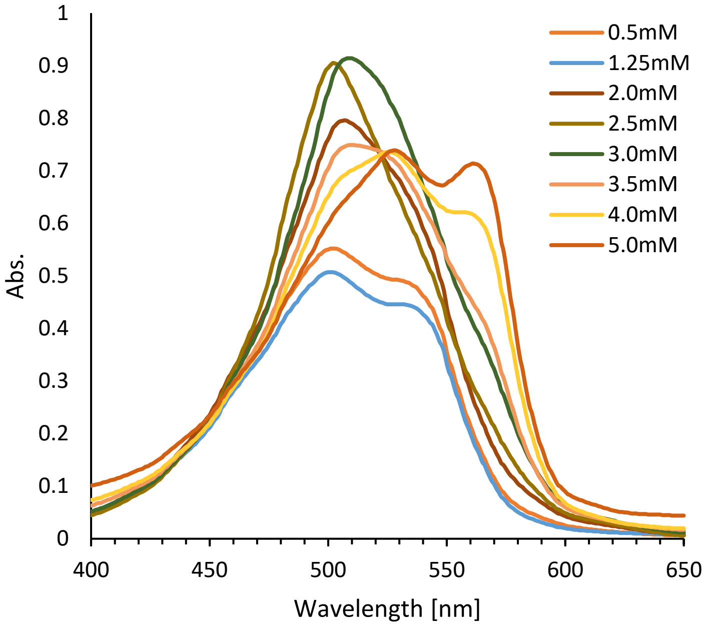

B

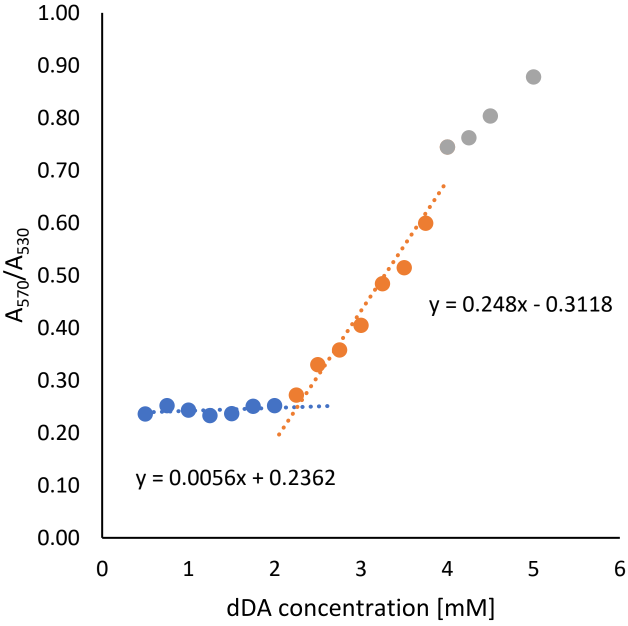

Figure S245. Determination of the critical aggregation concentration of dDA for dDA control sample. Absorption spectra of merocyanine 540 in the presence of increasing concentrations of dDA obtained for dDA control sample at pH 7.4 (A). Absorption ratio at 570 nm and 530 nm as a function of dDA concentration. The intersection between the two fitting lines represents the CAC of dDA – ca. 2.5 mM (B).

A

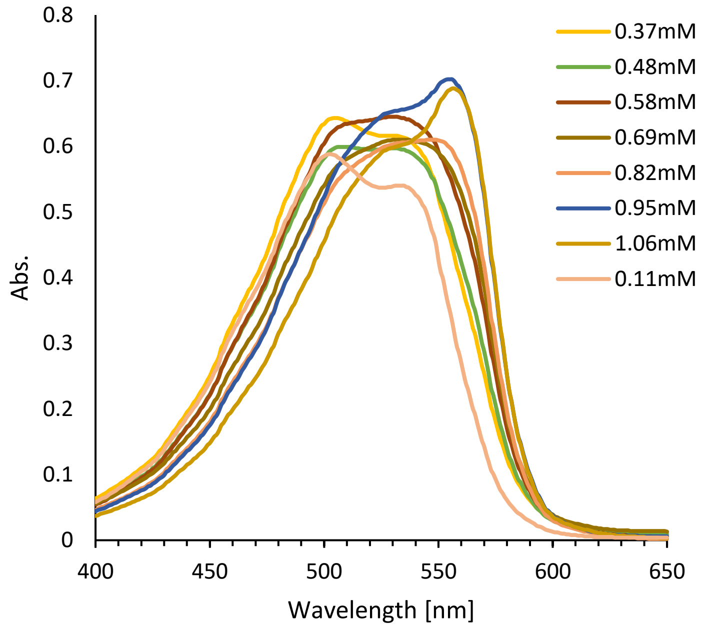

B

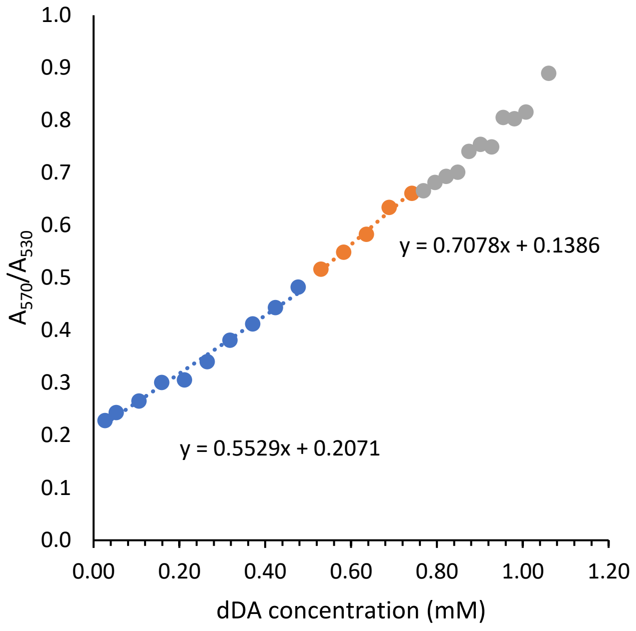

Figure S246. Determination of the critical aggregation concentration of dDA for dDA:LA reaction product at 1:4 molar ratio. Absorption spectra of merocyanine 540 in the presence of increasing concentrations of dDA obtained for the reaction product of dDA:LA at a 1:4 molar ratio at pH 7.4 (A). Absorption ratio at 570 nm and 530 nm as a function of dDA concentration. The intersection between the two fitting lines represents the CAC of dDA – ca 0.4 mM (B).

A

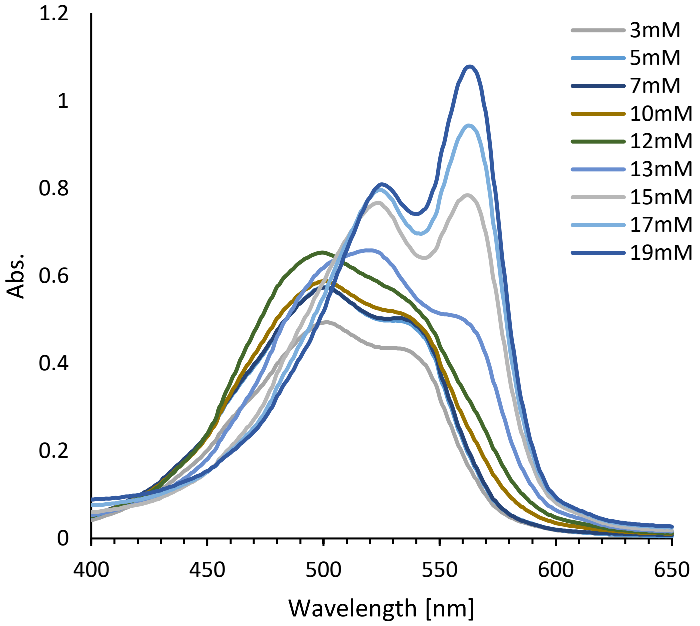

B

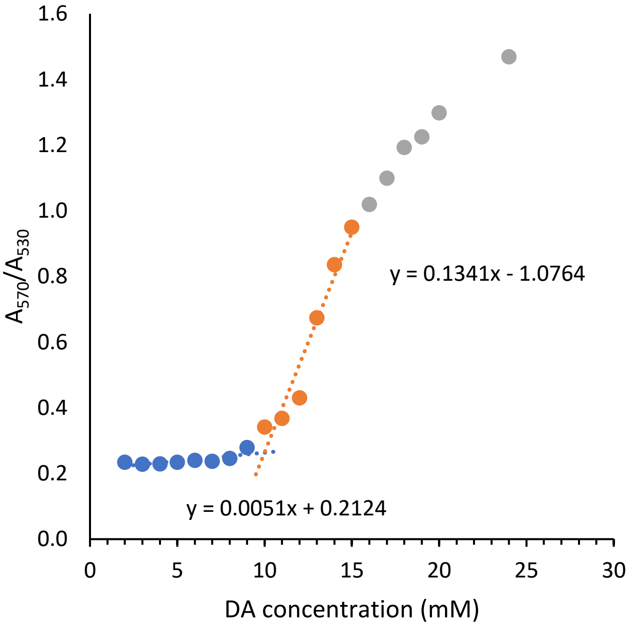

Figure S247. Determination of the critical aggregation concentration of DA for DA:PLA fresh monomers at 1:1 molar ratio. Absorption spectra of merocyanine 540 in the presence of increasing concentrations of DA obtained for DA:PLA fresh monomers at 1:1 molar ratio at pH 6.8 (A). Absorption ratio at 570 nm and 530 nm as a function of DA concentration. The intersection between the two fitting lines represents the CAC of DA – ca. 10 mM (B).

A

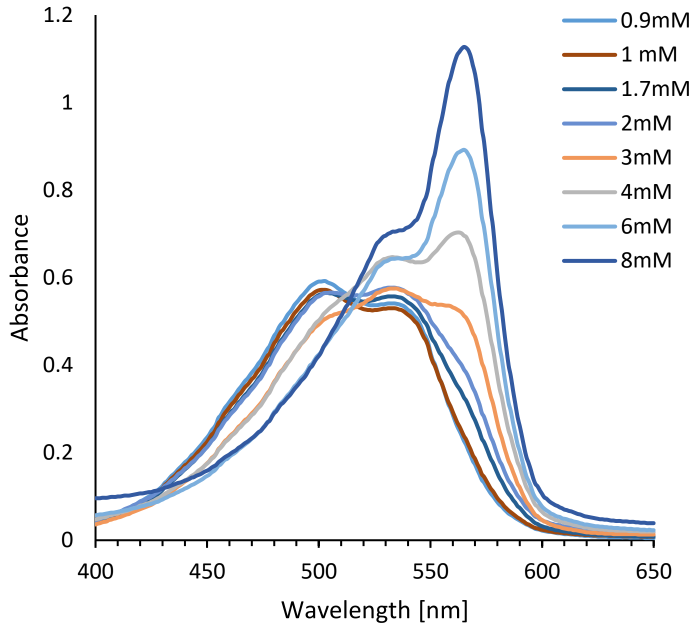

B

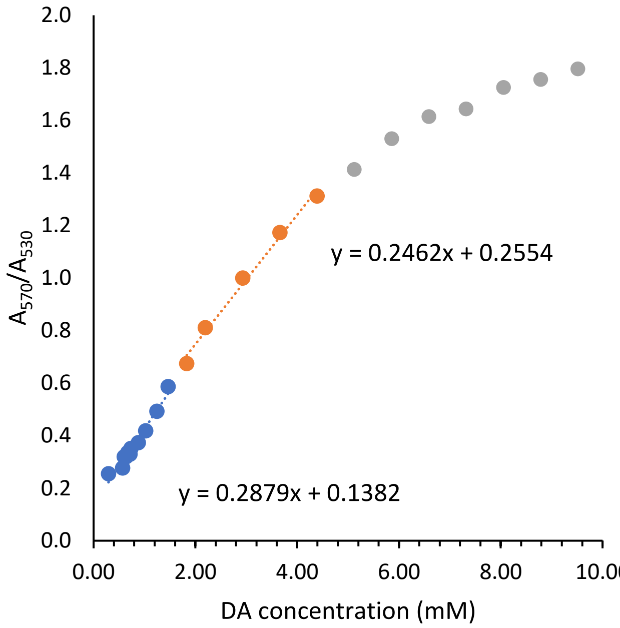

Figure S248. Determination of the critical aggregation concentration of DA for DA:PLA reaction product at 1:1 molar ratio. Absorption spectra of merocyanine 540 in the presence of increasing concentrations of DA obtained for the reaction product of DA:PLA at a 1:1 molar ratio at pH 6.8 (A). Absorption ratio at 570 nm and 530 nm as a function of DA concentration. The intersection between the two fitting lines represents the CAC of DA – ca 3 mM (B).
