# Supplementary material for: Hydroxy acid conjugation to lipids increases structural and hydrolytic stability
Source: Nat Commun. 2026 Jul 27;17:6973. doi: 10.1038/s41467-026-75192-5 (PMC13408983; doi:10.1038/s41467-026-75192-5)
Supplement: Supplementary file 2 — Transparent Peer Review file [file 41467_2026_75192_MOESM2_ESM.pdf]

nature portfolio

# Peer Review File

# Hydroxy Acid Conjugation to Lipids Increases Structural and Hydrolytic Stability

Corresponding Author: Dr Moran Frenkel-Pinter

**This file contains all reviewer reports in order by version, followed by all author rebuttals in order by version.**

Version 1:

Reviewer comments:

Reviewer #1

(Remarks to the Author)
The rigorous chemical analysis in this work is very good. The role of the mutualism between oligoester and decanoic acid in enhancing the self-assembly properties of decanoic acid and the protection from hydrolysis conferred by the vesicle on the oligoester is convincingly demonstrated by combining detailed LC-MS, NMR, and FTIR structural data with functional self-assembly and stability assays. The authors show that covalent conjugation to oligoester lowers the critical aggregation concentration of decanoic acid and dramatically enhances compartment formation, while self-assembly into vesicles provides clear physical sequestration that slows hydrolytic degradation of the oligoester

However, I have a few issues with the conceptualization of the paper, specifically how the authors has framed this work.

1. The use of “homoester” in the paper is indeed confusing. What the authors call “homoester” actually refers to oligoesters/polyesters formed exclusively from repeated hydroxy acid units without other structural diversity. In contrast, their “heteroester” products are simply hydroxy acid oligoesters with an additional ester bond formed to a fatty acid, i.e., decanoic acid. The distinction comes down to whether the ester oligomer is composed of only hydroxy acids (homoester/oligoester) or includes a fatty acid moiety attached (heteroester/fatty acid–linked oligoester). Clarifying this terminology would help prevent confusion, since both product classes are fundamentally oligoesters—just with or without a lipid conjugation.

2. The authors should specify how short prebiotic fatty acids compare to phospholipids in the introduction.

3. I don’t think references 33 and 34 accurately reflect the molecular diversity of the early Earth. There are several stronger and more relevant examples that the authors should consider citing, including the well-known Schmitt-Kopplin PNAS (2010) paper, Andrew Surman's PNAS (2019) paper, and Wołos et al.'s Science (2020) paper.

4. The author does not cite the earlier researchers who produced the first prebiotifc oligoester paper in 2018, using the same HA as in this study (except for MA, which was used elsewhere). The direction the author is taking aligns with the concept of a non-biomolecular prebiotic-molecule scenario, which has already been discussed in papers by Chandru and Jia. While most contemporary molecular building blocks in prebiotic chemistry are biomolecules, there is growing interest in the field of non-biomolecular prebiotic chemistry that may have scaffolded the origins of life on Earth or may have been the “building blocks” of extraterrestrial life elsewhere. I think the author’s paper is moving in that direction; if not, I am unsure why one would focus on only a single class of molecules (in this case, HA) as being especially relevant to prebiotic chemistry. The paper can be experimentally valuable in presenting their ideas by at least linking their work on non-biomolecular prebiotic chemistry scenarios, which I think will be highly relevant for advancing non-biomolecular origin-of-life models.

5. The method described in the paper for conjugating a hydroxy acid with a fatty acid is very similar to that reported by Lago et al. (2018) https://www.mdpi.com/2077-0375/12/9/858, who used 14 different amino acids instead of hydroxy acids to demonstrate ester bond formation with decanol. Notably, the use of the four types of hydroxy acids in this manuscript is motivated by the fact that they are congeners of amino acids (with the $NH_2$ group replaced by an OH group). However, Lago et al. (2018) were neither cited nor discussed in the manuscript.

6. The author must present a table to show all the products that were formed. How many n-mers of oligoesters and

oligoester-lipid compounds, etc, can be easily known from a table.

7. Additionally, the authors have demonstrated that the single step dry method, under similar temperatures, can produce oligoesters; however, they didn't compare their work with earlier work done by Chandru 2018, which could reveal differences in the oligoester product presented in this manuscript.

8. When the authors use the term "aggregates", what they mean is membraneless droplets made by oligoesters. The term was already used by Jia et al when they showed that their oligesters were forming membraneless microdroplets with ACN as a surfactant (they have also reported elsewhere that a surfactant is not necessary to show droplet formation. I think the author should mention this term.

9.Additionally, the encapsulation of Fatty acids on the authors' oligoester "aggregates" was confirmed by Jia et al.'s use of an amphiphilic dye, Rhodamine-PE, on their droplets. I think this should be mentioned in the manuscript.

10. What seems to be happening, under the subtopic "The universality of mutualism between hydroxy acids and lipids" and the overall manuscript, to be honest, is a conceptual slippage on the author's part in their attempt to frame oligoester/polyesters as a kind of "proto-peptide." Without denying that there may be some heuristic value in drawing analogies with peptide chemistry, the terminology is misleading. The term "proto-peptide" appears to derive from the depsipeptide literature, in which mixed ester–amide oligomers are indeed discussed as plausible transitional polymers between peptides and polyesters. However, in this case, the systems described are clearly prebiotic polyesters/ oligoesters, which are well-established classes of prebiotic polymers with their own mechanistic and structural rationale in origin-of-life research. It would therefore be more consistent (and less confusing to the reader) to adopt the established nomenclature rather than introducing a redundant or hybrid term.

11. There is a typo in page 20. A period is missing after "...540 assay."

Reviewer #2

(Remarks to the Author)
The manuscript by Edri et al. demonstrates that hydroxyacids can react with decanoic acid and other lipids, and that these conjugated molecules are both more stable to hydrolysis and capable of forming membranes. The study is very detailed and appears to be carefully conducted. The results are highly significant and impactful in the field of prebiotic chemistry and the origins of life. The data appear to support the conclusions and provide important and creative results to the community. After minor revisions (as suggested below), I believe the manuscript is suitable for publication in Nature Communications. I commend the authors for their work on such a detailed, comprehensive, and impactful study.

1) If I'm understanding correctly, reactions were carried out entirely in the dry state. At a few points in the manuscript, this wasn't entirely clear, which I've noted in the attached file. If this is indeed the case, how were reactions mixed together and what procedures were done to ensure uniform mixing/distribution between various reactions? I could imagine that unmixed vs. mixed reactions, or the degree of mixing, could play a big role in the reaction rates or yields. Along similar lines, how would changing the mass of the reactants (while keeping mole ratios constant) change or not change the reaction products?

2) To investigate the size and shape of the structures formed, microscopy was used and some conclusions were drawn about sizes and numbers of vesicles. While microscopy is valuable for visualizing the structures, could a technique such as dynamic light scattering be used to obtain a more wholistic view of the size distribution? If so, perhaps more quantitative statements could be made about the trends in sizes between samples, which could be beneficial.

3) The use of rhodamine to dye the membranes of vesicles is certainly valuable and much of the microscopy appears convincing that structures are forming. However, in some of the images where "deformed vesicles" are stated to be present, does rhodamine dye alone allow for distinct determination of vesicles, or is it possible that these are some type of lipid aggregate that does not include an inner aqueous component? The authors could attempt to encapsulate an aqueous dye inside these structures, purify any unencapsulated dye, and visualize by microscopy to answer this question.

Minor comments as noted in the attached pdf.

Reviewer #3

(Remarks to the Author)
The authors explore a main class of condensation product arising from mixtures of hydroxy acids and fatty acids as a model system representing the condensation products for amphiphiles with polymer-forming building blocks. The most interesting species to emerge are the heteroesters of lipid-hydroxy acid, where the presence of the hydroxy acid oligomer decreases the critical aggregation of the self-assembled structures, and the presence of the self-assembled lipid structures protects the hydroxy acid oligomers from hydrolysis.

The key missing insight is whether this heteroester species is actually useful - does the presence of the oligomer alter (improve/compromise) the encapsulation properties and/or permeability of the self-assembled structures? Does the presence of the lipid alter whether hydroxy acid oligomers conserve their functon(indeed, this is a major missing point - what

is the function and thus what is the purpose of preventing hydrolysis of hydroxy acid polymers?) I could imagine that such lipids might nucleate the formation of hydroxy acid droplets (e.g. refs 38-40) at the membrane interface, which could lead to a functional difference from just a fatty acid membrane encapsulating a condensate. With these additional insights, this paper would be much more impactful.

Here are the minor suggestions:
- Introduction: it's quite long, and could do with a rewrite to focus on the key background details important to this work. Parts seem incorrect (e.g. first paragraph ignores condensates as a type of compartment), irrelevant (e.g. end of first and third paragraph's discussion about permeability, which isn't addressed in this work, and entire second paragraph).
- Typos - pKa should be formatted correctly, there should be spaces between numbers and units
- Keep ratio order consistent e.g. HA:DA then DA:LA is confusing
- What is the 'total associative hydrophobicity' (top pg 13)
- Pg 14 - 'molar' missing 'ratio'?
- Ref 41 raises an interesting point, the present result should be discussed more in this context
- "The universality of mutualism…' title is confusing, perhaps "Effect extends to other fatty acids"
- Fig 3 - the vesicle images are low magnification and low resolution. Encapsulation of an aqueous dye, followed by dilution, could inform of how viable the self-assembled structures are as vesicles.
- Fig 4 - "ill-defined" lipid structures. Are they? They seem like vesicles.

Version 2:

Reviewer comments:

Reviewer #1

(Remarks to the Author)
The authors responded apt to all my queries.

Reviewer #2

(Remarks to the Author)
The authors have adequately responded to my comments from the initial version of the manuscript. I think the manuscript is suitable for publication in its current form. I would like to commend the authors for such a detailed and methodical response to these comments, and for a compelling and significant piece of scientific work.

Reviewer #3

(Remarks to the Author)
I appreciate the substantial additional experimental work undertaken in response to my earlier comments. The new encapsulation and leakage experiments do improve the manuscript and help clarify that the DA-LA reaction products are capable of forming cargo-containing assemblies.

I do, however, still have an important methodological concern regarding the calcein assay. I am curious why 5 mM calcein was chosen, since this is typically in a regime where increasing local calcein concentration can reduce fluorescence because of self-quenching. In that case, fluorescence changes may not map straightforwardly onto encapsulated amount. I therefore encourage the authors to comment explicitly on the validity and limitations of the conclusions drawn from this assay, and to moderate the quantitative interpretation accordingly unless further validation is available.

The revised manuscript tends to frame the increased permeability of the reaction products as potentially advantageous in a prebiotic context. At the same time, the authors note that this effect may arise from smaller vesicle size, higher curvature, and/or the presence of micelles that could destabilize vesicles in a detergent-like manner. I therefore encourage a more balanced discussion, since permeability and leakiness are two sides of the same coin, and in DA-based systems an increase in permeability may just as well compromise retention and compartment integrity rather than improve function. In that sense, the observed behavior could reflect a substantial functional cost even if the new species are more persistent.

More broadly, I think the manuscript would be stronger if the Introduction and Conclusions were reframed to better articulate why this chemistry matters. At present, the work risks reading mainly as a demonstration that one prebiotically plausible species reacts with another to give mixed products with altered physicochemical behavior, without yet making clear why those changes are important. I do think there are plausible functional consequences worth highlighting more explicitly, even if they remain hypotheses at this stage. For example, lipid-conjugated oligoesters might promote interfacial chemistry at membrane surfaces, might alter uptake-versus-retention behavior in primitive compartments, or might enable more complex hierarchical compartmentalization under alternative preparation conditions. The manuscript would benefit from presenting these as concrete possible consequences of the reported chemistry, while clearly distinguishing them from effects that have actually been demonstrated here.

Finally, regarding fit for Nature Communications, I think this depends strongly on the framing. If the work is presented primarily as an observation that one prebiotically plausible species reacts with another to generate mixed products with

altered physicochemical behavior, the broader significance is limited. If, however, the Introduction and Conclusions are reframed to more clearly articulate the possible functional consequences of this chemistry for prebiotic compartment behavior and the emergence of coupled structure-function relationships, then the relevance to the journal's readership would be much stronger.

Version 3:

Reviewer comments:

Reviewer #3

(Remarks to the Author)
The revisions have addressed my concerns.

**Open Access** This Peer Review File is licensed under a Creative Commons Attribution 4.0 International License, which permits use, sharing, adaptation, distribution and reproduction in any medium or format, as long as you give appropriate credit to the original author(s) and the source, provide a link to the Creative Commons license, and indicate if changes were made.
In cases where reviewers are anonymous, credit should be given to 'Anonymous Referee' and the source.
The images or other third party material in this Peer Review File are included in the article's Creative Commons license, unless indicated otherwise in a credit line to the material. If material is not included in the article's Creative Commons license and your intended use is not permitted by statutory regulation or exceeds the permitted use, you will need to obtain permission directly from the copyright holder.
To view a copy of this license, visit https://creativecommons.org/licenses/by/4.0/

# Response to Reviewers

**Reviewer #1 (Remarks to the Author):**

**Reviewer 1, Comment 1.** The rigorous chemical analysis in this work is very good. The role of the mutualism between oligoester and decanoic acid in enhancing the self-assembly properties of decanoic acid and the protection from hydrolysis conferred by the vesicle on the oligoester is convincingly demonstrated by combining detailed LC-MS, NMR, and FTIR structural data with functional self-assembly and stability assays. The authors show that covalent conjugation to oligoester lowers the critical aggregation concentration of decanoic acid and dramatically enhances compartment formation, while self-assembly into vesicles provides clear physical sequestration that slows hydrolytic degradation of the oligoester

We thank the reviewer for this positive evaluation. We are also pleased that the reviewer finds the chemical analysis rigorous.

**Reviewer 1, Comment 2.** However, I have a few issues with the conceptualization of the paper, specifically how the authors has framed this work.

We thank the reviewer for raising this point. We agree that this work can be framed from different perspectives and can emphasize different, valid lines of inquiry within chemical evolution. While one possible framing would be in terms of a "non-biomolecular prebiotic molecule" scenario or a "lipid world" perspective, we intentionally chose a broader conceptualization. Our aim here is not to position the system within a specific origin-of-life scenario, but rather to use it as a model system to investigate the coupling between compartmentalization and oligomerization, and the mutualistic interactions that can emerge from this coupling. We agree that the molecules generated in this study (lipid-conjugated oligoesters) may also be relevant to non-biomolecular prebiotic chemistry scenarios, and we address this point further in our response to Reviewer 1, Comment 6.

**Reviewer 1, Comment 3.** 1. The use of "homoester" in the paper is indeed confusing. What the authors call "homoester" actually refers to oligoesters/polyesters formed exclusively from repeated hydroxy acid units without other structural diversity. In contrast, their "heteroester" products are simply hydroxy acid oligoesters with an additional ester bond formed to a fatty acid, i.e., decanoic acid. The distinction comes down to whether the ester oligomer is composed of only hydroxy acids (homoester/oligoester) or includes a fatty acid moiety attached (heteroester/fatty acid–linked oligoester). Clarifying this terminology would help prevent confusion, since both product classes are fundamentally oligoesters—just with or without a lipid conjugation.

We thank the reviewer for pointing out this ambiguity. We agree that the terminology "homoester" and "heteroester," as originally used, may be confusing. We changed the terms "homoesters" and "heteroesters" to "hydroxy acid oligoesters" (or simply "oligoesters" in some cases) and "lipid-conjugated oligoesters", respectively, across the entire manuscript, to prevent confusion.

**Reviewer 1, Comment 4.** 2. The authors should specify how short prebiotic fatty acids compare to phospholipids in the introduction.

The following clarification was added:

> "While most abundant prebiotic fatty acids are commonly short (up to C12-C15),[26,27] contemporary phospholipids contain long-chain acids (typically C16-C26).[28]"

**Reviewer 1, Comment 5.** 3. I don't think references 33 and 34 accurately reflect the molecular diversity of the early Earth. There are several stronger and more relevant examples that the authors should consider citing, including the well-known Schmitt-Kopplin PNAS (2010) paper, Andrew Surman's PNAS (2019) paper, and Wołos et al.'s Science (2020) paper.

References were added.

**Reviewer 1, Comment 6.** 4. The author does not cite the earlier researchers who produced the first prebiotifc oligoester paper in 2018, using the same HA as in this study (except for MA, which was used elsewhere). The direction the author is taking aligns with the concept of a non-biomolecular prebiotic-molecule scenario, which has already been discussed in papers by Chandru and Jia. While most contemporary molecular building blocks in prebiotic chemistry are biomolecules, there is growing interest in the field of non-biomolecular prebiotic chemistry that may have scaffolded the origins of life on Earth or may have been the "building blocks" of extraterrestrial life elsewhere. I think the author's paper is moving in that direction; if not, I am unsure why one would focus on only a single class of molecules (in this case, HA) as being especially relevant to prebiotic chemistry. The paper can be experimentally valuable in presenting their ideas by at least linking their work on non-biomolecular prebiotic chemistry scenarios, which I think will be highly relevant for advancing non-biomolecular origin-of-life models (add reference to Chandru and Jia).

We thank the reviewer for pointing out this oversight regarding the original prebiotic polyester paper in 2018. We added that paper along with several additional relevant papers (#59-63):

- Chandru, K., Jia, T. Z., Mamajanov, I., Bapat, N. & Cleaves, H. J. Prebiotic oligomerization and self-assembly of structurally diverse xenobiological monomers. *Scientific Reports* **10** (2020). https://doi.org/10.1038/s41598-020-74223-5
- Mamajanov, I. *et al.* Ester formation and hydrolysis during wet–dry cycles: generation of far-from-equilibrium polymers in a model prebiotic reaction. *Macromolecules* **47**, 1334-1343 (2014).
- Chandru, K. *et al.* Simple prebiotic synthesis of high diversity dynamic combinatorial polyester libraries. *Communications Chemistry* **1**, 30 (2018).
- Chandru, K., Mamajanov, I., Cleaves, H. J. & Jia, T. Z. Polyesters as a Model System for Building Primitive Biologies from Non-Biological Prebiotic Chemistry. *Life* **10**, 6 (2020). https://doi.org/10.3390/life10010006
- Chandru, K., Potiszil, C. & Jia, T. Z. Alternative Pathways in Astrobiology: Reviewing and Synthesizing Contingency and Non-Biomolecular Origins of Terrestrial and Extraterrestrial Life. Life 14, 1069 (2024). https://doi.org/10.3390/life14091069

Regarding the comment about the concept of a non-biomolecular prebiotic-molecule scenario, while we have established a model system to investigate the coupling between compartmentalization and oligomerization, we agree with the reviewer that this concept is also very relevant in the context of our work. We modified the sentence regarding hydroxy acids in the Introduction as follows:

> "HAs have been suggested as key non-biomolecular players in prebiotic chemistry, and the polymerization and aggregation properties of their resulting oligoesters have been thoroughly studied.[56-63]"

We also added the relevant references in this sentence, as suggested.

**Reviewer 1, Comment 7.** 5. The method described in the paper for conjugating a hydroxy acid with a fatty acid is very similar to that reported by Lago et al. (2018) https://www.mdpi.com/2077-0375/12/9/858, who used 14 different amino acids instead of hydroxy acids to demonstrate ester bond formation with decanol. Notably, the use of the four types of hydroxy acids in this manuscript is motivated by the fact that they are congeners of amino acids (with the $NH_2$ group replaced by an OH group). However, Lago et al. (2018) were neither cited nor discussed in the manuscript.

We thank the reviewer for pointing out the relevant work by Lago *et al.*, which demonstrates esterification between amino acids and decanol. We agree that this work is relevant as a reference and added the following sentence in the Introduction:

> "Other non-covalent and covalent associations of lipids have been reported,[42-46] providing evolutionary advantages and underlining the importance of cooperation between different classes of chemical species during molecular evolution.[33,34,47]"

That said, the overlap is mainly at the general reaction class (a single esterification between the carboxylic acid of the amino acid and decanol's hydroxyl group, no further oligomerization is involved), while the chemical space and resulting products in our study are fundamentally different. In Lago *et al.*, the products are amino-acid alkyl esters and do not involve polymerization, as opposed to the bifunctional hydroxy acid molecules in our paper that enable oligomer growth. Consequently, our drying reactions that yield both hydroxy acid oligoesters and lipid-conjugated oligoesters enable us to test mutualistic coupling between polymerization, self-assembly, and hydrolytic persistence, which is a regime not accessed by the amino-acid alkyl ester system (or any other reported prebiotically-relevant system thus far).

**Reviewer 1, Comment 8.** 6. The author must present a table to show all the products that were formed. How many n-mers of oligoesters and oligoester-lipid compounds, etc, can be easily known from a table.

It's a great idea. We added Table 1 that summarizes this information in the Results and Discussion section.

**Table 1.** The extent of oligomerization of both hydroxy acid oligoesters and lipid-conjugated oligoesters in the absence and presence of decanoic acid (DA).

| | **In the absence of DA** | **In the presence of DA** | |
|---|---|---|---|
| **Hydroxy acid** | **Hydroxy acid oligoesters** | **Hydroxy acid oligoesters** | **Lipid-conjugated oligoesters** |
| Glycolic acid | 14-mer | 12-mer | 6-mer |
| Lactic acid | 18-mer | 13-mer | 12-mer |
| Malic acid | 17-mer | 16-mer | 5-mer |
| Phenyllactic acid | 7-mer | 7-mer | 6-mer |

**Reviewer 1, Comment 9.** 7. Additionally, the authors have demonstrated that the single step dry method, under similar temperatures, can produce oligoesters; however, they didn't compare their work with earlier work done by Chandru 2018, which could reveal differences in the oligoester product presented in this manuscript.

We added the following sentence in the Results and Discussion section:

> “The oligomerization degree of the HAs formed in the absence of DA is in good agreement with previous studies, although we observed deviations in the maximum oligomer length detected, likely due to differences in analytical methodology.[61,65]”

**Reviewer 1, Comment 10.** 8. When the authors use the term “aggregates”, what they mean is membraneless droplets made by oligoesters. The term was already used by Jia et al when they showed that their oligesters were forming membraneless microdroplets with ACN as a surfactant (they have also reported elsewhere that a surfactant is not necessary to show droplet formation. I think the author should mention this term.

In the manuscript we used the term “aggregate” to denote any form of physical association between molecules (e.g., clustering, phase-separated domains, vesicles), without implying a specific geometry. We first used this terminology when describing LA oligoesters. However, in our case, oligoesters of LA are predominantly observed as disorder clusters and not as microdroplets, as can be seen in figure S197. This can be explained by the difference in methodology compared to previous reports, where in this paper we resuspended samples in water and not in water-acetonitrile mixture. To clarify this, we added the following to Results and Discussion:

> “Throughout this work, we use the term ‘aggregates’ broadly to refer to any form of molecular association (e.g., clustering, phase-separated domains, vesicles), without implying a particular morphology or degree of structural order.”

> “While LA-derived oligoesters have been reported to form microdroplets when dispersed in water-acetonitrile mixtures,[56,59,65] we did not observe microdroplet formation under our conditions, likely due to differences in sample preparation.”

**Reviewer 1, Comment 11.** 9.Additionally, the encapsulation of Fatty acids on the authors' oligoester “aggregates” was confirmed by Jia et al.'s use of an amphiphilic dye, Rhodamine-PE, on their droplets. I think this should be mentioned in the manuscript.

We assume the reviewer refers to the following original text:” Under a fluorescent microscope, the samples consisting of DA and LA free oligoesters without DA-LA conjugated oligoesters were found to form vesicles of various sizes for which the membrane was visualized. Aggregates related to LA oligomers were also observed (Figures S197).” As mentioned, under our conditions, oligoesters of LA are predominantly observed as disorder clusters and not as microdroplets.

This text refers to a control experiment that was designed to examine the interactions between LA oligoesters and non-reacted DA. Under our conditions (pH 6.8, 50 mM phosphate buffer and 100-200 mM LA, referring to the initial amount prior to reaction), we did not observe microdroplet formation nor encapsulation of DA within such structures.

Recently, Jia and colleagues reported in a ChemRxiv paper the assembly of phospholipids around polyphenyllactate droplets, which is very interesting (https://doi.org/10.26434/chemrxiv-2025-l8dvx). We added the following text in the Results and Discussion:

> “Recently, non-covalent interactions between phospholipids and HA assemblies were reported by Jia and colleagues, in which the assembly of phospholipids around polyphenyllactate droplets has been observed.[76]”

**Reviewer 1, Comment 12.** 10. What seems to be happening, under the subtopic "The universality of mutualism between hydroxy acids and lipids" and the overall manuscript, to be honest, is a conceptual slippage on the author's part in their attempt to frame oligoester/polyesters as a kind of "proto-peptide." Without denying that there may be some heuristic value in drawing analogies with peptide chemistry, the terminology is misleading. The term "proto-peptide" appears to derive from the depsipeptide literature, in which mixed ester–amide oligomers are indeed discussed as plausible transitional polymers between peptides and polyesters. However, in this case, the systems described are clearly prebiotic polyesters/ oligoesters, which are well-established classes of prebiotic polymers with their own mechanistic and structural rationale in origin-of-life research. It would therefore be more consistent (and less confusing to the reader) to adopt the established nomenclature rather than introducing a redundant or hybrid term.

We changed "proto-peptide" to "proto-polymer" in that section. It now reads as follows:

> "In the past, decanoic acid (DA) and hydroxy acids (HAs) have been investigated separately in the context of origins of life as a protocell model amphiphile and as a proto-polymer model, respectively."

**Reviewer 1, Comment 13.** 11. There is a typo in page 20. A period is missing after "...540 assay."

Fixed.

## Reviewer #2 (Remarks to the Author):

**Reviewer 2, Comment 1.** The manuscript by Edri et al. demonstrates that hydroxyacids can react with decanoic acid and other lipids, and that these conjugated molecules are both more stable to hydrolysis and capable of forming membranes. The study is very detailed and appears to be carefully conducted. The results are highly significant and impactful in the field of prebiotic chemistry and the origins of life. The data appear to support the conclusions and provide important and creative results to the community. After minor revisions (as suggested below), I believe the manuscript is suitable for publication in Nature Communications. I commend the authors for their work on such a detailed, comprehensive, and impactful study.

We thank the reviewer for the positive feedback and for acknowledging the importance and relevance of our work in the origins of life field. We appreciate the reviewer's constructive suggestions and have addressed each point in detail below.

**Reviewer 2, Comment 2.** 1) If I'm understanding correctly, reactions were carried out entirely in the dry state. At a few points in the manuscript, this wasn't entirely clear, which I've noted in the attached file. If this is indeed the case, how were reactions mixed together and what procedures were done to ensure uniform mixing/distribution between various reactions? I could imagine that unmixed vs. mixed reactions, or the degree of mixing, could play a big role in the reaction rates or yields. Along similar lines, how would changing the mass of the reactants (while keeping mole ratios constant) change or not change the reaction products?

These are great questions. When starting this project, we put a lot of thought into the experimental design and considered multiple factors to maximize reproducibility and minimize errors. The reviewer is correct that the esterification reactions were performed in the dry state. We have clarified this as indicated in this response and in subsequent comments (see also responses to Reviewer 2, Comment 9 and to Reviewer 2, Comment 37). For example, we added the following in Methods:

“All reagents were used as received under neat (solvent-free) conditions.“

And the following in Results and Discussion:

> “Decanoic acid and single HAs (either GA, LA, MA, or PLA) were allowed to react under dry conditions in the absence of water other than water originating in the raw materials for 7 days at 85 °C at a 1:1, 1:2, and 1:4 molar ratio (in favor of the HAs).”

We decided to perform dry reactions and intentionally avoided aqueous premixing followed by evaporation (“dry-down”) because of the reaction conditions. The reactions occurred under acidic conditions (to favor esterification). Under these conditions, decanoic acid is largely protonated and poorly soluble in water, leading to rapid phase separation even after vigorous agitation. The rate at which decanoic acid undergoes phase separation is much faster compared to the evaporation rate of water in dry-down conditions. As a result, dry-down from an aqueous dispersion is expected to pass through a prolonged period of heterogeneity.

In contrast, in our dry-state protocol, the mixture is heated to 85 °C, well above the melting point of decanoic acid (~31 °C), resulting in a molten phase. This molten phase facilitates diffusive redistribution and mixing of reactants, mitigating imperfect initial spatial homogeneity. Consistent with this, we observed high reproducibility across independent repeats by HPLC analysis and structural characterization (Supplementary Figures S2-S21).

For this revision, to directly test whether increased pre-reaction homogeneity changes the outcome, we conducted the reaction (three independent repeats) between decanoic acid (DA) and the four tested hydroxy acids (glycolic acid (GA), lactic acid (LA), malic acid (MA), and phenyllactic acid (PLA)) at three different molar ratios, starting from vortexed water dispersions that were immediately frozen with liquid nitrogen and underwent lyophilization. Immediately after the sublimation process, lyophilized samples were incubated at 85 °C for 7 days and DA’s conversion was determined via HPLC and compared to the dry reactions that were presented in the original submission:

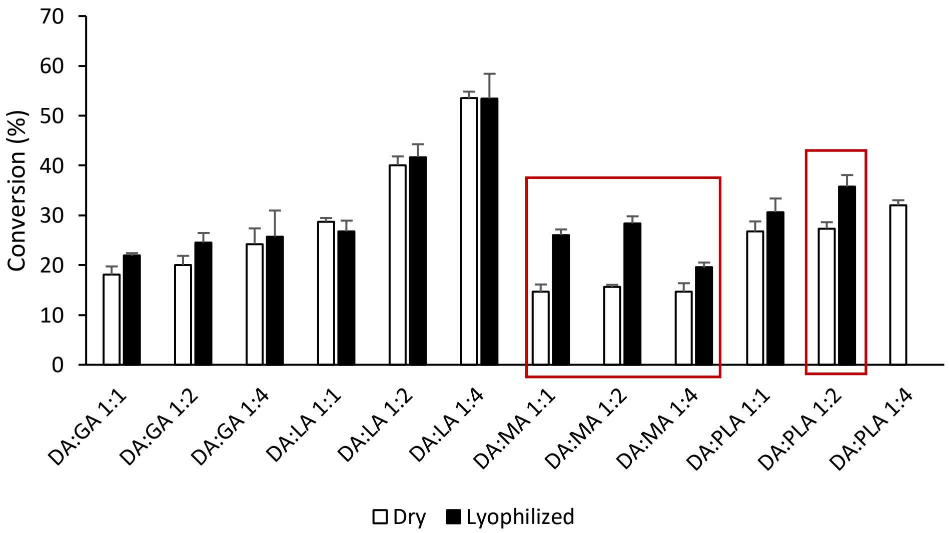


As can be seen from the graph, DA conversion is essentially unchanged between the original dry-state protocol and the lyophilized samples for most conditions. The main exception is malic acid (MA), for which at DA:MA 1:1 and 1:2 molar ratios, the measured conversion of DA was higher (27% compared to 14%). At a DA:MA 1:4 molar

ratio, only a minor increase was observed. For DA:PLA at a 1:2 molar ratio also minor increase in the conversion was observed. The physical state of the hydroxy acids seems to greatly affect the conversion. As was also mentioned in the manuscript, among the four hydroxy acids, MA and PLA are in a solid state at 85 °C (reaction temperature), whereas GA and LA are liquids at this temperature. Initial spatial homogeneity is expected to matter more when one reactant remains solid, and this is consistent with the observed behavior. We also confirmed that the product distribution obtained for the lyophilized samples is similar to that obtained for the dry product. For example, here is a comparison of chromatograms obtained for reactions of PLA and DA in dry vs. lyophilized samples:

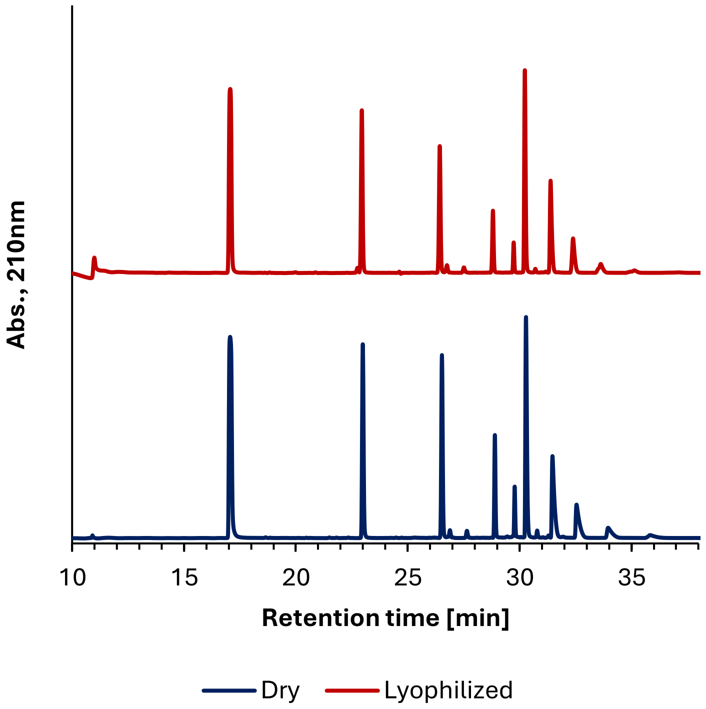


Altogether, **these additional experiments support our conclusion that our original dry-state approach provides robust, reproducible, and representative reaction outcomes**, and that the main conclusions of the manuscript are not dependent on initial mixing.

As for the absolute mass used for the experiments, it can affect the reaction yield. For this revision, we evaluated the effect of batch size (absolute mass used) on DA conversion for the reactions of DA and either LA or GA. For DA-LA mixtures we varied the masses at all tested molar ratios (1:1, 1:2, and 1:4), and for DA-GA we varied the masses at a 1:1 molar ratio (see the table below). All samples were prepared in triplicates.

| Mixture | 1:1 molar ratio (μmol/μmol) | 1:2 molar ratio | 1:4 molar ratio |
|---|---|---|---|
| DA:LA | 200:200<br>400:400<br>600:600<br>800:800<br>1000:1000 | 200:400<br>400:800<br>800:1600 | 200:800<br>400:1600<br>800:3200 |
| DA:GA | 200:200<br>400:400<br>800:800 | - | - |

For each mixture we determined DA conversion (see figures below) using HPLC analyses:

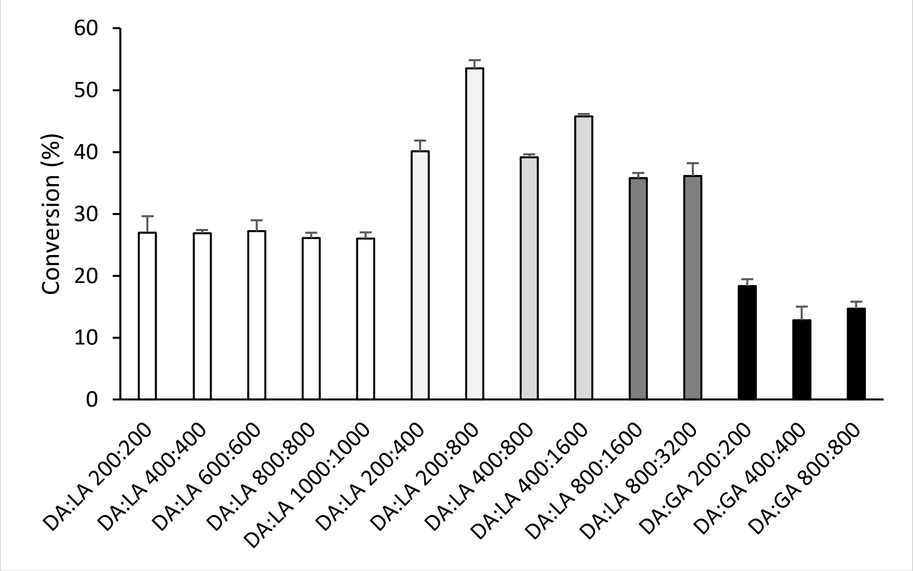


As demonstrated in the figure, for DA:LA at a 1:1 molar ratio, increasing the batch size from 200 to 1000 µmol (white bars) did not result in a significant change in DA conversion. In contrast, for GA, increasing the batch size (400 and 800 µmol) resulted in a slight decrease in DA conversion (from ~18% to 13% and 15%, respectively). We attribute this small effect to GA's relatively high reactivity and its propensity to undergo self-oligomerization under our conditions, which competes with GA–DA condensation. Upon scaling up the batch, the relative extent of these competing pathways may shift (e.g., due to differences in local reactant distribution and kinetics in the condensed phase), resulting in a slight reduction in the fraction of GA reacting with DA and, consequently, a small decrease in DA conversion.

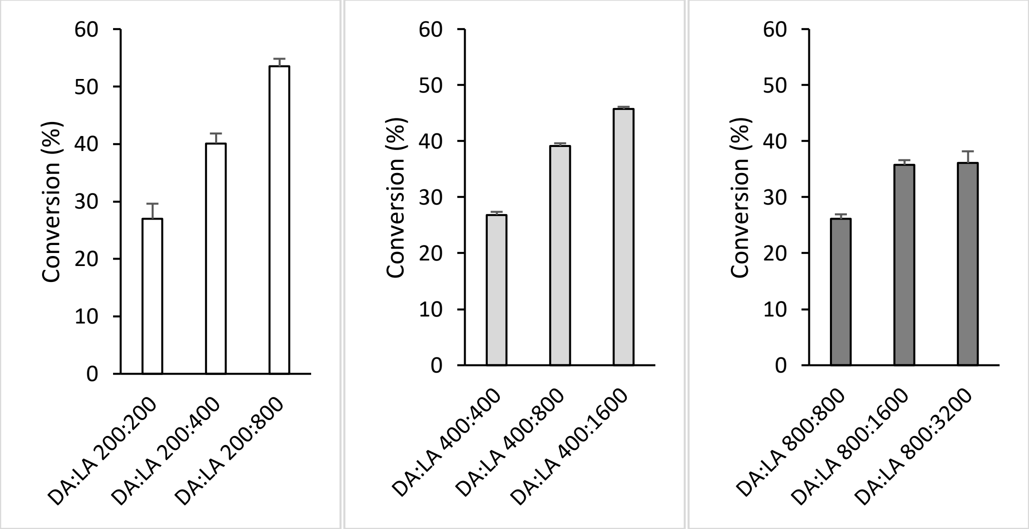


When examining the effect of DA:LA molar ratio on DA conversion across different batch sizes, we observe similar trends for 200 and 400 µmol DA, with conversion increasing as the LA fraction increases. However, for 800 µmol DA, conversion reaches a plateau at a 1:2 molar ratio (DA:LA). In addition, increasing batch size decreases DA conversion, most notably at a 1:4 molar ratio (~55%, 45% and 35% conversion for 200, 400, and 800 µmol, respectively). This trend is consistent with reduced effective reactant contact and mass transport limitations in the melted mixture.

As batch size increases, the effects of mixing, diffusion path length, reaction time, and reaction-vessel dimensions become increasingly important.

As another example of how reaction conditions affect yield in this system, we carried out the reaction between decanoic acid (200 µmol) and lactic acid (either 200, 400, or 800 µmol) under identical conditions, except that the reaction vessel differed in capacity and geometry, and we obtained higher yields (~76% regardless of the molar ratio). In the graph below, MTSC indicates 1.5mL microtube with a screwcap, MT indicates a standard "Eppendorf" 1.5mL microtube, and GV indicates 7mL glass vial:

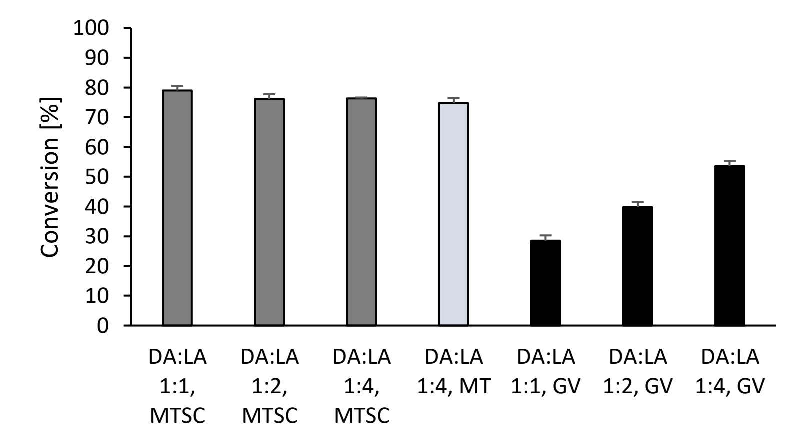


**To summarize, there are many parameters that can affect the reaction yield, including the batch size**. However, a systematic evaluation of each was beyond the scope of this work. Moreover, the different yields obtained at different DA:LA molar ratios in our original setup enabled us to more effectively monitor changes in structural behavior, underscoring the critical role of the balance between DA and DA–LA conjugates. We added the following to Results and Discussion:

> "When examining the effect of DA:LA molar ratio on DA conversion across different batch sizes, we observed similar trends for 200 and 400 µmol DA, with conversion increasing as the LA fraction increases (Figures S22-23). However, for 800 µmol DA, conversion reaches a plateau at a 1:2 molar ratio (DA:LA)."

We also added Figures S22-S23 to the SI.

**Reviewer 2, Comment 3.** 2) To investigate the size and shape of the structures formed, microscopy was used and some conclusions were drawn about sizes and numbers of vesicles. While microscopy is valuable for visualizing the structures, could a technique such as dynamic light scattering be used to obtain a more wholistic view of the size distribution? If so, perhaps more quantitative statements could be made about the trends in sizes between samples, which could be beneficial.

This is a great point. Dynamic light scattering (DLS) is a widely used and well-established technique to study size properties of various dispersed systems, particularly when the samples are relatively monodisperse. Micellar systems are typically monodispersions and routinely characterized with DLS. Vesicular systems are more complex since unlike micelles, monodispersity is not an inherent property of vesicles, and it is highly dependent on various parameters such as preparation method and post-preparation treatments (extrusion, filtration, homogenization, etc.). In our case, the vesicles were prepared without applying any of the treatments mentioned, resulting in polydisperse systems. Moreover, the presence of micelles in the reaction products poses an additional challenge as it significantly increases the polydispersity. As DLS provides an intensity-weighted size distribution, it can be dominated by the largest scatterers, which complicates quantitative interpretation for mixed and highly

polydisperse populations. For these reasons, DLS measurements of our samples could be challenging and would not necessarily provide the most accurate and realistic description of our systems.

Nonetheless, following this comment we conducted DLS measurements for DA control as well as for DA-LA fresh monomers and DA-LA reaction products at 1:1, 1:2 and 1:4 molar ratios. All measurements were carried out using a Zetasizer Nano-S instrument (Malvern Panalytical) with back scattering detection, scatter detection (173°) at 25°C (equilibration time: 120 sec). Each sample was measured 5 times. Optical settings, as well as measurement duration and time interval, were determined automatically by the software following an optimization step in which the measurement position and attenuation were assessed. All samples were prepared in triplicate and measured as described.

Initially we carried out preliminary measurements with the DA control at different concentrations in order to select an appropriate concentration for the remaining analyses. The best concentration was 25 mM, for which the attenuation levels, count rate, noise level (as indicated by the correlogram intercept), and reproducibility were acceptable. Once the concentration was selected, all samples (mentioned above) were measured. It is worth noting that the original working concentration used in all our tests in the original paper was 50 mM (with respect to DA). Hence, in order to keep sample preparation as consistent as possible, all samples were prepared with 50 mM DA in 50 mM phosphate buffer (PB) at pH 6.8 and further diluted with 50 mM PB at pH 6.8 to a final concentration of 25 mM for DLS measurements.

The figure below demonstrates representative correlograms obtained for the DA control (panel A), DA-LA fresh monomers at a 1:4 molar ratio (panel B), and DA:LA reaction products at a 1:4 molar ratio (panel C):

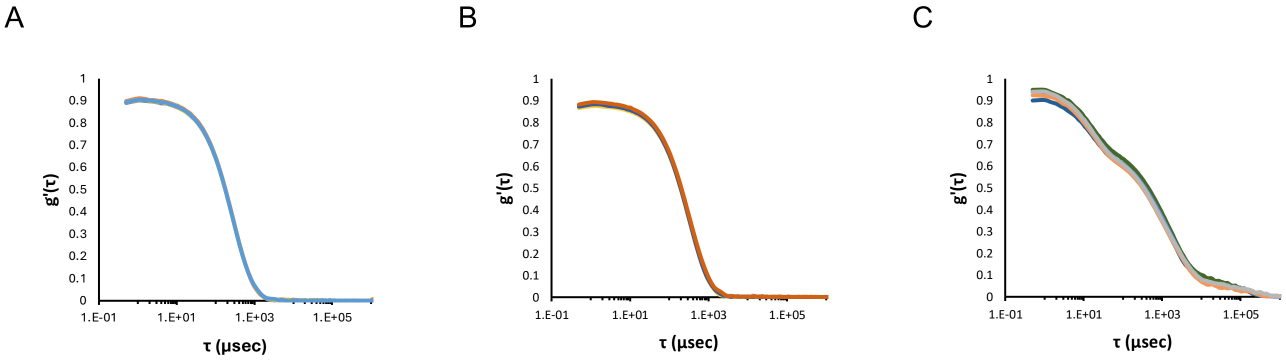


While the correlograms of both the DA control and the DA-LA fresh monomers showed a single decay, the correlogram obtained for the DA-LA reaction products distinctly exhibits two decays. As estimated by the cumulant algorithm, the slope of the initial part of the correlogram (at smaller τ), corresponds to the mean size (Z-average), while the later part, where the correlation function deviates from linearity, corresponds to the PDI. For both the DA control and the DA-LA fresh monomers, the correlograms almost overlap, suggesting very similar distributions in terms of size and polydispersity. In the case of the reaction products, the two decays represent two populations of distinct sizes. The first decay corresponds to the micellar system with a smaller size, and the second one represents a vesicular population with larger structures. Calculation of Z-ave and PDI as reported by the software are summarized in the figures below (F- fresh; RP – reaction products):

A

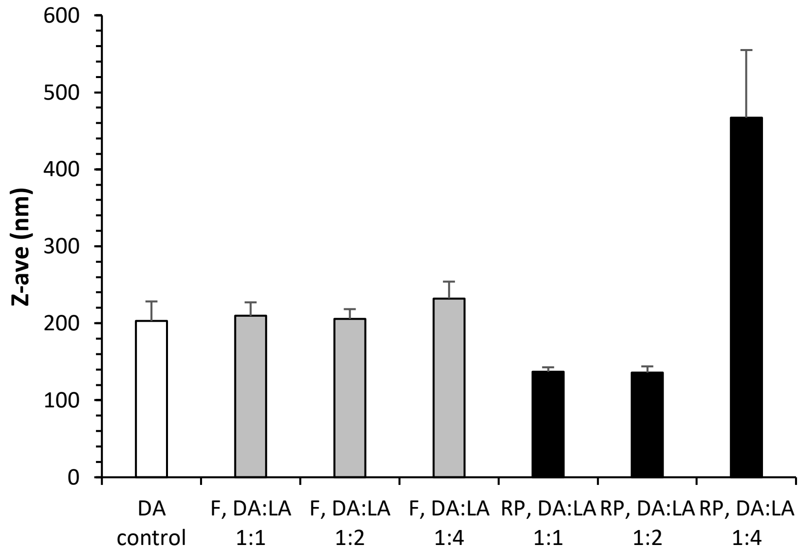


B

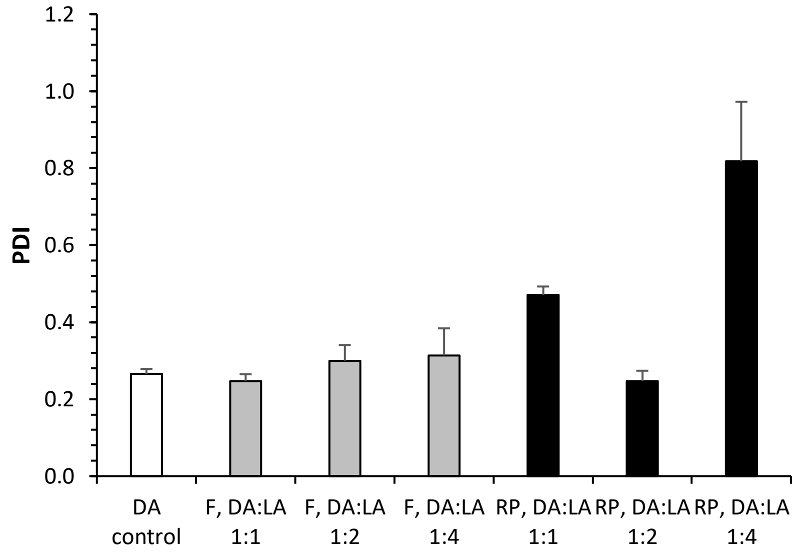


Panels A and B show the Z-ave and PDI values for all tested samples, respectively (error bars represent the standard deviation of the triplicates). As indicated by the figures, both Z-ave and PDI of the DA control and the DA-LA fresh monomers are comparable. DA-LA reaction products, however, do not exhibit a uniform trend. At a DA:LA 1:1 and a 1:2 molar ratio, Z-ave is smaller compared to the DA control ($p<0.001$), while at a DA:LA 1:4 molar ratio, the Z-ave is significantly larger ($p<0.001$). When comparing the PDI of the reaction products, at DA:LA 1:1 and 1:4 molar ratios, the PDI is larger than the PDI of the control sample ($p<0.001$), while at a DA:LA 1:2 molar ratio, the PDI is smaller compared to the control ($p<0.05$). As noted above, DLS measurements and particularly the cumulant algorithm provide reliable information primarily for monodisperse samples, which is clearly not the case for the DA:LA reaction product at a 1:4 molar ratio.

Thus, to properly characterize this system, we used the so-called ‘distribution analysis model’ that utilizes a non-negative least squares (NNSL) algorithm. This model considers a sum of multiple exponential decays and finds the best fit by minimizing the deviation from the measured data. The resulting distribution curve is the intensity particle size distribution, which can further be transferred into volume-weighted and number-weighted distributions. The following intensity-, volume-, and number-weighted distributions were obtained for the fresh monomers and reaction products of DA:LA at a 1:4 molar ratio:

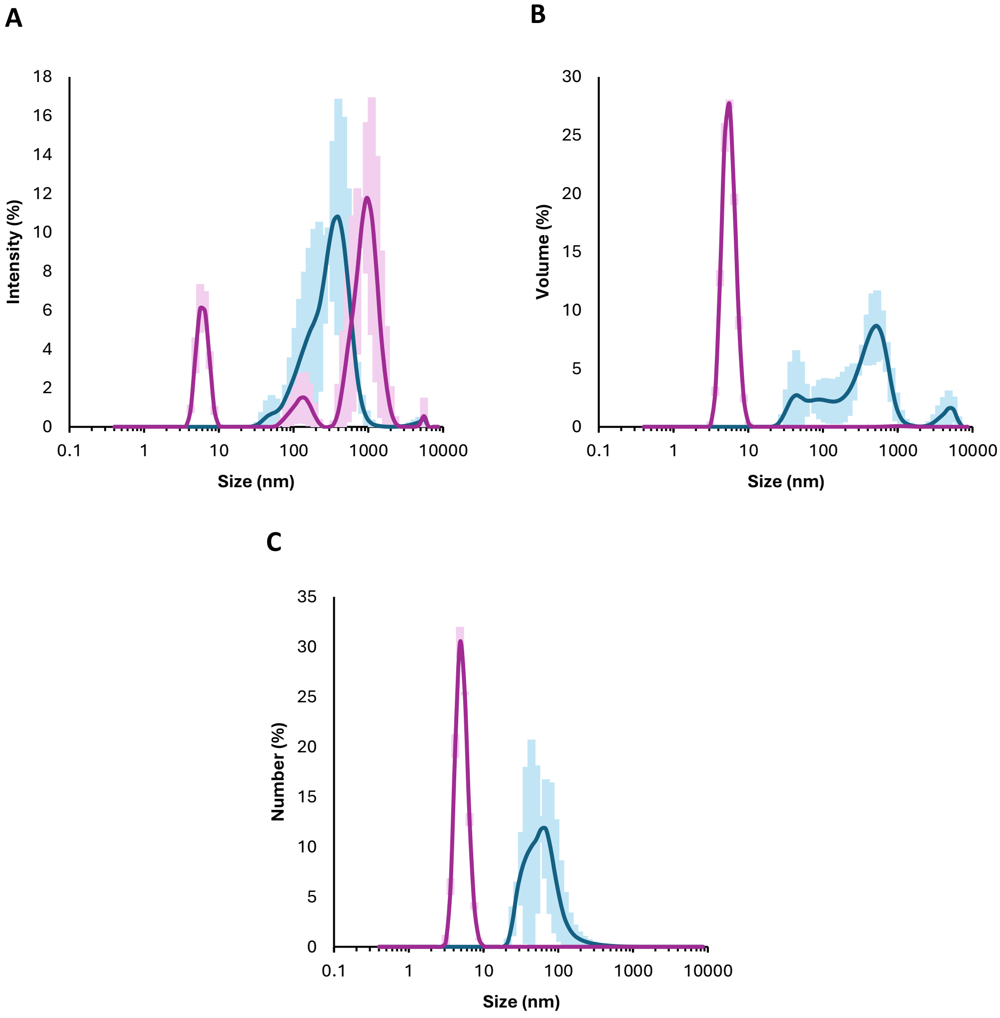


As indicated by the distribution curves, the vesicles formed in DA-LA fresh monomers are mostly within the submicron size. Remarkably, the reaction products are composed of two main, distinct populations that are detectable only in the intensity-weighted distribution. One population with a mean size of ca 960 nm (accounts for ca 70%) and another population with a mean size of ca 6 nm (accounting for ca 20%). However, in the volume- and number-weighted distributions, only the smallest population is observed. In general, the intensity-weighted distribution (obtained directly from the measured data) presents the measured intensity and therefore is more sensitive to larger particles, as anticipated by the Rayleigh approximation according to which $I \propto (d/2)^6$. The volume- and number-weighted curves (calculated from the intensity-based distribution) are more sensitive to smaller particles, which can explain the “disappearance” of the larger vesicle population.

The following text was added to the Results and Discussion under the section 'Structural characterization of the reaction products':

> "While microscopic methods provide high-resolution information, it is on the local level. Bulk characterization is essential to gain more comprehensive understanding of the explored systems. For that purpose, dynamic light scattering (DLS) measurements were employed. DA control as well as DA-LA fresh monomers and reaction products at all tested molar ratios were measured with a back-scattering DLS instrument, and based on the autocorrelation function, the size and polydispersity were determined. For DA:LA systems at 1:1 and 1:2 molar ratios, the average size obtained by the cumulant algorithm (Z-ave) was significantly lower for the reaction products compared to the fresh monomers (Figure S199-S200). For DA:LA 1:4 molar ratio samples, we used the size distribution profile obtained by the non-negative least square fit (Figure 4E-F and Figure S201) to compare the reaction products and fresh monomers. As indicated by Figure 4E, in the case of the fresh monomers, the intensity-weighted distribution demonstrates a broad population of structures with a maximum at ca 350 nm, while multiple populations were observed for the reaction products, as was also indicated in the correlograms (Figure S199). Two of them are within the nanometric and micron size ranges with a maximum at about 5.6 nm and 950 nm, respectively. When translating the intensity-weighted to a number-weighted distribution (Figure 4F), a clear single population of about 4-6 nm was observed for the reaction products, in agreement with cryo-TEM assessment of a micellar population."

The following text was added to Methods:

> "Dynamic light scattering (DLS) measurements. Mean particle size, polydispersity index, and size distribution were determined based on dynamic light scattering measurements. All measurements were carried out using a Nano-ZS instrument (Malvern Panalytical, Malvern, UK) equipped with a laser at a wavelength of 633 nm and a back scattering detector (173°). All measurements were carried out at 25°C following 120 sec equilibration step. Five repeat measurements were carried out for each sample. Instrument settings were automatically optimized. For DLS measurements, the samples were rehydrated as described above to reach DA concentration of 50 mM. The samples were diluted twice with 50 mM phosphate buffer at pH 6.8 to reach DA concentration of 25 mM. All DLS experiments were carried out in triplicates. Data was analyzed using Zetasizer software. Details on data analysis are provided in supplementary methods."

In addition, we added panels E-F to Fig. 4 and Supplementary Figures S199-S201.

Following the reviewer's comment, in addition to the DLS measurements, we also conducted a statistical analysis based on cryo-TEM micrographs. We measured the size (diameter) of at least 80 vesicles for each sample, taken from at least 3 regions in the grid (see the number of vesicles taken for the analysis in the table below). We considered only spherical or almost spherical vesicles for the statistical analysis. Micelles were not included in the analysis as their number is substantially higher. Yet we measured the diameter of several representative micelles and found that micelle size is about 4-6 nm, in agreement with the DLS measurements. Accordingly, the cryo-TEM-based statistics provide a complementary, morphology-based assessment of the vesicle size distribution.

The number of vesicles used for the statistical size distribution analysis based on cryo-TEM micrographs is given in the following table:

| | Fresh monomers | Reaction products |
|---|---|---|
| DA:LA 1:1 | 148 | 172 |
| DA:LA 1:2 | 83 | 185 |
| DA:LA 1:4 | 93 | 82 |

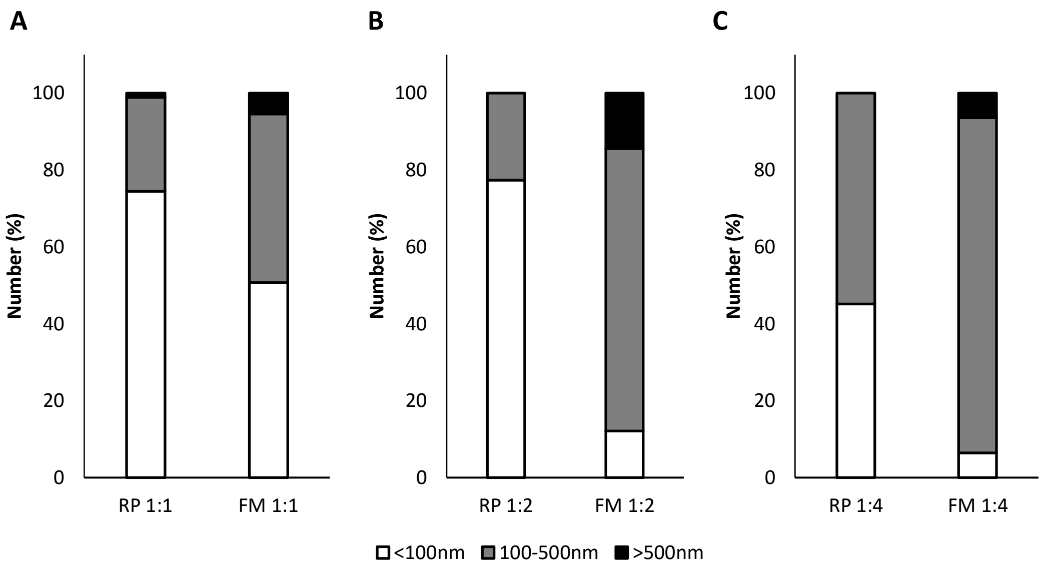


As indicated by the figures, when comparing the fresh monomers (FM) to the reaction products (RP), we could clearly see that the fraction of the smallest population of less than 100 nm is greater for the reaction products. In the case of the reaction products at a DA:LA 1:4 molar ratio, the distribution represents vesicles only, meaning that the fraction of structures less than 100 nm is much larger.

Together, Cryo-TEM and DLS analyses provide complementary information on size distribution. Cryo-TEM is well-suited for directly visualizing individual structures and is particularly informative for smaller vesicles in the submicron range, and provides morphology-based local information. DLS is a bulk technique that can provide information on structure size within a wider scale (from a few nanometers to >1μm). Moreover, shape and morphology are easily monitored using cryo-TEM, while standard DLS measurements provide information that assumes spherical scatters, as denoted by the hydrodynamic radius.

The following text was added to Results and Discussion under the section ‘Structural characterization of the reaction products’:

> “We complemented the DLS measurements with cryo-TEM–based vesicle size statistics (n≥80 vesicles per sample from ≥3 grid regions; micelles were excluded), which supported a greater fraction of <100 nm vesicles in the reaction products, especially at a DA:LA 1:4 molar ratio, while measured micelle diameters (4–6 nm) agreed with DLS (Figures S202-203).”

In addition, we added Supplementary Figures S202-S203.

**Reviewer 2, Comment 4.** 3) The use of rhodamine to dye the membranes of vesicles is certainly valuable and much of the microscopy appears convincing that structures are forming. However, in some of the images where "deformed vesicles" are stated to be present, does rhodamine dye alone allow for distinct determination of vesicles, or is it possible that these are some type of lipid aggregate that does not include an inner aqueous component? The authors could attempt to encapsulate an aqueous dye inside these structures, purify any unencapsulated dye, and visualize by microscopy to answer this question.

The deformed structures in the case of DA-LA fresh monomers were dyed with rhodamine 6G and for most of these structures, we could clearly see fluorescence consistent with a membrane boundary. However, we agree with the reviewer that membrane staining alone cannot unambiguously distinguish lumen-containing vesicles from other membrane/lipid aggregates. However, we think that this is unlikely, taking into consideration the conditions under which the vesicles were prepared (in terms of pH and DA concentration).

Following the reviewer's suggestion, we prepared the samples (DA control, fresh monomers of DA:LA at a 1:4 molar ratio, and reaction products of DA:LA at a 1:4 molar ratio) with 5 mM calcein (a hydrophilic dye). The vesicles were separated from free calcein using size exclusion chromatography (SEC; SepharoseTM 4B). We collected ten fractions (ca 0.5 mL each) and verified calcein encapsulation using both fluorescence measurements and fluorescence microscopy. The figure below obtained for the fourth fraction of DA-LA fresh monomers confirms that encapsulation occurred:

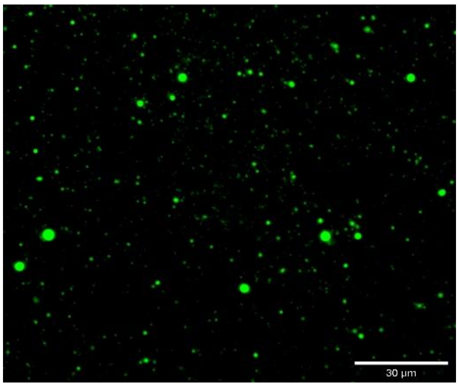


However, the deformed structures were not observed following this process of size exclusion. This can be attributed to several reasons, but mostly to the SEC treatment during which the sample is diluted and its small-molecule composition changes. It is possible that free LA is depleted from the vesicle-containing fractions during SEC (as expected for small molecules, which elute later than vesicles), and as a result, the membrane composition/packing shifts toward a more stable morphology, reducing the apparent fraction of "deformed" structures.

We added the following sentence to the Results and Discussion following the comment about the presence of deformed structures in fresh mixtures of DA and LA:

> "We cannot exclude the possibility that vesicle deformation observed under both fluorescence and electron microscopy might be due to the pressure and compression applied by the microscope cover slip and blotting step upon preparation.[86-88]"

Minor comments as noted in the attached pdf (copied here by the authors):

**Reviewer 2, Comment 5.** Does compartmentalization alone hinder hydrolysis? If molecules are partiitoned in the aqueous component of the compartment and if they don't interact with the membrane, would hydrolysis rates be expected to be different?

I don't disagree that interactions with the membrane could slow hydrolysis. Just wondering about the wording here.

This is a great point. Encapsulation of molecules in vesicles can take place in several locations (e.g., the aqueous lumen, the membrane-water interface, or within the bilayer itself). If a molecule remains fully partitioned in the aqueous lumen and does not interact with the membrane, one would not necessarily expect its intrinsic hydrolysis rate to differ from that in bulk solution, assuming identical pH and ionic strength. However, it is also important to consider that the nature of the inner aqueous core is not necessarily identical to the continuous aqueous phase in terms of local pH and ionic strength. These factors depend on osmotic gradients, membrane permeability, and surface charge, and such microenvironmental differences can influence hydrolysis rates. We found several additional papers that show that encapsulation can protect the encapsulated molecules from hydrolysis. However, in these references, the protected molecules are probably located at the membrane-water interface or within the bilayer itself. For better accuracy, we added to the Introduction the following sentence (in green) after the above-mentioned sentence (in black):

> "Compartmentalization can also hinder polymer hydrolysis, which is thermodynamically favorable in aqueous environments. This effect can be achieved, for example, when molecules partition to, or are shielded by, the membrane interface, or when the compartment lumen differs from the bulk solution in pH or ionic strength.[4-10]"

We also added the following citations to this statement:

- Wang, W., Xin, X., Zhang, M., Li, X., Zhao, G., & Bai, W. D. (2024). Improving physicochemical characteristics and cytotoxicity of baicalin esters by liposome encapsulation. Journal of Microencapsulation, 41(4), 312–325. https://doi.org/10.1080/02652048.2024.2348462
- Todd ZR, House CH. Vesicles Protect Activated Acetic Acid. Astrobiology. 2014;14(10):859-865. doi:10.1089/ast.2014.1185
- Anna Grochmal, Luba Prout, Robert Makin-Taylor, Rafel Prohens, and Salvador Tomas Journal of the American Chemical Society 2015 137 (38), 12269-12275 DOI: 10.1021/jacs.5b06207
- Woods B, Thompson KC, Szita N, Chen S, Milanesi L, Tomas S. Confinement effect on hydrolysis in small lipid vesicles. Chem Sci. 2023 Feb 15;14(10):2616-2623. doi: 10.1039/d2sc05747f. PMID: 36908967; PMCID: PMC9993861.
- Alim A. Fatah and L. M. Loew, The Journal of Organic Chemistry 1983 48 (11), 1886-1890, DOI: 10.1021/jo00159a020
- Trishool Namani and Peter Walde, Langmuir 2005 21 (14), 6210-6219, DOI: 10.1021/la047028z

**Reviewer 2, Comment 6.** There could be other ways to allow for molecule transfer between vesicles, e.g. Rubio-Sanchez et al. 2021 (https://pubmed.ncbi.nlm.nih.gov/34597506/)

We modified this sentence as follows and added the mentioned reference:

> "wherein greater diffusion of small molecules through the membrane or other transport mechanisms were likely necessary in the absence of highly evolved specific membrane transporters.[14]"

**Reviewer 2, Comment 7.** May want to consider the findings from Cohen et al. (2022), Cohen et al. (2024) that vesicles can retain some functional properties when in the gel phase or in salty waters.

Thanks for bringing this to our attention. For better accuracy, we modified the relevant sentence in the manuscript and added the two citations as follows:

> "By themselves, fatty acids suffer from poor physical stability, which may affect their robustness as protocells. However, this fragility can be attenuated under certain conditions, such as in the gel phase[33] or through cooperative aggregation in the presence of other building blocks that help to stabilize fatty acid vesicles.[20,34]."

**Reviewer 2, Comment 8.** citations for this? "The prebiotic molecular inventory was highly rich in short- to medium-chain fatty acids (up to 10-12-carbon long chains are considered abundant) as well as other simple organic molecules such as hydroxy acids (HAs)."

We modified the sentence as follows:

> "While most abundant prebiotic fatty acids are commonly short (up to C12-C15),[26,27]…"

The following citations were added to this statement:

1. McCollom, T. M., Ritter, G. & Simoneit, B. R. T. Lipid Synthesis Under Hydrothermal Conditions by Fischer- Tropsch-Type Reactions. *Origins of life and evolution of the biosphere* **29**, 153-166 (1999). https://doi.org/10.1023/a:1006592502746
2. James C.-Y. Lai, Ben K.D. Pearce, Ralph E. Pudritz, Drake Lee, Meteoritic abundances of fatty acids and potential reaction pathways in planetesimals, Icarus, Volume 319, 2019, Pages 685-700, ISSN 0019-1035, https://doi.org/10.1016/j.icarus.2018.09.028.

**Reviewer 2, Comment 9.** were they mixed as aqueous solutions and then allowed to dry? If so, what volume of water was used initially and how long did it typically take to evaporate?

After reading the entire paper and SI, I'm pretty sure this is just two powders being mixed together with no aqueous dissolution at all. Could you make that abundantly clear here, if that is the case?

The reviewer is correct: We did not prepare aqueous stock solutions and did not mix the reagents in water prior to heating. All reagents were used as received under neat (solvent-free) conditions. Specifically, decanoic acid, glycolic acid, malic acid, and phenyllactic acid were used as solids. Lactic acid was obtained as a concentrated solution (≥ 85 wt%) and therefore was used volumetrically as liquid. Thus, no water was added, and any water present originated only from the starting material itself. This was also clarified in the Results and Discussion:

> "Decanoic acid and single HAs (either GA, LA, MA, or PLA) were allowed to react under dry conditions in the absence of water other than water originating in the raw materials for 7 days at 85 °C at a 1:1, 1:2, and 1:4 molar ratio (in favor of the HAs)"

We also added the following to Methods:

> "All reagents were used as received under neat (solvent-free) conditions. "

Please also see response to Reviewer 2, Comment 2.

**Reviewer 2, Comment 10.** Have you tested the "concentration" or mass dependence of the reactions? For example, if the reactants are doubled in mass, are products formed in different ratios, or are additional products formed?

Along the same lines, what were the masses used for these experiments?

To answer this question, we performed new experiments in which we kept the molar ratio of decanoic acid (DA) and lactic acid (LA) constant but changed the batch size. Briefly, when examining the effect of DA:LA molar ratio on DA conversion across different batch sizes, we observed similar trends for 200 and 400 µmol DA, with conversion increasing as the LA fraction increases. However, for 800 µmol DA, conversion reaches a plateau at a 1:2 molar ratio (DA:LA). In addition, increasing batch size decreases DA conversion, most notably at a 1:4 molar ratio (~55%, 45% and 35% conversion for 200, 400 and 800 µmol, respectively). This trend is consistent with reduced efficient contact between the reactant molecules due to mass transport limitations in the melted mixture. As batch size increases, the effects of mixing, diffusion path length, reaction time, and reaction-vessel dimensions become increasingly important. In general, as the batch size increased, the variety of products decreased, mostly the longest products. This was considerably prominent at 800 and 1000 µmol of DA. That is, in the absence of proper mixing to compensate for the loss of homogeneity, the formation of longer products is compromised.

For a more detailed response, please see response to Reviewer 2, Comment 2.

We added the following to Results and Discussion:

> "When examining the effect of DA:LA molar ratio on DA conversion across different batch sizes, we observed similar trends for 200 and 400 µmol DA, with conversion increasing as the LA fraction increases (Figures S22-23). However, for 800 µmol DA, conversion reaches a plateau at a 1:2 molar ratio (DA:LA)."

We also added Figures S22-S23 to the SI.

Regarding the question about masses used for these experiments, we used fixed amount for decanoic acid (200 µmol) and varied amounts for hydroxy acids (either 200, 400, or 800 µmol). The masses were determined based on the molecular weight and the purity of each material. We clarified this as follows in Methods:

> "DA's amount was fixed at 200 µmol and HAs' amount was adjusted accordingly (either 200, 400, or 800 µmol)."

**Reviewer 2, Comment 11.** is concentration the right word if this isn't occurring in an aqueous solution? "Typically, the number of products and their concentration.."

This sentence refers to the observations obtained from the HPLC analysis. Although the reactions were performed under dry conditions, the samples were dissolved prior to HPLC, so the terminology 'concentration' is appropriate.

**Reviewer 2, Comment 12.** extra space here "...length oligomers **of  up** to 5-6 HA."

Fixed.

**Reviewer 2, Comment 13.** I thought (perhaps mistakenly?) the reactions were carried out in the dry state? Where is the liquid coming from? Or did I misunderstand? "Under reaction conditions, GA remained in the liquid state as well as LA."

The reactions were carried out under dry (no added water) conditions at 85 °C. At this temperature, glycolic acid and lactic acid are liquids because their melting points are ~75 °C and ~53 °C, respectively. On the other hand, malic acid and phenyllactic acid remain solid at 85 °C, consistent with their higher melting point (~135 °C and ~121 °C, respectively). To clarify this, we modified the Results and Discussion as follows:

> "However, MA and PLA remained in the solid state and therefore their mobility was lower, in accordance with their higher melting points (~135 °C and ~121 °C, respectively)."

**Reviewer 2, Comment 14.** is this the right word choice? Maybe surfactant instead? "A possible explanation for the positive effect of DA on PLA oligomerization may suggest that DA serves as a **solvent**, allowing partial solubility of PLA; consequently, PLA molecules became more mobile and thus reacted to a greater extent."

We agree that 'solvent' may be a bit of a confusing term. Our intent was to indicate that molten decanoic acid can form a fluid hydrophobic phase that partially solubilizes PLA, thereby increasing PLA mobility and facilitating oligomerization. We have replaced 'solvent' with 'molten hydrophobic medium' to better reflect the physicochemical situation.

> "A possible explanation for the positive effect of DA on PLA oligomerization may suggest that DA serves as a molten hydrophobic medium, allowing partial solubility of PLA;"

**Reviewer 2, Comment 15.** Is there a way to experimentally test this? "It is possible that the oligomerization also took place at the solid-liquid interface formed by both PLA and DA molecules."

That is an interesting question. While our experiments do not directly localize where oligomerization occurs, we can in principle control the particle size of PLA and examine whether it affects these reactions. Smaller particles have a larger surface area and therefore are expected to promote the reaction and result in greater conversion of the reactant. Another complementary experiment could be the addition of a third liquid component that is immiscible with both DA and PLA, inert (i.e., cannot react with either DA or PLA under the reaction conditions), and able to form a liquid barrier between DA and PLA. In that case, the ternary mixture (DA, PLA, and the inert solvent) should be well mixed and allowed to reach equilibrium (i.e., complete phase separation). Then the mixture can be left to react under similar conditions (i.e., 85 °C). If the reaction between DA and PLA takes place at the interface, then the yields would be expected to decrease. However, testing reaction localization would require dedicated experiments that are beyond the scope of this study.

We have softened the wording in this sentence to 'may have occurred at':

> "It is possible that the oligomerization may have occurred at the solid-liquid interface formed by both PLA and DA molecules."

**Reviewer 2, Comment 16.** What is the concentration of DA-HA lipids in these solutions? "To study how the presence of a DA-HA reaction products affects the structure formation, we rehydrated the reaction products of DA and each of the four HAs in a 50 mM phosphate buffer and adjusted the pH to 6.8, at which vesicles are expected to form."

The concentrations were: 50 mM DA and either 50, 100 or 200 mM HA refereeing to the initial amount prior to reaction. The following sentence was also added to the manuscript:

> "The concentrations of DA and the HAs were 50 mM and either 50, 100, or 200 mM, respectively, referring to the initial amounts prior to the reaction."

**Reviewer 2, Comment 17.** can you be more specific about what types of structures? "As indicated by both turbidity measurements and visual observations, most of the samples exhibited turbidity to some extent, suggesting that **structures** formed upon the rehydration process (Figures S189-S190)."

At this point in the text, turbidity and visual inspection can only indicate the formation of assembled material upon rehydration, but they do not allow us to assign a specific morphology. In the next parts of this chapter, we have characterized the specific morphologies using fluorescence microscopy, bright-field microscopy, dynamic light scattering, and cryo-TEM. We have now indicated that the specific morphologies are characterized later using various techniques. We added the following clarification in the main text:

> "As indicated by both turbidity measurements and visual inspection, most of the samples exhibited turbidity to some extent, suggesting that structures formed upon the rehydration process (Figures S191-S192). The specific structural characteristics of these assemblies are characterized below using fluorescence microscopy, bright-field microscopy, dynamic light scattering, and cryo-TEM."

**Reviewer 2, Comment 18.** You may also consider consulting/citing Wang et al. 2019 https://doi.org/10.1016/j.bpj.2019.01.006

Reference was added.

**Reviewer 2, Comment 19.** It might be more staightforward to always use HA:DA ratio and use higher or lower, rather than flipping this to be the opposite order here.

Thank you for the suggestion. To avoid flipping the order of ratios across the manuscript, we adopted a single convention and now report ratios consistently as DA:HA throughout. Where relevant, we additionally clarify which component is in excess (i.e., whether the formulation is DA-rich or HA-rich).

**Reviewer 2, Comment 20.** fewer? "the turbidity of the reaction products significantly decreased and **less** structures of larger size were observed

You are correct - 'Less' was replaced by 'fewer'.

**Reviewer 2, Comment 21.** Consider doing DLS to measure the size distributions? "..the samples consisting of DA and LA homoesters without DA-LA heteroesters were found to form vesicles of various sizes.."

Thank you for the suggestion. For this revision, we have performed DLS measurements, which were indeed very helpful. Please refer to response to Reviewer 2 Comment 3.

**Reviewer 2, Comment 22.** What form of rhodamine was used? Rhodamine B, Rhodamine 6g, etc?

Thank you for pointing this out. We used rhodamine 6G and have now specified this explicitly throughout the manuscript.

**Reviewer 2, Comment 23.** fraction? "As the DA:LA ratio increased to 1:2 and 1:4, vesicle abundance decreased, consistent with the fluorescence microscopy results and the **portion** of ULVs increased."

Yes - 'portion' was replaced by 'fraction'.

**Reviewer 2, Comment 24.** monomers of what? "Vesicles were also observed in the control samples of fresh **monomers**"

By "fresh monomers" we meant the corresponding control system composed of unreacted decanoic acid and lactic acid (i.e., decanoic acid and lactic acid that were not subjected to the reaction conditions). We clarified this in the manuscript as follows:

> "Vesicles were also observed in the control samples prepared by mixing DA and LA fresh monomers at similar molar ratios, however the nature of these vesicles differed (Figure 4A and 4B)."

**Reviewer 2, Comment 25.** General-ish question: these differences in morphology are visualized by microscopy (I'm guessing, will confirm). How is one confident that they are getting a full understanding of the sample when using microscopy and focusing on certain fields within the entire sample?

That is a good question and relates to the broader issue of how representative microscopy fields-of-view are of the bulk sample. How can we infer anything about the bulk when using microscopy only? We repeated the preparations of the control samples (fresh DA and LA monomers) and the reaction product several times (n> three independent preparations) and conducted the measurements very carefully to ensure reproducibility and minimize sampling bias, as follows:

1. Sample preparation was highly consistent: all components were added in a fixed order, mixing time and shear were within well-defined ranges, pH adjustment was carried out with NaOH only (with the exception of DA fresh control which was titrated with HCl. For this sample, the total NaCl concentration was not greater than 10 mM), and the total moles of NaOH were within a relatively narrow range.
2. Imaging was performed within a narrow window after sample preparation (16-24 hr).

Importantly, although microscopy captures local fields, the same qualitative features and morphology differences were reproducibly observed across multiple independently prepared samples and across multiple fields of view per sample. We also repeated the measurements with a lower Rhodamine 6G concentration to rule out a possible effect of the dye, and the results remained the same. The deformed membranous structures in the freshly prepared samples (at high LA concentration, mostly at DA:LA 1:4 molar ratio) were observed repeatedly.

Overall, the differences between reacted samples and freshly prepared samples were highly prominent across all of our measurements (visual appearance, turbidity measurements, both light- and electron- microscopies, and DLS measurements), for example in the following figure comparing rhodamine 6G staining of fresh vs. dry samples containing DA:LA at a 1:4 molar ratio:

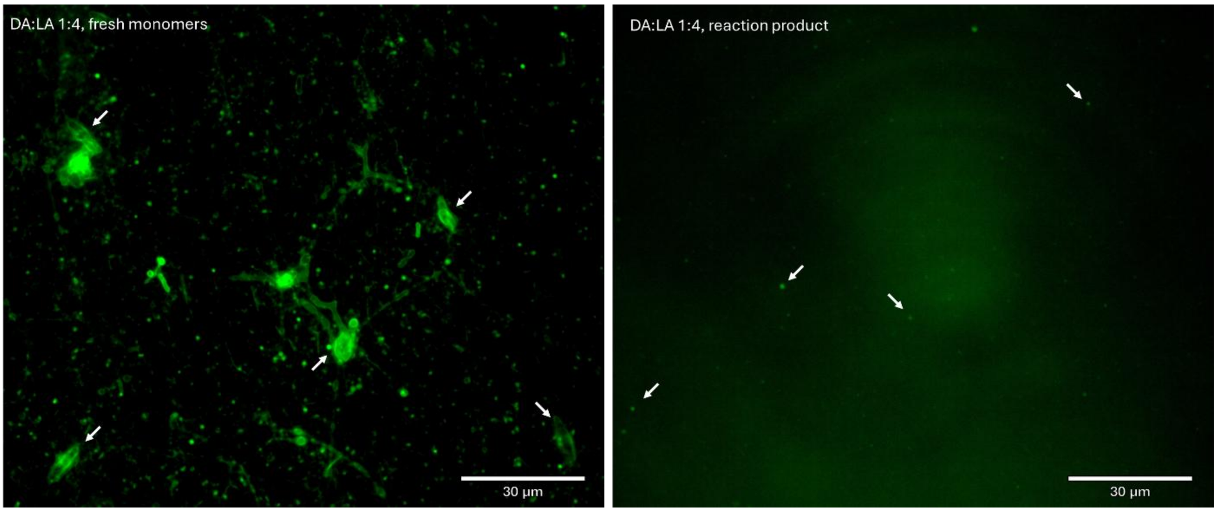


Nevertheless, relying on bright-field microscopy alone is of course not sufficient and complementary methods are required (as we did, using DLS and cryo-TEM).

**Reviewer 2, Comment 26.** Comments on figure 3:

200mM

Corrected (a space was added – 200 mM).

What is rhodamine 6G concentration?

Rhodamine concentration was 1 mM. We have now specified this explicitly in the figure legend.

> "Rhodamine 6G was used as a fluorescent probe (1 mM)."

**Reviewer 2, Comment 27.** Comments on figure 4:

perhaps indicate a few of these with arrows in the figure to guide the reader? "Many small dots, mostly of about 4-6 nm, were also observed, which we attribute to micellar structures."

Great suggestion – we now labelled several micellar structures with black arrows in panel D.

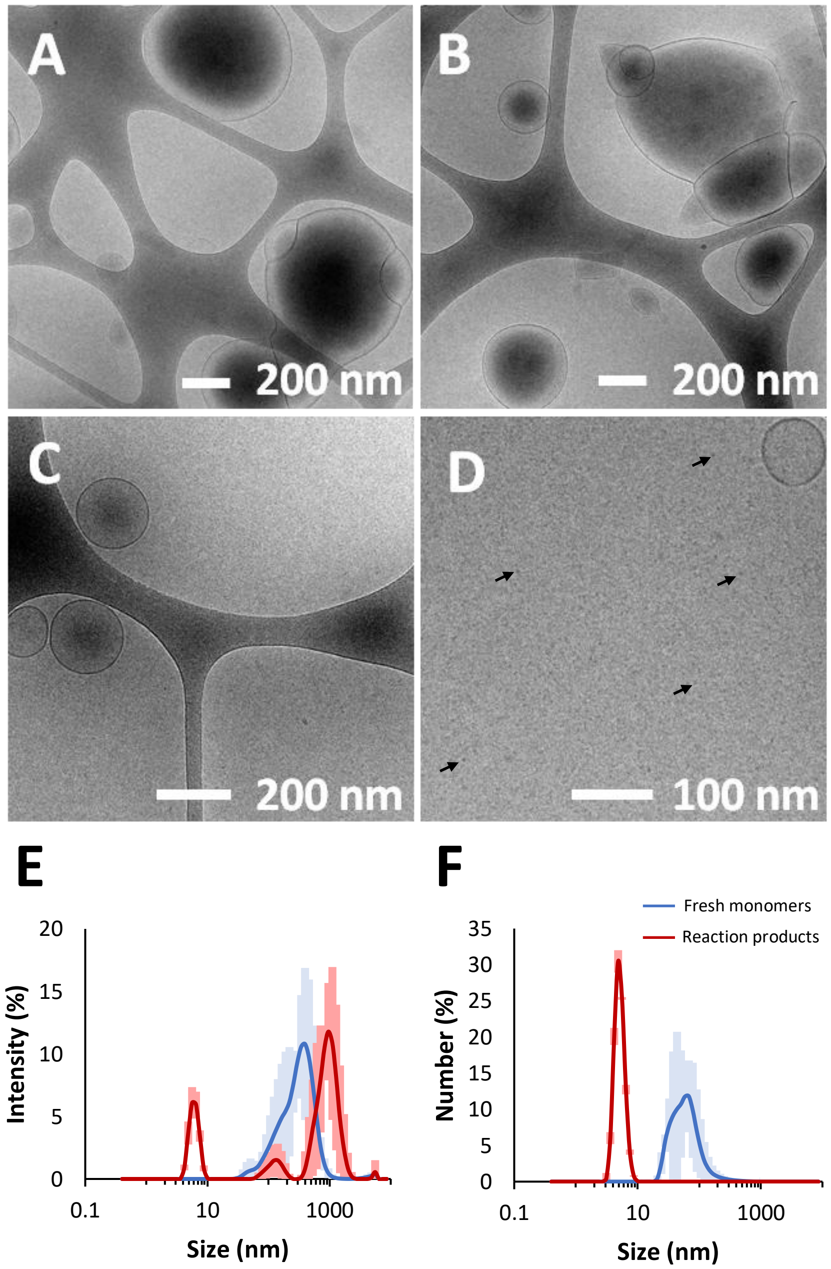


**Figure 1.** Cryo-TEM images of DA:LA fresh monomers (A, B) and reaction products (C, D) at a 1:4 molar ratio. Cryo-TEM images confirm the formation of spherical and ill-defined membranous structures in the fresh monomers. In the reaction products we observed vesicles that were almost exclusively of the unilamellar subtype, and mostly less than 200 nm in size. Many small dots (black arrows), mostly of about 4-6 nm, were also observed, consistent with micellar structures. DLS results (E,F): Intensity-weighted size distribution (E) and number-weighted size distribution (F) obtained for fresh monomers (blue line) and reaction products (red line) at DA:LA 1:4 molar ratio. DA and LA concentrations were 25mM and 100mM respectively. Error bars represent SD of three independent preparations.

**Reviewer 2, Comment 28.** has this been defined previously? If not, do so here. "To test this hypothesis, we measured the **CAC** of DA.."

You are correct - we defined the CAC (critical aggregation concentration):

> "To test this hypothesis, we measured the critical aggregation concentration (CAC) of DA or DA-LA reaction products at pH 6.8."

**Reviewer 2, Comment 29.** Comments on figure 5:

It could be useful to include a legend indicating what the different colors mean. This information is in the caption, but it took me a minute to understand what each color/column corresponded to.

Good idea. We added a legend defining the different groups by color:

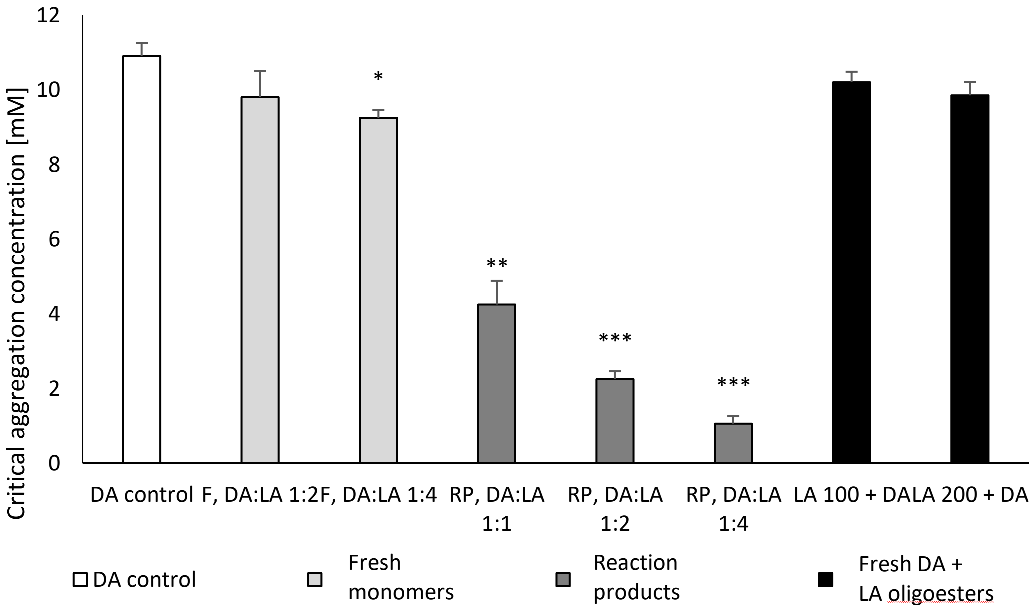


Also, can you mention somewhere how the error bars are calculated? Are these from replicate experiments?

In this case, error bars were calculated based on individual duplicates. We added a sentence to the figure legend and in the Method section about CAC:

> "All experiments were carried out in duplicates."

**Reviewer 2, Comment 30.** molar ratio "To that end, we focused on the reaction product of DA:LA at 1:4 **molar** and studied

Fixed.

**Reviewer 2, Comment 31.** peak areas from LC-MS? "The hydrolysis rate of the products was further determined in terms of product degradation by monitoring the changes in the **peak area** of each product.

We utilized LC-UV/MS that combines HPLC separation, UV absorbance, and mass detection. Peak area obtained from HPLC chromatograms. We clarified this as follows:

> "The hydrolysis rate of the products was further determined in terms of product degradation by monitoring the changes in the peak area of each product in HPLC chromatograms."

**Reviewer 2, Comment 32.** evaporation of water or of DA? If DA, is evaporation the correct word? Perhaps volatilization instead?

We referred to DA. You are right - we changed 'evaporation' to 'volatilization'.

> "Yet the levels of DA were slightly decreased, probably due to minor volatilization of DA over the course of the reaction (Figure S216)"

**Reviewer 2, Comment 33.** give numbered citation as well here? "A study by **Bonfio *et al.*** Achieved

Citation was added.

**Reviewer 2, Comment 34.** pharmaceutical? "...in the food and **pharma** industries."

You are correct. We changed to 'pharmaceutical":

> "applications in the food and pharmaceutical industries.[110,111]"

**Reviewer 2, Comment 35.** Comment on figure 6:

consider indicating the peaks for DA and LA specifically on the chromatograms? This could make interpeting and following (b)-(f) easier for the reader.

This is a good suggestion. We modified Figure 7 (formerly Figure 6) as follows:

- Panel A was reconstructed to enable the identification of LA and DA monomers.
- Panel B (Changes in peak areas of DA and LA monomers) was removed from Figure 7 and added to the SI (Figure S216).

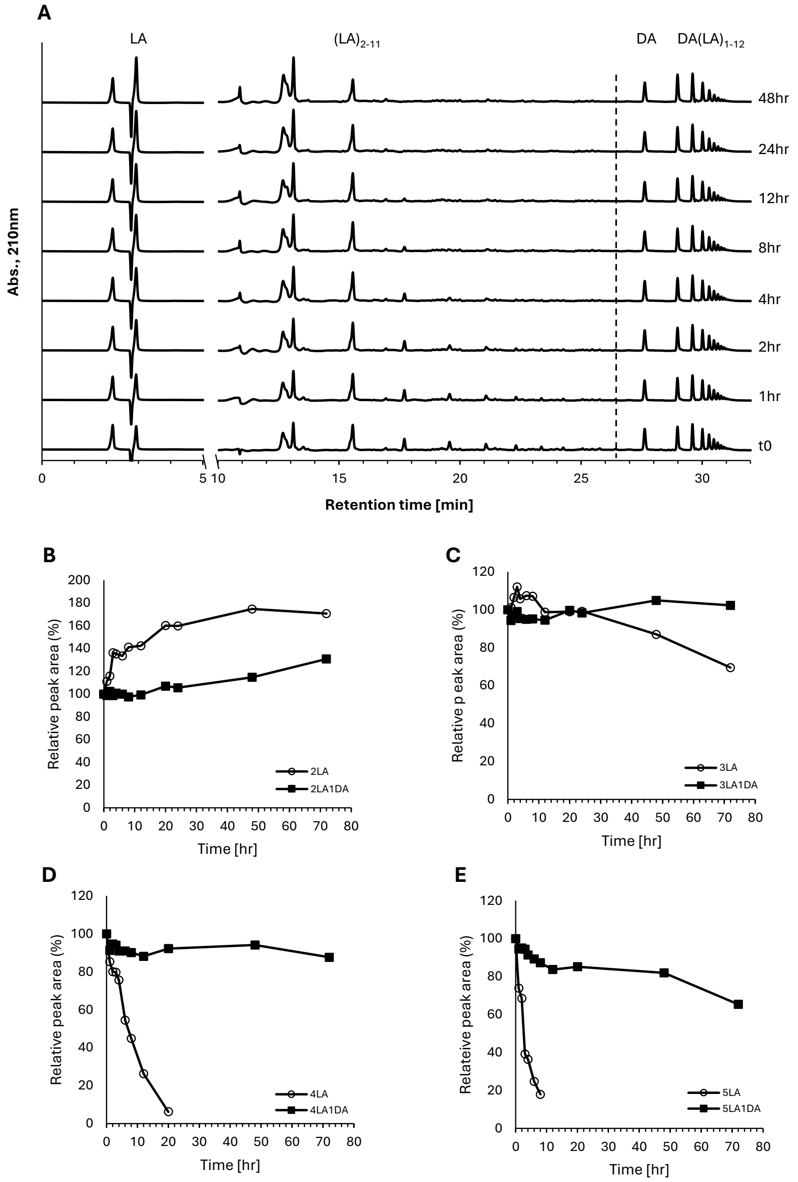


**Reviewer 2, Comment 36.** indicate where in the SI? "Specifically, we performed hydrolysis assays and determination of the CAC for mixtures of either octanoic acid and dodecanoic acid with LA, DA with PLA, and a ternary mixture of DA with both LA and GA **(Supplementary Methods).**

We changed as follows:

> "Specifically, we performed hydrolysis assays and determination of the CAC for mixtures of either octanoic acid and dodecanoic acid with LA, DA with PLA, and a ternary mixture of DA with both LA and GA (Supplementary Methods and Figures S233-S248)."

**Reviewer 2, Comment 37. ["Single-step dry reactions.** Binary mixtures .... **Similar** procedure"]

a) Just to clarify - these reactions were done completely in the solid state? Both reactants were added as solids? Or was there any dissolution/aqueous addition?
b) "A similar"

a) Yes. All components were weighed in the reaction vial except for LA which was obtained as a concentrated solution (>= 85wt%) and therefore was used volumetrically. This was also clarified in Methods:
"All reagents were used as received under neat (solvent-free) conditions."

Please also see the responses to Reviewer 2, Comments 2 and 9.

b) Fixed

**Reviewer 2, Comment 38. [Samples rehydration and structure reconstitution]**

a) What was the final concentration of phosphate and NaOH in the reconstituted solutions?
b) Aha, here it is. Maybe move this up earlier when mentioning the rehydration for clarity?
c) Were these stationary or shaking/tumbling?

a-b) Exactly, the phosphate concentration was specified later in the paragraph. We have now moved it earlier, as suggested. As for NaOH concentration, it was not constant but varied depending on the hydroxy acid and DA:HA molar ratio (in the range of 40-210 mM).

c) The samples were left at RT without any shaking or tumbling. We have clarified this in the manuscript as follows:

> "All samples (reaction products and controls) were kept stationary at room temperature for 16-24 hr prior to characterization."

**Reviewer 2, Comment 39.** [Methods section: **Hydrolysis and degradation experiments**] Missing space "...preparation and after **12hr** and 4 days."

A space was added (12 hr).

**<u>Reviewer #3 (Remarks to the Author):</u>**

**Reviewer 3, Comment 1.** The authors explore a main class of condensation product arising from mixtures of hydroxy acids and fatty acids as a model system representing the condensation products for amphiphiles with polymer-forming building blocks. The most interesting species to emerge are the heteroesters of lipid-hydroxy acid, where the presence of the hydroxy acid oligomer decreases the critical aggregation of the self-assembled structures, and the presence of the self-assembled lipid structures protects the hydroxy acid oligomers from hydrolysis.

We are pleased that the reviewer appreciated the novelty of our work, and we thank them for their constructive comments, which we believe have strengthened the manuscript.

**Reviewer 3, Comment 2.** The key missing insight is whether this heteroester species is actually useful - does the presence of the oligomer alter (improve/compromise) the encapsulation properties and/or permeability of the self-assembled structures? Does the presence of the lipid alter whether hydroxy acid oligomers conserve their functon(indeed, this is a major missing point - what is the function and thus what is the purpose of preventing hydrolysis of hydroxy acid polymers?) I could imagine that such lipids might nucleate the formation of hydroxy acid droplets (e.g. refs 38-40) at the membrane interface, which could lead to a functional difference from just a fatty acid membrane encapsulating a condensate. With these additional insights, this paper would be much more impactful.

These are important questions, and we addressed them in three main ways. First, we performed new experiments to directly test the "usefulness" of the heteroester-containing assemblies, in terms of (i) encapsulation (calcein, labelled ssDNA, and labelled poly-L-lysine) and (ii) permeability measurements, which show that DA–LA reaction products retain encapsulation capability and exhibit higher permeability than DA controls/fresh monomers, consistent with their smaller assemblies. Second, regarding the function of these lipid-conjugated oligoesters, we clarify that under our preparation conditions, we did not observe HA microdroplets or vesicle-droplet coexistence, and we now explicitly discuss hierarchical compartmentalization as an important future direction. Finally, we expanded the Discussion to explain why slowing ester hydrolysis can be functionally meaningful in prebiotic contexts, particularly for depsipeptide chemistry and peptide-bond enrichment - and we frame the coupled benefits to both lipid assembly (lower CAC) and oligomer persistence as a form of chemical mutualism. Below, we address each point in turn.

Regarding the question about "usefulness", we conducted encapsulation experiments with different dyes as well as permeability tests. All experiments were carried out in triplicates (three independent preparations). For that purpose, we used calcein, a hydrophilic fluorescent dye, and prepared samples (DA control, fresh monomers of DA:LA at a 1:4 molar ratio, and reaction product of DA:LA at a 1:4 molar ratio) with 5 mM calcein. To remove free calcein, we used size exclusion chromatography (SEC; SepharoseTM 4B). We collected ten fractions of ca 0.5 mL and measured their fluorescence (see figure below). The third, fourth, and fifth fractions were considered to contain encapsulated calcein, as was also confirmed by fluorescence microscopy:

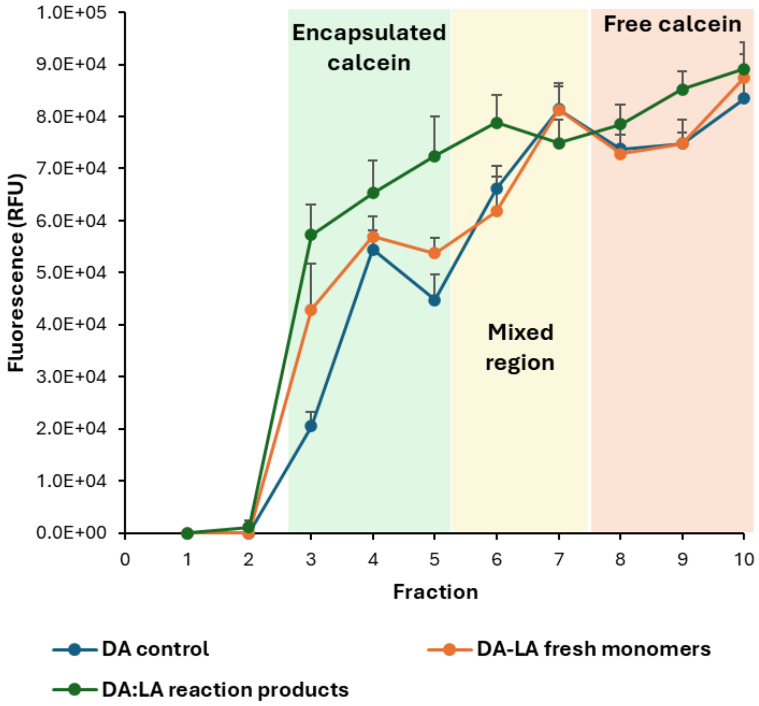


The encapsulation was successful and clearly observed for all three samples, as indicated in the figure below:

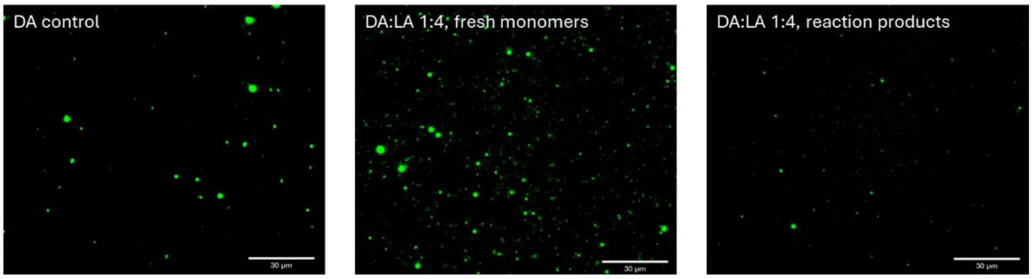


As another direction to show usefulness/function, we also examined the encapsulation of biologically relevant molecules in the novel DA-LA reaction product system. We used labelled ssDNA and labelled Poly-(L)-Lys for that purpose. As indicated by the figure below, labelled-DNA (5'-|56-FAM/CGCTAAATCG-3', Mw 3549.5 g/mol) was encapsulated into the vesicles, even at a very low concentration of ssDNA (20µM prior to isolation of encapsulated fraction via SEC).

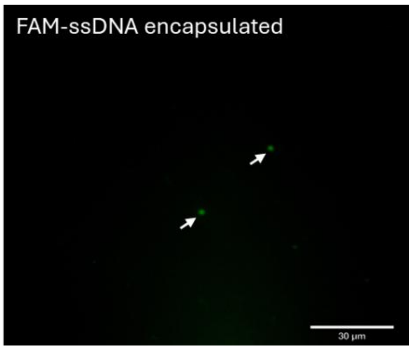


In the case of FITC-labelled poly-(L)-Lys (~ 42 kDa), upon pH adjustment, precipitate formation was observed, and the sample was therefore centrifuged. Nonetheless, the supernatant was examined under a fluorescence microscope, and while aggregates

were observed (labelled with red arrows), we could also detect spherical structures (white arrows), which may correspond to vesicles. As the labelled poly-(L)-Lys is positively charged, it is possible that instead of being encapsulated, it was adsorbed onto the surface of the vesicles via electrostatic interactions with the negatively charged DA-LA oligoesteres.

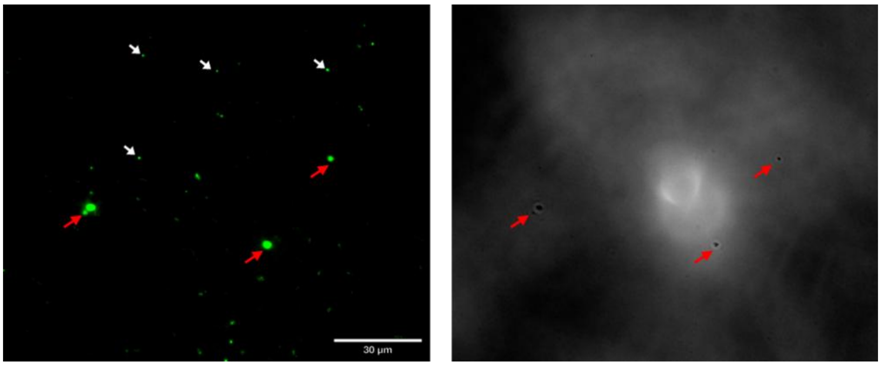


We also conducted permeability experiments (in triplicates) testing the three samples: DA control, fresh monomers of DA:LA at a 1:4 molar ratio, and reaction products of DA:LA at a 1:4 molar ratio. For that purpose, we used the encapsulated calcein (fractions 3-5) of all three samples and measured the samples' fluorescence in the absence and presence of Triton x-100 (final concentration of 0.3% (wt/vol)) over a period of 48 hours. Then calcein encapsulation percentage was calculated according to the following equation (Sahai *et al.*, Scientific reports, 2017):

$$\%\ encapsulation = \left(1 - \frac{F_t - F_0}{F_{Tx} - F_0}\right) \times 100\%$$

In that assay, the encapsulation percent is inversely related to the permeability. That is, the larger the encapsulation percent, the less permeable the vesicles are. The figure below demonstrates calcein encapsulation percent over time:

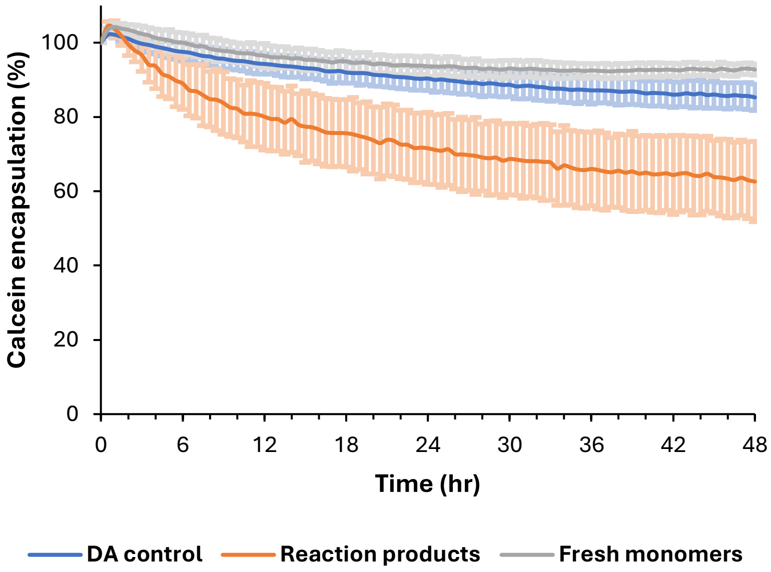


As indicated by the figure, while DA control vesicles and DA-LA fresh monomers exhibited comparable permeability, DA-LA reaction products exhibited greater permeability, as indicated by the lower encapsulation percent. Despite the relatively

large errors, this trend (lower encapsulation percent for DA-LA reaction products) was reproducible and repeated in every independent experiment. These results are consistent with the size distribution determined for each sample. In general, higher membrane curvature in smaller vesicles is expected to increase permeability; thus, the enhanced permeability of the reaction products is consistent with their enrichment in smaller structures. Notably, based on DLS and Cryo-TEM measurements, we know that the reaction products contain both vesicles and micelles. Therefore, it is possible that the measured fractions also contained both vesicles and micelles. Micelles provide a reservoir of amphiphiles, with rapid exchange between assemblies and the surrounding solution, which can destabilize vesicle membranes in a detergent-like manner and promote calcein release from the vesicles.

As discussed in the Introduction, enhanced permeation of small molecules across primitive membranes, or other non-specific transport mechanisms, were likely important during chemical evolution in the absence of highly evolved, selective membrane transporters, further highlighting the potential significance of such lipid-conjugated oligoesters.

The following section was added in Results and Discussion:

> "**Permeability and encapsulation by lipid-conjugated oligoesters.** Cellular permeability is a critical prerequisite in order to promote Darwinian evolution. While passive diffusion through the cell membrane is limited to certain molecules, most of the biologically relevant building blocks require active transportation. Under prebiotic conditions, encapsulation is feasible and almost exclusively relies on transitions in the physical state of the lipids.[93-94] We studied the encapsulation capabilities of our DA-LA lipid-conjugated oligoester products compared to the corresponding controls, and further evaluated the permeability using calcein leakage assay.[95] Encapsulation of calcein (5 mM), a hydrophilic fluorescent dye, was carried out for DA control and DA:LA fresh monomers and reaction products at a 1:4 molar ratio. We confirmed calcein encapsulation using both fluorescence intensity measurements and fluorescence microscopy (Figures S212A-F). Indeed, we observed encapsulation in all tested systems. Next, we carried out a leakage assay using Triton X-100, a non-ionic surfactant that increases vesicle permeability.[96,97] To that end, we measured the fluorescence of the samples in the absence or presence of Triton X-100 and determined the encapsulation percent of calcein over a period of 48 hr. Higher permeability is correlated with a smaller encapsulation percent.[98] As demonstrated by Figure 6 and Figure S212G-I, the leakage of calcein from DA-LA reaction products was greater compared to DA control and DA-LA fresh monomers. This is probably due to the differences in vesicle characteristics, including size, curvature, membrane packing, and membrane thickness.[99,100] The vesicles observed in DA-LA reaction products were considerably smaller compared to the control samples (Figures S200-S203). As vesicle size decreases, its curvature increases and as a result, the permeability through the membrane increases. Another possible explanation for the observed differences is the presence of micelles, in addition to vesicles, in the calcein encapsulated vesicles of the reaction products, which are not present in DA control and DA:LA fresh monomers. We also studied the encapsulation of biologically relevant molecules, specifically 56-FAM-ssDNA (Figure 6B) and poly-(L)-Lysine, within DA:LA reaction products at a 1:4 molar ratio (Figure S213)."

We now added Fig. 6 as follows:

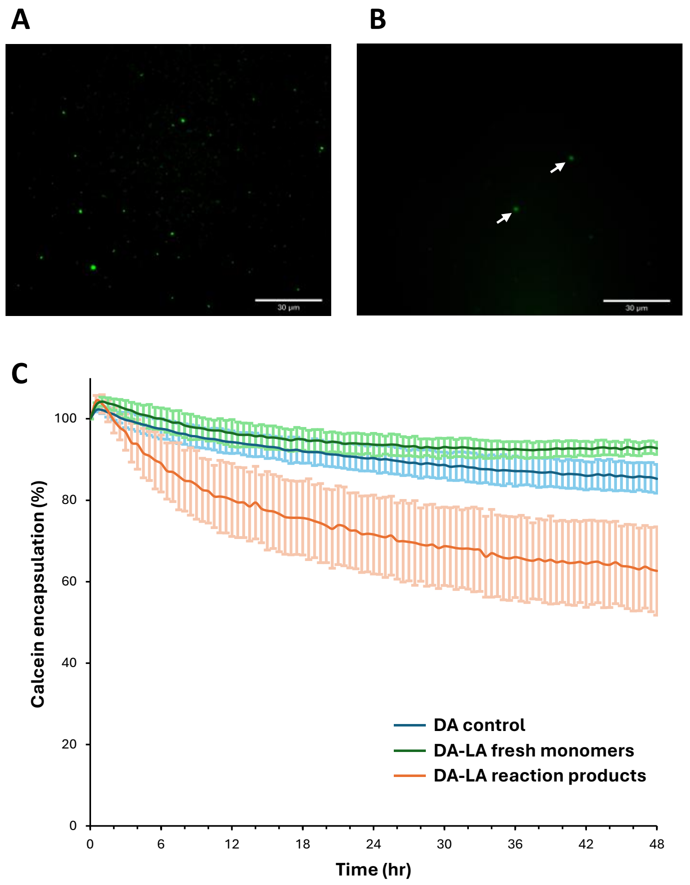


**Figure 2.** DA-LA reaction products exhibit encapsulation capabilities and greater permeability compared to DA control and DA-LA fresh monomers. Encapsulation of the hydrophilic dye calcein (A) and 56-FAM-ssDNA (B) within DA-LA reaction products. Calcein leakage expressed as encapsulation percent as a function of time obtained for DA control samples (blue line), DA:LA fresh monomers at a 1:4 molar ratio (orange line), and DA:LA reaction products at a 1:4 molar ratio (C). Calcein encapsulation percent represents vesicle permeability. As indicated in the figure, calcein leakage from DA-LA reaction products was greater compared to both DA control and the DA-LA fresh monomers, suggesting that vesicles formed in the reaction products are more permeable compared to the unreacted monomers.

The following text was added in Methods:

**Calcein encapsulation and leakage.** Encapsulation experiments were carried out using clacein (a hydrophilic dye). Samples were prepared as described in the above-mentioned sections. The only exception was that calcein (80 mM solution, 167 mM NaOH) was added to the DA prior to the addition of either LA or phosphate monobasic solutions in order to introduce it to the lipid phase prior to the formation of vesicles. Calcein final concentration was 5 mM. Samples were left at RT overnight prior to isolation of the encapsulated calcein. The isolation of calcein-encapsulated vesicles was achieved by size exclusion chromatography (SEC) over Sepharose$^{TM}$ 4B beads. Sepharose columns were prepared in 5 mL disposable syringe into which 5 mL Sepharose beads were added. The mobile phase used for the separation was a vesicular system of 30 mM DA in 50 mM phosphate buffer at pH 6.8. The column was first washed with 50 mM phosphate buffer at pH 6.8 following by equilibration with the mobile phase (about 3 column volumes). Then, 0.5 mL samples were loaded into the column and 10 fractions of 0.5 mL were collected. To confirm the encapsulation, the collected fractions were examined under a fluorescence microscope (FITC filter, EX: 470/40, EM: 525/50, DM: 495). In addition, fluorescence measurements were carried out using a Synergy H1 plated reader at Ex: 490/10, Em 520/10. Calcein-encapsulated fractions were further used for the leakage assay

Leakage assay was carried out as previously described,[95] with minor modifications. Briefly, the third, forth and fifth fractions (i.e., the calcein-

encapsulated fractions) obtained from SEC separation were mixed together. Then, the mixture was divided into two. Triton X-100 was added to one of the aliquots at a final concentration of 0.3% (wt/vol). In addition, control samples of 0.025 mM calcein in either 50 mM phosphate buffer or mobile phase were prepared similarly with or without Triton X-100. Additional control was prepared using the eighth, ninth, and tenth fractions obtained from the SEC, which are the free calcein fractions. All samples were placed in 96-wells black plate covered with sealing tape, and the fluorescence was measured (Ex: 490/10, Em 520/10) over a period of 48 hr in 5 min intervals. The encapsulation percentage of calcein was then calculated according to the formula:

$$\%\ encapsulation = \left(1 - \frac{F_t - F_0}{F_{Tx,t} - F_0}\right) \cdot 100\%$$

Where $F_t$ is the measured fluorescence of the samples without Triton X-100 at time t, $F_0$ is the measured fluorescence of the samples without Triton X-100 at $t_0$, and $F_{Tx,t}$ is the measured fluorescence of the samples with 0.3% Triton X-100 (wt/vol) at time t.

**ssDNA encapsulation.** The encapsulation of ss-DNA (5’-|56-FAM/CGCTAAATCG-3’, Mw 3549.5 g/ mol) was carried out using the reaction product of DA:LA at a 1:4 molar ratio. For sample preparation, the dry reaction product was dissolved in acetonitrile to a final concentration of 100 mM. Then, aliquots of 100 µL (corresponding to 10 µmol DA and 40 µmol LA) were withdrawn and acetonitrile was removed under vacuum at 30°C. Then labeled ss-DNA (100 µM solution) was added to the dry DA-LA mixture following by the addition of phosphate monobasic and sodium hydroxide. The pH was adjusted with sodium hydroxide (1.0 M and 100 mM solutions) to pH 6.8 using an InLab Micro pH electrode (Mettler Toledo). Following preparation, the DNA-encapsulated fraction was isolated using Sepharose™ 4B SEC and the encapsulated fraction was inspected under a fluorescence microscope (FITC filter, EX: 470/40, EM: 525/50, DM: 495).

We also added supplementary Figures S212-213 and the section ‘Poly-Lys encapsulation’ to the Supplementary Methods.

Regarding the point about the function of these lipid-conjugated oligoesters in light of hydroxy acid droplets, we clarify that under our preparation conditions, we did not observe HA microdroplets or vesicle-droplet coexistence. The reviewer is correct that it has been previously reported that hydroxy acid oligoesters can form microdroplets, which by themselves can serve as compartments. Our characterization revealed that under our preparation conditions, there is no co-existence of microdroplets and vesicles. This may be due to several reasons: i) Microdroplet formation in prior reports was observed under specific solvent conditions (e.g., water–acetonitrile mixtures), whereas no cosolvent was used here. ii) The polymerization degree and total concentration of hydroxy acid oligoesters did not reach the critical level required for microdroplet formation. Iii) In general, sample preparation can critically affect the way the system assembles. We did not explore different preparation methods that could result in different arrangements of the lipids and hydroxy acid polyesters. For example, it is possible that encapsulation of microdroplets would take place if microdroplets were formed first and then exposed to the lipids.

Understanding whether coexistence of polyester-based microdroplets and lipid vesicles is feasible and under what conditions is important, but was not in the scope of this work. We added the following clarification to Results and Discussion in the section 'Structural characterization of the reaction products' to address this point:

> "While LA-derived oligoesters have been reported to form microdroplets when dispersed in water-acetonitrile mixtures,[56,59,65] we did not observe microdroplet formation under our conditions, likely due to differences in sample preparation. Nonetheless, further work is needed to explore possible co-existence of lipidic structures such as vesicles and HAs microdroplets. Recently, non-covalent interactions between phospholipids and HA assemblies were reported by Jia and colleagues, in which the assembly of phospholipids around polyphenyllactate droplets has been observed.[76] Moreover, several works studying the interactions of coacervates and lipids have shown that coacervate-containing vesicles enhance both permeability and encapsulation of hydrophilic components, contribute to vesicle uniformity, and alter physicochemical properties of the lipid vesicles.[77-79] Hierarchical compartmentalization and multi-compartment systems are an essential property of living organisms as they construct a complex network of interconnected environments and promote segregated processes.[80-82] Accordingly, concomitant formation of vesicle and microdroplet from simple, prebiotically plausible molecules such as fatty acids and hydroxy acids could provide a tractable model for studying emergent, cell-like compartmentalization."

Last, we expanded the Discussion to explain why slowing ester hydrolysis can be functionally meaningful in prebiotic contexts. Our results show that the conjugation of hydroxy acid oligoesters to lipids protects them compared to free (non-lipid conjugated) oligoesters. Our work also revealed that the same conjugation benefits the lipid as it decreases its CAC. In our view, these coupled effects point to a broader principle in chemical evolution, specifically molecular mutualism (or molecular cooperation), in which distinct chemical subsystems can become mutually reinforcing: the conjugates both stabilize the oligomeric component and promote assembly of the amphiphile component. In this sense, conjugation generates a species that is functionally more stable than its separate, unconjugated components. This conclusion is conceptual, but we view it as an important implication of the data.

Beyond this conceptual insight, we also see potential functional relevance in slowing ester hydrolysis, for example by enabling reactive oligoesters to persist long enough to participate in downstream chemistry at membrane interfaces. One potential example is catalysis of peptide-bond formation via ester–amide exchange on lipid-associated oligoester surfaces. However, directly testing such catalytic activity and additional functional consequences will be an important direction for future work and is beyond the scope of the present study. The present work establishes the DA-HA heteroester conjugates as a novel model system that couples oligomerization and compartmentalization, and defines its core physicochemical properties as a foundation for future investigations. We added the following to the Discussion in the section 'The universality of cooperation between additional hydroxy acids and fatty acids':

> "The implication of such mutualism may be beyond the mere persistence/stability level. HAs could have played a significant role as prebiotic catalysts,[114] for example in proto-peptide synthesis,[65] and perhaps the existence of oligoesters on lipid surfaces could have catalyzed reactions such as lipid-mediated peptide bond formation.[85] Hence, it is possible that hydrolytic

stability can be harnessed to control and catalyze various chemical reactions on lipid surfaces, possibly even in an aqueous environment."

Here are the minor suggestions:

**Reviewer 3, Comment 3.** - Introduction: it's quite long, and could do with a rewrite to focus on the key background details important to this work. Parts seem incorrect (e.g. first paragraph ignores condensates as a type of compartment), irrelevant (e.g. end of first and third paragraph's discussion about permeability, which isn't addressed in this work, and entire second paragraph).

We thank the reviewer for the constructive suggestion. We have rewritten the Introduction to focus on the background most directly relevant to this study. Key changes include:

- The Introduction has been substantially reorganized to improve focus and flow.
- We now explicitly acknowledge membraneless compartments in addition to lipid membranes as a type of compartment ("In modern biology, the role of compartmentalization is dominantly played by phospholipids,[9] the key building blocks of cell membranes, although other mechanisms of sub-cellular compartmentalization exist, such as membraneless liquid-liquid phase separation condensates.[12]").
- We revised the discussion of permeability: because permeability is now addressed in this work (Fig. 6), we retained a brief, relevant discussion.

**Reviewer 3, Comment 4.** - Typos - pKa should be formatted correctly, there should be spaces between numbers and units

All typos were corrected.

**Reviewer 3, Comment 5.** - Keep ratio order consistent e.g. HA:DA then DA:LA is confusing

For consistency, we changed all instances to either DA:LA or DA:HA notation throughout the manuscript.

**Reviewer 3, Comment 6.** - What is the 'total associative hydrophobicity' (top pg 13)

We modified the phrase as follows:

> "We attribute this effect to the presence of the new DA-LA conjugates that promote aggregation, likely by increasing the overall hydrophobic driving force for association in the system."

**Reviewer 3, Comment 7.** - Pg 14 - 'molar' missing 'ratio'?

Fixed.

**Reviewer 3, Comment 8.** - Ref 41 raises an interesting point, the present result should be discussed more in this context

We elaborated the concept of 'assembly-driven protection from hydrolysis' as follows:

> "The protective effect of self-assembly has been reported previously in the context of chemical evolution.[64] It has been demonstrated that chemical entities capable of self-assembly in aqueous environments are recalcitrant (i.e., protected by assembly) and therefore undergo hydrolysis to a lesser extent due to kinetic trapping associated with the assembled state. That is, molecules capable of self-assembly possess an evolutionary advantage over other molecules lacking self-assembly. Our result underscores the crucial protective

role of self-assembly against degradation in water, which is a universal phenomenon not restricted to any particular assembly mode.[64]”

**Reviewer 3, Comment 9. -** “The universality of mutualism…’ title is confusing, perhaps “Effect extends to other fatty acids”

We changed the title as follows:

“The universality of cooperation between additional hydroxy acids and fatty acids.”

**Reviewer 3, Comment 10.** - Fig 3 - the vesicle images are low magnification and low resolution. Encapsulation of an aqueous dye, followed by dilution, could inform of how viable the self-assembled structures are as vesicles.

We replaced the images in Fig. 3C with higher resolution ones:

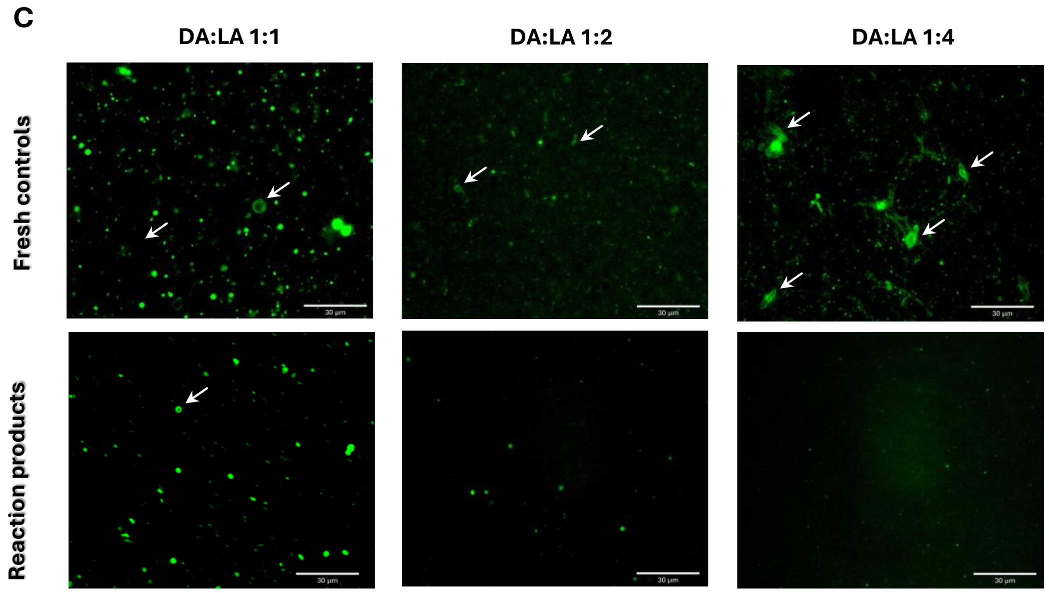


Moreover, as mentioned in response to Reviewer 3 Comment 2, we conducted additional encapsulation tests using hydrophilic dyes, which confirm the formation of vesicles with an aqueous core (new Fig. 6).

**Reviewer 3, Comment 11.** - Fig 4 - “ill-defined” lipid structures. Are they? They seem like vesicles.

We agree that the structures are vesicles. By “ill-defined,” we meant that some vesicles (not all) appear deformed and non-spherical rather than well-defined spherical vesicles. Additional images are provided in the SI (Supplementary Figure S198), and some representative images are shown below:

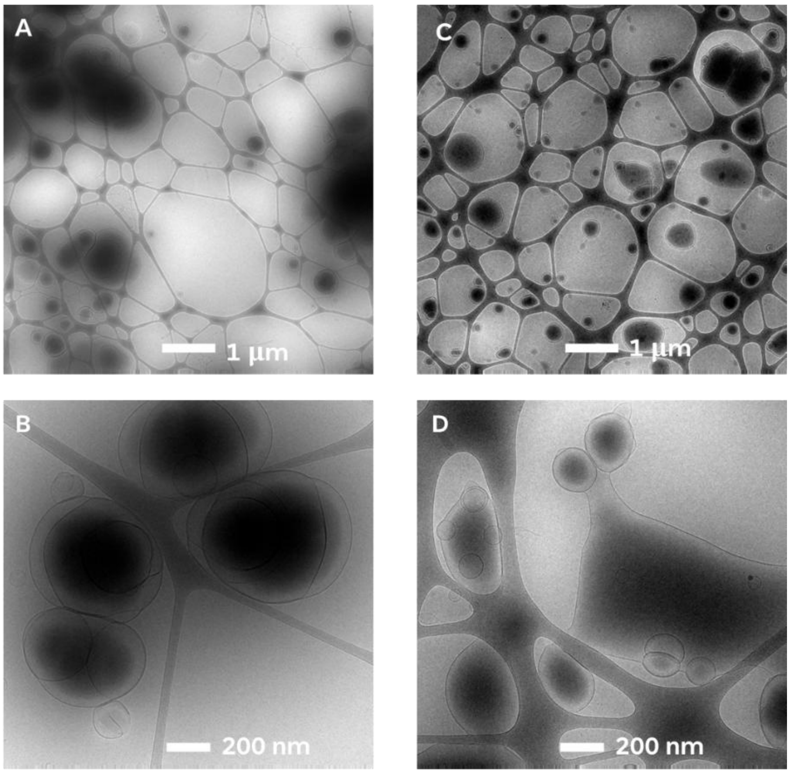


The figure legend was also modified as follows for clarification:

Figure 3. Cryo-TEM images of DA:LA fresh monomers (A, B) and reaction products (C, D) at 1:4 molar ratio. Cryo-TEM images confirm the formation of spherical and non-spherical membranous structures in the fresh monomers. In the reaction products we observed vesicles that were almost exclusively of the unilamellar subtype, and mostly less than 200 nm in size. Many small dots (black arrows), mostly of about 4-6 nm, were also observed, consistent with micellar structures. Intensity-weighted size distribution (E) and number-weighted size distribution (F) obtained for fresh monomers (blue line) and reaction products (red line) at DA:LA of a 1:4 molar ratio. DA and LA concentrations were 25 mM and 100 mM, respectively. Error bars represent SD of three independent preparations.

We also added the following clarification in Results and Discussion:

> "We cannot exclude the possibility that vesicle deformation observed under both fluorescence and electron microscopy might be due to the pressure and compression applied by the microscope cover slip and blotting step upon preparation.[86-88]"

# Response to Reviewers

## Reviewer #1 (Remarks to the Author):

**Reviewer 1, Comment 1.** The authors responded apt to all my queries.

We thank the reviewer for helping us improve the manuscript and are pleased that all concerns have been satisfactorily addressed.

## Reviewer #2 (Remarks to the Author):

**Reviewer 2, Comment 1.** The authors have adequately responded to my comments from the initial version of the manuscript. I think the manuscript is suitable for publication in its current form. I would like to commend the authors for such a detailed and methodical response to these comments, and for a compelling and significant piece of scientific work.

We thank the reviewer for the thoughtful comments on the original version, which helped us strengthen the manuscript. We are happy that the reviewer finds the manuscript suitable for publication in its current form.

## Reviewer #3 (Remarks to the Author):

**Reviewer 3, Comment 1.** I appreciate the substantial additional experimental work undertaken in response to my earlier comments. The new encapsulation and leakage experiments do improve the manuscript and help clarify that the DA-LA reaction products are capable of forming cargo-containing assemblies.

We thank the reviewer for this positive assessment and are pleased that the additional experiments improved the manuscript and demonstrated one possible functional implication of the system we studied.

**Reviewer 3, Comment 2.** I do, however, still have an important methodological concern regarding the calcein assay. I am curious why 5 mM calcein was chosen, since this is typically in a regime where increasing local calcein concentration can reduce fluorescence because of self-quenching. In that case, fluorescence changes may not map straightforwardly onto encapsulated amount. I therefore encourage the authors to comment explicitly on the validity and limitations of the conclusions drawn from this assay, and to moderate the quantitative interpretation accordingly unless further validation is available.

We thank the reviewer for this comment. We agree that the calcein assay should be interpreted comparatively and not as a direct quantitative measure of encapsulation efficiency. We are aware that self-quenching of calcein at high concentrations (above ~3 mM) has been reported in the literature (e.g., Hamann, Steffen, et al. *Journal of fluorescence* 12.2 (2002): 139-145). In selecting the assay conditions, we followed established calcein encapsulation and leakage protocols reported in the literature (e.g., Shimanouchi, Toshinori, et al. *Colloids and Surfaces B: Biointerfaces* 73.1 (2009): 156-160, Sahai, Nita, et al. *Scientific reports* 7.1 (2017): 43418, Dutta, Sayan, et al. *Bio-protocol* 10.14 (2020): e3690-e3690), including concentrations in this general range (20-100mM). However, it is important to note that 5 mM refers to the initial calcein concentration used during vesicle preparation, before vesicle isolation by SEC. While we did not quantify the efficiency of encapsulation (i.e., the concentration of encapsulated calcein), we know based on the analysis of all collected fractions that not all calcein was encapsulated. Therefore, the actual calcein concentration within the purified vesicle-containing fractions is lower than the starting concentration.

Moreover, in the leakage assay, we measured the fluorescence of control samples containing free calcein in phosphate buffer at two concentrations (0.025 mM and 0.050 mM). The fluorescence of the 0.050 mM control sample was comparable to that of the tested samples after addition of Triton X-100 (see graph below representing 24-hr endpoint fluorescence).

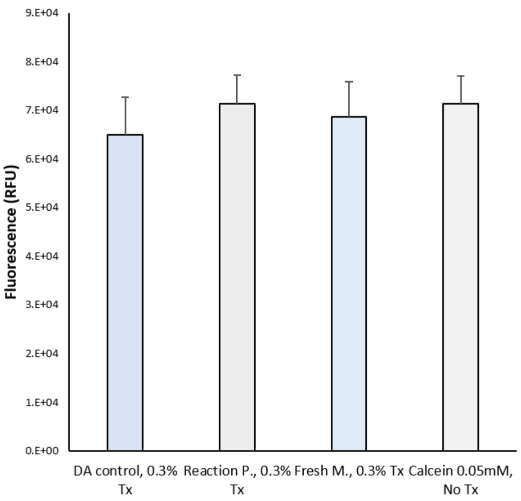


We also conducted a similar experiment using an initial calcein concentration of 2 mM. However, when using 2 mM calcein, the isolated fractions in the presence and absence of Triton X-100 had comparable fluorescence. Under these conditions, the signal difference was too small to provide a reliable readout, likely because the amount of encapsulated dye after purification was below the useful detection range of the assay.

Overall, we considered the potential effect of calcein self-quenching and selected assay conditions under which the fluorescence signal provided a useful comparative readout between the groups of interest.

We clarified this in the text as follows:

> "Encapsulation of calcein (5 mM prior to isolation of encapsulated fractions), a hydrophilic fluorescent dye, was carried out for DA control and DA:LA fresh monomers and reaction products at a 1:4 molar ratio."

> "Because we do not know the exact concentration of calcein inside the isolated vesicle fractions, the calcein assay should be interpreted comparatively and not as a direct quantitative measure of encapsulation efficiency."

**Reviewer 3, Comment 3.** The revised manuscript tends to frame the increased permeability of the reaction products as potentially advantageous in a prebiotic context. At the same time, the authors note that this effect may arise from smaller vesicle size, higher curvature, and/or the presence of micelles that could destabilize vesicles in a detergent-like manner. I therefore encourage a more balanced discussion, since permeability and leakiness are two sides of the same coin, and in DA-based systems an increase in permeability may just as well compromise retention and compartment integrity rather than improve function. In that sense, the observed behavior could reflect a substantial functional cost even if the new species are more persistent.

We agree with the reviewer that increased permeability can have both potential benefits (enhanced encapsulation) and potential drawbacks (increased leakage). We

added the following discussion to the manuscript to explicitly acknowledge this tradeoff:

> "Notably, the increased permeability of the reaction products may represent a tradeoff, potentially facilitating exchange with the environment while at the same time compromising cargo retention and compartment integrity."

**Reviewer 3, Comment 4.** More broadly, I think the manuscript would be stronger if the Introduction and Conclusions were reframed to better articulate why this chemistry matters. At present, the work risks reading mainly as a demonstration that one prebiotically plausible species reacts with another to give mixed products with altered physicochemical behavior, without yet making clear why those changes are important. I do think there are plausible functional consequences worth highlighting more explicitly, even if they remain hypotheses at this stage. For example, lipid-conjugated oligoesters might promote interfacial chemistry at membrane surfaces, might alter uptake-versus-retention behavior in primitive compartments, or might enable more complex hierarchical compartmentalization under alternative preparation conditions. The manuscript would benefit from presenting these as concrete possible consequences of the reported chemistry, while clearly distinguishing them from effects that have actually been demonstrated here.

We thank the reviewer for this comment. We agree that the potential significance of the reported chemistry should be articulated more explicitly. In response, we revised both the Introduction and the Discussion to better explain why these conjugates may matter in a prebiotic context. Specifically, we now emphasize that the reported chemistry does not merely generate mixed products with altered physicochemical properties, but couples two prebiotically relevant processes - amphiphile assembly and oligomer persistence - in a single molecular system. We also expanded the Discussion to highlight concrete possible consequences of this coupling, including altered interfacial chemistry, changes in uptake versus retention behavior in primitive compartments, and the potential for more complex modes of compartmentalization under alternative conditions, while making clear that these are prospective implications rather than effects directly demonstrated here. Specifically, we modified the last paragraph of the Introduction as follows:

> "Here we investigate whether simple reactions between prebiotically plausible fatty acids and hydroxy acids can generate molecular systems in which oligomerization, protection from hydrolysis, and compartmentalization become directly coupled. To that end, we investigated the formation of novel prebiotic amphiphiles obtained by reacting decanoic acid (DA) and additional fatty acids with four different HAs. Under simple drying conditions, we were able to produce a wide variety of lipid products that were characterized via various analytical and structural techniques. Our results indicate that DA is mostly reactive towards lactic acid, although lipid-conjugated oligoesters comprised of DA and HAs were obtained for all tested HAs. We then asked whether these conjugates alter properties that could matter functionally in prebiotic settings. Specifically, we examined how conjugation affects amphiphile assembly, permeability-related behavior, and resistance of hydroxy acid esters to hydrolysis. Our results indicate synergistic interactions and pure molecular mutualism, where, on one hand, conjugation of hydroxy acids to lipids promoted greater self-assembly propensity of the lipids, and on the other hand, this conjugation protected hydroxy acid esters from hydrolysis. Moreover, the resulting lipid-conjugated oligoesters exhibited greater permeability

compared to unreacted DA. Together, these results identify lipid-conjugated oligoesters as a model system in which covalent coupling between two molecular classes gives rise to emergent physicochemical consequences and highlight the importance of cooperative interactions between different classes of molecules in shaping early chemical evolution."

We modified the end of the Discussion as follows:

"In this sense, the conjugates do not simply represent new products, but a molecular system in which assembly propensity and oligomer stability become linked.

The functional implications of coupling between lipid moieties and oligoesters remain prospective and may go beyond the mere persistence/stability level. First, HAs could have played a significant role as prebiotic catalysts,[114] for example in proto-peptide synthesis,[65] and perhaps the existence of oligoesters on lipid surfaces could have catalyzed reactions such as lipid-mediated peptide bond formation.[85] Hence, it is possible that hydrolytic stability can be harnessed to control and catalyze various chemical reactions on lipid surfaces, possibly even in an aqueous environment. Second, HAs and the conjugated lipids described here are individually capable of self-assembly and therefore could support hierarchical compartmentalization where HA droplets could be encapsulated within DA-HA conjugates. Such hierarchical compartmentalization could further be used to support a network of different concomitant processes, each taking place in a different microenvironment. Third, by changing both assembly behavior and permeability-related properties, such conjugates may shift the balance between uptake and retention in primitive compartments. Our results support the idea that oligomerization and compartmentalization could have become coupled during chemical evolution, such that interactions between distinct molecular subsystems generated emergent properties not present in either subsystem alone."

**Reviewer 3, Comment 5.** Finally, regarding fit for Nature Communications, I think this depends strongly on the framing. If the work is presented primarily as an observation that one prebiotically plausible species reacts with another to generate mixed products with altered physicochemical behavior, the broader significance is limited. If, however, the Introduction and Conclusions are reframed to more clearly articulate the possible functional consequences of this chemistry for prebiotic compartment behavior and the emergence of coupled structure-function relationships, then the relevance to the journal's readership would be much stronger.

As described in our response to Reviewer 3 Comment 4, we agree that the broader significance of this work is best conveyed through a clear framing of its possible functional implications. Accordingly, we revised the Introduction and Discussion to more explicitly highlight how this chemistry may affect prebiotic compartment behavior and give rise to coupled structure–function relationships. We believe these revisions strengthen the manuscript's broader relevance to the readership of *Nature Communications*.
